# Annotated catalog and bibliography of the cyclocephaline scarab beetles (Coleoptera, Scarabaeidae, Dynastinae, Cyclocephalini)

**DOI:** 10.3897/zookeys.745.23685

**Published:** 2018-03-22

**Authors:** Matthew R. Moore, Ronald D. Cave, Marc A. Branham

**Affiliations:** 1 Department of Entomology and Nematology, University of Florida, Building 1881 Natural Area Drive, Steinmetz Hall, Gainesville, FL 32611, USA; 2 Department of Entomology and Nematology, University of Florida, Indian River Research and Education Center, 2199 South Rock Road, Fort Pierce, FL 34945, USA

**Keywords:** masked chafers, rhinoceros beetles, catalog, bibliography

## Abstract

Cyclocephaline scarab beetles represent the second largest tribe of the subfamily Dynastinae, and the group includes the most speciose genus of dynastines, *Cyclocephala*. The period following publication of Sebő Endrődi’s *The Dynastinae of the World* has seen a huge increase in research interest on cyclocephalines, and much of this research has not been synthesized. The objective of this catalog and bibliography is to compile an exhaustive list of taxa in Cyclocephalini. This paper provides an updated foundation for understanding the taxonomy and classification of 14 genera and over 500 species in the tribe. It discusses the history of cataloging dynastine species, clarifies issues surrounding the neotype designations in Endrődi’s revision of Cyclocephalini, synthesizes all published distribution data for cyclocephaline species, and increases accessibility to the voluminous literature on the group by providing an easily searchable bibliography for each species. We propose the nomen novum *Cyclocephala rogerpauli*, **new replacement name**, for *C. nigra* Dechambre.

## Introduction

The Cyclocephalini, a group first defined by French naturalist Francis de Laporte de Castelnau in 1840, represents the second largest tribe of the subfamily Dynastinae. *Cyclocephala* Dejean, the type genus of Cyclocephalini, is the most speciose dynastine genus and comprises over 350 species-group taxa as of 2017. The last comprehensive, synoptic treatment of the tribe was *The Dynastinae of the World* (Endrődi 1985a). Endrődi’s foundational book revolutionized the study of the subfamily and paved the way for a veritable explosion of new research into dynastines. This influence is most apparent in the scientific literature covering cyclocephaline scarab beetles. The post-Endrődi era of cyclocephaline research has been marked by ever diversifying interests and approaches to the group. Papers on cyclocephalines now span all modern entomological disciplines from taxonomy, evolutionary biology, ecology, ethology, agronomics, and physiology.

**Figure 1.**
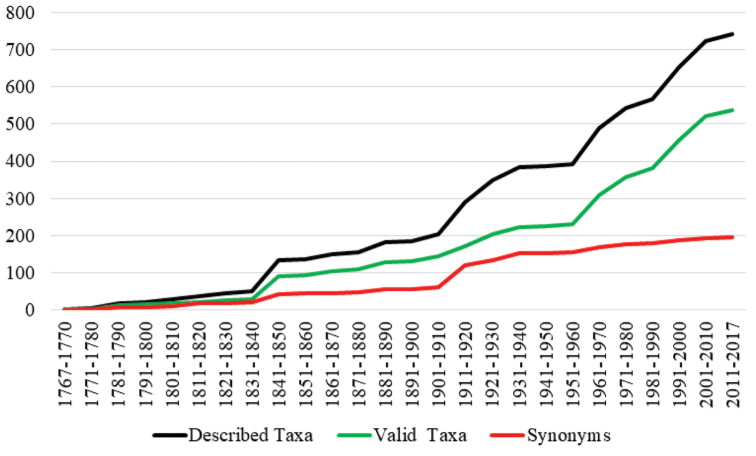
Cumulative number of described cyclocephaline species-group taxa by decade. Species description accumulation was based on the compiled catalog. The synonymy curve also includes names that were homonyms and later replaced.

Ever-growing numbers of cyclocephaline species have, at times, engendered lighthearted dismay among researchers of the group. For example, the Costa Rican *C. unamas* Ratcliffe (Spanish “una mas”) was named after the overwhelming feeling one gets after the discovery of *yet another* new *Cyclocephala* species, epitomized by the species name *C. nodanotherwon* Ratcliffe. Over 170 new cyclocephaline species-group taxa have been described since 1985, and this has created challenges for species identification in several genera. While the most intense period of new species descriptions has probably passed (Fig. 1), many new South American taxa are likely to be discovered, especially in the genera *Cyclocephala* and *Stenocrates* Burmeister (Ratcliffe 2015).

Starting in the mid-1970s, a growing body of research covering the floral ecology of cyclocephalines began to develop. Many faunistic studies in Mesoamerica (especially Mexico) and South America have reported a great deal of cyclocephaline locality data that has yet to be synthesized. Additionally, researchers in the United States and South America have greatly expanded the agronomic literature on the tribe since publication of *The Dynastinae of the World*. This hugely expanded literature for the tribe has not been adequately synthesized and is unwieldy and inaccessible as a result. The objective of this catalog is to: 1) provide an updated foundation for understanding the taxonomic history of 14 genera and over 500 species of cyclocephaline scarab beetles; 2) identify destabilizing issues in the classification and nomenclature of the genera and species; 3) create an easily searchable bibliography to further promote research on these beetles; and 4) synthesize known distribution data for all species in the tribe.

## Brief history of cyclocephalines in catalogs, checklists, and bibliographies

Coleopterists have a long history of compiling species catalogs at a global or regional scale. Prior to the Information Age, these catalogs were invaluable resources for the entomological community because they served to organize biodiversity research. Cataloging the diversity of cyclocephaline species began in Germany with the fourth volume of *Catalogus Coleopterorum Hucusque Descriptorum Synonymicus et Systematicus* (Harold 1869b). This catalog included information on over 120 valid species in the group and provided a brief list of citations for each taxon along with locality information. French entomologists Louis Chevrolat, Albert Fauvel, Auguste Sallé, and Edmond Fleutriaux provided early lists of cyclocephaline diversity in French Guiana (Fauvel 1861), Cuba (Chevrolat 1865), and Guadeloupe (Fleutiaux and Sallé 1889). Gilbert Arrow (1937b) published a comprehensive catalog of Dynastinae in the *Coleoptorum Catalogus* series. Arrow’s catalog featured an updated classification of the subfamily and the tribe Cyclocephalini. His concept of Cyclocephalini was broader than that of later workers, and he included several genera in the tribe that would be subsequently included in Oryctoderini. This new catalog also updated the bibliographic information for each species (Arrow 1937b). Burgeon (1947) followed up Arrow’s work and cataloged dynastine species of the Democratic Republic of Congo and provided an image and redescription of the African cyclocephaline *Ruteloryctes morio* (Fabricius). Friedrich Ohaus and Johann Machatschke published several catalogs of Rutelinae in the mid-20^th^ century in the *Coleopterorum Catalogus* and *Genera Insectorum* series, which at the time included information about the cyclocephaline genera *Peltonotus* Burmeister and *Acrobolbia* Ohaus (Ohaus 1918, 1934b, Machatschke 1972, 1974). Milan Krajcik (2005, 2012) published exhaustive checklists of world Dynastinae and Scarabaeoidea in his Annima.X series.

Many North American Coleopterists created catalogs and checklists that included cyclocephalines. Frederick Melsheimer, Samuel Haldeman, and John LeConte compiled the first catalog of Coleoptera of the United States (Melsheimer et al. 1853). George Horn’s (1894) list of Coleoptera of Baja California included three species of *Cyclocephala*. W. S. Blatchley (1910, 1930) cataloged the Coleoptera of Indiana and created a checklist of scarabs of Florida. Charles Leng, Andrew Mutchler, and Richard Blackwelder compiled enormous catalogs and checklists of beetles, which included cyclocephalines, throughout the New World, including the West Indies (Leng and Mutchler 1914, 1917, Leng 1920, Blackwelder 1939, 1944, 1948). George Wolcott (1923, 1936) assembled annotated checklists of the insects of Puerto Rico and later provided interesting observations of the natural history of Puerto Rican scarabs in other works. Alan Hardy (1991) created a very detailed annotated catalog of Rutelinae and Dynastinae of North America. Andrew Smith created a checklist of all scarabaeoid beetles of the Nearctic Realm (Smith 2009). Stewart Peck, along with collaborators, has cataloged Coleoptera from parts of the West Indies (Peck 2009, 2010, 2016, Peck et al. 2002). Relatively modern checklists or reviews of scarabaeoids have been produced for Canada (McNamara 1991, Bousquet et al. 2013, Ratcliffe and Cave 2017) and portions of the United States including Florida (Woodruff 1973, Peck and Thomas 1998), Maryland (Staines 1984), Nebraska (Ratcliffe 1991, Ratcliffe and Paulsen 2008), South Carolina (Harpootlian 2001), and Texas (Riley and Wolfe 2003).

Checklists of the economically injurious insects of Honduras (Passoa 1983), Nicaragua (Maes and Robleto 1988), Colombia (Posada Ochoa), French Guiana (Remillet 1988), and Suriname (Van Dinther 1960) report unique and fascinating records of cyclocephalines causing damage in agroecosystems. Cyclocephaline floral association data were compiled for the family Araceae (Gibernau 2003, 2011) and for the beetle tribe (Moore and Jameson 2013). Relatively recent checklists focusing on mainland Neotropical scarabaeoid or dynastine taxa have been produced for parts of Mexico (Deloya et al. 2014a, 2016), Nicaragua (Maes 1987, Maes et al. 1997), Panama (Ratcliffe 2002a), Colombia (Restrepo et al. 2003, Gasca-Álvarez and Amat-García 2010), Peru (Ratcliffe et al. 2015), and French Guiana (Ponchel 2011). Regional checklists of Dynastinae were published for Costa Rica and Panama (Ratcliffe 2003), Honduras, Nicaragua, and El Salvador (Ratcliffe and Cave 2006), Mexico, Guatemala, and Belize (Ratcliffe et al. 2013), the West Indies (Ratcliffe and Cave 2015), and the United States (Ratcliffe and Cave 2017). An updated catalog of *Stenocrates* was provided by Ratcliffe (2015), and Dupuis (2017) added details about *Stenocrates* in French Guiana. An updated checklist of the *Cyclocephala* of Colombia was provided by Gasca-Álvarez and Deloya (2016). *Cyclocephala* is the only genus in the tribe for which a tailored bibliography has been produced (Pike et al. 1976).

## Materials and methods

All available literature was reviewed for compiling this catalog and bibliography. References to a cyclocephaline genus only were not included in the list of references. Cited references must have used a trackable specific epithet to have been included. The institutional and collection acronyms used throughout the catalog follow Evenhuis (2016) when possible.

**CAS** California Academy of Sciences, San Francisco, California, USA

**CERPE** Coleção Entomológica da Universidade Federal Rural de Pernambuco, Recife, Brazil

**CMNC** Canadian Museum of Nature, Ottawa, Ontario, Canada

**CNC** Canadian National Collection of Insects, Ontario, Ottawa, Ontario, Canada

**BMNH** The Natural History Museum, London, United Kingdom

**FSCA** Division of Plant Industry, Florida State Collection of Arthropods, Gainesville, Florida, USA

**FDPC** Fabien Dupuis Collection, Saint-Chamond, France

**FUJI** Masayuki Fujioka Collection, Tokyo, Japan

**HNHM** Hungarian Natural History Museum, Budapest, Hungary

**ICN** Universidad Nacional de Colombia, Insituto de Ciencias Naturales de la Universidad Nacional, Bogotá, Colombia

**IEE** Institute of Ecology and Evolution, Russian Academy of Sciences, Moscow, Russia

**IEXA** Colección Entomológica, Instituto de Ecología, A.C., Xalapa, México

**IMQC** Insectarium de Montreal, Montreal, Québec, Canada

**INPA** Instituto Nacional de Pesquisas da Amazonia, Colecão Sistemática da Entomologia, Manaus, Amazonas, Brazil

**IREC** Institut de Recherches Entomologique de la Caribe, Pointe-a-Pitre, Guadeloupe (also known as Centre de Recherches Agronomiques Antilles Guyana, Duclos, Petit-Bourg [CRAAG])

**JPVC** J. Pierre Voirin Collection, Le Luc, France

**LEMQ** Ste. Anne de Bellevue, McGill University, Lyman Entomological Museum, Québec, Canada

**MACN** Museo Argentina de Ciencias Naturales “Bernardino Rivadavia”, Buenos Aires, Argentina

**MCMC** Museo de Historia Natural de la Ciudad de Mexico, Distrito Federal, Mexico

**MCZ** Harvard University, Museum of Comparative Zoology, Cambridge, Massachusetts, USA

**MIZA** Museo del Instituto de Zoología Agrícola, Maracay, Venezuela

**MLUH** Zentralmagazin Naturwissenschaftlicher Sammlungen, Martin-Luther Universität Halle-Wittenberg, Halle, Germany

**MNCR** Museo Nacional de Costa Rica, San José, Costa Rica

**MNHN** Muséum National d’Histoire Naturelle, Paris, France

**MNNC** Coleccion Nacional de Insectos, Museo Nacional de Historia Natural, Santiago, Chile

**MUSENUV** Universidad de Valle, Museo de Entomología, Cali, Colombia

**MUSM** Museo de Historia Natural de la Universidad Nacional Mayor de San Marco, Lima, Peru

**MTD** Museum für Tierkunde, Dresden, Germany

**MXAL** Miguel Ángel Morón Collection, Xalapa, Mexico

**MZSP** Museu de Zoologia da Universidade de São Paulo, São Paulo, Brazil.

**NHMB** Naturhistorisches Museum, Basel, Switzerland

**NHRS** Naturhistoriska riksmuseet, Stockholm, Sweden

**NMPC** National Museum (Natural History), Prague, Czech Republic

**NSMT** National Science Museum (Natural History), Tokyo, Japan

**QSBG** Queen Sirikit Botanic Garden, Chiang Mai, Thailand

**RIEB** Research Institute of Evolutionary Biology, Tokyo, Japan

**RPDC** Roger-Paul Dechambre Collection, Paris, France

**SDEI** Senckenberg Deutsches Entomologisches Institut, Müncheberg, Germany

**UCDC** UCDC

**UNSM** University of Nebraska State Museum, Lincoln, Nebraska, USA

**USNM** National Museum of Natural History, Washington, District of Columbia, USA

**UUZM** Uppsala University, Uppsala, Sweden

**UVGC** Universidad del Valle de Guatemala, Colección de Artrópodos, Guatemala City, Guatemala

**WADA** Kaoru Wada Collection, Tokyo, Japan

**ZMH** Zoologiska Museum, University of Helsinki, Helsinki, Finland

**ZMHB** Museum für Naturkunde der Humboldt-Universität, Berlin, Germany

**ZMUC** University of Copenhagen, Zoological Museum, Copenhagen, Denmark

**ZMUH** Universität von Hamburg, Zoologisches Institut und Zoologisches Museum, Hamburg, Germany

**ZMUK** Universität Kiel, Zoologisches Museum, Kiel, Germany

**ZSMC** Zoologische Staatssammlung des Bayerischen Staates, Munich, Germany

A special note must be made about the type specimen housing institution reporting herein. For older literature, the remarks on type depositories relied on Endrődi’s (1966) explanations. However, many private collections (or portions of collection holdings) have changed hands in the intervening period. This is most relevant for the Antonio Martínez Collection, Sebő Endrődi Collection, Henry and Anne Howden Collection, Frey Collection, and for some Fabrician types. These collections contain a significant number of primary type material for cyclocephaline species. Holotypes deposited in the Martínez Collection should be at MACN. Holotypes and invalid neotypes deposited in the Endrődi Collection should be at HNHM. Holotypes deposited in the Howden collection should be at CMNC. Holotypes deposited in the Frey Collection should be at NHMB. Fabrician lectotypes designated by Endrődi (1966) were originally at ZMUK, but they should now be at ZMUC.

Most of Endrődi’s (1966) lectotypes were clearly designated. However, the types of many species (especially Arrow and Bates types at BMNH and Casey types at USNM) were not clearly discussed. The original descriptions of these species were not always explicit about the number of specimens in a type series. Endrődi (1966) does not clarify these cases and simply listed that a “Type” was at an institution. Herein, these “Type” specimens were not speculated to be holotypes by monotypy or as parts of a syntype series. Future workers who further examine the original descriptions and the type material will have to make those judgements.

As noted by a few authors, Endrődi’s cyclocephaline neotype designations are invalid on several grounds (Dechambre 1991b, Ratcliffe and Hoffman 2011). Endrődi’s (1966) revision of Cyclocephalini was published after the recently adopted 1964 version of the International Code of Zoological Nomenclature (ICZN 1964). ICZN (1964) Article 75c had six conditions that must have been met by Endrődi for his neotype designations to be valid: 1) a statement of characters for differentiating the taxon for which the neotype was designated (or a reference to such a statement); 2) data and description sufficient so that the neotype can be recognized; 3) explanation for believing that all of the original type material is lost or destroyed and the steps that were taken to determine this was the case; 4) explanation of why the neotype specimen is considered consistent with the original-type material; 5) explanation that the neotype came for as near as possible to the original type-locality; and 6) a statement that the neotype is immediately, or upon publication, the property of a recognized scientific or educational institution that maintains a research collection.

Every one of Endrődi’s (1966) twenty neotype designations variably violates the conditions of ICZN (1964) Article 75c. Endrődi possibly satisfied condition (1) of Article 75c in some cases because his neotype designations were accompanied by detailed descriptions (but not necessarily explicitly stated to be descriptions of the neotype). Condition (2) was violated in every case. For example, he did not describe the labels of any of his neotype specimens, hampering recognition of the neotypes. He sometimes also omitted an explicit statement about the sex of the neotype (though they are presumably male). Usually, Endrődi only made vague statements about his search for type materials, amounting to the fact that he did not find a type. This violated condition (3). He typically did not report where exactly he had searched for the material that he did not find. For example, after his description of *Cyclocephala villosa* Blanchard, Endrődi only stated, “Die Type war trotz sorgfältiger Nachforschung nicht aufzufinden, darum designierte Ich mein einziges Exemplar als Neotype ♂” [“The type was not found despite a careful search, so I designated my only specimen as a neotype ♂”]. In contrast, he went into detail about his search (with the help of Bengt-Olof Landin) for Linnean type material of *C. amazona amazona*.

Endrődi never described why he thought his neotypes were consistent with the original type material, nor did he explicitly mention type locality (violating conditions 4 and 5). He was obviously aware of the concept of type locality as evidenced by his decisions when designating neotypes. For example, he designated a neotype of *C. castanea* (Olivier) from “Surinam”, which is the type locality of this species based on the original description (Olivier 1789). However, he offered no explanation of these concepts. Lastly, all of Endrődi’s neotypes were deposited in his personal collection (now at HNHM). Dechambre (1991b) recognized that deposition in Endrődi’s collection did not satisfy condition (6). Endrődi’s invalid neotypes are listed below, in their original name combinations, and are noted in the catalog. In some cases, Dechambre (1991b) discovered syntypes of these species at MNHN and designated lectotypes. Those lectotypes are noted here where applicable.

***Chalepus luridus* Burmeister**: invalid neotype ♂ at HNHM (Endrődi Collection) (Endrődi 1966). Joly and Escalona (2002a) listed the housing institution as MLUH.

***Cyclocephala concolor* Burmeister**: invalid neotype ♂ at HNHM (Endrődi Collection) (Endrődi 1966). Lectotype ♀ at MNHN (Dechambre 1991b).

***Cyclocephala fulvipennis* Burmeister**: invalid neotype ♂ at HNHM (Endrődi Collection) (Endrődi 1966). Lectotype ♀ at MNHN (Dechambre 1991b).

***Cyclocephala gregaria* Heyne & Taschenberg**: invalid neotype ♂ at HNHM (Endrődi Collection) (Endrődi 1966).

***Cyclocephala nigricollis* Burmeister**: invalid ♂ neotype at HNHM (Endrődi Collection) (Endrődi 1966). Lectotype ♂ at MNHN (Dechambre 1991b).

***Cyclocephala occipitalis* Fairmaire**: invalid neotype ♂ at HNHM (Endrődi Collection) (Endrődi 1966).

***Cyclocephala octopunctata* Burmeister**: invalid neotype ♂ at HNHM (Endrődi Collection) (Endrődi 1966). Lectotype ♀ at MNHN (Dechambre 1991b).

***Cyclocephala putrida* Burmeister**: invalid neotype at HNHM (Endrődi Collection) (Endrődi 1966). Lectotype ♀ of *C. putrida* at MNHN (Dechambre 1991b).

***Cyclocephala rubescens* Bates**: invalid neotype ♂ at HNHM (Endrődi Collection) (Endrődi 1966).

***Cyclocephala signaticollis* Burmeister**: invalid neotype ♂ at HNHM (Endrődi Collection) (Endrődi 1966). Lectotype ♀ at MNHN (Dechambre 1991b).

***Cyclocephala subsignata* Burmeister**: invalid neotype ♂ at HNHM (Endrődi Collection) (Endrődi 1966). Lectotype ♀ at MNHN (Dechambre 1991b).

***Cyclocephala tetrica* Burmeister**: invalid neotype ♂ at HNHM (Endrődi Collection) (Endrődi 1966).

***Cyclocephala villosa* Blanchard**: invalid ♂ neotype at MNHN (Endrődi Collection) (Endrődi 1966).

***Cyclocephala villosa* Burmeister**: invalid neotype ♂ at HNHM (Endrődi Collection) (Endrődi 1966).

***Melolontha castanea* Olivier**: invalid neotype ♂ at HNHM (Endrődi Collection) (Endrődi 1966).

***Melolontha immaculata* Olivier**: invalid neotype ♂ at HNHM (Endrődi Collection) (Endrődi 1966). Chalumeau and Gruner (1977) stated this neotype was at MNHN.

***Melolontha picipes* Olivier**: invalid neotype ♂ at HNHM (Endrődi Collection) (Endrődi 1966).

***Melolontha rustica* Olivier**: invalid neotype at HNHM (Endrődi Collection) (Endrődi 1966).

***Melolontha undata* Olivier**: invalid ♂ neotype at HNHM (Endrődi Collection) (Endrődi 1966).

***Scarabaeus amazonus* Linnaeus**: invalid neotype at HNHM (Endrődi Collection) (Endrődi 1966).

### How to use this catalog

Entries for genera follow a format that tracks the history of genera as either valid or invalid through time. The generic-level entries also include the type species of the genus, a list of references that contain identification keys for the genus and/or its species, and the number of valid species and subspecies in the genus.

The very first line, in bold, is the current valid name of the taxon. Underneath that, the taxonomic history of the name is presented in chronological order. These histories can be extremely complicated and difficult to track. The types of changes included here are: 1) changes in generic classification; 2) changes in subgeneric classification; 3) names that the current valid name may have been synonymized under for a period; 4) revalidation of names as either species or subspecies; 5) changes in status that include movement between specific, subspecific, and infrasubspecific categories. These changes, outlined above, are cited using an abbreviated reference, with pagination, and what actions that author took.

Entries for synonyms are indented and labeled “syn.” in bold. The taxonomic histories of synonyms can be just as complicated as valid names and those details are provided here in the same format as the taxonomic history of the valid name. Information about the primary types, as far as could be ascertained, is provided beneath the taxonomic histories. These include citations for that information and the primary type repository. A generalized distribution from the literature is provided below the type information. Country records are in all capital letters and state/department/commune-level records are given after the country. Lastly, a bibliography of each species is provided in rough chronological order and sorted by author.

Remarks are also given that clarify some data about a taxon where it is applicable. Remarks given here generally relate to conflicting distribution data in the literature or potential minor taxonomic and nomenclatural issues. Because so little is known of cyclocephaline relationships, the catalog is presented in alphabetic order by genus and then species-group taxa, instead of systematic order.

## Annotated catalog and bibliography of the cyclocephaline scarab beetles (Coleoptera, Scarabaeidae, Dynastinae, Cyclocephalini)

### Tribe Cyclocephalini

Taxon classificationAnimaliaColeopteraScarabaeidae

Laporte, 1840

Cyclocephalites Laporte, 1840: 124 [original usage].
Cyclocephalidae [Burmeister, 1847: 21].
Cyclocephalides [Lacordaire, 1856: 393].
Cyclocephalini [LeConte, 1862: 143].
Cyclocephalinae [Bates, 1888: 296].

#### Type genus.

*Cyclocephala* Dejean, 1821.

### Genus Acrobolbia

Taxon classificationAnimaliaColeopteraScarabaeidae

Ohaus, 1912

Acrobolbia Ohaus, 1912: 316 [original usage].

#### Type species.

*Acrobolbia macrophylla* Ohaus, 1912, by monotypy.

#### Keys.

Jameson et al. 2002.

#### Valid taxa.

1 species.

### Acrobolbia macrophylla

Taxon classificationAnimaliaColeopteraScarabaeidae

Ohaus, 1912

Acrobolbia macrophylla Ohaus, 1912: 317–318 [original combination].
**syn.** Acrobolbia triangularis Benderitter, 1922: 147 [original combination].
Acrobolbia macrophylla Ohaus [synonymy by Ohaus 1934a: 14].

#### Types.

Holotype ♂ of *A. macrophylla* at ZMHB (Jameson et al. 2002). Neotype ♂ of *A. triangularis* at UNSM (Jameson et al. 2002).

#### Distribution.

ECUADOR: Napo, Pastaza. PERU: Huanuco, Madre de Dios. VENEZUELA.

#### References.

Lucas 1918a, Benderitter 1922, Ohaus 1912, 1918, 1934a, b, Anonymous 1940, Machatschke 1972, Jameson 1998, Dechambre and Ponchel 1999, Jameson et al. 2002, Breeschoten et al. 2013.

### Genus Ancognatha

Taxon classificationAnimaliaColeopteraScarabaeidae

Erichson, 1847

Ancognatha Erichson, 1847a: 97 [original usage].
Cyclocephala Dejean [synonymy by Lacordaire 1856: 398].
Ancognatha Erichson [revalidated genus status by Bates 1888: 297].
**syn.** Barotheus Bates, 1891: 30–31 [original usage]. Type species: Barotheus andinus Bates, 1891, by monotypy.
Ancognatha Erichson [synonymy by Endrődi 1966: 365].
**syn.** Lissodon Paulian, 1954: 1154 [original usage]. Type species: Lissodon argodi Paulian, 1954, by monotypy.
Ancognatha Erichson [synonymy by Endrődi 1969b: 38].
**syn.** Pseudoancognatha Otoya, 1945: 275 [original usage]. Proposed as a subgenus. Type species: Ancognatha nigriventris Otoya, 1945, by original designation. Ancognatha Erichson [synonymy by Martínez 1965a: 64].

#### Type species.

*Ancognatha scarabaeoides* Erichson, subsequent designation by Casey 1915: 111.

#### Keys.

Saylor 1945 (USA), Arnett 1968 (USA), Endrődi 1966, 1985a, Figueroa and Ratcliffe 2016 (Peru), Morón and Deloya 1991 (Mexico, Durango), Morón-Ríos and Morón 2001 (Mexico, Chiapas), Ratcliffe et al. 2013 (Mexico), Neita-Moreno and Morón 2008 (larvae), Vallejo and Morón 2008 (larvae), Pardo-Locarno et al. 2006, Gasca-Álvarez and Amat-García 2010 (Colombia), Mondaca 2011 (Chile), Villalobos-Moreno et al. 2017 (Colombia), Ratcliffe and Cave 2017 (USA and Canada).

#### Valid taxa.

22 species.

### Ancognatha atacazo

Taxon classificationAnimaliaColeopteraScarabaeidae

(Kirsch, 1885)

Cyclocephala atacazo Kirsch, 1885: 223 [original combination].
Ancognatha atacazo (Kirsch) [new combination by Endrődi 1966: 370].

#### Types.

Lectotype ♀ at MTD (Endrődi 1966).

#### Distribution.

COSTA RICA: Cartago, San José. COLOMBIA: Quindío, Tolima, Valle del Cauca. ECUADOR: Pichincha.

#### References.

Kirsch 1885, Bertkau 1886, Arrow 1937b, Blackwelder 1944, Pike et al. 1976, Endrődi 1966, 1967a, 1985a, Ratcliffe 2003, Pardo-Locarno et al. 2006, Krajcik 2005, 2012.

### Ancognatha aymara

Taxon classificationAnimaliaColeopteraScarabaeidae

Mondaca, 2016

Ancognatha aymara Mondaca, 2016: 60–63 [original combination].

#### Types.

Holotype ♂ at MNNC (Mondaca 2016).

#### Distribution.

CHILE: Arica y Parinacota.

#### References.

Mondaca 2016.

#### Remarks.

Specimens of *A. lutea* reported from Chile (e.g., see Gutiérrez 1950, Mondaca 2011, Ferrú and Elgueta 2011) were later determined to be *A. aymara* (Mondaca 2016).

### Ancognatha castanea

Taxon classificationAnimaliaColeopteraScarabaeidae

Erichson, 1847

Ancognatha castanea Erichson, 1847a: 98 [original combination].
Cyclocephala castanea (Erichson) [new combination by Lacordaire 1856: 398, 399].
Barotheus castaneus (Erichson) [new combination by Arrow 1911: 169].
Ancognatha castanea Erichson [revised combination by Endrődi 1966: 365].
**syn.** Barotheus andinus Bates, 1891: 31 [original combination].
Barotheus castaneus (Erichson) [synonymy by Arrow 1911: 169].
**syn.** Lissodon argodi Paulian, 1954: 1154–1155 [original combination].
Ancognatha castanea Erichson [synonymy by Endrődi 1969b: 38].

#### Types.

Lectotype ♀ of *A. castanea* at ZMHB (Endrődi 1966). Type of *B. andinus* at BMNH (Endrődi 1966). Holotype ♂ at MNHN (Paulian 1954).

#### Distribution.

COLOMBIA: Nariño. ECUADOR: Chimborazo, Napo, Pichincha. PERU: Ayacucho, Cuzco, Lima.

#### References.

Erichson 1847a, Lacordaire 1856, Marschall 1857, Harold 1869b, Bates 1891, Bertkau 1892, Arrow 1911, 1937b, Blackwelder 1944, Paulian 1954, Endrődi 1966, 1969b, 1974, 1985a, Onore 1997, 2005, Pardo-Locarno et al. 2006, Krajcik 2005, 2012, Breeschoten et al. 2013, Ratcliffe et al. 2015, Dossey et al. 2016, Figueroa and Ratcliffe 2016, Mitasuhashi 2016.

#### Remarks.

Lacordaire’s (1856) rejection of *Ancognatha* created a case of homonymy between the names *C. castanea* (Erichson) and *C. castanea* (Olivier). *Cyclocephala peruana* was proposed as a replacement for the junior homonym *C. castanea* (Erichson) (Harold 1869a). Subsequent authors did not use this replacement name (e.g., Arrow 1911, 1937b), with Endrődi (1966) stating that it was an “incorrect” new name.

### Ancognatha corcuerai

Taxon classificationAnimaliaColeopteraScarabaeidae

Figueroa & Ratcliffe, 2016

Ancognatha corcuerai Figueroa & Ratcliffe 2016: 65–67 [original combination].

#### Types.

Holotype ♂ at MUSM (Figueroa and Ratcliffe 2016).

#### Distribution.

PERU: Cajamarca.

#### References.

Figueroa and Ratcliffe 2016.

### Ancognatha erythrodera

Taxon classificationAnimaliaColeopteraScarabaeidae

(Blanchard, 1846)

Cyclocephala erythrodera Blanchard, 1846: 191 [original combination].
Ancognatha erythrodera (Blanchard) [new combination by Arrow 1937b: 6].

#### Types.

Efforts to find type specimens were unsuccessful (Endrődi 1966).

#### Distribution.

ARGENTINA: Tucumán. BOLIVIA: La Paz. PERU: Arequipa, Puno.

#### References.

Blanchard 1846, Erichson 1847b, Harold 1869b, Arrow 1937b, Blackwelder 1944, Endrődi 1966, 1985a, Krajcik 2005, 2012, Breeschoten et al. 2013, Ratcliffe et al. 2015, Figueroa and Ratcliffe 2016.

### Ancognatha falsa

Taxon classificationAnimaliaColeopteraScarabaeidae

Arrow, 1911

Ancognatha falsa Arrow, 1911: 170 [original combination].
Cyclocephala falsa (Arrow) [new combination by Endrődi 1966: 194].
Ancognatha falsa Arrow [revised combination by Endrődi 1985a: 162].

#### Types.

Type ♂ at BMNH (Arrow 1911, Endrődi 1966).

#### Distribution.

MEXICO: Chiapas, Estado de México, Guerrero, Hidalgo, Jalisco, Michoacán, Morelos, Oaxaca, Puebla, Veracruz.

#### References.

Arrow 1911, 1937b, Blackwelder 1944, Pike et al. 1976, Endrődi 1966, 1985a, Ratcliffe and Morón 1997, Pacheco-F. et al. 2008, Múñoz-Hernández et al. 2008, Ramírez-Ponce et al. 2009, Krajcik 2005, 2012, Romero-López et al. 2012, 2015, Ratcliffe et al. 2013, Deloya et al. 2016.

### Ancognatha gracilis

Taxon classificationAnimaliaColeopteraScarabaeidae

Endrődi, 1966

Ancognatha gracilis Endrődi, 1966: 372–373 [original combination].

#### Types.

Holotype ♂ at ZMHB (Endrődi 1966).

#### Distribution.

COSTA RICA: Cartago, Heredia, Limón, San José. PANAMA: Chiriquí.

#### References.

Endrődi 1966, 1985a, Ratcliffe 1992d, 2002a, 2003, Krajcik 2005, 2012.

### Ancognatha horrida

Taxon classificationAnimaliaColeopteraScarabaeidae

Endrődi, 1967

Ancognatha horrida Endrődi, 1967a: 409–411 [original combination].

#### Types.

Holotype ♂ at NHMB (Frey Collection) (Endrődi 1967a).

#### Distribution.

COLOMBIA: Nariño. ECUADOR: Cañar, Loja, Pichincha.

#### References.

Endrődi 1967a, 1985a, Dechambre 2000, Pardo-Locarno et al. 2006, Krajcik 2005, 2012.

### Ancognatha humeralis

Taxon classificationAnimaliaColeopteraScarabaeidae

(Burmeister, 1847)

Cyclocephala humeralis Burmeister, 1847: 40 [original combination].
Ancognatha humeralis (Burmeister) [new combination by Arrow 1914: 274].
**syn.** Cyclocephala longiceps Kirsch, 1870: 354–355 (paginated incorrectly as 370–371) [original combination].
Ancognatha humeralis (Burmeister) [synonymy by Arrow 1914: 274].

#### Types.

Lectotype ♂ of *C. humeralis* at MLUH (Endrődi 1966).

#### Distribution.

BOLIVIA: Cochabamba. CHILE. COLOMBIA: Antioquia, Caldas, Cauca, Cundinamarca, Quindío, Risaralda, Valle del Cauca. ECUADOR. PERU: Lima.

#### References.

Burmeister 1847, Harold 1869b, Kirsch 1870, Bates 1888, Arrow 1911, 1914, 1937b, Blackwelder 1944, Martínez 1965a, Pike et al. 1976, Endrődi 1966, 1985a, Restrepo et al. 2003, Útima and Vallejo 2008, Krajcik 2005, 2012, Breeschoten et al. 2013, Ratcliffe et al. 2015, Figueroa and Ratcliffe 2016.

#### Remarks.

*Ancognatha humeralis* was reported from Panama, Costa Rica, and Venezuela (Bates 1888, Blackwelder 1944). Some of these data probably refer to *A. vulgaris* (see Arrow 1911). Major faunistic studies have not reported *A. humeralis* from Panama or Costa Rica (Ratcliffe 2003).

### Ancognatha hyltonscottae

Taxon classificationAnimaliaColeopteraScarabaeidae

Martínez, 1965

Ancognatha hyltonscottae Martínez, 1965a: 64–70 [original combination].

#### Types.

Holotype ♂ at MACN (Antonio Martínez Collection) (Martínez 1965a).

#### Distribution.

BOLIVIA: Cochabamba.

#### References.

Martínez 1965a, Endrődi 1966, 1985a, Krajcik 2005, 2012.

### Ancognatha jamesoni

Taxon classificationAnimaliaColeopteraScarabaeidae

Murray, 1857

Ancognatha jamesoni Murray, 1857: 230–232 [original combination].
**syn.** Ancognatha crassimanus Murray, 1857: 232–234 [original combination].
Ancognatha jamesoni Murray [synonymy by Endrődi 1966: 374].

#### Types.

Types of both *A. jamesoni* and *A. crassimanus* are at BMNH (Endrődi 1966).

#### Distribution.

ECUADOR: Pichincha.

#### References.

Murray 1857, Gerstaecker 1858, Harold 1869b, Arrow 1937b, Blackwelder 1944, Endrődi 1966, 1985a, Dechambre 2000, Onore 1997, 2005, Krajcik 2005, 2012, Dossey et al. 2016.

### Ancognatha lutea

Taxon classificationAnimaliaColeopteraScarabaeidae

Erichson, 1847

Ancognatha lutea Erichson, 1847a: 97 [original combination].
Cyclocephala lutea (Erichson) [new combination by Lacordaire 1856: 398, 399].
Ancognatha lutea Erichson [revised combination by Arrow 1937b: 6].

#### Types.

Lectotype ♂ at ZMHB (Endrődi 1966).

#### Distribution.

ARGENTINA. BOLIVIA. BRAZIL. COLOMBIA: Bogotá, D. C., Cundinamarca, Santander. GUYANA. PERU: Cajamarca, Cuzco, Lima, Puno. URUGUAY.

#### References.

Erichson 1847a, Lacordaire 1856, Harold 1869b, Arrow 1937b, Blackwelder 1944, Pike et al. 1976, Endrődi 1966, 1985a, Restrepo et al. 2003, Gasca-Álvarez and Amat-García 2010, Ferrú and Elgueta 2011, Mondaca 2011, Krajcik 2005, 2012, López-García et al. 2015, Ratcliffe et al. 2015, Figueroa and Ratcliffe 2016, Mitasuhashi 2016.

### Ancognatha manca

Taxon classificationAnimaliaColeopteraScarabaeidae

(LeConte, 1866)

Cyclocephala manca LeConte, 1866: 382 [original combination].
Ancognatha manca (LeConte) [new combination by Bates 1888: 298].
**syn.** Ancognatha aequata Bates, 1888: 297 [original combination].
Ancognatha manca (LeConte) [synonymy by Arrow 1911: 169].
**syn.** Ancognatha durangoana Casey, 1915: 125 [original combination].
Ancognatha manca (LeConte) [synonymy by Saylor 1945: 125].
**syn.** Ancognatha laevigata Bates, 1888: 297–298 [original combination].

Ancognatha manca (LeConte) [synonymy by Saylor 1945: 379].
**syn.** Ancognatha perspicua Casey, 1915: 126 [original combination].
Ancognatha manca (LeConte) [synonymy by Arrow 1937b: 6].
**syn.** Ancognatha zuniella Casey, 1915: 127 [original combination].
Ancognatha manca (LeConte) [synonymy by Arrow 1937b: 6].

#### Types.

Type of *C. manca* at MCZ (Endrődi 1966). Types of *A. aequata* and *A. laevigata* at BMNH (Endrődi 1966). Types of *A. zuniella*, *A. perspicua*, and *A. durangoana* at USNM (Endrődi 1966).

#### Distribution.

MEXICO: Chihuahua, Durango, Estado de México, Guanajuato, Jalisco, Nayarit, Michoacán, San Luis Potosí, Sinaloa, Sonora, Zacatecas. UNITED STATES: Arizona, New Mexico.

#### References.

LeConte 1866, Gerstaecker 1866, Harold 1869b, Henshaw 1885, Bates 1888, Fall and Cockerell 1907, Casey 1915, Leng 1920, Arrow 1911, 1937b, Blackwelder 1944, Saylor 1945, Blackwelder and Blackwelder 1948, Ritcher 1966, Arnett 1968, Ritcher and Baker 1974, Pike et al. 1976, Endrődi 1966, 1985a, Dimmitt and Ruibal 1980, Morón 1981, Hardy 1991, Hardy 1991, Morón and Deloya 1991, Poole and Gentili 1996, Ratcliffe and Morón 1997, Navarrete-Heredia et al. 2001, Ratcliffe 2002b, Smith 2003, 2009, Krajcik 2005, 2012, Morón and Márquez 2012, Breeschoten et al. 2013, Ratcliffe et al. 2013, Deloya et al. 2016, Ratcliffe and Cave 2017.

### Ancognatha matilei

Taxon classificationAnimaliaColeopteraScarabaeidae

Dechambre, 2000

Ancognatha matilei Dechambre, 2000: 183–184 [original combination].

#### Types.

Holotype ♂ at MNHN (Dechambre 2000).

#### Distribution.

COLOMBIA: Valle del Cauca.

#### References.

Dechambre 2000, Restrepo et al. 2003, Gasca-Álvarez and Amat-García 2010, Krajcik 2005, 2012.

### Ancognatha quadripunctata

Taxon classificationAnimaliaColeopteraScarabaeidae

Bates, 1888

Ancognatha quadripunctata Bates, 1888: 298 [original combination].

#### Types.

Type at BMNH (Endrődi 1966).

#### Distribution.

MEXICO: Chihuahua, Colima, Distrito Federal, Durango, Estado de México, Guanajuato, Guerrero, Hidalgo, Jalisco, Michoacán, Morelos, Nayarit, Oaxaca, Puebla, Sinaloa, Sonora, Veracruz.

#### References.

Bates 1888, Arrow 1937b, Blackwelder 1944, Barrera 1969, Endrődi 1966, 1985a, Ratcliffe and Morón 1997, Deloya et al. 1993, 2014a, 2016, Navarrete-Heredia et al. 2001, Ramírez-Ponce et al. 2009, Krajcik 2005, 2012, Morón and Márquez 2012, Ratcliffe et al. 2013.

#### Remarks.

*Ancognatha quadripunctata* has been reported from Ecuador (Endrődi 1966, 1985a) and Guatemala (Blackwelder 1944, Deloya et al. 2016). Major faunistic studies did not record additional specimens from Guatemala (Ratcliffe et al. 2013). No new data from Ecuador has been reported since Endrődi (1966).

### Ancognatha rugulosa

Taxon classificationAnimaliaColeopteraScarabaeidae

Endrődi, 1966

Ancognatha rugulosa Endrődi, 1966: 378–379 [original combination].

#### Types.

Holotype ♂ at ZMHB (Endrődi 1966, Ratcliffe et al. 2013).

#### Distribution.

MEXICO: Durango.

#### References.

Endrődi 1966, 1985a, Ratcliffe and Morón 1997, Krajcik 2005, 2012, Ratcliffe et al. 2013.

### Ancognatha scarabaeoides

Taxon classificationAnimaliaColeopteraScarabaeidae

Erichson, 1847

Ancognatha scarabaeoides Erichson, 1847a: 97 [original combination].
Cyclocephala scarabaeoides (Erichson) [new combination by Lacordaire 1856: 398, 399].
Ancognatha scarabaeoides Erichson [revised combination by Bates 1888: 297].
**syn.** Chalepides unduavicus Prokofiev, 2012: 3–5 [original combination].
Ancognatha scarabaeoides Erichson [synonymy by Prokofiev 2013: 131].

#### Types.

Lectotype ♂ of *A. scarabaeoides* at ZMHB (Endrődi 1966). Holotype ♂ of *C. unduavicus* at IEE (Prokofiev 2012).

#### Distribution.

BOLIVIA: Cochabamba, La Paz. COLOMBIA: Antioquia, Atlántico, Bogota, D. C., Boyacá, Caldas, Cauca, Cundinamarca, Huila, Meta, Nariño, Quindío, Risaralda, Santander, Tolima, Valle del Cauca. PANAMA: Chiriquí. PERU: Ancash, Apurimac, Cajamarca, Cusco, Huancavelica, Huánuco, Junín, La Libertad, Puno, San Martín. VENEZUELA.

#### References.

Dejean 1833, 1836b, Sturm 1843, Erichson 1847a, 1848b, Burmeister 1847, Lacordaire 1856, Marschall 1857, Harold 1869b, Bates 1888, Arrow 1937b, Blackwelder 1944, Apolinar Maria 1946, Pike et al. 1976, Endrődi 1966, 1967b, 1985a, Ruiz and Posada 1986, Posada Ochoa 1989, Ruiz and Pamalpa 1990, Montoya et al. 1994, Díaz et al. 1997, Ratcliffe 2002a, 2003, Restrepo et al. 2003, Marino et al. 2004, Lucero Malfa et al. 2006, Neita-Moreno and Gaigl 2008, Vallejo and Morón 2008, Gasca-Álvarez and Amat-García 2010, Krajcik 2005, 2012, Breeschoten et al. 2013, Prokofiev 2012, 2013, 2014, Ratcliffe et al. 2015, López-García et al. 2015, Figueroa and Ratcliffe 2016, Villalobos-Moreno et al. 2016, 2017.

#### Remarks.

Prokofiev (2014) treated A. scarabaeoides ab. unduavica as an infrasubspecific entity (color variant) after treating the name as a synonym (Prokofiev 2013).

### Ancognatha sellata

Taxon classificationAnimaliaColeopteraScarabaeidae

Arrow, 1911

Ancognatha sellata Arrow, 1911: 170 [original combination].

#### Types.

Type at BMNH (Endrődi 1966).

#### Distribution.

EL SALVADOR: Chalatenango, Santa Ana. GUATEMALA: Alta Verapaz, Baja Verapaz, Chimaltenango, Chiquimula, El Progreso, Escuintla, Guatemala, Huehuetenango, Izabal, Jalapa, Quetzaltenango, Quiché, Sacatepéquez, San Marcos, Sololá, Suchitepéquez, Zacapa. HONDURAS: Cortés, El Paraíso, Francisco Morazán, Intibucá, La Paz, Lempira, Ocotepeque, Olancho. MEXICO: Chiapas, Oaxaca. NICARAGUA: Jinotega.

#### References.

Arrow 1911, 1937b, Blackwelder 1944, Endrődi 1966, 1985a, Morón-Ríos and Morón 2001, Ramírez-Salinas et al. 2004, Méndez-Aguilar et al. 2005, Ratcliffe and Cave 2006, Krajcik 2005, 2012, Ratcliffe et al. 2013.

### Ancognatha ustulata

Taxon classificationAnimaliaColeopteraScarabaeidae

(Burmeister, 1847)

Cyclocephala ustulata Burmeister, 1847: 39 [original combination].
Ancognatha ustulata (Burmeister) [new combination by Bates 1888: 297].
**syn.** Ancognatha ustulata ustulatoides Höhne, 1922d: 373–374 [original combination].
Ancognatha ustulata var. ustulatoides Höhne [new infrasubspecific status by Arrow 1937b: 6].
Ancognatha ustulata ab. ustulatoides Höhne [revised infrasubspecific status by Endrődi 1985a: 162].

#### Types.

Lectotype ♂ of *C. ustulata* at MLUH (Endrődi 1966). Lectotype ♂ of *A. ustulata ustulatoides* at ZMHB (Endrődi 1966).

#### Distribution.

COLOMBIA: Antioquia, Bogotá D. C., Boyacá, Caldas, Cauca, Chocó, Cundinamarca, Tolima, Valle del Cauca. ECUADOR. PERU: Pasco. VENEZUELA: Mérida.

#### References.

Dejean 1833, 1836b, Sturm 1843, Burmeister 1847, Höhne 1922d, Arrow 1937b, Blackwelder 1944, Apolinar Maria 1946, Pike et al. 1976, Endrődi 1966, 1985a, Restrepo et al. 2003, Neita-Moreno and Morón 2008, Gasca-Álvarez and Amat-García 2010, Krajcik 2005, 2012, Breeschoten et al. 2013, López-García et al. 2015, Figueroa and Ratcliffe 2016.

#### Remarks.

*Ancognatha ustulata ustulatoides* was a validly described subspecies (Höhne 1922d). The subspecies was considered an infrasubspecific entity after Arrow (1937b) and was referred to as an “ab.” or “var.” (e.g., see Endrődi 1966, 1985a). The name has not been clearly synonymized with *A. ustulata* (Burmeister), but was listed as a synonym by Krajcik (2005). Endrődi (1966, 1985a) reported *A. ustulata* from Mexico and Panama, but major faunistic studies have not found additional specimens from these countries (Ratcliffe 2003, Ratcliffe et al. 2013).

### Ancognatha veliae

Taxon classificationAnimaliaColeopteraScarabaeidae

Pardo-Locarno, Gonzalez, & Montoya-Lerma, 2006

Ancognatha veliae Pardo-Locarno, Gonzalez, & Montoya-Lerma, 2006: 64–67 [original combination].

#### Types.

Holotype ♂ at MUSENUV (Pardo-Locarno et al. 2006).

#### Distribution.

COLOMBIA: Chocó.

#### References.

Pardo-Locarno et al. 2006, Gasca-Álvarez and Amat-García 2010, Krajcik 2012.

### Ancognatha vexans

Taxon classificationAnimaliaColeopteraScarabaeidae

Ratcliffe, 1992

Ancognatha vexans Ratcliffe, 1992d: 256–259 [original combination].

#### Types.

Holotype ♂ at UNSM (Ratcliffe 1992d).

#### Distribution.

COSTA RICA: Alajuela, Cartago, Guanacaste, Heredia, Puntarenas, San José. PANAMA: Chiriquí.

#### References.

Ratcliffe 1992d, 2002a, 2003, Krajcik 2005, 2012.

### Ancognatha vulgaris

Taxon classificationAnimaliaColeopteraScarabaeidae

Arrow, 1911

Ancognatha vulgaris Arrow, 1911: 169–170 [original combination].
**syn.** Ancognatha (Pseudoancognatha) nigriventris Otoya, 1945: 275–282 [original combination].
Ancognatha vulgaris Arrow [synonymy by Martínez 1965a: 64].

#### Types.

Type of *A. vulgaris* at BMNH (Endrődi 1966). Holotype ♂ of *A. nigriventris* at ICN (Otoya 1945).

#### Distribution.

BOLIVIA. BRAZIL: Amazonas. COLOMBIA: Antioquia, Boyacá, Cauca, Cundinamarca, Huila, Magdalena, Meta, Nariño, Norte de Santander, Quindío, Risaralda, Santander, Tolima. COSTA RICA: Alajuela, Cartago, Guanacaste, Heredia, Limón, Puntarenas, San José. ECUADOR. PANAMA: Bocas del Toro, Chiriquí, Coclé, Panamá, Veraguas. PERU: Ayacucho, Cajamarca, Cuzco, Huánuco, La Libertad, Lima, Loreto, Pasco, Piura, San Martín, Ucayali. VENEZUELA: Mérida.

#### References.

Arrow 1911, 1937b, Blackwelder 1944, Otoya 1945, Gutiérrez 1950, Martínez 1965a, Howden and Campbell 1974, Endrődi 1966, 1985a, Ruiz and Pamalpa 1990, Montoya et al. 1994, Restrepo et al. 2003, Ratcliffe 2002a, 2003, Onore 1997, 2005, Pardo-Locarno et al. 2005a, Útima and Vallejo 2008, Gasca-Álvarez and Amat-García 2010, Krajcik 2005, 2012, Moore 2012, Breeschoten et al. 2013, López-García et al. 2015, Ratcliffe et al. 2015, Dossey et al. 2016, Figueroa and Ratcliffe 2016, Mitasuhashi 2016, Villalobos-Moreno et al. 2016, 2017.

#### Remarks.

Some authors attributed the name *A. humeralis* to Bates (1888) and subsequently treated this taxon as a synonym of *A. vulgaris* (e.g., Arrow 1937b, Endrődi 1966, 1985a, Krajcik 2005, 2012). Bates (1888) clearly attributed the name *A. humeralis* to Burmeister (1847), and his notes on this species should not be considered a description of a new species.

The identity and species status of *A. nigriventris* is ambiguous and needs clarification. *Ancognatha nigriventris* was described from male and female specimens collected in the Colombian departments of Meta and Santander (Otoya 1945). The species was placed in a new subgenus based on its relatively well-developed maxillary teeth (Otoya 1945). Gutiérrez (1950) discussed the subgenus and Martínez (1965a) implied that the species was a synonym of *A. vulgaris*. Endrődi (1966) remarked that he had not seen the type series, or any specimens, of *A. nigriventris* and treated the species as valid. Endrődi (1985a) did not further treat *A. nigriventris*. Some subsequent papers have cited the species from Colombia (Ruiz and Pamalpa 1990, Montoya et al. 1994), while others have ignored it (e.g., Restrepo et al. 2003 and Gasca-Álvarez and Amat-García 2010). *Ancognatha nigriventris* is reported from the Colombian states of Meta, Nariño, and Santander (Otoya 1945, Ruiz and Pamalpa 1990, Montoya et al. 1994).

### Genus Arriguttia

Taxon classificationAnimaliaColeopteraScarabaeidae

Martínez, 1960

Arriguttia Martínez, 1960a: 97–98 [original usage].

#### Type species.

*Cyclocephala brevissima* Arrow, 1911, by monotypy.

#### Keys.

Endrődi 1966, 1985a, Ratcliffe 1985, Jameson et al. 2002.

#### Valid taxa.

2 species.

### Arriguttia bolivari

Taxon classificationAnimaliaColeopteraScarabaeidae

Martínez, 1968

Arriguttia bolivari Martínez, 1968a: 185–188 [original combination].

#### Types.

Holotype ♂ at MACN (Antonio Martínez Collection) (Martínez 1968a).

#### Distribution.

BRAZIL: Amazonas.

#### References.

Martínez 1968a, Krajcik 2005, 2012.

### Arriguttia brevissima

Taxon classificationAnimaliaColeopteraScarabaeidae

(Arrow, 1911)

Cyclocephala brevissima Arrow, 1911: 175–176 [original combination].
Arriguttia brevissima (Arrow) [new combination by Martínez 1960a: 98].

#### Types.

Type at BMNH (Endrődi 1966).

#### Distribution.

BRAZIL: Mato Grosso, Pará. GUYANA. FRENCH GUIANA: Cayenne.

#### References.

Arrow 1911, 1937b, Blackwelder 1944, Martínez 1960a, 1968a, Pike et al. 1976, Endrődi 1966, 1985a, Krajcik 2005, 2012, Ponchel 2006, 2011, 2015, Costa et al. 2017.

### Genus Aspidolea

Taxon classificationAnimaliaColeopteraScarabaeidae

Bates, 1888

Aspidolea Bates, 1888: 296 [original usage].
**syn.** Paraspidolea Höhne, 1922a: 90–91 [original usage]. Type species: Paraspidolea suturalis Höhne, 1922, by original designation.
Aspidolea Bates [synonymy by Endrődi 1966: 338].

#### Type species.

*Aspidolea singularis* Bates, 1888, by monotypy.

#### Keys.

Arnett 1968 (USA), Endrődi 1966, 1985a, Morón 1979 (Veracruz, Mexico), Morón et al. 1985 (Chiapas, Mexico), Ratcliffe 1985, Dechambre 1992, Maes 1994 (Nicaragua), Jameson et al. 2002, Ratcliffe 2003 (Costa Rica and Panama), Ratcliffe and Cave 2006 (Honduras, Nicaragua, and El Salvador), Neita-Moreno et al. 2007 (larvae), Gasca-Álvarez and Amat-García 2010 (Colombia), Pardo-Locarno 2013 (Valle del Cauca, Colombia), Ratcliffe et al. 2013 (Guatemala, Belize, and Mexico).

#### Valid taxa.

24 species.

### Aspidolea bleuzeni

Taxon classificationAnimaliaColeopteraScarabaeidae

Dechambre, 1992

Aspidolea bleuzeni Dechambre, 1992: 73, 74–75 [original combination].

#### Types.

Holotype ♂ at MNHN (Dechambre 1992).

#### Distribution.

FRENCH GUIANA: Kourou, Roura, St.-Laurent du Maroni.

#### References.

Dechambre 1992, Touroult et al. 2010, Ponchel 2011, Krajcik 2005, 2012.

### Aspidolea boulardi

Taxon classificationAnimaliaColeopteraScarabaeidae

Dechambre, 1992

Aspidolea boulardi Dechambre, 1992: 73, 74 [original combination].

#### Types.

Holotype ♂ at MNHN (Dechambre 1992).

#### Distribution.

BRAZIL: Pará.

#### References.

Dechambre 1992, Krajcik 2005, 2012, Breeschoten et al. 2013.

### Aspidolea brunnea

Taxon classificationAnimaliaColeopteraScarabaeidae

Höhne, 1922

Aspidolea brunnea Höhne, 1922a: 90 [original combination].

#### Types.

Holotype ♂ at ZMHB (Endrődi 1966).

#### Distribution.

BOLIVIA: Cochabamba, La Paz, Santa Cruz. COLOMBIA: Cundinamarca, Meta. PERU: Ayacucho, Cusco, Madre de Dios, Puno.

#### References.

Höhne 1922a, Arrow 1937b, Blackwelder 1944, Endrődi 1966, 1985a, Restrepo-Giraldo et al. 2003, Krajcik 2005, 2012, López-García et al. 2015, Ratcliffe et al. 2015).

### Aspidolea chalumeaui

Taxon classificationAnimaliaColeopteraScarabaeidae

Endrődi, 1977

Aspidolea chalumeaui Endrődi, 1977a: 5–6 [original combination].

#### Types.

Holotype ♂ at HNHM (Dechambre 1992).

#### Distribution.

BRAZIL: Mato Grosso.

#### References.

Endrődi 1977a, 1985a, Dechambre 1992, Krajcik 2005, 2012.

### Aspidolea cognata

Taxon classificationAnimaliaColeopteraScarabaeidae

Höhne, 1922

Aspidolea cognata Höhne, 1922a: 83–84 [original combination].

#### Types.

Holotype ♂ at ZMHB (Endrődi 1966).

#### Distribution.

COLOMBIA: Boyacá, Cauca, Cundinamarca, Risaralda. ECUADOR: Morona-Santiago. PERU. VENEZUELA: Aragua, Capital District (Caracas).

#### References.

Höhne 1922a, Arrow 1937b, Blackwelder 1944, Roze 1955, Martínez 1975, Endrődi 1966, 1985a, Restrepo-Giraldo et al. 2003, Krajcik 2005, 2012, Breeschoten et al. 2013, López-García et al. 2015.

#### Remarks.

Ratcliffe et al. (2013) do not record *A. cognata* from Mexico, Guatemala, or Belize. The data from Mexico previously reported for *A. cognata* may be erroneous (Höhne 1922a, Arrow 1937b, Blackwelder 1944, Endrődi 1966, 1985a, Ratcliffe et al. 2013).

### Aspidolea collaris

Taxon classificationAnimaliaColeopteraScarabaeidae

Endrődi, 1966

Aspidolea collaris Endrődi, 1966: 342, 346–347 [original combination].

#### Types.

Holotype ♂ at NHMB (Frey Collection) (Endrődi 1966).

#### Distribution.

PERU: Madre de Dios.

#### References.

Endrődi 1966, 1985a, Krajcik 2005, 2012, Ratcliffe et al. 2015.

### Aspidolea clypeata

Taxon classificationAnimaliaColeopteraScarabaeidae

(Burmeister, 1847)

Cyclocephala clypeata Burmeister, 1847: 42 [original combination].
Aspidolea clypeata (Burmeister) [new combination by Höhne 1922a: 81].

#### Types.

Lectotype ♂ at MLUH (Endrődi 1966).

#### Distribution.

BOLIVIA: Beni. COLOMBIA. FRENCH GUIANA: Cayenne, Mana, St.-Laurent du Maroni. GUYANA: Upper Demerara-Berbice.

#### References.

Burmeister 1847, Harold 1869b, Arrow 1937b, Blackwelder 1944, Gruner 1971, Pike et al. 1976, Endrődi 1966, Endrődi 1973a, 1985a, Restrepo-Giraldo et al. 2003, Ponchel 2011, Krajcik 2005, 2012.

#### Remarks.

*Aspidolea clypeata* possibly occurs in Mato Grosso, Brazil based on reported locality data for the unavailable name A. clypeata ab. brasiliana (Endrődi 1966).

### Aspidolea ecuadoriana

Taxon classificationAnimaliaColeopteraScarabaeidae

Endrődi, 1985

Aspidolea ecuadoriana Endrődi, 1985b: 74 [original combination].

#### Types.

Holotype ♂ at JPVC (Colette Voirin) (Endrődi 1985b).

#### Distribution.

ECUADOR: Pichincha.

#### References.

Endrődi 1985b, Krajcik 2005, 2012.

### Aspidolea epipleuralis

Taxon classificationAnimaliaColeopteraScarabaeidae

Höhne, 1922

Aspidolea epipleuralis Höhne, 1922a: 84–85 [original combination].

#### Types.

Holotype ♂ at ZMHB (Endrődi 1966).

#### Distribution.

ECUADOR: Morona-Santiago.

#### References.

Höhne 1922a, Arrow 1937b, Blackwelder 1944, Endrődi 1966, 1985a, Krajcik 2005, 2012.

### Aspidolea fuliginea

Taxon classificationAnimaliaColeopteraScarabaeidae

(Burmeister, 1847)

Cyclocephala fuliginea Burmeister, 1847: 42 [original combination].
Paraspidolea fuliginea (Burmeister) [new combination by Höhne 1922a: 81, 91].
Aspidolea fuliginea (Burmeister) [new combination by Endrődi 1966: 348–350.

#### Types.

Lectotype ♂ at MLUH (Endrődi 1966).

#### Distribution.

ARGENTINA. BELIZE: Cayo, Stann Creek, Toledo. BRAZIL. COLOMBIA: Antioquia, Bolívar, Boyacá, Caldas, Cauca, Chocó, Cundinamarca, Meta, Risaralda, Valle del Cauca. COSTA RICA: Alajuela, Cartago, Heredia, Limón, Puntarenas, San José. ECUADOR: Guayas. EL SALVADOR: Ahuachapán, Cuscatlán, La Libertad. GUATEMALA: Alto Verapaz, Baja Verapaz, Chimaltenango, Escuintla, Guatemala, Huehuetenango, Izabal, Jutiapa, Petén, Sacatepéquez, San Marcos, Suchitepéquez, Zacapa. HONDURAS: Atlántida, Choluteca, Comayagua, Cortés, Francisco Morazán, Gracias a Dios, Lempira, Olancho, Yoro. MEXICO: Chiapas, Guerrero, Hidalgo, Jalisco, Morelos, Oaxaca, Puebla, Tabasco, Veracruz. NICARAGUA: Masaya, Río San Juan. PANAMA: Bocas del Toro, Panama Canal Zone, Chiriquí, Colón, Darien, Panamá. PERU: Cusco, Madre de Dios, Puno. TRINIDAD AND TOBAGO: Trinidad (Couva-Tabaquite-Talparo). VENEZUELA: Capital District (Caracas), Mérida.

#### References.

Bates 1888, Arrow 1937b, Martorell and Salas 1939, Blackwelder 1944, Roze 1955, Pike et al. 1976, Endrődi 1966, 1985a, Morón 1979, Thomas 1993, Lobo and Morón 1993, Morón 1994, Ratcliffe and Morón 1997, Navarrete-Heredia et al. 2001, Carrillo-Ruiz and Morón 2003, Ratcliffe 2002a, 2003, Restrepo-Giraldo et al. 2003, Espino 2005, Pardo-Locarno et al. 2003, 2005a, Neita-Moreno et al. 2006, Ratcliffe and Cave 2006, Pacheco-F. et al. 2008, Núñez-Avellaneda and Rojas-Robles 2008, Útima and Vallejo 2008, García-López et al. 2011, Neita-Moreno 2011, Krajcik 2005, 2012, Moore 2012, Breeschoten et al. 2013, Pardo-Locarno 2013, Deloya et al. 1993, 2014, López-García et al. 2015, Ratcliffe et al. 2013, 2015.

### Aspidolea gaudairethorei

Taxon classificationAnimaliaColeopteraScarabaeidae

Endrődi, 1980

Aspidolea gaudairethorei Endrődi, 1980: 39–40 [original combination].

#### Types.

Holotype ♀ in André Gaudaíre-Thore Collection (Sens, France) (Endrődi 1980).

#### Distribution.

FRENCH GUIANA: Cayenne, Roura.

#### References.

Endrődi 1980, 1985a, Touroult et al. 2010, Ponchel 2011, Krajcik 2005, 2012.

### Aspidolea helleri

Taxon classificationAnimaliaColeopteraScarabaeidae

(Höhne, 1922)

Paraspidolea helleri Höhne, 1922b: 371 [original combination].
Aspidolea helleri (Höhne) [new combination by Endrődi 1966: 341, 351].

#### Types.

Lectotype ♂ at MTD (Dechambre 1992).

#### Distribution.

BRAZIL: Pará. FRENCH GUIANA: Cayenne. SURINAME.

#### References.

Höhne 1922b, Arrow 1937b, Blackwelder 1944, Endrődi 1966, 1985a, Dechambre 1979a, 1992, Krajcik 2005, 2012.

### Aspidolea kuntzeni

Taxon classificationAnimaliaColeopteraScarabaeidae

Höhne, 1922

Aspidolea kuntzeni Höhne, 1922a: 87–89 [original combination].
**syn.** Aspidolea pygidialis Höhne, 1922a: 89–90 [original combination].
Aspidolea kuntzeni ab. pygidialis Höhne [new status by Endrődi 1966: 351].
Aspidolea kuntzeni Höhne [synonymy by Ratcliffe 2002a: 32].

#### Types.

Holotype of *A. kuntzeni* at ZMHB (Endrődi 1966). Holotype of *A. pygidialis* at ZMHB (Endrődi 1966).

#### Distribution.

COLOMBIA: Chocó, Valle del Cauca. COSTA RICA: Alajuela, Cartago, Guanacaste, Heredia, Limón, Puntarenas, San José. PANAMA: Bocas del Toro, Panama Canal Zone, Darien, Panamá. SURINAME. VENEZUELA: Aragua, Capital District (Caracas), Carabobo, Mérida.

#### References.

Höhne 1922a, Arrow 1937b, Blackwelder 1944, Roze 1955, Endrődi 1966, 1985a, Poole and Gentili 1996, Ratcliffe 2002a, 2003, Restrepo-Giraldo et al. 2003, Neita-Moreno 2011, Krajcik 2005, 2012.

#### Remarks.

Endrődi (1966, 1985a) erroneously reported *A. kuntzeni* from the United States (New Mexico) (Ratcliffe 2003).

### Aspidolea laticeps

Taxon classificationAnimaliaColeopteraScarabaeidae

(Harold, 1869)

Cyclocephala laticeps Harold, 1869a: 124 [original combination].
Aspidolea laticeps (Harold) [new combination by Höhne 1922a: 81].
**syn.** Cyclocephala clypeata Erichson, 1847a: 97 [original combination].
Cyclocephala laticeps Harold [new replacement name by Harold 1869a: 124, homonym of Cyclocephala clypeata Burmeister, 1847: 42–43].

#### Types.

Lectotype ♂ at ZMHB (Endrődi 1966).

#### Distribution.

PERU: Pasco. VENEZUELA: Carabobo.

#### References.

Erichson 1847a, Burmeister 1847, Harold 1869a, Arrow 1937b, Pike et al. 1976, Endrődi 1966, 1985a, Krajcik 2005, 2012, Ratcliffe et al. 2015.

### Aspidolea lindae

Taxon classificationAnimaliaColeopteraScarabaeidae

Ratcliffe, 1977

Aspidolea lindae Ratcliffe, 1977: 429–430 [original combination].

#### Types.

Holotype ♂ at UNSM (Ratcliffe 1977).

#### Distribution.

COLOMBIA: Amazonas. PERU.

#### References.

Ratcliffe 1977, Dupuis 1999, Restrepo-Giraldo et al. 2003, Krajcik 2005, 2012, Otavo et al. 2013, Ratcliffe et al. 2015.

### Aspidolea mimethes

Taxon classificationAnimaliaColeopteraScarabaeidae

(Höhne, 1922)

Paraspidolea mimethes Höhne, 1922a: 93–94 [original combination].
Aspidolea mimethes (Höhne) [new combination by Endrődi 1966: 342, 353–354].

#### Types.

Male type was not found. A female type is at MTD (Endrődi 1966).

#### Distribution.

PERU: Pasco.

#### References.

Höhne 1922a, Arrow 1937b, Blackwelder 1944, Endrődi 1966, 1985a, Krajcik 2005, 2012, Ratcliffe et al. 2015.

### Aspidolea notaticollis

Taxon classificationAnimaliaColeopteraScarabaeidae

Höhne, 1922

Aspidolea notaticollis Höhne, 1922a: 86 [original combination].
**syn.** Aspidolea bigutticollis Höhne, 1922a: 87 [original combination].
Aspidolea notaticollis Höhne [synonymy by Endrődi 1966: 339, 354].
**syn.** Aspidolea tibialis Höhne, 1922a: 85–86 [original combination].
Aspidolea notaticollis ab. tibialis (Höhne) [new infrasubspecific status by Endrődi 1980: 41].

#### Types.

Holotype ♂ of *A. notaticollis*, type of *A. bigutticollis*, and holotype ♂ of *A. tibialis* at ZMHB (Endrődi 1966).

#### Distribution.

BOLIVIA: Cochabamba. COLOMBIA: Antioquia, Chocó, Cundinamarca, Meta, Tolima. COSTA RICA: Alajuela, Cartago, Guanacaste, Heredia, Puntarenas. ECUADOR: Morona-Santiago, Orellana, Pastaza. Panama: Bocas del Toro, Colón, Darien, Panama Canal Zone. PERU: Ayacucho, Huánuco, Junín, Madre de Dios, Cusco, Pasco.

#### References.

Höhne 1922a, Arrow 1937b, Blackwelder 1944, Endrődi 1966, 1980, 1985a, Ratcliffe 2002a, 2003, Restrepo-Giraldo et al. 2003, García-López et al. 2010, Neita-Moreno 2011, Krajcik 2005, 2012, Moore 2012, López-García et al. 2015, Ratcliffe et al. 2015.

### Aspidolea pelioptera

Taxon classificationAnimaliaColeopteraScarabaeidae

(Burmeister, 1847)

Cyclocephala pelioptera Burmeister, 1847: 42 [original combination].
Paraspidolea pelioptera (Burmeister) [new combination by Höhne 1922a: 81, 91].
Aspidolea pelioptera (Burmeister) [new combination by Endrődi 1966: 338, 343, 355–356].

#### Types.

Lectotype ♂ at MLUH (Endrődi 1966).

#### Distribution.

ARGENTINA: Buenos Aires. BRAZIL: Espírito Santo, Paraná, Rio de Janeiro, Rio Grande so Sul, Santa Catarina, São Paulo.

#### References.

Burmeister 1847, Harold 1869b, Höhne 1922a, Arrow 1937b, Blackwelder 1944, Guimarães 1944, Pike et al. 1976, Endrődi 1966, 1985a, Grossi et al. 2011, Krajcik 2005, 2012, Fuhrmann 2013.

### Aspidolea pokornyi

Taxon classificationAnimaliaColeopteraScarabaeidae

Dupuis, 2014

Aspidolea pokornyi Dupuis, 2014: 54–56 [original combination].

#### Types.

Holotype ♂ in Pokorny Collection (Prague, Czech Republic) (Dupuis 2014).

#### Distribution.

ECUADOR: Napo.

#### References.

Dupuis 2014.

### Aspidolea quadrata

Taxon classificationAnimaliaColeopteraScarabaeidae

Endrődi, 1980

Aspidolea quadrata Endrődi, 1980: 40–41 [original combination].

#### Types.

Holotype ♂ in André Gaudaíre-Thore Collection (Sens, France) (Endrődi 1980).

#### Distribution.

FRENCH GUIANA: Kourou, Roura, Sinnamary.

#### References.

Endrődi 1980, 1985a, Gibernau et al. 2003, Touroult et al. 2010, Krajcik 2005, 2012, Dupuis 2014, Ponchel 2006, 2015.

### Aspidolea singularis

Taxon classificationAnimaliaColeopteraScarabaeidae

Bates, 1888

Aspidolea singularis Bates, 1888: 296–297 [original combination].
**syn.** Aspidolea cevallosi Martínez, 1975a: 307–313 [original combination].
Aspidolea singularis Bates [synonymy by Endrődi 1985a: 155].
**syn.** Aspidolea similis Höhne, 1922a: 82–83 [original combination].
Aspidolea singularis ab. similis (Höhne) [new infrasubspecific status by Endrődi 1966: 356].
Aspidolea singularis Bates [synonymy by Ratcliffe 2002a: 27].
**syn.** Aspidolea texana Höhne, 1922a: 84 [original combination].
Aspidolea singularis Bates [synonymy by Endrődi 1966: 356].

#### Types.

Type of *A. singularis* at BMNH (Endrődi 1966). Holotypes of *A. texana* and *A. similis* both at ZMHB (Endrődi 1966). Holotype ♂ of *A. cevallosi* at MACN (Antonio Martínez Collection) (Martínez 1975).

#### Distribution.

BELIZE: Cayo, Stann Creek. BRAZIL. COLOMBIA: Amazonas, Antioquia, Boyacá, Cauca, Chocó, Cundinamarca, Meta, Risaralda, Santander, Tolima, Valle del Cauca. COSTA RICA: Alajuela, Cartago, Guanacaste, Heredia, Limón, Puntarenas, San José. ECUADOR: Bolívar, Guayas, Loja, Los Ríos. EL SALVADOR: Ahuachapán, La Libertad, Morazán, San Salvador, Santa Ana. GUATEMALA: Alta Veracruz, Baja Veracruz, Chiquimula, El Progresso, Escuintla, Huehuetenango, Izabal, Quetzaltenango, Quiché, Retalhuleu, San Marcos, Santa Rosa, Suchitepéquez, Zacapa. HONDURAS: Atlántida, Choluteca, Copán, Cortés, El Paraíso, Francisco Morazán, Gracias a Dios, Lempira, Olancho, Santa Bárbara, Yoro. MEXICO: Chiapas, Oaxaca, Puebla, Tabasco, Veracruz. NICARAGUA: Chontales, Jinotega, Matagalpa, Nueva Segovia, RAA Norte, Río San Juan. Panama: Bocas del Toro, Chiriquí, Coclé, Colón, Darien, Herrera, Panamá, Panama Canal Zone, San Blas, Veraguas. PERU.

#### References.

Bates 1888, Höhne 1922a, Arrow 1937b, Blackwelder 1944, Martínez 1975, Endrődi 1966, 1985a, Morón et al. 1985, Palacios-Rios et al. 1990, Thomas 1993, Maes 1987, 1994, Poole and Gentili 1996, Ratcliffe and Morón 1997, Dupuis 1999, Elizondo-Solís 2002, Ratcliffe 2002a, 2003, Restrepo-Giraldo et al. 2003, Pardo-Locarno et al. 2003, 2005a, Huerta-Espino 2005, Neita-Moreno et al. 2006, 2007, Ratcliffe and Cave 2006, Pacheco-F. et al. 2008, Útima and Vallejo 2008, Neita-Moreno 2011, Krajcik 2005, 2012, García-López et al. 2010, 2011, 2013, Pardo-Locarno 2013, Otavo et al. 2013, Yepes-Rodriguez et al. 2013, López-García et al. 2015, Ratcliffe et al. 2013, 2015.

#### Remarks.

*Aspidolea singularis* was reported from San Antonio, Texas (United States) (Höhne 1922a, Blackwelder and Blackwelder 1948, Endrődi 1966, 1985a). Saylor (1945) suggested that these data were likely erroneous. More complete data indicate that the northern range limit of *A. singularis* is near the state of Puebla, Mexico (Ratcliffe et al. 2013).

### Aspidolea suturalis

Taxon classificationAnimaliaColeopteraScarabaeidae

(Höhne, 1922)

Paraspidolea suturalis Höhne, 1922a: 91–92 [original combination].
Aspidolea suturalis (Höhne) [new combination by Endrődi 1966: 338, 341, 357–358].
**syn.** Paraspidolea ohausi Höhne, 1922a: 94–95 [original combination].
Aspidolea suturalis (Höhne) [synonymy by Endrődi 1966: 357].

#### Types.

Holotypes of *P. suturalis* and *P. ohausi* at ZMHB (Endrődi 1966).

#### Distribution.

BOLIVIA: Cochabamba, La Paz, Yungas. COLOMBIA: Antioquia. ECUADOR: Bolívar, Loja. PERU: Ayacucho. VENEZUELA: Mérida.

#### References.

Höhne 1922a, Arrow 1937b, Blackwelder 1944, Endrődi 1966, 1985a, Restrepo-Giraldo et al. 2003, Krajcik 2005, 2012, Ratcliffe et al. 2015.

#### Remarks.

Endrődi (1966, 1985a) reported *A. suturalis* from Mexico, but this is likely an erroneous record (Ratcliffe et al. 2013).

### Aspidolea suturella

Taxon classificationAnimaliaColeopteraScarabaeidae

(Höhne, 1922)

Paraspidolea suturella Höhne, 1922a: 95 [original combination].
Aspidolea suturella (Höhne) [new combination by Endrődi 1966: 338, 342, 358–359].

#### Types.

Holotype ♂ at ZMHB (Endrődi 1966).

#### Distribution.

COLOMBIA: Antioquia, Cauca, Cundinamarca, Valle de Cauca.

#### References.

Höhne 1922a, Arrow 1937b, Blackwelder 1944, Endrődi 1966, 1985a, Restrepo-Giraldo et al. 2003, Krajcik 2005, 2012.

### Aspidolea testacea

Taxon classificationAnimaliaColeopteraScarabaeidae

(Höhne, 1922)

Paraspidolea testacea Höhne, 1922a: 92–93 [original combination].
Aspidolea testacea (Höhne) [new combination by Endrődi 1966: 338, 342, 359].

#### Types.

Holotype ♂ at ZMHB (Endrődi 1966).

#### Distribution.

BOLIVIA. COLOMBIA.

#### References.

Höhne 1922a, Arrow 1937b, Blackwelder 1944, Endrődi 1966, 1985a, Restrepo-Giraldo et al. 2003, Krajcik 2005, 2012.

### Aspidolea theresae

Taxon classificationAnimaliaColeopteraScarabaeidae

Dupuis, 1999

Aspidolea theresae Dupuis, 1999: 186–187 [original combination].

#### Types.

Holotype ♂ at MNHN (Dupuis 1999).

#### Distribution.

ECUADOR: Napo.

#### References.

Dupuis 1999, Krajcik 2005, 2012.

### Aspidolea vicina

Taxon classificationAnimaliaColeopteraScarabaeidae

Dechambre, 1992

Aspidolea vicina Dechambre, 1992: 73, 74 [original combination].

#### Types.

Holotype ♂ at MNHN (Dechambre 1992).

#### Distribution.

BRAZIL: Pará.

#### References.

Dechambre 1992, Krajcik 2005, 2012.

### Genus Augoderia

Taxon classificationAnimaliaColeopteraScarabaeidae

Burmeister, 1847

Augoderia Burmeister, 1847: 33–34 [original usage].

#### Type species.

*Augoderia nitidula* Burmeister, 1847, by monotypy.

#### Keys.

Endrődi 1966, 1985a, Ratcliffe 1985, Jameson et al. 2002.

#### Valid taxa.

5 species and subspecies.

### Augoderia boliviana

Taxon classificationAnimaliaColeopteraScarabaeidae

Endrődi, 1981

Augoderia boliviana Endrődi, 1981: 198 [original combination].

#### Types.

Holotype ♂ at ZSMC (Endrődi 1981).

#### Distribution.

BOLIVIA: Santa Cruz.

#### References.

Endrődi 1981, 1985a, Ponchel 2009, Krajcik 2005, 2012.

### Augoderia freyi

Taxon classificationAnimaliaColeopteraScarabaeidae

Endrődi, 1967

Augoderia freyi Endrődi, 1967a: 407–409 [original combination].

#### Types.

Holotype ♂ at NHMB (Frey Collection) (Endrődi 1967a).

#### Distribution.

BOLIVIA. PERU: Madre de Dios.

#### References.

Endrődi 1967a, 1981, 1985a, Ponchel 2009, Krajcik 2005, 2012, Ratcliffe et al. 2015.

### Augoderia giuglarisi

Taxon classificationAnimaliaColeopteraScarabaeidae

Ponchel, 2009

Augoderia giuglarisi Ponchel, 2009: 183–184 [original combination].

#### Types.

Holotype ♂ in the Yannig Ponchel Collection (Ponchel 2009).

#### Distribution.

FRENCH GUIANA.

#### References.

Ponchel 2009, Krajcik 2012.

### Augoderia nitidula nitidula

Taxon classificationAnimaliaColeopteraScarabaeidae

Burmeister, 1847

Augoderia nitidula Burmeister, 1847: 34 [original combination].

#### Types.

Lectotype at MLUH (Endrődi 1966).

#### Distribution.

ARGENTINA: Buenos Aires, Misiones. BOLIVIA: Cochabamba, Santa Cruz. BRAZIL: Minas Gerais, São Paulo, Paraná. VENEZUELA: Caracas.

#### References.

Burmeister 1847, Harold 1869b, Ohaus 1909, Arrow 1937b, Anonymous 1900, 1940, Blackwelder 1944, Guimarães 1944, Lima 1953, Martínez 1966, Gibbs et al. 1977, Endrődi 1966, 1981, 1985a, Riehs 2005, Ronqui and Lopes 2006, Ponchel 2009, Grossi et al. 2011, Krajcik 2005, 2012, Breeschoten et al. 2013.

### Augoderia nitidula yungana

Taxon classificationAnimaliaColeopteraScarabaeidae

Martínez, 1966

Augoderia nitidula yungana Martínez, 1966: 73–75 [original combination].

#### Types.

Holotype ♂ at MACN (Antonio Martínez Collection) (Martínez 1966).

#### Distribution.

BOLIVIA: Cochabamba.

#### References.

Martínez 1966, Endrődi 1985a, Ponchel 2009, Krajcik 2005, 2012.

### Genus Chalepides

Taxon classificationAnimaliaColeopteraScarabaeidae

Casey, 1915

Parachalepus (Chalepides) Casey, 1915: 176–177 [original usage]. Proposed as a subgenus.
Chalepides Casey [new genus status by Prell 1936: 151; see also Arrow 1937a: 36].
**syn.** Parachalepus (Parachalepus) Casey, 1915: 175–176 [original usage]. Parachalepus Casey is a junior homonym of Parachalepus Baly, 1885.

#### Type species.

Parachalepus (Chalepides) eucephalus Casey, 1915, by original designation.

#### Keys.

Casey 1915, Endrődi 1966, 1985a, Jameson et al. 2002, Joly and Escalona 2002a, Gasca-Álvarez and Amat-García 2010 (Colombia).

#### Valid taxa.

15 species.

### Chalepides alliaceus

Taxon classificationAnimaliaColeopteraScarabaeidae

(Burmeister, 1847)

Chalepus alliaceus Burmeister, 1847: 77 [original combination].
Dyscinetus alliaceus (Burmeister) [new combination by Harold 1869a: 123].
Parachalepus (Chalepides) alliaceus (Burmeister) [new combination and new subgeneric classification by Casey 1915: 176].
Chalepides alliaceus (Burmeister) [new combination by Prell 1936: 151].

#### Types.

Lectotype ♂ at MLUH (Endrődi 1966, Joly and Escalona 2002a).

#### Distribution.

BOLIVIA: Beni, Santa Cruz. BRAZIL: Mato Grosso, Mato Grosso do Sul, Paraná, Rio de Janeiro.

#### References.

Burmeister 1847, Harold 1869a, Ohaus 1909, Casey 1915, Prell 1936, Arrow 1937b, Anonymous 1940, Blackwelder 1944, Endrődi 1966, 1973a, 1985a, Joly and Escalona 2002a, Riehs 2005, Krajcik 2005, 2012.

### Chalepides anomalus

Taxon classificationAnimaliaColeopteraScarabaeidae

Martínez, 1978

Chalepides anomalus Martínez, 1978b: 17–19 [original combination].

#### Types.

Holotype ♂ at MACN (Antonio Martínez Collection) (Martínez 1978b, Joly and Escalona 2002a).

#### Distribution.

ARGENTINA: Corrientes, Entre Ríos. URUGUAY: Artigas.

#### References.

Martínez 1978b, Endrődi 1985a, Joly and Escalona 2002a, Krajcik 2005, 2012.

### Chalepides barbatus

Taxon classificationAnimaliaColeopteraScarabaeidae

(Fabricius, 1787)

Scarabaeus barbatus Fabricius, 1787: 10 [original combination].
Melolontha barbata (Fabricius) [new combination by Fabricius 1801: 167].
Chalepus barbatus (Fabricius) [new combination by Burmeister 1847: 77].
Dyscinetus barbatus (Fabricius) [new combination by Harold 1869a: 123].
Parachalepus (Parachalepus) barbatus (Fabricius) [new combination and new subgeneric classification by Casey 1915: 175].
Chalepides barbatus (Fabricius) [new combination by Prell 1936: 151].
**syn.** Chalepides hydrophiloides argentinus Prell, 1937c: 9 [original combination].
Chalepides barbatus argentinus Prell [new subspecific status by Endrődi 1966: 403].
Chalepides barbatus (Fabricius) [synonymy by Ratcliffe and Cave 2015: 71].

#### Types.

Lectotype ♀ of *S. barbatus* deposited at ZMUK, now housed at ZMUC (Endrődi 1966). Lectotype ♀ of *C. hydrophiloides argentinus* at ZMHB (Endrődi 1966).

#### Distribution.

ANTIGUA: St. Philip. ARGENTINA: Buenos Aires, Corrientes, Entre Ríos, Santa Fe. BRAZIL: Mato Grosso, Rio Grande do Sul, Santa Catarina, São Paulo. BRITISH VIRGIN ISLANDS: Anegada. DOMINICAN REPUBLIC: Distrito Nacional, Duarte, Espaillat, Hato Mayor, La Altagracia, La Vega, María Trinidad Sánchez, Monseñor Nouel, Puerto Plata, Samana, San Cristóbal, San Juan. MARTINIQUE: Fort-de-France. PARAGUAY: Alto Paraná, Caaguazú, Itapúa, Misiones, San Pedro. PUERTO RICO: Aguada, Aguadilla, Añasco, Arecibo, Barceloneta, Barranquitas, Bayamón, Cabo Rojo, Caguas, Camuy, Carolina, Cataño, Cayey, Ciales, Fajardo, Florida, Guánica, Guaynabo, Isabela, Jayuya, Lajas, Lares, Las Piedras, Loiza, Luquillo, Manatí, Maricao, Mayagüez, Morovis, Nagüabo, Patillas, Ponce, Quebradillas, Rincón, Río Grande, San Germán, San Juan, Santa Isabel, Toa Baja, Utuado, Vega Alta, Vega Baja, Villalba. SAINT BARTHÉLEMY: Lorient. SAINT KITTS AND NEVIS: St. Kitts, Nevis. SAINT LUCIA: Micoud, Vieux Fort. SAINT MARTIN. SAINT VINCENT: St. Andrew, St. David. URUGUAY: Artigas, Colonia. UNITED STATES VIRGIN ISLANDS: St. Croix, St. Thomas.

#### References.

Fabricius 1787, 1801, Dejean 1821, 1833, 1836b, Moritz 1836, Sturm 1843, Burmeister 1847, Reiche 1859, Harold 1869a, Stahl 1882, Casey 1915, Smyth 1916, Leng and Mutchler 1917, Stevenson 1918, Box 1925, Wolcott 1936, 1937, Prell 1937c, Paulian 1947, Van Dinther 1960, Zimsen 1964, Barrera 1969, Miskimen and Bond 1970, Chalumeau 1982, Endrődi 1966, 1985a, Audureau 2001, Joly and Escalona 2002a, Soave et al. 2006, Krajcik 2005, 2012, Breeschoten et al. 2013, Ratcliffe and Cave 2015, Peck 2009, 2016.

#### Remarks.

*C. barbatus* was reported from Guatemala (Bates 1888) and Cuba (Endrődi 1966, 1985a), but major faunistic studies have not found additional specimens from these countries (Ratcliffe et al. 2013, Ratcliffe and Cave 2015).

### Chalepides carinatus

Taxon classificationAnimaliaColeopteraScarabaeidae

Joly & Escalona, 2002

Chalepides carinatus Joly & Escalona, 2002a: 42, 44, 65–67 [original combination].

#### Types.

Holotype ♂ at USNM (Joly and Escalona 2002a).

#### Distribution.

ARGENTINA: Buenos Aires. BRAZIL: Rio Grande do Sul. URUGUAY: Montevideo.

#### References.

Joly and Escalona 2002a, Krajcik 2005, 2012.

### Chalepides comes

Taxon classificationAnimaliaColeopteraScarabaeidae

Prell, 1937

Chalepides comes Prell, 1937a: 187 [original combination].
**syn.** Chalepides punctulatus Arrow, 1937a [original combination].
Chalepides comes Prell [synonymy by Endrődi 1966: 405].
**syn.** Chalepides semipunctatus Prell, 1937c: 8 [original combination].
Chalepides comes Prell [synonymy by Endrődi 1966: 405].

#### Types.

Lectotype ♀ of *C. comes* and lectotype ♂ of *C. semipunctatus* both at ZMHB (Endrődi 1966). Type of *C. punctulatus* at BMNH (Endrődi 1966).

#### Distribution.

BOLIVIA: Beni, Santa Cruz. BRAZIL: Amapá, Bahia, Distrito Federal, Mato Grosso, Minas Gerais, Pará, Pernambuco, Rio Grande do Sul, São Paulo. COLOMBIA: Antioquia, Bolívar. FRENCH GUIANA. PARAGUAY: Amambay, San Pedro. VENEZUELA: Amazonas, Apure, Bolívar, Guárico, Monagas, Táchira.

#### References.

Arrow 1937a, b, Prell 1937a, c, Blackwelder 1944, Roze 1955, Martínez 1978b, Endrődi 1966, 1985a, Joly and Escalona 2002a, Restrepo-Giraldo et al. 2003, Gasca-Álvarez and Amat-García 2010, Ponchel 2011, Krajcik 2005, 2012.

### Chalepides dilatatus

Taxon classificationAnimaliaColeopteraScarabaeidae

(Mannerheim, 1829)

Apogonia dilatata Mannerheim, 1829: 55–56 [original combination].
Chalepus dilatatus (Mannerheim) [new combination by Burmeister 1847: 77].
Dyscinetus dilatatus (Mannerheim) [new combination by Harold 1869a: 123].
Chalepides dilatatus (Mannerheim) [new combination by Arrow 1937b: 18].

#### Types.

Lectotype ♀ at ZMH (Endrődi 1966).

#### Distribution.

BRAZIL: Minas Gerais, São Paulo.

#### References.

Mannerheim 1829, Burmeister 1847, Harold 1869a, Arrow 1937b, Endrődi 1966, 1985a, Joly and Escalona 2002a, Krajcik 2005, 2012.

### Chalepides eucephalus

Taxon classificationAnimaliaColeopteraScarabaeidae

(Casey, 1915)

Parachalepus (Chalepides) eucephalus Casey, 1915: 176–177 [original combination].
Chalepides eucephalus (Casey) [new combination by Prell 1936: 151].

#### Types.

Holotype ♀ at USNM (Endrődi 1966, Joly and Escalona 2002a).

#### Distribution.

BRAZIL: Espírito Santo. PARAGUAY: Alto Paraná.

#### References.

Casey 1915, Prell 1936, Endrődi 1966, 1981, 1985a, Joly and Escalona 2002a, Krajcik 2005, 2012.

### Chalepides euhirtus

Taxon classificationAnimaliaColeopteraScarabaeidae

Prokofiev, 2012

Chalepides euhirtus Prokofiev, 2012: 1–2 [original combination].

#### Types.

Holotype ♀ at IEE (Prokofiev 2012).

#### Distribution.

Peru: Junín.

#### References.

Prokofiev 2012.

#### Remarks.

Based on the original description and figures of the holotype, *C. euhirtus* was described from a misidentified female specimen of *Aspidolea fuliginea*.

### Chalepides fuliginosus

Taxon classificationAnimaliaColeopteraScarabaeidae

(Burmeister, 1847)

Chalepus fuliginosus Burmeister, 1847: 78 [original combination].
Dyscinetus fuliginosus (Burmeister) [new combination by Harold 1869a: 123].
Parachalepus (Chalepides) fuliginosus (Burmeister) [new combination and new subgeneric classification by Casey 1915: 176].
Chalepides fuliginosus (Burmeister) [new combination by Prell 1936: 151].

#### Types.

Lectotype ♀ at MLUH (Endrődi 1966).

#### Distribution.

ARGENTINA: Misiones. BOLIVIA: Santa Cruz. BRAZIL: Espírito Santo, Minas Gerais, Paraná, Rio de Janeiro, Rio Grande do Sul, São Paulo, Santa Catarina. CHILE: Santiago. URUGUAY: Artigas.

#### References.

Burmeister 1847, Harold 1869a, Casey 1915, Prell 1936, Arrow 1937b, Blackwelder 1944, Endrődi 1966, 1985a, Joly and Escalona 2002a, Grossi et al. 2011, Krajcik 2005, 2012.

#### Remarks.

*Chalepides fuliginosus* was collected at lights at night in Wakayama, Japan, but a population has not established there (Kusui 1992).

### Chalepides howdenorum

Taxon classificationAnimaliaColeopteraScarabaeidae

Joly & Escalona, 2002

Chalepides howdenorum Joly & Escalona, 2002a: 42, 43, 57–59 [original combination].

#### Types.

Holotype ♂ at CNC (Joly and Escalona 2002a).

#### Distribution.

BOLIVIA: Beni.

#### References.

Joly and Escalona 2002a, Krajcik 2005, 2012.

### Chalepides hydrophiloides

Taxon classificationAnimaliaColeopteraScarabaeidae

(Burmeister, 1847)

Chalepus hydrophiloides Burmeister, 1847: 77 [original combination].
Dyscinetus hydrophiloides (Burmeister) [new combination by Harold 1869a: 123].
Dyscinetus barbatus (Fabricius) [synonymy by Bates 1888: 313].
Parachalepus (Parachalepus) hydrophiloides (Burmeister) [revalidated status, new combination, and new subgeneric classification by Casey 1915: 175].
Chalepides hydrophiloides (Burmeister) [new combination by Prell 1936: 151].
Chalepides barbatus (Fabricius) [synonymy by Arrow 1937b: 18].
Chalepides barbatus hydrophiloides (Burmeister) [new subspecies status by Endrődi 1966: 403].
Chalepides hydrophiloides (Burmeister) [revalidated species status by Joly and Escalona 2002a: 41, 43, 49].
**syn.** Parachalepus (Parachalepus) rhomboidalis Casey, 1915: 175 [original combination].
Chalepides rhomboidalis (Casey) [new combination by Prell 1936: 151].
Chalepides barbatus (Fabricius) [synonymy by Arrow 1937b: 18].
Chalepides barbatus hydrophiloides (Burmeister) [synonymy by Endrődi 1966: 403].
**syn.** Chalepides acillioides Prell, 1937c: 8–9 [original combination].
Chalepides barbatus hydrophiloides (Burmeister) [synonymy by Endrődi 1966: 403].

#### Types.

Lectotype ♀ of *C. hydrophiloides* at MLUH (Endrődi 1966). Type of *C. rhomboidalis* at USNM (Endrődi 1966). Lectotype ♂ of *C. acillioides* at ZMHB (Endrődi 1966).

#### Distribution.

ARGENTINA: Chaco, Santa Fe. BOLIVIA. BRAZIL: Bahia, Espírito Santo, Rio de Janeiro, Rio Grande do Sul, Santa Catarina, São Paulo. PARAGUAY: Distrito Capital. URUGUAY: Artigas, Canelones, Cerro Largo, Durazno, Florida, Maldonado, Montevideo, Rivera, Treinta y Tres.

#### References.

Burmeister 1847, Reiche 1859, Harold 1869b, Steinheil 1874, Bates 1888, Tremoleras 1910, Casey 1915, Heikertinger 1919, Arrow 1937b, Prell 1936, 1937c, Endrődi 1966, 1969a, 1985a, Saenz and Morelli 1985, Joly and Escalona 2002a, Krajcik 2005, 2012.

### Chalepides luridus

Taxon classificationAnimaliaColeopteraScarabaeidae

(Burmeister, 1847)

Chalepus luridus Burmeister, 1847: 78 [original combination].
Dyscinetus luridus (Burmeister) [new combination by Harold 1869a: 123].
Parachalepus (Parachalepus) luridus (Burmeister) [new combination and new subgeneric classification by Casey 1915: 175].
Chalepides luridus (Burmeister) [new combination by Prell 1936: 151].

#### Types.

Invalid neotype ♂ at HNHM (Endrődi Collection) (Endrődi 1966). Joly and Escalona (2002a) listed the housing institution as MLUH.

#### Distribution.

ARGENTINA: Buenos Aires, Chaco Misiones, Corrientes, Entre Ríos, Formosa, Santa Fe, Tucumán. BOLIVIA: Beni, La Paz, Santa Cruz. BRAZIL: Mato Grosso, Rio Grande so Sul. PARAGUAY: Cordillera, Paraguarí. URUGUAY: Canelones, Florida, Montevideo, Salto, Soriano.

#### References.

Burmeister 1847, Harold 1869a, Frenzel 1891, Casey 1915, Prell 1936, Arrow 1937b, Blackwelder 1944, Endrődi 1966, 1969a, 1973a, 1985a, Saenz and Morelli 1985, Joly and Escalona 2002a, Krajcik 2005, 2012.

### Chalepides narcisoi

Taxon classificationAnimaliaColeopteraScarabaeidae

Martínez, 1978b

Chalepides narcisoi Martínez, 1978b: 15–17 [original combination].

#### Types.

Holotype ♂ at MACN (Antonio Martínez Collection) (Martínez 1978b).

#### Distribution.

BRAZIL: Goiás, Mato Grosso, Minas Gerais. PARAGUAY: Caaguazú, Concepción.

#### References.

Martínez 1978b, Endrődi 1985a, Joly and Escalona 2002a, Krajcik 2005, 2012.

### Chalepides osunai

Taxon classificationAnimaliaColeopteraScarabaeidae

Joly & Escalona, 2002

Chalepides osunai Joly & Escalona, 2002a: 42, 43, 55–57 [original combination].

#### Types.

Holotype ♂ at MIZA (Joly and Escalona 2002a).

#### Distribution.

VENEZUELA: Amazonas, Apure, Aragua, Guárico.

#### References.

Joly and Escalona 2002a, Krajcik 2005, 2012.

### Chalepides unicolor

Taxon classificationAnimaliaColeopteraScarabaeidae

(Endrődi, 1963)

Cyclocephala unicolor Endrődi, 1963: 331 [original combination].
Chalepides unicolor (Endrődi) [new combination by Endrődi 1964: 466].
Chalepides luridus (Burmeister) [synonymy by Endrődi 1966: 408].
Chalepides unicolor (Endrődi) [revalidated species status by Joly and Escalona 2002a: 42, 43, 64].

#### Types.

Holotype ♂ at ZSMC (Endrődi 1966).

#### Distribution.

ARGENTINA: La Rioja. BOLIVIA: Beni, La Paz, Santa Cruz. BRAZIL: Amapá, Amazonas, São Paulo, Rondônia.

#### References.

Pike et al. 1976, Endrődi 1963, 1964, 1966, 1985a, Joly and Escalona 2002a, Krajcik 2005, 2012.

### Genus Cyclocephala

Taxon classificationAnimaliaColeopteraScarabaeidae

Dejean, 1821

Cyclocephala Dejean, 1821: 57 [original useage].
**syn.** Aclinidia Casey, 1915: 113 [original usage]. Type species: Melolontha castanea Olivier, by original designation.
Cyclocephala (Aclinidia) Casey [new subgenus status by Arrow 1937b: 8].
Cyclocephala Dejean [synonymy by Endrődi 1966: 34].
**syn.** Albridarollia Bolívar y Pieltain, Jiménez-Asúa, & Martínez, 1963: 182 [original usage]. Type species: Albridarollia ocellata Bolívar y Pieltain, Jiménez-Asúa, & Martínez, by original designation.
Cyclocephala Dejean [synonymy by Endrődi 1966: 34].
**syn.** Aspidolella Prell, 1936: 374 [original usage]. Proposed as a subgenus, replacement name for the subgenus Aspidolites Höhne. Type species: Aspidolea (Aspidolites) atricollis Höhne, by monotypy.
Cyclocephala Dejean [synonymy by Endrődi 1966: 61].
**syn.** Diapatalia Casey, 1915: 111 [original usage]. Type species: Cyclocephala discicollis Arrow, by original designation.
Cyclocephala (Diapatalia) Casey [new subgenus status by Arrow 1937b: 8].
Cyclocephala Dejean [synonymy by Endrődi 1966: 33].
**syn.** Dichromina Casey, 1915: 112 [original usage]. Type species: Cyclocephala dimidiata Burmeister, by original designation.
Cyclocephala (Dichromina) Casey [new subgenus status by Arrow 1937b: 8].
Cyclocephala Dejean [synonymy by Saylor 1945: 380].
**syn.** Graphalia Casey, 1915: 159 [original usage]. Proposed as a subgenus of Ochrosidia. Type species: not yet designated.
Cyclocephala (Graphalia) Casey [new subgenus classification by Arrow 1937b: 8].
Cyclocephala Dejean [synonymy by Endrődi 1966: 34].
**syn.** Halotosia Casey, 1915: 113 [original usage]. Type species: Cyclocephala fasciolata Bates, by original designation.
Cyclocephala (Halotosia) Casey [new subgenus status by Arrow 1937b: 8].
Cyclocephala Dejean [synonymy by Endrődi 1966: 62].
**syn.** Homochromina Casey, 1915: 111 [original usage]. Type species: Homochromina divisa Casey, by original designation.
Cyclocephala (Homochromina) Casey [new subgenus status by Arrow 1937b: 8].
Cyclocephala Dejean [synonymy by Endrődi 1966: 34].
**syn.** Isocoryna Casey, 1915: 136 [original usage]. Proposed as a subgenus. Type species: Cyclocephala (Isocoryna) jalapensis Casey, by monotypy.
Cyclocephala Dejean [synonymy by Endrődi 1966: 62].
**syn.** Mimeoma Casey, 1915: 111 [original usage]. Type species: Cyclocephala maculata Burmeister, by original designation.
Cyclocephala Dejean [synonymy by Moore et al. 2015: 898].
**syn.** Mononidia Casey, 1915: 110 [original usage]. Type species: Cyclocephala carbonaria Arrow, by original designation.
Cyclocephala (Mononidia) Casey [new subgenus status by Arrow 1937b: 8].
Cyclocephala Dejean [synonymy by Endrődi 1966: 33].
**syn.** Ochrosidia Casey, 1915: 112 [original usage]. Type species: Melolontha immaculata Olivier, by original designation.
Cyclocephala (Ochrosidia) Casey [new subgenus status by Arrow 1937b: 8].
Cyclocephala Dejean [synonymy by Saylor 1945: 380].
**syn.** Paraclinidia Martínez, 1965b: 13 [original usage]. Proposed as a subgenus. Type species: Cyclocephala (Paraclinidia) endrodii Martínez, by original designation.
Cyclocephala Dejean [synonymy by Endrődi 1966: 34].
**syn.** Plagiosalia Casey, 1915: 135 [original usage]. Proposed as a subgenus. Type species: not yet designated.
Cyclocephala Dejean [synonymy by Endrődi 1966: 62].
**syn.** Spilosota Casey, 1915: 112 [original usage]. Type species: Spilosota nubeculina Casey, by original designation.
Cyclocephala (Spilosota) Casey [new subgenus status by Arrow 1937b: 8].
Cyclocephala Dejean [synonymy by Saylor 1945: 380].
**syn.** Stigmalia Casey, 1915: 111 [original usage]. Type species: Cyclocephala mafaffa Burmeister, by original designation.
Cyclocephala (Stigmalia) Casey [new subgenus status by Arrow 1937b: 8].
Cyclocephala Dejean [synonymy by Endrődi 1966: 33].
**syn.** Surutoides Endrődi, 1981: 198 [original usage]. Type species: Surutoides mirabilis Endrődi, by original designation.
Cyclocephala Dejean [synonymy by Dechambre 1991a: 282].

#### Type species.

*Melolontha signata* Fabricius, subsequent designation by Casey 1915: 112.

#### Keys.

LeConte 1863 (North America), Horn 1871 (North America), Wickham 1894 (Canada), Blatchley 1910 (Indiana, USA), Dawson 1922 (Nebraska, USA), Hayes 1928, 1929 (larvae, USA), Chapin 1932 (Cuba), Saylor 1937 (California, USA), Sanderson 1940 (Arkansas, USA), Saylor 1945 (USA), Paulian 1947 (French Antilles), Arnett 1968 (USA), Chalumeau and Gruner 1977 (French Antilles), Cartwright and Chalumeau 1978 (Dominica), Morón 1979 (Veracruz, Mexico), Dechambre 1980, Martínez and Morón 1984, Morón et al. 1985 (Chiapas, Mexico), Morón et al. 1988 (Jalisco, Mexico), Morón and Deloya 1991 (Durango, Mexico), Morelli 1989, 1991(larvae, Uruguay), Dechambre 1992, Delgado and Castañeda 1994, Ratcliffe 1991 (Nebraska, USA), Ratcliffe 1992a, Ratcliffe 1992b (Brazil), Deloya and Morón 1994 (Morelos, Mexico), Dupuis 1996, Dechambre 1997, Morón et al. 1998 (Nayarit, Mexico), Joly 2000a, Ratcliffe 2003 (Costa Rica and Panama), Reyes Novelo and Morón 2005 (Yucatán, Mexico), Pacheco-F. et al. 2006 (Guerrero, Mexico), Bran et al. 2006 (larvae), Ratcliffe and Cave 2006 (Honduras, Nicaragua, and El Salvador), Ratcliffe and Paulsen 2008 (Nebraska, USA), Dupuis 2008, Joly 2009, Gasca-Álvarez and Amat-García 2010 (Colombia), Stechauner-Rohringer and Pardo-Locarno 2010 (Larvae, Colombia), Mondaca 2011 (Chile), Mora-Aguilar and Delgado 2012, Ratcliffe et al. 2013 (Mexico, Guatemala, and Belize), Pardo-Locarno 2013 (Colombia), Albuquerque et al. 2014 (larvae), Souza et al. 2014a (larvae), Ratcliffe and Cave 2015 (West Indies), Ratcliffe and Cave 2017 (USA and Canada), Villalobos-Moreno et al. 2017 (Colombia), Romero-López and Morón 2017 (Mexico).

#### Valid taxa.

359 species and subspecies.

#### Remarks.

The generic and subgeneric synonyms of *Cyclocephala* have been treated unevenly in the literature. This makes summarizing the synonymies confusing. Casey (1915) proposed most of the generic-level synonyms of *Cyclocephala*, and the usefulness of these groups was first discussed in detail by Arrow (1937a). Arrow (1937b) went on to treat all of Casey’s new genus-group names as subgenera of *Cyclocephala*, except for *Mimeoma* which he accepted as valid. However, Arrow (1937b) did not clearly place any *Cyclocephala* species into these subgenera in his catalog of Dynastinae. Some authors following Arrow used these subgenera. For example, Saylor (1937) treated *Spilosota* as a valid subgenus of *Cyclocephala*. However, Saylor (1945) later abandoned use of the subgenera (at least in North American taxa) and listed *Dichromina*, *Ochrosidia*, and *Spilosota* in synonymy with *Cyclocephala*. Endrődi (1966) was the first author to discuss these generic groups in totality and proposed many synonyms in the early portion of his monograph. However, Endrődi (1966) impled later in the monograph that *Aclinidia*, *Halotosia*, and *Paraclinidia* were valid subgenera without further comment (see Hardy 1991). Endrődi’s (1966) meaning was ambiguous, and subsequent authors have treated all the above generic-group names as synonyms of *Cyclocephala*.

### Cyclocephala abrelata

Taxon classificationAnimaliaColeopteraScarabaeidae

Ratcliffe & Cave, 2002

Cyclocephala abrelata Ratcliffe & Cave, 2002: 155–156 [original combination].

#### Types.

Holotype ♂ at UNSM (Ratcliffe and Cave 2002).

#### Distribution.

HONDURAS: Yoro.

#### References.

Ratcliffe and Cave 2002, 2006, Krajcik 2005, 2012.

### Cyclocephala acoma

Taxon classificationAnimaliaColeopteraScarabaeidae

Ratcliffe, 2008

Cyclocephala acoma Ratcliffe, 2008: 222–224 [original combination].

#### Types.

Holotype ♂ at BMNH (Ratcliffe 2008).

#### Distribution.

BOLIVIA: Santa Cruz.

#### References.

Ratcliffe 2008, Krajcik 2012.

### Cyclocephala acuta

Taxon classificationAnimaliaColeopteraScarabaeidae

Arrow, 1902

Cyclocephala acuta Arrow, 1902: 139–140 [original combination].
Mimeoma acuta (Arrow) [new combination by Endrődi 1966: 361].
Cyclocephala acuta Arrow [revised combination by Moore et al. 2015: 898].

#### Types.

Type at BMNH (Endrődi 1966).

#### Distribution.

BELIZE: Cayo, Toledo. COLOMBIA: Chocó. COSTA RICA: Alajuela, Cartago, Heredia, Limón, Puntarenas. ECUADOR: Guayas. GUATEMALA: Izabal. HONDURAS: Atlántida, El Paraíso, Gracias a Dios, Olancho. MEXICO: Chiapas. NICARAGUA: Matagalpa, RAA Norte, Río San Juan. PANAMA: Bocas del Toro, Darien, Former Canal Zone, Panamá.

#### References.

Arrow 1902, 1937b, Blackwelder 1944, Pike et al. 1976, Endrődi 1966, 1985a, Bullock 1981, Beach 1984, Morón et al. 1985, Thomas 1993, Ratcliffe and Morón 1997, Ratcliffe 2002a, 2003, Restrepo et al. 2003, Ratcliffe and Cave 2006, Neita-Moreno 2011, Krajcik 2005, 2012, Moore 2012, Ratcliffe et al. 2013, Moore et al. 2015, Gasca-Álvarez and Deloya 2016.

### Cyclocephala aequatoria

Taxon classificationAnimaliaColeopteraScarabaeidae

Endrődi, 1963

Cyclocephala aequatoria Endrődi, 1963: 332 [original combination].

#### Types.

Holotype ♀ at HNHM (Endrődi Collection) (Endrődi 1963).

#### Distribution.

ECUADOR: Cañar, Cotopaxi, Esmeraldas, Guayas, Los Ríos, Manabí, Pichincha. GUATEMALA: Alta Verapaz, Izabal. MEXICO: Chiapas, Guerrero, Jalisco, Michoacán, Nayarit, Oaxaca, Veracruz.

#### References.

Barrera 1969, Pike et al. 1976, Dechambre 1982, Endrődi 1963, 1964, 1966, 1985a, Balslev and Henderson 1987, Morón et al. 1988, Ratcliffe 1992c, Thomas 1993, Lobo and Morón 1993, Delgado and Castañeda 1994, Ratcliffe and Morón 1997, Ervik et al. 1999, Navarrete-Heredia et al. 2001, Pacheco-F. et al. 2008, Ulmen et al. 2010, Krajcik 2005, 2012, Ratcliffe et al. 2013, Deloya et al. 2014a, 2016, Romero-López and Morón 2017.

### Cyclocephala affinis

Taxon classificationAnimaliaColeopteraScarabaeidae

Endrődi, 1966

Cyclocephala affinis Endrődi, 1966: 88, 144, 145–146 [original combination].

#### Types.

Holotype ♂ at NHMB (Frey Collection) (Endrődi 1966).

#### Distribution.

BRAZIL: Amazonas. BOLIVIA: La Paz. COLOMBIA: Valle del Cauca. PERU: Cusco, Huánuco, Madre de Dios.

#### References.

Pike et al. 1976, Endrődi 1966, 1985a, Dupuis 1996, Andreazze and Fonseca 1998, Dechambre 1999, Restrepo-Giraldo et al. 2003, Krajcik 2005, 2012, Ratcliffe et al. 2015, Gasca-Álvarez and Deloya 2016.

### Cyclocephala alazonia

Taxon classificationAnimaliaColeopteraScarabaeidae

Ratcliffe, 2003

Cyclocephala alazonia Ratcliffe, 2003: 60, 65, 69, 75, 80–81 [original combination].

#### Types.

Holotype ♂ at MNCR (originally deposited at INBio) (Ratcliffe 2003).

#### Distribution.

COSTA RICA: Alajuela.

#### References.

Ratcliffe 2003, Krajcik 2005, 2012.

### Cyclocephala alexi

Taxon classificationAnimaliaColeopteraScarabaeidae

Ratcliffe & Delgado-Castillo, 1990

Cyclocephala alexi Ratcliffe & Delgado-Castillo, 1990: 48–51 [original combination].

#### Types.

Holotype at UNSM (Ratcliffe and Delgado-Castillo 1990).

#### Distribution.

GUATEMALA: Baja Verapaz, El Progreso, Huehuetenango, Izabal, Petén, Quiché, San Marcos, Suchitepéquez, Zacapa. MEXICO: Chiapas.

#### References.

Ratcliffe and Delgado-Castillo 1990, Delgado and Castañeda 1994, Thomas 1993, Ratcliffe and Morón 1997, Gómez et al. 1999, Méndez-Aguilar et al. 2005, Krajcik 2005, 2012, Ratcliffe et al. 2013.

### Cyclocephala almitana

Taxon classificationAnimaliaColeopteraScarabaeidae

Dechambre, 1992

Cyclocephala almitana Dechambre, 1992: 65–66 [original combination].
**syn.** Cyclocephala dissimulata Ratcliffe, 1992a: 218–219 [original combination].
Cyclocephala almitana Dechambre [synonymy by Ratcliffe 2003: 81].

#### Types.

Holotype ♂ of *C. almitana* at MNHN (Dechambre 1992). Holotype ♂ of *C. dissimulata* at UNSM (Ratcliffe 1992).

#### Distribution.

COLOMBIA: Chocó. COSTA RICA: Alajuela, Cartago, Guanacaste, Heredia, Limón, San José. ECUADOR: Esmeraldas. PANAMA: Chiriquí, Bocas del Toro, Panamá. PERU.

#### References.

Dechambre 1992, Dupuis 1996, Ratcliffe 1992a, 2002a, 2003, Neita-Moreno 2011, Krajcik 2005, 2012, García-López et al. 2013, Ratcliffe et al. 2015, Gasca-Álvarez and Deloya 2016.

### Cyclocephala altamontana

Taxon classificationAnimaliaColeopteraScarabaeidae

Dechambre, 1999

Cyclocephala altamontana Dechambre, 1999: 3–4 [original combination].

#### Types.

Holotype ♂ at MNHN (Dechambre 1999).

#### Distribution.

PERU: Amazonas.

#### References.

Dechambre 1999, Krajcik 2005, 2012, Ratcliffe et al. 2015.

### Cyclocephala alutacea

Taxon classificationAnimaliaColeopteraScarabaeidae

Höhne, 1923

Cyclocephala alutacea Höhne, 1923b: 359–360 [original combination].

#### Types.

Lectotype ♂ at ZMHB (Endrődi 1966).

#### Distribution.

ARGENTINA: Córdoba.

#### References.

Höhne 1923b, Arrow 1937b, Pike et al. 1976, Endrődi 1966, 1985a, Joly 2000a, Krajcik 2005, 2012.

### Cyclocephala alvarengai

Taxon classificationAnimaliaColeopteraScarabaeidae

Dechambre, 1980

Cyclocephala alvarengai Dechambre, 1980: 42–44 [original combination].

#### Types.

Holotype ♂ at MNHN (Dechambre 1980).

#### Distribution.

BRAZIL: Rio de Janeiro.

#### References.

Dechambre 1980, Endrődi 1985a, Krajcik 2005, 2012.

### Cyclocephala amazona amazona

Taxon classificationAnimaliaColeopteraScarabaeidae

(Linnaeus, 1767)

Scarabaeus amazonus Linnaeus, 1767: 551 [original combination].
Melolontha amazona (Linnaeus) [new combination by Schönherr 1817: 188].
Cyclocephala amazona (Linnaeus) [new combination by Burmeister 1847: 45].
Cyclocephala amazona amazona (Linnaeus) [new subspecies status by Endrődi 1966: 147].
**syn.** Cyclocephala (Cyclocephala) auriculata Casey, 1915: 141 [original combination].
Cyclocephala detecta Bates [synonymy by Arrow 1937b: 10].
**syn.** Cyclocephala detecta Bates, 1888: 300 [original combination].
Cyclocephala (Cyclocephala) detecta Bates [new subgeneric classification by Casey 1915: 141].
Cyclocephala detecta Bates [removal of subgeneric classification by Arrow 1937b: 8, 10].
Cyclocephala amazona (Linnaeus) [synonymy by Endrődi 1964: 466].
**syn.** Cyclocephala (Cyclocephala) beaumonti Casey, 1915: 140 [original combination].
Cyclocephala detecta Bates [synonymy by Arrow 1937b: 10].
**syn.** Cyclocephala signata var. inconstans Burmeister, 1847: 45 [original combination].
Cyclocephala (Cyclocephala) inconstans Burmeister [new subgeneric classification and new species status by Casey 1915: 140].
Cyclocephala inconstans Burmeister [removal of subgeneric classification by Arrow 1937b: 8, 11].
Cyclocephala amazona ab. inconstans Burmeister [revised infrasubspecific status by Endrődi 1966: 147].
**syn.** Melolontha pallens Fabricius, 1798: 132 [original combination].
Cyclocephala pallens (Fabricius) [new combination by Burmeister 1847: 46].
Cyclocephala amazona ab. pallens (Fabricius) [new subspecific status by Endrődi 1964: 466].
**syn.** Melolontha uncinata Illiger, 1802b: 49 [original combination].
Cyclocephala signata (Fabricius) [synonymy by Arrow 1937b: 16].
**syn.** Melolontha signata Fabricius, 1781: 39 [original combination].
Cyclocephala signata (Fabricius) [new combination by Burmeister 1847: 43].
Cyclocephala (Cyclocephala) signata (Fabricius) [new subgeneric classification by Casey 1915: 137].
Cyclocephala signata (Fabricius) [removal of subgeneric classification by Arrow 1937b: 8, 16].
Cyclocephala amazona signata (Fabricius) [new subspecies status by Endrődi 1964: 466].
Cyclocephala amazona (Linnaeus) [synonymy by Ratcliffe 2003: 83].
**syn.** Scarabaeus nigrocephalus DeGeer, 1774: 321 [original combination].
Melolontha signata Fabricius [synonymy by Illiger 1802b: 49].

#### Types.

Invalid neotype of *S. amazonus* at HNHM (Endrődi Collection) (Endrődi 1966). Type of *M. pallens* deposited at ZMUK, now housed at ZMUC (Endrődi 1966). Type of *S. nigrocephalus* at NHRS (Endrődi 1966). Type of *M. uncinata* is missing (Endrődi 1966). Type of *C. inconstans* at MLUH (Endrődi 1966). Type of *C. detecta* at BMNH (Endrődi 1966). Types of *C. beaumonti* at USNM (Endrődi 1966). Type of *C. auriculata* at USNM (Endrődi 1966).

#### Distribution.

BARBADOS. BOLIVIA: Beni, La Paz, Santa Cruz. BRAZIL: Amazonas, Paraná, Pernambuco, Piauí, Santa Catarina, São Paulo. CHILE. COLOMBIA: Amazonas, Antioquia, Bolívar, Boyacá, Caldas, Caquetá, Cauca, Cesar, Chocó, Córdoba, Cundinamarca, Guajira, Magdalena, Meta, Nariña, Quindío, Risaralda, Santander, Sucre, Tolima, Valle del Cauca. COSTA RICA: Alajuela, Cartago, Guanacaste, Heredia, Limón, Puntarenas. CUBA: Holguin. DOMINICAN REPUBLIC: Samana. ECUADOR: Cañar, Chimborazo, Los Ríos. FRENCH GUIANA: Cayenne, Sinnamary, St.-Laurent du Maroni. GRENADA: St. Andrew, St. George, St. John. GUYANA: Demerara-Mahaica. HAITI: Ouest. Panama: Bocas del Toro, Chiriquí, Coclé, Colón, Former Canal Zone, Panamá, San Blas. PARAGUAY. PERU: Lima, Loreto, San Martín. SURINAME: Paramaribo. ST. BARTHÉLEMY. ST. MARTIN. TRINIDAD AND TOBAGO: Trinidad, Tobago. VENEZUELA: Aragua, Capital District.

#### References.

Linnaeus 1767, DeGeer 1774, Fabricius 1781, 1798, Illiger 1802b, Schönherr 1817, Dejean 1821, 1833, 1836b, Drury 1770, 1837, Sturm 1843, Burmeister 1847, Gerstaecker 1866, Harold 1869b, Stahl 1882, Bates 1888, Gundlach 1891, Leng and Mutchler 1914, Casey 1915, Stahl and Scaramuzza 1929, Arrow 1900, 1937b, Martorell 1939, Martorell and Salas 1939, Blackwelder 1944, Gruner 1971, Martínez 1975b, Pike et al. 1976, Mora-Urpí and Solís 1980, Bullock 1981, Mora-Urpí 1982, Remillet et al. 1982, Pava-O. et al. 1983, Beach 1982, 1984, Henderson 1984, Gottsberger 1986, Endrődi 1963, 1964, 1966, 1979, 1985a, Villalta 1988, Posada Ochoa 1989, Rickson et al. 1990, Silberbauer-Gottsberger 1990, Murray 1993, Bernal and Ervik 1996, Woodruff et al. 1998, Dechambre 1999, Ervik et al. 1999, Johnson and Nicolson 2001, Peck et al. 2002, Ratcliffe 2002a, 2003, Restrepo et al. 2003, Bouchard and Bjorndal 2006, Fernández García 2006, Neita Moreno et al. 2006, Pardo-Locarno et al. 2005a, 2008, Útima and Vallejo 2008, Núñez-Avellaneda and Neita-Moreno 2009, Neita-Moreno 2011, Krajcik 2005, 2012, Breeschoten et al. 2013, Yepes-Rodriguez et al. 2013, López-García et al. 2015, Ratcliffe and Cave 2015, Ratcliffe et al. 2015, Ponchel 2006, 2011, 2015, Gasca-Álvarez and Deloya 2016, Peck 2016, Villalobos-Moreno et al. 2016, 2017.

#### Remarks.

The distribution of *C. amazona amazona* is obscured due to historical confusion of this species with *C. multiplex*. The data reported here follow Ratcliffe (2003), which hypothesized that *C. amazona amazona* occurs in South America north through Costa Rica and the West Indies.

### Cyclocephala amazona boliviensis

Taxon classificationAnimaliaColeopteraScarabaeidae

Höhne, 1923

Cyclocephala signata boliviensis Höhne, 1923b: 354–355 [original combination].
Cyclocephala signata var. boliviensis Höhne [new infrasubspecific status by Arrow 1937b: 16].
Cyclocephala amazona boliviensis Höhne [revalidated subspecies status by Endrődi 1966: 147].

#### Types.

Type at ZMHB (Endrődi 1966).

#### Distribution.

BOLIVIA: Beni, La Paz, Santa Cruz. BRAZIL: Amazonas, Mato Grosso, Rondônia.

#### References.

Höhne 1923b, Arrow 1937b, Blackwelder 1944, Pike et al. 1976, Endrődi 1966, 1973a, 1985a, Krajcik 2005, 2012.

### Cyclocephala amblyopsis

Taxon classificationAnimaliaColeopteraScarabaeidae

Bates, 1888

Cyclocephala amblyopsis Bates, 1888: 307–308 [original combination].

#### Types.

Type at BMNH (Endrődi 1966).

#### Distribution.

BOLIVIA: Santa Cruz. BRAZIL: São Paulo. COLOMBIA: Antioquia, Cauca, Chocó, Córdoba, Nariña, Risaralda, Tolima, Valle del Cauca. COSTA RICA: Alajuela, Cartago, Guanacaste, Heredia, Limón, Puntarenas, San José. ECUADOR: Imbabura. EL SALVADOR: Ahuachapán, La Libertad. GUATEMALA: Guatemala, Izabal, Quetzaltenango, San Marcos, Suchitepéquez, Zacapa. HONDURAS: Atlántida, Copán, Cortés, El Paraíso, Olancho, Santa Bárbara, Yoro. MEXICO: Chiapas. NICARAGUA: Chontales, Matagalpa, RAA Norte. PANAMA: Bocas del Toro, Chiriquí, Coclé, Colón, Darien, Panamá, Veraguas. PERU: Cusco.

#### References.

Bates 1888, Arrow 1937b, Blackwelder 1944, Barrera 1969, Martínez 1975b, Pike et al. 1976, Endrődi 1966, 1985a, Valerio 1984, 1988, Young 1986, 1988a, b, 1990, Maes 1987, 1994, Morón 1997, Ratcliffe and Morón 1997, Croat 1997, Beath 1998, 1999, Morón-Ríos and Morón 2001, Ratcliffe 2002a, 2003, Restrepo et al. 2003, García-Robledo et al. 2004, 2005, Ratcliffe and Cave 2006, Pacheco-F. et al. 2008, Pardo-Locarno et al. 2005a, 2008, García-Robledo 2010, Neita-Moreno 2011, Krajcik 2005, 2012, Ratcliffe et al. 2013, García-López et al. 2013, Gasca-Álvarez and Deloya 2016.

### Cyclocephala ampliata

Taxon classificationAnimaliaColeopteraScarabaeidae

Bates, 1888

Cyclocephala ampliata Bates, 1888: 311 [original combination].

#### Types.

Type at BMNH (Endrődi 1966).

#### Distribution.

COSTA RICA: Heredia, Limón. NICARAGUA: Chontales. PANAMA: Bocas de Toro, Chiriquí.

#### References.

Bates 1888, Arrow 1937b, Blackwelder 1944, Pike et al. 1976, Endrődi 1966, 1985a, Young 1990, Maes 1987, 1994, Croat 1997, Beath 1998, 1999, Ratcliffe 1992a, 2002a, 2003, Ratcliffe and Cave 2006, Krajcik 2005, 2012.

### Cyclocephala amplitarsis

Taxon classificationAnimaliaColeopteraScarabaeidae

Ratcliffe, 1992

Cyclocephala amplitarsis Ratcliffe, 1992b: 179–180 [original combination].

#### Types.

Holotype ♂ at UNSM (Ratcliffe 1992b).

#### Distribution.

BRAZIL: Rondônia.

#### References.

Ratcliffe 1992b, Krajcik 2005, 2012.

### Cyclocephala anibali

Taxon classificationAnimaliaColeopteraScarabaeidae

Joly, 2009

Cyclocephala anibali Joly, 2009: 49, 62–64 [original combination].

#### Types.

Holotype ♂ at MIZA (Joly 2009).

#### Distribution.

VENEZUELA: Apure.

#### References.

Joly 2009, Krajcik 2012.

### Cyclocephala antoinei

Taxon classificationAnimaliaColeopteraScarabaeidae

Dupuis, 2018

Cyclocephala antoinei Dupuis, 2018: 2–4 [original combination].

#### Types.

Holotype ♂ at MNHN (Dupuis 2018).

#### Distribution.

VENEZUELA: Apure (Dupuis 2018).

#### References.

Dupuis 2018.

### Cyclocephala aravaipensis

Taxon classificationAnimaliaColeopteraScarabaeidae

Ratcliffe, 1992

Cyclocephala aravaipensis Ratcliffe, 1992c: 253–255 [original combination].

#### Types.

Holotype ♂ at FSCA (Ratcliffe 1992c).

#### Distribution.

UNITED STATES: Arizona.

#### References.

Ratcliffe 1992c, Poole and Gentili 1996, Smith 2003, 2009, Krajcik 2005, 2012, Ratcliffe and Cave 2017.

### Cyclocephala arenosa

Taxon classificationAnimaliaColeopteraScarabaeidae

Howden & Endrődi, 1966

Cyclocephala arenosa Howden & Endrődi, 1966: 296–298 [original combination].

#### Types.

Holotype ♂ at CAS (Howden and Endrődi 1966).

#### Distribution.

MEXICO: Sonora.

#### References.

Howden and Endrődi 1966, Martínez 1968a, Pike et al. 1976, Endrődi 1985a, Ratcliffe and Morón 1997, Smith 2003, 2009, Krajcik 2005, 2012, Ratcliffe et al. 2013.

### Cyclocephala arnaudi

Taxon classificationAnimaliaColeopteraScarabaeidae

Dechambre, 1980

Cyclocephala arnaudi Dechambre, 1980: 44 [original combination].
**syn.** Cyclocephala carlsoni Ratcliffe, 2008: 224–226 [original combination].
Cyclocephala arnaudi Dechambre [synonymy by Ponchel 2010: 172].

#### Types.

Holotype ♂ of *C. arnaudi* at MNHN (Dechambre 1980). Holotype ♂ of *C. carlsoni* at UNSM (Ratcliffe 2008).

#### Distribution.

FRENCH GUIANA: Cayenne, Roura, St.-Laurent du Maroni.

#### References.

Dechambre 1980, Endrődi 1985a, Ratcliffe 2008, Ponchel 2011, Krajcik 2005, 2012, Dupuis 2018.

### Cyclocephala arrowiana

Taxon classificationAnimaliaColeopteraScarabaeidae

Martínez, 1967

Cyclocephala arrowiana Martínez, 1967: 127–131 [original combination].

#### Types.

Holotype ♂ at MACN (Antonio Martínez Collection) (Martínez 1967).

#### Distribution.

BOLIVIA: Cochabamba.

#### References.

Martínez 1967, Pike et al. 1976, Dechambre 1979a, Endrődi 1985a, Ratcliffe 1992a, Dechambre 1999, Krajcik 2005, 2012.

### Cyclocephala atricapilla

Taxon classificationAnimaliaColeopteraScarabaeidae

Mannerheim, 1829

Cyclocephala atricapilla Mannerheim, 1829: 53–53 [original combination].
Stigmalia atricapilla (Mannerheim) [new combination by Casey 1915: 123].
Cyclocephala atricapilla Mannerheim [revised combination by Arrow 1937b: 8].
**syn.** Cyclocephala pinguis Höhne, 1923b: 365–366 [original combination].
Cyclocephala atricapilla Mannerheim [synonymy by Endrődi 1964: 466].

#### Types.

Lectotype ♂ of *C. atricapilla* at ZMH (Endrődi 1966). Lectotype of *C. pinguis* at ZMHB (Endrődi 1966).

#### Distribution.

ARGENTINA: Entre Ríos, Mendoza, Salta, Santa Fe. BOLIVIA. BRAZIL: Bahia, Distrito Federal, Goiás, Maranhão, Mato Grosso, Mato Grosso do Sul, Minas Gerais, São Paulo. COLOMBIA: Córdoba. PARAGUAY: Cordillera, Paraguarí. VENEZUELA: Bolívar.

#### References.

Mannerheim 1829, Burmeister 1847, Harold 1869b, Casey 1915, Luederwaldt 1916, Höhne 1923b, Arrow 1937b, Blackwelder 1944, Martínez 1975b, Pike et al. 1976, Dechambre 1980, Endrődi 1963, 1964, 1966, 1985a, Ramírez 1992, Lunau 2002, Restrepo et al. 2003, Gottsberger and Silberbauer-Gottsberger 1988, 2006, Silberbauer-Gottsberger et al. 2001, 2003, Cavalcante et al. 2009, Krajcik 2005, 2012, Maia et al. 2012, Breeschoten et al. 2013, Paulino-Neto 2014, Gasca-Álvarez and Deloya 2016, Gottsberger 1986, 1988, 1989, 1992, 1999, 2016, Costa et al. 2017.

#### Remarks.

A single no-data specimen of *C. atricapilla* was reported from Mexico (Endrődi 1966), but this species is South American (Ratcliffe et al. 2013).

### Cyclocephala atriceps

Taxon classificationAnimaliaColeopteraScarabaeidae

(Casey, 1915)

Homochromina atriceps Casey, 1915: 164 [original combination].
Cyclocephala atriceps (Casey) [new combination by Arrow 1937b: 8].

#### Types.

Type at USNM (Endrődi 1966).

#### Distribution.

MEXICO: Veracruz.

#### References.

Casey 1915, Arrow 1937b, Blackwelder 1944, Pike et al. 1976, Endrődi 1966, 1985a, Krajcik 2005, 2012.

### Cyclocephala atricolor

Taxon classificationAnimaliaColeopteraScarabaeidae

Chapin, 1932

Cyclocephala atricolor Chapin, 1932: 287, 289–290 [original combination].

#### Types.

Type at USNM (Endrődi 1966).

#### Distribution.

CUBA: Ciego de Ávila, Granma, Guantánamo, Holguin, Pinar del Río, Santiago de Cuba.

#### References.

Chapin 1932, Arrow 1937b, Blackwelder 1944, Howden and Endrődi 1966, Endrődi 1966, 1985a, Fernández García 2006, Krajcik 2005, 2012, Breeschoten et al. 2013, Ratcliffe and Cave 2015, 2017.

### Cyclocephala atripes

Taxon classificationAnimaliaColeopteraScarabaeidae

Bates, 1888

Cyclocephala atripes Bates, 1888: 309 [original combination].
Stigmalia atripes (Bates) [new combination by Casey 1915: 123].
Cyclocephala atripes Bates [revised combination by Arrow 1937b: 8].

#### Types.

Lectotype at BMNH (Endrődi 1966).

#### Distribution.

COLOMBIA: Antioquia, Boyacá, Chocó, Cundinamarca, Magdalena. COSTA RICA: Alajuela, Guanacaste, Heredia, Limón. ECUADOR: Cañar. HONDURAS: Atlántida, Olancho. NICARAGUA: Chontales, Río San Juan. PANAMA: Bocas del Toro, Chiriquí, Colón, Former Canal Zone, Panamá.

#### References.

Bates 1888, Casey 1915, Arrow 1937b, Blackwelder 1944, Pike et al. 1976, Beach 1982, Endrődi 1966, 1975b, 1985a, Young 1986, 1988a, 1990, Maes 1987, 1994, Ratcliffe 1992a, 2002a, 2003, Restrepo et al. 2003, Ratcliffe and Cave 2006, Neita-Moreno 2011, Krajcik 2005, 2012, Moore and Jameson 2013, Gasca-Álvarez and Deloya 2016.

### Cyclocephala aulustjaorum

Taxon classificationAnimaliaColeopteraScarabaeidae

Hielkema, 2017

Cyclocephala pubescens brevis Höhne, 1923b: 373 [original combination].
Cyclocephala pubescens var. brevis Höhne [new infrasubspecific status by Arrow 1937b: 15].
Cyclocephala brevis Höhne [new species status by Ratcliffe 2002a: 28].
Cyclocephala aulustjaorum Hielkema [new replacement name by Hielkema 2017: 8].
**syn.** Cyclocephala pubescens Burmeister, 1847: 68–69 [original combination].
Cyclocephala brevis Höhne [synonymy by Ratcliffe 2002a: 28].

#### Types.

Lectotype ♀ of *C. pubescens* Burmeister at MLUH (Endrődi 1966). Endrődi (1966) did not comment on the location of the Höhne type material.

#### Distribution.

COLOMBIA: Amazonas, Antioquia, Chocó, Cundinamarca, Meta, Risaralda, Santander. COSTA RICA: Alajuela, Guanacaste, Heredia, Limón, San José. GUATEMALA: Baja Verapaz, Izabal, San Marcos, Suchitepéquez, Zacapa. HONDURAS: Atlántida, Cortés, Olancho, Santa Bárbara, Yoro. MEXICO: Chiapas, Jalisco, Michoacán, Oaxaca, Veracruz. NICARAGUA: Jinotega, RAA Norte, Río San Juan. PANAMA: Bocas del Toro, Chiriquí, Colón, Darien, San Blas, Veraguas, Former Canal Zone.

#### References.

Burmeister 1847, Harold 1869b, Höhne 1923b, Arrow 1937b, Blackwelder 1944, Pike et al. 1976, Endrődi 1966, 1985a, Silberbauer-Gottsberger et al. 2001, Ratcliffe 2002a, 2003. Neita-Moreno et al. 2006, Ratcliffe and Cave 2006, Neita-Moreno 2011, Otavo et al. 2013, Ratcliffe et al. 2013, López-García et al. 2015, Moore et al. 2015, Deloya et al. 2014a, 2016, Gasca-Álvarez and Deloya 2016, Hielkema 2017.

### Cyclocephala barrerai

Taxon classificationAnimaliaColeopteraScarabaeidae

Martínez, 1969

Cyclocephala barrerai Martínez, 1969: 2–5 [original combination].
**syn.** Cyclocephala pasadenae mexica Martínez, 1969: 5–6 [original combination].
Cyclocephala barrerai Martínez [synonymy by Ratcliffe et al. 2013: 114].

#### Types.

Holotype ♂ of *C. barrerai* at MCMC (Martínez 1969). Holotype ♂ of *C. pasadenae mexica* at MACN (Martínez 1969).

#### Distribution.

MEXICO: Aguascalientes, Chihuahua, Distrito Federal, Durango, Guanajuato, Jalisco, Michoacán, Morelos, Puebla, Tlaxcala.

#### References.

Martínez 1969, Pike et al. 1976, Endrődi 1985a, Morón and Deloya 1991, Deloya et al. 1993, Lobo and Morón 1993, Ratcliffe and Morón 1997, Marín Jarillo 2001, Navarrete-Heredia et al. 2001, Smith 2003, 2009, Krajcik 2005, 2012, Ratcliffe et al. 2013, Morón et al. 2014, Minor and Morón 2016, Deloya et al. 2016.

### Cyclocephala batesi

Taxon classificationAnimaliaColeopteraScarabaeidae

Delgado & Castañeda, 1994

Cyclocephala batesi Delgado & Castañeda, 1994: 456–458 [original combination].

#### Types.

Holotype ♂ at UVGC (Delgado and Castañeda 1994).

#### Distribution.

GUATEMALA: Baja Verapaz, Chiquimula, Huehuetenango, Izabal, Zacapa. HONDURAS: Copán, Cortés.

#### References.

Delgado and Castañeda 1994, Ratcliffe and Cave 2006, Krajcik 2005, 2012, Ratcliffe et al. 2013, Romero-López and Morón 2017.

### Cyclocephala bella

Taxon classificationAnimaliaColeopteraScarabaeidae

Endrődi, 1969

Cyclocephala bella Endrődi, 1969b: 32–33 [original combination].

#### Types.

Holotype ♂ at MZSP (Pereira Collection) (Endrődi 1969b).

#### Distribution.

BRAZIL: Minas Gerais. São Paulo.

#### References.

Pike et al. 1976, Endrődi 1969b, 1985a, Joly 2000a, Krajcik 2005, 2012.

### Cyclocephala berti

Taxon classificationAnimaliaColeopteraScarabaeidae

Delgado, 1992

Cyclocephala berti Delgado, 1992: 75–78 [original combination].
**syn.** Cyclocephala picopijola Ratcliffe & Cave, 2006: 58, 61, 141–144 [original combination].
Cyclocephala berti Delgado [synonymy by Ratcliffe et al. 2013: 118].

#### Types.

Holotype ♂ of *C. berti* at CMNC (Henry and Anne Howden Collection) (Delgado 1992). Holotype ♂ of *C. picopijola* at UNSM (Ratcliffe and Cave 2006).

#### Distribution.

GUATEMALA: Baja Verapaz, Huehuetenango, Izabal. HONDURAS: Atlántida, Cortés, Yoro. MEXICO: Veracruz.

#### References.

Delgado 1992, Ratcliffe and Cave 2006, Krajcik 2005, 2012, Mora-Aguilar and Delgado 2012, Ratcliffe et al. 2013.

### Cyclocephala bicolor

Taxon classificationAnimaliaColeopteraScarabaeidae

Laporte, 1840

Cyclocephala bicolor Laporte, 1840: 124–125 [original combination].

#### Type.

Type at MNHN (Endrődi 1966).

#### Distribution.

BRAZIL: Acre, Amazonas, Amapá, Bahia, Ceará, Mato Grosso, Pará, Pernambuco, Rio Grande do Norte, Rondônia. BOLIVIA: Beni. COLOMBIA: Caquetá. GUYANA: Cuyuni-Mazaruni, Potaro-Siparuni, Upper Demerara-Berbice. FRENCH GUIANA: Cayenne, Kourou, Mana, Maripasoula, Régina, Sinnamary, St.-Élie, St.-Georges, St.-Laurent du Maroni. PERU: Huánuco. SURINAME: Brokopondo, Marowijne, Paramaribo. VENEZUELA: Amazonas, Bolívar, Táchira.

#### Reference.

Laporte 1840, Burmeister 1847, Harold 1869b, Prudhomme 1906, Bodkin 1919, Arrow 1937b, Gruner 1971, Pike et al. 1976, Endrődi 1964, 1966, 1985a, Remillet et al. 1982, Dechambre 1979a, 1992, Ratcliffe 1992b, Andreazze and Fonseca 1998, Andreazze 2001, Joly 2009, Potascheff 2010, Ponchel 2011, Krajcik 2005, 2012, Breeschoten et al. 2013, Potascheff et al. 2014, Ratcliffe et al. 2015, Gasca-Álvarez and Deloya 2016.

### Cyclocephala bicolorata

Taxon classificationAnimaliaColeopteraScarabaeidae

Endrődi, 1964

Cyclocephala bicolorata Endrődi, 1964: 441–442 [original combination].

#### Types.

Holotype ♂ at HNHM (Endrődi 1964).

#### Distribution.

BRAZIL: Pará. VENEZUELA: Apure, Bolívar.

#### References.

Pike et al. 1976, Endrődi 1964, 1966, 1985a, Dechambre 1979a, 1992, Joly 2009, Krajcik 2005, 2012.

### Cyclocephala bimaculata

Taxon classificationAnimaliaColeopteraScarabaeidae

Dechambre, 1999

Cyclocephala bimaculata Dechambre, 1999: 4–5 [original combination].

#### Types.

Holotype ♂ at MNHN (Dechambre 1999).

#### Distribution.

BOLIVIA: Cochabamba.

#### References.

Dechambre 1999, Krajcik 2005, 2012.

### Cyclocephala binotata

Taxon classificationAnimaliaColeopteraScarabaeidae

Dechambre, 1999

Cyclocephala binotata Dechambre, 1999: 5–6 [original combination].

#### Types.

Holotype ♂ at MNHN (Dechambre 1999).

#### Distribution.

ARGENTINA: Chaco.

#### References.

Dechambre 1999, Krajcik 2005, 2012.

### Cyclocephala bleuzeni

Taxon classificationAnimaliaColeopteraScarabaeidae

Dechambre, 1995

Cyclocephala bleuzeni Dechambre, 1995: 12 [original combination].

#### Types.

Holotype ♂ at MNHN (Dechambre 1995).

#### Distribution.

VENEZUELA: Bolívar.

#### References.

Dechambre 1995, Krajcik 2005, 2012.

### Cyclocephala boliviana

Taxon classificationAnimaliaColeopteraScarabaeidae

Dechambre, 1992

Cyclocephala weidneri boliviana Dechambre, 1992: 71 [original combination].
Cyclocephala boliviana Dechambre [new species status by Dupuis 2018: 4].

#### Types.

Holotype ♂ at MNHN (Dechambre 1992, Dupuis 2018).

#### Distribution.

BOLIVIA: Beni, Cochabamba. PERU: Cusco.

#### References.

Dechambre 1992, Krajcik 2005, 2012, Dupuis 2018.

### Cyclocephala bollei

Taxon classificationAnimaliaColeopteraScarabaeidae

Dechambre & Endrődi, 1984

Cyclocephala bollei Dechambre & Endrődi, 1984: 168–169 [original combination].

#### Types.

Holotype ♂ at MNHN (Dechambre and Endrődi 1984).

#### Distribution.

ARGENTINA: Santiago del Estero.

#### References.

Dechambre and Endrődi 1984, Krajcik 2005, 2012.

### Cyclocephala borburatae

Taxon classificationAnimaliaColeopteraScarabaeidae

Endrődi, 1980

Cyclocephala borburatae Endrődi, 1980: 38 [original combination].

#### Types.

Holotype ♀ in André Gaudaíre-Thore Collection (Sens, France) (Endrődi 1980).

#### Distribution.

VENEZUELA: Carabobo.

#### References.

Endrődi 1980, 1985a, Krajcik 2005, 2012.

### Cyclocephala borealis

Taxon classificationAnimaliaColeopteraScarabaeidae

Arrow, 1911

Cyclocephala borealis Arrow, 1911: 172 [original combination, new replacement name for Cyclocephala villosa Burmeister).
**syn.** Cyclocephala villosaBurmeister 1847: 54 [original combination, homonym of Cyclocephala villosa Blanchard].
Cyclocephala angularis (Knoch) [synonymy by Harold 1869b: 1241].
Cyclocephala villosa Burmeister [revalidated species status by Horn 1871: 336–337].
Ochrosidia (Ochrosidia) villosa (Burmeister) [new combination and new subgeneric classification by Casey 1915: 147].
Cyclocephala borealis Arrow [synonymy by Arrow 1937b: 8].

#### Types.

Invalid neotype ♂ of *C. villosa* at HNHM (Endrődi Collection) (Endrődi 1966).

#### Distribution.

UNITED STATES: Alabama, Arkansas, Connecticut, Delaware, District of Columbia, Georgia, Illinois, Indiana, Kansas, Kentucky, Louisiana, Maryland, Michigan, Mississippi, Missouri, New Jersey, New York, North Carolina, Ohio, Pennsylvania, Rhode Island, South Carolina, Tennessee, Virginia, West Virginia.

#### References.

Knoch 1801, Burmeister 1847, Melsheimer et al. 1853, Reiche 1859, Harold 1869b, Horn 1871, Crotch 1873, Henshaw 1885, Fall 1901, Fall and Cockerell 1907, Blatchley 1910, Swenk 1911, 1913, King 1914, Casey 1915, Hayes 1918, Arrow 1911, 1937b, Leng 1920, Dawson 1922, Leonard 1923, Sim 1934, Anderson 1936, Britton 1932, 1938, Metcalf and Flint 1939, Sanderson 1940, Johnson 1941a, b, Blackwelder 1939, Riegel 1942a, 1948, White 1947, Blackwelder and Blackwelder 1948, Fleming 1948, Adams 1949, Metcalf et al. 1951, Neiswander 1938a, b, 1951, Peterson 1951, Jaques 1951, Bigger and Blanchard 1955, Maddock and Fehn 1958, Harris 1959, Martínez 1954, Dutky 1941, 1963, Polivka 1950, 1952, 1959, 1960, 1965, Howden and Endrődi 1966, Ritcher 1958, 1966, App and Kerr 1969, Beard 1972, Swan and Papp 1972, Woodruff 1973, Gerhardt and Stanghellini 1971, Kawanishi 1974, Pike et al. 1976, MacLean 1977, Pike et al. 1977, Meyer 1978, 1979, 1981, Potter 1980, 1981, 1995, 1998, Warren and Potter 1983, Bulla et al. 1985, Endrődi 1966, 1985a, Tashiro 1987, Klein 1988, Sutton 1988, Gaugler and Georgis 1991, Hardy 1991, McNamara 1991, Youngman et al. 1993, Suggars Downing 1994, Haynes and Potter 1995, Poole and Gentili 1996, Potter et al. 1996, Fuxa 1998, Peck and Thomas 1998, Bhargava 1999, Brandenburg and Royals 1999, Rothwell and Smitley1999, Vittum et al. 1999, Held et al. 2000a, b, c, Harpootlian 2001, DeFoliart 2002, Cappaert and Koppenhöfer 2003, Riley and Wolfe 2003, Rogers et al. 2003, Wilson et al. 2003, Cranshaw 2004, Grewal et al. 2001, 2002, 2004, Rogers and Potter 2004a, b, c, Koppenhöfer and Grewal 2005, Grewal et al. 2005, Shapiro-Ilan and Cottrell 2005, Harris 2006, Georgis et al. 2006, Jackson and Klein 2006, Heller and Walker 1999a, b, c, d, 2000a, b, 2001a, b, c, d, 2002a, b, c, d, e, f, g, 2003a, b, c, d, e, f, g, 2004a, b, c, 2005a, b, c, d, e, Heller and Kline 2005a, b, c, 2007a, b, c, d, e, f, g, h, i, Bixby et al. 2007, An and Grewal 2007, Ratcliffe and Paulsen 2008, Dingman 2008, Koppenhöfer et al. 2002b, 2005, 2006, 2007, 2008, 2013, Counts and Hasiotis 2009, Koppenhöfer and Fuzy 2003a, b, 2006, 2008a, b 2009, Krinsky 2009, Heller et al. 2000a, b, 2006a, b, c, d, e, f, 2008a, b, c, d, e, f, g, h, i, j, k, l, m, 2009a, b, c, d, e, f, g, h, i, j, k, l, Li et al. 2007, 2009, Peck 2009, Popay 2009, Power et al. 2009, Samples et al. 2009, Smith 2003, 2009, Bélair et al. 2010, Ennis et al. 2010, Holmstrup et al. 2010, Mashtoly et al. 2009, 2010, Redmond and Potter 2010, Jordan et al. 2012, Krajcik 2005, 2012, Redmond et al. 2012a, b, Bousquet et al. 2013, Gardner 2013, Guo et al. 2013, Ranger et al. 2009, 2013, Williamson et al. 2004, 2008, 2013, Hussaini 2014, Anderson et al. 2015, Behle et al. 2015, Chong and Hinson 2015, Renkema et al. 2015, Shetlar and Andon 2013, 2015, An and Grewal 2016, Behle and Goett 2016, Christians et al. 2016, Dossey et al. 2016, Gyawaly et al. 2016, Koppenhöfer and Wu 2016, Mitasuhashi 2016, Park and Tak 2016, Schrader et al. 2016, Subramanian and Muthulakshmi 2016, Del Valle et al. 2017, Ratcliffe and Cave 2017.

#### Remarks.

A specimen of *C. borealis* was reported from Mexico (Durango), but this record is considered erroneous (Endrődi 1966, 1985a, Ratcliffe and Morón 1997, Ratcliffe et al. 2013). Records for Iowa, Nebraska, and Texas for *C. borealis* are doubtful or spurious (Ratcliffe and Cave 2017). *Cyclocephala borealis* has been recorded in Canada (Ontario and Nova Scotia) (McNamara 1991, Bousquet et al. 2013), though major faunistic studies did not report additional data (Ratcliffe and Cave 2017).

### Cyclocephala boucheri

Taxon classificationAnimaliaColeopteraScarabaeidae

Dechambre, 1997

Cyclocephala boucheri Dechambre, 1997: 14, 23–24 [original combination].

#### Types.

Holotype ♂ at MNHN (Dechambre 1997).

#### Distribution.

FRENCH GUIANA: Montsinéry-Tonnegrande, Régina, Roura, St.-Laurent du Maroni.

#### References.

Dechambre 1997, Ponchel 2011, Krajcik 2005, 2012.

### Cyclocephala boulardi

Taxon classificationAnimaliaColeopteraScarabaeidae

Dechambre, 1979

Cyclocephala boulardi Dechambre, 1979a: 161–162 [original combination].

#### Types.

Holotype ♂ at MNHN (Dechambre 1979a).

#### Distribution.

BRAZIL: Amazonas. FRENCH GUIANA. SURINAME.

#### References.

Dechambre 1979a, 1980, Endrődi 1985a, Ratcliffe 1992b, Küchmeister et al. 1998, Silberbauer-Gottsberger et al. 2001, Ponchel 2011, Krajcik 2005, 2012.

### Cyclocephala brasiliana

Taxon classificationAnimaliaColeopteraScarabaeidae

Endrődi, 1966

Cyclocephala brasiliana Endrődi, 1966: 72, 131, 159–160 [original combination].

#### Types.

Holotype ♂ at ZMHB (Endrődi 1966).

#### Distribution.

BRAZIL: Goiás, Rio de Janeiro.

#### References.

Pike et al. 1976, Endrődi 1966, 1985a, Krajcik 2005, 2012.

### Cyclocephala brevipennis

Taxon classificationAnimaliaColeopteraScarabaeidae

Endrődi, 1985

Cyclocephala brevipennis Endrődi, 1985b: 69 [original combination].

#### Types.

Holotype ♂ at JPVC (Colette Voirin) (Endrődi 1985b).

#### Distribution.

ECUADOR: Imbabura, Pichincha, Santo Domingo de los Tsáchilas.

#### References.

Endrődi 1985b, Malý 2006, Krajcik 2005, 2012.

### Cyclocephala brittoni

Taxon classificationAnimaliaColeopteraScarabaeidae

Endrődi, 1964

Cyclocephala brittoni Endrődi, 1964: 438–440 [original combination].

#### Types.

Holotype ♂ at BMNH (Endrődi 1964).

#### Distribution.

COLOMBIA: Antioquia, Boyacá, Chocó, Cundinamarca, Magdalena, Santander, Valle del Cauca. COSTA RICA: Heredia, Limón, Puntarenas, San José. FRENCH GUIANA: Cayenne, Kourou, St.-Élie, St.-Laurent du Maroni. GUYANA: Mahaica-Berbice. PANAMA: Bocas del Toro, Chiriquí, Darien, Former Canal Zone, Panamá. SURINAME. TRINIDAD AND TOBAGO: Trinidad.

#### References.

Martínez 1967, Pike et al. 1976, Dechambre 1979a, Endrődi 1964, 1966, 1969b, 1985a, Bullock 1981, Villalta 1988, Silberbauer-Gottsberger et al. 2001, Ratcliffe 1992a, 2002a, 2003, Restrepo et al. 2003, Neita-Moreno et al. 2006, Ponchel 2011, Neita-Moreno 2011, Krajcik 2005, 2012, Moore 2012, Breeschoten et al. 2013, Gasca-Álvarez 2013, López-García et al. 2015, Gasca-Álvarez and Deloya 2016.

### Cyclocephala burmeisteri

Taxon classificationAnimaliaColeopteraScarabaeidae

Endrődi, 1964

Cyclocephala burmeisteri Endrődi, 1964: 449–451 [original combination].

#### Types.

Holotype ♂ at USNM (Endrődi 1964).

#### Distribution.

BOLIVIA: Santa Cruz.

#### References.

Martínez 1975b, Pike et al. 1976, Endrődi 1964, 1966, 1985a, Krajcik 2005, 2012.

### Cyclocephala caelestis

Taxon classificationAnimaliaColeopteraScarabaeidae

Delgado-Castillo & Ratcliffe, 1990

Cyclocephala caelestis Delgado & Ratcliffe, 1990: 51–56 [original combination].

#### Types.

Holotype ♂ at MXAL (Ratcliffe and Delgado-Castillo 1990).

#### Distribution.

MEXICO: Tamaulipas.

#### References.

Ratcliffe and Delgado-Castillo 1990, Ratcliffe and Morón 1997, Dieringer et al. 1998, 1999, Smith 2003, 2009, Thien et al. 2009, Krajcik 2005, 2012, Ratcliffe et al. 2013.

### Cyclocephala camachicola

Taxon classificationAnimaliaColeopteraScarabaeidae

Ohaus, 1910

Cyclocephala camachicola Ohaus, 1910: 672 [original combination].

#### Types.

Type at SDEI (Endrődi 1966).

#### Distribution.

ECUADOR: Azuay.

#### References.

Ohaus 1910, Arrow 1937a, Blackwelder 1944, Pike et al. 1976, Dechambre and Endrődi 1984, Endrődi 1966, 1975b, 1985a, Krajcik 2005, 2012.

### Cyclocephala capitata

Taxon classificationAnimaliaColeopteraScarabaeidae

Höhne, 1923

Cyclocephala capitata Höhne, 1923a: 253–254 [original combination].

#### Types.

Type specimens, housing institution is uncertain, but Endrődi (1966) thought they should be at ZMHB or ZMUH.

#### Distribution.

MEXICO: Chiapas, Colima, Guerrero, Jalisco, Michoacán, Nayarit, Oaxaca, Sinaloa.

#### References.

Höhne 1923a, Arrow 1937a, Blackwelder 1944, Pike et al. 1976, Martínez and Martínez 1981, Endrődi 1966, 1967b, 1985a, Morón et al. 1988, Ratcliffe and Morón 1997, Navarrete-Heredia et al. 2001, Ramos-Elorduy and Pino Moreno 2004, Krajcik 2005, 2012, Ratcliffe et al. 2013, Deloya et al. 2014a, 2016, Dossey et al. 2016, Romero-López and Morón 2017.

### Cyclocephala carbonaria

Taxon classificationAnimaliaColeopteraScarabaeidae

Arrow, 1911

Cyclocephala carbonaria Arrow, 1911: 173–174 [original combination].
Mononidia carbonaria (Arrow) [new combination by Casey 1915: 114–115].
Cyclocephala carbonaria Arrow [revised combination by Arrow 1937b: 8, 9].
Mononidia carbonaria (Arrow) [revised combination by Bolívar y Pieltain et al. 1963: 185].
Cyclocephala carbonaria Arrow [revised combination by Endrődi 1966: 164].
**syn.** Mononidia carbonaria punctulata Prell, 1934: 162 [original combination].
Cyclocephala carbonaria var. punctulata (Prell) [new combination and new infrasubspecific status by Arrow 1937b: 9].
Cyclocephala carbonaria ab. punctulata (Prell) [revised infrasubspecific status by Endrődi 1964: 466].
Cyclocephala carbonaria Arrow [synonymy by Endrődi 1966: 164].
**syn.** Mononidia carbonaria trachypyga Prell, 1934: 162 [original combination].
Cyclocephala carbonaria Arrow [synonymy by Arrow 1937b: 9].
**syn.** Cyclocephala howdeni Endrődi, 1967b: 83–84 [original combination].
Cyclocephala carbonaria Arrow [synonymy by Ratcliffe 2003: 99].

#### Types.

Type of *C. carbonaria* at BMNH (Endrődi 1966). Holotype ♂ of *C. howdeni* at ZMHB (Endrődi 1967b). Location of the Prell types was not reported by Endrődi (1966).

#### Distribution.

BELIZE: Cayo, Stann Creek. BOLIVIA. COLOMBIA: Boyacá, Chocó, Córdoba, Cundinamarca, Santander, Valle del Cauca. COSTA RICA: Alajuela, Cartago, Guanacaste, Heredia, Limón, Puntarenas. ECUADOR: Guayas, Santo Domingo de los Tsáchilas. GUATEMALA: Alta Verapaz, Huehuetenango, Izabal, Petén, Zacapa. HONDURAS: Atlántida, Colón, Cortés, Olancho, Santa Bárbara, Yoro. MEXICO: Chiapas, Puebla. NICARAGUA: Chontales, Jinotega, RAA Norte, Río San Juan. PANAMA: Bocas del Toro, Chiriquí, Coclé, Colón, Former Canal Zone, Panamá, San Blas. VENEZUELA: Amazonas, Bolívar.

#### References.

Casey 1915, Prell 1934, Arrow 1911, 1937b, Blackwelder 1944, Bolívar y Pieltain et al. 1963, Pike et al. 1976, Endrődi 1964, 1966, 1967b, 1985a, Maes 1987, 1994, Joly 1995a, Dechambre 1991a, 1997, Ratcliffe and Morón 1997, Beath 1998, Ratcliffe 2002a, 2003, Restrepo et al. 2003, Neita-Moreno et al. 2006, Ratcliffe and Cave 2006, Pacheco-F. et al. 2008, Neita-Moreno 2011, Krajcik 2005, 2012, Breeschoten et al. 2013, Ratcliffe and Cave 2013, García-López et al. 2013, Ratcliffe et al. 2013, Yepes-Rodriguez et al. 2013, López-García et al. 2015, Gasca-Álvarez and Deloya 2016.

### Cyclocephala cardini

Taxon classificationAnimaliaColeopteraScarabaeidae

Chapin, 1935

Cyclocephala cardini Chapin, 1935a: 74 [original combination, new replacement name for Cyclocephala signatoides Chapin].
**syn.** Cyclocephala signatoides Chapin, 1932: 287, 289 [original combination, junior homonym of Cyclocephala signatoides Höhne, 1923].

#### Types.

Holotype ♂ at USNM (Endrődi 1966, Ratcliffe and Cave 2015).

#### Distribution.

CUBA: Artemisa, La Habana, Pinar del Río, Santiago de Cuba.

#### References.

Chapin 1932, 1935a, Arrow 1937b, Blackwelder 1944, Bruner et al. 1975, Pike et al. 1976, Endrődi 1966, 1985a, Fernández García 2006, Krajcik 2005, 2012, Breeschoten et al. 2013, Ratcliffe and Cave 2015, 2017.

### Cyclocephala carinatipennis

Taxon classificationAnimaliaColeopteraScarabaeidae

Martínez & Morón, 1984

Cyclocephala carinatipennis Martínez & Morón, 1984: 48–52 [original combination].

#### Types.

Holotype ♂ at MACN (Antonio Martínez Collection).

#### Distribution.

VENEZUELA: Táchira.

#### References.

Martínez and Morón 1984, Krajcik 2005, 2012.

### Cyclocephala cartwrighti

Taxon classificationAnimaliaColeopteraScarabaeidae

Endrődi, 1964

Cyclocephala cartwrighti Endrődi, 1964: 442–444 [original combination].

#### Types.

Holotype ♂ at USNM (Endrődi 1964).

#### Distribution.

BOLIVIA: Beni. FRENCH GUIANA: Saül

#### References.

Pike et al. 1976, Endrődi 1964, 1966, 1985a, Ratcliffe 1992a, Dechambre and Duranton 2005, Ponchel 2011, Krajcik 2005, 2012, Breeschoten et al. 2013.

#### Remarks.

Endrődi (1966, 1985a) reported *C. cartwrighti* from Panama (Canal Zone), but this specimen was misidentified (Ratcliffe 2003). *Cyclocephala cartwrighti* does not occur in Panama (Ratcliffe 2003).

### Cyclocephala casanova

Taxon classificationAnimaliaColeopteraScarabaeidae

Ratcliffe & Cave, 2009

Cyclocephala casanova Ratcliffe & Cave, 2009: 326–328 [original combination].

#### Types.

Holotype ♂ at UNSM (Ratcliffe and Cave 2009).

#### Distribution.

GUATEMALA: Baja Verapaz.

#### References.

Ratcliffe and Cave 2009, Krajcik 2012, Ratcliffe et al. 2013.

### Cyclocephala castanea

Taxon classificationAnimaliaColeopteraScarabaeidae

(Olivier, 1789)

Melolontha castanea Olivier, 1789: 79 [original combination].
Cyclocephala castanea (Olivier) [new combination by Hope 1837: 40].
Aclinidia castanea (Olivier) [new combination by Casey 1915: 113, 165].
Cyclocephala castanea (Olivier) [revised combination by Arrow 1937b: 8, 9].
**syn.** Melolontha elongata Olivier, 1789: 23–24 [original combination].
Cyclocephala castanea (Olivier) [synonymy by Burmeister 1847: 49].
**syn.** Cyclocephala latipes Laporte, 1840: 125 [original combination].
Cyclocephala castanea (Olivier) [synonymy by Burmeister 1847: 49].
**syn.** Melolontha valida Schönherr, 1817: 187 [original combination].
Cyclocephala castanea (Olivier) [synonymy by Burmeister 1847: 49].

#### Types.

Invalid neotype ♂ of *M. castanea* at HNHM (Endrődi Collection) (Endrődi 1966). Status of other types was not reported by Endrődi (1966).

#### Distribution.

BRAZIL: Amapá, Amazonas, Pará. COLOMBIA: Amazonas, Guaviare. GUYANA: Demerara-Mahaica, Upper Demerara-Berbice. FRENCH GUIANA: Cayenne, St.-Laurent du Maroni. SURINAME: Paramaribo. VENEZUELA: Amazonas, Bolívar.

#### References.

Olivier 1789, Schönherr 1817, Dejean 1821, 1833, 1836b, Hope 1837, Laporte 1840, Sturm 1843, Burmeister 1847, Erichson 1848a, Fauvel 1861, Harold 1869b, von Bayern 1897, Knuth et al. 1904, Arcangeli 1908, Ohaus 1909, 1911, Casey 1915, Bodkin 1919, Arrow 1937b, Anonymous 1940, Blackwelder 1944, Gessner 1962, Cramer et al. 1975, Pike et al. 1976, Prance and Anderson 1976, Faegri and van der Pijl 1979, Endrődi 1966, 1969b, 1975c, 1985a, Lachaume 1992, Joly 2000b, Ponchel 2011, Krajcik 2005, 2012, Breeschoten et al. 2013, Otavo et al. 2013, Maia et al. 2014, Gasca-Álvarez and Deloya 2016.

### Cyclocephala castaniella

Taxon classificationAnimaliaColeopteraScarabaeidae

Bates, 1888

Cyclocephala castaniella Bates, 1888: 304 [original combination].
**syn.** Cyclocephala obscurata Endrődi, 1966: 84, 270–271 [original combination].
Cyclocephala castaniella Bates [synonymy by Ratcliffe 2003].

#### Types.

Type of *C. castaniella* at BMNH (Endrődi 1966). Holotype ♂ of *C. obscurata* at ZMHB (Endrődi 1966).

#### Distribution.

COSTA RICA: Alajuela, Cartago, Guanacaste, Heredia, Puntarenas, San José. PANAMA: Chiriquí.

#### References.

Bates 1888, Arrow 1937b, Blackwelder 1944, Martínez 1964, Pike et al. 1976, Endrődi 1966, 1985a, Abarca and Quesada 1997, Vargas and Abarca 1998, Ratcliffe 1992a, 2002a, 2003, Krajcik 2005, 2012.

#### Remarks.

Endrődi (1966, 1985a) reported a single specimen of *C. castaniella* from Brazil with no further details. These data are likely erroneous (Ratcliffe 2003).

### Cyclocephala caussaneli

Taxon classificationAnimaliaColeopteraScarabaeidae

Dechambre, 1999

Cyclocephala caussaneli Dechambre, 1999: 6 [original combination].

#### Types.

Holotype ♂ at MNHN (Dechambre 1999).

#### Distribution.

ARGENTINA: Chaco.

#### References.

Dechambre 1999, Krajcik 2005, 2012.

### Cyclocephala cearae

Taxon classificationAnimaliaColeopteraScarabaeidae

Höhne, 1923

Cyclocephala cearae Höhne, 1923b: 363–364 [original combination].

#### Types.

Lectotype at ZMHB (Endrődi 1966).

#### Distribution.

BRAZIL: Bahia, Ceará, Pernambuco, Rio Grande do Norte, São Paulo.

#### References.

Höhne 1923b, Arrow 1937b, Blackwelder 1944, Pike et al. 1976, Endrődi 1966, 1985a, Krajcik 2005, 2012, Breeschoten et al. 2013, Maia et al. 2012, 2013a, b, 2014, Albuquerque et al. 2016.

#### Remarks.

The specific epithet *cearae* is misspelled as *clarae* in some catalogs (Arrow 1937b, Blackwelder 1944). Krajcik (2005) lists *C. clarae* Arrow as a synonym of *C. cearae* Höhne.

### Cyclocephala celata

Taxon classificationAnimaliaColeopteraScarabaeidae

Dechambre, 1980

Cyclocephala celata Dechambre, 1980: 44–46 [original combination].

#### Types.

Holotype ♂ at MNHN (Dechambre 1980).

#### Distribution.

BRAZIL: Pernambuco, Tocantins. PARAGUAY: Guairá.

#### References.

Dechambre 1980, Endrődi 1985a, Gonçalves and Maia 2006, Maia and Schlindwein 2006, Krajcik 2005, 2012, Maia et al. 2010, 2012, 2013a, b, 2014, Souza et al. 2014b, Gottsberger 2016.

### Cyclocephala cerea

Taxon classificationAnimaliaColeopteraScarabaeidae

Burmeister, 1847

Cyclocephala cerea Burmeister, 1847: 51 [original combination].
Cyclocephala sanguinicollis cerea (Burmeister) [new subspecies status by Endrődi 1966: 301].
Cyclocephala cerea Burmeister [revised species status by Endrődi 1967b: 88].
Cyclocephala sanguinicollis cerea (Burmeister) [revalidated subspecies status by Endrődi 1985a: 115].
Cyclocephala cerea Burmeister [revalidated species status by Ratcliffe and Cave 2015: 75, 83].
**syn.** Cyclocephala sororia Bates, 1888: 303 [original combination].
Cyclocephala cerea Burmeister [synonymy by Ratcliffe and Cave 2015: 83].
**syn.** Cyclocephala flava Dechambre, 1999: 10–11 [original combination].
Cyclocephala sororia Bates [synonymy by Ratcliffe and Cave 2006: 155].
Cyclocephala cerea Burmeister [synonymy by Ratcliffe and Cave 2015: 83].

#### Types.

Lectotype ♀ of *C. cerea* at MLUH (Endrődi 1966). Type of *C. sororia* at BMNH (Endrődi 1966). Holotype ♂ of *C. flava* at MNHN (Dechambre 1999).

#### Distribution.

BELIZE: Toledo. COSTA RICA: Cartago, Guanacaste, Heredia, Puntarenas, San José. CUBA: Camagüey, Ciego de Ávila, Cienfuegos, Guantánamo, Holguin, La Habana, Pinar del Río, Santiago de Cuba, Villa Clara. DOMINICAN REPUBLIC: Azua, Barahona, Independencia, Pedernales, San José de Ocoa, San Juan. GUATEMALA: Baja Verapaz, El Progreso, Escuintla, Guatemala, Huehuetenango, Izabal, Petén, Quiché, Sacatepéquez, San Marcos, Sololá, Suchitepéquez, Zacapa. HONDURAS: Atlántida, Cortés, Francisco Morazán, Ocotepeque. JAMAICA: Clarendon, Manchester, Portland, St. Andrew, St. Catherine, St. Elizabeth, St. James, Westmoreland. MEXICO: Chiapas, Colima, Durango, Guerrero, Hidalgo, Jalisco, Michoacán, Morelos, Nayarit, Oaxaca, Puebla, Querétaro, San Luis Potosí, Sinaloa, Tamaulipas, Veracruz.

#### References.

Burmeister 1847, Harold 1869b, Bates 1888, Leng and Mutchler 1914, Arrow 1937b, Blackwelder 1944, Howden 1970, Endrődi 1966, Endrődi 1967b, 1985a, Thomas 1993, Lobo and Morón 1993, Morón 1994, Ratcliffe and Morón 1997, Dechambre 1999, Navarrete-Heredia et al. 2001, Ratcliffe 2003, Luna et al. 2007, Krajcik 2005, 2012, Breeschoten et al. 2013, Ratcliffe et al. 2013, Ratcliffe and Cave 2006, 2015, Deloya et al. 1993, 2014a, 2016.

### Cyclocephala chalumeaui

Taxon classificationAnimaliaColeopteraScarabaeidae

Martínez, 1978

Cyclocephala chalumeaui Martínez, 1978b: 9–12 [original combination].

#### Types.

Holotype ♂ at MACN (Antonio Martínez Collection) (Martínez 1978b).

#### Distribution.

ECUADOR: Pichincha.

#### References.

Martínez 1978b, Endrődi 1985a, Krajcik 2005, 2012.

### Cyclocephala chera

Taxon classificationAnimaliaColeopteraScarabaeidae

Ratcliffe, 2008

Cyclocephala chera Ratcliffe, 2008: 226–227 [original combination].

#### Types.

Holotype ♀ at USNM (Ratcliffe 2008).

#### Distribution.

GUYANA: Potaro-Siparuni.

#### References.

Ratcliffe 2008, Krajcik 2012.

### Cyclocephala chiquitita

Taxon classificationAnimaliaColeopteraScarabaeidae

Ratcliffe, 2008

Cyclocephala chiquitita Ratcliffe, 2008: 227–229 [original combination].

#### Types.

Holotype ♂ at UNSM (Ratcliffe 2008).

#### Distribution.

ECUADOR: Napo.

#### References.

Ratcliffe 2008, Krajcik 2012.

### Cyclocephala colasi

Taxon classificationAnimaliaColeopteraScarabaeidae

Endrődi, 1964

Cyclocephala colasi Endrődi, 1964: 440–441 [original combination].
**syn.** Cyclocephala hayekaeEndrődi 1966: 92, 212–213 [original combination].
Cyclocephala colasi ab. hayekae Endrődi [new infrasubspecific status by Endrődi 1985a: 110].

#### Types.

Holotype ♂ of *C. colasi* at HNHM (Endrődi Collection) (Endrődi 1964). Holotype ♂ of *C. hayekae* at BMNH (Endrődi 1966).

#### Distribution.

BOLIVIA: Beni. BRAZIL: Amazonas, Pernambuco. COLOMBIA: Casanare. FRENCH GUIANA: Campoi, Cayenne, Kourou, Maripasoula, Sinnamary, St.-Laurent du Maroni. PERU: Loreto. SURINAME. VENEZUELA: Bolívar.

#### References.

Gruner 1971, Pike et al. 1976, Dechambre 1979a, 1980, Dechambre and Endrődi 1984, Endrődi 1964, 1966, 1973a, 1985a, Andreazze and Fonseca 1998, Andreazze 2001, Gibernau et al. 1999, 2000, 2003, Milius 2003, Davis et al. 2008, Seymour et al. 2003, 2009, Breeschoten et al. 2013, Thien et al. 2009, Ponchel 2006, 2011, 2015, Krajcik 2005, 2012, Ratcliffe et al. 2015, Gasca-Álvarez and Deloya 2016.

### Cyclocephala collaris

Taxon classificationAnimaliaColeopteraScarabaeidae

Burmeister, 1847

Cyclocephala collaris Burmeister, 1847: 47 [original combination].
Cyclocephala (Cyclocephala) collaris Burmeister [new subgeneric classification by Casey 1915: 138].
Cyclocephala collaris Burmeister [removal of subgeneric classification by Arrow 1937b: 8, 9].

#### Types.

Lectotype ♂ at MLUH (Endrődi 1966).

#### Distribution.

BRAZIL: Bahia, Rio de Janeiro. ECUADOR. MARTINIQUE. SURINAME. VENEZUELA: Bolívar.

#### References.

Burmeister 1847, Harold 1869b, Bates 1888, Casey 1915, Arrow 1937b, Blackwelder 1944, Martínez 1975b, Pike et al. 1976, Dechambre 1982, Dechambre and Endrődi 1984, Endrődi 1964, 1966, 1985a, Marques and Gil-Santana 2009, Krajcik 2005, 2012, Ratcliffe et al. 2013.

#### Remarks.

A few authors reported *C. collaris* from Guatemala (Alto Verapaz), Honduras, and Belize (Bates 1888, Blackwelder 1944, Endrődi 1966, 1985a). Faunistic studies have not recorded *C. collaris* from these areas, and it is possible that these data are erroneous (Ratcliffe and Cave 2006, Ratcliffe et al. 2013).

### Cyclocephala comata

Taxon classificationAnimaliaColeopteraScarabaeidae

Bates, 1888

Cyclocephala comata Bates, 1888: 305–306 [original combination].
Ochrosidia (Graphalia) comata (Bates) [new combination by Casey 1915: 159].
Cyclocephala comata Bates [revised combination by Arrow 1937b: 8, 9].

#### Types.

Type at BMNH (Endrődi 1966).

#### Distribution.

MEXICO: Durango, Estado de México, Guanajuato, Jalisco, Michoacán, Oaxaca, San Luis Potosí, Tamaulipas.

#### References.

Bates 1888, Casey 1915, Arrow 1937b, Blackwelder 1944, Pike et al. 1976, Endrődi 1966, 1985a, Ratcliffe and Morón 1997, Marín Jarillo 2001, Navarrete-Heredia et al. 2001, Díaz Mederos et al. 2006, Lugo-García et al. 2009, Smith 2003, 2009, Joly 2010, Krajcik 2005, 2012, Ratcliffe et al. 2013, Deloya et al. 2016.

### Cyclocephala compacta

Taxon classificationAnimaliaColeopteraScarabaeidae

Ratcliffe, 2008

Cyclocephala compacta Ratcliffe, 2008: 229–231 [original combination].

#### Types.

Holotype ♂ at UNSM (Ratcliffe 2008).

#### Distribution.

BRAZIL: Rondônia.

#### References.

Ratcliffe 2008, Krajcik 2012.

### Cyclocephala complanata

Taxon classificationAnimaliaColeopteraScarabaeidae

Burmeister, 1847

Cyclocephala complanata Burmeister, 1847: 48–49 [original combination].
Cyclocephala (Plagiosalia) complanata Burmeister [new subgeneric classification by Casey 1915: 135].
Cyclocephala complanata Burmeister [removal of subgeneric classification by Arrow 1937b: 8, 9].
**syn.** Cyclocephala (Plagiosalia) emacerata Casey, 1915: 136 [original combination].
Cyclocephala complanata Burmeister [synonymy by Arrow 1937b: 9].
**syn.** Cyclocephala (Plagiosalia) obliquata Casey, 1915: 135 [original combination].
Cyclocephala complanata Burmeister [synonymy by Arrow 1937b: 9].

#### Types.

Lectotype ♂ of *C. complanata* at MLUH (Endrődi 1966). Types of *C. emacerata* and *C. obliquata* at USNM (Endrődi 1966).

#### Distribution.

BELIZE: Cayo, Orange Walk, Toledo, Stann Creek. COSTA RICA: Alajuela, Cartago, Guanacaste, Heredia, Limón, Puntarenas, San José. EL SALVADOR: Ahuachapán, Chalatenango, San Salvador, Santa Ana. GUATEMALA: Alta Verapaz, Baja Verapaz, El Progreso, Escuintla, Guatemala, Huehuetenango, Izabal, Jutiapa, Petén, Quetzaltenango, Quiché, Retalhuleu, Sacatepéquez, San Marcos, Santa Rosa, Sololá, Suchitepéquez, Zacapa. HONDURAS: Atlántida, Choluteca, Comayagua, Cortés, El Paraíso, Francisco Morazán, Gracias a Dios, Lempira, Olancho, Santa Bárbara, Yoro. MEXICO: Chiapas, Morelos, Oaxaca, Quintana Roo, Tabasco, Veracruz, Yucatán. NICARAGUA: Chontales, Jinotega, Matagalpa, RAA Norte, Río San Juan. PANAMA: Bocas del Toro.

#### References.

Burmeister 1847, Harold 1869b, Bates 1888, Casey 1915, Arrow 1937b, Pike et al. 1976, Endrődi 1966, 1985a, Morón 1979, Maes 1987, Thomas 1993, Lobo and Morón 1993, Ratcliffe and Morón 1997, García-Luna et al. 2002, Alcázar-Ruiz et al. 2003, Ratcliffe 1992c, 2002a, 2003, Ratcliffe and Cave 2006, Pacheco-F. et al. 2008, Krajcik 2005, 2012, Breeschoten et al. 2013, Ratcliffe et al. 2013.

### Cyclocephala concolor

Taxon classificationAnimaliaColeopteraScarabaeidae

Burmeister, 1847

Cyclocephala concolor Burmeister, 1847: 50 [original combination].

#### Types.

Lectotype ♀ at MNHN (Dechambre 1991b). Invalid neotype ♂ at HNHM (Endrődi Collection) (Endrődi 1966).

#### Distribution.

COLOMBIA: Antioquia, Tolima. COSTA RICA: Alajuela, Cartago, Heredia, Puntarenas. GUATEMALA: Alta Verapaz, Baja Verapaz, Escuintla, Huehuetenango, Izabal, Zacapa. HONDURAS: Atlántida, Cortés, Francisco Morazán, Lempira, Ocotepeque, Santa Bárbara, Yoro. MEXICO: Chiapas, Oaxaca. PANAMA: Bocas del Toro. PARAGUAY: Paraguarí.

#### References.

Burmeister 1847, Reiche 1859, Harold 1869b, Arrow 1937b, Blackwelder 1944, Pike et al. 1976, Endrődi 1966, 1985a, Dechambre 1991b, 1992, Ratcliffe and Morón 1997, Ratcliffe 2002a, 2003, Restrepo et al. 2003, Ratcliffe and Cave 2006, Pacheco-F. et al. 2008, Krajcik 2005, 2012, Breeschoten et al. 2013, Ratcliffe et al. 2013, García-López et al. 2013, Gasca-Álvarez and Deloya 2016, Romero-López and Morón 2017.

### Cyclocephala confusa

Taxon classificationAnimaliaColeopteraScarabaeidae

Endrődi, 1966

Cyclocephala confusa Endrődi, 1966: 90, 141, 143, 174–175 [original combination].

#### Types.

Holotype ♂ at NHMB (Frey Collection) (Endrődi 1966).

#### Distribution.

BELIZE: Cayo. COLOMBIA: Antioquia, Santander. COSTA RICA: Alajuela, Cartago, Guanacaste, Heredia, Limón, Puntarenas. EL SALVADOR: Ahuachapán, Morazán. FRENCH GUIANA: Macouria. GUATEMALA: Alta Verapaz, Chiquimula, Izabal, San Marcos. HONDURAS: Atlántida, Copán, Cortés, El Paraíso, Gracias a Dios, Olancho, Yoro. MEXICO: Chiapas, Oaxaca. NICARAGUA: Jinotega, RAA Norte, Río San Juan. PANAMA: Bocas del Toro, Chiriquí, Colón, Former Canal Zone, Panamá. PERU.

#### References.

Gruner 1971, Pike et al. 1976, Endrődi 1966, 1985a, Dechambre 1992, Maes et al. 1997, Ratcliffe 1992a, b, 2002a, 2003, Restrepo et al. 2003, Ratcliffe and Cave 2006, Krajcik 2005, 2012, García-López et al. 2013, Dupuis 2014, Ratcliffe et al. 2013, 2015, Gasca-Álvarez and Deloya 2016.

### Cyclocephala conspicua

Taxon classificationAnimaliaColeopteraScarabaeidae

Sharp, 1877

Cyclocephala conspicua Sharp, 1877: 135 [original combination].
Stigmalia conspicua (Sharp) [new combination by Casey 1915: 123].
Cyclocephala conspicua Sharp [revised combination by Arrow 1937b: 8, 9].
**syn.** Cyclocephala conspicua gregaroides Dechambre, 1992: 71 [original combination].
Cyclocephala conspicua Sharp [synonymy by Ratcliffe 2003: 114].
**syn.** Cyclocephala conspicua fusca Dechambre, 1992: 72 [original combination].
Cyclocephala conspicua Sharp [synonymy by Ratcliffe 2003: 114].

#### Types.

Type of *C. conspicua* at MNHN (Endrődi 1966). Holotype ♂ of *C. conspicua gregaroides* at MNHN (Dechambre 1992). Holotype ♂ of *C. conspicua fusca* at MNHN (Dechambre 1992).

#### Distribution.

BRAZIL: Amazonas. COSTA RICA: Alajuela, Cartago, Guanacaste, Heredia, Puntarenas, San José. ECUADOR: Napo, Pichincha. HONDURAS: El Paraíso. NICARAGUA: Chontales, Jinotega, Matagalpa, RAA Norte. PANAMA: Bocas del Toro, Chiriquí, Coclé, Former Canal Zone, Panamá, Veraguas. PERU.

#### References.

Sharp 1877, Bertkau 1878, Bates 1888, Casey 1915, Arrow 1937b, Blackwelder 1944, Pike et al. 1976, Beach 1982, Endrődi 1966, 1985a, Young 1988a, 1990, Dechambre 1992, Maes 1987, 1994, Croat 1997, Ratcliffe 2002a, 2003, Ratcliffe and Cave 2006, Krajcik 2005, 2012, García-López et al. 2013.

### Cyclocephala contraria

Taxon classificationAnimaliaColeopteraScarabaeidae

Kirsch, 1873

Cyclocephala contraria Kirsch, 1873: 343–344 [original combination].

#### Types.

Lectotype ♂ at MTD (Endrődi 1966).

#### Distribution.

BOLIVIA: La Paz. COLOMBIA: Meta. ECUADOR: Napo. PERU: Pasco.

#### References.

Kirsch 1873, Arrow 1937b, Blackwelder 1944, Martínez 1975b, Pike et al. 1976, Endrődi 1966, 1985a, Restrepo et al. 2003, Ratcliffe 2008, Krajcik 2005, 2012, Breeschoten et al. 2013, Ratcliffe et al. 2015, Gasca-Álvarez and Deloya 2016.

#### Remarks.

Authors since Kirsch (1873) have referred to this species as *C. contracta* without explanation (Arrow 1937b, Blackwelder 1944, Endrődi 1966, 1985a, Restrepo et al. 2003, Ratcliffe et al. 2015). The original and correct spelling is *C. contraria*, as listed in Gasca-Álvarez and Deloya (2016).

### Cyclocephala coriacea

Taxon classificationAnimaliaColeopteraScarabaeidae

Dechambre, 1992

Cyclocephala coriacea Dechambre, 1992: 58 [original combination].

#### Types.

Holotype ♂ at MNHN (Dechambre 1992).

#### Distribution.

ECUADOR: Sucumbíos.

#### References.

Dechambre 1992, Dupuis 2008, 2014, Krajcik 2005, 2012.

### Cyclocephala couturieri

Taxon classificationAnimaliaColeopteraScarabaeidae

Dechambre, 1999

Cyclocephala couturieri Dechambre, 1999: 7 [original combination].

#### Types.

Holotype ♂ at MNHN (Dechambre 1999).

#### Distribution.

PERU: San Martín.

#### References.

Dechambre 1999, Krajcik 2005, 2012, Ratcliffe et al. 2015.

### Cyclocephala crassa

Taxon classificationAnimaliaColeopteraScarabaeidae

Endrődi, 1967

Cyclocephala crassa Endrődi, 1967c: 1–3 [original combination].

#### Types.

Holotype ♂ at ZMHB (Endrődi 1967c).

#### Distribution.

COLOMBIA: Amazonas. ECUADOR.

#### References.

Martínez 1975b, Pike et al. 1976, Endrődi 1967c, 1985a, Restrepo et al. 2003, Krajcik 2005, 2012, Gasca-Álvarez and Deloya 2016.

### Cyclocephala crepuscularis

Taxon classificationAnimaliaColeopteraScarabaeidae

Martínez, 1954

Cyclocephala crepuscularis Martínez, 1954: 17–26 [original combination].

#### Types.

Holotype ♂ at MACN (Antonio Martínez Collection) (Martínez 1954).

#### Distribution.

ARGENTINA: Buenos Aires.

#### References.

Pike et al. 1976, Martínez 1954, 1978a, Endrődi 1966, 1985a, Schawaller 1994, Krajcik 2005, 2012, Breeschoten et al. 2013.

### Cyclocephala cribrata

Taxon classificationAnimaliaColeopteraScarabaeidae

Burmeister, 1847

Cyclocephala cribrata Burmeister, 1847: 69–70 [original combination].

#### Types.

Type at MLUH (Endrődi 1966).

#### Distribution.

COLOMBIA. BRAZIL: Bahia, Espírito Santo, Pernambuco, Rio de Janeiro, Rio Grande do Sul, Santa Catarina, São Paulo.

#### References.

Burmeister 1847, Reiche 1859, Harold 1869b, Luederwaldt 1926, Luederwaldt and Pinto da Fonseca 1922, Arrow 1937b, Blackwelder 1944, Pike et al. 1976, Endrődi 1966, 1985a, Gottsberger and Amaral 1984, Gottsberger 1986, Dechambre 1997, Weber et al. 2001, Restrepo et al. 2003, Malý 2006, Weber 2008, Marques and Gil-Santana 2009, Krajcik 2005, 2012, Gasca-Álvarez and Deloya 2016.

### Cyclocephala curta

Taxon classificationAnimaliaColeopteraScarabaeidae

Bates, 1888

Cyclocephala curta Bates, 1888: 305 [original combination].
**syn.** Cyclocephala fusciventris Arrow, 1902: 139 [original combination].
Cyclocephala curta Bates [synonymy by Endrődi 1964: 466].

#### Types.

Types of *C. curta* and *C. fusciventris* at BMNH (Endrődi 1966).

#### Distribution.

COSTA RICA: Guanacaste. EL SALVADOR: San Salvador. HONDURAS: Comayagua, Cortés, La Paz. MEXICO: Chiapas, Guerrero, Michoacán, Nayarit, Oaxaca, Sinaloa, Veracruz.

#### References.

Bates 1888, Arrow 1902, 1937b, Blackwelder 1944, Martínez 1964, Pike et al. 1976, Endrődi 1964, 1966, 1985a, Ratcliffe and Morón 1997, Ratcliffe 2003, Ratcliffe and Cave 2006, Pacheco-F. et al. 2008, Krajcik 2005, 2012, Breeschoten et al. 2013, Ratcliffe et al. 2013, Deloya et al. 2014a, 2016.

### Cyclocephala dalensi

Taxon classificationAnimaliaColeopteraScarabaeidae

Ponchel, 2009

Cyclocephala dalensi Ponchel, 2009: 184–185 [original combination].

#### Types.

Holotype ♂ in the Yannig Ponchel Collection (Ponchel 2009).

#### Distribution.

FRENCH GUIANA: Maripasoula.

#### References.

Ponchel 2009, 2011, Krajcik 2012.

### Cyclocephala deceptor

Taxon classificationAnimaliaColeopteraScarabaeidae

(Casey, 1915)

Stigmalia deceptor Casey, 1915: 117–118 [original combination].
Cyclocephala mafaffa (Burmeister) [synonymy by Arrow 1937b: 12].
Cyclocephala deceptor (Casey) [revalidated species status by Ratcliffe and Delgado 1990: 43–45].
**syn.** Stigmalia cuernavacana Casey, 1915: 116-117 [original combination].
Cyclocephala mafaffa Burmeister [synonymy by Arrow 1937b: 12].
Cyclocephala mafaffa ab. cuernavacana (Casey) [new infrasubspecific status by Endrődi 1966: 247].
Cyclocephala deceptor (Casey) [synonymy by Ratcliffe and Delgado-Castillo 1990: 43].
**syn.** Stigmalia deficiens Casey, 1915: 117 [original combination].
Cyclocephala mafaffa Burmeister [synonymy by Arrow 1937b: 12].
Cyclocephala deceptor (Casey) [synonymy by Ratcliffe and Delgado-Castillo 1990: 43].
**syn.** Stigmalia fallaciosa Casey, 1915: 117 [original combination].
Cyclocephala mafaffa Burmeister [synonymy by Arrow 1937b: 12].
Cyclocephala mafaffa ab. fallaciosa (Casey) [new infrasubspecific status by Endrődi 1966: 247].
Cyclocephala deceptor (Casey) [synonymy by Ratcliffe and Delgado-Castillo 1990: 43].

#### Types.

These Casey types are at USNM (Endrődi 1966).

#### Distribution.

BELIZE: Cayo. EL SALVADOR: Ahuachapán, La Libertad, La Paz, Morazán, San Salvador, San Vicente, Santa Ana, Sonsonate. GUATEMALA: Baja Verapaz, Chimaltenango, Chiquimula, El Progreso, Escuintla, Guatemala, Huehuetenango, Izabal, Jalapa, Jutiapa, Petén, Quiché, Sacatepéquez, San Marcos, Santa Rosa, Suchitepéquez, Zacapa. HONDURAS: Choluteca, Comayagua, Copán, Cortés, El Paraíso, Francisco Morazán, La Paz, Olancho. MEXICO: Aguascalientes, Chiapas, Colima, Durango, Estado de México, Guerrero, Hidalgo, Jalisco, Michoacán, Morelos, Nayarit, Oaxaca, Puebla, Sinaloa, Sonora, Tamaulipas, Veracruz. NICARAGUA: Estelí, Matagalpa.

#### References.

Casey 1915, Arrow 1937b, Blackwelder 1944, Endrődi 1966, 1985a, Ratcliffe and Delgado-Castillo 1990, Ratcliffe and Cave 2006, Krajcik 2005, 2012, Ratcliffe et al. 2013, Deloya et al. 2014a, 2016, Romero-López and Morón 2017.

### Cyclocephala dechambrei

Taxon classificationAnimaliaColeopteraScarabaeidae

Dupuis, 2018

Cyclocephala boliviana Dechambre, 1997: 14, 21–23 [original combination, homonym of C. bolivianaDechambre 1992].
Cyclocephala dechambrei Dupuis [new replacement name by Dupuis 2018: 8].

#### Types.

Holotype ♂ at MNHN (Dechambre 1997, Dupuis 2018).

#### Distribution.

BOLIVIA: Chuquisaca, Cochabamba, La Paz.

#### References.

Dechambre 1997, 1999, Krajcik 2005, 2012, Dupuis 2018.

### Cyclocephala decorella

Taxon classificationAnimaliaColeopteraScarabaeidae

Endrődi, 1966

Cyclocephala decorella Endrődi, 1966: 76, 134, 181–182 [original combination].

#### Types.

Holotype ♂ at ZMHB (Endrődi 1966).

#### Distribution.

BRAZIL: Rio de Janeiro.

#### References.

Pike et al. 1976, Endrődi 1966, 1969b, 1985a, Krajcik 2005, 2012.

### Cyclocephala defecta

Taxon classificationAnimaliaColeopteraScarabaeidae

Endrődi, 1970

Cyclocephala defecta Endrődi, 1970: 105–106 [original combination].

#### Types.

Holotype ♂ at “Pereira Collection in Sao Paulo” (Endrődi 1970). This is possibly referring to MZSP.

#### Distribution.

COLOMBIA: Antioquia.

#### References.

Pike et al. 1976, Endrődi 1970, 1985a, Restrepo et al. 2003, Krajcik 2005, 2012, Gasca-Álvarez and Deloya 2016.

### Cyclocephala deltoides

Taxon classificationAnimaliaColeopteraScarabaeidae

Ratcliffe, 1992

Cyclocephala deltoides Ratcliffe, 1992b: 181–183 [original combination].

#### Types.

Holotype ♂ at UNSM (Ratcliffe 1992b).

#### Distribution.

BRAZIL: Pará.

#### References.

Ratcliffe 1992b, Krajcik 2005, 2012.

### Cyclocephala dichroa

Taxon classificationAnimaliaColeopteraScarabaeidae

Dechambre, 1992

Cyclocephala dichroa Dechambre, 1992: 67–68 [original combination].

#### Types.

Holotype ♂ at MNHN (Dechambre 1992).

#### Distribution.

BRAZIL: Pará. FRENCH GUIANA: Régina, Saül, St.-Laurent du Maroni. PERU: Huánuco. VENEZUELA: Amazonas, Barinas, Bolívar, Portuguesa, Táchira.

#### References.

Dechambre 1992, Joly 2009, Ponchel 2011, Krajcik 2005, 2012.

### Cyclocephala dilatata

Taxon classificationAnimaliaColeopteraScarabaeidae

(Prell, 1934)

Mononidia dilatata Prell, 1934: 162 [original combination].
Cyclocephala dilatata (Prell) [new combination by Arrow 1937b: 8, 10].

#### Types.

Lectotype ♀ at ZMHB (Endrődi 1966).

#### Distribution.

BOLIVIA: Cochabamba, La Paz, Santa Cruz. BRAZIL: Mato Grosso. ECUADOR: Napo. FRENCH GUIANA: Roura. PERU: Huánuco, Pasco, Madre de Dios. SURINAME: Brokopondo. VENEZUELA: Amazonas, Bolívar.

#### References.

Prell 1934, Arrow 1937b, Blackwelder 1944, Pike et al. 1976, Endrődi 1966, 1967b, 1985a, Joly 1995a, Dechambre 1997, 1999, Dupuis 1999, Ponchel 2011, Krajcik 2005, 2012, Ratcliffe et al. 2015, Dupuis 2018.

### Cyclocephala diluta

Taxon classificationAnimaliaColeopteraScarabaeidae

Erichson, 1847

Cyclocephala diluta Erichson, 1847a: 97 [original combination].

#### Types.

Lectotype ♀ at ZMHB (Endrődi 1966).

#### Distribution.

BOLIVIA: Cochabamba, Santa Cruz. BRAZIL: Pará, Santa Catarina. ECUADOR: Cañar, Guayas. FRENCH GUIANA: Saül, St.-Élie. PERU: Ayacucho, Cusco, Junín, Madre de Dios, Pasco.

#### References.

Erichson 1847a, Harold 1869b, Bates 1891, Gruner 1971, Pike et al. 1976, Endrődi 1966, 1985a, Dechambre 1979a, 1999, Dechambre and Duranton 2005, Ponchel 2011, Krajcik 2005, 2012, Breeschoten et al. 2013, Ratcliffe et al. 2015.

### Cyclocephala discicollis

Taxon classificationAnimaliaColeopteraScarabaeidae

Arrow, 1902

Cyclocephala discicollis Arrow, 1902: 140–141 [original combination].
Diapatalia discicollis (Arrow) [new combination by Casey 1915: 111, 129].
Cyclocephala discicollis Arrow [revised combination by Arrow 1937b: 8, 10].

#### Types.

Type at BMNH (Endrődi 1966).

#### Distribution.

COLOMBIA: Casanare. FRENCH GUIANA: Cayenne. PANAMA: Bocas del Toro, Chiriquí, Coclé, Colón, Darien, Former Canal Zone, Panamá, Veraguas. PERU. VENEZUELA.

#### References.

Casey 1915, Arrow 1902, 1937b, Blackwelder 1944, Gruner 1971, Pike et al. 1976, Endrődi 1966, 1985a, Ratcliffe 2003, Núñez-Avellaneda and Neita 2009, Krajcik 2005, 2012, Núñez-Avellaneda 2014.

#### Remarks.

Specimens of *C. discicollis* were reported from Nayarit, Mexico (Endrődi 1966, 1985a). Deloya et al. (2014) reported *C. discicollis* from Guerrero, Jalisco, and Nayarit. Navarrete-Heredia et al. (2001) reported *C. discicollis* from Jalisco. Major faunistic studies did not record any specimens from Mexico (Ratcliffe et al. 2013).

### Cyclocephala discolor

Taxon classificationAnimaliaColeopteraScarabaeidae

(Herbst, 1790)

Melolontha discolor Herbst, 1790: 73 [original combination].
Cyclocephala discolor (Herbst) [new combination by Burmeister 1847: 45–46].
**syn.** Cyclocephala andina Bréthes, 1905: 331–332 [original combination].
Cyclocephala discolor andina Bréthes [new subspecies status by Endrődi 1966: 185].
Cyclocephala discolor (Herbst) [synonymy by Ratcliffe 2003: 118].
**syn.** Cyclocephala aurantiaca Prell, 1937b: 496 [original combination].
Cyclocephala discolor ab. aurantiaca Prell [new infrasubspecific status by Endrődi 1966: 185].
Cyclocephala discolor (Herbst) [synonymy by Ratcliffe 2003: 118].
**syn.** Melolontha unciata Schönherr, 1817: 189 [original combination].
Cyclocephala discolor (Herbst) [synonymy by Burmeister 1847: 46].

#### Types.

Lectotype ♀ of *C. discolor* at ZMHB (Endrődi 1966). Type of *C. aurantiaca* at ZMHB (Endrődi 1966). Endrődi (1966) apparently did not examine the type of *C. andina* but wrote that the type was at “Mus. Buenos Aires”, possibly referring to MACN.

#### Distribution.

ARGENTINA: Salta, Tucumán. BELIZE: Cayo, Stann Creek. BOLIVIA: Beni, Cochabamba, La Paz. BRAZIL: Amazonas, Minas Gerais. COLOMBIA: Amazonas, Antioquia, Cesar, Chocó, Cundinamarca, Meta, Valle del Cauca. COSTA RICA: Alajuela, Cartago, Guanacaste, Heredia, Limón, Puntarenas. ECUADOR: Los Ríos, Morona-Santiago, Napo, Pastaza. FRENCH GUIANA: Cayenne. GUATEMALA: Alta Verapaz, Izabal. GUYANA. HONDURAS: Atlántida, Colón, Comayagua, Cortés, El Paraíso, Francisco Morazán, Gracias a Dios, La Paz, Olancho, Yoro. MEXICO: Chiapas, Colima, Jalisco, Michoacán, Nayarit, Oaxaca, San Luis Potosí. NICARAGUA: Jinotega, RAA Norte, Río San Juan. PANAMA: Bocas del Toro, Chiriquí, Coclé, Colón, Darien, Former Canal Zone, Panamá. PERU: Cusco, Huánuco, Loreto, San Martín. SURINAME. TRINIDAD AND TOBAGO: Trinidad. VENEZUELA: Aragua, Capital District, Monagas.

#### References.

Herbst 1790, Schönherr 1817, Burmeister 1847, Erichson 1848a, Senoner 1864, Harold 1869b, Bréthes 1905, Seidlitz 1905, Arrow 1937b, Prell 1937b, Blackwelder 1944, Martínez 1954, Pike et al. 1976, Endrődi 1963, 1966, 1969b, 1985a, Maes et al. 1997, Ratcliffe and Morón 1997, Ervik et al. 1999, Morón-Ríos and Morón 2001, Navarrete-Heredia et al. 2001, Ratcliffe 2002a, 2003, Restrepo et al. 2003, Ponchel 2006, Ratcliffe and Cave 2006, Pacheco-F. et al. 2008, Núñez-Avellaneda and Rojas-Robles 2008, Núñez-Avellaneda and Neita 2009, Neita-Moreno 2011, Krajcik 2005, 2012, Breeschoten et al. 2013, Ratcliffe et al. 2013, 2015, Deloya et al. 2016, Gasca-Álvarez and Deloya 2016, Romero-López and Morón 2017.

#### Remarks.

Endrődi (1966) reported *C. discolor* from Haiti with no further details. This record is either spurious or erroneous (Ratcliffe and Cave 2015).

### Cyclocephala dispar

Taxon classificationAnimaliaColeopteraScarabaeidae

(Herbst, 1790)

Melolontha dispar Herbst, 1790: 65–66 [original combination].
Cyclocephala dispar (Herbst) [new combination by Reiche 1859: 7].
**syn.** Cyclocephala dorsalis Burmeister, 1847: 64 [original combination].
Cyclocephala dispar (Herbst) [synonymy by Reiche 1859: 7].
**syn.** Cyclocephala stolata Erichson, 1848a: 562].
Cyclocephala dispar (Herbst) [synonymy by Arrow 1911: 171].

#### Types.

Lectotype ♀ of *M. dispar* at ZMHB (Endrődi 1966). Type of *C. dorsalis* at MLUH (Endrődi 1966, Dupuis 2018). Type of *C. stolata* at ZMHB (Endrődi 1966, Dupuis 2018).

#### Distribution.

BRAZIL: Acre, Amazonas, Pará, Roraima. COLOMBIA: Meta. GUYANA: Demerara-Berbice. PARAGUAY. PERU: Loreto.

#### References.

Herbst 1790, Burmeister 1847, Erichson 1848a, Reiche 1859, Harold 1869b, Arrow 1911, 1937b, Blackwelder 1944, Pike et al. 1976, Endrődi 1966, 1985a, Krajcik 2005, 2012, Ratcliffe and Cave 2015, Ratcliffe et al. 2015, Dupuis 2018.

#### Remarks.

Endrődi (1966, 1985a) reported *C. dispar* from Puerto Rico. This record is likely erroneous (Ratcliffe and Cave 2015).

### Cyclocephala distincta

Taxon classificationAnimaliaColeopteraScarabaeidae

Burmeister, 1847

Cyclocephala distincta Burmeister, 1847: 47 [original combination].

#### Types.

Lectotype ♂ at MLUH (Endrődi 1966).

#### Distribution.

BOLIVIA: Beni. BRAZIL: Amazonas, Bahia, Pará, Pernambuco, Rio de Janeiro, Santa Catarina, São Paulo. COLOMBIA. GUYANA.

#### References.

Burmeister 1847, Harold 1869b, Arrow 1937b, Blackwelder 1944, Pike et al. 1976, Dechambre 1980, Endrődi 1966, 1973a, 1985a, Voeks 2002, Restrepo et al. 2003, Marques and Gil-Santana 2009, Krajcik 2005, 2012, Souza et al. 2014a, 2015, Albuquerque et al. 2016, Gasca-Álvarez and Deloya 2016.

### Cyclocephala divaricata

Taxon classificationAnimaliaColeopteraScarabaeidae

Joly, 2005

Cyclocephala divaricata Joly, 2005: 1–5 [original combination].

#### Types.

Holotype ♂ at MIZA (Joly 2005).

#### Distribution.

VENEZUELA: Amazonas.

#### References.

Joly 2005, Krajcik 2012.

### Cyclocephala dolichotarsa

Taxon classificationAnimaliaColeopteraScarabaeidae

Ratcliffe & Cave, 2008

Cyclocephala dolichotarsa Ratcliffe & Cave, 2008: 3–5 [original combination].

#### Types.

Holotype ♂ at FSCA (Ratcliffe and Cave 2008).

#### Distribution.

BAHAMAS: Great Inagua.

#### References.

Krajcik 2012, Ratcliffe and Cave 2008, 2015.

### Cyclocephala dominicana

Taxon classificationAnimaliaColeopteraScarabaeidae

Endrődi, 1985

Cyclocephala dominicana Endrődi, 1985b: 70–71 [original combination].

#### Types.

Holotype ♂ at JPVC (Endrődi 1985b).

#### Distribution.

ECUADOR: Pichincha, Santo Domingo de los Tsáchilas.

#### References.

Endrődi 1985b, Krajcik 2005, 2012.

### Cyclocephala duodecimpunctata

Taxon classificationAnimaliaColeopteraScarabaeidae

Endrődi, 1966

Cyclocephala duodecimpunctata Endrődi, 1966: 82, 127, 189–190 [original combination].

#### Types.

Holotype ♂ at ZMHB (Endrődi 1966).

#### Distribution.

BRAZIL: Espírito Santo, Rio de Janeiro. COLOMBIA.

#### References.

Pike et al. 1976, Endrődi 1966, 1975b, 1985a, Ratcliffe 1992a, Restrepo et al. 2003, Krajcik 2005, 2012, Gasca-Álvarez and Deloya 2016.

### Cyclocephala dupuisi

Taxon classificationAnimaliaColeopteraScarabaeidae

Ratcliffe, 2014

Cyclocephala dupuisi Ratcliffe, 2014: 664–666 [original combination].

#### Types.

Holotype ♂ at UNSM (Ratcliffe 2014).

#### Distribution.

BOLIVIA: Santa Cruz.

#### References.

Ratcliffe 2014.

### Cyclocephala durantonorum

Taxon classificationAnimaliaColeopteraScarabaeidae

Dechambre, 1999

Cyclocephala durantonorum Dechambre, 1999: 8–9 [original combination].

#### Types.

Holotype ♂ at MNHN (Dechambre 1999).

#### Distribution.

FRENCH GUIANA: Régina, Roura, Sinnamary, St.-Élie.

#### References.

Dechambre 1999, Ponchel 2011, Krajcik 2005, 2012.

### Cyclocephala dyscinetoides

Taxon classificationAnimaliaColeopteraScarabaeidae

Dechambre, 1999

Cyclocephala dyscinetoides Dechambre, 1999: 9–10 [original combination].

#### Types.

Holotype ♂ at MNHN (Dechambre 1999).

#### Distribution.

ECUADOR: Santo Domingo de los Tsáchilas.

#### References.

Dechambre 1999, Krajcik 2005, 2012.

### Cyclocephala emarginata

Taxon classificationAnimaliaColeopteraScarabaeidae

Endrődi, 1966

Cyclocephala emarginata Endrődi, 1966: 67, 123, 190–191 [original combination].

#### Types.

Holotype ♂ at ZMHB (Endrődi 1966).

#### Distribution.

BRAZIL: Amazonas. FRENCH GUIANA: St.-Élie.

#### References.

Pike et al. 1976, Dechambre 1979a, Endrődi 1966, 1985a, Gibernau et al. 1999, Krajcik 2005, 2012, Ponchel 2006, 2011, 2015.

### Cyclocephala endrodii

Taxon classificationAnimaliaColeopteraScarabaeidae

Martínez, 1965

Cyclocephala (Paraclinidia) endrodii Martínez, 1965b: 14–18 [original combination].

#### Types.

Holotype ♂ at MACN (Antonio Martínez Collection) (Martínez 1965b, Endrődi 1966).

#### Distribution.

BRAZIL: Pará, Rondônia.

#### References.

Martínez 1965b, Pike et al. 1976, Endrődi 1966, 1985a, Dechambre 1992, Krajcik 2005, 2012.

### Cyclocephala endroedyyoungai

Taxon classificationAnimaliaColeopteraScarabaeidae

Endrődi, 1964

Cyclocephala endroedyyoungai Endrődi, 1964: 435–436 [original combination].

#### Types.

Holotype ♂ at HNHM (Endrődi Collection) (Endrődi 1964).

#### Distribution.

BRAZIL: Espírito Santo.

#### References.

Pike et al. 1976, Endrődi 1964, 1966, 1985a, Krajcik 2005, 2012.

### Cyclocephala englemani

Taxon classificationAnimaliaColeopteraScarabaeidae

(Ratcliffe, 1977)

Mimeoma englemani Ratcliffe, 1977: 430–432 [original combination].
Cyclocephala englemani (Ratcliffe) [new combination by Moore et al. 2015: 898].

#### Types.

Holotype ♂ at UNSM (Ratcliffe 1977).

#### Distribution.

PANAMA: Darien, Former Canal Zone, Panamá.

#### References.

Ratcliffe 1977, 2002a, 2003, Endrődi 1985a, Krajcik 2005, 2012, Moore et al. 2015.

### Cyclocephala enigma

Taxon classificationAnimaliaColeopteraScarabaeidae

Ratcliffe, 2003

Cyclocephala enigma Ratcliffe, 2003: 60, 70, 121–123 [original combination].

#### Types.

Holotype ♂ at MNCR (originally deposited at INBio) (Ratcliffe 2003).

#### Distribution.

COSTA RICA: Guanacaste.

#### References.

Ratcliffe 2003, Krajcik 2005, 2012.

### Cyclocephala epistomalis

Taxon classificationAnimaliaColeopteraScarabaeidae

Bates, 1888

Cyclocephala epistomalis Bates, 1888: 303–304 [original combination].
Homochromina epistomalis (Bates) [new combination by Casey 1915: 165].
Cyclocephala epistomalis Bates [revised combination by Arrow 1937b: 8, 10].
**syn.** Cyclocephala mollis Endrődi, 1963: 323–325 [original combination].
Cyclocephala epistomalis ab. mollis Endrődi [new infrasubspecific status by Endrődi 1985a: 90].
Cyclocephala epistomalis Bates [synonymy by Ratcliffe 2003: 123].

#### Types.

Type of *C. epistomalis* at BMNH (Endrődi 1966). Holotype ♂ of *C. mollis* at ZSMC (1966).

#### Distribution.

BOLIVIA: Beni, Santa Cruz. BRAZIL: Amazonas, Mato Grosso, Mato Grosso do Sul, Pará. COLOMBIA: Amazonas, Caquetá, Cundinamarca, Meta, Risaralda, Valle del Cauca. FRENCH GUIANA: Cayenne. GUATEMALA: Sacatepéquez. PANAMA: Coclé. PARAGUAY. VENEZUELA: Apure.

#### References.

Bates 1888, Casey 1915, Arrow 1937b, Blackwelder 1944, Gruner 1971, Pike et al. 1976, Prance 1980, Endrődi 1963, 1964, 1966, 1985a, Ratcliffe 2003, Pardo-Locarno et al. 2011, Krajcik 2005, 2012, Ratcliffe et al. 2013, Maia et al. 2014, López-Garcia et al. 2015, Gasca-Álvarez and Deloya 2016, Santos Fava and Gomes 2017.

### Cyclocephala ergastuli

Taxon classificationAnimaliaColeopteraScarabaeidae

Dechambre, 1997

Cyclocephala ergastuli Dechambre, 1997: 14, 16–17 [original combination].

#### Types.

Holotype ♂ at MNHN (Dechambre 1997).

#### Distribution.

COLOMBIA: Valle del Cauca. FRENCH GUIANA: Iracoubo, Kourou, Régina, Roura, St.-Laurent du Maroni. TRINIDAD AND TOBAGO: Trinidad. VENEZUELA: Bolívar.

#### References.

Dechambre 1997, Restrepo et al. 2003, Krajcik 2005, 2012, Ponchel 2011, 2015, Gasca-Álvarez and Deloya 2016.

### Cyclocephala erotylina

Taxon classificationAnimaliaColeopteraScarabaeidae

Arrow, 1914

Cyclocephala erotylina Arrow, 1914: 275 [original combination].

#### Types.

Type at BMNH (Endrődi 1966).

#### Distribution.

COSTA RICA: Alajuela, Guanacaste, Limón, Puntarenas, San José. GUATEMALA: Escuintla, Guatemala, Jutiapa, Quetzaltenango, San Marcos, Santa Rosa, Sololá, Suchitepéquez. HONDURAS: Olancho, Yoro. MEXICO: Chiapas, Colima. NICARAGUA: Jinotega, Matagalpa, RAA Norte. PANAMA: Coclé, Panamá.

#### References.

Arrow 1914, 1937b, Blackwelder 1944, Pike et al. 1976, Endrődi 1966, 1980, 1985a, Maes et al. 1997, Ratcliffe and Morón 1997, Ratcliffe 2002a, 2003, Ratcliffe and Cave 2006, Pacheco-F. et al. 2008, Krajcik 2005, 2012, García-López et al. 2013, Ratcliffe et al. 2013, Romero-López and Morón 2017.

### Cyclocephala everardoi

Taxon classificationAnimaliaColeopteraScarabaeidae

Grossi, Santos, & Almeida, 2016

Cyclocephala everardoi Grossi, Santos, & Almeida, 2016: 249–250 [original combination].

#### Types.

Holotype ♂ at CERPE (Grossi et al. 2016).

#### Distribution.

BRAZIL: Minas Gerais.

#### References.

Grossi et al. 2016.

### Cyclocephala fankhaeneli

Taxon classificationAnimaliaColeopteraScarabaeidae

Endrődi, 1964

Cyclocephala fankhaeneli Endrődi 1964: 461–462 [original combination].

#### Types.

Holotype ♂ at HNHM (Endrődi Collection) (Endrődi 1964).

#### Distribution.

BOLIVIA: Tarija. BRAZIL: Paraná, Rio Grande do Sul.

#### References.

Pike et al. 1976, Endrődi 1964, 1966, 1985a, Grossi et al. 2011, Krajcik 2005, 2012, Breeschoten et al. 2013.

### Cyclocephala fasciolata

Taxon classificationAnimaliaColeopteraScarabaeidae

Bates, 1888

Cyclocephala fasciolata Bates, 1888: 301 [original combination].
Halotosia fasciolata (Bates) [new combination by Casey 1915: 113].
Cyclocephala fasciolata Bates [revised combination by Arrow 1937b: 8, 10].

#### Types.

Type at BMNH (Endrődi 1966).

#### Distribution.

COLOMBIA: Antioquia, Chocó. COSTA RICA: Guanacaste, Puntarenas. GUATEMALA: Alta Verapaz, Chiquimula, Izabal. MEXICO: Chiapas, Jalisco, Puebla, Veracruz. PANAMA: Chiriquí.

#### References.

Bates 1888, Casey 1915, Arrow 1937b, Blackwelder 1944, Pike et al. 1976, Endrődi 1966, 1985a, Búrquez et al. 1987, Lobo and Morón 1993, Ratcliffe and Morón 1997, Carrillo-Ruiz and Morón 2003, Restrepo et al. 2003, Ratcliffe 2002a, 2003, Ramos-Elorduy and Pino Moreno 2002, 2004 Malý 2006, Múñoz-Hernández et al. 2008, Pacheco-F. et al. 2008, Neita-Moreno 2011, Aguirre et al. 2011, Krajcik 2005, 2012, Breeschoten et al. 2013, Ratcliffe et al. 2013, García-López et al. 2013, Morón et al. 2014, Dossey et al. 2016, Gasca-Álvarez and Deloya 2016, Mitasuhashi 2016.

### Cyclocephala figurata

Taxon classificationAnimaliaColeopteraScarabaeidae

Burmeister, 1847

Cyclocephala figurata Burmeister, 1847: 65 [original combination].

#### Types.

Lectotype ♂ at MNHN (Dechambre 1991b).

#### Distribution.

FRENCH GUIANA: Cayenne.

#### References.

Burmeister 1847, Harold 1869b, Arrow 1937b, Blackwelder 1944, Pike et al. 1976, Endrődi 1966, 1985a, Dechambre 1991b, Ponchel 2011, Krajcik 2005, 2012.

### Cyclocephala flavipennis

Taxon classificationAnimaliaColeopteraScarabaeidae

Arrow, 1914

Cyclocephala flavipennis Arrow, 1914: 275 [original combination].

#### Types.

Type at BMNH (Endrődi 1966).

#### Distribution.

BOLIVIA. BRAZIL: Rio de Janeiro, Rio Grande do Sul. ECUADOR: Bolívar.

#### References.

Arrow 1914, 1937b, Blackwelder 1944, Pike et al. 1976, Endrődi 1966, 1967b, 1985a, Dechambre 1999, Bolliger et al. 2006, Krajcik 2005, 2012, Cherman et al. 2013, Diez-Rodríquez et al. 2015.

### Cyclocephala flavoscutellaris

Taxon classificationAnimaliaColeopteraScarabaeidae

Höhne, 1923

Cyclocephala flavoscutellaris Höhne, 1923b: 357–358 [original combination].

#### Types.

Lectotype ♂ at ZMHB (Endrődi 1966).

#### Distribution.

BRAZIL: Amazonas. COLOMBIA. ECUADOR: Morona-Santiago, Pichincha. PERU: Cusco.

#### References.

Höhne 1923b, Arrow 1937b, Blackwelder 1944, Pike et al. 1976, Endrődi 1966, 1985a, Krajcik 2005, 2012, Ratcliffe et al. 2015, Gasca-Álvarez and Deloya 2016.

### Cyclocephala flora

Taxon classificationAnimaliaColeopteraScarabaeidae

Arrow, 1911

Cyclocephala flora Arrow, 1911: 175 [original combination].

#### Types.

Type at BMNH (Endrődi 1966).

#### Distribution.

BRAZIL: Amazonas. PERU: Loreto.

#### References.

Arrow 1911, 1937b, Blackwelder 1944, Pike et al. 1976, Martínez 1978b, Endrődi 1964, 1966, 1985a, Krajcik 2005, 2012, Ratcliffe et al. 2015.

### Cyclocephala forcipulata

Taxon classificationAnimaliaColeopteraScarabaeidae

Howden & Endrődi, 1966

Cyclocephala forcipulata Howden & Endrődi, 1966: 299–301 [original combination].

#### Types.

Holotype ♂ at CNC (Howden and Endrődi 1966).

#### Distribution.

MEXICO: Durango, Guerrero, Jalisco, Nayarit, Oaxaca, Sinaloa.

#### References.

Howden and Endrődi 1966, Martínez 1975b, Pike et al. 1976, Endrődi 1985a, Ratcliffe and Delgado-Castillo 1990, Ratcliffe and Morón 1997, Joly 2000a, Navarrete-Heredia et al. 2001, Smith 2003, 2009, Krajcik 2005, 2012, Ratcliffe et al. 2013.

### Cyclocephala forsteri forsteri

Taxon classificationAnimaliaColeopteraScarabaeidae

Endrődi, 1963

Cyclocephala forsteri forsteri Endrődi, 1963: 325–326 [original combination].

#### Types.

Holotype ♂ at ZSMC (Endrődi 1966).

#### Distribution.

BOLIVIA: La Paz, Santa Cruz. BRAZIL: Amazonas, Distrito Federal, Goiás, Mato Grosso, Mato Grosso do Sul, Rio de Janeiro, Santa Catarina. COLOMBIA: Casanare, Meta. PARAGUAY: Concepción, Distrito Capital.

#### References.

Pike et al. 1976, Endrődi 1963, 1964, 1966, 1985a, Scariot et al. 1991, Hardy 1991, Poole and Gentili 1996, Santos and Ávila 2007, Núñez-Avellaneda and Neita-Moreno 2009, Coutinho et al. 2011, Oliveira and Ávila 2011, Rodrigues et al. 2011, Krajcik 2005, 2012, Breeschoten et al. 2013, Gasca-Álvarez and Deloya 2016.

### Cyclocephala forsteri maracayensis

Taxon classificationAnimaliaColeopteraScarabaeidae

Endrődi, 1963

Cyclocephala forsteri maracayensis Endrődi, 1963: 326 [original combination].

#### Types.

Holotype ♂ at ZSMC (Endrődi 1966).

#### Distribution.

VENEZUELA: Aragua, Carabobo.

#### References.

Pike et al. 1976, Endrődi 1963, 1966, 1985a, Krajcik 2005, 2012.

### Cyclocephala freudei

Taxon classificationAnimaliaColeopteraScarabaeidae

Endrődi, 1963

Cyclocephala freudei Endrődi, 1963: 328–329 [original combination].

#### Types.

Holotype ♂ at ZSMC (Endrődi 1966).

#### Distribution.

EL SALVADOR: Cuscatlán, San Salvador. GUATEMALA: Totonicapán, Zacapa. MEXICO: Colima, Durango, Guerrero, Jalisco, Michoacán, Nayarit, Oaxaca, Puebla, Querétaro, Sinaloa, Sonora, Veracruz.

#### References.

Pike et al. 1976, Endrődi 1963, 1966, 1985a, Hardy 1991, Thomas 1993, Poole and Gentili 1996, Morón et al. 1988, 1996, 1998, Ratcliffe and Morón 1997, Navarrete-Heredia et al. 2001, Riley and Wolfe 2003, Ratcliffe and Cave 2006, Smith 2003, 2009, Krajcik 2005, 2012, Ratcliffe et al. 2013, Deloya et al. 2014a, 2016.

#### Remarks.

*Cyclocephala freudei* was reported from the United States (Texas), Costa Rica (Santa Elena), and Ecuador (San José de Canelos) (Endrődi 1966, 1985a). This species does not occur in the United States and the Costa Rican record is based only on one specimen (Ratcliffe 2003, Ratcliffe and Cave 2006, Ratcliffe et al. 2013). The Ecuadorian record has not been evaluated with further sampling (Ratcliffe and Cave 2006).

### Cyclocephala freyi freyi

Taxon classificationAnimaliaColeopteraScarabaeidae

Endrődi, 1964

Cyclocephala freyi Endrődi, 1964: 464–466 [original combination].

#### Types.

Holotype ♂ at NHMB (Frey Collection) (Endrődi 1964).

#### Distribution.

BOLIVIA: Santa Cruz. PERU: Cusco, Junín, Madre de Dios.

#### References.

Pike et al. 1976, Dechambre 1979a, Endrődi 1964, 1966, 1985a, Krajcik 2005, 2012, Ratcliffe et al. 2015.

### Cyclocephala freyi integra

Taxon classificationAnimaliaColeopteraScarabaeidae

Dechambre, 1999

Cyclocephala freyi integra Dechambre, 1999: 22 [original combination].

#### Types.

Holotype ♂ at MNHN (Dechambre 1999).

#### Distribution.

FRENCH GUIANA: Saül, Sinnamary.

#### References.

Dechambre 1999, Ponchel 2011, Krajcik 2005, 2012.

### Cyclocephala frontalis

Taxon classificationAnimaliaColeopteraScarabaeidae

Chevrolat, 1844

Cyclocephala frontalis Chevrolat, 1844: 90–91 [original combination].
Cyclocephala cubana Chapin [synonymy by Endrődi 1966: 179].
Cyclocephala frontalis Chevrolat [revalidated species status by Ratcliffe and Cave 2015: 87].
**syn.** Cyclocephala cubana Chapin, 1932: 291–292 [original combination].
Cyclocephala frontalis Chevrolat [synonymy by Ratcliffe and Cave 2015: 87].

#### Types.

Neotype ♂ of *C. frontalis* at UNSM (Ratcliffe and Cave 2015). Type of *C. cubana* at USNM (Endrődi 1966).

#### Distribution.

BAHAMAS: Eleuthera. CUBA: Artemisa, Camagüey, Ciego de Ávila, Cienfuegos, Granma, La Habana, Las Tunas, Matanzas, Pinar del Río, Sancti Spíritus, Santiago de Cuba, Villa Clara. DOMINICAN REPUBLIC: Azua, Barahona, Elías Piña, Monte Cristi, Pedernales, San Juan, Valverde. HAITI: Grand Anse, Ouest, Sud. PUERTO RICO: Cabo Rojo, Guánica, Lajas.

#### References.

Latreille 1829, 1837, Marschall 1857, Chevrolat 1844, 1865, Burmeister 1847, Harold 1869b, Gundlach 1891, Leng and Mutchler 1914, Chapin 1932, Arrow 1937b, Blackwelder 1944, Bruner et al. 1975, Pike et al. 1976, Endrődi 1966, 1969b, 1985a, González et al. 1998, Fernández García 2006, Krajcik 2005, 2012, Ratcliffe and Cave 2015.

### Cyclocephala fulgurata

Taxon classificationAnimaliaColeopteraScarabaeidae

Burmeister, 1847

Cyclocephala fulgurata Burmeister, 1847: 63 [original combination].
Ochrosidia (Graphalia) fulgurata (Burmeister) [new combination and new subgeneric classification by Casey 1915: 159].
Cyclocephala fulgurata Burmeister [revised combination and removal of subgeneric classification by Arrow 1937b: 8, 11].

#### Types.

Lectotype at MLUH (Endrődi 1966).

#### Distribution.

ARGENTINA: Buenos Aires. BELIZE: Stann Creek. BOLIVIA: Beni, Cochabamba, Santa Cruz. BRAZIL: Pará. COLOMBIA: Antioquia, Boyacá, Cauca, Chocó, Cundinamarca, Risaralda, Santander, Tolima, Valle del Cauca. COSTA RICA: Alajuela, Cartago, Guanacaste, Limón, Puntarenas. ECUADOR: Bolívar, Cañar, Los Ríos. FRENCH GUIANA: Cayenne. GUATEMALA: Alta Verapaz, Baja Verapaz, Chimaltenango, Guatemala, Huehuetenango, Izabal, Petén, San Marcos, Suchitepéquez, Zacapa. HONDURAS: Atlántida, Cortés, El Paraíso, Olancho, Yoro. MEXICO: Chiapas, Distrito Federal, Hidalgo, Jalisco, Nayarit, Oaxaca, Tamaulipas, Veracruz, Zacatecas. PANAMA: Bocas del Toro, Chiriquí, Coclé, Darien, Former Canal Zone, Panamá, Veraguas. PERU: Cajamarca, Junín, Madre de Dios. VENEZUELA: Capital District, Carabobo, Mérida, Monagas.

#### References.

Burmeister 1847, Harold 1869b, Bates 1888, Casey 1915, Arrow 1937b, Blackwelder 1944, Figueroa-P. 1952, Pike et al. 1976, Dechambre 1979a, Endrődi 1964, 1966, 1969b, 1985a, Dechambre 1979a, Thomas 1993, Ratcliffe and Morón 1997, Caicedo and Bellotti 2002, Ratcliffe 2002a, 2003, Restrepo et al. 2003, Villegas-Urbano 2004, Patiño 2004, Vásquez and Sánchez 2004, Pardo-Locarno et al. 1995, 2003, 2005a, Ratcliffe and Cave 2006, Neita-Moreno et al. 2006, Bran et al. 2006, Pacheco-F. et al. 2008, Villegas-Urbano et al. 2008, Neita-Moreno 2011, Krajcik 2005, 2012, Breeschoten et al. 2013, Pardo-Locarno 2013, Yepes-Rodriguez et al. 2013, López-García et al. 2015, Ratcliffe et al. 2013, 2015, Gasca-Álvarez and Deloya 2016, Villalobos-Moreno et al. 2016, 2017.

### Cyclocephala fulvipennis

Taxon classificationAnimaliaColeopteraScarabaeidae

Burmeister, 1847

Cyclocephala fulvipennis Burmeister, 1847: 71 [original combination].

#### Types.

Lectotype ♀ at MNHN (Dechambre 1991b). Invalid neotype ♂ at HNHM (Endrődi Collection) (Endrődi 1966).

#### Distribution.

BOLIVIA: Beni, La Paz. BRAZIL: Bahia, Rio de Janeiro, São Paulo. PERU.

#### References.

Burmeister 1847, Harold 1869b, Arrow 1937b, Blackwelder 1944, Pike et al. 1976, Endrődi 1966, 1973a, 1985a, Dechambre 1979b, 1991b, 1992, Ratcliffe 1992a, Malý 2006, Dupuis 2008, 2014, Krajcik 2005, 2012, Ratcliffe et al. 2015.

#### Remarks.

*Cyclocephala fulvipennis* was reported from Honduras and Nicaragua (Endrődi 1966, 1985a). These records are likely based on misidentified specimens of *C. porioni* Dechambre (Ratcliffe 2003).

### Cyclocephala gabaldoni

Taxon classificationAnimaliaColeopteraScarabaeidae

Martínez & Martínez, 1981

Cyclocephala gabaldoni Martínez & Martínez, 1981: 203–206 [original combination].

#### Types.

Holotype ♂ at MACN (Antonio Martínez Collection) (Martínez and Martínez 1981).

#### Distribution.

FRENCH GUIANA. VENEZUELA: Amazonas.

#### References.

Martínez and Martínez 1981, Endrődi 1985a, Ponchel 2011, Krajcik 2005, 2012.

### Cyclocephala genieri

Taxon classificationAnimaliaColeopteraScarabaeidae

Joly, 2010

Cyclocephala genieri Joly, 2010: 141–146 [original combination].

#### Types.

Holotype ♂ at USNM (Joly 2010).

#### Distribution.

PERU: Ucayali.

#### References.

Joly 2010, Krajcik 2012, Ratcliffe et al. 2015.

### Cyclocephala gigantea

Taxon classificationAnimaliaColeopteraScarabaeidae

Dupuis, 1999

Cyclocephala gigantea Dupuis, 1999: 186 [original combination].

#### Types.

Holotype ♀ at MNHN (Dupuis 1999).

#### Distribution.

ECUADOR: Pastaza, Sucumbíos.

#### References.

Dupuis 1999, Malý 2006, Krajcik 2005, 2012.

### Cyclocephala goetzi

Taxon classificationAnimaliaColeopteraScarabaeidae

Endrődi, 1966

Cyclocephala goetzi Endrődi, 1966: 208–209 [original combination].

#### Types.

Holotype ♂ at NHMB (Frey Collection) (Endrődi 1966).

#### Distribution.

BOLIVIA: Beni. PERU: Madre de Dios.

#### References.

Pike et al. 1976, Endrődi 1966, 1985a, Ratcliffe 1992a, Dechambre and Duranton 2005, Krajcik 2005, 2012, Ratcliffe et al. 2015.

### Cyclocephala gravis

Taxon classificationAnimaliaColeopteraScarabaeidae

Bates, 1888

Cyclocephala gravis Bates, 1888: 308 [original combination].
**syn.** Cyclocephala meinanderi Endrődi, 1964: 457–459 [original combination].
Cyclocephala gravis ab. meinanderi Endrődi [new infrasubspecific status by Endrődi 1967: 90].
Cyclocephala gravis Bates [synonymy by Maes 1994: 11].

#### Types.

Type of *C. gravis* at BMNH (Endrődi 1966). Holotype ♂ of *C. meinanderi* at HNHM (Endrődi Collection) (Endrődi 1964).

#### Distribution.

BELIZE: Cayo, Orange Walk, Stann Creek, Toledo. BOLIVIA: Cochabamba, Santa Cruz. BRAZIL: Bahia, Espírito Santo, Mato Grosso, Minas Gerais, Pernambuco, Rio de Janeiro. COLOMBIA: Antioquia, Boyacá, Caquetá, Chocó, Cundinamarca, Magdalena, Santander, Tolima. COSTA RICA: Alajuela, Cartago, Guanacaste, Heredia, Limón, Puntarenas, San José. ECUADOR: Santo Domingo de los Tsáchilas. EL SALVADOR: Morazán. FRENCH GUIANA: Cayenne, St.-Élie, St.-Laurent du Maroni. GUATEMALA: Alta Verapaz, Huehuetenango, Izabal, Petén, Sololá, Zacapa. GUYANA: Upper Demerara-Berbice. HONDURAS: Atlántida, Choluteca, Cortés, El Paraíso, Francisco Morazán, Gracias a Dios, Olancho, Santa Bárbara, Yoro. MEXICO: Campeche, Chiapas, Guanajuato, Oaxaca, Quintana Roo, San Luis Potosí, Tabasco, Veracruz, Yucatán. NICARAGUA: Chontales, Jinotega, Matagalpa, RAA Norte, Río San Juan. PANAMA: Bocas del Toro, Chiriquí, Colón, Former Canal Zone, Panamá. PARAGUAY: Distrito Capital. SURINAME. VENEZUELA: Capital District, Guárico.

#### References.

Bates 1888, Bodkin 1919, Arrow 1937b, Blackwelder 1944, Pike et al. 1976, Dechambre 1979a, 1980, Endrődi 1964, 1966, 1967, 1985a, Young 1986, 1988a, b, 1990, Thomas 1993, Lobo and Morón 1993, Maes 1987, 1994, Ramírez and Brito 1992, Croat 1997, Ratcliffe and Morón 1997, Beath 1998, 1999, Ratcliffe 1992b, 2002a, 2003, Restrepo et al. 2003, Reyes Novelo and Morón 2005, Ratcliffe and Cave 2006, Krajcik 2005, 2012, Neita-Moreno 2011, Ponchel 2011, García-López et al. 2013, Moore and Jameson 2013, Ratcliffe et al. 2013, López-García et al. 2015, Gasca-Álvarez and Deloya 2016.

### Cyclocephala gregaria

Taxon classificationAnimaliaColeopteraScarabaeidae

Heyne & Taschenberg, 1907

Cyclocephala gregaria Heyne & Taschenberg, 1907: 91–92 [original combination]
Stigmalia gregaria (Heyne & Taschenberg) [new combination by Casey 1915: 115, 122].
Cyclocephala gregaria Heyne & Taschenberg [revised combination by Arrow 1937b: 8, 11].
**syn.** Cyclocephala gregaria pallida Arrow, 1911: 172 [original combination].
Cyclocephala gregaria ab. pallida Arrow [new infrasubspecific status by Endrődi 1966: 210].

#### Types.

Invalid neotype ♂ of *C. gregaria* at HNHM (Endrődi Collection) (Endrődi 1966). Endrődi (1966) did not examine the type of *C. gregaria pallida*, and it may be at BMNH.

#### Distribution.

BOLIVIA: Santa Cruz. BRAZIL: Pará, São Paulo. COLOMBIA: Antioquia, Boyacá, Caldas, Cauca, Cundinamarca, Magdalena, Quindío, Risaralda, Santander, Tolima, Valle del Cauca. ECUADOR: Morona-Santiago. VENEZUELA: Mérida.

#### References.

Heyne and Taschenberg 1907, Casey 1915, Arrow 1911, 1937b, Blackwelder 1944, Daniel 1945, Martínez 1968c, Pike et al. 1976, Endrődi 1966, 1985a, Montoya et al. 1994, Dechambre 1995, Restrepo et al. 2003, García-Robledo et al. 2004, 2005, Útima and Vallejo 2008, García-Robledo 2010, Krajcik 2005, 2012, Breeschoten et al. 2013, Yepes-Rodriguez et al. 2013, López-García et al. 2015, Gasca-Álvarez and Deloya 2016.

#### Remarks.

Heyne and Taschenberg (1907) briefly described an *in litt*. specimen of *C. gregaria* from Steinheil’s collection. Arrow (1911) published a longer description and designated types for *C. gregaria* that were sent to him from insect specimen dealers Otto Staudinger and Andreas Bang-Haas. *Cyclocephala gregaria* Arrow could be considered a homonym and synonym of *C. gregaria* Heyne & Taschenberg. Endrődi (1966) could not locate the type of *C. gregaria* and designated an invalid neotype. Endrődi (1966, 1985a) reported *C. gregaria* Heyne & Taschenberg from Boquete (Chiriquí, Panama). Ratcliffe (2003) considers this record as likely erroneous and probably based on misidentified specimens of *C. conspicua* Sharp. Arrow (1911) described C. gregaria var. pallida. However, Arrow (1911) did not discuss or name subspecies, meaning that C. gregaria var. pallida is ambiguously infrasubspecific and should be treated as a subspecies at original description.

### Cyclocephala guaguarum

Taxon classificationAnimaliaColeopteraScarabaeidae

Dechambre & Endrődi, 1984

Cyclocephala guaguarum Dechambre & Endrődi, 1984: 169 [original combination].

#### Types.

Holotype ♂ at MNHN (Dechambre and Endrődi 1984).

#### Distribution.

COLOMBIA: Valle del Cauca. ECUADOR: Cotopaxi, Pichincha.

#### References.

Dechambre and Endrődi 1984, Restrepo et al. 2003, Krajcik 2005, 2012, Gasca-Álvarez and Deloya 2016.

### Cyclocephala guianae

Taxon classificationAnimaliaColeopteraScarabaeidae

Endrődi, 1969

Cyclocephala guianae Endrődi, 1969b: 33–34 [original combination].

#### Types.

Holotype ♂ at USNM (Endrődi 1969b).

#### Distribution.

BRAZIL: Amazonas. COLOMBIA: Casanare, Meta. FRENCH GUIANA: Kourou, St.-Élie, St.-Laurent du Maroni. SURINAME: Brokopondo.

#### References.

Pike et al. 1976, Dechambre 1979a, Endrődi 1969b, 1985a, Ratcliffe 1992b, Küchmeister et al. 1998, Andreazze 2001, Silberbauer-Gottsberger et al. 2001, Andreazze and da Silva Motta 2002, Krajcik 2005, 2012, Gasca-Álvarez et al. 2014, Núñez-Avellaneda 2014, Ponchel 2011, 2015, Gasca-Álvarez and Deloya 2016.

### Cyclocephala guttata

Taxon classificationAnimaliaColeopteraScarabaeidae

Bates, 1888

Cyclocephala guttata Bates, 1888: 306 [original combination].
Dichromina guttata (Bates) [new combination by Casey 1915: 160].
Cyclocephala guttata Bates [revised combination by Arrow 1937b: 8, 11].

#### Types.

Type at BMNH (Endrődi 1966).

#### Distribution.

GUATEMALA: El Progreso, Escuintla, Izabal, Retalhuleu, Suchitepéquez, Zacapa. HONDURAS: Atlántida, Choluteca, Yoro. MEXICO: Chiapas, Morelos, Oaxaca, Puebla, San Luis Potosí, Tabasco, Veracruz. NICARAGUA: Granada, Masaya, Río San Juan, Rivas.

#### References.

Bates 1888, Casey 1915, Arrow 1937b, Blackwelder 1944, Pike et al. 1976, Endrődi 1966, 1985a, Morón 1979, Morón et al. 1985, Thomas 1993, Lobo and Morón 1993, Maes and Ratcliffe 1996, Camino-Lavín et al. 1996, Sanchez Soto 1997, Ratcliffe and Morón 1997, Ramos-Elorduy and Pino Moreno 2004, Ratcliffe and Cave 2006, Pacheco-F. et al. 2008, Krajcik 2005, 2012, Ratcliffe et al. 2013, Dossey et al. 2016.

### Cyclocephala guycolasi

Taxon classificationAnimaliaColeopteraScarabaeidae

Dechambre, 1992

Cyclocephala guycolasi Dechambre, 1992: 62 [original combination].

#### Types.

Holotype ♂ at MNHN (Dechambre 1992).

#### Distribution.

PERU: Junín.

#### References.

Dechambre 1992, Krajcik 2005, 2012, Ratcliffe et al. 2015.

### Cyclocephala halffteriana

Taxon classificationAnimaliaColeopteraScarabaeidae

Martínez, 1968

Cyclocephala halffteriana Martínez, 1968a: 188–190 [original combination].

#### Types.

Holotype ♂ at MACN (Antonio Martínez Collection) (Martínez 1968a).

#### Distribution.

MEXICO: Baja California Sur, Sonora.

#### References.

Martínez 1968a, Pike et al. 1976, Endrődi 1985a, Ratcliffe and Morón 1997, Krajcik 2005, 2012, Ratcliffe et al. 2013.

### Cyclocephala hardyi

Taxon classificationAnimaliaColeopteraScarabaeidae

Endrődi, 1975

Cyclocephala hardyi Endrődi, 1975c: 281–284 [original combination].

#### Types.

Holotype ♂ at INPA (Endrődi 1975c).

#### Distribution.

BRAZIL: Amazonas. GUAYANA: Upper Takutu-Upper Essequibo.

#### References.

Endrődi 1975c, Prance and Arias 1975, Pike et al. 1976, Faegri and van der Pijl 1979, Endrődi 1975c, 1985a, Andreazze and Fonseca 1998, Joly 2000b, Andreazze 2001, Seymour and Matthews 2006, Davis et al. 2008, Soderstrom 2008, Thien et al. 2009, Krajcik 2005, 2012, Gottsberger 2016.

### Cyclocephala hartmannorum

Taxon classificationAnimaliaColeopteraScarabaeidae

Malý, 2006

Cyclocephala hartmannorum Malý, 2006: 2–5 [original combination].

#### Types.

Holotype ♂ at NMPC (Malý 2006).

#### Distribution.

COSTA RICA: Cartago, Guanacaste, Heredia, San José. PANAMA: Chiriquí, Colón, Panamá.

#### References.

Malý 2006, Krajcik 2012, Dupuis 2008, 2014.

### Cyclocephala helavai

Taxon classificationAnimaliaColeopteraScarabaeidae

Endrődi, 1975

Cyclocephala helavai Endrődi, 1975b: 258–260 [original combination].

#### Types.

Holotype ♂ at CMNC (Henry and Anne Howden Collection) (Endrődi 1975b).

#### Distribution.

COLOMBIA: Antioquia.

#### References.

Pike et al. 1976, Endrődi 1975b, 1985a, Restrepo et al. 2003, Gasca-Álvarez and Deloya 2016.

### Cyclocephala herteli

Taxon classificationAnimaliaColeopteraScarabaeidae

Endrődi, 1964

Cyclocephala herteli Endrődi, 1964: 447–449 [original combination].
**syn.** Cyclocephala barroensis Endrődi, 1979: 216 [original combination].
Cyclocephala herteli Endrődi, 1964 [synonymy by Ratcliffe 2002a: 29].

#### Types.

Holotype ♂ of *C. herteli* at USNM (Endrődi 1964). Holotype ♂ of *C. barroensis* at USNM (Endrődi 1979).

#### Distribution.

PANAMA: Coclé, Colón, Former Canal Zone, Panamá.

#### References.

Pike et al. 1976, Endrődi 1964, 1966, 1979, 1985a, Ratcliffe 1992a, 2002a, 2003, Krajcik 2005, 2012.

#### Remarks.

*Cyclocephala barroensis* is incorrectly listed as a synonym of *C. helavai* Endrődi in Krajcik (2005).

### Cyclocephala hiekei

Taxon classificationAnimaliaColeopteraScarabaeidae

Endrődi, 1964

Cyclocephala hiekei Endrődi, 1964: 454–456 [original combination].

#### Types.

Holotype ♂ at ZMHB (Endrődi 1964).

#### Distribution.

COLOMBIA: Cauca, Chocó.

#### References.

Pike et al. 1976, Endrődi 1964, 1966, 1985a, Restrepo et al. 2003, Neita-Moreno 2011, Krajcik 2005, 2012, Gasca-Álvarez and Deloya 2016.

### Cyclocephala hirsuta

Taxon classificationAnimaliaColeopteraScarabaeidae

Höhne, 1923

Cyclocephala hirsuta Höhne, 1923b: 358–359 [original combination].

#### Types.

Lectotype at ZMHB (Endrődi 1966).

#### Distribution.

BOLIVIA: La Paz. BRAZIL: São Paulo. ECUADOR: Orellana. PERU: San Martín.

#### References.

Höhne 1923b, Arrow 1937b, Blackwelder 1944, Pike et al. 1976, Endrődi 1966, 1985a, Joly 2000a, Krajcik 2005, 2012, Ratcliffe et al. 2015.

### Cyclocephala hirta hirta

Taxon classificationAnimaliaColeopteraScarabaeidae

LeConte, 1861

Cyclocephala hirta LeConte, 1861: 346 [original combination].
Spilosota hirta (LeConte) [new combination by Casey 1915: 132].
Cyclocephala (Spilosota) hirta LeConte [new subgeneric classification by Saylor 1937: 68].
Cyclocephala hirta LeConte [removal of subgeneric classification by Arrow 1937b: 8, 11].
**syn.** Spilosota inconspicua Casey, 1915: 133 [original combination].
Cyclocephala inconspicua (Casey) [new combination by Arrow 1937b: 8, 11].
Cyclocephala (Spilosota) hirta LeConte [synonymy by Saylor 1937: 68].
**syn.** Spilosota magister Casey, 1915: 132 [original combination].
Cyclocephala magister (Casey) [new combination by Arrow 1937b: 8, 12].
Cyclocephala (Spilosota) hirta LeConte [synonymy by Saylor 1937: 68].
**syn.** Spilosota nubeculina Casey, 1915: 131 [original combination].
Cyclocephala nubeculina (Casey) [new combination by Arrow 1937b: 8, 13].
Cyclocephala hirta LeConte [synonymy by Saylor 1945: 384].
**syn.** Spilosota pallidissima Casey, 1915: 133 [original combination].
Cyclocephala pallidissima (Casey) [new combination by Arrow 1937b: 8, 14].
Cyclocephala (Spilosota) hirta LeConte [synonymy by Saylor 1937: 68].

#### Types.

Endrődi (1966) was uncertain about the housing institutions for the types of *C. hirta* and its synonyms. He thought they were all at USNM.

#### Distribution.

MEXICO: Aguascalientes, Baja California, Chihuahua, Coahuila, Durango, Estado de México, Sonora. USA: Arizona, California, Colorado, Iowa, Kansas, Nebraska, Nevada, New Mexico, Oklahoma, South Dakota, Texas, Utah.

#### References.

LeConte 1861, Gerstaecker 1862, Harold 1869b, Horn 1871, Crotch 1873, Henshaw 1885, Fall 1901, Casey 1915, Leng 1920, Dawson 1922, Moore 1937, Arrow 1937b, Saylor 1936, 1937, 1945, 1948, Blackwelder 1939, 1944, Bohart 1947, Blackwelder and Blackwelder 1948, Howden and Endrődi 1966, Hatch 1971, Kirk and Balsbaugh 1975, Ritcher and Baker 1974, Pike et al. 1976, 1977, Endrődi 1966, 1969b, 1985a, Hardy 1991, Ratcliffe 1991, Stahly and Klein 1992, Tanada and Kaya 1993, Thurston et al. 1993, 1994, Kaya et al. 1992, 1993, 1995, Cranshaw and Ward 1996, Poole and Gentili 1996, Ratcliffe and Morón 1997, Converse and Grewal 1998, Koppenhöfer and Kaya 1996, 1997, 1998, Theunis 1998, Bauernfeind 2001, Stock et al. 2001, Koppenhöfer et al. 1999, 2000a, b, c, 2002a, b, 2007, 2012, 2013, Riley and Wolfe 2003, Yildrim and Hoy 2003, Ansari et al. 2004, Cranshaw 2004, Mottern et al. 2004, Koppenhöfer and Grewal 2005, Grewal et al. 2005, Jaramillo et al. 2005, Jackson and Klein 2006, Oestergaard et al. 2006, Stuart et al. 2006, Koppenhöfer 2008, Koppenhöfer and Fuzy 2003b, c, 2008b, Ratcliffe and Paulsen 2008, Morales-Rodriguez and Peck 2009, Smith 2003, 2009, Bélair et al. 2010, Holmstrup et al. 2010, Negrisoli et al. 2010, Krajcik 2005, 2012, Perera et al. 2012, Breeschoten et al. 2013, de Coninck et al. 2013, Ratcliffe et al. 2013, Hussaini 2014, Li and Bouwer 2014, Christians et al. 2016, Gyawaly et al. 2016, Del Valle et al. 2017, Wu et al. 2017, Ratcliffe and Cave 2017.

### Cyclocephala hirta pilosicollis

Taxon classificationAnimaliaColeopteraScarabaeidae

Saylor, 1936

Spilosota hirta pilosicollis Saylor, 1936: 2 [original combination].
Cyclocephala (Spilosota) hirta pilosicollis Saylor [new subgeneric classification by Saylor 1937: 69].
Cyclocephala pilosicollis (Saylor) [removal of subgeneric classification and new species status by Arrow 1937b: 8].
Cyclocephala hirta LeConte [synonymy by Ratcliffe et al. 2013: 165].
Cyclocephala hirta pilosicollis Saylor [revalidated subspecies status by Ratcliffe and Cave 2017: 67].

#### Types.

Type at USNM (Endrődi 1966).

#### Distribution.

USA: California.

#### References.

Saylor 1936, 1937, 1945, Blackwelder and Blackwelder 1948, Howden and Endrődi 1966, Pike et al. 1976, Endrődi 1966, 1985a, Hardy 1991, Poole and Gentili 1996, Krajcik 2005, 2012, Ratcliffe et al. 2013, Ratcliffe and Cave 2017.

### Cyclocephala histrionica

Taxon classificationAnimaliaColeopteraScarabaeidae

Burmeister, 1847

Cyclocephala histrionica Burmeister, 1847: 41 [original combination].
Paraspidolea histrionica (Burmeister) [new combination by Arrow 1937b: 7].
Cyclocephala histrionica Burmeister [revised combination by Endrődi 1966: 77, 127, 217–218].
**syn.** Aspidolea (Aspidolites) atricollis Höhne, 1922c: 374–376 [original combination].
Aspidolea (Aspidolella) atricollis Höhne [new subgeneric classification by Prell 1936: 146].
Cyclocephala histrionica Burmeister [synonymy by Endrődi 1966: 217, 339].

#### Types.

Lectotype ♂ of *C. histrionica* at MLUH (Endrődi 1966). Lectotype ♂ of *A. atricollis* at ZMHB (Endrődi 1966).

#### Distribution.

BRAZIL: Minas Gerais, São Paulo.

#### References.

Burmeister 1847, Harold 1869b, Höhne 1922c, Arrow 1937b, Blackwelder 1944, Pike et al. 1976, Endrődi 1966, 1985a, Krajcik 2005, 2012.

### Cyclocephala holmbergi

Taxon classificationAnimaliaColeopteraScarabaeidae

Martínez, 1968

Cyclocephala holmbergi Martínez, 1968b: 23–26 [original combination].

#### Types.

Holotype ♂ at MACN (Antonio Martínez Collection) (Martínez 1968b).

#### Distribution.

BOLIVIA: Santa Cruz.

#### References.

Martínez 1968b, Pike et al. 1976, Endrődi 1985a, Krajcik 2005, 2012.

### Cyclocephala howdenannae

Taxon classificationAnimaliaColeopteraScarabaeidae

Endrődi, 1975

Cyclocephala howdenannae Endrődi, 1975b: 257–258 [original combination].

#### Types.

Holotype ♂ at CMNC (Henry and Anne Howden Collection) (Endrődi 1975b).

#### Distribution.

COLOMBIA: Valle del Cauca.

#### References.

Pike et al. 1976, Endrődi 1975b, 1985a, Ratcliffe 1992a, Restrepo et al. 2003, Krajcik 2005, 2012, Gasca-Álvarez and Deloya 2016.

### Cyclocephala huamilule

Taxon classificationAnimaliaColeopteraScarabaeidae

Romero-López & Morón, 2017

Cyclocephala huamilule Romero-López & Morón, 2017: 890–894 [original combination].

#### Types.

Holotype ♂ at MXAL (Romero-López and Morón 2017).

#### Distribution.

MEXICO: Guerrero (Romero-López and Morón 2017).

#### References.

Romero-López and Morón 2017.

### Cyclocephala huesingi

Taxon classificationAnimaliaColeopteraScarabaeidae

Endrődi, 1964

Cyclocephala huesingi Endrődi, 1964: 436–438 [original combination].

#### Types.

Holotype ♂ at MLUH (Endrődi 1966, Endrődi [1964] mistakenly stated the holotype was at BMNH).

#### Distribution.

COLOMBIA. VENEZUELA: Mérida.

#### References.

Pike et al. 1976, Endrődi 1964, 966, 1985a, Restrepo et al. 2003, Krajcik 2005, 2012, Gasca-Álvarez and Deloya 2016.

### Cyclocephala iani

Taxon classificationAnimaliaColeopteraScarabaeidae

Ratcliffe, 1992

Cyclocephala iani Ratcliffe, 1992b: 183 [original combination].

#### Types.

Holotype ♂ at UNSM (Ratcliffe 1992b).

#### Distribution.

BRAZIL: Amazonas.

#### References.

Ratcliffe 1992b, Krajcik 2005, 2012.

### Cyclocephala immaculata ferruginea

Taxon classificationAnimaliaColeopteraScarabaeidae

(Fabricius, 1801)

Melolontha ferruginea Fabricius, 1801: 170 [original combination].
Cyclocephala ferruginea (Fabricius) [new combination by Burmeister 1847: 58].
Cyclocephala immaculata (Olivier) [synonymy by Chalumeau and Gruner 1977: 582].
Cyclocephala immaculata ferruginea (Fabricius) [revalidated subspecies status by Endrődi 1985a: 101].
**syn.** Melolontha nigriceps Gyllenhal, 1817a: 188–189 [original combination].
Cyclocephala ferruginea (Fabricius) [synonymy by Burmeister 1847: 58].

#### Types.

Lectotype ♀ of *M. ferruginea* deposited at ZMUK, now housed at ZMUC (Endrődi 1966). The type of *M. nigriceps* was unknown to Endrődi (1966).

#### Distribution.

FRENCH GUIANA: Cayenne.

#### References.

Fabricius 1801, Gyllenhal 1817a, Schönherr 1817, Dejean 1821, 1833, 1836b, Sturm 1843, Burmeister 1847, Harold 1869b, Arrow 1937b, Blackwelder 1944, Zimsen 1964, Pike et al. 1976, Chalumeau and Gruner 1977, Dechambre 1980, Endrődi 1966, 1985a, Ponchel 2011, Krajcik 2005, 2012, Ratcliffe and Cave 2015.

### Cyclocephala immaculata immaculata

Taxon classificationAnimaliaColeopteraScarabaeidae

(Olivier, 1789)

Melolontha immaculata Olivier, 1789: 29 [original combination].
Cyclocephala immaculata (Olivier) [new combination by Dejean 1821: 57].
Ochrosidia (Ochrosidia) immaculata (Olivier) [new combination and new subgeneric classification by Casey 1915: 112, 142].
Cyclocephala immaculata (Olivier) [revised combination and removal of subgeneric classification by Arrow, 1937b: 8, 11].
**syn.** Cyclocephala danforthi Chapin, 1935b: 69 [original combination].
Cyclocephala immaculata (Olivier) [synonymy by Chalumeau and Gruner 1977: 582].

#### Types.

Invalid neotype ♂ of *M. immaculata* at HNHM (Endrődi Collection) (Endrődi 1966). Chalumeau and Gruner (1977) stated this neotype was at MNHN. Type of *C. danforthi* at USNM (Endrődi 1966).

#### Distribution.

ANTIGUA: St. Paul. BARBUDA. BRITISH VIRGIN ISLANDS: Anegada. DOMINICA: St. Paul. GUADELOUPE: Grande-Terre, La Désirade. JAMAICA: St. Andrew. SAINT BÁRTHÉLEMY. SAINT KITTS AND NEVIS: Nevis, St. Kitts. SAINT MARTIN. U. S. VIRGIN ISLANDS: Buck Island, St. Croix.

#### References.

Olivier 1789, Dejean 1821, 1833, 1836b, Sturm 1843, Reiche 1859, Bates 1888, Leng and Mutchler 1914, Casey 1915, Chapin 1935b, Arrow 1937b, Blackwelder 1944, Paulian 1947, Howden and Endrődi 1966, Pike et al. 1976, Chalumeau and Gruner 1977, Endrődi 1963, 1966, 1969b, 1985a, Dupuis 1996, Riley and Wolfe 2003, Krajcik 2005, 2012, Breeschoten et al. 2013, Ratcliffe and Cave 2015, Peck 2016.

### Cyclocephala inca

Taxon classificationAnimaliaColeopteraScarabaeidae

Endrődi, 1966

Cyclocephala inca Endrődi, 1966: 73, 133, 221–222 [original combination].

#### Types.

Holotype ♂ at NHMB (Frey Collection) (Endrődi 1966).

#### Distribution.

BOLIVIA: Beni. COLOMBIA. PERU: Cusco, Madre de Dios, Puno.

#### References.

Pike et al. 1976, Dechambre 1979a, 1980, Endrődi 1966, 1985a, Núñez-Avellaneda and Neita-Moreno 2009, Krajcik 2005, 2012, Moore et al. 2015, Ratcliffe et al. 2015, Gasca-Álvarez and Deloya 2016.

### Cyclocephala insulicola

Taxon classificationAnimaliaColeopteraScarabaeidae

Arrow, 1937

Cyclocephala insulicola Arrow, 1937a: 40–41 [original combination].

#### Types.

Type at BMNH (Endrődi 1966).

#### Distribution.

GUADELOUPE: Basse-Terre, Grande-Terre. MARTINIQUE: Saint-Pierre. ST. VINCENT.

#### References.

Arrow 1937a, b, Blackwelder 1944, Paulian 1947, Pike et al. 1976, Endrődi 1966, 1985a, Gruner 1975, Gruner and Marival 1974, Chalumeau and Gruner 1977, Chalumeau 1983, Dutrillaux et al. 2007, Krajcik 2005, 2012, Giannoulis et al. 2012, Dutrillaux et al. 2013, 2014, Ratcliffe and Cave 2015, Peck 2016.

### Cyclocephala isabellina

Taxon classificationAnimaliaColeopteraScarabaeidae

Höhne, 1923

Cyclocephala isabellina Höhne, 1923b: 368–369 [original combination].

#### Types.

Lectotype ♂ at ZMHB (Endrődi 1966).

#### Distribution.

COLOMBIA: Cundinamarca, Meta, Nariño. ECUADOR. PERU.

#### References.

Höhne 1923b, Arrow 1937b, Blackwelder 1944, Martínez 1968c, 1975b, Pike et al. 1976, Endrődi 1964, 1966, 1985a, Dechambre 1999, Restrepo et al. 2003, Krajcik 2005, 2012, López-García et al. 2015, Ratcliffe et al. 2015, Gasca-Álvarez and Deloya 2016.

### Cyclocephala isthmiensis

Taxon classificationAnimaliaColeopteraScarabaeidae

Ratcliffe, 1992

Cyclocephala isthmiensis Ratcliffe, 1992a: 219–220 [original combination].

#### Types.

Holotype ♂ at UNSM (Ratcliffe 1992a).

#### Distribution.

PANAMA: Former Canal Zone.

#### References.

Ratcliffe 1992a, 2002a, 2003, Krajcik 2005, 2012.

### Cyclocephala italoi

Taxon classificationAnimaliaColeopteraScarabaeidae

Dupuis, 1999

Cyclocephala italoi Dupuis, 1999: 185–186 [original combination].

#### Types.

Holotype ♂ at MNHN (Dupuis 1999).

#### Distribution.

ECUADOR: Imbabura.

#### References.

Dupuis 1999, Krajcik 2005, 2012.

### Cyclocephala jalapensis

Taxon classificationAnimaliaColeopteraScarabaeidae

Casey, 1915

Cyclocephala (Isocoryna) jalapensis Casey, 1915: 136–137 [original combination].
Cyclocephala jalapensis Casey [removal of subgeneric classification by Arrow 1937b: 8, 11].

#### Types.

Type at USMN (Endrődi 1966).

#### Distribution.

MEXICO: Chiapas, Hidalgo, Oaxaca, Puebla, Querétaro, Veracruz.

#### References.

Casey 1915, Arrow 1937b, Blackwelder 1944, Pike et al. 1976, Endrődi 1966, 1985a, Morón 1977b, 1994, Thomas 1993, Dieringer and Delgado 1994, Dieringer and Espinosa 1994, Ratcliffe and Morón 1997, Carrillo-Ruiz and Morón 2003, Múñoz-Hernández et al. 2008, Krajcik 2005, 2012, Morón and Márquez 2012, Ratcliffe et al. 2013, Martínez-Morales and Morón 2013, Morón et al. 2014, Romero-López and Morón 2017.

### Cyclocephala jauffreti

Taxon classificationAnimaliaColeopteraScarabaeidae

Dechambre, 1979

Cyclocephala jauffreti Dechambre, 1979a: 163–164 [original combination].

#### Types.

Holotype ♂ at MNHN (Dechambre 1979a).

#### Distribution.

BRAZIL: Pará.

#### References.

Dechambre 1979a, Endrődi 1985a, Krajcik 2005, 2012.

### Cyclocephala kahanoffae

Taxon classificationAnimaliaColeopteraScarabaeidae

Martínez, 1975

Cyclocephala kahanoffae Martínez, 1975b: 270–274 [original combination].

#### Types.

Holotype ♂ at MACN (Antonio Martínez Collection) (Martínez 1975b).

#### Distribution.

BRAZIL: Federal District.

#### References.

Martínez 1975b, Pike et al. 1976, Endrődi 1985a, Krajcik 2005, 2012.

### Cyclocephala kaszabi

Taxon classificationAnimaliaColeopteraScarabaeidae

Endrődi, 1964

Cyclocephala kaszabi Endrődi, 1964: 433–435 [original combination].

#### Types.

Holotype ♂ at BMNH (Endrődi 1964).

#### Distribution.

COLOMBIA: Risaralda. COSTA RICA: Alajuela, Cartago, Guanacaste, Heredia, Limón, Puntarenas. ECUADOR. GUATEMALA: Huehuetenango, Izabal. HONDURAS: Atlántida, Colón, Cortés, Olancho, Yoro. NICARAGUA: Jinotega, RAA Norte. PANAMA: Bocas del Toro, Chiriquí, Coclé, Colón, Former Canal Zone, Panamá, San Blas. PERU.

#### References.

Pike et al. 1976, Endrődi 1964, 1966, 1985a, Young 1986, 1987, 1988a, 1990, Croat 1997, Ratcliffe 1992a, 2002a, 2003, Garcia-Robledo et al. 2004, 2005, Ratcliffe and Cave 2006, Krajcik 2005, 2012, García-López et al. 2013, Ratcliffe et al. 2013, 2015, Gasca-Álvarez and Deloya 2016.

### Cyclocephala kechua

Taxon classificationAnimaliaColeopteraScarabaeidae

(Martínez, 1957)

Mimeoma kechua Martínez, 1957: 29–32 [original combination].
Cyclocephala kechua (Martínez) [new combination by Endrődi 1966: 75, 129, 225].

#### Types.

Holotype ♂ at MACN (Antonio Martínez Collection) (Martínez 1957).

#### Distribution.

ARGENTINA: Jujuy, Salta, Tucumán. BOLIVIA: Santa Cruz.

#### References.

Martínez 1957, 1975b, Pike et al. 1976, Endrődi 1966, 1985a, Dechambre 1999, Krajcik 2005, 2012.

### Cyclocephala krombeini

Taxon classificationAnimaliaColeopteraScarabaeidae

Endrődi, 1979

Cyclocephala krombeini Endrődi, 1979: 215 [original combination].
**syn.** Cyclocephala rorschachoides Ratcliffe, 1992a: 227–229 [original combination].
Cyclocephala krombeini Endrődi [synonymy by Ratcliffe 2002a: 29].

#### Types.

Holotype ♂ of *C. krombeini* at USNM (Endrődi 1979). Holotype ♂ of *C. rorschachoides* at UNSM (Ratcliffe 1992a).

#### Distribution.

COLOMBIA: Chocó. COSTA RICA: Heredia, Limón, Puntarenas. PANAMA: Bocas del Toro, Chiriquí, Colón, Former Canal Zone, Panamá, San Blas.

#### References.

Endrődi 1979, 1985a, Joly 2000a, Ratcliffe 1992a, 2002a, 2003, Neita-Moreno 2011, Krajcik 2005, 2012, Gasca-Álvarez and Deloya 2016.

### Cyclocephala kuntzeniana

Taxon classificationAnimaliaColeopteraScarabaeidae

Höhne, 1923

Cyclocephala kuntzeniana Höhne, 1923b: 366–368 [original combination].

#### Types.

Lectotype ♂ at ZMHB (Endrődi 1966).

#### Distribution.

BOLIVIA: Cochabamba. BRAZIL: Amazonas. COLOMBIA: Cundinamarca, Meta. FRENCH GUIANA. SURINAME: Sipaliwini. VENEZUELA: Táchira.

#### References.

Höhne 1923b, Arrow 1937b, Blackwelder 1944, Martínez 1967, 1975b, Pike et al. 1976, Endrődi 1964, 1966, 1985a, Dechambre 1979a, Restrepo et al. 2003, Ponchel 2011, Krajcik 2005, 2012, López-García et al. 2015, Gasca-Álvarez and Deloya 2016.

### Cyclocephala labidion

Taxon classificationAnimaliaColeopteraScarabaeidae

Ratcliffe, 2003

Cyclocephala labidion Ratcliffe, 2003: 64, 68, 74, 79, 143–144 [original combination].

#### Types.

Holotype ♂ at UNSM (Ratcliffe 2003).

#### Distribution.

COSTA RICA: Puntarenas. PANAMA: Chiriquí.

#### References.

Ratcliffe 2003, Krajcik 2005, 2012.

### Cyclocephala lachaumei

Taxon classificationAnimaliaColeopteraScarabaeidae

Dechambre, 1992

Cyclocephala lachaumei Dechambre, 1992: 57 [original combination].

#### Types.

Holotype ♂ at MNHN (Dechambre 1992).

#### Distribution.

BOLIVIA: La Paz.

#### References.

Dechambre 1992, Dupuis 2008, 2014, Krajcik 2005, 2012.

### Cyclocephala laevis

Taxon classificationAnimaliaColeopteraScarabaeidae

Arrow, 1937

Cyclocephala laevis Arrow, 1937a: 40 [original combination].

#### Types.

Type at BMNH (Endrődi 1966).

#### Distribution.

DOMINICAN REPUBLIC: Barahona, Dajabón, Distrito Nacional, Duarte, Elías Piña, La Altagracia, La Romana, La Vega, Monseñor Nouel, Monte Cristi, Monte Plata, Pedernales, Puerto Plata, Samana, San Cristóbal, San José de Ocoa, San Juan, Santiago, Santo Domingo, Valverde. HAITI: Ouest.

#### References.

Arrow 1937a, b, Blackwelder 1944, Pike et al. 1976, Endrődi 1966, 1985a, Krajcik 2005, 2012, Ratcliffe and Cave 2015.

### Cyclocephala lamarcki

Taxon classificationAnimaliaColeopteraScarabaeidae

Dechambre, 1999

Cyclocephala lamarcki Dechambre, 1999: 11 [original combination].

#### Types.

Holotype ♂ at MNHN (Dechambre 1999).

#### Distribution.

ECUADOR: Napo.

#### References.

Dechambre 1999, Krajcik 2005, 2012.

### Cyclocephala laminata

Taxon classificationAnimaliaColeopteraScarabaeidae

Burmeister, 1847

Cyclocephala laminata Burmeister, 1847: 57 [original combination].
**syn.** Dichromina regularis Casey, 1915: 161 [original combination].
Cyclocephala regularis (Casey) [new combination by Arrow 1937b: 8, 15].
Cyclocephala laminata Burmeister [synonymy by Ratcliffe et al. 2013: 171].

#### Types.

Lectotype ♂ of *C. laminata* at MLUH (Endrődi 1966). Endrődi (1966) did not examine or report the type housing location for *D. regularis* (probably at USNM).

#### Distribution.

ARGENTINA: Buenos Aires, Córdoba, Santa Fe. BOLIVIA: Beni, Cochabamba, La Paz, Santa Cruz. BRAZIL: Bahia, Espírito Santo, Minas Gerais, Pará, Paraná, Rio de Janeiro, Rio Grande do Sul, Santa Catarina, São Paulo. COLOMBIA: Bolívar, Valle del Cauca. FRENCH GUIANA: Cayenne, Kourou, St.-Laurent du Maroni. GUATEMALA: Alta Verapaz, El Progreso, Escuintla, Zacapa. GUYANA. MEXICO: Chiapas, Durango, Oaxaca. PARAGUAY: Concepción, Distrito Capital, Paraguarí. PERU: Huánuco, Madre de Dios. SURINAME. UNITED STATES: Texas.

#### References.

Burmeister 1847, Harold 1869b, Tremoleras 1910, Casey 1915, Arrow 1937b, Blackwelder 1944, Gruner 1971, Pike et al. 1976, Dechambre 1979a, Endrődi 1966, 1973a, 1985a, Hardy 1991, Dechambre 1992, Dupuis 1996, Poole and Gentili 1996, Ratcliffe and Morón 1997, Rosa et al. 1995, 1999, Lachance et al. 2001, Freitas et al. 2002, Restrepo et al. 2003, Riley and Wolfe 2003, Pinto et al. 2004, Marques and Gil-Santana 2009, Krajcik 2005, 2012, Breeschoten et al. 2013, Ratcliffe et al. 2013, Ponchel 2011, 2015, Gasca-Álvarez and Deloya 2016, Ratcliffe and Cave 2017.

#### Remarks.

*Cyclocephala laminata* was reported from Costa Rica, Panama, and Puerto Rico (Endrődi 1966, Dechambre 1979a). *Cyclocephala laminata* has not been recorded from these areas again, and these records may be based on misidentifications (Ratcliffe 2003, Ratcliffe and Cave 2013). Tremoleras (1910) reported *C. laminata* from Uruguay.

### Cyclocephala larssoni

Taxon classificationAnimaliaColeopteraScarabaeidae

Endrődi, 1964

Cyclocephala larssoni Endrődi, 1964: 451–452 [original combination].

#### Types.

Holotype ♂ at USNM (Endrődi 1964).

#### Distribution.

GUATEMALA: El Progresso, Zacapa. MEXICO: Guerrero, Jalisco, Nayarit, Oaxaca, Sonora. NICARAGUA: Chinandega, León, Managua, Masaya.

#### References.

Pike et al. 1976, Endrődi 1964, 1966, 1985a, Maes and Téllez Robleto 1988, Morón et al. 1988, Maes 1987, 1994, Ratcliffe and Morón 1997, Navarrete-Heredia et al. 2001, Ratcliffe and Cave 2006, Krajcik 2005, 2012, Ratcliffe et al. 2013, Deloya et al. 2014a.

### Cyclocephala latericia

Taxon classificationAnimaliaColeopteraScarabaeidae

Höhne, 1923

Cyclocephala latericia Höhne, 1923b: 360–362 [original combination].

#### Types.

Lectotype ♂ at ZMHB (Endrődi 1966).

#### Distribution.

ARGENTINA: Misiones. BOLIVIA: Santa Cruz. BRAZIL: Goiás, Mato Grosso, Pará, Pernambuco, Rio Grande do Norte, Rio Grande do Sul, Santa Catarina. PARAGUAY: Itapúa.

#### References.

Höhne 1923b, Arrow 1937b, Blackwelder 1944, Martínez 1968a, Pike et al. 1976, Dechambre 1979a, Endrődi 1966, 1985a, Cavalcante et al. 2009, Krajcik 2005, 2012, Breeschoten et al. 2013.

### Cyclocephala latipennis

Taxon classificationAnimaliaColeopteraScarabaeidae

Arrow, 1911

Cyclocephala latipennis Arrow, 1911: 174 [original combination].

#### Types.

Type at BMNH (Endrődi 1966).

#### Distribution.

ECUADOR: Morona-Santiago.

#### References.

Arrow 1911, 1937b, Blackwelder 1944, Pike et al. 1976, Endrődi 1966, 1985a, Dechambre 1997, Krajcik 2005, 2012.

### Cyclocephala latreillei

Taxon classificationAnimaliaColeopteraScarabaeidae

Dechambre, 1999

Cyclocephala latreillei Dechambre, 1999: 12 [original combination].

#### Types.

Holotype ♂ at MNHN (Dechambre 1999).

#### Distribution.

ECUADOR: Cotopaxi.

#### References.

Dechambre 1999, Krajcik 2005, 2012.

### Cyclocephala lecourti

Taxon classificationAnimaliaColeopteraScarabaeidae

Dechambre, 1992

Cyclocephala lecourti Dechambre, 1992: 63–64 [original combination].

#### Types.

Holotype ♂ at MNHN (Dechambre 1992).

#### Distribution.

BOLIVIA: La Paz.

#### References.

Dechambre 1992, Krajcik 2005, 2012.

### Cyclocephala letiranti

Taxon classificationAnimaliaColeopteraScarabaeidae

Young, 1992

Cyclocephala letiranti Young, 1992: 52–55 [original combination].

#### Types.

Holotype ♂ at CAS (Young 1992).

#### Distribution.

COLOMBIA. COSTA RICA: Alajuela, Heredia, Puntarenas.

#### References.

Young 1992, Ratcliffe 2003, Krajcik 2005, 2012, Gasca-Álvarez and Deloya 2016.

### Cyclocephala lichyi

Taxon classificationAnimaliaColeopteraScarabaeidae

Dechambre, 1980

Cyclocephala lichyi Dechambre, 1980: 46 [original combination].

#### Types.

Holotype ♂ at MNHN (Dechambre 1980).

#### Distribution.

VENEZUELA: Amazonas.

#### References.

Dechambre 1980, 1999, Endrődi 1985a, Dupuis 1996, Krajcik 2005, 2012.

### Cyclocephala ligyrina

Taxon classificationAnimaliaColeopteraScarabaeidae

Bates, 1888

Cyclocephala ligyrina Bates, 1888: 309 [original combination].
Stigmalia ligyrina (Bates) [new combination by Casey 1915: 123].
Cyclocephala ligyrina Bates [revised combination by Arrow 1937b: 8, 12].

#### Types.

Type at BMNH (Endrődi 1966).

#### Distribution.

BRAZIL: Espírito Santo. COLOMBIA: Boyacá, Cauca, Chocó, Meta, Tolima, Valle del Cauca. COSTA RICA: Alajuela, Cartago, Guanacaste, Heredia, Limón, Puntarenas. ECUADOR: Morona-Santiago. GUATEMALA: Izabal. HONDURAS: Atlántida, Colón. NICARAGUA: Chontales, Jinotega, Matagalpa, RAA Norte. PANAMA: Bocas del Toro, Chiriquí, Coclé, Former Canal Zone, Panamá. PERU: Junín.

#### References.

Bates 1888, Casey 1915, Arrow 1937b, Blackwelder 1944, Pike et al. 1976, Endrődi 1964, 1966, 1967b, 1975b, 1985a, Young 1986, 1988a, 1990, Maes 1987, 1994, Croat 1997, Joly 2000a, Ratcliffe 2002a, 2003, Restrepo et al. 2003, Ratcliffe and Cave 2006, Neita-Moreno et al. 2006, Neita-Moreno 2011, Krajcik 2005, 2012, García-López et al. 2013, Ratcliffe et al. 2013, 2015, López-García et al. 2015, Gasca-Álvarez and Deloya 2016.

### Cyclocephala lineata

Taxon classificationAnimaliaColeopteraScarabaeidae

Dupuis, 2008

Cyclocephala lineata Dupuis, 2008: 123–124 [original combination].

#### Types.

Holotype ♂ at FDPC (Dupuis 2008).

#### Distribution.

COLOMBIA: Boyacá.

#### References.

Dupuis 2008, Krajcik 2012, Gasca-Álvarez and Deloya 2016.

### Cyclocephala lineigera

Taxon classificationAnimaliaColeopteraScarabaeidae

Höhne, 1923

Cyclocephala lineigera Höhne, 1923b: 355–357 [original combination].

#### Types.

Lectotype ♂ at ZMHB (Endrődi 1966).

#### Distribution.

BRAZIL: Amazonas. COLOMBIA: Caquetá, Cauca. PERU: Loreto.

#### References.

Höhne 1923b, Arrow 1937b, Blackwelder 1944, Pike et al. 1976, Endrődi 1964, 1966, 1985a, Restrepo et al. 2003, Krajcik 2005, 2012, Ratcliffe et al. 2015, Gasca-Álvarez and Deloya 2016.

#### Remarks.

Endrődi (1966, 1985a) reported a single *C. lineigera* specimen from Guatemala (Panzos). *Cyclocephala lineigera* has not been reported from Central America since this record (e.g., Ratcliffe et al. 2013) and this species is possibly distributed only in South America.

### Cyclocephala liomorpha

Taxon classificationAnimaliaColeopteraScarabaeidae

Arrow, 1911

Cyclocephala liomorpha Arrow, 1911: 174–175 [original combination].

#### Types.

Type at BMNH (Endrődi 1966).

#### Distribution.

BRAZIL: Amazonas, Pará. GUYANA. PERU.

#### References.

Arrow 1911, 1937b, Blackwelder 1944, Pike et al. 1976, Dechambre 1979a, Endrődi 1964, 1966, 1985a, Krajcik 2005, 2012, Ratcliffe et al. 2015.

### Cyclocephala literata

Taxon classificationAnimaliaColeopteraScarabaeidae

Burmeister, 1847

Cyclocephala literata Burmeister, 1847: 60–61 [original combination].

#### Types.

Lectotype ♂ at MLUH (Endrődi 1966).

#### Distribution.

BRAZIL: Rio de Janeiro, Santa Catarina, São Paulo. FRENCH GUIANA: St.-Laurent du Maroni.

#### References.

Burmeister 1847, Arrow 1937b, Blackwelder 1944, Guimarães 1944, Pike et al. 1976, Endrődi 1966, 1969b, 1985a, Krajcik 2005, 2012, Gottsberger and Silberbauer-Gottsberger 2006, Gottsberger et al. 2012, Breeschoten et al. 2013, Maia et al. 2014, Gottsberger 1986, 1989, 1992, 2016.

### Cyclocephala lizeri

Taxon classificationAnimaliaColeopteraScarabaeidae

Martínez, 1964

Cyclocephala lizeri Martínez, 1964: 87–91 [original combination].

#### Types.

Holotype ♂ at MACN (Antonio Martínez Collection) (Martínez 1964).

#### Distribution.

ARGENTINA: Salta. BOLIVIA: Chuquisaca.

#### References.

Martínez 1964, Pike et al. 1976, Endrődi 1966, 1971b, 1985a, Krajcik 2005, 2012.

### Cyclocephala longa

Taxon classificationAnimaliaColeopteraScarabaeidae

Endrődi, 1963

Cyclocephala longa Endrődi, 1963: 332 [original combination].

#### Types.

Holotype ♂ at ZSMC (Endrődi 1966).

#### Distribution.

BOLIVIA: Santa Cruz. BRAZIL: Acre. COLOMBIA: Amazonas, Boyacá, Casanare, Cundinamarca.

#### References.

Pike et al. 1976, Endrődi 1963, 1966, 1985a, Ratcliffe 1992b, Dechambre 1999, Krajcik 2005, 2012, López-García et al. 2015, Gasca-Álvarez and Deloya 2016.

### Cyclocephala longicollis

Taxon classificationAnimaliaColeopteraScarabaeidae

Burmeister, 1847

Cyclocephala longicollis Burmeister, 1847: 43 [original combination].

#### Types.

Lectotype ♂ at MLUH (Endrődi 1966).

#### Distribution.

BRAZIL. COLOMBIA.

#### References.

Burmeister 1847, Reiche 1859, Harold 1869b, Arrow 1937b, Blackwelder 1944, Pike et al. 1976, Endrődi 1966, 1985a, Krajcik 2005, 2012, Restrepo et al. 2003, Gasca-Álvarez and Deloya 2016.

#### Remarks.

A single specimen of *C. longicollis* was reported from Mexico without further details (Endrődi 1966, 1985a). This specimen was likely mislabeled, and *C. longicollis* Burmeister is considered a South American species (Ratcliffe et al. 2013).

### Cyclocephala longimana

Taxon classificationAnimaliaColeopteraScarabaeidae

Dechambre, 1980

Cyclocephala longimana Dechambre, 1980: 46–47 [original combination].

#### Types.

Holotype ♂ at MNHN (Dechambre 1980).

#### Distribution.

BRAZIL: Minas Gerais.

#### References.

Dechambre 1980, Endrődi 1985a, Ratcliffe 1992b, Krajcik 2005, 2012.

### Cyclocephala longitarsis

Taxon classificationAnimaliaColeopteraScarabaeidae

Dechambre, 1999

Cyclocephala longitarsis Dechambre 1999: 13 [original combination].

#### Types.

Holotype ♂ at MNHN (Dechambre 1999).

#### Distribution.

ECUADOR: Esmeraldas, Pichincha.

#### References.

Dechambre 1999, Krajcik 2005, 2012.

### Cyclocephala longula

Taxon classificationAnimaliaColeopteraScarabaeidae

LeConte, 1863

Cyclocephala longula LeConte, 1863: 79 [original combination].
Ochrosidia (Ochrosidia) longula (LeConte) [new combination and new subgeneric classification by Casey 1915: 142].
Cyclocephala longula LeConte [revised combination and removal of subgeneric classification by Arrow 1937b: 8, 12].
**syn.** Ochrosidia (Ochrosidia) abrupta Casey, 1915: 152 [original combination].
Cyclocephala abrupta (Casey) [new combination and removal of subgeneric classification by Arrow 1937b: 8].
Cyclocephala (Spilosota) abrupta (Casey) [new subgeneric classification by Saylor 1937: 69].
Cyclocephala longula LeConte [synonymy by Saylor 1945: 384].
**syn.** Ochrosidia (Ochrosidia) ambiens Casey, 1915: 155 [original combination].
Cyclocephala ambiens (Casey) [new combination and removal of subgeneric classification by Arrow 1937b: 8].
Cyclocephala longula LeConte [synonymy by Saylor 1945: 384].
**syn.** Ochrosidia (Ochrosidia) rustica Casey, 1915: 157 [original combination].
Cyclocephala californica Arrow, 1937b: 9 [original combination, new replacement name for Cyclocephala rustica (Casey)].
Cyclocephala (Spilosota) abrupta (Casey) [synonymy by Saylor 1937: 69].
Cyclocephala longula LeConte [synonymy by Saylor 1945: 384].
**syn.** Ochrosidia (Ochrosidia) marcida Casey, 1915: 155 [original combination].
Cyclocephala marcida (Casey) [new combination and removal of subgeneric classification by Arrow 1937b, 8, 12].
Cyclocephala longula LeConte [synonymy by Saylor 1945: 384].
**syn.** Ochrosidia (Ochrosidia) modulata Casey, 1915: 154 [original combination].
Cyclocephala modulata (Casey) [new combination and removal of subgeneric classification by Arrow 1937b: 8, 13].
Cyclocephala longula LeConte [synonymy by Saylor 1945: 384].
**syn.** Ochrosidia (Ochrosidia) obesula Casey, 1915: 156 [original combination].
Cyclocephala obesula (Casey) [new combination by Arrow 1937b: 8, 13].
Cyclocephala (Spilosota) abrupta (Casey) [synonymy by Saylor 1937: 69].
Cyclocephala longula LeConte [synonymy by Saylor 1945: 384].
**syn.** Ochrosidia (Ochrosidia) oblongula Casey, 1915: 156 [original combination].
Cyclocephala oblongula (Casey) [new combination and removal of subgeneric classification by Arrow 1937b: 8, 13].
Cyclocephala (Spilosota) abrupta (Case) [synonymy by Saylor 1937: 69].
**syn.** Ochrosidia (Ochrosidia) phasma Casey, 1915: 153 [original combination].
Cyclocephala phasma (Casey) [new combination and removal of subgeneric classification by Arrow 1937b: 8, 14].
Cyclocephala (Spilosota) abrupta (Casey) [synonymy by Saylor 1937: 69].
**syn.** Ochrosidia (Ochrosidia) prona Casey, 1915: 157 [original combination].
Cyclocephala prona (Casey) [new combination and removal of subgeneric classification by Arrow 1937b: 8, 14].
Cyclocephala longula LeConte [synonymy by Saylor 1945: 384].
**syn.** Ochrosidia (Ochrosidia) reflexa Casey, 1915: 153 [original combination].
Cyclocephala reflexa (Casey) [new combination and removal of subgeneric classification by Arrow 1937b: 8, 15].
Cyclocephala (Spilosota) abrupta (Casey) [synonymy by Saylor 1937: 69].
**syn.** Ochrosidia (Ochrosidia) rugulifrons Casey, 1915: 154 [original combination].
Cyclocephala rugulifrons (Casey) [new combination and removal of subgeneric classification by Arrow 1937b: 8, 15].
Cyclocephala longula LeConte [synonymy by Saylor 1945: 384].

#### Types.

Type of *C. longula* at MCZ (Endrődi 1966). Types of the Casey synonyms are at USNM (Endrődi 1966).

#### Distribution.

CANADA: British Columbia. MEXICO: Baja California, Baja California Sur, Chihuahua, Sonora. UNITED STATES: Arizona, California, Colorado, Idaho, Illinois, Kansas, Montana, Nebraska, Nevada, New Mexico, Oklahoma, Oregon, South Dakota, Texas, Utah, Washington, Wyoming.

#### References.

LeConte 1863, Gerstaecker 1865, Crotch 1873, Henshaw 1885, Horn 1894, Wickham 1896, Fall 1901, Casey 1915, Leng 1920, Dawson 1922, Arrow 1937b, Moore 1937, Blackwelder 1939, 1944, Riegel 1942b, Saylor 1937, 1945, 1948, Blackwelder and Blackwelder 1948, Allred and Beck 1965, Ritcher 1966, Howden and Endrődi 1966, Arnett 1968, Pike et al. 1976, Endrődi 1966, 1969b, 1985a, Hatch 1971, Kirk and Balsbaugh 1975, Bechtel et al. 1983, Hardy 1991, McNamara 1991, Ratcliffe 1991, Poole and Gentili 1996, Ratcliffe and Morón 1997, Bauernfeind et al. 1999, Bauernfeind 2001, Riley and Wolfe 2003, Ratcliffe and Paulsen 2008, Smith 2003, 2009, Krajcik 2005, 2012, Haviland and Hernandez 2012, Bousquet et al. 2013, Breeschoten et al. 2013, Ratcliffe et al. 2013, Ratcliffe and Cave 2017.

#### Remarks.

*Cyclocephala longula* was reported from Wisconsin and Florida (USA) (Endrődi 1966, 1985a). These records are likely based on misidentifications (Ratcliffe and Paulsen 2008). Additionally, *C. longula* was reported from Nicaragua (Boaco and Managua) (Maes 1987, 1994). The validity of these records is uncertain as faunistic studies focused on Nicaragua did not report additional specimens of *C. longula* (Ratcliffe and Cave 2006).

### Cyclocephala lunulata

Taxon classificationAnimaliaColeopteraScarabaeidae

Burmeister, 1847

Cyclocephala lunulata Burmeister, 1847: 62 [original combination].
Ochrosidia (Graphalia) lunulata (Burmeister) [new combination and new subgeneric classification by Casey 1915: 159].
Cyclocephala lunulata Burmeister [revised combination and removal of subgeneric classification by Arrow 1937b: 8, 12].
**syn.** Cyclocephala nubeculosa Burmeister, 1847: 63 [original combination].
Cyclocephala lunulata Burmeister [synonymy by Endrődi 1964: 466].
**syn.** Ochrosidia (Graphalia) oblita Casey, 1915: 159 [original combination].
Cyclocephala oblita (Casey) [new combination and removal of subgeneric classification by Arrow 1937b: 8, 13].
Cyclocephala lunulata Burmeister [synonymy by Endrődi 1964: 466].

#### Types.

Lectotype ♀ of *C. lunulata* at MLUH (Endrődi 1966). Lectotype ♂ of *C. nubeculosa* at MLUH (Endrődi 1966). Type of *O. oblita* at USNM (Endrődi 1966).

#### Distribution.

ARGENTINA: Buenos Aires, Tucumán. BELIZE: Cayo, Orange Walk, Stann Creek, Toledo. BOLIVIA: Beni, Cochabamba, La Paz, Santa Cruz. BRAZIL: Acre, Amazonas, Bahia, Espírito Santo, Goiás, Minas Gerais, Paraná, Rio de Janeiro, Rio Grande do Sul, Santa Catarina, São Paulo. COLOMBIA: Antioquia, Bolívar, Boyacá, Caldas, Caquetá, Cauca, Cesar, Chocó, Córdoba, Cundinamarca, Huila, La Guajira, Meta, Nariño, Norte de Santander, Putumayo, Quindío, Risaralda, Santander, Tolima, Valle del Cauca. COSTA RICA: Alajuela, Cartago, Guanacaste, Heredia, Limón, Puntarenas, San José. ECUADOR: Bolívar, Cañar, Chimborazo, El Oro, Guayas, Loja, Morona-Santiago. EL SALVADOR: Ahuachapán, Cabañas, La Libertad, La Unión, Morazán, San Miguel, San Salvador, Santa Ana, Usulután. FRENCH GUIANA: Cayenne, Sinnamary, St.-Laurent du Maroni. GUATEMALA: Alta Verapaz, Baja Verapaz, Chiquimula, El Progreso, Escuintla, Guatemala, Huehuetenango, Izabal, Jalapa, Jutiapa, Petén, Quetzaltenango, Quiché, Talhuleu, Sacatepéquez, San Marcos, Santa Rosa, Suchitepéquez, Zacapa. GUYANA: Cuyuni-Mazaruni, East Berbice-Corentyne. HONDURAS: Atlántida, Choluteca, Comayagua, Copán, Cortés, El Paraíso, Francisco Morazán, Gracias a Dios, Intibucá, Isla de la Bahía, Lempira, Ocotepeque, Olancho, Santa Bárbara, Valle, Yoro. MEXICO: Aguascalientes, Chiapas, Colima, Baja California Sur, Campeche, Chiapas, Coahuila, Distrito Federal, Estado de México, Guanajuato, Guerrero, Hidalgo, Jalisco, Michoacán, Morelos, Nayarit, Oaxaca, Puebla, Querétaro, Quintana Roo, San Luis Potosí, Sinaloa, Sonora, Tabasco, Tamaulipas, Veracruz, Yucatán, Zacatecas. NICARAGUA: Carazo, Chinandega, Chontales, Estelí, Jinotega, León, Managua, Masaya, Matagalpa, RAA Norte, RAA Sur, Río San Juan. PANAMA: Bocas del Toro, Chiriquí, Coclé, Colón, Darien, Former Canal Zone, Veraguas. PARAGUAY: Alto Paraná, Amambay. PERU: Cusco, Junín, Lima, Madre de Dios, Pasco. TRINIDAD AND TOBAGO: Trinidad. UNITED STATES: Arizona, Texas. VENEZUELA: Aragua, Capital District, Carabobo, Mérida.

#### References.

Burmeister 1847, Reiche 1859, Harold 1869b, Bates 1888, Casey 1915, Bodkin 1919, Arrow 1937b, Blackwelder 1944, Guimarães 1944, Howden 1955, Carrillo-S. et al. 1966, Gruner 1971, Pike et al. 1976, Dechambre 1979a, Pava-O. et al. 1983, King 1984, Endrődi 1963, 1964, 1966, 1969a, b, 1985a, Maes and Tellez Robleto 1988, Hardy 1991, Pérez Dominguez 1991, Thomas 1993, Lobo and Morón 1993, Deloya and Morón 1994, Maes 1987, 1992, 1994, Morón 1994, Poole and Gentili 1996, Abraca and Quesada 1997, Ratcliffe and Morón 1997, Sanchez Soto 1997, Aragón-Garcia and Morón 2000, Marín Jarillo 2001, Navarrete-Heredia et al. 2001, Andreazze and da Silva Motta 2002, Ratcliffe 2002a, 2003, Restrepo et al. 2003, Pardo-Locarno et al. 2005a, Díaz Mederos et al. 2006, Neita-Moreno et al. 2006, Ratcliffe and Cave 2006, Múñoz-Hernández et al. 2008, Pacheco-F. et al. 2006, 2008, Útima and Vallejo 2008, García et al. 2009, Smith 2003, 2009, Stechauner-Rohringer and Pardo-Locarno 2010, Neita-Moreno 2011, Yanes-Gómez and Morón 2010, Ponchel 2011, Aragón-García et al. 2012, Krajcik 2005, 2012, Lugo et al. 2012, Breeschoten et al. 2013, García-López et al. 2013, Pardo-Locarno 2013, Rivera-Gasperín et al. 2013, Rivera-Gasperín and Morón 2013, Yepes-Rodriguez et al. 2013, Morón et al. 1985, 1988, 1998, 2014, Ratcliffe et al. 2013, 2015, López-García et al. 2015, Deloya et al. 1993, 2014a, b, 2016, Gasca-Álvarez and Deloya 2016, Ratcliffe and Cave 2017.

#### Remarks.

Endrődi (1966) reported a single *C. lunulata* specimen, without details, from New Mexico (USA). Major faunistic studies did not find further specimens from New Mexico (Ratcliffe and Cave 2017).

### Cyclocephala lurida coahuilae

Taxon classificationAnimaliaColeopteraScarabaeidae

Bates, 1888

Cyclocephala coahuilae Bates, 1888: 302 [original combination].
Ochrosidia (Ochrosidia) coahuilae (Bates) [new combination and new subgeneric classification by Casey 1915: 151].
Cyclocephala coahuilae Bates [revised combination and removal of subgeneric classification by Arrow 1937b: 8, 9].
Cyclocephala immaculata coahuilae Bates [new subspecific status by Endrődi 1966: 219].
Cyclocephala lurida coahuilae Bates [revised subspecific status by Endrődi 1985a: 107].

#### Types.

Type at BMNH (Endrődi 1966).

#### Distribution.

MEXICO: Chihuahua, Coahuila, Durango, Estado de México, Guanajuato, Hidalgo, Jalisco, Nuevo León, Puebla, Querétaro, San Luis Potosí, Sonora, Veracruz.

#### References.

Bates 1888, Casey 1915, Arrow 1937b, Blackwelder 1944, Pike et al. 1976, Endrődi 1966, 1985a, Ratcliffe and Morón 1997, Marín Jarillo 2001, Carrillo-Ruiz and Morón 2003, Díaz Mederos et al. 2006, Múñoz-Hernández et al. 2008, Smith 2003, 2009, Krajcik 2005, 2012, Morón and Márquez 2012, Ratcliffe et al. 2013, Ratcliffe and Cave 2017.

#### Remarks.

Endrődi (1966, 1985a) reported *C. lurida coahuilae* from Costa Rica (Cartago), Colombia (no details), and Brazil (no details). This species has not been recorded again from Costa Rica, and these data are considered erroneous (Ratcliffe 2003). *Cyclocephala lurida coahuilae* is apparently known only from Mexico in Central America (Ratcliffe et al. 2013), and the South American records are possibly incorrect.

### Cyclocephala lurida lurida

Taxon classificationAnimaliaColeopteraScarabaeidae

Bland, 1863

Cyclocephala lurida Bland, 1863: 354 [original combination].
Spilosota lurida (Bland) [new combination by Casey 1915: 131].
Cyclocephala lurida Bland [revised combination by Arrow 1937b: 8, 12].
Cyclocephala hirta LeConte [synonymy by Saylor 1945: 384].
Cyclocephala lurida Bland [revalidated species status by Endrődi 1985a: 107].
**syn.** Ochrosidia (Ochrosidia) pagana Casey, 1915: 148 [original combination].
Cyclocephala pagana (Casey) [new combination and removal of subgeneric classification by Arrow 1937b: 8, 14].
Cyclocephala immaculata (Olivier) [synonymy by Saylor 1945: 385].
Cyclocephala lurida lurida Bland [synonymy by Endrődi 1985a: 107].
**syn.** Ochrosidia (Ochrosidia) protenta Casey, 1915: 144 [original combination].
Cyclocephala protenta (Casey) [new combination and removal of subgeneric classification by Arrow 1937b: 8, 14].
Cyclocephala immaculata (Olivier) [synonymy by Saylor 1945: 385].
Cyclocephala lurida lurida Bland [synonymy by Endrődi 1985a: 107].
**syn.** Ochrosidia (Ochrosidia) rufifrons Casey, 1915: 145 [original combination].
Cyclocephala rufifrons (Casey) [new combination and removal of subgeneric classification by Arrow 1937b: 8, 15].
Cyclocephala immaculata (Olivier) [synonymy by Saylor 1945: 385].
Cyclocephala lurida lurida Bland [synonymy by Endrődi 1985a: 107].
**syn.** Ochrosidia (Ochrosidia) tenuicutis Casey, 1915: 146 [original combination].
Cyclocephala tenuicutis (Casey) [new combination and removal of subgeneric classification by Arrow 1937b: 8, 16].
Cyclocephala immaculata (Olivier) [synonymy by Saylor 1945: 385].
Cyclocephala lurida lurida Bland [synonymy by Endrődi 1985a: 107].

#### Types.

Endrődi (1966) speculated that the type(s) of *C. lurida* were at USNM.

#### Distribution.

CANADA: Ontario. UNITED STATES: Alabama, Arkansas, Connecticut, Delaware, District of Columbia, Florida, Georgia, Illinois, Indiana, Iowa, Kansas, Kentucky, Louisiana, Maine, Maryland, Massachusetts, Michigan, Minnesota, Mississippi, Missouri, Nebraska, New Jersey, North Carolina, Ohio, Oklahoma, Pennsylvania, South Carolina, Tennessee, Texas, Vermont, Virginia, West Virginia, Wisconsin.

#### References.

Bland 1863, Lederer 1864a, b, Gerstaecker 1865, Harold 1869b, Miller 1870, Henshaw 1885, Moffat 1890, Lucas 1896, 1899, Forbes 1890, 1891, 1894, 1896, 1907, Swenk 1911, 1913, Casey 1915, Davis 1916, Leng 1920, Jaynes and Gardner 1924, Hayes 1928, Arrow 1937b, Sanderson 1939, 1940, Saylor 1945, 1948, Peterson 1951, Dutky 1963, Roberts 1963a, b, Ritcher 1940a, b, 1944, 1958, 1966, App and Kerr 1969, Beard 1972, Woodruff 1973, Harris 1977, Arnaud 1978; Barrows and Gordh 1978, Warren and Potter 1983, Potter and Gordon 1984, Endrődi 1966, 1969b, 1985a, Tashiro 1987, Ratcliffe 1991, Crocker 1992a, b, Crocker et al. 1992, Haynes et al. 1992, Potter and Haynes 1993, Youngman et al. 1993, Rice 1994, Dougherty and Knapp 1994, Suggars Downing 1994, Crutchfield et al. 1995, Crutchfield and Potter 1994, 1995a, b, c, Crutchfield et al. 1995, Haynes and Potter 1995, Merchant and Crocker 1996, Poole and Gentili 1996, Wedin and Huff 1996, Peck and Thomas 1998, Potter 1980, 1981, 1982, 1983, 1995, 1998, Bauernfeind et al. 1999, Crocker et al. 1999, Parker et al. 1999, Vittum et al. 1999, Weinhold et al. 1999, Held et al. 2000a, b, c, d, Muegge et al. 2000, Potter et al. 1992, 1996, 2000, Flanders et al. 2000, Mankin et al. 2000, Bauernfeind 2001, Harpootlian 2001, Weinhold and Baxendale 2001, Zenger and Gibb 2001, Carstens et al. 2002, Muegge and Quigg 2002, Walker and Royer 2002, Riley and Wolfe 2003, Rogers et al. 2003, Zhang et al. 2003a, b, Anderson et al. 2004, Cranshaw 2004, Hatch and White 2004, Rogers and Potter 2004a, b, c, Daly and Buntin 2005, Grewal et al. 2005, Kriska and Young 2005, Royer and Walker 2005a, b, Buss 2006, Eickhoff et al. 2005, 2006, Jackson and Klein 2006, Bixby et al. 2007, Toda et al. 2006, 2007, Dingman 2008, Kuldau and Bacon 2008, Koppenhöfer 2008, Pierson et al. 2008, Ratcliffe and Paulsen 2008, Schaeffer et al. 2008, Su et al. 2008, Counts and Hasiotis 2009, Held and Ray 2009, Popay 2009, Rebek et al. 2009a, b, Samples et al. 2009, Smith 2003, 2009, Royer et al. 2009, Baxendale et al. 2003, 2005, 2010, Caceres et al. 2010, Heng-Moss et al. 2005, 2010, Mashtoly et al. 2009, 2010, Ramm et al. 2010, Redmond and Potter 2010, Barden et al. 2011, Krajcik 2005, 2012, Lewis et al. 2012, Redmond et al. 2012a, b, Brill et al. 2013, Koppenhöfer et al. 2004, 2013, Rebek 2013, Williamson et al. 2004, 2008, 2013, Young et al. 2013, Wu et al. 2014, Behle et al. 2015, Chong and Hinson 2015, Stamm et al. 2008a, b, 2009, 2012, 2013, 2015, Behle and Goett 2016, Christians et al. 2016, Gyawaly et al. 2015, 2016, Koppenhöfer and Wu 2016, Reynolds et al. 2016, Buss and Dale 2017, Ratcliffe and Cave 2017.

#### Remarks.

Ratcliffe et al. (2013) listed all the Casey synonyms under *C. lurida coahuilae* Bates. It is more appropriate to treat these as synonyms of the nominate subspecies as all the Casey names were applied to species described from the United States (Alabama, Kansas, Louisiana, Missouri, Oklahoma, Texas) (Casey 1915).

### Cyclocephala lutea

Taxon classificationAnimaliaColeopteraScarabaeidae

Endrődi, 1966

Cyclocephala lutea Endrődi, 1966: 92, 141, 244 [original combination].

#### Types.

Holotype ♂ at HNHM (Endrődi Collection) (Endrődi 1966).

#### Distribution.

ARGENTINA: Chaco, Córdoba, Mendoza. BOLIVIA: Potosí. BRAZIL: Pará. CHILE: Atacama.

#### References.

Martínez 1968a, Pike et al. 1976, Vidal et al. 1979, Endrődi 1966, 1985a, Krajcik 2005, 2012, Breeschoten et al. 2013.

### Cyclocephala machadoi

Taxon classificationAnimaliaColeopteraScarabaeidae

Grossi, Santos, & Almeida, 2016

Cyclocephala machadoi Grossi, Santos, & Almeida, 2016: 246–247 [original combination].

#### Types.

Holotype ♂ at CERPE (Grossi et al. 2016).

#### Distribution.

BRAZIL: Minas Gerais.

#### References.

Grossi et al. 2016.

### Cyclocephala macrophylla

Taxon classificationAnimaliaColeopteraScarabaeidae

Erichson, 1847

Cyclocephala macrophylla Erichson, 1847a: 97 [original combination].

#### Types.

Lectotype ♂ at ZMHB (Endrődi 1966).

#### Distribution.

BOLIVIA: Chapare. COLOMBIA: Bolívar, Chocó, Meta, Tolima, Valle del Cauca. COSTA RICA: Alajuela, Cartago, Guanacaste, Heredia, Limón, Puntarenas, San José. ECUADOR: Guayas. GUYANA: Demerara-Mahaica. PANAMA: Bocas del Toro, Chiriquí, Coclé, Former Canal Zone, Panamá, San Blas. PERU: Ayacucho, Callao, La Libertad, Lima, Madre de Dios, Piura.

#### References.

Erichson 1847a, Harold 1869b, Arrow 1937b, Blackwelder 1944, Pike et al. 1976, Dechambre 1980, Endrődi 1966, 1985a, Dupuis 1996, Ratcliffe 1992a, 2002a, 2003, Neita-Moreno 2011, Krajcik 2005, 2012, Ratcliffe et al. 2015, Gasca-Álvarez and Deloya 2016.

#### Remarks.

*Cyclocephala macrophylla* was reported from Guadeloupe (La Désirade) (Endrődi 1966, 1985a). This species has not been reported from the Antilles since these records (e. g., see Ratcliffe and Cave 2015). It is possible that this record was based on misidentified specimens of the similar species *C. melanocephala*, which occurs on Guadeloupe.

### Cyclocephala maculata

Taxon classificationAnimaliaColeopteraScarabaeidae

Burmeister, 1847

Cyclocephala maculata Burmeister, 1847: 40 [original combination].
Mimeoma maculata (Burmeister) [new combination by Casey 1915: 111, 128].
Cyclocephala maculata Burmeister [revised combination by Moore et al. 2015: 898].
**syn.** Cyclocephala hielkemaorum Ratcliffe, 2008: 231–234 [original combination].
Mimeoma maculata (Burmeister) [synonymy by Ponchel 2010: 172].

#### Types.

Lectotype ♂ of *C. maculata* at MLUH (Endrődi 1966). Holotype ♂ of *C. hielkemaorum* at UNSM (Ratcliffe 2008).

#### Distribution.

BRAZIL: Amazonas. COLOMBIA. FRENCH GUIANA: Cayenne, St.-Laurent du Maroni. GUYANA.

#### References.

Burmeister 1847, Harold 1869b, Casey 1915, Arrow 1937b, Blackwelder 1944, Pike et al. 1976, Endrődi 1966, 1985a, Restrepo et al. 2003, Krajcik 2005, 2012, Breeschoten et al. 2013, Moore et al. 2015, Ponchel 2006, 2010, 2011, 2015, Gasca-Álvarez and Deloya 2016.

### Cyclocephala maculiventris

Taxon classificationAnimaliaColeopteraScarabaeidae

Höhne, 1923

Cyclocephala maculiventris Höhne, 1923b: 345–346 [original combination].
**syn.** Cyclocephala warneri Ratcliffe, 1992c: 250–253 [original combination].
Cyclocephala maculiventris Höhne [synonymy by Ratcliffe et al. 2013: 186].

#### Types.

Lectotype ♂ of *C. maculiventris* at ZMHB (Endrődi 1966). Holotype ♂ of *C. warneri* at UNSM (Ratcliffe 1992c).

#### Distribution.

BELIZE: Toledo. COSTA RICA: Alajuela, Cartago, Guanacaste, Heredia, Limón, Puntarenas, San José. GUATEMALA: Alta Verapaz, Huehuetenango. HONDURAS: Colón, Yoro. MEXICO: Chiapas, Veracruz. NICARAGUA: Jinotega.

#### References.

Höhne 1923b, Arrow 1937b, Blackwelder 1944, Pike et al. 1976, Endrődi 1963, 1966, 1985a, Thomas 1993, Lobo and Morón 1993, Ratcliffe and Morón 1997, Ratcliffe 1992c, 2003, Ratcliffe and Cave 2006, Krajcik 2005, 2012, Ratcliffe et al. 2013, Romero-López and Morón 2017.

### Cyclocephala mafaffa grandis

Taxon classificationAnimaliaColeopteraScarabaeidae

Burmeister, 1847

Cyclocephala grandis Burmeister, 1847: 69 [original combination].
Cyclocephala mafaffa ab. grandis Burmeister [new infrasubspecific status by Endrődi 1964: 466].
Cyclocephala mafaffa grandis Burmeister [new subspecific status by Chalumeau 1982: 336].
Cyclocephala mafaffa ab. grandis Burmeister [revalidated infrasubspecific status by Endrődi 1985a: 87].
Cyclocephala mafaffa Burmeister [synonymy by Ratcliffe 2002a: 29].
Cyclocephala mafaffa grandis Burmeister [revalidated subspecific status by Ratcliffe and Cave 2015].

#### Types.

Endrődi (1966) did not find the type material of *C. grandis*. Neotype of *C. mafaffa grandis* at USNM (Chalumeau 1982).

#### Distribution.

GUADELOUPE: Basse-Terre, Grande-Terre. MONTSERRAT: Saint Georges, Saint Peter. SABA. ST. KITTS AND NEVIS: St. Kitts.

#### References.

Dejean 1833, 1836b, Burmeister 1847, Harold 1869b, Fleutiaux and Sallé 1889, Arrow 1937b, Blackwelder 1944, Paulian 1947, Pike et al. 1976, Chalumeau and Gruner 1977, Chalumeau 1982, Endrődi 1966, 1985a, Ratcliffe 2002a, 2003, Dutrillaux et al. 2007, Ivie et al. 2008, Krajcik 2005, 2012, Dutrillaux and Dutrillaux 2013, Ratcliffe et al. 2013, Dutrillaux et al. 2013, 2014, Gillett and Gillett 2015, Ratcliffe and Cave, 2006, 2015.

### Cyclocephala mafaffa mafaffa

Taxon classificationAnimaliaColeopteraScarabaeidae

Burmeister, 1847

Cyclocephala mafaffa Burmeister, 1847: 69 [original combination].
Stigmalia mafaffa (Burmeister) [new combination by Casey 1915: 119].
Cyclocephala mafaffa Burmeister [revised combination by Arrow 1937b: 8, 12].
**syn.** Stigmalia mafaffa histrionica Casey, 1915: 119 [original combination].
Cyclocephala mafaffa Burmeister [synonymy by Arrow 1937b: 12].

#### Types.

Lectotype ♂ of *C. mafaffa* at MLUH (Endrődi 1966). Type material of *Stigmalia mafaffa histrionica* at USNM (Endrődi 1966).

#### Distribution.

BELIZE: Cayo, Stann Creek, Toledo. BRAZIL: Amazonas. COLOMBIA: Antioquia, Boyacá, Cesar, Chocó, Cundinamarca, Magdalena, Nariño, Risaralda, Valle del Cauca. COSTA RICA: Alajuela, Cartago, Guanacaste, Heredia, Limón, Puntarenas, San José. ECUADOR: Cañar, Napo. EL SALVADOR: Ahuachapán, Chalatenango, La Libertad, Morazán, San Salvador, Santa Ana, Usulután. GUATEMALA: Alta Verapaz, Baja Verapaz, Chiquimula, El Progreso, Escuintla, Guatemala, Huehuetenango, Izabal, Jalapa, Jutiapa, Petén, Quetzaltenango, Quiché, Sacatepéquez, San Marcos, Santa Rosa, Sololá, Suchitepéquez, Zacapa. HONDURAS: Atlántida, Choluteca, Comayagua, Copán, Cortés, El Paraíso, Francisco Morazán, La Paz, Ocotepeque, Olancho, Santa Bárbara, Yoro. MEXICO: Aguascalientes, Chiapas, Colima, Durango, Estado de México, Guerrero, Hidalgo, Jalisco, Michoacán, Morelos, Nayarit, Oaxaca, Puebla, San Luis Potosí, Sinaloa, Sonora, Tamaulipas, Veracruz. NICARAGUA: Chontales, Estelí, Granada, Jinotega, Managua, Nueva Segovia, RAA Norte. PANAMA: Bocas del Toro, Chiriquí, Coclé, Colón, Darien, Former Canal Zone, Panamá. TRINIDAD AND TOBAGO: Trinidad. VENEZUELA: Carabobo.

#### References.

Burmeister 1847, Harold 1869b, Bates 1888, Leng and Mutchler 1914, Casey 1915, Arrow 1937b, Blackwelder 1944, Carrillo-S. et al. 1966, Barrera 1969, Pike et al. 1976, Dechambre 1979a, Endrődi 1964, 1966, 1985a, Maes and Téllez Robleto 1988, Valerio 1988, Ratcliffe and Delgado-Castillo 1990, Murray 1993, Thomas 1993, Lobo and Morón 1993, Maes 1987, 1994, Poole and Gentili 1996, Croat 1997, Morón 1979, 1994, 1997, Ratcliffe and Morón 1997, Navarrete-Heredia et al. 2001, García-Luna et al. 2002, Carrillo-Ruiz and Morón 2003, Ratcliffe 1992a, 2002a, 2003, Restrepo et al. 2003, Ponchel 2006, Luna et al. 2007, Pacheco-F. et al. 2008, Útima and Vallejo 2008, Neita-Moreno and Gaigl 2008, Smith 2003, 2009, Neita-Moreno 2011, Krajcik 2005, 2012, García-López et al. 2013, Moore and Jameson 2013, Ratcliffe et al. 2013, Giannoulis et al. 2012, Yepes-Rodriguez et al. 2013, López-García et al. 2015, Ratcliffe and Cave 2006, 2015, 2017, Deloya et al. 1993, 2014a, 2016, Gasca-Álvarez and Deloya 2016.

### Cyclocephala magdalenae

Taxon classificationAnimaliaColeopteraScarabaeidae

Young & Le Tirant, 2005

Cyclocephala magdalenae Young & Le Tirant, 2005: 267–270.

#### Types.

Holotype ♂ at IMQC (Young and Le Tirant 2005).

#### Distribution.

COLOMBIA: Huila.

#### References.

Young and Le Tirant 2005, Krajcik 2012, Gasca-Álvarez and Deloya 2016.

### Cyclocephala malleri

Taxon classificationAnimaliaColeopteraScarabaeidae

Martínez, 1968

Cyclocephala malleri Martínez, 1968c: 81–84 [original combination].

#### Types.

Holotype ♂ at MACN (Antonio Martínez Collection) (Martínez 1968c).

#### Distribution.

BRAZIL: Mato Grosso.

#### References.

Martínez 1968c, Pike et al. 1976, Endrődi 1985a, Krajcik 2005, 2012.

### Cyclocephala malyi

Taxon classificationAnimaliaColeopteraScarabaeidae

Dupuis, 2014

Cyclocephala malyi Dupuis, 2014: 52–54 [original combination].

#### Types.

Holotype ♂ in Pokorny Collection (Prague, Czech Republic) (Dupuis 2014).

#### Distribution.

ECUADOR: Pastaza.

#### References.

Dupuis 2014.

### Cyclocephala mannheimsi

Taxon classificationAnimaliaColeopteraScarabaeidae

Endrődi, 1964

Cyclocephala mannheimsi Endrődi, 1964: 444–445 [original combination].

#### Types.

Holotype ♂ at USNM (Endrődi 1964).

#### Distribution.

BOLIVIA: Santa Cruz. COLOMBIA: Amazonas, Caquetá, Nariño. ECUADOR: Pichincha. PERU.

#### References.

Pike et al. 1976, Endrődi 1964, 1966, 1985a, Pardo-Locarno et al. 2011, Krajcik 2005, 2012, Gasca-Álvarez 2014, Ratcliffe et al. 2015, Gasca-Álvarez and Deloya 2016.

### Cyclocephala marginalis

Taxon classificationAnimaliaColeopteraScarabaeidae

Kirsch, 1870

Cyclocephala marginalis Kirsch, 1870: 356–357 [original combination].
**syn.** Cyclocephala cincta Prell, 1937b: 496 [original combination].
Cyclocephala marginalis ab. cincta Prell [new infrasubspecific status by Endrődi 1966: 249].
**syn.** Cyclocephala intermissa Prell, 1937b: 496 [original combination].
Cyclocephala marginalis ab. intermissa Prell [new infrasubspecific status by Endrődi 1966: 249].

#### Types.

Holotype ♀ of *C. marginalis* at MTD (Endrődi 1966). Types of *C. intermissa* and *C. cincta* at ZMHB (Endrődi 1966).

#### Distribution.

BOLIVIA: Cochabamba. BRAZIL: Amazonas, Pará, Rio de Janeiro, Rio Grande do Sul, Santa Catarina, São Paulo. COLOMBIA: Meta. FRENCH GUIANA: St.-Élie, St.-Laurent du Maroni. GUYANA: East Berbice-Corentyne. PERU.

#### References.

Kirsch 1870, Arrow 1937b, Prell 1937b, Blackwelder 1944, Pike et al. 1976, Dechambre 1979a, Endrődi 1966, 1985a, Küchmeister et al. 1998, Silberbauer-Gottsberger et al. 2001, Núñez-Avellaneda and Neita-Moreno 2009, Ponchel 2011, Krajcik 2005, 2012, Ratcliffe et al. 2015, Gasca-Álvarez and Deloya 2016.

### Cyclocephala marginicollis

Taxon classificationAnimaliaColeopteraScarabaeidae

Arrow, 1902

Cyclocephala marginicollis Arrow, 1902: 138 [original combination].

#### Types.

Type at BMNH (Endrődi 1966).

#### Distribution.

MEXICO: Chiapas, Quintana Roo, Tabasco, Veracruz, Yucatán.

#### References.

Arrow 1902, 1937b, Blackwelder 1944, Pike et al. 1976, Endrődi 1964, 1966, 1985a, Morón 1979, Thomas 1993, Ratcliffe and Morón 1997, Krajcik 2005, 2012, Ratcliffe et al. 2013.

### Cyclocephala marianista

Taxon classificationAnimaliaColeopteraScarabaeidae

Dechambre & Endrődi, 1984

Cyclocephala marianista Dechambre & Endrődi, 1984: 170 [original combination].

#### Types.

Holotype ♂ at MNHN (Dechambre and Endrődi 1984).

#### Distribution.

ECUADOR: Napo.

#### References.

Dechambre and Endrődi 1984, Krajcik 2005, 2012.

### Cyclocephala marqueti

Taxon classificationAnimaliaColeopteraScarabaeidae

Dechambre, 1997

Cyclocephala marqueti Dechambre, 1997: 14, 22 [original combination].

#### Types.

Holotype ♂ at MNHN (Dechambre 1997).

#### Distribution.

ECUADOR: Napo.

#### References.

Dechambre 1997, Krajcik 2005, 2012.

### Cyclocephala martinezi

Taxon classificationAnimaliaColeopteraScarabaeidae

Endrődi, 1964

Cyclocephala martinezi Endrődi, 1964: 456–457 [original combination].

#### Types.

Holotype ♂ at HNHM (Endrődi Collection) (Endrődi 1964, Dupuis 2018).

#### Distribution.

COLOMBIA: Antioquia, Caldas, Meta. VENEZUELA: Apure, Bolívar.

#### References.

Pike et al. 1976, Endrődi 1964, 1966, 1985a, Restrepo et al. 2003, Krajcik 2005, 2012, Gasca-Álvarez and Deloya 2016, Dupuis 2018.

### Cyclocephala marylizae

Taxon classificationAnimaliaColeopteraScarabaeidae

Ratcliffe, 2003

Cyclocephala marylizae Ratcliffe, 2003: 61, 66, 71, 76, 157–161 [original combination].

#### Types.

Holotype ♂ at MNCR (originally deposited at INBio) (Ratcliffe 2003).

#### Distribution.

COSTA RICA: Puntarenas.

#### References.

Ratcliffe 2003, Krajcik 2005, 2012.

### Cyclocephala mathani

Taxon classificationAnimaliaColeopteraScarabaeidae

Dechambre, 1982

Cyclocephala mathani Dechambre, 1982: 1–2 [original combination].

#### Types.

Holotype ♂ at MNHN (Dechambre 1982).

#### Distribution.

ECUADOR: Bolívar, Napo. PERU.

#### References.

Dechambre 1982, 1992, Krajcik 2005, 2012.

### Cyclocephala mechae

Taxon classificationAnimaliaColeopteraScarabaeidae

Martínez, 1978

Cyclocephala mechae Martínez, 1978a: 5–8 [original combination].

#### Types.

Holotype ♂ at MACN (Antonio Martínez Collection) (Martínez 1978a).

#### Distribution.

PARAGUAY: Misiones.

#### References.

Martínez 1978a, Endrődi 1985a, Krajcik 2005, 2012.

### Cyclocephala mecynotarsis

Taxon classificationAnimaliaColeopteraScarabaeidae

Höhne, 1923

Cyclocephala mecynotarsis Höhne, 1923b: 351–354 [original combination].

#### Types.

Lectotype ♂ (Endrődi 1966). Endrődi did not state clearly where the lectotype was deposited. It may be at ZMHB.

#### Distribution.

BRAZIL: Amazonas, Distrito Federal, Mato Grosso. PARAGUAY. PERU: Ayacucho, Cusco. VENEZUELA (Höhne 1923b, Endrődi 1966, 1985a).

#### References.

Höhne 1923b, Arrow 1937b, Blackwelder 1944, Martínez 1968b, Pike et al. 1976, Endrődi 1964, 1966, 1985a, Scariot et al. 1991, Andreazze and Fonseca 1998, Gottsberger and Silberbauer-Gottsberger 2006, Krajcik 2005, 2012, Breeschoten et al. 2013, Ratcliffe et al. 2015.

### Cyclocephala megalophylla

Taxon classificationAnimaliaColeopteraScarabaeidae

Endrődi, 1966

Cyclocephala megalophylla Endrődi, 1966: 89, 142, 253–254 [original combination].

#### Types.

Holotype ♂ at ZMHB (Endrődi 1966).

#### Distribution.

ARGENTINA: Santa Fe.

#### References.

Pike et al. 1976, Endrődi 1966, 1985a, Krajcik 2005, 2012.

### Cyclocephala melane

Taxon classificationAnimaliaColeopteraScarabaeidae

Bates, 1888

Cyclocephala melane Bates, 1888: 310 [original combination].

#### Types.

Type at BMNH (Endrődi 1966).

#### Distribution.

COSTA RICA: Cartago, Heredia, Puntarenas, San José. PANAMA: Chiriquí.

#### References.

Bates 1888, Arrow 1937b, Blackwelder 1944, Pike et al. 1976, Endrődi 1966, 1985a, Croat 1997, Ratcliffe 1992a, 2002a, 2003, Krajcik 2005, 2012, Moore and Jameson 2013.

#### Remarks.

The original spelling of this specific epithet is “*melane*” (Bates 1888). Subsequently, some authors have spelled the name as “*melanae*” without explanation (Endrődi 1966, 1985a, Ratcliffe 1992a, 2002a, 2003, Croat 1997, Moore and Jameson 2013).

### Cyclocephala melanocephala

Taxon classificationAnimaliaColeopteraScarabaeidae

(Fabricius, 1775)

Melolontha melanocephala Fabricius, 1775: 36 [original combination].
Cyclocephala melanocephala (Fabricius) [new combination by Burmeister 1847: 56–57].
Dichromina melanocephala (Fabricius) [new combination by Casey 1915: 160].
Cyclocephala melanocephala (Fabricius) [revised combination by Arrow 1937b: 8, 13].
**syn.** Chalepus leucophthalmus Fischer, 1823: 265 [original combination].
Dyscinetus leucophthalmus (Fischer) [new combination by Harold 1869a: 123].
Cyclocephala melanocephala (Fabricius) [synonymy by Arrow 1911: 172].
**syn.** Cyclocephala dimidiata Burmeister, 1847: 57 [original combination].
Dichromina dimidiata (Burmeister) [new combination by Casey 1915: 161].
Cyclocephala (Dichromina) dimidiata (Burmeister) [revised combination and new subgeneric classification by Saylor 1937: 70].
Cyclocephala dimidiata Burmeister [removal of subgeneric classification by Arrow 1937b: 8, 10].
Cyclocephala melanocephala (Fabricius) [synonymy by Endrődi 1964: 466].
**syn.** Cyclocephala elegans Horn, 1871: 337 [original combination].
Cyclocephala dimidiata Burmeister [synonymy by Horn 1875: 143].
Dichromina elegans (Horn) [revalidated species status and new combination by Casey 1915: 162].
Cyclocephala dimidiata Burmeister [synonymy by Saylor 1945: 382].
Cyclocephala melanocephala (Fabricius) [synonymy by Endrődi 1964: 466].
**syn.** Cyclocephala rubiginosa Burmeister, 1847: 59 [original combination].
Cyclocephala melanocephala rubiginosa Burmeister [new subspecific status by Chalumeau and Gruner 1977: 584].
Cyclocephala melanocephala (Fabricius) [synonymy by Ratcliffe and Cave 2015].
**syn.** Cyclocephala ventralis Erichson, 1847a: 97 [original combination].
Cyclocephala melanocephala (Fabricius) [synonymy by Endrődi 1964: 466].
**syn.** Dichromina ocularis Casey, 1915: 162 [original combination].
Cyclocephala ocularis (Casey) [new combination by Arrow 1937b: 8, 14].
Cyclocephala dimidiata Burmeister [synonymy by Saylor 1945: 382].

#### Types.

Type of *M. melanocephala* at BMNH (Banks Collection) (Endrődi 1966). Lectotype of *C. ventralis* at ZMHB (Endrődi 1966). Type of *C. dimidiata* is missing (Endrődi 1966). The type of *Cyclocephala leucophthalmus* is unknown (Endrődi 1966). Type of *D. ocularis* at USNM (Endrődi 1966). Type of *C. elegans* at USNM (Endrődi 1966). The type of *C. rubiginosa* is missing (Endrődi 1966).

#### Distribution.

ARGENTINA: Córdoba, Salta, Santa Fe, Tucumán. BELIZE: Cayo, Orange Walk, Stann Creek, Toledo. BOLIVIA: Beni, Cochabamba, Santa Cruz. BRAZIL: Amazonas, Bahia, Ceará, Espírito Santo, Goiás, Mato Grosso, Mato Grosso do Sul, Minas Gerais, Paraná, Pernambuco, Rio de Janeiro, Rio Grande do Norte, Rio Grande do Sul, Roraima, Santa Catarina, São Paulo. COLOMBIA: Antioquia, Atlántico, Boyacá, Casanare, Cauca, Cesar, Chocó, Córdoba, Cundinamarca, Huila, La Guajira, Meta, Risaralda, Santander, Tolima, Valle del Cauca. COSTA RICA: Alajuela, Cartago, Guanacaste, Heredia, Limón, Puntarenas, San José. DOMINICA: St. David, St. Joseph, St. Patrick, St. Paul. DOMINICAN REPUBLIC: Barahona. ECUADOR: Bolívar, Cañar, Esmeraldas, Guayas, Loja, Los Ríos, Morona-Santiago, Santa Elena. EL SALVADOR: Ahuachapán, Chalatenango, La Libertad, Morazán, Santa Ana. FRENCH GUIANA: Cayenne. GRENADA: St. Andrew, St. David, St. George, St. John. GRENADINES: Bequia, Canouan, Carriacou, Union Island. GUADELOUPE: Basse-Terre, Îles des Saintes, Marie-Galante. GUATEMALA: Alta Verapaz, Baja Verapaz, Chiquimula, El Progreso, Escuintla, Guatemala, Huehuetenango, Izabal, Petén, Quetzaltenango, Quiché, Retalhuleu, San Marcos, Santa Rosa, Sololá, Suchitepéquez, Zacapa. GUYANA: Cuyuni-Mazaruni, East Berbice-Corentyne, Pomeroon-Supenaam. HONDURAS: Atlántida, Choluteca, Comayagua, Cortés, El Paraíso, Francisco Morazán, Gracias a Dios, Intibucá, Olancho, Santa Bárbara, Yoro. MARTINIQUE: Fort-de-France, La Trinité, Le Marigot, Le Marin, Saint-Pierre. MEXICO: Aguascalientes, Baja California, Baja California Sur, Chiapas, Chihuahua, Distrito Federal, Colima, Durango, Guerrero, Hidalgo, Jalisco, Michoacán, Morelos, Nayarit, Nuevo León, Oaxaca, Puebla, Querétaro, Quintana Roo, San Luis Potosí, Sinaloa, Sonora, Tabasco, Tamaulipas, Veracruz, Yucatán, Zacatecas. MONTSERRAT. NICARAGUA: Carazo, Chinandega, Granada, Jinotega, León, Matagalpa, RAA Norte, RAA Sur, Río San Juan, Rivas. PANAMA: Bocas del Toro, Chiriquí, Coclé, Colón, Darien, Former Canal Zone, Panamá, San Blas, Veraguas. PARAGUAY: Asunción, Paraguarí. PERU: Ayacucho, Callao, Cusco, Junín, La Libertad, Madre de Dios, Pasco. PUERTO RICO. SAINT BARTHÉLEMY. SURINAME: Marowjine. TRINIDAD AND TOBAGO: Trinidad. UNITED STATES: Arizona, Arkansas, California, Kansas, Louisiana, Mississippi, Nevada, New Mexico, Oklahoma, Texas, Utah. VENEZUELA: Bolívar, Capital District, Carabobo, Mérida, Zulia.

#### References.

Fabricius 1775, 1801, Herbst 1790, Illiger 1802a, 1805, Dejean 1833, 1836b, Sturm 1843, Erichson 1847a, Burmeister 1847, Harold 1869b, Bertkau 1873, Crotch 1873, Austin 1880, Henshaw 1885, Bates 1888, Cockerell 1897, Horn 1871, 1894, Fall 1901, Fall and Cockerell 1907, Ohaus 1910, Leng and Mutchler 1914, Casey 1915, Bodkin 1919, Leng 1920, Arrow 1900, 1911, 1937b, Moore 1937, Guimarães 1944, Saylor 1937, 1945, 1948, Sanderson 1940, Blackwelder 1939, 1944, Paulian 1947, Blackwelder and Blackwelder 1948, Mariconi 1959, Linsley 1960, Zimsen 1964, Carrillo-S. et al. 1966, Martínez 1968a, Squire 1972, Ritcher and Baker 1974, Pike et al. 1976, Chalumeau and Gruner 1977, Cartwright and Chalumeau 1978, Chalumeau 1982, 1983, Boiça et al. 1984, Endrődi 1964, 1966, 1973a, 1985a, Ungaro et al. 1985, Gottsberger 1986, Morón et al. 1985, 1988, Sutton 1988, Dechambre 1992, Rogers 1992, Murray 1993, Thomas 1993, Lobo and Morón 1993, Deloya and Morón 1994, Morón 1979, 1994, Dupuis 1996, Poole and Gentili 1996, Charlet et al. 1997, Ratcliffe and Morón 1997, Sanchez Soto 1997, Woodruff et al. 1998, Bauernfeind 2001, Navarrete-Heredia et al. 2001, DeFoliart 2002, Peck et al. 2002, Carrillo-Ruiz and Morón 2003, Joly 2003, Luna et al. 2003, Marquet and Roquet 2003, Raguso et al. 2003, Riley and Wolfe 2003, Ratcliffe 1992c, 2002a, 2003, Restrepo et al. 2003, Pardo-Locarno et al. 2005a, Neita-Moreno et al. 2006, Ponchel 2006, Pacheco-F. et al. 2008, Múñoz-Hernández et al. 2008, Útima and Vallejo 2008, Barbosa Moreira et al. 2009, Smith 2003, 2009, Yanes-Gómez and Morón 2010, Neita-Moreno 2011, Giannoulis et al. 2012, Hay et al. 2012, Krajcik 2005, 2012, Breeschoten et al. 2013, García-López et al. 2013, Lima Nogueira et al. 2013, Pardo-Locarno 2013, Yepes-Rodriguez et al. 2013, Dutrillaux et al. 2007, 2014, Alberio et al. 2015, García-Atencia and Martínez-Hernández 2015, López-García et al. 2015, Ratcliffe et al. 2013, 2015, Deloya et al. 2014a, b, 2016, Dossey et al. 2016, Gasca-Álvarez and Deloya 2016, Mitasuhashi 2016, Peck 2016, Schrader et al. 2016, Ratcliffe and Cave 2006, 2015, 2017, Romero-López and Morón 2017.

### Cyclocephala melanopoda

Taxon classificationAnimaliaColeopteraScarabaeidae

Ratcliffe, 2008

Cyclocephala melanopoda Ratcliffe, 2008: 234–236 [original combination].

#### Types.

Holotype ♂ at UNSM (Ratcliffe 2008).

#### Distribution.

ECUADOR: Pichincha, Santo Domingo de los Tsáchilas.

#### References.

Ratcliffe 2008, Krajcik 2012.

### Cyclocephala melolonthida

Taxon classificationAnimaliaColeopteraScarabaeidae

Ratcliffe & Cave, 2002

Cyclocephala melolonthida Ratcliffe & Cave, 2002: 153–155 [original combination].

#### Types.

Holotype ♂ at UNSM (Ratcliffe and Cave 2002).

#### Distribution.

EL SALVADOR: La Paz. GUATEMALA: Escuintla, Santa Rosa.

#### References.

Ratcliffe and Cave 2002, 2006, Krajcik 2005, 2012, Ratcliffe et al. 2013.

### Cyclocephala mesophylla

Taxon classificationAnimaliaColeopteraScarabaeidae

Mora-Aguilar & Delgado, 2012

Cyclocephala mesophylla Mora-Aguilar & Delgado, 2012: 139–141 [original combination].

#### Types.

Holotype ♂ at IEXA (Mora-Aguilar and Delgado 2012).

#### Distribution.

MEXICO: Chiapas, Oaxaca, Veracruz.

#### References.

Mora-Aguilar and Delgado 2012, Ratcliffe et al. 2013.

### Cyclocephala metrica

Taxon classificationAnimaliaColeopteraScarabaeidae

Steinheil, 1874

Cyclocephala metrica Steinheil, 1874: 559–560 [original combination].
**syn.** Cyclocephala parvula Berg, 1881a: 100 [original combination].
Cyclocephala metrica Steinheil [synonymy by Arrow 1937b: 13].

#### Types.

Type of *C. metrica* at MNHN (Endrődi 1966). The location of the type(s) of *C. parvula* is unknown (Endrődi 1966).

#### Distribution.

ARGENTINA: Buenos Aires, Córdoba, La Pampa, Salta, San Luis, Tucumán. BRAZIL: Rio Grande do Sul. URUGUAY: Canelones, Maldonado, Montevideo.

#### References.

Steinheil 1874, Berg 1881a, b, Anonymous 1881, 1886, Kolbe 1907, Arrow 1937b, Blackwelder 1944, Hayward 1946, Pike et al. 1976, Endrődi 1966, 1985a, Saenz and Morelli 1984, Krajcik 2005, 2012, Breeschoten et al. 2013, Cherman et al. 2013.

### Cyclocephala miamiensis

Taxon classificationAnimaliaColeopteraScarabaeidae

Howden & Endrődi, 1966

Cyclocephala miamiensis Howden & Endrődi, 1966: 295–296 [original combination].

#### Types.

Holotype ♂ at CNC (Howden and Endrődi 1966).

#### Distribution.

UNITED STATES: Florida.

#### References.

Howden and Endrődi 1966, Woodruff 1973, Pike et al. 1976, Endrődi 1985a, Hardy 1991, Poole and Gentili 1996, Peck and Thomas 1998, Smith 2003, 2009, Krajcik 2005, 2012, Ratcliffe and Cave 2017.

### Cyclocephala minuchae

Taxon classificationAnimaliaColeopteraScarabaeidae

Joly, 2003

Cyclocephala minuchae Joly, 2003: 38–40 [original combination].

#### Types.

Holotype ♂ at MIZA (Joly 2003).

#### Distribution.

VENEZUELA: Anzoátegui, Aragua, Falcón, Miranda.

#### References.

Joly 2003, Krajcik 2005, 2012.

### Cyclocephala minuta

Taxon classificationAnimaliaColeopteraScarabaeidae

Burmeister, 1847

Cyclocephala minuta Burmeister, 1847: 59 [original combination].

#### Types.

Lectotype ♂ at MLUH (Endrődi 1966).

#### Distribution.

FRENCH GUIANA: Cayenne. GUYANA: Pomeroon-Supenaam. PARAGUAY: Paraguarí. SURINAME: Para. VENEZUELA.

#### References.

Burmeister 1847, Harold 1869b, Arrow 1937b, Blackwelder 1944, Pike et al. 1976, Dechambre 1980, Endrődi 1966, 1985a, Gruner 1971, Ponchel 2011, Krajcik 2005, 2012, Breeschoten et al. 2013.

### Cyclocephala modesta

Taxon classificationAnimaliaColeopteraScarabaeidae

Burmeister, 1847

Cyclocephala modesta Burmeister, 1847: 38 [original combination].

#### Types.

Lectotype ♀ at MLUH (Endrődi 1966).

#### Distribution.

ARGENTINA: Buenos Aires, Catamarca, Chaco, Córdoba, Mendoza, Misiones, Salta, Santa Fe, Tucumán. BOLIVIA: Cochabamba. BRAZIL: Bahia, Espírito Santo, Mato Grosso do Sul, Pará, Rio de Janeiro, Rio Grande do Sul, Santa Catarina, São Paulo. CHILE: Santiago Metropolitan Region. PARAGUAY: Guairá. SURINAME. URUGUAY: Canelones, Maldonado, Montevideo, Paysandú, Rivera, San José, Soriano, Tacuarembó.

#### References.

Burmeister 1847, Harold 1869b, Tremoleras 1910, Arrow 1937b, Blackwelder 1944, Martínez 1954, Pike et al. 1976, Alvarado 1980, Saenz and Morelli 1984, Endrődi 1966, 1969a, 1985a, Achinelly and Camino 2008, Mondaca 2011, Krajcik 2005, 2012, Breeschoten et al. 2013, Cherman et al. 2013, Crespo and Castelo 2013, Barrantes and Castelo 2014, Salgado-Neto et al. 2016.

### Cyclocephala molesta

Taxon classificationAnimaliaColeopteraScarabaeidae

Endrődi, 1969

Cyclocephala molesta Endrődi, 1969b: 35–36 [original combination].

#### Types.

Holotype ♂ at “Pereira Collection in Sao Paulo” (Endrődi 1969b). This is possibly referring to MZSP.

#### Distribution.

BOLIVIA: Cochabamba. BRAZIL: Pará. PERU.

#### References.

Pike et al. 1976, Dechambre 1979a, Endrődi 1969b, 1985a, Krajcik 2005, 2012, Ratcliffe et al. 2015.

### Cyclocephala monacha

Taxon classificationAnimaliaColeopteraScarabaeidae

Ratcliffe, 2008

Cyclocephala monacha Ratcliffe, 2008: 236–237 [original combination].

#### Types.

Holotype ♂ at UNSM (Ratcliffe 2008).

#### Distribution.

COLOMBIA: Amazonas, Boyacá.

#### References.

Ratcliffe 2008, Krajcik 2012, Gasca-Álvarez 2013, López-García et al. 2015, Gasca-Álvarez and Deloya 2016.

### Cyclocephala monzoni

Taxon classificationAnimaliaColeopteraScarabaeidae

Ratcliffe & Cave, 2009

Cyclocephala monzoni Ratcliffe & Cave, 2009: 328–332 [original combination].

#### Types.

Holotype ♂ at UVGC (Ratcliffe and Cave 2009).

#### Distribution.

GUATEMALA: Baja Verapaz, El Progreso, San Marcos, Zacapa.

#### References.

Ratcliffe and Cave 2009, Ratcliffe et al. 2013.

### Cyclocephala moreti

Taxon classificationAnimaliaColeopteraScarabaeidae

Dechambre, 1992

Cyclocephala moreti Dechambre, 1992: 68–70 [original combination].

#### Types.

Holotype ♂ at MNHN (Dechambre 1992).

#### Distribution.

ECUADOR: Napo. PERU.

#### References.

Dechambre 1992, Krajcik 2005, 2012, Ratcliffe et al. 2015.

### Cyclocephala morphoidina

Taxon classificationAnimaliaColeopteraScarabaeidae

Prell, 1937

Cyclocephala morphoidina Prell, 1937b: 495–496 [original combination].
Albridarollia morphoidina (Prell) [new combination by Bolivar et al. 1963: 185].
Cyclocephala morphoidina Prell [revised combination by Endrődi 1966: 61, 262].

#### Types.

Endrődi (1966) did not find the type(s) of *C. morphoidina* but he suspected that it was at ZMHB (Prell Collection).

#### Distribution.

COLOMBIA: Antioquia, Meta, Risaralda. BOLIVIA: La Paz. ECUADOR: Pastaza. PERU.

#### References.

Prell 1937b, Blackwelder 1944, Bolivar et al. 1963, Pike et al. 1976, Endrődi 1966, 1985a, Restrepo et al. 2003, Dupuis 2008, Útima and Vallejo 2008, Krajcik 2005, 2012, Yepes-Rodriguez et al. 2013, Ratcliffe et al. 2015, Gasca-Álvarez and Deloya 2016.

#### Remarks.

*Cyclocephala morphoidina* was previously reported from Mexico and Guatemala (Endrődi 1966, 1985a). These records are considered spurious and likely do not reflect permanent populations (Ratcliffe et al. 2013).

### Cyclocephala multiplex

Taxon classificationAnimaliaColeopteraScarabaeidae

Casey, 1915

Cyclocephala (Cyclocephala) multiplex Casey, 1915: 139 [original combination].
Cyclocephala detecta Bates [synonymy by Arrow 1937b: 10].
Cyclocephala amazona (Linneaus) [synonymy by Endrődi 1964: 466].
Cyclocephala multiplex Casey [revalidated species status by Ratcliffe 2003: 165].

#### Types.

Type of *C. multiplex* at USNM (Endrődi 1966).

#### Distribution.

BELIZE: Belize, Cayo, Stann Creek, Toledo. COSTA RICA: Alajuela, Guanacaste, Heredia. EL SALVADOR: La Libertad, Morazán, San Salvador, Santa Ana. GUATEMALA: Alta Verapaz, Baja Verapaz, Chiquimula, El Progresso, Huehuetenango, Izabal, Petén, Quiché, Santa Rosa, Zacapa. HONDURAS: Atlántida, Choluteca, Comayagua, Copán, Cortés, El Paraíso, Francisco Morazán, Gracias a Dios, Islas de la Bahía, Lempira, Olancho, Santa Bárbara, Yoro. MEXICO: Chiapas, Guerrero, Hidalgo, Oaxaca, Puebla, Quintana Roo, Tabasco, Veracruz. NICARAGUA: Carazo, Chontales, Estelí, Granada, Jinotega, Masaya, RAA Sur, Río San Juan.

#### References.

Casey 1915, Arrow 1937b, Barrera 1969, Pike et al. 1976, Morón 1979, Endrődi 1964, 1966, 1985a, Sanchez Soto 1998, Ratcliffe 2003, Ratcliffe and Cave 2006, Pacheco-F. et al. 2008, Krajcik 2005, 2012, Ratcliffe et al. 2013.

#### Remarks.

The complete distribution of *C. multiplex* is unknown due to this species' historical confusion with *C. amazona amazona*. The locality records from recent monographs are given here as these are based on authoritatively identified specimens (Ratcliffe 2003, Ratcliffe and Cave 2006, Ratcliffe et al. 2013). *Cyclocephala detecta* was described from specimens collected in Mexico and Nicaragua, and this species is currently considered a synonym of *C. amazona*. Based on these reported locality data, it is possible that *C. multiplex* is a junior synonym of *C. detecta*.

### Cyclocephala munda

Taxon classificationAnimaliaColeopteraScarabaeidae

Kirsch, 1870

Cyclocephala munda Kirsch, 1870: 355–356 [original combination].

#### Types.

Lectotype ♂ at MTD (Endrődi 1966).

#### Distribution.

BRAZIL: Amazonas. COLOMBIA: Boyacá, Cundinamarca. FRENCH GUIANA: Cayenne. PERU: Loreto, Pasco. SURINAME.

#### References.

Kirsch 1870, Arrow 1937b, Blackwelder 1944, Pike et al. 1976, Dechambre and Endrődi 1984, Endrődi 1964, 1966, 1985a, Ratcliffe 1992b, Restrepo et al. 2003, García-Robledo et al. 2005, Ponchel 2011, Krajcik 2005, 2012, López-García et al. 2015, Ratcliffe et al. 2015, Gasca-Álvarez and Deloya 2016.

### Cyclocephala mustacha

Taxon classificationAnimaliaColeopteraScarabaeidae

Ratcliffe, 2003

Cyclocephala mustacha Ratcliffe, 2003: 64, 68, 74, 78, 168–170 [original combination].

#### Types.

Holotype ♂ at UNSM (Ratcliffe 2003).

#### Distribution.

PANAMA: Panamá.

#### References.

Ratcliffe 2003, Krajcik 2005, 2012.

### Cyclocephala mutata

Taxon classificationAnimaliaColeopteraScarabaeidae

Harold, 1869

Cyclocephala mutata Harold, 1869a: 124 [original combination, new replacement name for Cyclocephala frontalis Burmeister, 1847].
Cyclocephala sanguinicollis ab. mutata Harold [new infrasubspecific status by Endrődi 1966: 301].
Cyclocephala mutata Harold [revalidated species status by Ratcliffe 2002a: 30].
**syn.** Cyclocephala frontalis Burmeister, 1847: 50 [original combination, homonym of Cyclocephala frontalisChevrolat 1844].
Cyclocephala mutata Harold [new replacement name by Harold 1869a: 124].
**syn.** Cyclocephala laevicauda Arrow, 1902: 138–139 [original combination].
Homochromina laevicauda (Arrow) [new combination by Casey 1915: 165].
Cyclocephala laevicauda Arrow [revised combination by Arrow 1937b: 8, 11].
Cyclocephala sanguinicollis ab. laevicauda Arrow [new infrasubspecific status by Endrődi 1966: 301].
Cyclocephala mutata Harold [synonymy by Ratcliffe 2002a: 30].
**syn.** Cyclocephala pseudoisabellina Endrődi, 1980: 38 [original combination].
Cyclocephala mutata Harold [synonymy by Ratcliffe 2003: 171].
**syn.** Cyclocephala vitracelisDechambre 1999: 21–22 [original combination].
Cyclocephala mutata Harold [synonymy by Ratcliffe 2003: 171].

#### Types.

Holotype ♂ of *C. vitracelis* at MNHN (Dechambre 1999). Holotype ♀ of *C. pseudoisabellina* in André Gaudaíre-Thore Collection (Sens, France) (Endrődi 1980). Lectotype ♂ of *C. frontalis* at MNHN (Dechambre 1991b). Type of *C. laevicauda* at BMNH (Endrődi 1966). Endrődi (1966) mentioned a specimen of *C. mutata* at MNHN could be important for the nomenclatural stability of this species, but he noted that this specimen was not a type.

#### Distribution.

COSTA RICA: Alajuela, Cartago, Guanacaste, Heredia, Limón, Puntarenas, San José. GUATEMALA: Alta Verapaz, Escuintla, San Marcos, Suchitepéquez, Zacapa. HONDURAS: Atlántida, La Paz, Lempira, Ocotepeque, Olancho, Yoro. MEXICO: Chiapas, Hidalgo, Veracruz, Yucatán. NICARAGUA: Río San Juan. PANAMA: Bocas del Toro, Chiriquí, Former Canal Zone, Panamá.

#### References.

Burmeister 1847, Harold 1869a, Bates 1888, Casey 1915, Arrow 1902, 1937b, Blackwelder 1944, Endrődi 1966, 1980, 1985a, Dechambre 1991b, 1999, Ratcliffe 2002a, 2003, Ratcliffe and Cave 2006, Krajcik 2005, 2012, Breeschoten et al. 2013, García-López et al. 2013, Ratcliffe et al. 2013.

#### Remarks.

*C. mutata* is listed as occurring in Colombia (Cauca) by Gasca-Álvarez and Deloya (2016), citing the data of Endrődi (1966). Endrődi (1966) considered this taxa as an “ab.” (infrasubspecific entity) of *C. sanguinicollis*. The Endrődi (1966) data from Colombia may refer to *C. sanguinicollis* and should be reevaluated.

### Cyclocephala nana

Taxon classificationAnimaliaColeopteraScarabaeidae

Dechambre, 1999

Cyclocephala nana Dechambre, 1999: 14 [original combination].

#### Types.

Holotype ♂ at MNHN (Dechambre 1999).

#### Distribution.

BOLIVIA: Cochabamba.

#### References.

Dechambre 1999, Krajcik 2005, 2012.

### Cyclocephala nicolasi

Taxon classificationAnimaliaColeopteraScarabaeidae

Dupuis, 2018

Cyclocephala nicolasi Dupuis, 2018: 9–12 [original combination].

#### Types.

Holotype ♂ at FDPC (Dupuis 2018).

#### Distribution.

PERU: Junín (Dupuis 2018).

#### References.

Dupuis 2018.

### Cyclocephala nigerrima

Taxon classificationAnimaliaColeopteraScarabaeidae

Bates, 1888

Cyclocephala nigerrima Bates, 1888: 310–311 [original combination].

#### Types.

Type ♂ at BMNH (Endrődi 1966).

#### Distribution.

COSTA RICA: Alajuela, Cartago, Guanacaste, Heredia, Puntarenas, San José. PANAMA: Chiriquí.

#### References.

Bates 1888, Arrow 1937b, Blackwelder 1944, Pike et al. 1976, Endrődi 1964, 1966, 1985a, Goldwasser 1987, 2000, Croat 1997, Ratcliffe 1992a, 2002a, 2003, García-Robledo et al. 2004, 2005, Krajcik 2005, 2012, Breeschoten et al. 2013, García-López et al. 2013.

### Cyclocephala nigra

Taxon classificationAnimaliaColeopteraScarabaeidae

(Endrődi, 1979)

Mimeoma nigra Endrődi, 1979: 216–217 [original combination].
Cyclocephala nigra (Endrődi) [new combination by Moore et al. 2015: 898].

#### Types.

Holotype ♂ at USNM (Endrődi 1979).

#### Distribution.

DOMINICAN REPUBLIC: Dajabón.

#### References.

Endrődi 1979, 1985a, Krajcik 2005, 2012, Ratcliffe and Cave 2015, Moore et al. 2015.

### Cyclocephala nigritarsis

Taxon classificationAnimaliaColeopteraScarabaeidae

Ratcliffe, 1992

Cyclocephala nigritarsis Ratcliffe, 1992a: 222–224 [original combination].

#### Types.

Holotype ♂ at UNSM (Ratcliffe 1992a).

#### Distribution.

COSTA RICA: Alajuela, Guanacaste, Heredia, Limón, San José. PANAMA: Coclé, Colón, Former Canal Zone, Panamá.

#### References.

Ratcliffe 1992a, 2002a, 2003, Krajcik 2005, 2012, García-López et al. 2013.

### Cyclocephala nigrobasalis

Taxon classificationAnimaliaColeopteraScarabaeidae

Höhne, 1923

Cyclocephala nigrobasalis Höhne, 1923b: 370 [original combination].

#### Types.

Lectotype ♂ at ZMHB (Endrődi 1966).

#### Distribution.

BRAZIL: Minas Gerais. COLOMBIA: Chocó, Cundinamarca. VENEZUELA: Mérida.

#### References.

Höhne 1923b, Arrow 1937b, Blackwelder 1944, Pike et al. 1976, Endrődi 1966, 1985a, Dechambre 1999, Krajcik 2005, 2012, López-García et al. 2015, Gasca-Álvarez and Deloya 2016.

### Cyclocephala nigropicta

Taxon classificationAnimaliaColeopteraScarabaeidae

Dechambre & Endrődi, 1983

Cyclocephala nigropicta Dechambre & Endrődi, 1983: 83–84 [original combination].

#### Types.

Holotype ♂ at MNHN (Dechambre and Endrődi 1983).

#### Distribution.

ECUADOR: Cotopaxi, Santo Domingo de los Colorados.

#### References.

Dechambre and Endrődi 1983, Ratcliffe 1989, Dechambre 1999, Dupuis 1999, Krajcik 2005, 2012.

### Cyclocephala niguasa

Taxon classificationAnimaliaColeopteraScarabaeidae

Dechambre & Endrődi, 1984

Cyclocephala niguasa Dechambre & Endrődi, 1984: 171 [original combination].

#### Types.

Holotype ♂ at MNHN (Dechambre and Endrődi 1984).

#### Distribution.

ECUADOR: Cotopaxi, Los Ríos, Pichincha.

#### References.

Dechambre and Endrődi 1984, Krajcik 2005, 2012.

### Cyclocephala nike

Taxon classificationAnimaliaColeopteraScarabaeidae

Ratcliffe, 1992

Cyclocephala nike Ratcliffe, 1992a: 224–226 [original combination].

#### Types.

Holotype ♂ at UNSM (Ratcliffe 1992a).

#### Distribution.

COSTA RICA: Puntarenas. PANAMA: Chiriquí.

#### References.

Ratcliffe 1992a, 2002a, 2003, Krajcik 2005, 2012.

### Cyclocephala nodanotherwon

Taxon classificationAnimaliaColeopteraScarabaeidae

Ratcliffe, 1992

Cyclocephala nodanotherwon Ratcliffe, 1992b: 184 [original combination].

#### Types.

Holotype ♂ at UNSM (Ratcliffe 1992b).

#### Distribution.

BRAZIL: Amazonas.

#### References.

Ratcliffe 1992b, Krajcik 2005, 2012.

### Cyclocephala notata

Taxon classificationAnimaliaColeopteraScarabaeidae

(Illiger, 1806)

Melolontha notata Illiger, 1806: 235–236 [original combination, new replacement name for Melolontha signata Olivier].
Cyclocephala notata (Illiger) [new combination by Burmeister 1847: 55].
**syn.** Cyclocephala insularis Laporte, 1840: 125 [original combination].
Cyclocephala notata (Illiger) [synonymy by Burmeister 1847: 55].
**syn.** Melolontha signata Olivier, 1789: 28–29 [original combination, homonym of Melolontha signata Fabricius].
Melolontha notata Illiger, 1806: 235–236 [new replacement name for Melolontha signata Olivier].

#### Types.

Lectotype ♂ of *M. notata* at ZMHB (Endrődi 1966). The type of *C. signata* is missing (Endrődi 1966). Endrődi (1966) did not determine where the type material of *C. insularis* is deposited.

#### Distribution.

CUBA: Camagüey, Guantánamo, Holguin, Santiago de Cuba. DOMINICAN REPUBLIC: Azua, Baoruco, Barahona, Dajabón, Distrito Nacional, Hato Mayor, Independencia, La Altagracia, La Romana, La Vega, Monseñor Nouel, Monte Cristi, Monte Plata, Pedernales, Peravia, Puerto Plata, Salcedo, Samana, San Cristóbal, San Jose Ocoa, San Juan, San Pedro de Macorís, Santiago, Santo Domingo, Valverde. HAITI: Artibonite, Centre, Grand Anse, Nord-Ouest, Ouest, Sud. JAMAICA: St. James, St. Thomas, Trelawny.

#### References.

Olivier 1789, Illiger 1806, Laporte 1840, Sturm 1843, Burmeister 1847, Harold 1869b, Leng and Mutchler 1914, 1917, Chapin 1932, Arrow 1937b, Blackwelder 1944, Howden 1970, Pike et al. 1976, Endrődi 1966, 1985a, Fernández García 2006, Krajcik 2005, 2012, Breeschoten et al. 2013, Ratcliffe and Cave 2015.

### Cyclocephala obscura

Taxon classificationAnimaliaColeopteraScarabaeidae

Endrődi, 1966

Cyclocephala obscura Endrődi, 1966: 85, 137, 269–270 [original combination].

#### Types.

Holotype ♂ at NHMB (Frey Collection) (Endrődi 1966).

#### Distribution.

BOLIVIA: Cochabamba. PERU: Madre de Dios.

#### References.

Endrődi 1966, 1985a, Dechambre 1999, Krajcik 2005, 2012, Ratcliffe et al. 2015.

### Cyclocephala occipitalis

Taxon classificationAnimaliaColeopteraScarabaeidae

Fairmaire, 1892

Cyclocephala occipitalis Fairmaire, 1892: 244–245 [original combination].

#### Types.

Invalid neotype ♂ at HNHM (Endrődi Collection) (Endrődi 1966).

#### Distribution.

ARGENTINA: La Rioja. BRAZIL: Bahia, Rio Grande do Sul, Santa Catarina.

#### References.

Fairmaire 1892, Bertkau 1893, Arrow 1937b, Blackwelder 1944, Pike et al. 1976, Endrődi 1966, 1985a, Marques and Gil-Santana 2009, Krajcik 2005, 2012, Breeschoten et al. 2013, Ratcliffe 2014.

### Cyclocephala ocellata

Taxon classificationAnimaliaColeopteraScarabaeidae

Burmeister, 1847

Cyclocephala ocellata Burmeister, 1847: 40 [original combination].
**syn.** Albridarollia ocellata Bolívar y Pieltain, 1963: 183 [original combination].
Cyclocephala ocellata Burmeister (synonymy by Endrődi 1966: 61, 272].

#### Types.

Lectotype ♂ of *C. ocellata* at MNHN (Dechambre 1991b). Holotype ♂ of *A. ocellata* at MACN (Antonio Martínez Collection) (Bolívar y Pieltain et al. 1963).

#### Distribution.

BRAZIL: Amazonas. COLOMBIA: Antioquia. ECUADOR: Morona-Santiago. FRENCH GUIANA: Kourou, St.-Élie. PERU: Cusco, Ucayali.

#### References.

Burmeister 1847, Harold 1869b, Arrow 1911, 1937b, Blackwelder 1944, Bolívar y Pieltain et al. 1963, Pike et al. 1976, Endrődi 1964, 1966, 1985a, Dechambre 1979a, 1991b, Ratcliffe 1992b, Restrepo et al. 2003, Ponchel 2011, Krajcik 2005, 2012, Breeschoten et al. 2013, Yepes-Rodriguez et al. 2013, Ratcliffe et al. 2015, Gasca-Álvarez and Deloya 2016.

#### Remarks.

*Cyclocephala ocellata* was originally described from Mexico (Burmeister 1847). These data were erroneous, and *C. ocellata* is considered a South American species (Endrődi 1966). Endrődi (1964) redescribed *C. ocellata* but did not mention Burmeister’s type material. A lectotype was later designated (Dechambre 1991b). *Albridarollia ocellata* was described as a separate species (Bolívar y Pieltain et al. 1963). Endrődi (1966) treated the genus *Albridarollia* as a synonym of *Cyclocephala* but did not clearly establish that *C. ocellata* and *A. ocellata* are subjective synonyms.

### Cyclocephala ochracea

Taxon classificationAnimaliaColeopteraScarabaeidae

Prell, 1937

Cyclocephala ochracea Prell, 1937b: 495 [original combination].

#### Types.

Endrődi (1966) did not find the type material of *C. ochracea* and speculated that it would be at ZMHB (Prell Collection).

#### Distribution.

ARGENTINA: Buenos Aires, Catamarca, Chaco, Córdoba, Corrientes, Formosa, Jujuy, Salta, Santiago del Estero, Tucumán. BOLIVIA: Beni, Cochabamba, La Paz, Santa Cruz. COLOMBIA. PARAGUAY: Paraguarí. URUGUAY: Rivera, Tacuarembó.

#### References.

Prell 1937b, Blackwelder 1944, Pike et al. 1976, Saenz and Morelli 1984, Endrődi 1964, 1966, 1967b, 1969a, 1985a, Restrepo et al. 2003, Krajcik 2005, 2012, Gasca-Álvarez and Deloya 2016.

### Cyclocephala octopunctata

Taxon classificationAnimaliaColeopteraScarabaeidae

Burmeister, 1847

Cyclocephala octopunctata Burmeister, 1847: 65–66 [original combination].

#### Types.

Lectotype ♀ at MNHN (Dechambre 1991b). Invalid neotype ♂ at HNHM (Endrődi Collection) (Endrődi 1966).

#### Distribution.

BOLIVIA: Cochabamba, Santa Cruz. BRAZIL: Goiás, Mato Grosso, Rio de Janeiro, Santa Catarina. FRENCH GUIANA: Cayenne. PERU: Madre de Dios, Pasco.

#### References.

Burmeister 1847, Harold 1869b, Arrow 1937b, Blackwelder 1944, Howden and Endrődi 1966, Pike et al. 1976, Endrődi 1964, 1966, 1985a, Dechambre 1991b, Joly 2000a, Cavalcante et al. 2009, Krajcik 2005, 2012, Moore and Jameson 2013, Ratcliffe et al. 2015, Costa et al. 2017.

### Cyclocephala ohausiana

Taxon classificationAnimaliaColeopteraScarabaeidae

Höhne, 1923

Cyclocephala ohausiana Höhne, 1923b: 362–363 [original combination].

#### Types.

Lectotype ♂ at ZMHB (Endrődi 1966).

#### Distribution.

BRAZIL: Goiás, Mato Grosso, Minas Gerais, São Paulo.

#### References.

Höhne 1923b, Arrow 1937b, Blackwelder 1944, Pike et al. 1976, Endrődi 1966, 1985a, Gottsberger 1986, 1988, 1989, 1992, Gottsberger and Silberbauer-Gottsberger 1988, 2006, Krajcik 2005, 2012, Costa et al. 2017.

### Cyclocephala olivieri

Taxon classificationAnimaliaColeopteraScarabaeidae

Arrow, 1911

Cyclocephala olivieri Arrow, 1911: 171 [original combination, new replacement name for Cyclocephala nigrocephala Schönherr].
**syn.** Melolontha melanocephala Olivier, 1789: 42 [original combination, junior homonym of Melolontha melanocephala Fabricius].
Melolontha nigrocephala Schönherr, 1817: 190 [original combination, new replacement name for Melolontha melanocephala Olivier].
**syn.** Melolontha nigrocephala Schönherr, 1817: 190 [original combination, new replacement name for Melolontha melanocephala Olivier and junior homonym of Melolontha nigrocephala (DeGeer)].
Cyclocephala nigrocephala (Schönherr) [new combination by Burmeister 1847: 58].
Cyclocephala olivieri Arrow [new replacement name by Arrow 1911: 171].

#### Types.

Type material for this species was not found, and Endrődi’s (1966) description was based upon Burmeister specimens.

#### Distribution.

ARGENTINA: Formosa, Tucumán. BOLIVIA: Beni. BRAZIL: Pernambuco. COLOMBIA: Cundinamarca. PARAGUAY: Asunción. SURINAME. TRINIDAD AND TOBAGO: Trinidad. URUGUAY: Canelones, Florida, Maldonado, Montevideo, Paysandú, Rocha. VENEZUELA: Apure, Capital District, Bolívar.

#### References.

Olivier 1789, Schönherr 1817, Burmeister 1847, Harold 1869b, Arrow 1911, 1937b, Blackwelder 1944, Pike et al. 1976, Dechambre and Endrődi 1984, Saenz and Morelli 1984, Dechambre and Endrődi 1984, Endrődi 1966, 1985a, Dechambre 1999, Restrepo et al. 2003, Krajcik 2005, 2012, Gasca-Álvarez and Deloya 2016.

### Cyclocephala ovulum

Taxon classificationAnimaliaColeopteraScarabaeidae

Bates, 1888

Cyclocephala ovulum Bates, 1888: 306–307 [original combination].
Ochrosidia (Ochrosidia) ovulum (Bates) [new combination and new subgeneric classification by Casey 1915: 158].
Cyclocephala ovulum Bates [revised combination and removal of subgeneric classification by Arrow 1937b: 8, 14].
Cyclocephala testacea ab. ovulum Bates [new infrasubspecific status by Endrődi 1966: 318].
Cyclocephala ovulum Bates [revalidated species status by Ratcliffe 2003: 180].

#### Types.

Endrődi (1966) did not discuss the type material of this species or report its housing institution.

#### Distribution.

ARGENTINA. BRAZIL: Amazonas. COLOMBIA: Amazonas, Atlántico, Chocó, Cundinamarca, Guainía. COSTA RICA: Alajuela, Cartago, Guanacaste, Limón, Puntarenas, San José. ECUADOR: Napo. EL SALVADOR: Ahuachapán, La Libertad, Morazán, San Miguel. FRENCH GUIANA: Cayenne, Campoi, St.-Georges, St-Laurent du Maroni. GUATEMALA: Baja Verapaz, El Progreso, Suchitepéquez, Zacapa. GUYANA. HONDURAS: Atlántida, Choluteca, Comayagua, El Paraíso, Francisco Morazán, La Paz, Olancho, Valle, Yoro. MEXICO: Chiapas, Guerrero, Jalisco, Morelos, Nayarit, Oaxaca, Puebla, Sinaloa, Veracruz. NICARAGUA: Chontales, León, Managua, RAA Norte. PANAMA: Bocas del Toro, Chiriquí, Coclé, Colón, Former Canal Zone, Panamá, Veraguas. PARAGUAY: Alto Paraná.

#### References.

Bates 1888, Casey 1915, Bodkin 1919, Arrow 1937b, Blackwelder 1944, Hayward 1946, Gruner 1971, Endrődi 1966, 1969a, 1985a, Maes 1987, Morón et al. 1988, Thomas 1993, Deloya and Morón 1994, Navarrete-Heredia et al. 2001, Ratcliffe 2002a, 2003, Ratcliffe and Cave 2006, Pacheco-F. et al. 2008, Gómez and Morón 2010, Neita-Moreno 2011, Krajcik 2005, 2012, Moore and Jameson 2013, Otavo et al. 2013, Ratcliffe et al. 2013, García-Atencia and Martínez-Hernández 2015, López-García et al. 2015, Gasca-Álvarez and Deloya 2016, Romero-López and Morón 2017.

### Cyclocephala pan

Taxon classificationAnimaliaColeopteraScarabaeidae

Ratcliffe, 1992

Cyclocephala pan Ratcliffe, 1992a: 226–227 [original combination].

#### Types.

Holotype ♂ at UNSM (Ratcliffe 1992a).

#### Distribution.

COSTA RICA: Alajuela, Guanacaste, Heredia, Limón. GUATEMALA: Izabal, Santa Rosa. PANAMA: Bocas del Toro, Colón, Panamá, San Blas.

#### References.

Ratcliffe 1992a, 2002a, 2003, Krajcik 2005, 2012, García-López et al. 2013, Ratcliffe et al. 2013.

### Cyclocephala panthera

Taxon classificationAnimaliaColeopteraScarabaeidae

Dechambre, 1979

Cyclocephala panthera Dechambre, 1979a: 164–165 [original combination].

#### Types.

Holotype ♂ at MNHN (Dechambre 1979a).

#### Distribution.

BRAZIL: Distrito Federal, Pará. PERU.

#### References.

Dechambre 1979a, Endrődi 1985a, Ratcliffe 1992b, Krajcik 2005, 2012, Ratcliffe et al. 2015.

### Cyclocephala paraflora

Taxon classificationAnimaliaColeopteraScarabaeidae

Martínez, 1978

Cyclocephala paraflora Martínez, 1978b: 12–15 [original combination].

#### Types.

Holotype ♂ at MACN (Antonio Martínez Collection) (Martínez 1978a).

#### Distribution.

BRAZIL: Roraima. COLOMBIA: Amazonas. FRENCH GUIANA: St.-Élie. PERU.

#### References.

Martínez 1978b, Endrődi 1985a, Dechambre 1979a, 1992, 1999, Krajcik 2005, 2012, Otavo et al. 2013, Ratcliffe et al. 2015, Gasca-Álvarez and Deloya 2016.

### Cyclocephala paraguayensis marginella

Taxon classificationAnimaliaColeopteraScarabaeidae

Endrődi, 1966

Cyclocephala paraguayensis marginella Endrődi, 1966: 129, 278–279 [original combination].

#### Types.

Holotype ♂ at NHMB (Frey Collection) (Endrődi 1966).

#### Distribution.

BRAZIL: Pernambuco, Rio Grande do Norte.

#### References.

Pike et al. 1976, Endrődi 1966, 1985a, Krajcik 2005, 2012.

### Cyclocephala paraguayensis paraguayensis

Taxon classificationAnimaliaColeopteraScarabaeidae

Arrow, 1903

Cyclocephala paraguayensis Arrow, 1903: 257 [original combination].

#### Types.

Endrődi (1966) did not discuss the type material of this species or report its housing institution.

#### Distribution.

ARGENTINA: Entre Ríos, Misiones, Santa Fe. BOLIVIA: Cochabamba, Santa Cruz. BRAZIL: Amazonas, Bahia, Goiás, Mato Grosso, Paraná, Pernambuco, Piauí, Rio Grande do Norte, Rio Grande do Sul, Santa Catarina, São Paulo. COLOMBIA: Cundinamarca. ECUADOR. PARAGUAY: Asunción, Cordillera, Guairá, Paraguarí. PERU: Quispicanchi. URUGUAY.

#### References.

Seidlitz 1904, Arrow 1903, 1937b, Blackwelder 1944, Pike et al. 1976, Endrődi 1966, 1985a, Gottsberger 1986, 1989, Restrepo et al. 2003, Riehs 2005, 2006, Costa and Iannuzzi 2010, Grossi et al. 2011, Krajcik 2005, 2012, Breeschoten et al. 2013, Albuquerque et al. 2014, 2016, Ratcliffe et al. 2015, Gasca-Álvarez and Deloya 2016.

#### Remarks.

*Cyclocephala paraguayensis* was recorded from Honduras (Endrődi 1966, 1985a). Further studies have not encountered *C. paraguayensis* in Honduras, and this species is probably restricted to South America (Ratcliffe and Cave 2006).

### Cyclocephala parallela

Taxon classificationAnimaliaColeopteraScarabaeidae

(Casey, 1915)

Ochrosidia (Ochrosidia) parallela Casey, 1915: 144 [original combination].
Cyclocephala parallela (Casey) [new combination and removal of subgeneric classification by Arrow 1937b: 8 14].
Cyclocephala borealis Arrow [synonymy by Saylor 1945: 385].
Cyclocephala parallela (Casey) [revalidated species status by Endrődi 1966: 279].

#### Types.

Types at USNM (Endrődi 1966).

#### Distribution.

UNITED STATES: Florida, Georgia, North Carolina, South Carolina.

#### References.

Casey 1915, Leng 1920, Arrow 1937b, Saylor 1945, Kirk 1970, Woodruff 1973, Pike et al. 1976, Reinert 1979, Gordon and Anderson 1981, Endrődi 1966, 1985a, Boucias et al. 1986, Cherry and Boucias 1989, Cherry et al. 1990, Hardy 1991, Raid and Cherry 1992, Cherry and Coale 1994, Stansly et al. 1994, Cherry and Klein 1997, Peck and Thomas 1998, Harpootlian 2001, Cappaert and Smitley 2002, Buss 2006, Jackson and Klein 2006, Frank 2008, Smith 2003, 2009, Krajcik 2005, 2012, Cherry 1984a, b, 1985, 2012, Breeschoten et al. 2013, Ratcliffe et al. 2013, Gyawaly et al. 2016, Ratcliffe and Cave 2017.

#### Remarks.

Endrődi (1966, 1985a) reported a no-data specimen of *C. parallela* from Mexico. This record is likely based on a misidentification and *C. parallela* is restricted to the southeastern United States (Ratcliffe et al. 2013). The data points listed outside of Florida for *C. parallela* should be reevaluated, as some authors have hypothesized that this species is endemic to Florida (Peck and Thomas 1998, Harpootlian 2001).

### Cyclocephala pardolocarnoi

Taxon classificationAnimaliaColeopteraScarabaeidae

Dechambre, 1995

Cyclocephala pardolocarnoi Dechambre, 1995: 12–13 [original combination].

#### Types.

Holotype ♂ at “Museo de Ciencias Naturales, Cali (Colombie)” (Dechambre 1995).

#### Distribution.

COLOMBIA: Amazonas, Chocó, Quindío, Sucre, Tolima, Valle del Cauca. PANAMA: Bocas del Toro, Former Canal Zone, Panamá.

#### References.

Dechambre 1995, Ratcliffe 2002a, 2003, Neita-Moreno et al. 2006, Neita-Moreno 2011, Krajcik 2005, 2012, Pardo-Locarno 2013, Gasca-Álvarez and Deloya 2016.

### Cyclocephala pasadenae

Taxon classificationAnimaliaColeopteraScarabaeidae

(Casey, 1915)

Ochrosidia (Ochrosidia) pasadenae Casey, 1915: 148 [original combination].
Cyclocephala pasadenae (Casey) [new combination and removal of subgeneric classification by Arrow 1937b: 8, 14].
**syn.** Ochrosidia (Ochrosidia) arizonica Casey, 1915: 149 [original combination].
Cyclocephala arizonica (Casey) [new combination and removal of subgeneric classification by Arrow 1937b: 8].
Cyclocephala pasadenae (Casey) [synonymy by Saylor 1945: 385].
**syn.** Ochrosidia (Ochrosidia) facilis Casey, 1915: 150 [original combination].
Cyclocephala facilis (Casey) [new combination and removal of subgeneric classification by Arrow 1937b: 8, 10].
Cyclocephala pasadenae (Casey) [synonymy by Saylor 1945: 385].
**syn.** Ochrosidia (Ochrosidia) melinaCasey 1915: 149 [original combination].
Cyclocephala melina (Casey) [new combination and removal of subgeneric classification by Arrow 1937b: 8, 13].
Cyclocephala pasadenae (Casey) [synonymy by Saylor 1945: 385].
**syn.** Ochrosidia (Ochrosidia) ovulata Casey, 1915: 151 [original combination].
Cyclocephala ovulata (Casey) [new combination and removal of subgeneric classification by Arrow 1937b: 8, 14].
Cyclocephala pasadenae (Casey) [synonymy by Saylor 1945: 385].
**syn.** Ochrosidia (Ochrosidia) pusilla Casey, 1915: 150 [original combination].
Cyclocephala melina var. pusilla [new combination, removal of subgeneric classification, and new infrasubspecific status by Arrow 1937b: 8, 13].
Cyclocephala pasadenae (Casey) [synonymy by Saylor 1945: 385].
**syn.** Ochrosidia (Ochrosidia) validicepsCasey 1915: 148 [original combination].
Cyclocephala validiceps (Casey) [new combination and removal of subgeneric classification by Arrow 1937b: 8, 16].
Cyclocephala pasadenae (Casey) [synonymy by Saylor 1945: 385].

#### Types.

The type material of all of these Casey species are at USNM (Endrődi 1966).

#### Distribution.

MEXICO: Baja California, Baja California Sur, Chihuahua, Coahuila, Durango, Hidalgo, Jalisco, Nuevo León, San Luis Potosí, Sonora, Tamaulipas, Zacatecas. UNITED STATES: Arizona, Arkansas, California, Colorado, Hawaii, Iowa, Kansas, Missouri, Nebraska, Nevada, New Mexico, Oklahoma, South Dakota, Texas, Utah.

#### References.

Casey 1915, Leng 1920, Arrow 1937b, Saylor 1945, 1948, Werner and Butler 1965, Ritcher 1944, 1966, Howden and Endrődi 1966, Martínez 1969, Pike et al. 1976, Endrődi 1966, 1985a, Miller 1985, Gerhardt and Stanghellini 1971, Stone 1986, Bueno et al. 1988, Hardy 1991, Ratcliffe 1991, Cokendolpher 1993, Blanco-Montero and Ward 1995, Merchant and Crocker 1996, Poole and Gentili 1996, Ratcliffe and Morón 1997, Koppenhöfer and Kaya 1997, 1998, Potter 1998, Bauernfeind et al. 1999, Bauernfeind 2001, Koppenhöfer and Fuzy 2003b, Riley and Wolfe 2003, Yildrim and Hoy 2003, Ansari et al. 2004, Cranshaw 2004, Rogers and Potter 2004b, Koppenhöfer et al. 1999, 2000a, 2002a, b, 2004, Koppenhöfer and Grewal 2005, Grewal et al. 2005, Blanco-Montero and Hernandez 1995, 2006, Ratcliffe and Paulsen 2008, Jameson et al. 2009, Morales-Rodriguez and Peck 2009, Smith 2003, 2009, Bélair et al. 2010, Holmstrup et al. 2010, Mashtoly et al. 2010, Negrisoli et al. 2010, Krajcik 2005, 2012, Perera et al. 2012, Breeschoten et al. 2013, de Coninck et al. 2013, Ratcliffe et al. 2013, Hussaini 2014, Li and Bouwer 2014, Christians et al. 2016, Gyawaly et al. 2016, Wu et al. 2017, Ratcliffe and Cave 2017.

### Cyclocephala perconfusa

Taxon classificationAnimaliaColeopteraScarabaeidae

Dechambre, 1992

Cyclocephala perconfusa Dechambre, 1992: 64–65 [original combination].

#### Types.

Holotype ♂ at MNHN (Dechambre 1992).

#### Distribution.

ECUADOR: Cañar, Cotopaxi, Pichincha, Santo Domingo de los Tsáchilas.

#### References.

Dechambre 1992, Ratcliffe 1992b, Krajcik 2005, 2012.

### Cyclocephala pereirai

Taxon classificationAnimaliaColeopteraScarabaeidae

(Martínez, 1960)

Eremophygus pereirai Martínez, 1960b: 131–133 [original combination].
Cyclocephala pereirai (Martínez) [new combination by Martínez 1975b: 264].

#### Types.

Holotype ♂ at MACN (Antonio Martínez Collection) (Martínez 1960b).

#### Distribution.

ARGENTINA: Jujuy, Río Negro.

#### References.

Pike et al. 1976, Martínez 1960b, 1975b, 1978a, Endrődi 1985a, Krajcik 2005, 2012.

### Cyclocephala perforata

Taxon classificationAnimaliaColeopteraScarabaeidae

Arrow, 1913

Cyclocephala perforata Arrow, 1913: 465 [original combination].

#### Types.

Type at BMNH (Endrődi 1966).

#### Distribution.

BRAZIL: Mato Grosso, Pará. FRENCH GUIANA: Kourou, Mana, St.-Élie, St.-Georges, St.-Laurent du Maroni.

#### References.

Arrow 1913, 1937b, Blackwelder 1944, Gruner 1971, Pike et al. 1976, Endrődi 1966, 1985a, Dechambre 1979a, 1992, Dupuis 2008, 2014, Ponchel 2011, Krajcik 2005, 2012, Breeschoten et al. 2013.

### Cyclocephala perplexa

Taxon classificationAnimaliaColeopteraScarabaeidae

Ratcliffe, 2008

Cyclocephala perplexa Ratcliffe, 2008: 237–238 [original combination].

#### Types.

Holotype ♂ at UNSM (Ratcliffe 2008).

#### Distribution.

BOLIVIA: La Paz.

#### References.

Ratcliffe 2008, Krajcik 2012.

### Cyclocephala peruana

Taxon classificationAnimaliaColeopteraScarabaeidae

Endrődi, 1966

Cyclocephala peruana Endrődi, 1966: 78, 136, 282–283 [original combination].

#### Types.

Holotype ♂ at NHMB (Frey Collection) (Endrődi 1966).

#### Distribution.

PERU: Loreto, Madre de Dios, Pasco.

#### References.

Pike et al. 1976, Endrődi 1966, 1985a, Ratcliffe 1992b, Krajcik 2005, 2012, Ratcliffe et al. 2015.

### Cyclocephala pichinchana

Taxon classificationAnimaliaColeopteraScarabaeidae

Dechambre, 1992

Cyclocephala pichinchana Dechambre, 1992: 61–62 [original combination].

#### Types.

Holotype ♂ at MNHN (Dechambre 1992).

#### Distribution.

ECUADOR: Cotopaxi, Pichincha, Santo Domingo de los Tsáchilas.

#### References.

Dechambre 1992, Krajcik 2005, 2012.

### Cyclocephala picipes

Taxon classificationAnimaliaColeopteraScarabaeidae

(Olivier, 1789)

Melolontha picipes Olivier, 1789: 80–81 [original combination].
Cyclocephala picipes (Olivier) [new combination by Burmeister 1847: 522].

#### Types.

Invalid neotype ♂ at HNHM (Endrődi Collection) (Endrődi 1966).

#### Distribution.

BRAZIL: Amazonas, Mato Grosso, Pará. FRENCH GUIANA: St.-Laurent du Maroni.

#### References.

Olivier 1789, Burmeister 1847, Reiche 1859, Harold 1869b, Arrow 1937b, Blackwelder 1944, Pike et al. 1976, Dechambre 1980, Webber 1981, Endrődi 1966, 1985a, Ratcliffe 1992b, Ponchel 2011, Krajcik 2005, 2012.

### Cyclocephala picta

Taxon classificationAnimaliaColeopteraScarabaeidae

Burmeister, 1847

Cyclocephala picta Burmeister, 1847: 68 [original combination].
Stigmalia picta (Burmeister) [new combination by Casey 1915: 123].
Cyclocephala picta Burmeister [revised combination by Arrow 1937b: 8, 14].
**syn.** Cyclocephala forsteri mexicoi Endrődi, 1966: 203 [original combination].
Cyclocephala picta Burmeister [synonymy by Ratcliffe et al. 2013: 213].

#### Types.

Lectotype ♀ of *C. picta* at MLUH (Endrődi 1966). Holotype ♂ of *C. forsteri mexicoi* at ZMHB (Endrődi 1966).

#### Distribution.

GUATEMALA: Huehuetenango. MEXICO: Chiapas, Hidalgo, Jalisco, Michoacán, Oaxaca, Puebla, Veracruz, Yucatán.

#### References.

Burmeister 1847, Harold 1869b, H. W. Bates 1888, Arrow 1937b, Blackwelder 1944, Pike et al. 1976, Morón 1977a, Endrődi 1964, 1966, 1967b, 1985a, Dechambre 1979a, 1982, 1992, Thomas 1993, Ratcliffe and Morón 1997, García-Luna et al. 2002, Luna et al. 2007, Krajcik 2005, 2012, Breeschoten et al. 2013, Ratcliffe et al. 2013, Deloya et al. 2016.

### Cyclocephala pilosa

Taxon classificationAnimaliaColeopteraScarabaeidae

Dupuis, 2006

Cyclocephala pilosa Dupuis, 2006: 309–310 [original combination].

#### Types.

Holotype ♂ at FDPC (Dupuis 2006).

#### Distribution.

PERU: Huánuco.

#### References.

Dupuis 2006, Krajcik 2012, Ratcliffe et al. 2015.

### Cyclocephala pokornyi

Taxon classificationAnimaliaColeopteraScarabaeidae

Dupuis, 2014

Cyclocephala pokornyi Dupuis, 2014: 49–51 [original combination].

#### Types.

Holotype ♂ in Pokorny Collection (Prague, Czech Republic).

#### Distribution.

PERU: Pasco.

#### References.

Dupuis 2014.

### Cyclocephala pompanoni

Taxon classificationAnimaliaColeopteraScarabaeidae

Dechambre, 1979

Cyclocephala pompanoni Dechambre, 1979a: 165–166 [original combination].

#### Types.

Holotype ♂ at MNHN (Dechambre 1979a).

#### Distribution.

BRAZIL: Pará.

#### References.

Dechambre 1979a, Endrődi 1985a, Krajcik 2005, 2012.

### Cyclocephala poncheli

Taxon classificationAnimaliaColeopteraScarabaeidae

Dechambre & Duranton, 2005

Cyclocephala poncheli Dechambre & Duranton, 2005: 69–76 [original combination].

#### Types.

Holotype ♂ at MNHN (Dechambre and Duranton 2005).

#### Distribution.

FRENCH GUIANA: Kourou, Roura, St.-Élie.

#### References.

Dechambre and Duranton 2005, Ponchel 2011, Krajcik 2012.

### Cyclocephala porioni

Taxon classificationAnimaliaColeopteraScarabaeidae

Dechambre, 1979

Cyclocephala porioni Dechambre, 1979b: 317–318 [original combination].

#### Types.

Holotype ♂ at MNHN (Dechambre 1979b).

#### Distribution.

COSTA RICA: Alajuela, Cartago, Guanacaste, Heredia, Limón, Puntarenas, San José. ECUADOR: Napo. HONDURAS: Lempira. NICARAGUA: Río San Juan. PANAMA: Bocas del Toro, Chiriquí, Colón, Darien Former Canal Zone, Panamá.

#### References.

Endrődi 1966, 1985a, Dechambre 1979b, 1992, Maes 1994, Ratcliffe 1992a, 2002a, 2003, Malý 2006, Ratcliffe and Cave 2006, Krajcik 2005, 2012, Dupuis 2008, 2014, García-López et al. 2013.

#### Remarks.

Malý (2006) hypothesized that *C. porioni* is restricted to South America and that previous Central American records of *C. porioni* may refer to *C. hartmannorum*.

### Cyclocephala prelli

Taxon classificationAnimaliaColeopteraScarabaeidae

Endrődi, 1967

Cyclocephala prelli Endrődi, 1967b: 86–87 [original combination, new replacement name for Cyclocephala vittoscutellarisEndrődi 1966].
**syn.** Cyclocephala vittoscutellaris Endrődi, 1966: 335–336 [original combination, junior homonym of C. vittoscutellaris Prell].
Cyclocephala prelli Endrődi 1967b [new replacement name by Endrődi 1967b: 86–87].

#### Types.

Holotype ♂ at HNHM (Endrődi Collection) (Endrődi 1966).

#### Distribution.

BRAZIL: Mato Grosso. COLOMBIA: Antioquia, Boyacá, Santander.

#### References.

Pike et al. 1976, Endrődi 1966, 1967b, 1985a, Restrepo et al. 2003, Krajcik 2005, 2012, López-García et al. 2015, Gasca-Álvarez and Deloya 2016.

#### Remarks.

Endrődi (1966) applied the name *C. vittoscutellaris* to his description of this species. Endrődi (1967b) later remarked that there were two different “*vittoscutellaris*” species. Endrődi (1967b) thought his 1966 description should be considered a new species description (attributed to him) with the name *C. vittoscutellaris* Endrődi being a junior homonym. This retroactive “new” species description was not labeled as being intentionally new, and may be invalid.

### Cyclocephala prolongata

Taxon classificationAnimaliaColeopteraScarabaeidae

Arrow, 1902

Cyclocephala prolongata Arrow, 1902: 140 [original combination].

#### Types.

Type at BMNH (Endrődi 1966).

#### Distribution.

BELIZE: Cayo, Orange Walk, Stann Creek, Toledo. COLOMBIA: Antioquia, Chocó, Cundinamarca, Huila, Magdalena, Tolima. COSTA RICA: Heredia, Limón, Puntarenas. GUATEMALA: Alta Verapaz, Izabal, Petén. HONDURAS: Atlántida, Colón, Comayagua, Cortés, Francisco Morazán, Gracias a Dios, Islas de la Bahía, Santa Bárbara, Yoro. MEXICO: Chiapas, Guerrero, Nayarit. NICARAGUA: Río San Juan. PANAMA: Bocas del Toro, Former Canal Zone, Panamá. PERU.

#### References.

Arrow 1902, 1937b, Blackwelder 1944, Bolívar y Pieltain et al. 1963, Pike et al. 1976, Martínez and Martínez 1981, Endrődi 1966, 1985a, Morón et al. 1985, Thomas 1993, Delgado and Castañeda 1994, Dechambre 1995, Ratcliffe and Morón 1997, Ratcliffe 1992a, 2002a, 2003, Restrepo et al. 2003, Ratcliffe and Cave 2006, Núñez-Avellaneda and Neita-Moreno 2009, Neita-Moreno 2011, Krajcik 2005, 2012, Yepes-Rodriguez et al. 2013, Deloya et al. 2014a, Ratcliffe et al. 2013, 2015, Gasca-Álvarez and Deloya 2016, Romero-López and Morón 2017.

### Cyclocephala proxima

Taxon classificationAnimaliaColeopteraScarabaeidae

Dechambre, 1997

Cyclocephala proxima Dechambre, 1997: 14, 24–25 [original combination].

#### Types.

Holotype ♂ at MNHN (Dechambre 1997).

#### Distribution.

COLOMBIA: Valle del Cauca. ECUADOR: Cañar, Cotopaxi, Napo, Pichincha, Santo Domingo de los Tsáchilas.

#### References.

Dechambre 1997, Restrepo et al. 2003, Krajcik 2005, 2012, Gasca-Álvarez and Deloya 2016.

### Cyclocephala pseudoconfusa

Taxon classificationAnimaliaColeopteraScarabaeidae

Ratcliffe, 1992

Cyclocephala pseudoconfusa Ratcliffe, 1992b: 185 [original combination].

#### Types.

Holotype ♂ at UNSM (Ratcliffe 1992b).

#### Distribution.

BRAZIL: Amazonas.

#### References.

Ratcliffe 1992b, Krajcik 2005, 2012.

### Cyclocephala pseudomelanocephala

Taxon classificationAnimaliaColeopteraScarabaeidae

Dupuis, 1996

Cyclocephala pseudomelanocephala Dupuis, 1996: 257 [original combination].

#### Types.

Holotype ♂ at MNHN (Dupuis 1996).

#### Distribution.

BOLIVIA: Cochabamba, La Paz. ECUADOR: Loja. PERU: Cusco.

#### References.

Dupuis 1996, Krajcik 2005, 2012.

### Cyclocephala puberula

Taxon classificationAnimaliaColeopteraScarabaeidae

LeConte, 1863

Cyclocephala puberula LeConte, 1863: 80 [original combination].
Ochrosidia (Ochrosidia) puberula (LeConte) [new combination by Casey 1915: 147].
Cyclocephala puberula LeConte [revised combination by Arrow 1937b: 8, 14].

#### Types.

Type at MCZ (Endrődi 1966).

#### Distribution.

UNITED STATES: Alabama, Florida, Georgia, Louisiana, Mississippi, North Carolina, South Carolina.

#### References.

LeConte 1863, Gerstaecker 1865, Harold 1869b, Horn 1871, Henshaw 1885, Casey 1915, Leng 1920, Arrow 1937b, Brimley 1938, Saylor 1945, Kirk 1970, Woodruff 1973, Pike et al. 1976, Endrődi 1966, 1985a, Hardy 1991, Poole and Gentili 1996, Peck and Thomas 1998, Harpootlian 2001, Smith 2003, 2009, Krajcik 2005, 2012, Ratcliffe and Cave 2017.

### Cyclocephala pugnax

Taxon classificationAnimaliaColeopteraScarabaeidae

Arrow, 1914

Cyclocephala pugnax Arrow, 1914: 274–275 [original combination].

#### Types.

Type at BMNH (Endrődi 1966).

#### Distribution.

BOLIVIA: Cochabamba, La Paz, Santa Cruz. BRAZIL: Amazonas, Mato Grosso do Sul, Pará. COLOMBIA: Amazonas. FRENCH GUIANA: St.-Laurent du Maroni. GUYANA. PERU: Junín, Loreto, Madre de Dios. SURINAME.

#### References.

Arrow 1914, 1937b, Blackwelder 1944, Pike et al. 1976, Endrődi 1966, 1985a, Ratcliffe 1992b, Santos Fava et al. 2011, Krajcik 2005, 2012, Breeschoten et al. 2013, Otavo et al. 2013, Ponchel 2011, 2015, Ratcliffe et al. 2015, Gasca-Álvarez and Deloya 2016.

### Cyclocephala pulchra

Taxon classificationAnimaliaColeopteraScarabaeidae

Dechambre, 1999

Cyclocephala pulchra Dechambre, 1999: 16–17 [original combination].

#### Types.

Holotype ♂ at MNHN (Dechambre 1999).

#### Distribution.

COLOMBIA: Valle del Cauca.

#### References.

Dechambre 1999, Restrepo et al. 2003, Krajcik 2005, 2012.

### Cyclocephala puncticollis

Taxon classificationAnimaliaColeopteraScarabaeidae

Endrődi, 1966

Cyclocephala puncticollis Endrődi, 1966: 75, 290 [original combination].

#### Types.

Holotype ♂ at ZMHB (Endrődi 1966).

#### Distribution.

ECUADOR.

#### References.

Pike et al. 1976, Endrődi 1966, 1985a, Krajcik 2005, 2012.

### Cyclocephala putrida

Taxon classificationAnimaliaColeopteraScarabaeidae

Burmeister, 1847

Cyclocephala putrida Burmeister, 1847: 51–52 [original combination].
**syn.** Cyclocephala tippmanni Endrődi, 1963: 329–331 [original combination].
Cyclocephala putrida Burmeister [synonymy by Endrődi 1964: 466].

#### Types.

Lectotype ♀ of *C. putrida* at MNHN (Dechambre 1991b). Invalid neotype of *C. putrida* at HNHM (Endrődi Collection) (Endrődi 1966). Endrődi (1963) did not clearly designate a holotype for *C. tippmanni*, and the type material of this species is not discussed in later works (Endrődi 1966).

#### Distribution.

ARGENTINA: Buenos Aires, Catamarca, Chaco, Córdoba, Entre Ríos, Mendoza, Salta, Santa Fe, Santiago del Estero, Tucumán. BOLIVIA: Cochabamba, Oruro, Santa Cruz. BRAZIL: Goiás, Mato Grosso, Minas Gerais, Paraná, Rio de Janeiro, Rio Grande do Norte, Rio Grande do Sul, Santa Catarina, São Paulo. CHILE: Bío Bío. FRENCH GUIANA: Cayenne. GUYANA: Upper Demerara-Berbice. PARAGUAY: Alto Paraná, Asunción. URUGUAY: Montevideo, Paysandú, Soriano, Tacuarembó. VENEZUELA: Apure.

#### References.

Burmeister 1847, Harold 1869b, Frenzel 1891, Bosq 1936, Arrow 1937b, Blackwelder 1944, Martínez 1968c, Pike et al. 1976, Vidal et al. 1979, Saenz and Morelli 1984, Endrődi 1963, 1966, 1969a, 1985a, Wiersema 1987, Morelli 1989, Dechambre 1980, 1991b, Dupuis 2009, Krajcik 2005, 2012, Breeschoten et al. 2013, Cherman et al. 2013, Barrantes and Castelo 2014, Maia et al. 2014.

### Cyclocephala pygidialis

Taxon classificationAnimaliaColeopteraScarabaeidae

Joly, 2000

Cyclocephala pygidialis Joly, 2000b: 521–526 [original combination].

#### Types.

Holotype ♂ at MIZA (Joly 2000b).

#### Distribution.

VENEZUELA: Guárico, Monagas.

#### References.

Joly 2000b, Krajcik 2005, 2012.

### Cyclocephala pygidiata

Taxon classificationAnimaliaColeopteraScarabaeidae

Dupuis, 1999

Cyclocephala pygidiata Dupuis, 1999: 183–184 [original combination].

#### Types.

Holotype ♂ at MNHN (Dupuis 1999).

#### Distribution.

COLOMBIA: Antioquia, Caldas, Chocó, Tolima, Valle del Cauca.

#### References.

Dupuis 1999, Joly 2010, Krajcik 2005, 2012, Yepes-Rodriguez et al. 2013, Gasca-Álvarez and Deloya 2016.

### Cyclocephala quadripunctata

Taxon classificationAnimaliaColeopteraScarabaeidae

Höhne, 1923

Cyclocephala quadripunctata Höhne, 1923b: 348–349 [original combination].

#### Types.

Lectotype ♂ at ZMHB (Endrődi 1966).

#### Distribution.

BOLIVIA: Santa Cruz. BRAZIL: Espírito Santo. COLOMBIA: Cauca, Chocó. ECUADOR: Guayas, Los Ríos, Morona Santiago, Napo, Pastaza. PANAMA: Darien, Former Canal Zone. PERU: Madre de Dios. VENEZUELA.

#### References.

Höhne 1923b, Arrow 1937b, Blackwelder 1944, Pike et al. 1976, Endrődi 1966, 1985a, Ervik et al. 1999, Ratcliffe 1992a, b, 2002a, 2003, 2008, Restrepo et al. 2003, Núñez-Avellaneda and Neita-Moreno 2009, Neita-Moreno 2011, Krajcik 2005, 2012, Ratcliffe et al. 2015, Gasca-Álvarez and Deloya 2016.

### Cyclocephala quatuordecimpunctata

Taxon classificationAnimaliaColeopteraScarabaeidae

Mannerheim, 1829

Cyclocephala quatuordecimpunctata Mannerheim, 1829: 52–53 [original combination].

#### Types.

Lectotype ♀ at ZMH (Endrődi 1966).

#### Distribution.

BRAZIL: Goiás, Mato Grosso, Minas Gerais, São Paulo.

#### References.

Mannerheim 1829, Dejean 1833, 1836b, Laporte 1840, Burmeister 1847, Harold 1869b, Arrow 1937b, Blackwelder 1944, Howden and Endrődi 1966, Pike et al. 1976, Endrődi 1966, 1969b, 1985a, Gottsberger 1986, 1988, 1989, 1992, 1999, Silberbauer-Gottsberger et al. 1997, 2001, Lunau 2002, Gottsberger and Silberbauer-Gottsberger 1988, 2006, Krajcik 2005, 2012, Breeschoten et al. 2013, Paulino-Neto 2014.

### Cyclocephala quercina

Taxon classificationAnimaliaColeopteraScarabaeidae

Burmeister, 1847

Cyclocephala quercina Burmeister, 1847: 54–55 [original combination].
**syn.** Cyclocephala obesa Burmeister, 1847: 59–60 [original combination].
Cyclocephala quercina Burmeister [synonymy by Dechambre and Duranton 2005: 67–68].

#### Types.

Lectotype ♂ of *C. quercina* at MNHN (Dechambre 1991b). Lectotype ♀ of *C. obesa* at MLUH (Endrődi 1966).

#### Distribution.

ECUADOR: Guayas. FRENCH GUIANA: Cayenne. GUYANA: Essequibo Islands-West Demerara, Pomeroon-Supenaam, Potaro-Siparuni. TRINIDAD AND TOBAGO: Trinidad. VENEZUELA: Monagas.

#### References.

Burmeister 1847, Reiche 1859, Harold 1869b, Arrow 1937b, Blackwelder 1944, Martínez 1969, Pike et al. 1976, Endrődi 1966, 1985a, Poole and Gentili 1996, Dechambre 1991b, 1999, Dechambre and Duranton 2005, Ponchel 2006, 2011, Krajcik 2005, 2012, Breeschoten et al. 2013.

#### Remarks.

Endrődi (1966, 1985a) reported specimens of *C. obesa* (= *C. quercina*) from Honduras (Islas de la Bahia), Costa Rica (Limón), and the United States (Arizona). These are the only records of *C. quercina* from these countries, and they are considered spurious or erroneous (Ratcliffe 2003). *Cyclocephala quercina* is a South American species (Ratcliffe 2003).

### Cyclocephala quisqueya

Taxon classificationAnimaliaColeopteraScarabaeidae

Joly, 1998

Cyclocephala quisqueya Joly, 1998: 50–54 [original combination].

#### Types.

Holotype ♂ at USNM (Joly 1998).

#### Distribution.

DOMINICAN REPUBLIC: Duarte, Elías Piña, La Vega, Monseñor Nouel, Puerto Plata, San Cristóbal, San Juan, Santiago.

#### References.

Joly 1998, Krajcik 2005, 2012, Ratcliffe and Cave 2015.

### Cyclocephala rangelana

Taxon classificationAnimaliaColeopteraScarabaeidae

Chapin, 1935

Cyclocephala rangelana Chapin, 1935a: 75 [original combination].
**syn.** Cyclocephala vidua Endrődi, 1966: 130, 331–332 [original combination].
Cyclocephala rangelana Chapin [synonymy by Ratcliffe and Cave 2015: 107].

#### Types.

Type of *C. rangelana* at USNM (Endrődi 1966). Holotype ♀ at *C. vidua* at BMNH (Endrődi 1966).

#### Distribution.

CUBA: Artemisa, Guantánamo, Pinar del Río. DOMINICAN REPUBLIC: Azua, Barahona, Distrito Nacional, Duarte, La Vega, Monseñor Nouel, Pedernales, Samana, Santiago, La Romana. HAITI: Grand Anse, Nippes, Ouest, Sud.

#### References.

Chapin 1935a, Arrow 1937a, Blackwelder 1944, Pike et al. 1976, Endrődi 1966, 1985a, Joly 1995b, Krajcik 2005, 2012, Ratcliffe and Cave 2015.

### Cyclocephala ratcliffei

Taxon classificationAnimaliaColeopteraScarabaeidae

Endrődi, 1977

Cyclocephala ratcliffei Endrődi, 1977b: 321 [original combination, new replacement name for Cyclocephala pereirai Endrődi].
**syn.** Cyclocephala pereirai Endrődi, 1969b: 31–32 [original combination, junior homonym of Cyclocephala pereirai (Martínez)].
Cyclocephala ratcliffei Endrődi [new replacement name by Endrődi 1977b: 321].

#### Types.

Holotype ♂ of *C. pereirai* at “Pereira Collection in Sao Paulo” (Endrődi 1969b). This is possibly referring to MZSP.

#### Distribution.

BRAZIL: Mato Grosso, São Paulo.

#### References.

Endrődi 1969b, Pike et al. 1976, Endrődi 1977b, 1985a, Krajcik 2005, 2012.

### Cyclocephala recta

Taxon classificationAnimaliaColeopteraScarabaeidae

Dupuis, 2008

Cyclocephala recta Dupuis, 2008: 117–122 [original combination].

#### Types.

Holotype ♂ at FDPC (Dupuis 2008).

#### Distribution.

COLOMBIA: Boyacá.

#### References.

Dupuis 2008, Krajcik 2012, Dupuis 2014, Gasca-Álvarez and Deloya 2016.

### Cyclocephala robusta

Taxon classificationAnimaliaColeopteraScarabaeidae

LeConte, 1863

Cyclocephala robusta LeConte, 1863: 79 [original combination].
Cyclocephala nigricollis Burmeister [synonymy by Horn 1871: 336].
Cyclocephala robusta LeConte [revalidated species status by Saylor 1945: 384].
Cyclocephala nigricollis Burmeister [synonymy by Endrődi 1964: 466].
Cyclocephala robusta LeConte [revalidated species status by Ratcliffe and Hoffman 2011].
**syn.** Cyclocephala nigricollis Burmeister, 1847: 54 [original combination].
Ochrosidia nigricollis (Burmeister) [new combination by Buchanan 1927: 167].
Cyclocephala robusta LeConte [synonymy by Saylor 1945: 384].
Cyclocephala nigricollis Burmeister [new status by Endrődi 1964: 466].
Cyclocephala nigricollis Burmeister [*nomen dubium* by Ratcliffe and Hoffman 2011: 136].
**syn.** Cyclocephala subvittata Brown, 1930: 5 [original combination].
Cyclocephala robusta LeConte [synonymy by Sanderson 1940: 380].
**syn.** Ochrosidia knobelae Brown, 1934: 23–24 [original combination].
Cyclocephala knobelae (Brown) [new combination by Arrow 1937b: 8, 11].
Cyclocephala robusta LeConte [synonymy by Ratcliffe and Cave 2017: 91].

#### Types.

Lectotype ♂ of *C. nigricollis* at MNHN (Dechambre 1991b). Invalid ♂ neotype of *C. nigricollis* at HNHM (Endrődi Collection) (Endrődi 1966, see discussion in Dechambre 1991b and Ratcliffe and Hoffman 2011). Type of *C. robusta* at MCZ (Endrődi 1966). Holotype ♂ of *C. subvittata* at CNC (Brown 1930). Types of *O. knobelae* at LEMQ (Endrődi 1966).

#### Distribution.

UNITED STATES: Alabama, Arkansas, Georgia, Kansas, Louisiana, Mississippi, Missouri, Oklahoma, South Carolina, Tennesee, Texas, Virginia.

#### References.

Burmeister 1847, Melsheimer et al. 1853, LeConte 1863, Gerstaecker 1865, Horn 1871, Henshaw 1885, Leng 1920, Buchanan 1927, Brown 1930, 1934, Leng and Mutchler 1933, Arrow 1937b, Brimley 1938, Sanderson 1940, Blackwelder 1944, Saylor 1945, Kirk 1970, Pike et al. 1976, 1977, Endrődi 1964, 1966, 1985a, Hardy 1991, Dechambre 1991b, Poole and Gentili 1996, Harpootlian 2001, Riley and Wolfe 2003, Smith 2003, 2009, Ratcliffe and Hoffman 2011, Krajcik 2005, 2012, Ratcliffe and Cave 2017.

### Cyclocephala rogerpauli

Taxon classificationAnimaliaColeopteraScarabaeidae

Moore, Branham & Cave, new replacement name

Cyclocephala nigra Dechambre, 1999: 15–16 [original combination, homonym of C. nigra (Endrődi)].

#### Types.

Holotype ♂ at MNHN (Dechambre 1999).

#### Distribution.

COLOMBIA: Sucre.

#### References.

Dechambre 1999, Restrepo et al. 2003, Krajcik 2005, 2012, Gasca-Álvarez and Deloya 2016.

#### Remarks.

Moore et al. (2015) synonymized the genus *Mimeoma* within *Cyclocephala*. This created a case of homonymy between *C. nigra* Dechambre and *C. nigra* (Endrődi) that went undetected at the time. Endrődi’s (1979) name has priority over Dechambre’s (1999) name. To rectify this homonym and to honor its original describer, we propose the *nomen novum Cyclocephala rogerpauli* as a **new replacement name** for the species described by Dechambre (1999).

### Cyclocephala rogezi

Taxon classificationAnimaliaColeopteraScarabaeidae

Dechambre, 1992

Cyclocephala rogezi Dechambre, 1992: 70 [original combination].

#### Types.

Holotype ♂ at MNHN (Dechambre 1992).

#### Distribution.

COLOMBIA: Chocó, Cauca, Valle del Cauca. PANAMA: Panamá.

#### References.

Dechambre 1992, Ratcliffe 2003, Restrepo et al. 2003, Krajcik 2005, 2012, Yepes-Rodriguez et al. 2013, Gasca-Álvarez and Deloya 2016.

### Cyclocephala rondoniana

Taxon classificationAnimaliaColeopteraScarabaeidae

Ratcliffe, 1992

Cyclocephala rondoniana Ratcliffe, 1992b: 185–187 [original combination].

#### Types.

Holotype ♂ at UNSM (Ratcliffe 1992b).

#### Distribution.

BRAZIL: Amazonas, Rondônia. FRENCH GUIANA.

#### References.

Ratcliffe 1992b, Küchmeister et al. 1998, Silberbauer-Gottsberger et al. 2001, Ponchel 2011, Krajcik 2005, 2012.

### Cyclocephala rorulenta

Taxon classificationAnimaliaColeopteraScarabaeidae

Höhne, 1923

Cyclocephala rorulenta Höhne, 1923b: 349–351 [original combination].

#### Types.

Lectotype ♂ at ZMHB (Endrődi 1966).

#### Distribution.

BRAZIL: Rio de Janeiro, Rio Grande do Sul, Santa Catarina. VENEZUELA.

#### References.

Höhne 1923b, Arrow 1937b, Blackwelder 1944, Pike et al. 1976, Endrődi 1966, 1985a, Ratcliffe 1992c, Krajcik 2005, 2012.

### Cyclocephala rotundipenis

Taxon classificationAnimaliaColeopteraScarabaeidae

Dupuis, 2009

Cyclocephala rotundipenis Dupuis, 2009: 29–32 [original combination].

#### Types.

Holotype ♂ at FDPC (Dupuis 2009).

#### Distribution.

COLOMBIA: Casanare.

#### References.

Dupuis 2009, Krajcik 2012, Gasca-Álvarez and Deloya 2016.

### Cyclocephala rubescens

Taxon classificationAnimaliaColeopteraScarabaeidae

Bates, 1891

Cyclocephala rubescens Bates, 1891: 31 [original combination].

#### Types.

Invalid neotype ♂ at HNHM (Endrődi Collection) (Endrődi 1966).

#### Distribution.

COLOMBIA: Antioquia. ECUADOR: Loja, Pichincha.

#### References.

Bates 1891, Bertkau 1892, Arrow 1937b, Blackwelder 1944, Pike et al. 1976, Endrődi 1964, 1966, 1967b, 1985a, Krajcik 2005, 2012, Breeschoten et al. 2013, Moore and Jameson 2013, Yepes-Rodriguez et al. 2013, Gasca-Álvarez and Deloya 2016.

#### Remarks.

*Cyclocephala rubescens* was reported from Panama (Croat 1997), but has not been recorded there since (Ratcliffe 2003).

### Cyclocephala rufa

Taxon classificationAnimaliaColeopteraScarabaeidae

Endrődi, 1967

Cyclocephala rufa Endrődi, 1967b: 83–84 [original combination].

#### Types.

Holotype ♂ at ZMHB (Endrődi 1967b).

#### Distribution.

COLOMBIA. VENEZUELA.

#### References.

Pike et al. 1976, Endrődi 1967b, 1985a, Restrepo et al. 2003, Krajcik 2005, 2012, Gasca-Álvarez and Deloya 2016.

### Cyclocephala rufescens

Taxon classificationAnimaliaColeopteraScarabaeidae

Endrődi, 1967

Cyclocephala rufescens Endrődi, 1967b: 86 [original combination].

#### Types.

Holotype ♂ at HNHM (Endrődi Collection) (Endrődi 1967b).

#### Distribution.

ECUADOR.

#### References.

Pike et al. 1976, Endrődi 1967b, 1975b, 1985a, Krajcik 2005, 2012.

### Cyclocephala ruficollis

Taxon classificationAnimaliaColeopteraScarabaeidae

Burmeister, 1847

Cyclocephala ruficollis Burmeister, 1847: 57 [original combination].

#### Types.

Lectotype ♀ at MLUH (Endrődi 1966).

#### Distribution.

COLOMBIA: Antioquia, Atlántico, Cesar, Chocó, Cundinamarca, Meta, Santander, Tolima, Valle del Cauca.

#### References.

Burmeister 1847, Harold 1869b, Arrow 1937b, Blackwelder 1944, Pike et al. 1976, Endrődi 1964, 1966, 1985a, Posada Ochoa 1989, Pardo-Locarno et al. 1995, Restrepo et al. 2003, Krajcik 2005, 2012, López-García et al. 2015, Gasca-Álvarez and Deloya 2016.

### Cyclocephala rufonigra

Taxon classificationAnimaliaColeopteraScarabaeidae

Demay, 1838

Cyclocephala rufonigra Demay, 1838: 23 [original combination].

#### Distribution.

GUYANA.

#### References.

Demay 1838, Burmeister 1847, Harold 1869b, Arrow 1937b, Blackwelder 1944, Pike et al. 1976, Endrődi 1966, 1985a, Krajcik 2005, 2012.

#### Remarks.

The identity of *C. rufonigra* is ambiguous. No additional specimens identified as *C. rufonigra* have been reported since this species' original description, and the type material is apparently lost (Endrődi 1966, 1985a).

### Cyclocephala rufovaria

Taxon classificationAnimaliaColeopteraScarabaeidae

Arrow, 1911

Cyclocephala rufovaria Arrow, 1911: 173 [original combination].

#### Types.

Type at BMNH (Endrődi 1966).

#### Distribution.

BRAZIL. COLOMBIA: Amazonas. ECUADOR. FRENCH GUIANA. PERU.

#### References.

Arrow 1911, 1937b, Blackwelder 1944, Pike et al. 1976, Endrődi 1966, 1985a, Dupuis 2006, Ponchel 2006, 2011, Krajcik 2005, 2012, Otavo et al. 2013, Ratcliffe et al. 2015, Gasca-Álvarez and Deloya 2016.

### Cyclocephala rustica

Taxon classificationAnimaliaColeopteraScarabaeidae

(Olivier, 1789)

Melolontha rustica Olivier, 1789: 27 [original combination].
Cyclocephala rustica (Olivier) [new combination by Burmeister 1847: 70].
**syn.** Cyclocephala rustica municipalis Höhne, 1923b: 365 [original combination].
Cyclocephala rustica var. municipalis Höhne [new infrasubspecific status by Arrow 1937b: 15].
Cyclocephala rustica municipalis Höhne [revalidated subspecies status by Endrődi 1985a: 35].
Cyclocephala rustica (Olivier) [synonymy by Ratcliffe et al. 2013: 597].

#### Types.

Invalid neotype of *M. rustica* at HNHM (Endrődi Collection) (Endrődi 1966). Lectotype of *C. rustica municipalis* at ZMHB (Endrődi 1966).

#### Distribution.

BRAZIL: Amazonas, Bahia, Mato Grosso, Pará, São Paulo. COLOMBIA: Casanare, Cundinamarca, Meta. FRENCH GUIANA: Cayenne, Kourou, Régina. PERU. SURINAME. TRINDAD AND TOBAGO: Trinidad. VENEZUELA: Capital District, Carabobo.

#### References.

Olivier 1789, Burmeister 1847, Harold 1869b, Höhne 1923b, Arrow 1937b, Blackwelder 1944, Pike et al. 1976, Endrődi 1963, 1966, 1985a, Pellmyr 1985, Ratcliffe and Morón 1997, Restrepo et al. 2003, Maia and Schlindwein 2006, Ponchel 2006, 2011, Krajcik 2005, 2012, Breeschoten et al. 2013, Moore and Jameson 2013, Gibernau 2015b, López-García et al. 2015, Ratcliffe et al. 2013, 2015, Gasca-Álvarez and Deloya 2016, Peck 2016.

#### Remarks.

*Cyclocephala rustica* was reported from Veracruz, Mexico (Höhne 1923b, Endrődi 1966, 1985a, Ratcliffe and Morón 1997) and Guadeloupe (Olivier 1789, Endrődi 1966, 1985a). *Cyclocephala rustica* has not been recorded from these areas again and is likely a South American species (Ratcliffe et al. 2013, Ratcliffe and Cave 2015).

### Cyclocephala saltini

Taxon classificationAnimaliaColeopteraScarabaeidae

Ratcliffe, 2008

Cyclocephala saltini Ratcliffe, 2008: 238–240 [original combination].

#### Types.

Holotype ♂ at UNSM (Ratcliffe 2008).

#### Distribution.

PERU: Huánuco.

#### References.

Ratcliffe 2008, Krajcik 2012, Ratcliffe et al. 2015.

### Cyclocephala sanguinicollis

Taxon classificationAnimaliaColeopteraScarabaeidae

Burmeister, 1847

Cyclocephala sanguinicollis Burmeister, 1847: 49–50 [original combination].
Homochromina sanguinicollis (Burmeister, 1847) [new combination by Casey 1915: 165].
Cyclocephala sanguinicollis Burmeister [revised combination by Arrow 1937b: 8, 15].
**syn.** Homochromina divisa Casey, 1915: 163 [original combination].
Cyclocephala divisa (Casey) [new combination by Arrow 1937b: 8, 10].
Cyclocephala sanguinicollis Burmeister [synonymy by Endrődi 1966: 301].
**syn.** Homochromina politicauda Casey, 1915: 164 [original combination].
Cyclocephala politicauda (Casey) [new combination by Arrow 1937b: 8, 14].
Cyclocephala sanguinicollis Burmeister [synonymy by Endrődi 1966: 301].

#### Types.

Lectotype ♀ of *C. sanguinicollis* at MLUH (Endrődi 1966). The types of *H. divisa* and *H. politicauda* are at USNM (Endrődi 1966).

#### Distribution.

BELIZE: Toledo. BRAZIL. COLOMBIA: Cauca. COSTA RICA: Alajuela, Cartago, Guanacaste, Limón, Puntarenas, San José. ECUADOR: Bolívar, Cañar. GUATEMALA: Alta Verapaz, Baja Verapaz, Huehuetenango, Izabal, San Marcos, Zacapa. HONDURAS: Atlántida, Colón, Comayagua, Cortés, El Paraíso, La Paz, Lempira, Olancho, Santa Bárbara, Yoro. MEXICO: Chiapas, Hidalgo, Nayarit, Oaxaca, Puebla, San Luis Potosí, Tabasco, Veracruz, Yucatán. NICARAGUA: Jinotega, Matagalpa, Río San Juan. PANAMA: Chiriquí. VENEZUELA: Carabobo, Vargas.

#### References.

Burmeister 1847, Reiche 1859, Harold 1869b, Anonymous 1879, Bates 1888, Casey 1915, Arrow 1937b, Blackwelder 1944, Pike et al. 1976, Endrődi 1966, 1967b, 1985a, Morón et al. 1985, Thomas 1993, Lobo and Morón 1993, Morón 1979, 1994, Ratcliffe and Morón 1997, Sanchez Soto 1997, Abarca and Quesada 1997, Vargas and Abarca 1998, Carrillo-Ruiz and Morón 2003, Ratcliffe 2003, Restrepo et al. 2003, Ratcliffe and Cave 2006, Smith 2003, 2009, Krajcik 2005, 2012, Breeschoten et al. 2013, Ratcliffe et al. 2013, Gasca-Álvarez and Deloya 2016.

#### Remarks.

The South American and Panamanian records for *C. sanguinicollis* are potentially based on erroneous identifications of a similar species (possibly *C. mutata*). These data need to be reevaluated (Ratcliffe et al. 2013).

### Cyclocephala santaritae

Taxon classificationAnimaliaColeopteraScarabaeidae

Ratcliffe, 1992

Cyclocephala santaritae Ratcliffe, 1992a: 229–230 [original combination].

#### Types.

Holotype ♂ at UNSM (Ratcliffe 1992a).

#### Distribution.

COLOMBIA: Amazonas, Chocó. ECUADOR: Napo. PANAMA: Colón, Panamá, San Blas.

#### References.

Ratcliffe 1992a, 2002a, 2003, 2008, Núñez-Avellaneda and Neita-Moreno 2009, Neita-Moreno 2011, Krajcik 2005, 2012, Moore and Jameson 2013, Otavo et al. 2013, Gasca-Álvarez and Deloya 2016.

### Cyclocephala sarahae

Taxon classificationAnimaliaColeopteraScarabaeidae

Ratcliffe, 1992

Cyclocephala sarahae Ratcliffe, 1992b: 187–189 [original combination].

#### Types.

Holotype ♂ at UNSM (Ratcliffe 1992b).

#### Distribution.

BRAZIL: Amazonas.

#### References.

Ratcliffe 1992b, Joly 2009, Krajcik 2005, 2012.

### Cyclocephala sardadebiae

Taxon classificationAnimaliaColeopteraScarabaeidae

Dechambre & Duranton, 2005

Cyclocephala sardadebiae Dechambre & Duranton, 2005: 68–69 [original combination].

#### Types.

Holotype ♂ at MNHN (Dechambre and Duranton 2005).

#### Distribution.

FRENCH GUIANA: Kourou, Maripasoula, Régina, Roura, Saül, St.-Élie, St.-Laurent du Maroni, St.-Georges.

#### References.

Dechambre and Duranton 2005, Ponchel 2011, Krajcik 2012.

### Cyclocephala sarpedon

Taxon classificationAnimaliaColeopteraScarabaeidae

Ratcliffe, 1992

Cyclocephala sarpedon Ratcliffe, 1992b: 188 [original combination].

#### Types.

Holotype ♂ at UNSM (Ratcliffe 1992b).

#### Distribution.

BRAZIL: Amazonas, Mato Grosso, Rondônia. SURINAME: Sipaliwini. VENEZUELA: Aragua.

#### References.

Ratcliffe 1992b, Küchmeister et al. 1998, Silberbauer-Gottsberger et al. 2001, Krajcik 2005, 2012.

### Cyclocephala scarabaeina

Taxon classificationAnimaliaColeopteraScarabaeidae

(Gyllenhal, 1817)

Melolontha scarabaeina Gyllenhal, 1817b: 103–104 [original combination].
Peltonotus scarabaeinus (Gyllenhal) [new combination by Burmeister 1847: 75].
Cyclocephala scarabaeina (Gyllenhal) [new combination by Burmeister 1847: 521].

#### Types.

Lectotype ♂ at UUZM (Endrődi 1966, see also Wallin 2001).

#### Distribution.

PERU: Madre de Dios.

#### References.

Gyllenhal 1817b, Burmeister 1847, Harold 1869b, Arrow 1937b, Blackwelder 1944, Pike et al. 1976, Endrődi 1966, 1985a, Wallin 2001, Krajcik 2005, 2012, Ratcliffe et al. 2015.

#### Remarks.

The type locality of *C. scarabaeina* is “India orientalis” (Gyllenhal 1817b, Burmeister 1847). These data are erroneous, and *C. scarabaeina* is only known from Peru (Endrődi 1966, 1985a, Ratcliffe et al. 2015).

### Cyclocephala schmitzorum

Taxon classificationAnimaliaColeopteraScarabaeidae

Ratcliffe, 1992

Cyclocephala schmitzorum Ratcliffe, 1992b: 189 [original combination].

#### Types.

Holotype ♂ at UNSM (Ratcliffe 1992b).

#### Distribution.

BRAZIL: Amazonas, Rondônia. SURINAME: Sipaliwini.

#### References.

Ratcliffe 1992b, Krajcik 2005, 2012.

### Cyclocephala seditiosa

Taxon classificationAnimaliaColeopteraScarabaeidae

LeConte, 1863

Cyclocephala seditiosa LeConte, 1863: 79 [original combination].
Ochrosidia (Ochrosidia) seditiosa (LeConte) [new combination and new subgeneric classification by Casey 1915: 142, 158].
Cyclocephala seditiosa LeConte [revised combination and removal of subgeneric classification by Arrow 1937b: 8, 15].

#### Types.

Type at MCZ (Endrődi 1966).

#### Distribution.

UNITED STATES: Alabama, Florida, Mississippi.

#### References.

LeConte 1863, Gerstaecker 1865, Harold 1869b, Horn 1871, Henshaw 1885, Casey 1915, Arrow 1937b, Blackwelder 1944, Saylor 1945, Richmond 1962, Howden and Endrődi 1966, Pike et al. 1976, Gordon and Anderson 1981, Endrődi 1966, 1969b, 1985a, Hardy 1991, Poole and Gentili 1996, Peck and Thomas 1998, Harpootlian 2001, Buss 2006, Smith 2003, 2009, Krajcik 2005, 2012, Ratcliffe and Cave 2017.

### Cyclocephala sexpunctata

Taxon classificationAnimaliaColeopteraScarabaeidae

Laporte, 1840

Cyclocephala sexpunctata Laporte, 1840: 125 [original combination].
**syn.** Cyclocephala pubescens Erichson, 1847a: 96 [original combination].
Cyclocephala sexpunctata Laporte [synonymy by Endrődi 1966: 306].
**syn.** Cyclocephala pubescens spermophila Ohaus, 1910: 671 [original combination].
Cyclocephala pubescens var. spermophila Ohaus [new infrasubspecific status by Arrow 1937b: 15].
Cyclocephala sexpunctata var. spermophila Ohaus [new infrasubspecific status by Endrődi 1966: 306].
Cyclocephala sexpunctata ab. spermophila Ohaus [revised infrasubspecific status by Endrődi 1985a: 82].
**syn.** Cyclocephala pubescens nigripes Höhne, 1923b: 372–373 [original combination].
Cyclocephala pubescens var. nigripes Höhne [new infrasubspecific status by Arrow 1937b: 15].
Cyclocephala sexpunctata ab. nigripes Höhne [revised infrasubspecific status by Endrődi 1966: 306].
**syn.** Cyclocephala lucida Burmeister, 1847: 67 [original combination].
Stigmalia lucida (Burmeister) [new combination by Casey 1915: 120].
Cyclocephala sexpunctata Laporte [synonymy by Endrődi 1964: 466].
Cyclocephala sexpunctata ab. lucida Burmeister [new infrasubspecific status by Endrődi 1966: 306].
**syn.** Stigmalia circulifer Casey, 1915: 121 [original combination].
Cyclocephala lucida Burmeister [synonymy by Arrow 1937b: 12].
Cyclocephala sexpunctata Laporte [synonymy by Endrődi 1966: 306].
**syn.** Stigmalia costaricana Casey, 1915: 121 [original combination].
Cyclocephala costaricana (Casey) [new combination by Arrow 1937b: 8, 9].
Cyclocephala sexpunctata Laporte [synonymy by Endrődi 1966: 306].
**syn.** Stigmalia discoidalis Casey, 1915: 120 [original combination].
Cyclocephala lucida var. discoidalis (Casey) [new combination and new infrasubspecific status by Arrow 1937b: 12].
Cyclocephala sexpunctata Laporte [synonymy by Endrődi 1966: 306].
**syn.** Stigmalia triangulifer Casey, 1915: 120 [original combination].
Cyclocephala lucida Burmeister [synonymy by Arrow 1937b: 12].
Cyclocephala sexpunctata Laporte [synonymy by Endrődi 1966: 306].

#### Types.

Type ♂ of *C. sexpunctata* at MNHN (Endrődi 1966). Lectotype ♂ of *C. pubescens* at ZMHB (Endrődi 1966). Lectotype ♂ of *C. lucida* at MLUH (Endrődi 1966). Lectotype ♂ of *C. pubescens nigripes* at ZMHB (Endrődi 1966). Types of *C. pubescens spermophila* at SDEI (Endrődi 1966). Types of the Casey synonyms at USNM (Endrődi 1966).

#### Distribution.

BOLIVIA. BRAZIL. COLOMBIA: Antioquia, Boyacá, Cauca, Cundinamarca, Meta, Santander, Tolima, Valle de Cauca. COSTA RICA: Alajuela, Cartago, Guanacaste, Heredia, Puntarenas, San José. ECUADOR. FRENCH GUIANA: Cayenne. GUATEMALA: Alta Verapaz, Baja Verapaz, Escuintla, Huehuetenango, Izabal, Quetzaltenango, Quiché, San Marcos, Suchitepéquez, Zacapa. HONDURAS: Atlántida, Colón, Comayagua, Cortés, El Paraíso, La Paz, Lempira, Olancho, Santa Bárbara, Yoro. MEXICO: Chiapas, Colima, Estado de México, Guerrero, Hidalgo, Jalisco, Oaxaca, Puebla, Veracruz. NICARAGUA: Jinotega, Matagalpa, Río San Juan. PANAMA: Bocas del Toro, Chiriquí, Coclé, Panamá. PERU: Madre de Dios. VENEZUELA: Capital District, Mérida.

#### References.

Laporte 1840, Erichson 1847a, Marschall 1857, Reiche 1859, Harold 1869b, Bates 1888, Ohaus 1910, Casey 1915, Arrow 1937b, Blackwelder 1944, Pike et al. 1976, Martínez 1967, 1978b, Dechambre 1992, Endrődi 1964, 1966, 1985a, Valerio 1988, Goldwasser 1987, 2000, Young 1992, Seres and Ramírez 1995, Croat 1997, Morón 1997, Ratcliffe and Morón 1997, Gibernau et al. 1999, Silberbauer-Gottsberger et al. 2001, Ratcliffe 1992a, 2002a, 2003, Restrepo et al. 2003, García-Robledo et al. 2005, Young and Le Tirant 2005, Neita-Moreno and Gaigl 2008, Útima and Vallejo 2008, Ponchel 2011, Moore and Jameson 2013, Breeschoten et al. 2013, Deloya et al. 2014a, López-García et al. 2015, Moore et al. 2015, Ratcliffe et al. 2015, Gasca-Álvarez and Deloya 2016, Villalobos-Moreno et al. 2016.

### Cyclocephala setosa

Taxon classificationAnimaliaColeopteraScarabaeidae

Burmeister, 1847

Cyclocephala setosa Burmeister, 1847: 38–39 [original combination].

#### Types.

Lectotype ♂ at MLUH (Endrődi 1966).

#### Distribution.

BRAZIL: Minas Gerais, São Paulo.

#### References.

Burmeister 1847, Harold 1869b, Arrow 1937b, Blackwelder 1944, Martínez 1954, Pike et al. 1976, Endrődi 1966, 1985a, Ratcliffe 1992a, Joly 2010, Krajcik 2005, 2012.

### Cyclocephala signaticollis

Taxon classificationAnimaliaColeopteraScarabaeidae

Burmeister, 1847

Cyclocephala signaticollis Burmeister, 1847: 63–64 [original combination].

#### Types.

Lectotype ♀ at MNHN (Dechambre 1991b). Invalid neotype ♂ at HNHM (Endrődi Collection) (Endrődi 1966).

#### Distribution.

ARGENTINA: Buenos Aires, Córdoba, Entre Ríos, La Pampa, Misiones, Santa Fe. AUSTRALIA: New South Wales. BOLIVIA: Cochabamba, La Paz, Santa Cruz. BRAZIL: Paraná, Rio Grande do Sul, Santa Catarina, São Paulo. COLOMBIA: Cauca, Valle del Cauca. FRENCH GUIANA: Mana. URUGUAY: Canelones, Maldonado, Montevideo, Paysandú, San José. VENEZUELA: Mérida.

#### References.

Burmeister 1847, Reiche 1859, Harold 1869b, Frenzel 1891, Tremoleras 1910, Arrow 1937b, Blackwelder 1944, Carne 1956, 1957, Gavotto 1964, Martínez 1968c, San Martín 1968, Britton 1970, Pike et al. 1976, Saenz and Morelli 1984, Endrődi 1966, 1985a, Dennis and Knutson 1988, Dechambre 1991b, Morelli 1989, 1991, Anonymous 1993, Thomas 1993, Manetti et al. 1994, 1996, Padin et al. 1996, Mondino et al. 1997, Castelo and Capurro 2000, Reboredo and Camino 2000, Restrepo et al. 2003, Castelo and Lazzari 2004, Ghys and Favero 2004, Mauco and Favero 2004, Berón and Diaz 2005, Camino and Reboredo 2005, Castelo et al. 2006, Jackson and Klein 2006, Consolo et al. 2010, Castelo and Corley 2010, Grossi et al. 2011, Krajcik 2005, 2012, Camino and Achinelly 2012, Groba and Castelo 2012, Castelo and Crespo 2012, Nussembaum and Lecuona 2012, Breeschoten et al. 2013, Martínez et al. 2013, Crespo and Castelo 2008, 2009, 2010, 2012, 2013, Ratcliffe et al. 2013, Barrantes and Castelo 2014, Crespo et al. 2011, 2015, Gasca-Álvarez and Deloya 2016, Beehag et al. 2016, Ramírez and Alonso 2016.

#### Remarks.

*Cyclocephala signaticollis* was reported from Mexico based on a single specimen (Endrődi 1966, 1985a). No further specimens of *C. signaticollis* have been reported from Mexico, and it is likely that this species is restricted to South America (Ratcliffe et al. 2013), except for Australia where it is adventive.

### Cyclocephala signatoides

Taxon classificationAnimaliaColeopteraScarabaeidae

Höhne, 1923

Cyclocephala signatoides Höhne, 1923b: 346–348 [original combination].
Mimeoma signatoides (Höhne) [new combination by Arrow 1937b: 5].
Cyclocephala signatoides Höhne [revised combination by Moore et al. 2015: 898].

#### Types.

Lectotype ♂ at ZMHB (Endrődi 1966).

#### Distribution.

BOLIVIA: Cochabamba. BRAZIL: Amazonas, Bahia. COLOMBIA: Meta. ECUADOR. FRENCH GUIANA: Cayenne, St.-Laurent du Maroni, St.-Georges. GUAYANA. PERU: Cusco. VENEZUELA.

#### References.

Höhne 1923b, Arrow 1937b, Blackwelder 1944, Gruner 1971, Pike et al. 1976, Endrődi 1966, 1985a, Restrepo et al. 2003, Krajcik 2005, 2012, Breeschoten et al. 2013, Moore and Jameson 2013, Moore et al. 2015, Ratcliffe et al. 2013, 2015, Ratcliffe and Cave 2015, Gasca-Álvarez and Deloya 2016.

#### Remarks.

Some authors reported *C. signatoides* from Cuba and Mexico (Höhne 1923b, Arrow 1937b, Blackwelder 1944, Endrődi 1966, 1985a). These data are erroneous, and *C. signatoides* is a South American species (Ratcliffe et al. 2013, Ratcliffe and Cave 2015).

### Cyclocephala similis

Taxon classificationAnimaliaColeopteraScarabaeidae

Dechambre, 1980

Cyclocephala similis Dechambre, 1980: 47–49 [original combination].

#### Types.

Holotype ♂ at MNHN (Dechambre 1980).

#### Distribution.

FRENCH GUIANA: Saül.

#### References.

Dechambre 1980, Endrődi 1985a, Ponchel 2011, Krajcik 2005, 2012.

### Cyclocephala simillima

Taxon classificationAnimaliaColeopteraScarabaeidae

Dechambre, 1999

Cyclocephala simillima Dechambre, 1999: 17 [original combination].

#### Types.

Holotype ♂ at MNHN (Dechambre 1999).

#### Distribution.

VENEZUELA: Bolívar.

#### References.

Dechambre 1999, Krajcik 2005, 2012.

### Cyclocephala simulatrix

Taxon classificationAnimaliaColeopteraScarabaeidae

Höhne, 1923

Cyclocephala simulatrix Höhne, 1923b: 372 [original combination].

#### Types.

Lectotype ♂ at ZMHB (Endrődi 1966).

#### Distribution.

BOLIVIA: La Paz. BRAZIL: Amazonas, Pará. FRENCH GUIANA: Kourou, St.-Georges. PARAGUAY. PERU: Madre de Dios, Mariscal Ramón Castilla, Pasco, San Martín. TRINIDAD AND TOBAGO: Trinidad.

#### References.

Höhne 1923b, Arrow 1937b, Blackwelder 1944, Gruner 1971, Pike et al. 1976, Dechambre 1979a, Endrődi 1966, 1985a, Silberbauer-Gottsberger et al. 2001, Andreazze and da Silva Motta 2002, Gibernau and Barabé 2002, Krajcik 2005, 2012, Breeschoten et al. 2013, Ponchel 2006, 2011, 2015, Ratcliffe et al. 2015.

#### Remarks.

A single specimen of *C. simulatrix* was reported from Costa Rica (Endrődi 1966, 1985a). No further specimens have been reported from Costa Rica or Panama and it is likely that *C. simulatrix* is restricted to South America (Ratcliffe 2003).

### Cyclocephala sinaloae

Taxon classificationAnimaliaColeopteraScarabaeidae

Howden & Endrődi, 1966

Cyclocephala sinaloae Howden & Endrődi, 1966: 298–299 [original combination].

#### Types.

Holotype ♂ at CNC (Howden and Endrődi 1966).

#### Distribution.

MEXICO: Baja California, Durango, Jalisco, Nayarit, Sinaloa, Sonora.

#### References.

Howden and Endrődi 1966, Pike et al. 1976, Endrődi 1985a, Ratcliffe and Morón 1997, Navarrete-Heredia et al. 2001, García et al. 2009, Smith 2003, 2009, Krajcik 2005, 2012, Lugo-García et al. 2012, Lugo et al. 2013, Morón et al. 2014.

### Cyclocephala sinuosa

Taxon classificationAnimaliaColeopteraScarabaeidae

Höhne, 1923

Cyclocephala sinuosa Höhne, 1923b: 369–370 [original combination].

#### Types.

Lectotype ♂ at ZMHB (Endrődi 1966).

#### Distribution.

COLOMBIA: Meta.

#### References.

Höhne 1923b, Arrow 1937b, Martínez 1967, Blackwelder 1944, Pike et al. 1976, Endrődi 1964, 1966, 1985a, Restrepo et al. 2003, Breeschoten et al. 2013, Gasca-Álvarez and Deloya 2016.

### Cyclocephala spangleri

Taxon classificationAnimaliaColeopteraScarabaeidae

Joly, 2000

Cyclocephala spangleri Joly, 2000a: 333–338 [original combination].

#### Types.

Holotype ♂ at USNM (Joly 2000a).

#### Distribution.

VENEZUELA: Apure, Guárico.

#### References.

Joly 2000a, Krajcik 2005, 2012.

### Cyclocephala sparsa

Taxon classificationAnimaliaColeopteraScarabaeidae

Arrow, 1902

Cyclocephala sparsa Arrow, 1902: 141 [original combination].
**syn.** Cyclocephala landini Endrődi, 1964: 445–447 [original combination].
Cyclocephala sparsa Arrow [synonymy by Ratcliffe and Delgado-Castillo 1990: 47].
**syn.** Cyclocephala virkkii Howden & Endrődi, 1966: 301 [original combination].
Cyclocephala sparsa Arrow [synonymy by Ratcliffe and Delgado-Castillo 1990: 47].

#### Types.

Type of *C. sparsa* at BMNH (Endrődi 1966). Holotype ♂ of *C. landini* at USNM (Endrődi 1964). Holotype ♂ of *C. virkkii* at CNC (Howden and Endrődi 1966).

#### Distribution.

BELIZE: Cayo, Orange Walk. COSTA RICA: Cartago, Guanacaste, Heredia, Limón, San José. EL SALVADOR: Ahuachapán, Cabañas, Morazán, San Salvador. GUATEMALA: Alta Verapaz, Izabal, Jalapa, Jutiapa, Petén. HONDURAS: Atlántida, Cortés, Lempira, Olancho, Santa Bárbara, Yoro. MEXICO: Campeche, Chiapas, Estado de México, Guerrero, Hidalgo, Jalisco, Michoacán, Morelos, Nayarit, Oaxaca, Quintana Roo, Veracruz, Yucatán. PANAMA: Former Canal Zone, Panamá.

#### References.

Arrow 1902, 1937b, Blackwelder 1944, Howden and Endrődi 1966, Martínez 1975b, Pike et al. 1976, Morón 1979, Endrődi 1964, 1966, 1985a, Bawa et al. 1985a, b, Schatz 1985, 1987, Ratcliffe and Delgado-Castillo 1990, Murray 1993, Thomas 1993, Lobo and Morón 1993, Kress and Beach 1994, Ratcliffe and Morón 1997, Morón et al. 1996, 1998, Joly 2000a, Ratcliffe 1992a, 2002a, 2003, Ratcliffe and Cave 2006, Smith 2003, 2009, Krajcik 2005, 2012, García-López et al. 2013, Ratcliffe et al. 2013, Deloya et al. 2014a, 2016.

### Cyclocephala spilopyga

Taxon classificationAnimaliaColeopteraScarabaeidae

Erichson, 1847

Cyclocephala spilopyga Erichson, 1847a: 96 [original combination].

#### Types.

Lectotype ♂ at ZMHB (Endrődi 1966).

#### Distribution.

BOLIVIA: Cochabamba, La Paz, Santa Cruz. BRAZIL: São Paulo. COLOMBIA: Antioquia, Huila, Tolima, Santander, Valle del Cauca. ECUADOR: Morona-Santiago, Napo. PERU: Cusco.

#### References.

Erichson 1847a, Harold 1869b, Arrow 1937b, Blackwelder 1944, Pike et al. 1976, Endrődi 1966, 1985a, Dupuis 1999, Restrepo et al. 2003, Joly 2010, Krajcik 2005, 2012, Yepes-Rodriguez et al. 2013, Ratcliffe et al. 2015, López-García et al. 2015, Gasca-Álvarez and Deloya 2016.

### Cyclocephala stictica

Taxon classificationAnimaliaColeopteraScarabaeidae

Burmeister, 1847

Cyclocephala stictica Burmeister, 1847: 66–67 [original combination].
Cyclocephala (Cyclocephala) stictica Burmeister [new subgeneric classification by Casey 1915: 138].
Cyclocephala stictica Burmeister [removal of subgeneric classification by Arrow 1937b: 8, 16].
**syn.** Cyclocephala stictica bilineata Höhne, 1923b: 357 [original combination].

Cyclocephala stictica var. bilineata Höhne [new infrasubspecific status by Arrow 1937b: 16].
Cyclocephala stictica ab. bilineata Höhne [revised infrasubspecific status by Endrődi 1966: 314].
Cyclocephala stictica Burmeister [synonymy by Ratcliffe 2002a: 31].
**syn.** Cyclocephala microspila Bates, 1888: 301 [original combination].
Cyclocephala stictica Burmeister [synonymy by Endrődi 1966: 314].
**syn.** Cyclocephala sexnotata Burmeister, 1847: 67 [original combination].
Cyclocephala stictica Burmeister [synonymy by Endrődi 1964: 466].

#### Types.

Lectotype ♂ of *C. stictica* and lectotype ♂ of *C. sexnotata* at MLUH (Endrődi 1966). Type of *C. microspila* at BMNH (Endrődi 1966). Lectotype of *C. stictica bilineata* at ZMHB (Endrődi 1966).

#### Distribution.

BELIZE: Cayo, Orange Walk, Stann Creek, Toledo. BOLIVIA: Cochabamba, Santa Cruz. BRAZIL: Bahia, Mato Grosso, Santa Catarina. COLOMBIA: Amazonas, Antioquia, Bolívar, Caquetá, Cauca, Chocó, Meta, Valle del Cauca. COSTA RICA: Alajuela, Cartago, Guanacaste, Heredia, Limón, Puntarenas, San José. ECUADOR: Cañar, Guayas. EL SALVADOR: Cabañas, Chalatenango, La Libertad, Morazán, San Vicente. FRENCH GUIANA: Cayenne. GUATEMALA: Alta Verapaz, Baja Verapaz, Chiquimula, Huehuetenango, Izabal, Petén, Quiché, Suchitepéquez, Zacapa. HONDURAS: Atlántida, Comayagua, Copán, Cortés, El Paraíso, Francisco Morazán, Gracias a Dios, Lempira, Ocotepeque, Olancho, Santa Bárbara, Yoro. MEXICO: Chiapas, Estado de México, Guerrero, Hidalgo, Jalisco, Michoacán, Morelos, Nayarit, Oaxaca, Puebla, San Luis Potosí, Tabasco, Tamaulipas, Veracruz. NICARAGUA: Granada, Jinotega, Managua, Matagalpa, RAA Norte, Río San Juan (Endrődi 1966,). PANAMA: Chiriquí, Coclé, Colón, Darien, Former Canal Zone, Panamá, San Blas, Veraguas. PERU. VENEZUELA.

#### References.

Burmeister 1847, Reiche 1859, Harold 1869b, Anonymous 1879, Bates 1888, Casey 1915, Höhne 1923b, Arrow 1937b, Blackwelder 1944, Barrera 1969, Pike et al. 1976, Dechambre 1979a, Bullock 1981, Endrődi 1964, 1966, 1985a, Villalta 1988, Thomas 1993, Lobo and Morón 1993, Maes et al. 1997, Morón 1994, 1997, Ratcliffe and Morón 1997, Ratcliffe 2002a, 2003, Restrepo et al. 2003, Pardo-Locarno et al. 2003, 2005a, Ratcliffe and Cave 2006, Núñez-Avellaneda and Rojas-Robles 2008, Neita-Moreno 2011, Ponchel 2011, Krajcik 2005, 2012, Morón and Márquez 2012, Breeschoten et al. 2013, García-López et al. 2013, Pardo-Locarno 2013, Yepes-Rodriguez et al. 2013, Deloya et al. 2014a, Ratcliffe et al. 2013, 2015, Gasca-Álvarez and Deloya 2016, Romero-López and Morón 2017.

### Cyclocephala stockwelli

Taxon classificationAnimaliaColeopteraScarabaeidae

Ratcliffe, 2003

Cyclocephala stockwelli Ratcliffe, 2003: 61, 71, 75, 78, 210–212 [original combination].

#### Types.

Holotype ♂ at UNSM (Ratcliffe 2003).

#### Distribution.

COSTA RICA: Heredia, Puntarenas. PANAMA: Panamá.

#### References.

Ratcliffe 2003, Krajcik 2005, 2012, García-López et al. 2013.

### Cyclocephala striata

Taxon classificationAnimaliaColeopteraScarabaeidae

Endrődi, 1963

Cyclocephala striata Endrődi, 1963: 326–328 [original combination].
**syn.** Cyclocephala fusiformis Chapin, 1932: 287 [original combination].
Cyclocephala striata Endrődi [synonymy by Ratcliffe and Cave 2015: 109].
**syn.** Cyclocephala striata hatiensisEndrődi 1963: 328 [original combination].
Cyclocephala striata Endrődi [synonymy by Ratcliffe and Cave 2015: 109].

#### Types.

Type of *C. fusiformis* at USNM (Endrődi 1966). Holotype ♂ of *C. striata striata* at ZSMC (Endrődi 1966). Holotype of *C. striata hatiensis* at HNHM (Endrődi Collection) (Endrődi 1966).

#### Distribution.

CUBA: Isla de la Juventad. DOMINICAN REPUBLIC: Barahona, Distrito Nacional, Duarte, El Seibo, Hato Mayor, Independencia, La Altagracia, La Vega, María Trinidad Sánchez, Monseñor Nouel, Monte Plata, Pedernales, Peravia, Puerto Plata, Salcedo, Samana, San Cristóbal, San José de Ocoa, San Juan, Santiago, Valverde. HAITI: Grand Anse, Ouest. JAMAICA: Portland, St. Mary.

#### References.

Chapin 1932, Arrow 1937b, Blackwelder 1944, Pike et al. 1976, Endrődi 1963, 1966, 1985a, Krajcik 2005, 2012, Ratcliffe and Cave 2015

#### Remarks.

*Cyclocephala striata* was reported from Brazil (Santa Catarina) (Endrődi 1963, 1966, 1985a). *Cyclocephala striata* has not been reported from Brazil since its original description and these data could be erroneous (Ratcliffe and Cave 2015).

### Cyclocephala subsignata

Taxon classificationAnimaliaColeopteraScarabaeidae

Burmeister, 1847

Cyclocephala subsignata Burmeister, 1847: 52 [original combination].

#### Types.

Lectotype ♀ at MNHN (Dechambre 1991b). Invalid neotype ♂ at HNHM (Endrődi Collection) (Endrődi 1966).

#### Distribution.

BRAZIL: Pará. FRENCH GUIANA: Apatou, Cayenne, St.-Élie, St.-Laurent du Maroni. GUYANA: Upper Demerara-Berbice. SURINAME.

#### References.

Burmeister 1847, Harold 1869b, Bodkin 1919, Arrow 1937b, Blackwelder 1944, Pike et al. 1976, Endrődi 1966, 1985a, Dechambre 1979a, 1991b, Ponchel 2011, Krajcik 2005, 2012.

### Cyclocephala supernana

Taxon classificationAnimaliaColeopteraScarabaeidae

Dechambre, 1999

Cyclocephala supernana Dechambre, 1999: 17–18 [original combination].

#### Types.

Holotype ♂ at MNHN (Dechambre 1999).

#### Distribution.

VENEZUELA: Amazonas.

#### References.

Dechambre 1999, Krajcik 2005, 2012.

### Cyclocephala suturalis

Taxon classificationAnimaliaColeopteraScarabaeidae

Ohaus, 1911

Cyclocephala suturalis Ohaus, 1911: 560–561 [original combination].

#### Types.

Type at SDEI (Endrődi 1966).

#### Distribution.

ARGENTINA: Misiones. BRAZIL: Minas Gerais, Paraná, Rio de Janeiro, Rio Grande do Sul, São Paulo. PARAGUAY. VENEZUELA: Carabobo.

#### References.

Ohaus 1911, Arrow 1937b, Blackwelder 1944, Guimarães 1944, Lima 1953, Martínez 1957, Pike et al. 1976, Dechambre 1979a, 1980, Endrődi 1966, 1985a, Grossi et al. 2011, Krajcik 2005, 2012, Breeschoten et al. 2013.

### Cyclocephala sylviae

Taxon classificationAnimaliaColeopteraScarabaeidae

Dechambre, 1995

Cyclocephala sylviae Dechambre, 1995: 13 [original combination].

#### Types.

Holotype ♂ at MNHN (Dechambre 1995).

#### Distribution.

BOLIVIA: La Paz, Santa Cruz.

#### References.

Dechambre 1995, Krajcik 2005, 2012.

### Cyclocephala tarsalis

Taxon classificationAnimaliaColeopteraScarabaeidae

Dechambre, 1979

Cyclocephala tarsalis Dechambre, 1979a: 166–167 [original combination].

#### Types.

Holotype ♂ at MNHN (Dechambre 1979a).

#### Distribution.

BRAZIL: Pará. FRENCH GUIANA.

#### References.

Dechambre 1979a, 1980, Endrődi 1985a, Ratcliffe 1992b, Ponchel 2011, Krajcik 2005, 2012.

### Cyclocephala testacea

Taxon classificationAnimaliaColeopteraScarabaeidae

Burmeister, 1847

Cyclocephala testacea Burmeister, 1847: 57–58 [original combination].

#### Types.

Endrődi (1966) could not find the type material of this species, but he did not designate a neotype.

#### Distribution.

ARGENTINA: Córdoba, La Rioja, Mendoza, Misiones, Salta, Santiago del Estero, Tucumán. BOLIVIA: Chuquisaca, Santa Cruz. BRAZIL: Acre, Amazonas, Ceará, Espírito Santo, Maranhão, Mato Grosso, Pará, Rio de Janeiro, Rio Grande do Sul, Roraima, Santa Catarina, São Paulo. COLOMBIA: Antioquia, Casanare, Cundinamarca. ECUADOR: Napo. FRENCH GUIANA: Cayenne, St.-Laurent du Maroni. GUYANA: Potaro-Siparuni, Upper Demerara-Berbice. PARAGUAY: Alto Paraná, Concepción, Cordillera, Gran Asunción. PERU: Loreto. SURINAME: Paramaribo. URUGUAY: Canelones, Montevideo, Treinta y Tres. VENEZUELA: Aragua, Zulia.

#### References.

Burmeister 1847, Harold 1869a, Arrow 1937b, Martorell 1939, Martorell and Salas 1939, Blackwelder 1944, Pike et al. 1976, Saenz and Morelli 1984, Endrődi 1966, 1985a, Morelli and Alzugaray 1994, Poole and Gentili 1996, Andreazze and Fonseca 1998, Riley and Wolfe 2003, Restrepo et al. 2003, Smith 2003, 2009, Ponchel 2011, Krajcik 2005, 2012, Breeschoten et al. 2013, López-García et al. 2015, Ratcliffe et al. 2015, Gasca-Álvarez and Deloya 2016.

#### Remarks.

Endrődi (1966, 1985a) considered *C. ovulum* Bates to be an “aberration” of *C. testacea* and reported these species' label data together for nearly all specimens. The distribution data for *C. testacea* in South America will need to be further evaluated.

### Cyclocephala tetrica

Taxon classificationAnimaliaColeopteraScarabaeidae

Burmeister, 1847

Cyclocephala tetrica Burmeister, 1847: 55 [original combination].

#### Types.

Invalid neotype ♂ at HNHM (Endrődi Collection) (Endrődi 1966).

#### Distribution.

JAMAICA: Clarendon, Hanover, Kingston, Manchester, Portland, St. Andrew, St. Catherine, St. Elizabeth, St. James, St. Mary, St. Thomas, Trelawny, Westmoreland.

#### References.

Burmeister 1847, Harold 1869b, Leng and Mutchler 1914, Arrow 1937b, Blackwelder 1944, Howden 1970, Pike et al. 1976, Endrődi 1966, 1969b, 1985a, Hagstrum and Subramanyam 2009, Krajcik 2005, 2012, Ratcliffe and Cave 2015.

### Cyclocephala tidula

Taxon classificationAnimaliaColeopteraScarabaeidae

Dechambre, 1999

Cyclocephala tidula Dechambre, 1999: 18–19 [original combination].

#### Types.

Holotype ♂ at MNHN (Dechambre 1999).

#### Distribution.

VENEZUELA: Bolívar.

#### References.

Dechambre 1999, Krajcik 2005, 2012.

### Cyclocephala toulgoeti

Taxon classificationAnimaliaColeopteraScarabaeidae

Dechambre, 1992

Cyclocephala toulgoeti Dechambre, 1992: 60 [original combination].

#### Types.

Holotype ♂ at MNHN (Dechambre 1992).

#### Distribution.

FRENCH GUIANA: Cayenne, Mana, Saül, St.-Élie, St.-Laurent du Maroni

#### References.

Dechambre 1992, Ponchel 2011, Krajcik 2005, 2012.

### Cyclocephala tridentata

Taxon classificationAnimaliaColeopteraScarabaeidae

(Fabricius, 1801)

Melolontha tridentata Fabricius, 1801: 170–171 [original combination].
Cyclocephala tridentata (Fabricius) [new combination by Burmeister 1847: 47].
**syn.** Cyclocephala annamariae Dutrillaux & Chalumeau, 2013: 64–65 [original combination in Dutrillaux et al. 2013].
Cyclocephala tridentata Fabricius [synonymy by Ratcliffe and Cave 2015: 113].
**syn.** Cyclocephala tridentata dominicensis Cartwright & Chalumeau, 1978: 25 [original combination].
Cyclocephala dominicensis Cartwright & Chalumeau [new species status by Dutrillaux et al. 2013: 64].
Cyclocephala tridentata (Fabricius) [synonymy by Ratcliffe and Cave 2015: 113].
**syn.** Melolontha biliturata Gyllenhal, 1817b: 105–106 [original combination].
Cyclocephala tridentata (Fabricius) [synonymy by Burmeister 1847: 48].

#### Types.

Lectotype ♀ of *M. tridentata* deposited at ZMUK, now housed at ZMUC (Endrődi 1966). Chalumeau (1982) did not consider the lectotype valid because Endrődi selected a specimen from Martinique rather than Guadeloupe, the type locality. Therefore, Chalumeau designated a neotype deposited at ZMUC.Holotype ♂ of *C. annamariae* at IREC (Dutrillaux et al. 2013). Holotype ♂ of *C. tridentata dominicensis* in the Fortuné Chalumeau Collection (Cartwright and Chalumeau 1978). The type material of *M. biliturata* was not reported by Endrődi (1966), and it is not with the other Gyllenhal *Melolontha* types at UUZM (Wallin 2001).

#### Distribution.

BARBADOS. COLOMBIA: Cauca. DOMINICA: St. David, St. George, St. John, St. Joseph, St. Luke, St. Mark, St. Patrick, St. Paul. GRENADA: St. Andrew. GUADELOUPE: Basse-Terre, Grande-Terre. MARTINIQUE: Fort-de-France, La Trinité, Le Marin, Saint-Pierre. MONTSERRAT: Saint Georges. ST. LUCIA: Anse la Raye, Castries, Choiseul, Dauphin, Dennery, Micoud, Praslin, Soufrière, Vieux Fort. SURINAME.

#### References.

Voet 1769, Fabricius 1801, Gyllenhal 1817b, Dejean 1821, 1833, Burmeister 1847, Harold 1869b, Leng and Mutchler 1914, 1917, Dash 1922, Arrow 1937b, Blackwelder 1944, Paulian 1947, Zimsen 1964, Pike et al. 1976, Chalumeau and Gruner 1977, Cartwright and Chalumeau 1978, Chalumeau 1982, Endrődi 1966, 1985a, Dechambre 1980, 1992, Marquet and Roguet 2003, Restrepo et al. 2003, Krajcik 2005, 2012, Giannoulis et al. 2012, Breeschoten et al. 2013, Dutrillaux et al. 2007, 2013, 2014, Ratcliffe and Cave 2015, Gasca-Álvarez and Deloya 2016, Peck 2009, 2016.

#### Remarks.

The identification of the specimens from Colombia and Suriname need to be critically evaluated (Endrődi 1966, 1985a, Ratcliffe and Cave 2015). Chalumeau (1982) reported four specimens of *C. tridentata* from Puerto Rico, though major faunistic studies did not find additional specimens from Puerto Rico (Ratcliffe and Cave 2015).

### Cyclocephala tronchonii

Taxon classificationAnimaliaColeopteraScarabaeidae

Martínez, 1975

Cyclocephala tronchonii Martínez, 1975b: 264–270 [original combination].

#### Types.

Holotype ♂ at MACN (Antonio Martínez Collection) (Martínez 1975b).

#### Distribution.

PERU: Huánuco.

#### References.

Martínez 1975b, Pike et al. 1976, Endrődi 1985a, Krajcik 2005, 2012, Ratcliffe et al. 2015.

### Cyclocephala tucumana

Taxon classificationAnimaliaColeopteraScarabaeidae

Bréthes, 1905

Cyclocephala tucumana Bréthes, 1905: 330–331 [original combination].

#### Types.

Endrődi (1966) did not examine the type material of this species and speculated that the material would be found in Buenos Aires.

#### Distribution.

ARGENTINA: Jujuy, Salta, Santa Fe, Tucumán. BOLIVIA: Cochabamba. BRAZIL: Mato Grosso do Sul, Rio Grande do Sul, Paraná. PARAGUAY. URUGUAY: Montevideo.

#### References.

Bréthes 1905, Seidlitz 1905, Schrottky 1913, Arrow 1937b, Blackwelder 1944, Pike et al. 1976, Endrődi 1966, 1985a, Krajcik 2005, 2012, Breeschoten et al. 2013, Lima Nogueira et al. 2013, Cherman et al. 2013.

### Cyclocephala tutilina

Taxon classificationAnimaliaColeopteraScarabaeidae

Burmeister, 1847

Cyclocephala tutilina Burmeister, 1847: 68 [original combination].
**syn.** Cyclocephala venezuelae Arrow, 1911: 171–172 [original combination].
Cyclocephala tutilina Burmeister [synonymy by Endrődi 1966: 323].

#### Types.

Lectotype ♂ of *C. tutilina* at MLUH (Endrődi 1966). Type of *C. venezuelae* at BMNH (Endrődi 1966).

#### Distribution.

BRAZIL. COLOMBIA: Antioquia, Cauca, Chocó, Santander, Tolima. ECUADOR. VENEZUELA: Aragua, Capital District, Carabobo, Mérida.

#### References.

Burmeister 1847, Reiche 1859, Harold 1869b, Ohaus 1910, Arrow 1911, 1937b, Anonymous 1940, Blackwelder 1944, Martínez 1967, Pike et al. 1976, Endrődi 1966, 1967b, 1985a, Seres and Ramírez 1995, Neita-Moreno 2011, Pardo-Locarno et al. 2005a, Ratcliffe and Cave 2006, Krajcik 2005, 2012, Breeschoten et al. 2013, Moore and Jameson 2013, Ratcliffe et al. 2013, Yepes-Rodriguez et al. 2013, López-García et al. 2015, Gasca-Álvarez and Deloya 2016.

#### Remarks.

Endrődi (1966, 1985a) reported *C. tutilina* from Mexico and Honduras. The validity of these Central American records was questioned by Ratcliffe (2003), and other recent data for Honduras and Nicaragua were based on erroneous identifications (Ratcliffe and Cave 2006, Ratcliffe et al. 2013). *Cyclocephala tutilina* is likely restricted to South America.

### Cyclocephala tylifera

Taxon classificationAnimaliaColeopteraScarabaeidae

Höhne, 1923

Cyclocephala tylifera Höhne, 1923b: 370–371 [original combination].

#### Types.

Lectotype ♂ at ZMHB (Endrődi 1966).

#### Distribution.

BOLIVIA: Cochabamba, La Paz. BRAZIL: Amazonas, Mato Grosso. COLOMBIA: Cauca, Boyacá. FRENCH GUIANA: Kourou, St.-Laurent du Maroni. GUYANA. PERU: Callao, Huánuco, Junín, Madre de Dios. SURINAME: Marowijne.

#### References.

Höhne 1923b, Arrow 1937b, Blackwelder 1944, Gruner 1971, Pike et al. 1976, Endrődi 1966, 1985a, Dechambre 1995, Silberbauer-Gottsberger 2001, Gibernau and Barabé 2002, Restrepo et al. 2003, Krajcik 2005, 2012, Ponchel 2006, 2011, 2015, Ratcliffe et al. 2015, Gasca-Álvarez 2013, Gasca-Álvarez and Deloya 2016.

### Cyclocephala unamas

Taxon classificationAnimaliaColeopteraScarabaeidae

Ratcliffe, 2003

Cyclocephala unamas Ratcliffe, 2003: 61, 67, 71, 78, 212–214 [original combination].

#### Types.

Holotype ♂ at MNCR (originally deposited at INBio) (Ratcliffe 2003).

#### Distribution.

COSTA RICA: Cartago, San José.

#### References.

Ratcliffe 2003, Krajcik 2005, 2012.

### Cyclocephala undata

Taxon classificationAnimaliaColeopteraScarabaeidae

(Olivier, 1789)

Melolontha undata Olivier, 1789: 80 [original combination].
Cyclocephala undata (Olivier) [new combination by Burmeister 1847: 61].
**syn.** Cyclocephala rubicunda Burmeister, 1847: 61–62 [original combination].

Cyclocephala undata (Olivier) [synonymy by Endrődi 1966: 325].
**syn.** Melolontha spilophthalma Herbst, 1790: 163–164 [original combination].
Cyclocephala undata (Olivier) [synonymy by Burmeister 1847: 61].

#### Types.

Invalid ♂ neotype of *M. undata* at HNHM (Endrődi Collection) (Endrődi 1966). Lectotype ♂ of *C. rubicunda* at MNHN (Dechambre 1991b). Endrődi (1966) did not comment on the *M. spilophthalma* type material.

#### Distribution.

BRAZIL: Amazonas, Mato Grosso. FRENCH GUIANA: Cayenne, St.-Laurent du Maroni. GUYANA. SURINAME: Paramaribo.

#### References.

Olivier 1789, Herbst 1790, Burmeister 1847, Harold 1869b, Arrow 1937b, Pike et al. 1976, Endrődi 1966, 1967b, 1969b, 1985a, Dechambre 1991b, Ratcliffe 1992b, Webber and Gottsberger 1993, Küchmeister et al. 1998, Gottsberger et al. 1998, Gottsberger 1999, Silberbauer-Gottsberger 2001, Ponchel 2006, 2011, Krajcik 2005, 2012, Moore and Jameson 2013, Costa et al. 2017.

### Cyclocephala unidentata

Taxon classificationAnimaliaColeopteraScarabaeidae

Endrődi, 1980

Cyclocephala unidentata Endrődi, 1980: 37–38 [original combination].

#### Types.

Holotype ♀ in André Gaudaíre-Thore Collection (Sens, France) (Endrődi 1980).

#### Distribution.

FRENCH GUIANA. VENEZUELA.

#### References.

Endrődi 1980, 1985a, Ponchel 2011, Krajcik 2005, 2012.

### Cyclocephala variabilis

Taxon classificationAnimaliaColeopteraScarabaeidae

Burmeister, 1847

Cyclocephala variabilis Burmeister, 1847: 44–45 [original combination].
Cyclocephala (Cyclocephala) variabilis Burmeister [new subgeneric classification by Casey 1915: 138].
Cyclocephala variabilis Burmeister [removal of subgeneric classification by Arrow 1937b: 8, 16].

#### Types.

Lectotype ♂ at MLUH (Endrődi 1966).

#### Distribution.

ARGENTINA: Buenos Aires. BOLIVIA: Cochabamba, Santa Cruz. BRAZIL: Amazonas, Bahia, Goiás, Minas Gerais, Paraná, Rio de Janeiro, Rio Grande do Sul, Santa Catarina, São Paulo. COLOMBIA: Cauca, Boyacá. ECUADOR: Morona-Santiago. FRENCH GUIANA: Cayenne. PANAMA: Darien, Former Canal Zone. URUGUAY: Rocha. VENEZUELA: Capital District.

#### References.

Burmeister 1847, Harold 1869b, Casey 1915, Luederwaldt 1915, Monte 1933, Arrow 1937b, Blackwelder 1944, Pike et al. 1976, Saenz and Morelli 1984, Endrődi 1963, 1966, 1985a, Gottsberger 1986, Andreazze and Fonseca 1994, Ratcliffe and Morón 1997, Ratcliffe 2002a, 2003, Restrepo et al. 2003, Smith 2003, 2009, Grossi et al. 2011, Ponchel 2011, Krajcik 2005, 2012, Breeschoten et al. 2013, Ratcliffe et al. 2013, López-García et al. 2015, Gasca-Álvarez and Deloya 2016.

#### Remarks.

*Cyclocephala variabilis* was reported from Mexico (Endrődi 1966, Ratcliffe and Morón 1997). Major faunistic studies did not record any further specimens, and this species likely does not occur in Mexico (Ratcliffe et al. 2013).

### Cyclocephala varians

Taxon classificationAnimaliaColeopteraScarabaeidae

Burmeister, 1847

Cyclocephala varians Burmeister, 1847: 64 [original combination].

#### Types.

Lectotype ♂ at MLUH (Endrődi 1966).

#### Distribution.

COLOMBIA: Cundinamarca, Meta, Tolima. FRENCH GUIANA: Kourou, Sinnamary.

#### References.

Burmeister 1847, Harold 1869b, Arrow 1937b, Blackwelder 1944, Pike et al. 1976, Endrődi 1966, 1985a, Gibernau et al. 2003, Krajcik 2005, 2012, Ponchel 2006, 2011, 2015, Gasca-Álvarez and Deloya 2016.

### Cyclocephala variipenis

Taxon classificationAnimaliaColeopteraScarabaeidae

Dechambre, 1999

Cyclocephala variipenis Dechambre, 1999: 19–20 [original combination].

#### Types.

Holotype ♂ at MNHN (Dechambre 1999).

#### Distribution.

BOLIVIA: La Paz.

#### References.

Dechambre 1999, Krajcik 2005, 2012.

### Cyclocephala variolosa

Taxon classificationAnimaliaColeopteraScarabaeidae

Burmeister, 1847

Cyclocephala variolosa Burmeister, 1847: 70 [original combination].
**syn.** Surutoides mirabilis Endrődi, 1981: 198–199 [original combination].
Cyclocephala variolosa Burmeister [synonymy by Dechambre 1991a: 282].

#### Types.

Lectotype ♂ of *C. variolosa* at MLUH (Endrődi 1966). Holotype ♂ of *S. mirabilis* at ZMHB (Endrődi 1981).

#### Distribution.

BRAZIL: Espírito Santo, Paraná, Pernambuco, Rio de Janeiro, Santa Catarina, São Paulo.

#### References.

Burmeister 1847, Harold 1869b, Arrow 1937b, Blackwelder 1944, Pike et al. 1976, Endrődi 1966, 1981, 1985a, Gottsberger and Amaral 1984, Dechambre 1991a, 1997, Weber et al. 2001, Riehs 2005, 2006, Weber 2008, Krajcik 2005, 2012, Gottsberger 1986, 2016.

### Cyclocephala verticalis

Taxon classificationAnimaliaColeopteraScarabaeidae

Burmeister, 1847

Cyclocephala verticalis Burmeister, 1847: 51 [original combination].
Cyclocephala sanguinicollis var. verticalis Burmeister [new infrasubspecific status by Arrow 1937b: 15].
Cyclocephala verticalis Burmeister [revalidated species status by Endrődi 1966: 329].

#### Types.

Types at MLUH (Endrődi 1966).

#### Distribution.

ARGENTINA. BOLIVIA: Cochabamba. BRAZIL: Acre, Amazonas, Mato Grosso, Mato Grosso do Sul, Pará, Paraná, Rio Grande do Norte, Roraima, Santa Catarina. COLOMBIA: Amazonas. ECUADOR. FRENCH GUIANA: Cayenne. GUYANA: Upper Takutu-Upper Essequibo. PERU: Huánuco, Loreto. SURINAME: Paramaribo. VENEZUELA: Barinas.

#### References.

Burmeister 1847, Reiche 1859, Chevrolat 1865, Harold 1869b, Gundlach 1891, Arrow 1937b, Blackwelder 1944, Cramer et al. 1975, Prance and Arias 1975, Pike et al. 1976, Prance 1976, Prance and Anderson 1976, Endrődi 1966, 1980, 1985a, Wiersema 1987, Dechambre 1980, 1999, Fernández García 2006, Seymour and Matthews 2006, Puker et al. 2009, Rodrigues et al. 2010, 2011, Coutinho et al. 2011, Krajcik 2005, 2012, Breeschoten et al. 2013, Otavo et al. 2013, Maia et al. 2014, Ratcliffe et al. 2015, Gasca-Álvarez and Deloya 2016.

#### Remarks.

The original description of *C. verticalis* was based on at least one specimen labeled from Cuba (Burmeister 1847). This Cuban locality has been reported by a few authors (Burmeister 1847, Chevrolat 1865, Blackwelder 1944), but *C. verticalis* does not occur in Cuba or the West Indies more broadly (Ratcliffe and Cave 2015).

### Cyclocephala vestita

Taxon classificationAnimaliaColeopteraScarabaeidae

Höhne, 1923

Cyclocephala vestita Höhne, 1923b: 359 [original combination].

#### Types.

Types at ZMHB (Endrődi 1966).

#### Distribution.

BRAZIL: Acre, Amazonas, Bahia, Pernambuco, Rio de Janeiro. FRENCH GUIANA: Cayenne, Kourou, Sinnamary. GUYANA. PARAGUAY. SURINAME: Paramaribo.

#### References.

Höhne 1923b, Arrow 1937b, Blackwelder 1944, Pike et al. 1976, Endrődi 1966, 1985a, Cavalcante 2000, Joly 2000a, Gibernau et al. 2003, Marques and Gil-Santana 2009, Krajcik 2005, 2012, Maia et al. 2010, 2012, Breeschoten et al. 2013, Maia et al. 2014, Ponchel 2006, 2011, 2015, Albuquerque et al. 2016, Gottsberger 2016.

### Cyclocephala vidanoi

Taxon classificationAnimaliaColeopteraScarabaeidae

Dechambre, 1992

Cyclocephala vidanoi Dechambre, 1992: 66–67 [original combination].

#### Types.

Holotype ♂ at MNHN (Dechambre 1992).

#### Distribution.

COLOMBIA: Antioquia, Chocó. ECUADOR: Pichincha.

#### References.

Dechambre 1992, Neita-Moreno 2011, Krajcik 2005, 2012, Gasca-Álvarez and Deloya 2016.

### Cyclocephala villosa

Taxon classificationAnimaliaColeopteraScarabaeidae

Blanchard, 1846

Cyclocephala villosa Blanchard, 1846: 192 [original combination].

#### Types.

Invalid ♂ neotype at MNHN (Endrődi Collection) (Endrődi 1966).

#### Distribution.

BOLIVIA: Santa Cruz. BRAZIL: Minas Gerais.

#### References.

Blanchard 1846, Harold 1869b, Arrow 1937b, Blackwelder 1944, Pike et al. 1976, Endrődi 1966, 1985a, Krajcik 2005, 2012.

### Cyclocephala vincentiae

Taxon classificationAnimaliaColeopteraScarabaeidae

Arrow, 1900

Cyclocephala vincentiae Arrow, 1900: 180–181 [original combination].

#### Types.

Types at BMNH (Endrődi 1966).

#### Distribution.

ST. VINCENT.

#### References.

Arrow 1900, 1937b, Leng and Mutchler 1914, Blackwelder 1944, Pike et al. 1976, Endrődi 1966, 1985a, Joly 2003, Krajcik 2005, 2012, Ratcliffe and Cave 2015, Peck 2010, 2016.

### Cyclocephala vinosa

Taxon classificationAnimaliaColeopteraScarabaeidae

Arrow, 1937

Cyclocephala vinosa Arrow, 1937a: 39–40 [original combination].

#### Types.

Types at BMNH (Endrődi 1966).

#### Distribution.

JAMAICA: Kingston, Manchester, Portland, St. Andrew, St. Thomas.

#### References.

Arrow 1937a, b, Blackwelder 1944, Pike et al. 1976, Endrődi 1966, 1985a, Joly 1998, Krajcik 2005, 2012, Ratcliffe and Cave 2015.

### Cyclocephala virgo

Taxon classificationAnimaliaColeopteraScarabaeidae

Dechambre, 1999

Cyclocephala virgo Dechambre, 1999: 20–21 [original combination].

#### Types.

Holotype ♀ at MNHN (Dechambre 1999).

#### Distribution.

BRAZIL: Pará. FRENCH GUIANA: Régina, Saül, St.-Laurent du Maroni.

#### References.

Dechambre 1999, Ponchel 2010, 2011, Krajcik 2005, 2012.

### Cyclocephala viridis

Taxon classificationAnimaliaColeopteraScarabaeidae

Dechambre, 1982

Cyclocephala viridis Dechambre, 1982: 2–3 [original combination].

#### Types.

Holotype ♂ at MNHN (Dechambre 1982).

#### Distribution.

BRAZIL: Amazonas. PERU. VENEZUELA.

#### References.

Dechambre 1982, Krajcik 2005, 2012, Ratcliffe et al. 2015.

### Cyclocephala vittoscutellaris

Taxon classificationAnimaliaColeopteraScarabaeidae

Prell, 1937

Cyclocephala vittoscutellaris Prell, 1937b: 496 [original combination].

#### Types.

Holotype ♀ at ZMHB (Endrődi 1967b).

#### Distribution.

BRAZIL: Mato Grosso. COLOMBIA: Boyacá,

#### References.

Prell 1937b, Blackwelder 1944, Pike et al. 1976, Endrődi 1966, 1967b, 1985a, Restrepo et al. 2003, Krajcik 2005, 2012, López-García et al. 2015, Gasca-Álvarez and Deloya 2016.

### Cyclocephala wandae

Taxon classificationAnimaliaColeopteraScarabaeidae

Hardy, 1974

Cyclocephala wandae Hardy, 1974: 160–161 [original combination].

#### Types.

Holotype ♂ at USNM (Hardy 1974).

#### Distribution.

UNITED STATES: California.

#### References.

Pike et al. 1976, Hardy 1974, 1991, Endrődi 1985a, Poole and Gentili 1996, Joly 2000a, Smith 2003, 2009, Krajcik 2005, 2012, Ratcliffe and Cave 2017.

### Cyclocephala weidneri

Taxon classificationAnimaliaColeopteraScarabaeidae

Endrődi, 1964

Cyclocephala weidneri Endrődi, 1964: 462–464 [original combination].

#### Types.

Holotype ♂ at HNHM (Endrődi Collection) (Endrődi 1964).

#### Distribution.

BRAZIL: Espírito Santo. COLOMBIA: Antioquia, Cauca, Chocó, Cundinamarca. COSTA RICA: Alajuela, Cartago, Guanacaste, Heredia, Limón, Puntarenas, San José. ECUADOR: Morona-Santiago, Pastaza. EL SALVADOR: Ahuachapán. GUATEMALA: Alta Verapaz, Baja Verapaz, Chimaltenango, El Progreso, Escuintla, Guatemala, Huehuetenango, Izabal, Jalapa, Jutiapa, Quetzaltenango, San Marcos, Sololá, Suchitepéquez, Zacapa. HONDURAS: Copán, Cortés, Lempira, Olancho, Yoro. MEXICO: Chiapas, Hidalgo, Oaxaca, Puebla, Veracruz. PANAMA: Bocas del Toro, Chiriquí, Former Canal Zone. PERU: Cusco, Junín. VENEZUELA: Aragua, Mérida.

#### References.

Pike et al. 1976, Dechambre 1979a, Endrődi 1964, 1966, 1985a, Ratcliffe and Morón 1997, Morón-Ríos and Morón 2001, Ratcliffe 2002a, 2003, Restrepo et al. 2003, Pardo-Locarno et al. 2005a, Ratcliffe and Cave 2006, Neita-Moreno and Gaigl 2008, Múñoz-Hernández et al. 2008, Krajcik 2005, 2012, Moore 2012, Breeschoten et al. 2013, García-López et al. 2013, Ratcliffe et al. 2013, Yepes-Rodriguez et al. 2013, López-García et al. 2015, Gasca-Álvarez and Deloya 2016, Dupuis 2018.

### Cyclocephala williami

Taxon classificationAnimaliaColeopteraScarabaeidae

Ratcliffe, 1992

Cyclocephala williami Ratcliffe, 1992a: 230–232 [original combination].

#### Types.

Holotype ♂ at UNSM (Ratcliffe 1992a).

#### Distribution.

COSTA RICA: Alajuela, Guanacaste, Limón, Puntarenas.

#### References.

Ratcliffe 1992a, 2003, Krajcik 2005, 2012, García-López et al. 2013.

### Cyclocephala zischkai

Taxon classificationAnimaliaColeopteraScarabaeidae

Martínez, 1965

Cyclocephala zischkai Martínez, 1965a: 70–74 [original combination].

#### Types.

Holotype ♂ at MACN (Antonio Martínez Collection) (Martínez 1965a).

#### Distribution.

BOLIVIA: Cochabamba.

#### References.

Martínez 1965a, 1975b, Pike et al. 1976, Endrődi 1985a, Krajcik 2005, 2012.

### Cyclocephala zodion

Taxon classificationAnimaliaColeopteraScarabaeidae

Ratcliffe, 1992

Cyclocephala zodion Ratcliffe, 1992a: 232–234 [original combination].

#### Types.

Holotype ♂ at UNSM (Ratcliffe 1992a).

#### Distribution.

COSTA RICA: Heredia, Limón. PANAMA: Bocas del Toro, Coclé, Panamá.

#### References.

Ratcliffe 1992a, 2002a, 2003, Krajcik 2005, 2012, García-López et al. 2013.

### Cyclocephala zurstrasseni

Taxon classificationAnimaliaColeopteraScarabaeidae

Endrődi, 1964

Cyclocephala zurstrasseni Endrődi, 1964: 459–461 [original combination].

#### Types.

Holotype ♂ at NHMB (Frey Collection) (Endrődi 1964).

#### Distribution.

PERU: Cusco, Huánuco, Madre de Dios.

#### References.

Pike et al. 1976, Endrődi 1964, 1966, 1985a, Krajcik 2005, 2012, Ratcliffe et al. 2015.

### Genus Dyscinetus

Taxon classificationAnimaliaColeopteraScarabaeidae

Harold, 1869

Dyscinetus Harold, 1869a: 123 [original usage, new replacement name for preoccupied genus name Chalepus MacLeay, 1819: 149].
**syn.** Palechus Casey, 1915: 174 [original usage]. Proposed as a subgenus. Type species: Palechus histrio Casey, by original designation.
Dyscinetus Harold [synonymy by Saylor 1945: 380].

#### Type species.

*Melolontha geminata* Fabricius, 1801, by monotypy.

#### Keys.

Dawson 1922 (Nebraska, USA), Chapin 1932 (Cuba), Saylor 1945 (USA), Paulian 1947 (French Antilles), Arnett 1968 (USA), Endrődi 1966, 1985a, Morón 1979 (Veracruz, Mexico), Gordon and Anderson 1981 (larvae and adults, Florida, United States), Ratcliffe 1985, Maes 1994 (Nicaragua), Morón and Zaragoza 1976 (Estado de México, Mexico), Morón et al. 1985 (Chiapas, Mexico), Morón et al. 1988 (Jalisco, Mexico), Ratcliffe 1991 (Nebraska, USA), Jameson et al. 2002, Ratcliffe 2003 (Costa Rica and Panama), Villegas-Urbano 2004 (larvae, Colombia), Ratcliffe and Cave 2006 (Honduras, Nicaragua, and El Salvador), Ratcliffe and Paulsen 2008 (Nebraska, USA), Gasca-Álvarez and Amat-García 2010 (Colombia), Joly and Escalona 2010 (Venezuela), Neita-Moreno and Yepes 2011 (larvae), Ratcliffe et al. 2013 (Guatemala, Belize, and Mexico), Pardo-Locarno 2013 (Valle del Cauca, Colombia), Cherman et al. 2013 (larvae, Rio Grande do Sul, Brazil), Ratcliffe and Cave 2015 (West Indies), Ratcliffe and Cave 2017 (USA and Canada).

#### Valid taxa.

21 species.

### Dyscinetus australis

Taxon classificationAnimaliaColeopteraScarabaeidae

Joly & Escalona, 2002

Dyscinetus australis Joly & Escalona, 2002b: 199, 201, 202–205 [original combination].

#### Types.

Holotype ♂ at CMNC (Henry and Anne Howden Collection) (Joly and Escalona 2002b).

#### Distribution.

ARGENTINA: Buenos Aires, Neuquén, Río Negro.

#### References.

Joly and Escalona 2002b, Krajcik 2005, 2012.

### Dyscinetus dubius

Taxon classificationAnimaliaColeopteraScarabaeidae

(Olivier, 1789)

Melolontha dubia Olivier, 1789: 32 [original combination].
Dyscinetus dubius (Olivier) [new combination by Bates 1891: 32].
Dyscinetus (Palechus) dubius (Olivier) [new subgeneric classification by Casey 1915: 174].
Dyscinetus dubius (Olivier) [removal of subgeneric classification by Arrow 1937b: 17].
**syn.** Geotrupes lugubris Quensel, 1806: 21 [original combination].
Melolontha geminata Fabricius [synonymy by Schönherr 1817: 187].
**syn.** Dyscinetus frater Bates, 1888: 312 [original combination].
Dyscinetus dubius (Olivier) [synonymy by Endrődi 1966: 387].
**syn.** Dyscinetus (Dyscinetus) obtusus Casey, 1915: 170 [original combination].
Dyscinetus frater Bates [synonymy by Chapin 1932: 295].
**syn.** Melolontha geminata Fabricius, 1801: 166–167 [original combination].
Chalepus geminatus (Fabricius) [new combination by MacLeay 1819: 149–150].
Cyclocephala geminata (Fabricius) [new combination by Laporte 1840: 124].
Chalepus geminatus (Fabricius) [revised combination by Burmeister 1847: 78–79].
Dyscinetus geminatus (Fabricius) [new combination by Harold 1869a: 123].
Dyscinetus dubius (Olivier) [synonymy by Prell 1937a: 187].

#### Types.

Type material of *M. dubia* is apparently missing (Endrődi 1966). Lectotype ♂ of *M. geminata* deposited at ZMUK, now housed at ZMUC (Endrődi 1966). Endrődi (1966) did not find the type material of *G. lugubris*. Lectotype ♂ of *D. frater* at BMNH (Endrődi 1966). Type of *D. obtusus* at USNM (Endrődi 1966).

#### Distribution.

ARGENTINA: Buenos Aires, Entre Ríos, Misiones. BELIZE: Cayo. BOLIVIA: Beni, Chuquisaca, Cochabamba, La Paz, Santa Cruz. BRAZIL: Acre, Amazonas, Bahia, Espírito Santo, Goiás, Mato Grosso, Pará, Paraná, Pernambuco, Rio de Janeiro, Rio Grande do Norte, Rio Grande do Sul, Rondônia, Santa Catarina, São Paulo. CHILE. COLOMBIA: Amazonas, Antioquia, Atlántico, Boyacá, Caquetá, Cauca, Casanare, Chocó, Córdoba, Cundinamarca, Huila, Meta, Risaralda, Santander, Tolima, Valle del Cauca. COSTA RICA: Alajuela, Cartago, Guanacaste, Heredia, Limón, Puntarenas, San José. CUBA: Camagüey, Ciego de Ávila, Isla de la Juventud, Matanzas, Pinar del Río, Sancti Spíritus. ECUADOR: Guayas. EL SALVADOR: Ahuachapán, Cabañas, Cuscatlán, La Libertad, San Miguel, San Salvador, Santa Ana. FRENCH GUIANA: Cayenne, St.-Laurent du Maroni. GUATEMALA: Alta Verapaz, Baja Verapaz, Chiquimula, Escuintla, Guatemala, Huehuetenango, Izabal, Petén, Quetzaltenango, Retalhuleu, San Marcos, Santa Rosa, Suchitepéquez, Zacapa. GUYANA: Demerara-Mahaica, Mahaica-Berbice. HONDURAS: Atlántida, Choluteca, Mayagua, Cortés, El Paraíso, Francisco Morazán, Gracias a Dios, La Paz, Olancho. MEXICO: Campeche, Chiapas, Distrito Federal, Guerrero, Jalisco, Nayarit, Oaxaca, Quintana Roo, San Luis Potosí, Sinaloa, Tabasco, Tamaulipas, Tlaxcala, Veracruz. NICARAGUA: Carazo, Granada, Managua, Masaya, RAA Sur, Río San Juan, Rivas. PANAMA: Bocas del Toro, Panama Canal Zone, Chiriquí, Coclé, Colón, Darien, Panamá, San Blas. PARAGUAY: Central, Cordillera, Guaíra, Paraguarí. PERU. SURINAME: Paramaribo District. TRINIDAD AND TOBAGO: Trinidad. VENEZUELA: Amazonas, Apure, Aragua, Bolívar, Carabobo, Delta Amacuro, Falcón, Guárico, Mérida, Monagas, Portuguesa, Táchira, Trujillo, Zulia.

#### References.

Olivier 1789, Fabricius 1801, Quensel 1806, Schönherr 1817, MacLeay 1819, Dejean 1821, 1833, 1836b, Latreille 1829, Laporte 1840, Sturm 1843, Burmeister 1847, Erichson 1848a, Reiche 1859, Fauvel 1861, Harold 1869a, Bates 1888, 1891, Ohaus 1900, Casey 1915, Cleare 1925, 1930, Chapin 1932, Squire 1932, 1933, Dash 1934, Prell 1937a, Arrow 1937b, Anonymous 1900, 1940, Blackwelder 1944, Guimarães 1944, Wiehe 1951, Figueroa-P. 1952, Lima 1953, Roze 1955, Gibson and Carrillo 1959, Van Dinther 1960, Zimsen 1964, Endrődi 1966, 1973a, 1985a, Dechambre 1979a, Morón 1979, Remillet 1988, Aguilera et al. 1993, Thomas 1993, Lobo and Morón 1993, Maes 1987, 1994, Ratcliffe and Morón 1997, Sanchez Soto 1998, Andreazze and Fonseca 1998, Ratcliffe 1986, 2002a, 2003, Restrepo-Giraldo et al. 2003, Pardo-Locarno et al. 1995, 2005a, Riehs 2005, Fernández García 2006, Ratcliffe and Cave 2006, Pacheco-F. et al. 2008, Marques and Gil-Santana 2009, Gasca-Álvarez and Amat-García 2010, Joly and Escalona 2010, Ponchel 2011, Neita-Moreno and Yepes 2011, Neita-Moreno 2011, Krajcik 2005, 2012, Breeschoten et al. 2013, Pardo-Locarno 2013, Otavo et al. 2013, García-López et al. 2013, Ratcliffe et al. 2013, Deloya et al. 2014a, Ratcliffe et al. 2013, 2015, Ratcliffe and Cave 2015, García-Atencia and Martínez-Hernández 2015, López-García et al. 2015, Albuquerque et al. 2016.

### Dyscinetus dytiscoides

Taxon classificationAnimaliaColeopteraScarabaeidae

Arrow, 1911

Dyscinetus dytiscoides Arrow, 1911: 168 [original combination].
Chalepides dytiscoides (Arrow) [new combination by Endrődi 1967a: 411].
Dyscinetus dytiscoides Arrow [revised combination by Joly and Escalona 2002a: 40].

#### Types.

Type at BMNH (Endrődi 1966).

#### Distribution.

BOLIVIA: Beni. COLOMBIA: Antioquia, Chocó. PERU: Loreto. VENEZUELA: Amazonas, Apure, Barinas, Delta Amacuro, Falcón, Monagas, Yaracuy, Zulia.

#### References.

Arrow 1911, 1937b, Roze 1955, Endrődi 1966, 1967a, 1985a, Lachaume 1992, Dechambre 1999, Restrepo-Giraldo et al. 2003, Gasca-Álvarez and Amat-García 2010, Joly and Escalona 2002a, 2010, Neita-Moreno 2011, Krajcik 2005, 2012, Ratcliffe et al. 2015.

### Dyscinetus fimosus

Taxon classificationAnimaliaColeopteraScarabaeidae

(Herbst, 1789)

Scarabaeus fimosus Herbst, 1789: 248–249 [original combination].
Geotrupes fimosus (Herbst) [new combination by Schönherr 1806: 22].
Chalepus fimosus (Herbst) [new combination by Burmeister 1847: 80].
Dyscinetus fimosus (Herbst) [new combination by Harold 1869a: 123].

#### Types.

Endrődi (1966) was unable to find type material of this species, and the species was completely unknown to him.

#### Distribution.

SURINAME. VENEZUELA: Aragua, Miranda.

#### References.

Herbst 1789, Schönherr 1806, Burmeister 1847, Harold 1869a, Roze 1955, Endrődi 1966, Krajcik 2005, 2012.

### Dyscinetus gagates

Taxon classificationAnimaliaColeopteraScarabaeidae

(Burmeister, 1847)

Chalepus gagates Burmeister, 1847: 81 [original combination].
Dyscinetus gagates (Burmeister) [new combination by Harold 1869a: 123].

#### Types.

Lectotype ♂ at MLUH (Endrődi 1966).

#### Distribution.

ARGENTINA: Buenos Aires. BOLIVIA: Beni. BRAZIL: Paraná, Rio Grande do Sul, São Paulo.

#### References.

Dejean 1833, 1836b, Burmeister 1847, Reiche 1859, Harold 1869a, Arrow 1937b, Bacigalupo 1939, López Cristóbal 1941, Bosq 1945, Mendheim 1953, Moore 1958, Endrődi 1966, 1985a, Balut 1970, Abrahão and Amante 1970, Mauco and Favero 2004, Riehs 2005, Dupuis 2006, Soave et al. 2006, Krajcik 2005, 2012, Breeschoten et al. 2013, Cherman et al. 2013, Salgado-Neto et al. 2016.

#### Remarks.

Ratcliffe et al. (2013) did not record *D. gagates* from Mexico, Guatemala, or Belize. The data from Mexico previously reported for *D. gagates* may be erroneous (Endrődi 1966, 1985a).

### Dyscinetus imitator

Taxon classificationAnimaliaColeopteraScarabaeidae

Ratcliffe, 1986

Dyscinetus imitator Ratcliffe, 1986: 75–78 [original combination].

#### Types.

Holotype ♂ at UCDC (Ratcliffe 1986).

#### Distribution.

CAYMAN ISLANDS: Cayman Brac, Grand Cayman. CUBA: Camagüey, La Habana, Isla de la Juventud, Mayabeque, Pinar del Río, Santiago de Cuba.

#### References.

Ratcliffe 1986, Askew 1994, Krajcik 2005, 2012, Ratcliffe and Cave 2010, 2015.

### Dyscinetus laevicollis

Taxon classificationAnimaliaColeopteraScarabaeidae

Arrow, 1937

Dyscinetus laevicollis Arrow, 1937a: 41 [original combination].

#### Types.

Type at BMNH (Endrődi 1966).

#### Distribution.

DOMINICAN REPUBLIC: Barahona, Distrito Nacional, Hato Mayor, La Altagracia, La Romana, La Vega, Monseñor Nouel, Monte Cristi, Monte Plata, Puerto Plata, Samana, Santo Domingo, Valverde. HAITI: Centre, Ouest. JAMAICA: St. Andrew, St. Elizabeth, Trelawny, Westmoreland. MEXICO: Aguascalientes, Chihuahua, Durango, Jalisco, Sonora, Tabasco. PUERTO RICO: Mayagüez. TURKS AND CAICOS ISLANDS: Middle Caicos, North Caicos. UNITED STATES: Arizona, New Mexico.

#### References.

Arrow 1937a, b, Blackwelder 1944, Howden 1970, Chalumeau 1982, Endrődi 1966, 1985a, Ratcliffe 1986, Ratcliffe and Morón 1997, Navarrete-Heredia et al. 2001, Smith 2003, 2009, Krajcik 2005, 2012, Ratcliffe et al. 2013, Ratcliffe and Cave 2015, Ratcliffe and Cave 2017.

### Dyscinetus laevipunctatus

Taxon classificationAnimaliaColeopteraScarabaeidae

Bates, 1888

Dyscinetus laevipunctatus Bates, 1888: 311–312 [original combination].
Dyscinetus (Palechus) laevipunctatus Bates [new subgeneric classification by Casey 1915: 174].
Dyscinetus laevipunctatus Bates [removal of subgeneric classification by Chapin 1932: 295].
**syn.** Dyscinetus (Palechus) histrio Casey, 1915: 174 [original combination].
Dyscinetus laevipunctatus Bates [synonymy by Chapin 1932: 295].

#### Types.

Type of *D. laevipunctatus* at BMNH (Endrődi 1966). Type of *D. histrio* at USNM (Endrődi 1966).

#### Distribution.

BELIZE: Cayo, Stann Creek, Toledo. BRAZIL. COLOMBIA: Caldas, Meta, Tolima, Valle del Cauca. COSTA RICA: Alajuela, Guanacaste, Heredia, Limón, Puntarenas. EL SALVADOR: Ahuachapán, La Libertad, San Salvador. GUATEMALA: Alta Verapaz, Baja Verapaz, Escuintla, Guatemala, Huehuetenango, Izabal, Jalapa, Petén, San Marcos, Santa Rosa, Suchitepéquez, Zacapa. HONDURAS: Atlántida, Choluteca, Colón, Comayagua, Copán, Cortés, Francisco Morazán, Gracias a Dios, Santa Bárbara, Yoro. MEXICO: Campeche, Chiapas, Coahuila, Colima, Distrito Federal, Guerrero, Jalisco, Michoacán, Nayarit, Nuevo León, Oaxaca, Puebla, Quintana Roo, San Luis Potosí, Sinaloa, Tabasco, Tamaulipas, Veracruz, Yucatán. NICARAGUA: Chontales, Granada, León, Managua, Masaya, RAA Sur, Río San Juan. PANAMA: Former Canal Zone.

#### References.

Bates 1888, Casey 1915, Chapin 1932, Arrow 1937b, Blackwelder 1944, Figueroa-P. 1952, Gibson and Carrillo 1959, Endrődi 1966, 1985a, Ratcliffe 1986, Maes 1987, 1994, Morón et al. 1985, 1988, Thomas 1993, Ratcliffe and Morón 1997, Sanchez Soto 1998, Navarrete-Heredia et al. 2001, Ratcliffe 2002a, 2003, Restrepo-Giraldo et al. 2003, Fernández García 2006, Ratcliffe and Cave 2006, Pacheco F. et al. 2008, Gasca-Álvarez and Amat-García 2010, Krajcik 2005, 2012, Moore 2012, Ratcliffe et al. 2013, Deloya et al. 2014a, 2016, García-Rivera and Contreras-Ramos 2015.

#### Remarks.

Casey (1915) reported *D. histrio* (= *D. laevipunctatus*) from the nonspecific locality “Amazon Valley”. This locality has been interpreted as being either erroneous or possibly Brazilian (Endrődi 1966, Ratcliffe 2003).

### Dyscinetus martinezi

Taxon classificationAnimaliaColeopteraScarabaeidae

Joly & Escalona, 2002

Dyscinetus martinezi Joly & Escalona, 2002b: 197–202 [original combination].

#### Types.

Holotype ♂ at CMNC (Henry and Anne Howden Collection) (Joly and Escalona 2002b).

#### Distribution.

ARGENTINA: Salta.

#### References.

Joly and Escalona 2002b, Krajcik 2005, 2012.

### Dyscinetus mendax

Taxon classificationAnimaliaColeopteraScarabaeidae

Joly & Escalona, 2010

Dyscinetus mendax Joly & Escalona, 2010: 207, 227–230 [original combination].

#### Types.

Holotype ♂ at MIZA (Joly and Escalona 2010).

#### Distribution.

BOLIVIA: Beni, Santa Cruz. BRAZIL: Acre, Amazonas, Mato Grosso, Pará, Rio de Janeiro, Rondônia. COLOMBIA: Antioquia. ECUADOR: Los Ríos. FRENCH GUIANA: Cayenne. MARTINIQUE: Le Marin. PERU: Loreto, Madre de Dios. SURINAME: Marowijne. TRINIDAD AND TOBAGO: Trinidad. VENEZUELA: Amazonas, Apure, Aragua, Bolívar, Carabobo, Cojedes, Delta Amacuro, Distrito Federal, Falcón, Guárico, Miranda, Monagas, Táchira.

#### References.

Joly and Escalona 2010, Krajcik 2012, Ratcliffe and Cave 2015.

### Dyscinetus minor

Taxon classificationAnimaliaColeopteraScarabaeidae

Chapin, 1935

Dyscinetus minor Chapin, 1935a: 74 [original combination].
Dyscinetus laevipunctatus minor Chapin [new subspecies status by Endrődi 1985a: 168].
Dyscinetus minor Chapin [revalidated species status by Ratcliffe and Cave 2015: 121, 129–130].

#### Types.

Type at USNM (Endrődi 1966).

#### Distribution.

CUBA: Artemisa, Cienfuegos, Isla de la Juventud, La Habana, Pinar del Río.

#### References.

Chapin 1935a, Bruner et al. 1975, Endrődi 1966, 1985a, Fernández García 2006, Krajcik 2005, 2012, Ratcliffe and Cave 2015.

### Dyscinetus morator

Taxon classificationAnimaliaColeopteraScarabaeidae

(Fabricius, 1798)

Scarabaeus morator Fabricius, 1798: 24 [original combination].
Geotrupes morator (Fabricius) [new combination by Schönherr 1806: 22].
Heteronychus morator (Fabricius) [new combination by Burmeister 1847: 97].
Dyscinetus morator (Fabricius) [new combination by Arrow 1937b: 17].
**syn.** Dyscinetus (Dyscinetus) bitumorosus Casey, 1915: 171 [original combination].
Dyscinetus bitumorosus Casey [removal of subgeneric classification by Arrow 1937b: 17].
Dyscinetus morator (Fabricius) [synonymy by Endrődi 1966: 393].
**syn.** Dyscinetus (Dyscinetus) borealis Casey, 1915: 171 [original combination].
Dyscinetus morator (Fabricius) [synonymy by Arrow 1937b: 18].
**syn.** Dyscinetus (Dyscinetus) trachypygus discedens Casey, 1915: 171 [original combination].
Dyscinetus morator (Fabricius) [synonymy by Arrow 1937b: 18].
**syn.** Chalepus trachypygus Burmeister, 1847: 79 [original combination].
Dyscinetus trachypygus (Burmeister) [new combination by Harold 1869a: 123].
Dyscinetus morator (Fabricius) [synonymy by Prell 1937a: 186].

#### Types.

Lectotype ♂ of *S. morator* deposited at ZMUK, now housed at ZMUC (Endrődi 1966). Lectotype ♂ of *C. trachypygus* at MLUH (Endrődi 1966). Types of the Casey synonyms at USNM (Endrődi 1966).

#### Distribution.

BAHAMAS: Andros, Eleuthera, Great Exuma, Rum Cay, San Salvador. CANADA: Ontario. MEXICO: Coahuila, San Luis Potosí, Tamaulipas, Veracruz. UNITED STATES: Alabama, Arkansas, Connecticut, Delaware, District of Columbia, Florida, Georgia, Illinois, Indiana, Iowa, Kansas, Kentucky, Louisiana, Maryland, Michigan, Minnesota, Missouri, Mississippi, Nebraska, New Jersey, New Mexico, New York, North Carolina, Ohio, Oklahoma, Pennsylvania, South Carolina, Tennessee, Texas, Virginia, West Virginia.

#### References.

Burmeister 1847, Melsheimer et al. 1853, Reiche 1859, Harold 1869b, Crotch 1873, Popenoe 1876, Schwarz 1878, Henshaw 1885, Smith 1910, King 1914, Casey 1915, Smyth 1915, 1916, Scammell 1917, Leng 1920, Dawson 1922, Leonard 1923, Phillips and Fox 1924, Blatchley 1930, Arrow 1937b, Sanderson 1940, Montgomery and Amos 1940, McAtee 1940, Blackwelder 1944, Saylor 1945, Blackwelder and Blackwelder 1948, Brower 1958, Richmond 1962, Zimsen 1964, Frost 1964, 1966, Moss and Funk 1965, Carrillo-S. et al. 1966, Fincher et al. 1969, Roth et al. 1972, Pfeiffer and Axtell 1980, Endrődi 1966, 1985a, Morrill 1979, Anonymous 1953, 1956, 1971, 1980, Gordon and Anderson 1981, Foster et al. 1986, Buckingham and Bennett 1989, Staines 1984, 1990, Price and Kring 1991, Forschler and Gardner 1991, Hardy 1991, Crocker et al. 1992, Poole and Gentili 1996, Peck and Thomas 1998, Flanders et al. 2000, Ratcliffe 1991, 2002b, Riley and Wolfe 2003, Shockley and Cline 2004, Buss 2006, Ratcliffe and Paulsen 2008, Smith 2003, 2009, Krajcik 2005, 2012, Ratcliffe et al. 2013, Woodruff 1970, 1973, 2013, Ratcliffe and Cave 2008, 2015, Chong and Hinson 2015, Ratcliffe, but Cave 2017.

#### Remarks.

*Dyscinetus morator* was recorded from Brazil (Riehs 2005) and these data may need to be reevaluated. Records of *D. morator* from Cuba (Gundlach 1891, Leng and Mutchler 1914, Blackwelder 1944, Ratcliffe and Cave 2008), Puerto Rico (Smyth 1915, 1916), and Guatemala (Casey 1915, Endrődi 1966, 1985a) were not verified by major faunistic studies (Ratcliffe et al. 2013, Ratcliffe and Cave 2015).

### Dyscinetus olivaceus

Taxon classificationAnimaliaColeopteraScarabaeidae

Höhne, 1923

Dyscinetus olivaceus Höhne, 1923a: 252–253 [original combination].

#### Types.

Lectotype ♂ at ZMHB (Endrődi 1966).

#### Distribution.

BOLIVIA: Beni. BRAZIL: Acre, Pará. COLOMBIA: Antioquia, Boyacá, Santander. FRENCH GUIANA: Cayenne, Roura. PERU: Loreto. SURINAME. TRINIDAD AND TOBAGO: Trinidad. VENEZUELA: Carabobo, Zulia.

#### References.

Höhne 1923a, Arrow 1937b, Blackwelder 1944, Gruner 1971, ICA 1977, Remillet et al. 1982, Endrődi 1966, 1973a, 1985a, Remillet 1988, Pardo-Locarno et al. 1995, Restrepo-Giraldo et al. 2003, Gasca-Álvarez and Amat-García 2010, Joly and Escalona 2010, Touroult et al. 2010, Ponchel 2011, Krajcik 2005, 2012, Breeschoten et al. 2013, López-García et al. 2015, Ratcliffe et al. 2015.

### Dyscinetus ornaticaudus

Taxon classificationAnimaliaColeopteraScarabaeidae

Ratcliffe, 1986

Dyscinetus ornaticaudus Ratcliffe, 1986: 77, 78–79 [original combination].

#### Types.

Holotype ♂ at UNSM (Ratcliffe 1986).

#### Distribution.

COLOMBIA: Nariño.

#### References.

Ratcliffe 1986, Restrepo-Giraldo et al. 2003, Gasca-Álvarez and Amat-García 2010, Krajcik 2005, 2012.

### Dyscinetus paradytis

Taxon classificationAnimaliaColeopteraScarabaeidae

(Ponchel & Dechambre, 2003)

Chalepides paradytis Ponchel & Dechambre, 2003: 267 [original combination].
Dyscinetus paradytis (Ponchel & Dechambre) [new combination by Joly and Escalona 2010: 206, 212].

#### Types.

Holotype ♂ at MNHN (Ponchel and Dechambre 2003).

#### Distribution.

BOLIVIA: Beni. BRAZIL: Mato Grosso. COLOMBIA: Caquetá. PERU: Loreto. VENEZUELA: Amazonas, Bolívar, Delta Amacuro, Guárico.

#### References.

Ponchel and Dechambre 2003, Joly and Escalona 2010, Krajcik 2005, 2012, Ratcliffe et al. 2015.

### Dyscinetus picipes

Taxon classificationAnimaliaColeopteraScarabaeidae

(Burmeister, 1847)

Chalepus picipes Burmeister, 1847: 79–80 [original combination].
Dyscinetus picipes (Burmeister) [new combination by Harold 1869a: 123].
Dyscinetus (Dyscinetus) picipes (Burmeister) [new subgeneric classification by Casey 1915: 169].
Dyscinetus picipes (Burmeister) [removal of subgeneric classification by Arrow 1937b: 18].
**syn.** Chalepus geminatus Jacquelin du Val, 1857: 54 [original combination].
Chalepus picipes Burmeister [synonymy by Chevrolat 1865: 31].
**syn.** Chalepus obsoletus LeConte, 1854: 222 [original combination].
Dyscinetus obsoletus (LeConte) [new combination by Harold 1869a: 123].
Dyscinetus picipes (Burmeister) [synonymy by Saylor 1945: 380].
**syn.** Dyscinetus (Dyscinetus) ebeninus Casey, 1915: 169 [original combination].
Dyscinetus picipes (Burmeister) [synonymy by Chapin 1932: 293].
Dyscinetus puncticauda Casey [synonymy by Arrow 1937b: 18].
Dyscinetus picipes (Burmeister) [synonymy by Blackwelder 1944: 253].
**syn.** Dyscinetus (Dyscinetus) laevissimus Casey, 1915: 167 [original combination].
Dyscinetus obsoletus (LeConte) [synonymy by Arrow 1937b: 18].
Dyscinetus picipes (Burmeister) [synonymy Saylor 1945: 380].
**syn.** Dyscinetus (Dyscinetus) obsidianus Casey, 1915: 168 [original combination].
Dyscinetus obsidianus Casey [removal of subgeneric classification by Arrow 1937b: 18].
Dyscinetus picipes (Burmeister) [synonymy by Endrődi 1966: 395].
**syn.** Dyscinetus (Dyscinetus) obsoletus gilanus Casey, 1915: 168 [original combination].
Dyscinetus obsoletus var. gilanus Casey [new infrasubspecific status by Blackwelder 1944: 253].
Dyscinetus picipes (Burmeister) [synonymy by Saylor 1945: 380].
**syn.** Dyscinetus picipes Bates, 1888: 312 [original combination, homonym of Dyscinetus picipes (Burmeister)].
Dyscinetus picipes (Burmeister) [synonymy by Chapin 1932: 293].
Dyscinetus punctipes Saylor, 1945: 380 [new replacement name for Dyscinetus picipes Bates].
**syn.** Dyscinetus picipes puertoricensis Chalumeau, 1982: 342 [original combination].
Dyscinetus picipes (Burmeister) [synonymy by Ratcliffe and Cave 2015: 132].
**syn.** Dyscinetus puncticauda Casey, 1909: 282–283 [original combination].
Dyscinetus picipes (Burmeister) [synonymy by Chapin 1932: 293].
**syn.** Dyscinetus (Dyscinetus) *subquadratus* Casey, 1915: 166 [original combination].

Dyscinetus picipes (Burmeister) [synonymy by Chapin 1932: 293].
Dyscinetus puncticauda Casey [synonymy by Arrow 1937b: 18].
Dyscinetus picipes (Burmeister) [synonymy by Blackwelder 1944: 253].

#### Types.

Endrődi (1966) commented that he doubted the *D. picipes* specimens in the Burmeister Collection at MLUH were the types. Chalumeau (1982) later designated a *Chalepus picipes* Burmeister lectotype ♂ at MLUH. Endrődi (1966) did not examine the types of *C. geminatus.* Type of *C. obsoletus* at MCZ (Endrődi 1966). Type of *D. picipes* Bates at BMNH (Endrődi 1966). The types of the Casey synonyms are at USNM (Endrődi 1966).

#### Distribution.

ANTIGUA AND BARBUDA: Barbuda. BAHAMAS: Great Inagua. CUBA: Artemisa, Camagüey, Ciego de Ávila, Cienfuegos, Granma, Guantánamo, Holguin, Isla de a Juventud, La Habana, Matanzas, Mayabeque, Pinar del Río, Sancti Spíritus, Santiago de Cuba, Villa Clara. DOMINICAN REPUBLIC: La Vega, San Pedro de Macorís, Santo Domingo. GUADELOUPE: Gourbeyre, Marie-Galante, Pointe-à-Pitre, Saint-Claude, Trois-Rivières. HAITI: Ouest. MARTINIQUE. MEXICO: Aguascalientes, Coahuila, Distrito Federal, Durango, Estado de México, Guerrero, Hidalgo, Jalisco, Michoacán, Nayarit, Nuevo León, Puebla, Querétaro, San Luis Potosí, Sinaloa, Tabasco, Tamaulipas, Veracruz. PUERTO RICO: Adjuntas, Aibonito, Añasco, Arecibo, Barceloneta, Bayamón, Cabo Rojo, Carolina, Cayey, Comerio, Dorado, Fajardo, Guánica, Hormigueros, Humacao, Lajas, Loiza, Mayagüez, Orocovis, Ponce, Río Grande, San Germán, San Juan, San Sebastián, Toa Baja, Vega Baja, Yabucoa, Yauco. UNITED STATES: Arizona, Colorado, Iowa, Kansas, Missouri, Montana, Nebraska, New Mexico, Oklahoma, South Dakota, Texas.

#### References.

Sturm 1843, Burmeister 1847, LeConte 1854, Jacquelin du Val 1857, Harold 1869a, Chevrolat 1865, Crotch 1873, Stahl 1882, Henshaw 1885, Bates 1888, Fleutiaux and Sallé 1889, Gundlach 1891, Casey 1909, 1915, Leng and Mutchler 1914, 1917, Leng 1920, Dawson 1922, Stahl and Scaramuzza 1929, Chapin 1932, Arrow 1937b, Blackwelder 1944, Saylor 1945, Paulian 1947, Blackwelder and Blackwelder 1948, Wolcott 1936, 1937, 1948, Barry 1951, Gibson and Carrillo 1959, Van Dinther 1960, Ritcher and Baker 1974, Bruner et al. 1975, Gruner 1975, Kirk and Balsbaugh 1975, Morón and Zaragoza 1976, Chalumeau and Gruner 1977, Martínez 1978b, Chalumeau 1982, Endrődi 1966, 1985a, Maes 1987, 1994, Hardy 1991, Morón et al. 1996, Poole and Gentili 1996, Ratcliffe and Morón 1997, González et al. 1998, Dechambre 1999, Navarrete-Heredia et al. 2001, Ratcliffe 1991, 2002b, Riley and Wolfe 2003, Fernández García 2006, Ratcliffe and Paulsen 2008, Smith 2003, 2009, Krajcik 2005, 2012, Breeschoten et al. 2013, Lugo et al. 2013, Ratcliffe et al. 2013, Ratcliffe and Cave 2008, 2015, 2017.

#### Remarks.

*Dyscinetus picipes* was reported from Nicaragua (Maes 1987, 1994), but major faunistic studies did not find additional specimens (Ratcliffe and Cave 2006).

### Dyscinetus plicatus

Taxon classificationAnimaliaColeopteraScarabaeidae

(Burmeister, 1847)

Chalepus plicatus Burmeister, 1847: 80–81 [original combination].
Dyscinetus plicatus (Burmeister) [new combination by Harold 1869a: 123].

#### Types.

Endrődi (1966) did not find any type material of this species.

#### Distribution.

BRAZIL: Mato Grosso, Paraná, São Paulo.

#### References.

Burmeister 1847, Harold 1869a, Arrow 1937b, Blackwelder 1944, Endrődi 1966, 1985a, Gottsberger 1989, Grossi et al. 2011, Krajcik 2005, 2012.

### Dyscinetus questeli

Taxon classificationAnimaliaColeopteraScarabaeidae

Chalumeau, 1982

Dyscinetus questeli Chalumeau, 1982: 340–341 [original combination].

#### Types.

Holotype ♂ of *D. questeli* at USNM (Chalumeau 1982).

#### Distribution.

GUADELOUPE: Gourbeyre, Marie-Galante, Petit-Bourg, Sainte-Anne, Saint-Claude, Saint-Louis, Sainte-Rose, Trois-Rivières.

#### References.

Chalumeau 1982, Krajcik 2005, 2012, Ratcliffe and Cave 2015, Peck 2016.

### Dyscinetus rugifrons

Taxon classificationAnimaliaColeopteraScarabaeidae

(Burmeister, 1847)

Chalepus rugifrons Burmeister, 1847: 80 [original combination].
Dyscinetus rugifrons (Burmeister) [new combination by Harold 1869a: 123].
**syn.** Chalepus planatus Burmeister, 1847: 80 [original combination].
Dyscinetus planatus (Burmeister) [new combination by Harold 1869a: 123].
Dyscinetus rugifrons (Burmeister) [synonymy by Endrődi 1966: 396].
**syn.** Euetheola sinyaevi Prokofiev, 2012: 8–10 [original combination].
Dyscinetus rugifrons (Burmeister) [synonymy by Prokofiev 2013: 131].

#### Types.

Lectotype ♂ of *C. rugifrons* and lectotype ♂ of *C. planatus* both at MLUH (Endrődi 1966). Holotype ♂ of *E. sinyaevi* at IEE (Prokofiev 2012).

#### Distribution.

ARGENTINA: Buenos Aires, Chaco, Córdoba, Entre Ríos, Formosa, Jujuy, Misiones, Santa Fe, Tucumán. BOLIVIA: Cochabamba, Santa Cruz. BRAZIL: Bahia, Espírito Santo, Minas Gerais, Goiás, Paraná, Pernambuco, Rio de Janeiro, Rio Grande do Sul, Santa Catarina, São Paulo. PARAGUAY: Guairá, Itapúa. URUGUAY: Canelones, Montevideo. VENEZUELA: Bolívar.

#### References.

Burmeister 1847, Reiche 1859, Harold 1869a, Steinheil 1874, Ohaus 1900, 1909, Tremoleras 1910, Luederwaldt 1915, Bosq 1936, Arrow 1937b, Anonymous 1900, 1940, Blackwelder 1944, Guimarães 1944, Guérin 1953, Silveira Guido 1965, Perkins 1974, Vidal et al. 1979, Endrődi 1966, 1969a, 1985a, Dennis and Knutson 1988, Costilla 1991, Lachaume 1992, Dechambre 1999, Vincini et al. 2000, Mauco and Favero 2004, Riehs 2005, Soave et al. 2006, Vitorino et al. 2008, Marques and Gil-Santana 2009, Joly and Escalona 2002b, 2010, Berón and Favero 2010, Krajcik 2005, 2012, Breeschoten et al. 2013, Cherman et al. 2013, Prokofiev 2012, 2013, 2014, Albuquerque et al. 2016.

### Dyscinetus sculptus

Taxon classificationAnimaliaColeopteraScarabaeidae

Dupuis, 2006

Dyscinetus sculptus Dupuis, 2006: 310–312 [original combination].

#### Types.

Holotype ♂ at FDPC (Dupuis 2006).

#### Distribution.

BOLIVIA: Santa Cruz.

#### References.

Dupuis 2006, Krajcik 2012.

### Dyscinetus subsericeus

Taxon classificationAnimaliaColeopteraScarabaeidae

(Burmeister, 1847)

Chalepus subsericeus Burmeister, 1847: 81 [original combination].
Dyscinetus subsericeus (Burmeister) [new combination by Harold 1869a: 123].

#### Types.

Lectotype ♂ at MLUH (Endrődi 1966).

#### Distribution.

BRAZIL.

#### References.

Burmeister 1847, Harold 1869a, Arrow 1937b, Blackwelder 1944, Endrődi 1966, 1985a, Krajcik 2005, 2012.

### Genus Erioscelis

Taxon classificationAnimaliaColeopteraScarabaeidae

Burmeister, 1847

Erioscelis Burmeister, 1847: 72–73 [original usage].

#### Type species.

*Apogonia emarginata* Mannerheim, 1829, by monotypy.

#### Keys.

Saylor 1946, Endrődi 1966, 1985a, Ratcliffe 1985, Maes 1994 (Nicaragua), Jameson et al. 2002, Ratcliffe 2003 (Costa Rica and Panama), Ratcliffe and Cave 2006 (Honduras, Nicaragua, and El Salvador), Gasca-Álvarez and Amat-García 2010 (Colombia).

#### Valid taxa.

5 species.

### Erioscelis columbica

Taxon classificationAnimaliaColeopteraScarabaeidae

Endrődi, 1966

Erioscelis columbica Endrődi, 1966: 410, 413–414 [original combination].

#### Types.

Holotype ♂ at HNHM (Endrődi collection) (Endrődi 1966).

#### Distribution.

COSTA RICA: Heredia, Limón. COLOMBIA: Chocó, Cundinamarca, Meta. NICARAGUA: Jinotega, RAA Norte. PANAMA: Bocas del Toro, Colón, Darien, Panamá.

#### References.

Endrődi 1966, 1985a, Young 1986, 1988a, 1988b, 1990, Maes et al. 1997, Beath 1998, 1999, Grayum 1996, Croat 1997, Morón 1997, Ratcliffe 2002a, 2003, Ratcliffe and Cave 2006, Gasca-Álvarez and Amat-García 2010, Neita-Moreno 2011, Krajcik 2005, 2012, Moore 2012, Moore and Jameson 2013.

### Erioscelis emarginata

Taxon classificationAnimaliaColeopteraScarabaeidae

(Mannerheim, 1829)

Apogonia emarginata Mannerheim, 1829: 54–55 [original combination].
Erioscelis emarginata (Mannerheim) [new combination by Burmeister 1847: 73].

#### Types.

Type at ZMH (Endrődi 1966).

#### Distribution.

ARGENTINA: Misiones. BRAZIL: Minas Gerais, Pará, Paraná, Rio de Janeiro, Rio Grande do Sul, São Paulo, Santa Catarina. ECUADOR. FRENCH GUIANA: Cayenne. PARAGUAY: Concepción.

#### References.

Mannerheim 1829, Dejean 1821, 1833, 1836b, Burmeister 1847, Harold 1869b, Schrottky 1908, 1910, Strand 1912, Luederwaldt 1915, 1916, Arrow 1937b, Ogloblin 1941, Blackwelder 1944, Saylor 1946, Martínez 1968a, Gruner 1971, Gottsberger and Amaral 1984, Endrődi 1966, 1985a, Gottsberger and Silberbauer-Gottsberger 1991, Weber et al. 2001, Riehs 2005, Weber 2008, Dötterl et al. 2012, Krajcik 2005, 2012, Breeschoten et al. 2013, Gottsberger et al. 2013, Moore and Jameson 2013, Maia et al. 2014, Pereira et al. 2014, Maldonado et al. 2015, Gottsberger 1986, 1990, 2016.

### Erioscelis peruana

Taxon classificationAnimaliaColeopteraScarabaeidae

Saylor, 1946

Erioscelis peruana Saylor, 1946: 63–65 [original combination].

#### Types.

Holotype ♂ at CAS (Saylor Collection) (Endrődi 1966).

#### Distribution.

PERU: Ucayali.

#### References.

Saylor 1946, Endrődi 1966, 1985a, Krajcik 2005, 2012, Ratcliffe et al. 2015.

### Erioscelis proba

Taxon classificationAnimaliaColeopteraScarabaeidae

(Sharp, 1877)

Cyclocephala proba Sharp, 1877: 135–136 [original combination].
Erioscelis proba (Sharp) [new combination by Endrődi 1966: 410, 412].
**syn.** Dyscinetus curtus Kirsch, 1873: 345–346 [original combination].
Erioscelis proba (Sharp) [synonymy and *nomen oblitum* by Endrődi 1966: 412].
**syn.** Erioscelis obtusa Prell, 1914: 197–198 [original combination].
Erioscelis proba (Sharp) [synonymy by Endrődi 1966: 412].

#### Types.

Lectotype ♂ *E. proba* at MNHN (Endrődi 1966). Type of *D. curtus* at MTD (Endrődi 1966). Holotype of *E. obtusa* at ZMHB (Endrődi 1966).

#### Distribution.

ARGENTINA: Salta. BOLIVIA: Cochabamba, La Paz. BRAZIL: Amazonas. COLOMBIA. ECUADOR: Pastaza. FRENCH GUIANA: Kourou, Sinnamary. PERU: Junín, Pasco.

#### References.

Kirsch 1873, Sharp 1877, Bates 1888, Prell 1914, Höhne 1921, Arrow 1937b, Blackwelder 1944, Saylor 1946, Martínez 1964, Pike et al. 1976, Endrődi 1966, 1985a, Maes 1994, Gibernau et al. 2003, Gasca-Álvarez and Amat-García 2010, Krajcik 2005, 2012, Breeschoten et al. 2013, Moore and Jameson 2013, Ponchel 2006, 2011, 2015, Ratcliffe et al. 2015, Gibernau 2015a, b.

#### Remarks.

*Erioscelis proba* was reported from Nicaragua (Bates 1888, Maes 1994), but major faunistic studies did not find further specimens (Ratcliffe and Cave 2006).

### Erioscelis sobrina

Taxon classificationAnimaliaColeopteraScarabaeidae

Höhne, 1921

Erioscelis sobrina Höhne, 1921: 108–109 [original combination].

#### Types.

Lectotype ♂ at ZMHB (Endrődi 1966).

#### Distribution.

BRAZIL: Pernambuco. COLOMBIA. PANAMA: Panamá. VENEZUELA: Carabobo.

#### References.

Höhne 1921, Arrow 1937b, Blackwelder 1944, Roze 1955, Endrődi 1966, 1985a, Ratcliffe 2003, Krajcik 2005, 2012.

### Genus Harposceles

Taxon classificationAnimaliaColeopteraScarabaeidae

Burmeister, 1847

Harposceles Burmeister, 1847: 22, 34 [original usage].

#### Type species.

*Harposceles paradoxus* Burmeister 1847, by monotypy.

#### Keys.

Endrődi 1966, 1985a, Ratcliffe 1985, Jameson et al. 2002.

#### Valid taxa.

1 species.

### Harposceles paradoxus

Taxon classificationAnimaliaColeopteraScarabaeidae

Burmeister, 1847

Harposceles paradoxus Burmeister, 1847: 35 [original combination].

#### Types.

Lectotype ♂ at MNHN (Endrődi and Dechambre 1976).

#### Distribution.

BRAZIL: Amazonas, Rondônia. ECUADOR: Sucumbíos. FRENCH GUIANA: Cayenne, Roura, St.-Laurent du Maroni. PERU: Loreto, Madre de Dios, San Martín. SURINAME: Brokopondo.

#### References.

Burmeister 1847, Harold 1869b, Arrow 1937b, Blackwelder 1944, Endrődi and Dechambre 1976, Dechambre 1979a, Endrődi 1966, 1985a, Couturier and Kahn 1992, Lachaume 1992, Andreazze 2001, Andreazze and da Silva Motta 2002, Touroult et al. 2010, Ponchel 2011, Krajcik 2005, 2012, Saltin and Ratcliffe 2012, Breeschoten et al. 2013, Ratcliffe et al. 2015, Hielkema 2017.

#### Remarks.

Endrődi and Dechambre (1976) reported *H. paradoxus* from Cali (Valle del Cauca Department), Colombia. Saltin and Ratcliffe (2012) considered these data as likely erroneous.

### Genus Peltonotus

Taxon classificationAnimaliaColeopteraScarabaeidae

Burmeister, 1847

Peltonotus Burmeister, 1847: 75 [original usage].

#### Type species.

*Peltonotus morio* Burmeister, 1847, by monotypy.

#### Keys.

Arrow 1910, 1917, Paulian 1958, Jameson and Wada 2004, 2009, Jameson and Jákl 2010 (Sumatra), Jameson and Drumont 2013.

#### Valid taxa.

25 species.

### Peltonotus adelphosimilis

Taxon classificationAnimaliaColeopteraScarabaeidae

Jameson & Wada, 2004

Peltonotus adelphosimilis Jameson & Wada, 2004: 11, 12, 14–16 [original combination].

#### Types.

Holotype ♂ at WADA (Jameson and Wada 2004).

#### Distribution.

INDONESIA: West Kalimantan.

#### References.

Jameson and Wada 2004, 2009, Krajcik 2005, 2012, Breeschoten et al. 2013, Jameson and Drumont 2013.

### Peltonotus animus

Taxon classificationAnimaliaColeopteraScarabaeidae

Jameson & Wada, 2009

Peltonotus animus Jameson & Wada, 2009: 4–6 [original combination].

#### Types.

Holotype ♂ at WADA (Jameson and Wada 2009).

#### Distribution.

INDONESIA: West Sumatra.

#### References.

Jameson and Wada 2009, Jameson and Jákl 2010, Krajcik 2012, Breeschoten et al. 2013, Jameson and Drumont 2013.

### Peltonotus brunnipennis

Taxon classificationAnimaliaColeopteraScarabaeidae

Benderitter, 1934

Peltonotus brunnipennis Benderitter, 1934: 255–256 [original combination].

#### Types.

Holotype ♂ at MNHN (Jameson and Wada 2004).

#### Distribution.

MALAYSIA: Sabah, Sarawak.

#### References.

Benderitter 1934, Machatschke 1972, Jameson and Wada 2004, 2009, Krajcik 2005, 2012.

### Peltonotus cybele

Taxon classificationAnimaliaColeopteraScarabaeidae

Jameson & Wada, 2009

Peltonotus cybele Jameson & Wada, 2009: 6–8 [original combination].

#### Types.

Holotype ♀ at WADA (Jameson and Wada 2009).

#### Distribution.

INDONESIA: West Sumatra.

#### References.

Jameson and Wada 2009, Jameson and Jákl 2010, Krajcik 2012, Breeschoten et al. 2013, Jameson and Drumont 2013.

### Peltonotus deltamentum

Taxon classificationAnimaliaColeopteraScarabaeidae

Jameson & Wada, 2004

Peltonotus deltamentum Jameson & Wada, 2004: 9, 18–19 [original combination].

#### Types.

Holotype ♂ at WADA (Jameson and Wada 2004).

#### Distribution.

INDONESIA: West Kalimantan.

#### References.

Jameson and Wada 2004, 2009, Krajcik 2005, 2012, Breeschoten et al. 2013, Jameson and Drumont 2013.

### Peltonotus favonius

Taxon classificationAnimaliaColeopteraScarabaeidae

Jameson & Wada, 2009

Peltonotus favonius Jameson & Wada, 2009: 8–11 [original combination].

#### Types.

Holotype ♂ at NSMT (Jameson and Wada 2009).

#### Distribution.

MYANMAR: Kachin. VIETNAM: Lâm Đồng.

#### References.

Jameson and Wada 2009, Krajcik 2012, Breeschoten et al. 2013, Jameson and Drumont 2013.

### Peltonotus fujiokai

Taxon classificationAnimaliaColeopteraScarabaeidae

Jameson & Wada, 2004

Peltonotus fujiokai Jameson & Wada, 2004: 10, 12, 19–21 [original combination].

#### Types.

Holotype ♂ at WADA (Jameson and Wada 2004).

#### Distribution.

INDONESIA: West Kalimantan. MALAYSIA: Sabah.

#### References.

Jameson and Wada 2004, 2009, Krajcik 2005, 2012, Jameson and Drumont 2013.

### Peltonotus gracilipodus

Taxon classificationAnimaliaColeopteraScarabaeidae

Jameson & Wada, 2004

Peltonotus gracilipodus Jameson & Wada, 2004: 10, 11, 21–23 [original combination].

#### Types.

Holotype ♂ at WADA (Jameson and Wada 2004).

#### Distribution.

INDONESIA: North Sumatra, West Sumatra.

#### References.

Jameson and Wada 2004, 2009, Jameson and Jákl 2010, Krajcik 2005, 2012, Breeschoten et al. 2013, Jameson and Drumont 2013.

### Peltonotus karubei

Taxon classificationAnimaliaColeopteraScarabaeidae

Muramoto, 2000

Peltonotus karubei Muramoto, 2000: 9–11 [original combination].

#### Types.

Holotype ♂ at Muramoto Collection (Jameson and Wada 2004).

#### Distribution.

VIETNAM: Lâm Đồng.

#### References.

Muramoto 2000, Jameson and Wada 2004, 2009, Breeschoten et al. 2013, Jameson and Drumont 2013.

### Peltonotus kyojinus

Taxon classificationAnimaliaColeopteraScarabaeidae

Jameson & Wada, 2004

Peltonotus kyojinus Jameson & Wada, 2004: 12, 25–26 [original combination].

#### Types.

Holotype ♂ at FUJI (Jameson and Wada 2004).

#### Distribution.

INDONESIA: West Kalimantan.

#### References.

Jameson and Wada 2004, 2009, Krajcik 2005, 2012, Jameson and Drumont 2013.

### Peltonotus malayensis

Taxon classificationAnimaliaColeopteraScarabaeidae

Arrow, 1910

Peltonotus malayensis Arrow, 1910: 155–156 [original combination].

#### Types.

Lectotype ♀ at BMNH (Jameson and Wada 2004).

#### Distribution.

BRUNEI: Temburong. INDONESIA: West Kalimantan. MALAYSIA: Sarawak.

#### References.

Arrow 1910, Ohaus 1918, 1934b, Machatschke 1972, Jameson and Wada 2004, 2009, Krajcik 2005, 2012, Breeschoten et al. 2013, Jameson and Drumont 2013.

### Peltonotus morio

Taxon classificationAnimaliaColeopteraScarabaeidae

Burmeister, 1847

Peltonotus morio Burmeister, 1847: 75 [original combination].
**syn.** Peltonotus morio sawaii Miyake, 2000: 112–113 [original combination].
Peltonotus morio Burmeister [synonymy by Jameson and Wada 2004: 29].

#### Types.

Neotype ♂ of *P. morio* at RIEB (Jameson and Wada 2004). Holotype of *P. morio sawaii* was missing from RIEB (Jameson and Wada 2004).

#### Distribution.

BHUTAN. INDIA: Assam, Manipur, Meghalaya, Sikkim, West Bengal. MYANMAR: Chin, Lumbini, Tanintharyi. NEPAL: Gandaki, Narayani. THAILAND: Chiang Mai. VIETNAM: Lào Cai.

#### References.

Burmeister 1847, Harold 1869b, Seidlitz 1905, Arrow 1910, 1917, Lucas 1918b, Ohaus 1934b, Abdullah and Roohi 1969, Machatschke 1972, Jameson and Wada 2004, 2009, Krajcik 2005, 2012, Moore 2012, Breeschoten et al. 2013, Jameson and Drumont 2013.

#### Remarks.

*Peltonotus morio* has been reported from Bangladesh (Abdullah and Roohi 1969, Jameson and Wada 2004), but these records need to be confirmed.

### Peltonotus mushiyaus

Taxon classificationAnimaliaColeopteraScarabaeidae

Jameson & Wada, 2009

Peltonotus mushiyaus Jameson & Wada, 2009: 11–12 [original combination].

#### Types.

Holotype ♀ at NSMT (Jameson and Wada 2009).

#### Distribution.

MALAYSIA: Sabah.

#### References.

Jameson and Wada 2009, Krajcik 2012, Jameson and Drumont 2013.

### Peltonotus nasutus

Taxon classificationAnimaliaColeopteraScarabaeidae

Arrow, 1910

Peltonotus nasutus Arrow, 1910: 155 [original combination].

#### Types.

Lectotype ♂ at BMNH (Jameson and Wada 2004).

#### Distribution.

CAMBODIA: Pailin, Pursat, Ratanakiri. CHINA: Guangxi, Guizhou, Yunnan. LAOS: Attapeu, Bokeo, Champasak, Luang Namtha, Vientiane, Xiangkhouang. MYANMAR: Rakhine. THAILAND: Chiang Mai, Chiang Rai, Chumphon, Kanchanaburi, Loei, Mae Hong Son, Nakhon Ratchasima, Nan, Prachuap Khiri Khan, Tak. VIETNAM.

#### References.

Fairmaire 1904, Arrow 1910, 1917, Ohaus 1918, 1934b, Abdullah and Roohi 1969, Machatschke 1972, 1974, Jameson and Wada 2004, 2009, Grimm 2009, Danell 2010, Jameson and Drumont 2013, Krajcik 2005, 2012, Breeschoten et al. 2013, Sima and Srivastava 2014a, 2014b, 2016, Sites 2017.

#### Remarks.

*Peltonotus nasutus* may occur in Bangladesh and Nepal, but these records may also refer to misidentified *P. morio* (Ohaus 1918, Abdullah and Roohi 1969, Machatschke 1972, 1974, Jameson and Wada 2004). *Peltonotus nasutus* was reported from Rajasthan state, India (Sima and Srivastava 2014a, 2014b, 2016). These data should be re-evaluated as they would represent a significant western extension of the known range of *Peltonotus*.

### Peltonotus nethis

Taxon classificationAnimaliaColeopteraScarabaeidae

Jameson & Wada, 2004

Peltonotus nethis Jameson & Wada, 2004: 11, 33–34 [original combination].

#### Types.

Holotype ♀ at ZMHB (Jameson and Wada 2004).

#### Distribution.

MALAYSIA: Sabah.

#### References.

Jameson and Wada 2004, 2009, Krajcik 2005, 2012, Jameson and Drumont 2013.

### Peltonotus podocrassus

Taxon classificationAnimaliaColeopteraScarabaeidae

Jameson & Wada, 2004

Peltonotus podocrassus Jameson & Wada, 2004: 10, 11, 34–37 [original combination].

#### Types.

Holotype ♂ at UNSM (Jameson and Wada 2004).

#### Distribution.

MALAYSIA: Pahang, Perak.

#### References.

Jameson and Wada 2004, 2009, Krajcik 2005, 2012, Breeschoten et al. 2013, Jameson and Drumont 2013.

### Peltonotus pruinosus

Taxon classificationAnimaliaColeopteraScarabaeidae

Arrow, 1910

Peltonotus pruinosus Arrow, 1910: 156–157 [original combination].

#### Types.

Holotype ♀ at BMNH (Jameson and Wada 2004).

#### Distribution.

INDIA: Assam.

#### References.

Arrow 1910, 1917, Ohaus 1918, 1934b, Machatschke 1972, Jameson and Wada 2004, 2009, Krajcik 2005, 2012, Jameson and Drumont 2013.

### Peltonotus rubripennis

Taxon classificationAnimaliaColeopteraScarabaeidae

Miyake & Yamaya, 1994

Peltonotus rubripennis Miyake & Yamaya, 1994: 42–43 [original combination].

#### Types.

Holotype ♀ at Yamaya Collection (Nigata, Japan) (Jameson and Wada 2004).

#### Distribution.

MALAYSIA: Sabah, Sarawak.

#### References.

Miyake and Yamaya 1994, Jameson and Wada 2004, 2009, Krajcik 2005, 2012, Breeschoten et al. 2013, Jameson and Drumont 2013.

### Peltonotus silvanus

Taxon classificationAnimaliaColeopteraScarabaeidae

Jameson & Wada, 2004

Peltonotus silvanus Jameson & Wada, 2004: 10, 12, 40–42 [original combination].

#### Types.

Holotype ♂ at FUJI (Jameson and Wada 2004).

#### Distribution.

INDONESIA: West Kalimantan. MALAYSIA: Sarawak.

#### References.

Jameson and Wada 2004, 2009, Krajcik 2005, 2012, Breeschoten et al. 2013, Jameson and Drumont 2013.

### Peltonotus similis

Taxon classificationAnimaliaColeopteraScarabaeidae

Arrow, 1931

Peltonotus similis Arrow, 1931: 612 [original combination].
**syn.** Peltonotus sakaii Miyake and Yamaya, 1994: 39–42 [original combination].
Peltonotus similis Arrow [synonymy by Jameson and Wada 2004: 42].

#### Types.

Lectotype ♂ of *P. similis* at BMNH (Jameson and Wada 2004). Holotype of *P. sakaii* at NSMT (Jameson and Wada 2004).

#### Distribution.

MALAYSIA: Sabah, Sarawak.

#### References.

Arrow 1931, Ohaus 1934b, Machatschke 1972, Miyake and Yamaya 1994, Jameson and Wada 2004, 2009, Krajcik 2005, 2012, Breeschoten et al. 2013, Jameson and Drumont 2013.

### Peltonotus sisyrus

Taxon classificationAnimaliaColeopteraScarabaeidae

Jameson & Wada, 2004

Peltonotus sisyrus Jameson & Wada, 2004: 44–46 [original combination].

#### Types.

Holotype ♂ at FUJI (Jameson and Wada 2004).

#### Distribution.

INDONESIA: Aceh.

#### References.

Jameson and Wada 2004, 2009, Jameson and Jákl 2010, Krajcik 2005, 2012, Breeschoten et al. 2013, Jameson and Drumont 2013.

### Peltonotus suehirogarus

Taxon classificationAnimaliaColeopteraScarabaeidae

Jameson & Wada, 2004

Peltonotus suehirogarus Jameson & Wada, 2004: 46–47 [original combination].

#### Types.

Holotype ♀ at WADA (Jameson and Wada 2004).

#### Distribution.

INDONESIA: West Kalimantan. MALAYSIA: Sarawak.

#### References.

Jameson and Wada 2004, 2009, Krajcik 2005, 2012, Jameson and Drumont 2013.

### Peltonotus talangensis

Taxon classificationAnimaliaColeopteraScarabaeidae

Jameson & Jákl, 2010

Peltonotus talangensis Jameson & Jákl, 2010: 143, 148–152 [original combination].

#### Types.

Holotype ♂ at NMPC (Jameson and Jákl 2010).

#### Distribution.

INDONESIA: West Sumatra.

#### References.

Jameson and Jákl 2010, Krajcik 2012, Breeschoten et al. 2013, Jameson and Drumont 2013.

### Peltonotus tigerus

Taxon classificationAnimaliaColeopteraScarabaeidae

Jameson & Wada, 2009

Peltonotus tigerus Jameson and Wada 2009: 12–14 [original combination].

#### Types.

Holotype ♀ at QSBG (Jameson and Wada 2009).

#### Distribution.

THAILAND: Phetchabun.

#### References.

Jameson and Wada 2009, Krajcik 2012, Jameson and Drumont 2013.

### Peltonotus vittatus

Taxon classificationAnimaliaColeopteraScarabaeidae

Arrow, 1910

Peltonotus vittatus Arrow, 1910: 157 [original combination].

#### Types.

Holotype ♀ at MNHN (Jameson and Wada 2004).

#### Distribution.

MALAYSIA: Sabah, Sarawak.

#### References.

Arrow 1910, Ohaus 1918, 1934b, Machatschke 1972, Jameson and Wada 2004, 2009, Krajcik 2005, 2012, Breeschoten et al. 2013, Jameson and Drumont 2013.

### Genus Rutelorcytes

Taxon classificationAnimaliaColeopteraScarabaeidae

Arrow, 1908

Ruteloryctes Arrow, 1908: 335–336 [original usage].

#### Type species.

*Ruteloryctes tristis* Arrow, 1908, by monotypy.

#### Keys.

Janssens 1942, Paulian 1954 (Sub-Saharan Africa), Endrődi 1960 (Southern Africa), 1966, 1985a, Jameson et al. 2002.

#### Valid taxa.

2 species.

### Ruteloryctes bis

Taxon classificationAnimaliaColeopteraScarabaeidae

Dechambre, 2006

Ruteloryctes bis Dechambre, 2006b: 53–55 [original combination].

#### Types.

Holotype ♂ at RPDC (Dechambre 2006b).

#### Distribution.

CAMEROON: Centre Province.

#### References.

Dechambre 2006b, Krajcik 2012.

### Ruteloryctes morio

Taxon classificationAnimaliaColeopteraScarabaeidae

(Fabricius, 1798)

Melolontha morio Fabricius, 1798: 131 [original combination].
Chalepus morio (Fabricius) [new combination by Hope 1837: 71].
Heteronychus morio (Fabricius) [new combination by Burmeister 1847: 95].
Ruteloryctes morio (Fabricius) [new combination by Prell 1937a: 188].
**syn.** Ruteloryctes tristis Arrow, 1908: 336 [original combination].
Ruteloryctes morio (Fabricius) [synonymy by Paulian 1954: 1123].
**syn.** Melolontha hottentotta Schönherr, 1817: 187 [original combination].
Heteronychus hottentotta (Schönherr) [new combination by Burmeister 1847: 95].
Ruteloryctes morio (Fabricius) [synonymy by Arrow 1937b: 20].

#### Types.

Endrődi (1966) stated that there was a “type” at MNHN (Desfontaines Collection) without further details. Types of *R. tristis* and *M. hottentotta* were not reported on by Endrődi (1966). Arrow (1908) stated that he had specimens of *R. tristis* at the BMNH and in the Oberthür Collection. Prell (1937a) examined a female specimen of *M. morio* at ZMUK (now at ZMUC), and it is possible that this Fabrician specimen is still in that collection.

#### Distribution.

ANGOLA. BÉNIN. CAMEROON. CHAD: Barh Köh. CÔTE D’IVOIRE: Zanzan. DEMOCRATIC REPUBLIC OF THE CONGO: Bandundu, Bas-Congo, Équateur, Orientale. GUINEA. GUINEA-BISSAU. NIGERIA: Cross River. SENEGAL: Kaolack, Tambacounda. SIERRA LEONE. THE GAMBIA.

#### References.

Fabricius 1798, 1801, Schönherr 1817, Hope 1837, Burmeister 1847, Harold 1869b, Arrow 1908, 1937b, Prell 1937a, Janssens 1942, Burgeon 1947, Paulian 1954, Zimsen 1964, Endrődi 1966, 1971b, 1985a, Krell et al. 2003, Ervik and Knudsen 2003, Hirthe and Porembski 2003, Krajcik 2005, 2012, Breeschoten et al. 2013, Moore and Jameson 2013.

### Genus Stenocrates

Taxon classificationAnimaliaColeopteraScarabaeidae

Burmeister, 1847

Stenocrates Burmeister, 1847: 83–84 [original usage].

#### Type species.

*Scarabaeus laborator* Fabricius, subsequent designation by Casey 1915: 114.

#### Keys.

Endrődi 1966, 1985a, Morón 1979 (Veracruz, Mexico), Morón et al. 1985 (Chiapas, Mexico), Morón et al. 1985 (Jalisco, Mexico), Jameson et al. 2002, Ratcliffe 2003 (Costa Rica and Panama), Ratcliffe and Cave 2006 (Honduras, Nicaragua, and El Salvador), Ratcliffe et al. 2013 (Mexico, Guatemala, and Belize), Ratcliffe and Cave 2015 (West Indies), Ratcliffe 2015 (catalog), Pardo-Locarno 2013 (Valle del Cauca, Colombia), Villalobos-Moreno et al. 2017 (Colombia), Dupuis 2017 (French Guiana).

#### Valid taxa.

52 species and subspecies

### Stenocrates agricola

Taxon classificationAnimaliaColeopteraScarabaeidae

Dechambre & Hardy, 2004

Stenocrates agricola Dechambre & Hardy, 2004: 210 [original combination].

#### Types.

Holotype ♂ at CMNC (Henry and Anne Howden Collection) (Dechambre and Hardy 2004).

#### Distribution.

ARGENTINA. PARAGUAY.

#### References.

Dechambre and Hardy 2004, Krajcik 2012, Ratcliffe 2015.

### Stenocrates amazonicus

Taxon classificationAnimaliaColeopteraScarabaeidae

Ratcliffe, 1978

Stenocrates amazonicus Ratcliffe, 1978: 491–492 [original combination].

#### Types.

Holotype ♂ at INPA (Ratcliffe 1978).

#### Distribution.

BRAZIL: Amazonas. SURINAME.

#### References.

Endrődi 1985a, Krajcik 2005, 2012, Ratcliffe 1978, 2015.

### Stenocrates ariasi

Taxon classificationAnimaliaColeopteraScarabaeidae

Ratcliffe, 1978

Stenocrates ariasi Ratcliffe, 1978: 492–492 [original combination].

#### Types.

Holotype ♂ at INPA (Ratcliffe 1978).

#### Distribution.

BOLIVIA: Amazonas. BRAZIL.

#### References.

Endrődi 1985a, Krajcik 2005, 2012, Ratcliffe 1978, 2015.

### Stenocrates batesi

Taxon classificationAnimaliaColeopteraScarabaeidae

Dechambre, 1979

Stenocrates batesi Dechambre, 1979c: 61 [original combination].

#### Types.

Holotype ♂ at MNHN (Dechambre 1979c).

#### Distribution.

BRAZIL: São Paulo. COLOMBIA. ECUADOR.

#### References.

Dechambre 1979c, Endrődi 1985a, Krajcik 2005, 2012, Ratcliffe 2015.

### Stenocrates beckeri

Taxon classificationAnimaliaColeopteraScarabaeidae

Howden, 1970

Stenocrates beckeri Howden, 1970: 9–10 [original combination].
**syn.** Stenocrates davisorum Endrődi, 1979: 217–218 [original combination].
Stenocrates beckeri Howden [synonymy by Ratcliffe and Cave 2015: 139].

#### Types.

Holotype ♂ of *S. beckeri* at CNC (Howden 1970). Holotype ♂ of *S. davisorum* at USNM (Endrődi 1979).

#### Distribution.

JAMAICA: Clarendon, Manchester, Portland, St. Catherine, Trelawny, Westmoreland.

#### References.

Howden 1970, Endrődi 1979, 1985a, Krajcik 2005, 2012, Ratcliffe and Cave 2015, Ratcliffe 2015.

### Stenocrates bicarinatus

Taxon classificationAnimaliaColeopteraScarabaeidae

Robinson, 1947

Stenocrates bicarinatus Robinson, 1947: 233–234 [original combination].
**syn.** Stenocrates difficilis Endrődi, 1966: 417, 427 [original combination].
Stenocrates bicarinatus Robinson [synonymy by Ratcliffe 2003: 241].

#### Types.

Holotype ♂ of *S. bicarinatus* at USNM (Mark Robinson Collection) (Robinson 1947). Holotype ♂ of *S. difficilis* at ZSMC (Endrődi 1966).

#### Distribution.

BELIZE: Cayo, Orange Walk, Stann Creek, Toledo. BRAZIL: Pará. COLOMBIA: Cauca, Caquetá, Chocó, Santander, Valle del Cauca. EL SALVADOR: San Salvador. FRENCH GUIANA: St.-Élie. GUATEMALA: Alta Verapaz, Baja Verapaz, Guatemala, Huehuetenango, Izabal, Petén, Santa Rosa. HONDURAS: Atlántida, Comayagua, Cortés, Gracias a Dios, Olancho. MEXICO: Campeche, Chiapas, Guerrero, Jalisco, Oaxaca, Puebla, San Luis Potosí, Tabasco, Veracruz. NICARAGUA: Chontales, Granada, RAA Norte, RAA Sur, Río San Juan. PANAMA: Colón, Darien, Former Canal Zone, Panamá. SURINAME.

#### References.

Robinson 1947, Endrődi 1966, 1969b, 1985a, Dechambre 1979a, Morón et al. 1985, Thomas 1993, Ratcliffe and Morón 1997, Maes et al. 1997, Ratcliffe 1978, 2002a, 2003, Restrepo et al. 2003, Dechambre and Hardy 2004, Pardo-Locarno et al. 2005a, Ratcliffe and Cave 2006, Gasca-Álvarez and Amat-García 2010, Neita-Moreno 2011, Ponchel 2011, Krajcik 2005, 2012, Pardo-Locarno 2013, Ratcliffe et al. 2013, López-García et al. 2015, Ratcliffe 2015, Dupuis 2017.

### Stenocrates bolivianus

Taxon classificationAnimaliaColeopteraScarabaeidae

Dechambre, 1979

Stenocrates bolivianus Dechambre, 1979c: 62 [original combination].

#### Types.

Holotype ♂ at MNHN (Dechambre 1979c).

#### Distribution.

BOLIVIA. BRAZIL.

#### References.

Dechambre 1979c, Krajcik 2005, 2012, Endrődi 1985a, Ratcliffe 2015.

### Stenocrates bollei

Taxon classificationAnimaliaColeopteraScarabaeidae

Dechambre, 1985

Stenocrates bollei Dechambre, 1985: 142 [original combination].

#### Types.

Holotype ♂ at MNHN (Dechambre 1985).

#### Distribution.

BRAZIL. VENEZUELA: Amazonas.

#### References.

Dechambre 1985, Krajcik 2005, 2012, Ratcliffe 2015.

### Stenocrates caiporae

Taxon classificationAnimaliaColeopteraScarabaeidae

Ratcliffe, 2014

Stenocrates caiporae Ratcliffe, 2014: 666–668 [original combination].

#### Types.

Holotype ♂ at UNSM (Ratcliffe 2014).

#### Distribution.

BRAZIL: Amazonas.

#### References.

Ratcliffe 2014, 2015.

### Stenocrates canuli

Taxon classificationAnimaliaColeopteraScarabaeidae

Delgado, 1991

Stenocrates canuli Delgado, 1991: 103–106 [original combination].

#### Types.

Holotype ♂ at MXAL (Delgado 1991).

#### Distribution.

BELIZE: Belize, Orange Walk, Stann Creek, Toledo. EL SALVADOR: San Salvador. GUATEMALA: Alta Verapaz, Chiquimula, Izabal, Petén. HONDURAS: Atlántida, Comayagua, Gracias a Dios. MEXICO: Campeche, Chiapas, Oaxaca, Quintana Roo, Veracruz, Yucatán. NICARAGUA: Jinotega, RAA Sur.

#### References.

Delgado 1991, Ratcliffe and Morón 1997, Ratcliffe and Cave 2006, Krajcik 2005, 2012, Ratcliffe et al. 2013, Ratcliffe 2015.

### Stenocrates carbo

Taxon classificationAnimaliaColeopteraScarabaeidae

Prell, 1937

Stenocrates carbo Prell, 1937c: 9–10 [original combination].

#### Types.

Holotype ♂ at ZMHB (Endrődi 1966).

#### Distribution.

BRAZIL: Amazonas. FRENCH GUIANA. PERU.

#### References.

Prell 1937c, Blackwelder 1944, Dechambre 1979c, 1985, Endrődi 1966, 1985a, Ponchel 2011, Krajcik 2005, 2012, Breeschoten et al. 2013, Ratcliffe 2015, Ratcliffe et al. 2015, Dupuis 2017.

### Stenocrates carinatus

Taxon classificationAnimaliaColeopteraScarabaeidae

Endrődi, 1966

Stenocrates carinatus Endrődi, 1966: 417, 423 [original combination].

#### Types.

Holotype ♂ at HNHM (Endrődi Collection) (Endrődi 1966).

#### Distribution.

BOLIVIA: Beni. BRAZIL: Rio de Janeiro.

#### References.

Endrődi 1966, 1985a, Krajcik 2005, 2012.

### Stenocrates celatus

Taxon classificationAnimaliaColeopteraScarabaeidae

Prell, 1937

Stenocrates celatus Prell, 1937c: 10 [original combination].

#### Types.

Holotype ♂ at ZMHB (Endrődi 1966).

#### Distribution.

BRAZIL: Mato Grosso, Santa Catarina. FRENCH GUIANA. GUYANA. PERU: Junín, Loreto.

#### References.

Prell 1937c, Endrődi 1966, 1967c, 1985a, Krajcik 2005, 2012, Ratcliffe et al. 2013, Ratcliffe 2015.

#### Remarks.

*Stenocrates celatus* was reported from Mexico and the United States (Arizona) (Endrődi 1966, 1985a). These data are erroneous, and *S. celatus* is a South American species (Ratcliffe et al. 2013). Dupuis (2017) does not list *S. celatus* as occurring in French Guiana.

### Stenocrates clipeatus

Taxon classificationAnimaliaColeopteraScarabaeidae

Endrődi, 1966

Stenocrates clipeatus Endrődi, 1966: 417, 424–425 [original combination].

#### Types.

Holotype ♂ at NHMB (Frey Collection) (Endrődi 1966).

#### Distribution.

BOLIVIA: Cochabamba. BRAZIL. COLOMBIA. FRENCH GUIANA. PERU: Loreto.

#### References.

Dechambre 1979c, Endrődi 1966, 1969b, 1985a, Moragues 2010, Ponchel 2011, Krajcik 2005, 2012, Ratcliffe et al. 2015, Ratcliffe 2015.

#### Remarks.

Dupuis (2017) does not list *S. clipeatus* as occurring in French Guiana.

### Stenocrates cognatus

Taxon classificationAnimaliaColeopteraScarabaeidae

Endrődi, 1966

Stenocrates cognatus Endrődi, 1966: 417, 425–426 [original combination].

#### Types.

Holotype ♂ at NHMB (Frey Collection) (Endrődi 1966).

#### Distribution.

COLOMBIA: Cundinamarca.

#### References.

Endrődi 1966, 1967c, 1985a, Restrepo et al. 2003, Gasca-Álvarez and Amat-García 2010, Krajcik 2005, 2012, Breeschoten et al. 2013, López-García et al. 2015, Ratcliffe 2015.

### Stenocrates cultor cultor

Taxon classificationAnimaliaColeopteraScarabaeidae

Burmeister, 1847

Stenocrates cultor Burmeister, 1847: 84–85 [original combination].

#### Types.

Lectotype ♂ at MLUH (Endrődi 1966).

#### Distribution.

ARGENTINA. BOLIVIA: La Paz, Santa Cruz. BRAZIL: Bahia, Espírito Santo, Mato Grosso, Pernambuco, Rio de Janeiro, Santa Catarina, São Paulo. ECUADOR: Pichincha. FRENCH GUIANA: Campoi. PARAGUAY: Alta Paraná. PERU. VENEZUELA: Carabobo.

#### References.

Dejean 1833, 1836b, Burmeister 1847, Redtenbacher 1868, Harold 1869b, Arrow 1937b, Blackwelder 1944, Guimarães 1944, Gruner 1971, Endrődi 1966, 1969a, 1985a, Venzon and Pallini Filho 1995, Marques and Gil-Santana 2009, Ponchel 2011, Krajcik 2005, 2012, Ratcliffe et al. 2015, Dupuis 2017.

#### Remarks.

*Stenocrates cultor* was reported from Honduras (Endrődi 1966, 1985a). Subsequent surveys of Central America have not found additional specimens (Ratcliffe and Cave 2006).

### Stenocrates cultor inelegans

Taxon classificationAnimaliaColeopteraScarabaeidae

Arrow, 1913

Stenocrates inelegans Arrow, 1913: 465–466 [original combination].
Stenocrates cultor inelegans Arrow [new subspecies status by Dupuis and Dechambre 1995: 61].
**syn.** Stenocrates carbunculus Prell, 1937c: 10 [original combination].
Stenocrates cultor inelegans Arrow [synonymy by Dupuis and Dechambre 1995: 59].

#### Types.

Type of *S. inelegans* at BMNH (Endrődi 1966). Holotype ♂ of *S. carbunculus* at ZMHB (Endrődi 1966).

#### Distribution.

BOLIVIA: Beni, Cochabamba. BRAZIL: Acre, Amazonas, Goiás Rondônia. COLOMBIA. SURINAME. VENEZUELA.

#### References.

Arrow 1913, 1937b, Prell 1937c, Blackwelder 1944, Gruner 1971, Endrődi 1966, 1967c, 1973a, 1985a, Dupuis and Dechambre 1995, Andreazze and Fonseca 1998, Krajcik 2005, 2012, Breeschoten et al. 2013, Ratcliffe 2015.

#### Remarks.

Gruner (1971) reported *S. cultor inelegans* from French Guiana, but Dupuis (2017) does not list this subspecies as occurring there.

### Stenocrates dubius

Taxon classificationAnimaliaColeopteraScarabaeidae

Endrődi, 1966

Stenocrates dubius Endrődi, 1966: 418, 427–428 [original combination].

#### Types.

Holotype ♂ at BMNH (Endrődi 1966).

#### Distribution.

BOLIVIA: Cochabamba.

#### References.

Endrődi 1966, 1967c, 1985a, Krajcik 2005, 2012, Ratcliffe 2015.

### Stenocrates duplicatus

Taxon classificationAnimaliaColeopteraScarabaeidae

Endrődi, 1967

Stenocrates duplicatus Endrődi, 1967c: 6–8 [original combination].
**syn.** Stenocrates frater Dechambre, 2006a: 19 [original combination].
Stenocrates duplicatus Endrődi [synonymy by Ratcliffe et al. 2013: 257].

#### Types.

Holotype ♂ of *S. duplicatus* at ZMHB (Endrődi 1967c). Holotype ♂ of *S. frater* at RPDC (Dechambre 2006a).

#### Distribution.

ECUADOR: Guayas. GUATEMALA: Petén. MEXICO: Chiapas, Jalisco, Oaxaca, Veracruz.

#### References.

Endrődi 1967c, 1985a, Morón et al. 1988, Thomas 1993, Ratcliffe and Morón 1997, Navarrete-Heredia et al. 2001, Dechambre 2006a, Krajcik 2005, 2012, Ratcliffe et al. 2013, Ratcliffe 2015, Dupuis 2017.

#### Remarks.

*Stenocrates duplicatus* was reported from French Guiana (Moragues 2010), but these specimens were later determined to be a new species, *S. seag* Dupuis (Dupuis 2017).

### Stenocrates eniocanoi

Taxon classificationAnimaliaColeopteraScarabaeidae

Ratcliffe & Cave, 2013

Stenocrates eniocanoi Ratcliffe & Cave, 2013: 252, 258–260 [original combination in Ratcliffe et al. 2013].

#### Types.

Holotype ♂ at UNSM (Ratcliffe et al. 2013).

#### Distribution.

GUATEMALA: Petén. MEXICO: Chiapas.

#### References.

Ratcliffe et al. 2013, Ratcliffe 2015.

### Stenocrates haackae

Taxon classificationAnimaliaColeopteraScarabaeidae

Ratcliffe, 1977

Stenocrates haackae Ratcliffe, 1977: 433– 444 [original combination].

#### Types.

Holotype ♂ at INPA (Ratcliffe 1977).

#### Distribution.

BRAZIL: Amazonas. ECUADOR.

#### References.

Krajcik 2005, 2012, Ratcliffe 1977, 1978, 2014, 2015, Hielkema 2017.

#### Remarks.

See Hielkema (2017) for a discussion on the correct spelling of “*haackae*”.

### Stenocrates hardyi

Taxon classificationAnimaliaColeopteraScarabaeidae

Dechambre, 1985

Stenocrates hardyi Dechambre, 1985: 143 [original combination].

#### Types.

Holotype ♂ at MNHN (Dechambre 1985).

#### Distribution.

COSTA RICA: Alajuela, Heredia, Limón, Puntarenas. NICARAGUA: RAA Norte. PANAMA: Coclé, Colón, Former Canal Zone, Panamá.

#### References.

Dechambre 1985, Maes et al. 1997, Ratcliffe and Cave 2006, Krajcik 2005, 2012, Ratcliffe 2002a, 2003, 2015.

### Stenocrates hastatus

Taxon classificationAnimaliaColeopteraScarabaeidae

Ratcliffe, 2015

Stenocrates hastatus Ratcliffe, 2015: 775–776 [original combination].

#### Types.

Holotype ♂ at UNSM (Ratcliffe 2015).

#### Distribution.

BRAZIL: Esprírito Santo, Rio de Janeiro.

#### References.

Ratcliffe 2015.

### Stenocrates hiekei

Taxon classificationAnimaliaColeopteraScarabaeidae

Endrődi, 1967

Stenocrates hiekei Endrődi, 1967c: 4–5 [original combination].

#### Types.

Holotype ♂ at ZMHB (Endrődi 1967c).

#### Distribution.

VENEZUELA: Carabobo.

#### References.

Endrődi 1967c, 1985a, Krajcik 2005, 2012, Ratcliffe 2015.

### Stenocrates holomelanus

Taxon classificationAnimaliaColeopteraScarabaeidae

(Germar, 1824)

Geotrupes holomelanus Germar, 1824: 116–117 [original combination].
Stenocrates holomelanus (Germar) [new combination by Burmeister 1847: 84].
**syn.** Dyscinetus (Dyscinetus) parensis Casey, 1915: 172 [original combination].
Stenocrates holomelanus (Germar) [synonymy by Arrow 1937b: 19].

#### Types.

Endrődi (1966) did not find the type material of *G. holomelanus*. Type of *D. parensis* at USNM (Endrődi 1966).

#### Distribution.

ARGENTINA: Buenos Aires, Chaco. BOLIVIA. BRAZIL: Acre, Amazonas, Bahia, Pará, São Paulo. COLOMBIA: Cundinamarca. ECUADOR: Morona Santiago. FRENCH GUIANA: Cayenne, Kourou, Sinnamary. PARAGUAY: Asunción. SURINAME.

#### References.

Germar 1824, Burmeister 1847, Reiche 1859, Harold 1869b, Casey 1915, Arrow 1937b, Blackwelder 1944, Gruner 1971, Dechambre 1979a, 1985, Endrődi 1966, 1967c, 1969a, 1985a, Restrepo et al. 2003, Gasca-Álvarez and Amat-García 2010, Ponchel 2011, Krajcik 2005, 2012, Breeschoten et al. 2013, Ratcliffe 2015, Albuquerque et al. 2016, Dupuis 2018.

### Stenocrates howdeni

Taxon classificationAnimaliaColeopteraScarabaeidae

Dechambre & Hardy, 2004

Stenocrates howdeni Dechambre & Hardy, 2004: 211[original combination].

#### Types.

Holotype ♂ at CMNC (Dechambre and Hardy 2004).

#### Distribution.

URUGUAY.

#### References.

Dechambre and Hardy 2004, Krajcik 2012, Ratcliffe 2015.

### Stenocrates impeditus

Taxon classificationAnimaliaColeopteraScarabaeidae

Dechambre & Hardy, 2004

Stenocrates impeditus Dechambre and Hardy 2004: 212–213 [original combination].

#### Types.

Holotype ♂ at CMNC (Dechambre and Hardy 2004).

#### Distribution.

BRAZIL: Minas Gerais (Dechambre and Hardy 2004).

#### References.

Dechambre and Hardy 2004, Krajcik 2012, Ratcliffe 2015.

### Stenocrates laborator

Taxon classificationAnimaliaColeopteraScarabaeidae

(Fabricius, 1775)

Scarabaeus laborator Fabricius: 18 [original combination].
Geotrupes laborator (Fabricius) [new combination by Eschscholtz 1822: 121].
Stenocrates laborator (Fabricius) [new combination by Burmeister 1847: 85].
**syn.**Geotrupes globator Thunberg, 1814: 400 [original combination].
Stenocrates laborator (Fabricius) [synonymy by Endrődi 1966: 430].
**syn.** Geotrupes thoracicus Eschscholtz, 1818: 453–454 [original combination].
Geotrupes laborator var. thoracicus Eschscholtz [new infrasubspecific status by Eschscholtz 1822: 121].
Stenocrates laborator var. thoracicus (Eschscholtz) [new combination by Arrow 1937b: 19].
Stenocrates laborator (Fabricius) [synonymy by Endrődi 1985a: 184].
**syn.** Stenocrates australis Endrődi, 1973b: 319 [original combination].
Stenocrates laborator (Fabricius) [synonymy by Ratcliffe 2015: 778].

#### Types.

Type of *S. laborator* at BMNH (Endrődi 1966). The types of *G. globator* and *G. thoracicus* were not found by Endrődi (1966). Holotype ♂ of *S. laborator australis* at NHMB (Frey Collection) (Endrődi 1973b).

#### Distribution.

ARGENTINA. BOLIVIA. BRAZIL: Acre, Amazonas, Espírito Santo, Mato Grosso, Rio de Janeiro, São Paulo. COLOMBIA: Valle del Cauca. PARAGUAY: Alto Paraná, Caaguazú, Itapúa. SURINAME.

#### References.

Fabricius 1775, Olivier 1789, Thunberg 1814, Eschscholtz 1818, 1822, Dejean 1833, 1836b, Burmeister 1847, Harold 1869b, Bates 1888, Luederwaldt 1915, Arrow 1937b, Blackwelder 1944, Robinson 1947, Lima 1953, Zimsen 1964, Endrődi 1966, 1967c, 1973b, 1985a, Restrepo et al. 2003, Gasca-Álvarez and Amat-García 2010, Krajcik 2005, 2012, Ratcliffe et al. 2013, Ratcliffe 2015.

#### Remarks.

*Stenocrates laborator* was reported from Mexico and Guatemala (Bates 1888, Arrow 1937b, Blackwelder 1944, Endrődi 1966, 1985a). These records are likely based on misidentifications, and *S. laborator* is a South American species (Ratcliffe et al. 2013).

### Stenocrates laceyi

Taxon classificationAnimaliaColeopteraScarabaeidae

Ratcliffe, 1978

Stenocrates laceyi Ratcliffe, 1978: 493–494 [original combination].

#### Types.

Holotype ♂ at INPA (Ratcliffe 1978).

#### Distribution.

BRAZIL: Amazonas.

#### References.

Endrődi 1985a, Krajcik 2005, 2012, Ratcliffe 1978, 2015.

### Stenocrates lachaumei

Taxon classificationAnimaliaColeopteraScarabaeidae

Dechambre, 1985

Stenocrates lachaumei Dechambre, 1985: 143–144 [original combination].

#### Types.

Holotype ♂ at MNHN (Dechambre 1985).

#### Distribution.

BOLIVIA: Cochabamba, La Paz.

#### References.

Dechambre 1985, Ratcliffe and Morón 1997, Krajcik 2005, 2012, Ratcliffe et al. 2013, Ratcliffe 2015.

#### Remarks.

A specimen of *S. lachaumei* was reported from Veracruz, Mexico (Ratcliffe and Morón 1997, Ratcliffe et al. 2013). However, these data are considered either erroneous or the specimen was inadvertently transported there from South America (Ratcliffe et al. 2013).

### Stenocrates laevicollis

Taxon classificationAnimaliaColeopteraScarabaeidae

Kirsch, 1870

Stenocrates laevicollis Kirsch, 1870: 357–358 [original combination, pages incorrectly numbered].

#### Types.

Lectotype ♀ at MTD (Endrődi 1966).

#### Distribution.

BELIZE: Stann Creek. COLOMBIA: Antioquia, Boyacá, Capital District, Cundinamarca, Meta, Santander, Tolima, Valle del Cauca. COSTA RICA: Alajuela, Cartago, Guanacaste, Heredia, Limón, Puntarenas. ECUADOR: Morona Santiago. GUATEMALA: Alta Verapaz, Izabal. HONDURAS: Gracias a Dios, El Paraíso, Olancho. MEXICO: Chiapas, Hidalgo, Veracruz. NICARAGUA: Granada, Jinotega, RAA Norte, Río San Juan. PANAMA: Chiriquí, Panamá.

#### References.

Kirsch 1870, Arrow 1937b, Blackwelder 1944, Dechambre 1985, Endrődi 1966, 1985a, Thomas 1993, Ratcliffe and Morón 1997, Maes et al. 1997, Ratcliffe 2002a, 2003, Restrepo et al. 2003, Ratcliffe and Cave 2006, Gasca-Álvarez and Amat-García 2010, Krajcik 2005, 2012, Ratcliffe et al. 2013, García-López et al. 2013, López-García et al. 2015, Ratcliffe 2015.

### Stenocrates latus

Taxon classificationAnimaliaColeopteraScarabaeidae

Dechambre, 1979

Stenocrates latus Dechambre, 1979c: 63 [original combination].

#### Types.

Holotype ♂ at MNHN (Dechambre 1979c).

#### Distribution.

BRAZIL: Amazonas. COLOMBIA: Amazonas. ECUADOR.

#### References.

Dechambre 1979c, Krajcik 2005, 2012, Endrődi 1985a, Ratcliffe 2015.

### Stenocrates lecourti

Taxon classificationAnimaliaColeopteraScarabaeidae

Dechambre, 2006

Stenocrates lecourti Dechambre, 2006a: [original combination].

#### Types.

Holotype ♂ at RPDC (Dechambre 2006a).

#### Distribution.

PANAMA.

#### References.

Dechambre 2006a, Krajcik 2012, Ratcliffe 2015.

### Stenocrates lichyi

Taxon classificationAnimaliaColeopteraScarabaeidae

Dechambre, 1979

Stenocrates lichyi Dechambre, 1979c: 63–64 [original combination].

#### Types.

Holotype ♂ at MNHN (Dechambre 1979c).

#### Distribution.

BRAZIL: Amazonas. VENEZUELA: Amazonas, Guárico.

#### References.

Dechambre 1979c, Endrődi 1985a, Krajcik 2005, 2012, Ratcliffe 2015.

### Stenocrates ligneus

Taxon classificationAnimaliaColeopteraScarabaeidae

Arrow, 1911

Stenocrates ligneus Arrow, 1911: 168 [original combination].

#### Types.

Type at BMNH (Endrődi 1966).

#### Distribution.

BRAZIL: Amazonas, Pará. COLOMBIA. PARAGUAY.

#### References.

Arrow 1911, 1937b, Blackwelder 1944, Endrődi 1966, 1985a, Andreazze and Fonseca 1998, Dechambre and Hardy 2004, Ulmen et al. 2010, Krajcik 2005, 2012, Ratcliffe 2015.

### Stenocrates mahunkai

Taxon classificationAnimaliaColeopteraScarabaeidae

Endrődi, 1973

Stenocrates mahunkai Endrődi, 1973a: 59–61 [original combination].

#### Types.

Holotype ♂ at HNHM (Endrődi 1973a).

#### Distribution.

BRAZIL: Amazonas. BOLIVIA: Beni. ECUADOR.

#### References.

Dechambre 1985, Andreazze and da Silva Motta 2002, Endrődi 1973a, 1985a, Krajcik 2005, 2012, Ratcliffe 2015.

### Stenocrates mimeomus

Taxon classificationAnimaliaColeopteraScarabaeidae

Ratcliffe, 2015

Stenocrates mimeomus Ratcliffe, 2015: 776–777 [original combination].

#### Types.

Holotype ♂ at USNM (Ratcliffe 2015).

#### Distribution.

PERU: Madre de Dios.

#### References.

Ratcliffe 2015.

### Stenocrates minutus

Taxon classificationAnimaliaColeopteraScarabaeidae

Endrődi, 1966

Stenocrates minutus Endrődi, 1966: 415, 432–433 [original combination].
**syn.** Stenocrates rabbanii Ratcliffe, 1977: 432–433 [original combination].
Stenocrates mahunkai Endrődi [synonymy by Endrődi 1985a: 176].
Stenocrates minutus Endrődi [synonymy by Ratcliffe 2015: 778]

#### Types.

Holotype ♂ of *S. minutus* at NHMB (Frey Collection) (Endrődi 1966). Holotype ♂ of *S. rabbanii* at INPA (Ratcliffe 1977).

#### Distribution.

BOLIVIA: Beni. BRAZIL. ECUADOR. PERU: Loreto.

#### References.

Endrődi 1966, 1973a, 1985a, Dechambre 1985, Krajcik 2005, 2012, Ratcliffe et al. 2015, Ratcliffe 1977, 2015.

### Stenocrates mollis

Taxon classificationAnimaliaColeopteraScarabaeidae

Endrődi, 1966

Stenocrates mollis Endrődi, 1966: 416, 433–434 [original combination].

#### Types.

Holotype ♂ at ZMHB (Endrődi 1966).

#### Distribution.

BRAZIL. FRENCH GUIANA: Cayenne.

#### References.

Endrődi 1966, 1967c, 1985a, Ponchel 2011, Krajcik 2005, 2012, Ratcliffe 2015, Dupuis 2017.

### Stenocrates nasutus

Taxon classificationAnimaliaColeopteraScarabaeidae

Dechambre, 1979

Stenocrates nasutus Dechambre, 1979c: 64 [original combination].

#### Types.

Holotype ♂ at MNHN (Dechambre 1979c).

#### Distribution.

FRENCH GUIANA. PERU: Amazonas.

#### References.

Dechambre 1979c, Endrődi 1985a, Ponchel 2011, Krajcik 2005, 2012, Ratcliffe et al. 2015, Ratcliffe 2015, Dupuis 2017.

### Stenocrates omissus

Taxon classificationAnimaliaColeopteraScarabaeidae

Endrődi, 1966

Stenocrates omissus Endrődi, 1966: 416, 434–435 [original combination].

#### Types.

Holotype ♂ at HNHM (Endrődi Collection) (Endrődi 1966).

#### Distribution.

BOLIVIA: Beni, La Paz. BRAZIL: Amazonas, Mato Grosso. COLOMBIA: Cundinamarca. ECUADOR. FRENCH GUIANA: Kourou. PERU: Junín, Madre de Dios. TRINDAD AND TOBAGO: Trinidad.

#### References.

Dechambre 1979a, 1985, Endrődi 1966, 1973b, 1985a, Andreazze and da Silva Motta 2002, Restrepo et al. 2003, Gasca-Álvarez and Amat-García 2010, Ponchel 2011, Krajcik 2005, 2012, Breeschoten et al. 2013, López-García et al. 2015, Ratcliffe 2015, Dupuis 2017.

### Stenocrates pereirai

Taxon classificationAnimaliaColeopteraScarabaeidae

Endrődi, 1969

Stenocrates pereirai Endrődi, 1969b: 38–39 [original combination].

#### Types.

Holotype ♂ at “Pereira Collection in Sao Paulo” (Endrődi 1969b). This is possibly referring to MZSP.

#### Distribution.

BRAZIL: Mato Grosso, Rondônia.

#### References.

Endrődi 1969b, 1985a, Krajcik 2005, 2012, Ratcliffe 2015.

### Stenocrates popei

Taxon classificationAnimaliaColeopteraScarabaeidae

Endrődi, 1971

Stenocrates popei Endrődi, 1971a: 179–181 [original combination].
**syn.** Stenocrates inpai Ratcliffe, 1978: 491 [original combination].
Stenocrates popei Endrődi [synonymy by Ratcliffe 2015: 777].

#### Types.

Holotype ♂ of *S. popei* at BMNH (Endrődi 1971a). Holotype ♂ of *S. inpai* at INPA (Ratcliffe 1978).

#### Distribution.

BRAZIL: Amapá, Amazonas. FRENCH GUIANA. GUYANA. PERU. SURINAME.

#### References.

Dechambre 1979a, Endrődi 1971a, 1985a, Moragues 2010, Ponchel 2011, Krajcik 2005, 2012, Ratcliffe 1978, 2015, Ratcliffe et al. 2015, Dupuis 2017.

### Stenocrates porioni

Taxon classificationAnimaliaColeopteraScarabaeidae

Dechambre, 1985

Stenocrates porioni Dechambre, 1985: 144 [original combination].

#### Types.

Holotype ♂ at MNHN (Dechambre 1985).

#### Distribution.

ARGENTINA: Salta. BOLIVIA: La Paz.

#### References.

Dechambre 1985, Krajcik 2005, 2012, Ratcliffe 2015.

### Stenocrates pseudoligneus

Taxon classificationAnimaliaColeopteraScarabaeidae

Dechambre & Hardy, 2004

Stenocrates pseudoligneus Dechambre & Hardy, 2004: 213 [original combination].

#### Types.

Holotype ♂ at CMNC (Dechambre and Hardy 2004).

#### Distribution.

BOLIVIA: Beni (Dechambre and Hardy 2004).

#### References.

Dechambre and Hardy 2004, Krajcik 2012, Ratcliffe 2015.

### Stenocrates rionegroensis

Taxon classificationAnimaliaColeopteraScarabaeidae

Ratcliffe, 1978

Stenocrates rionegroensis Ratcliffe, 1978: 489–490 [original combination].

#### Types.

Holotype ♂ at INPA (Ratcliffe 1978).

#### Distribution.

BRAZIL: Amazonas.

#### References.

Endrődi 1985a, Dechambre and Hardy 2004, Krajcik 2005, 2012, Ratcliffe 1978, 2015.

### Stenocrates rufipennis

Taxon classificationAnimaliaColeopteraScarabaeidae

(Fabricius, 1801)

Melolontha rufipennis Fabricius, 1801: 167 [original combination].
Stenocrates rufipennis (Fabricius) [new combination by Burmeister 1847: 86].
**syn.** Stenocrates saucius Burmeister, 1847: 85–86 [original combination].
Stenocrates rufipennis (Fabricius) [synonymy by Arrow 1937b: 19].

#### Types.

Lectotype ♀ of *M. rufipennis* deposited at ZMUK, now housed at ZMUC (Endrődi 1966). Endrődi (1966) did not find the type material of *S. saucius.*

#### Distribution.

ARGENTINA: Córdoba. BRAZIL: Amazonas, Pará. COLOMBIA: Putumayo. ECUADOR: Guayas. FRENCH GUIANA: Cayenne. GUYANA.

#### References.

Fabricius 1801, Burmeister 1847, Harold 1869b, Arrow 1937b, Blackwelder 1944, Zimsen 1964, Dechambre 1979c, Endrődi 1966, 1985a, Andreazze and da Silva Motta 2002, Restrepo et al. 2003, Gasca-Álvarez and Amat-García 2010, Krajcik 2005, 2012, Ratcliffe 1977, 2015, Dupuis 2017.

### Stenocrates rugulosus

Taxon classificationAnimaliaColeopteraScarabaeidae

Endrődi, 1966

Stenocrates rugulosus Endrődi, 1966: 416, 436–437 [original combination].

#### Types.

Holotype ♂ at ZMHB (Endrődi 1966).

#### Distribution.

VENEZUELA: Capital District, Carabobo.

#### References.

Endrődi 1966, 1985a, Dechambre 1985, Krajcik 2005, 2012, Ratcliffe 2015.

### Stenocrates seag

Taxon classificationAnimaliaColeopteraScarabaeidae

Dupuis, 2017

Stenocrates seag Dupuis, 2017: 55–58 [original combination].

#### Types.

Holotype ♂ at MNHN (Dupuis 2017).

#### Distribution.

FRENCH GUIANA.

#### References.

Dupuis 2017.

### Stenocrates serendipitus

Taxon classificationAnimaliaColeopteraScarabaeidae

Ratcliffe, 2015

Stenocrates serendipitus Ratcliffe, 2015: 774–775 [original combination].

#### Types.

Holotype ♂ at UNSM (Ratcliffe 2015).

#### Distribution.

PERU: Loreto.

#### References.

Ratcliffe 2015.

### Stenocrates spinosus

Taxon classificationAnimaliaColeopteraScarabaeidae

Ponchel a&nd Dechambre, 2003

Stenocrates spinosus Ponchel & Dechambre, 2003: 268–270 [original combination].

#### Types.

Holotype ♂ at MNHN (Ponchel and Dechambre 2003).

#### Distribution.

BRAZIL. FRENCH GUIANA.

#### References.

Ponchel and Dechambre 2003, Ponchel 2011, Krajcik 2005, 2012, Ratcliffe 2015, Dupuis 2017.

### Stenocrates varzeaensis

Taxon classificationAnimaliaColeopteraScarabaeidae

Ratcliffe, 1978

Stenocrates varzeaensis Ratcliffe, 1978: 490–491 [original combination].

#### Types.

Holotype ♂ at INPA (Ratcliffe 1978).

#### Distribution.

BRAZIL: Amazonas.

#### References.

Dechambre 1979c, Endrődi 1985a, Krajcik 2005, 2012, Ratcliffe 1978, 2015.

### Genus Surutu

Taxon classificationAnimaliaColeopteraScarabaeidae

Martínez, 1955

Surutu Martínez, 1955: 242–244 [original usage].

#### Type species.

*Surutu dytiscoides* Martínez, 1955, by monotypy.

#### Keys.

Endrődi 1966, 1975a, 1985a, Ratcliffe 1981, Jameson et al. 2002.

#### Valid taxa.

5 species.

### Surutu dytiscoides

Taxon classificationAnimaliaColeopteraScarabaeidae

Martínez, 1955

Surutu dytiscoides Martínez, 1955: 245–249 [original combination].

#### Types.

Holotype ♂ at MACN (Antonio Martínez Collection) (Martínez 1955).

#### Distribution.

BOLIVIA: Cochabamba, Santa Cruz. COLOMBIA: Amazonas.

#### References.

Martínez 1955, 1956, Ratcliffe 1981, Endrődi 1966, 1975a, 1985a, Krajcik 2005, 2012, Otavo et al. 2013.

### Surutu fenni

Taxon classificationAnimaliaColeopteraScarabaeidae

Ratcliffe, 1981

Surutu fenni Ratcliffe, 1981: 107–111 [original combination].

#### Types.

Holotype ♂ at INPA (Ratcliffe 1981).

#### Distribution.

BRAZIL: Amazonas.

#### References.

Ratcliffe 1981, Andreazze 2001, Krajcik 2005, 2012.

### Surutu hesperius

Taxon classificationAnimaliaColeopteraScarabaeidae

Ratcliffe, 1981

Surutu hesperius Ratcliffe, 1981: 107, 111 [original combination].

#### Types.

Holotype ♂ at INPA (Ratcliffe 1981).

#### Distribution.

BRAZIL: Amazonas.

#### References.

Ratcliffe 1981, Krajcik 2005, 2012.

### Surutu schulzei

Taxon classificationAnimaliaColeopteraScarabaeidae

Endrődi, 1975

Surutu schulzei Endrődi, 1975a: 155, 156–157 [original combination].

#### Types.

Holotype ♂ at HNHM (Endrődi Collection) (Endrődi 1975a).

#### Distribution.

BRAZIL: Mato Grosso.

#### References.

Ratcliffe 1981, Endrődi 1975a, 1985a, Krajcik 2005, 2012.

### Surutu seabrai

Taxon classificationAnimaliaColeopteraScarabaeidae

D’Andretta & Martínez, 1956

Surutu seabrai D’Andretta & Martínez, 1956: 185–195 [original combination].

#### Types.

Holotype ♂ at MACN (Antonio Martínez Collection) (Martínez 1956).

#### Distribution.

BRAZIL: Amazonas, Pará.

#### References.

D’Andretta and Martínez 1956, Endrődi 1975a, 1985a, Ratcliffe 1981, Andreazze 2001, Krajcik 2005, 2012, Breeschoten et al. 2013.

## References

[B1] Abdullah M Roohi RA (1969) The cockchafers and dung-rollers of Pakistan of the Desmonycinae, Euchirinae and Rutelinae (Peltonotini, Parastasiini and Adorrhinyptiini) along with the description of five new species of *Anomala* (Coleoptera: Scarabaeidae). Pakistan Journal of Science and Industrial Research 12: 121–126.

[B2] Abraca G Quesada M (1997) Especies del complejo de jobotos (*Phyllophaga* spp., *Anomala* spp. y *Cyclocephala* spp.) asociadas a cultivos, en el valle Central y Pacífico seco de Costa Rica. Agronomía Mesoamericana 8: 44–53.

[B3] Abrahão J Amante E (1970) Fungos e insetos causadores de tombamento de mudas de algodoeiro no ano agrícola 1969–70. O Biológico 36: 24–25.

[B4] Achinelly MF Camino NB (2008) A new Nematoda (Thelastomatidae) parasite of Coleoptera larvae from Argentina. Helminthologia 45: 86–88. https://doi.org/10.2478/s11687-008-0016-1

[B5] Adams JA (1949) *Cyclocephala borealis* as a turf pest associated with the Japanese beetle in New York. Journal of Economic Entomology 42: 626–628. https://doi.org/10.1093/jee/42.4.626

[B6] Aguilera E Ramo C Busto B (1993) Food habits of the scarlet and white ibis in the Orinoco plains. The Condor 95: 739–741. https://doi.org/10.2307/1369623

[B7] Aguirre A Guevara R Dirzo R (2011) Effects of forest fragmentation on assemblages of pollinators and floral visitors to male- and female-phase inflorescences of *Astrocaryum mexicanum* (Arecaceae) in a Mexican rainforest. Journal of Tropical Ecology 27: 25–33. https://doi.org/10.1017/S0266467410000556

[B8] Alberio C Izquierdo NG Aguirrezábal LAN (2015) Sunflower crop physiology and agronomy. In: Martínez-Force E, Dunford NT, Salas JJ (Eds) Sunflower: chemistry, production, processing, and utilization. AOCS Press, Urbana, 53–91. https://doi.org/10.1016/B978-1-893997-94-3.50009-X

[B9] Albuquerque LSC de Souza TB de Maia ACD Iannuzzi L (2014) New biological and immature morphological records of the masked chafer, *Cyclocephala paraguayensis* Journal of Insect Science 14(101): 1–11. https://doi.org/10.1673/031.014.101 10.1093/jis/14.1.101 PMC4212850 25201356

[B10] Albuquerque LSC de Grossi PC Iannuzzi L (2016) Flight patterns and sex ratio of beetles of the subfamily Dynastinae (Coleoptera, Melolonthidae). Revista Brasileira de Entomologia 60: 248–254. https://doi.org/10.1016/j.rbe.2016.03.002

[B11] Alcázar-Ruiz JA Morón-Rios A Morón MA (2003) Fauna de Coleoptera Melolonthidae de Villa las Rosas, Chiapas, México. Acta Zoológica Mexicana (n. s.) 88: 59–86.

[B12] Allred DM Beck DE (1965) A list of Scarabaeidae beetles of the Nevada test site. The Great Basin Naturalist 25: 77–79.

[B13] Alvarado L (1980) Sistemática y bionomía de los estados inmaduros de coleópteros Scarabaeidae que habitan en el suelo. PhD thesis, La Plata, Argentina: Facultad de Ciencias Naturales y Museo-UNLP.

[B14] An R Grewal PS (2007) Differences in the virulence of *Heterorhabditis bacteriophora* and *Steinernema scarabaei* to three white grub species: the relative contribution of the nematodes and their symbiotic bacteria. Biological Control 43: 310–316. https://doi.org/10.1016/j.biocontrol.2007.07.004

[B15] An R Grewal PS (2016) Comparative analysis of *Xenorhabdus koppenhoeferi* gene expression during symbiotic persistence in the host nematode. PLOS One: DOI: 10.1371/journal.pone.0145739. 10.1371/journal.pone.0145739 PMC4706420 26745883

[B16] Anderson BA Klein MG Reding ME Moyseenko JJ (2015) Common white grubs of northeast Ohio nurseries. The Horticultural Insects Laboratory USDA-ARS, Wooster, 11 pp.

[B17] Anderson WG Baxendale FP Eickhoff TE Heng-Moss TM (2004) Application timing of Merit for control of billbugs and white grubs, 2003. Arthropod Management Tests 29(1): G12. https://doi.org/10.1093/amt/29.1.G12

[B18] Anderson WH (1936) A comparative study of the labium of coleopterous larvae. Smithsonian Miscellaneous Collections 95 (13): 1–29. [8 pls]

[B19] Andreazze R (2001) Dinastineos (Coleoptera, Scarabaeidae, Dynastinae) do Parque Naçional do Jaú, Amazonas Brasil. Acta Amazonica 31: 431–435. https://doi.org/10.1590/1809-43922001313435

[B20] Andreazze R Fonseca CRV (1998) Dinastínos (Coleoptera, Scarabaeoidea, Melolonthidae) em uma área de terra firme na Amazônia central, Brasil. Acta Amazonica 28: 59–66. https://doi.org/10.1590/1809-43921998281066

[B21] Andreazze R da Silva Motta C (2002) Besouros dinastineos (Coleoptera, Scarabaeidae, Dynastinae) de Querari, Municipio de São Gabriel da Cachoeira, Estado do Amazonas, Brasil. Acta Amazonica 32: 725–727. https://doi.org/10.1590/1809-43922002324727

[B22] Anonymous (1879) Siebenundsechzigster Jahresbericht des Steiermärkisch-Landschaftlichen Joanneums zu Graz über das Jahr 1878. Leykam-Josefthal, Graz, 1–40.

[B23] Anonymous (1881) Alphabetisches Register. Entomologische Zeitung 42: 505–512.

[B24] Anonymous (1886) Repertorium der 8 Jahrgänge (von 1879–1886) der Stettiner entomologischen Zeitung. Entomologische Zeitung 47: 377–467.

[B25] Anonymous (1900) Alphabetisches Register. Entomologische Zeitung 61: 398–401.

[B26] Anonymous (1940) Repertorium der Jahrgänge 48–100 (1887–1939). 1. Alphabetisches Autoren- und Schriftenverzeichnis. Entomologische Zeitung 101: 3–585.

[B27] Anonymous (1953) USDA Cooperative Economic Insect Report 3(41): 725.

[B28] Anonymous (1956) USDA Cooperative Economic Insect Report 6: 1079.

[B29] Anonymous (1971) A scarab (*Dyscinetus morator*)-Florida. USDA Cooperative Economic Insect Report 21: 25.

[B30] Anonymous (1980) USDA Cooperative Plant Pest Report 5: 66.

[B31] Anonymous (1993) Relevamiento de los insectos del suelo en cultivos de papa del sudeste bonarerense. Boletín Técnico-Estación Experimental Regional Agropecuaria Balcare 118: 5–18.

[B32] Ansari MA Tirry L Moens M (2004) Interaction between *Metarhizium anisopliae* CLO 53 and entomopathogenic nematodes for the control of *Hoplia philanthus* Biological Control 31: 172–180. https://doi.org/10.1016/j.biocontrol.2004.04.002

[B33] Apolinar Maria H (1946) Miscelanea entomologica. Revista de la Academia Colombiana de Ciencias Exactas, Fiscas y Naturales 5: 552–553.

[B34] App BA Kerr SH (1969) Harmful insects. In: Hanson AA, Juska FV (Eds) Turfgrass science. Agronomy Monograph 14. American Society of Agronomy, Madison, 336–357.

[B35] Aragón-García A Morón MA (2000) Los Coleopteros Melolonthidae asociados a la rizofera de la caña de azucar en Chietla, Puebla, Mexico. Folia Entomológica Mexicana 108: 79–94.

[B36] Aragón-García A Morón MA Damián-Huato MA López-Olguín JF Pinson-Rincón EP Pérez-Quintanilla JN (2012) Fauna de Coleoptera Lamellicornia de la zona cañera del ingenio de Atencingo, Puebla, México. Acta Zoológica Mexicana (n. s.) 28: 161–171.

[B37] Aragón A Morón MA Tapia-Rojas AM Rojas-García R (2001) Fauna de Coleoptera Melolonthidae en el rancho “La Joya”, Atlixco, Puebla, México. Acta Zoológica Mexicana (n. s.) 83: 143–164.

[B38] Arcangeli G (1908) Studi sulla *Victoria regia* Lindl. Atti della Società Toscana di Scienze Naturali Residente in Pisa 24: 59–78.

[B39] Arnaud PH (1978) A host-parasite catalog of North American Tachinidae (Diptera). United States Department of Agriculture Miscellaneous Publication 1319: 1–860.

[B40] Arnett Jr RH (1968) The beetles of the United States: A manual for identification. The American Entomological Institute, Ann Arbor, 1112 pp.

[B41] Arrow GJ (1900) On pleurostict lamellicorns from Grenada and St. Vincent (West Indies). Transactions of the Entomological Society of London 1900: 175–182. https://doi.org/10.1111/j.1365-2311.1900.tb00999.x

[B42] Arrow GJ (1902) Notes and descriptions of some Dynastidae from tropical America, chiefly supplementary to the ‘Biologia Centrali-Americana’. The Annals and Magazine of Natural History 7(10): 137–147. https://doi.org/10.1080/00222930208678646

[B43] Arrow GJ (1903) Descriptions of a few new species of Coleoptera from Sapucay, Paraguay. Proceedings of the General Meetings for Scientific Business of the Zoological Society of London 1903(2): 255–258.

[B44] Arrow GJ (1908) Contributions to the classification of the coleopterous family Dynastidae Transactions of the Entomological Society of London 1908: 321–358. https://doi.org/10.1111/j.1365-2311.1908.tb02151.x

[B45] Arrow GJ (1910) On the lamellicorn beetles of the genus *Peltonotus* with descriptions of four new species. Annals and Magazine of Natural History (Series 8)5: 153–157.

[B46] Arrow GJ (1911) Notes on the coleopterous subfamily Dynastinae, with descriptions of new genera and species. The Annals and Magazine of Natural History 8 (8^th^ series): 151–176.

[B47] Arrow GJ (1913) Some new species of lamellicorn beetles from Brazil. The Annals and Magazine of Natural History 11(8^th^ series): 456–466. https://doi.org/10.1080/00222931308693338

[B48] Arrow GJ (1914) Some further notes on lamellicorn beetles of the subfamily Dynastinae. Annals and Magazine of Natural History (series 8) 14: 257–276, 360.

[B49] Arrow GJ (1931) Two new species of lamellicorn beetles belonging to the genus *Peltonotus*. Annals and Magazine of Natural History (series 8)48: 611–612.

[B50] Arrow G J (1937a) Systematic notes on beetles of the subfamily Dynastinae, with descriptions of a few new species in the British Museum collection (Coleoptera) Transactions of the Entomological Society of London 86: 35–58. https://doi.org/10.1111/j.1365-2311.1937.tb00246.x

[B51] Arrow GJ (1937b) Pars 156: Scarabaeidae: Dynastinae In: Schenkling S (Ed.) Coleopterorum Catalogus. Volumen XXI. Scarabaeidae III. W Junk, Berlin, 1–124.

[B52] Askew RR (1994) Insects of the Cayman Islands. In: Brunt MA, Davies JE (Eds) The Cayman Islands: Natural History and Biogeography. Kluwer Academic Publishers, Dordrecht, 333–354. https://doi.org/10.1007/978-94-011-0904-8_17

[B53] Audureau A (2001) Présence de *Chalepides barbatus* (F., 1787) à Sainte-Lucie, Petites Antilles (Col. Dynastidae). Bulletin de la Société Entomologique de France 106: 426.

[B54] Austin EP (1880) Supplement to the check list of the Coleoptera of America, north of Mexico. SE Cassino, Boston, 67 pp.

[B55] Bacigalupo J (1939) El *Dyscinetus gagates* Burm, huésped intermediario de la *Hymenolepis diminuta* (Rudolphi). La Semana Médica 1: 1318–1319.

[B56] Balslev H Henderson A (1987) A new *Ammandra* (Palmae) from Ecuador. Systematic Botany 12: 501–504. https://doi.org/10.2307/2418885

[B57] Balut FF (1970) “Bicho-bôlo” em cultura de arroz (*Oryza sativa* L.). O Biológico 36: 321–322.

[B58] Baly JS (1885) Class Insecta Order Coleoptera Tribe Phytophaga (Continued). Fam. Hispidae In: Godman FD, Salvin O (Eds) Biologia Centrali-Americana, Insecta, Coleoptera, Phytophaga (part.), volume 6, part 2. RH Porter, London, 1–72, pls. 1–3.

[B59] Barbosa Moreira MA Lira MA Talamini V Carvalho HWL Santos Alves MC Sobrinho EE Souza Oliveira AM (2009) Avaliação de genótipos de girassol quanto à infestação do besouro amarelo, *Ciclocephala melanocephala* no estado do Rio Grande do Norte. ANAIS-XVIII Reunião Nacional de Pesquisa do Girassol VI Simpósio Nacional sobre a Cultura do Girassol: 154–159.

[B60] Barden SA Held DW Graham LCF (2011) Lack of interactions between fire ant control products and white grubs (Coleoptera: Scarabaeidae) in turfgrass. Journal of Economic Entomology 104: 2009–2016. https://doi.org/10.1603/EC11131 10.1603/ec11131 22299364

[B61] Barrantes ME Castelo MK (2014) Host specificity in the host-seeking larva of the dipteran parasitoid *Mallophora ruficauda* and the influence of age on parasitism decisions. Bulletin of Entomological Research 104: 295–306. https://doi.org/10.1017/S0007485314000029 10.1017/S0007485314000029 24548616

[B62] Barrera A (1969) Coleoptera Lamellicornia en la Coleccion Nacional. Acta Zoológica Mexicana 9: 1–93.

[B63] Barrows EM Gordh G (1978) Sexual behavior in the Japanese beetle, *Popillia japonica*, and comparative notes on sexual behavior of other scarabs (Coleoptera: Scarabaeidae). Behavioral Biology 23: 341–354. https://doi.org/10.1016/S0091-6773(78)91365-2

[B64] Barry FV (1951) Control de gusanos blancos. Asociación de Técnicas Azucareros de Cuba, Memoria de la Conferencía Annual 25: 51–56.

[B65] Bates HW (1888) Pectinicornia and Lamellicornia, Family Dynastidae In: Salvin O, Godman FD (Eds) Biologia Centrali–Americana. Insecta, Coleoptera, volume 2, part 2. RH Porter, London, 296–342.

[B66] Bates HW (1891) Coleoptera. In: Whymper E (Ed.) Supplementary Appendix to Travels Amongst the Great Andes of the Equator. John Murray, London, 7–39.

[B67] Bauernfeind RJ (2001) Distribution of *Cyclocephala* spp. (Coleoptera: Scarabaeidae) in Kansas. Environmental Entomology 30: 899–902. https://doi.org/10.1603/0046-225X-30.5.899

[B68] Bauernfeind RJ Haynes KF Potter DA (1999) Responses of three *Cyclocephala* (Coleoptera: Scarabaeidae) species to hexane extracts of *Cyclocephala lurida* sex pheromone. Journal of the Kansas Entomological Society 72: 246–247.

[B69] Bawa KS Bullock SH Perry DR Coville RE Grayum MH (1985a) Reproductive biology of tropical lowland rainforest trees. II. Pollination systems. American Journal of Botany 72: 346–356. https://doi.org/10.1002/j.1537-2197.1985.tb05358.x

[B70] Bawa KS Perry DR Beach JH (1985b) Reproductive biology of tropical lowland rainforest trees. I. Sexual systems and incompatibility mechanisms. American Journal of Botany 72: 331–345. https://doi.org/10.1002/j.1537-2197.1985.tb05357.x

[B71] Baxendale FP Eickhoff TE Heng-Moss TM (2003) Control of white grubs with the entomopathogenic nematode, *Heterorhabditis zealandica*, 2003. Arthropod Management Tests 28(1): G9. https://doi.org/10.1093/amt/28.1.G9

[B72] Baxendale FP Baxendale RW Rochefort S (2005) Use of nemagreen and SDS502 for control of white grubs, 2004. Arthropod Management Tests 30(1): G7. https://doi.org/10.1093/amt/30.1.G7

[B73] Baxendale RW Baxendale FP Stamm MD Schaeffer SK (2010) Meridian for control of southern masked chafers, 2009. Arthropod Management Tests 35(1): G11. https://doi.org/10.4182/amt.2010.G11

[B74] Beach JH (1982) Beetle pollination of *Cyclanthus bipartitus* (Cyclanthaceae). American Journal of Botany 69: 1074–1081. https://doi.org/10.1002/j.1537-2197.1982.tb13352.x

[B75] Beach JH (1984) The reproductive biology of the peach or “pejibaye” palm (*Bactris gasipaes*) and a wild congener (*B. porschiana*) in the Atlantic lowlands of Costa Rica. Principes 28: 107–119.

[B76] Beard JB (1972) Turfgrass: science and culture. Prentice Hall, Upper Saddle River, 658 pp.

[B77] Beath DN (1998) Pollination Ecology of the Araceae International Aroid Society, Inc. http://www.aroid.org/pollination/beath/index.php [5 September 2015]

[B78] Beath DN (1999) Dynastine scarab beetle pollination in *Dieffenbachia longispatha* (Araceae) on Barro Colorado Island (Panama) compared with La Selva Biological Station (Costa Rica). Aroideana 22: 63–71.

[B79] Bechtel RC Hanks LM Rust RW (1983) Coleoptera of Sand Mountain and Blow Sand Mountains, Nevada. The Southwestern Naturalist 28: 473–478. https://doi.org/10.2307/3670837

[B80] Beehag G Kaapro J Manners A (2016) Pest management of turfgrass for sport and recreation. CSIRO, Clayton, 312 pp.

[B81] Behle RW Goett EJ (2016) Dosage response mortality of Japanese beetle, masked chafer, and June beetle (Coleoptera: Scarabaeidae) adults when exposed to experimental and commercially available granules containing *Metarhizium brunneum* Journal of Economic Entomology 109: 1109–1115. https://doi.org/10.1093/jee/tow080 10.1093/jee/tow080 27133581

[B82] Behle RW Richmond DS Jackson MA Dunlap CA (2015) Evaluation of *Metarhizium brunneum* F52 (Hypocreales: Clavicipitaceae) for control of Japanese beetle larvae in turfgrass. Journal of Economic Entomology 108: 1587–1595. https://doi.org/10.1093/jee/tov176 10.1093/jee/tov176 26470299

[B83] Bélair G Koppenhöfer AM Dionne J Simard L (2010) Current and potential use of pathogens in the management of turfgrass insects as affected by new pesticide regulations in North America. International Journal of Pest Management 56: 51–60. https://doi.org/10.1080/09670870903076012

[B84] Benderitter E (1922) Un Rutélide [Col.] nouveau du Venezuela. Bulletin de la Société Entomologique de France 1922: 147.

[B85] Benderitter E (1934) Description d’un *Peltonotus* nouveau [Col. Rutelidae]. Bulletin de la Société Entomologique de France 39: 255–256.

[B86] Bennett FD Zwolfer H (1968) Exploration for natural enemies of the water hyacinth in northern South America and Trinidad. Hyacinth Control Journal 7: 44–52.

[B87] Berg C (1881a) Insectos. In: Doering A, Berg C, Holmberg EL (Eds) Informe Oficial de la Comision Científica Agregada al Estado Mayor General de la Expedicion al Rio Negro (Patagonia). Realizada en los mese de Abril, Mayo y Junio de 1879, bajo las órdenes del General D. Julio A. Roca (con 16 Láminas). Entrega I.-Zoología. Ostwald y Martinez, Buenos Aires, 77–115.

[B88] Berg C (1881b) Entomologisches aus dem Indianergebiet der Pampa. Entomologische Zeitung 42: 36–72.

[B89] Bernal R Ervik F (1996) Floral biology and pollination of the dioecious palm *Phytelephas seemannii* in Colombia: an adaptation to staphylinid beetles. Biotropica 28: 682–696. https://doi.org/10.2307/2389054

[B90] Berón MP Favero M (2010) Monitoreo de la dieta de la gaviota de olrog (*Larus atlanticus*) en la laguna Mar Chiquita (Buenos Aires, Argentina) durante el período no reproductive. Ornitologia Neotropical 21: 215–224.

[B91] Berón CM Diaz BM (2005) Pathogenicity of hyphomycetous fungi against *Cyclocephala signaticollis* BioControl 50: 143–150. https://doi.org/10.1007/s10526-004-0586-x

[B92] Bertkau P (1873) Bericht über die Leistungen in der Naturgeschichte der Insekten während der Jahre 1871 und 1872. Archiv für Naturgeschichte 39(2): 221–412.

[B93] Bertkau P (1878) Bericht über die wissenschaftlichen Leistungen im Gebiete der Arthropoden während der Jahre 1877–78. Archiv für Naturgeschichte 44(2): 219–562.

[B94] Bertkau P (1886) Bericht über die wissenschaftlichen Leistungen im Gebiete der Entomologie während des Jahres 1885. Archiv für Naturgeschichte 52(2, 2): 1–328.

[B95] Bertkau P (1892) ericht über die wissenschaftlichen Leistungen im Gebiete der Entomologie während des Jahres 1891. Archiv für Naturgeschichte 58(2, 2): 1–341.

[B96] Bertkau P (1893) Bericht über die wissenschaftlichen Leistungen im Gebiete der Entomologie während des Jahres 1892. Archiv für Naturgeschichte 59(2, 2): 1–348.

[B97] Bhargava D (1999) Carabid beetle invasion of the ear in Oman. Wilderness and Environmental Medicine 10: 157–160. https://doi.org/10.1580/1080-6032(1999)010[0157:CBIOTE]2.3.CO;2 10.1580/1080-6032(1999)010[0157:cbiote]2.3.co;2 10560309

[B98] Bigger JH Blachard RA (1955) Ecology and control of soil insects attacking corn in Illinois. Journal of Economic Entomology 48: 255–260. https://doi.org/10.1093/jee/48.3.255

[B99] Bixby A Alm SR Power K Grewal P Swier SR (2007) Susceptibility of four species of turfgrass-infesting scarabs (Coleoptera: Scarabaeidae) to *Bacillus thuringiensis* Serovar *japonensis* strain Buibui. Journal of Economic Entomology 100: 1604–1610. https://doi.org/10.1093/jee/100.5.1604 10.1603/0022-0493(2007)100[1604:sofsot]2.0.co;2 17972638

[B100] Blackwelder RE (1939) Fourth supplement 1933 to 1938 (inclusive) to the Leng catalogue of Coleoptera of America, north of Mexico. John D Sherman Jr, Mount Vernon, 146 pp.

[B101] Blackwelder RE (1944) Checklist of coleopterous insects of Mexico, Central America, the West Indies, and South America, part 2. Bulletin of the United States National Museum 185: 189–341.

[B102] Blackwelder RE Blackwelder RM (1948) Fifth supplement 1939 to 1947 (inclusive) to the Leng catalogue of Coleoptera of America, north of Mexico. John D Sherman Jr, Mount Vernon, 87 pp.

[B103] Blanchard CÉ (1846) In: Bertrand P (Ed.)Voyage dan L’Amérique Méridionale: (Le Brésil, la République Orientale de L’Uruguay, la République Argentine, la Patagonie, la République du Chile, la République de Bolivia, la République du Pérou), exécuté pendant les années 1826, 1827, 1828, 1829, 1830, 1831, 1832, et 1833 par Alcide d’Orbigny, volume 6, part 2: Insects. Pitois-Levrault, Paris, 105–222.

[B104] Blanco-Montero CA Ward CR (1995) Mitigation effects of cytokinin plant growth regulator on turfgrass root-biomass loss by white grubs. Southwestern Entomologist 20: 11–15.

[B105] Blanco-Montero CA Hernandez G (1995) Mechanical control of white grubs (Coleoptera: Scarabaeidae) in turfgrass using aerators. Environmental Entomology 2: 243–245. https://doi.org/10.1093/ee/24.2.243

[B106] Blanco-Montero CA Hernandez G (2006) Prediction of masked chafer, *Cyclocephala pasadenae*, capture in light traps using a degree-day model. Journal of Insect Science 6(36): 1–6. https://doi.org/10.1673/031.006.3601

[B107] Bland JHB (1863) Descriptions of a few supposed new species of North American Coleoptera. Proceedings of the Entomological Society of Philadelphia 1: 353–356.

[B108] Blatchley WS (1910) An illustrated descriptive catalogue of the Coleoptera or beetles (exclusive of the Rhynchophora) known to occur in Indiana. With bibliography and descriptions of new species. The Nature Publishing Co., Indianapolis, 1386 pp. https://doi.org/10.5962/bhl.title.56580

[B109] Blatchley WS (1930) The Scarabaeidae of Florida. Florida Entomologist 14: 13–17. https://doi.org/10.2307/3492197

[B110] Bodkin GE (1919) Notes on the Coleoptera of British Guiana. The Entomologist’s Monthly Magazine 55: 210–219.

[B111] Bohart RM (1947) Sod webworms and other lawn pests in California. Hilgardia 17: 267–308.

[B112] Boiça Jr AL Bolonhezi AC Paccini Neto K (1984) Levantamento de insetos-pragas e seus inimigos naturais em girassol (*Helianthus annuus* L.), cultivado em primeira e segunda época, no município de Selvíria-MS. Anais da Sociedade Entomológica do Brasil 13: 189–196. https://doi.org/10.3733/hilg.v17n08p267

[B113] Bolívar y Pieltain C Jiménez-Asúa L Martínez A (1963) Notas sobre Dynastinae Neotropicales con especial referencia a especies Mexicanas (Col., Scarab.). Ciencia (Mexico) 22: 181–190.

[B114] Bolliger A Magid J Amado TJC Neto FS dos Santos Ribeiro M de F Calegari A Ralisch R de Neergaard A (2006) Taking stock of the Brazilian “zero-till revolution”: A review of landmark research and farmers’ practice. Advances in Agronomy 91: 47–64. https://doi.org/10.1016/S0065-2113(06)91002-5

[B115] Bosq JM (1936) Cópula de dos Scarabaeidae de distintos géneros (*Cyclocephala putrida* Burm. ♂ con *Ligyrus burmeisteri* Steinh. ♀) (Col., Scarabaeidae, Dynastini.). Revista Chilena de Historia Natural 40: 26–28.

[B116] Bosq JM (1945) El “escarabajo negro del trigo” puede ser dañino a la silvicultura. Almanaque del Ministerio de Agricultura para el año 1945. Ministerio de Agricultura, Buenos Aires, Argentina, 65–67.

[B117] Bouchard SS Bjorndal KA (2006) Nonadditive interactions between animal and plant diet items in an omnivorous freshwater turtle *Trachemys scripta* Comparative Biochemistry and Physiology, Part B 144: 77–85. https://doi.org/10.1016/j.cbpb.2006.01.008 10.1016/j.cbpb.2006.01.008 16503179

[B118] Boucias DG Cherry RH Anderson DL (1986) Incidence of *Bacillus popilliae* in *Ligyrus subtropicus* and *Cyclocephala parallela* (Coleoptera: Scarabaeidae) in Florida sugarcane fields. Environmental Entomology 15: 703–705. https://doi.org/10.1093/ee/15.3.703

[B119] Bousquet Y Bouchard P Davies AE Sikes DS (2013) Checklist of beetles (Coleoptera) of Canada and Alaska. Second Edition. ZooKeys 360: 1–44. https://doi.org/10.3897/zookeys.360.4742 10.3897/zookeys.360.4742 PMC3867111 24363590

[B120] Box HE (1925) Porto Rican cane-grubs and their natural enemies, with suggestions for the control of lamellicorn larvae by means of wasp-parasites (Scoliidae). The Journal of the Department of Agriculture of Porto Rico 9: 291–356.

[B121] Bran AM Londoño ME Pardo LC (2006) Morfología de estados inmaduros de tres especies de *Cyclocephala* (Coleoptera: Melolonthidae) con una clave para larvas de tercer estado en Colombia. Revista Corpoica-Ciencia y Tecnología Agropecuaria 7: 58–66. https://doi.org/10.21930/rcta.vol7_num2_art:71

[B122] Brandenburg RL Royals BM (1999) Surface applied insecticides for the control of white grubs, 1998. Arthropod Management Tests 24(1): G21. https://doi.org/10.1093/amt/24.1.G21

[B123] Breeschoten T Clark DR Schilthuizen M (2013) Evolutionary patterns of asymmetric genitalia in the beetle tribe Cyclocephalini (Coleoptera: Scarabaeidae: Dynastinae). Contributions to Zoology 82: 95–106. https://doi.org/10.1603/EN13123

[B124] Bréthes J (1905) Insectos de Tucumán. Anales del Museo Nacional de Buenos Aires 3(4): 329–347.

[B125] Brill NL Osborne J Abney MR (2013) A spatial ecology study on the effects of field conditions and crop rotation on the incidence of *Plectris aliena* (Coleoptera: Scarabaeidae) Grub Damage to Sweetpotato Roots. Environmental Entomology 42: 1046–1051. 10.1603/EN13123 24331614

[B126] Brimley CS (1938) The insects of North Carolina, being a list of the insects of North Carolina and their close relatives. North Carolina Department of Agriculture Division of Entomology, Raleigh, 560 pp.

[B127] Britton EA (1970) Coleoptera (beetles). In: Mackerras IM (Ed.)The Insects of Australia: A textbook for students and research workers. Melbourne University Press, Canberra, 495–621.

[B128] Britton WE (1932) Lawns injured by *Ochrosidia*. Connecticut Agricultural Experiment Station Bulletin 338: 593.

[B129] Britton WE (1938) Additions to the check-list of the insects of Connecticut (first supplement to Bulletin No. 31). State of Connecticut, State Geological and Natural History Survey 60: 1–201.

[B130] Brower J van Zandt (1958) Experimental studies of mimicry in some North American butterflies: Part 1. The Monarch, *Danaus plexippus*, and Viceroy, *Limenitis archippus* Evolution 12: 32–47. https://doi.org/10.2307/2405902

[B131] Brown WJ (1930) Studies in the Scarabaeidae (IV)*. The Canadian Entomologist 62: 2–6. https://doi.org/10.4039/Ent622-1

[B132] Brown WJ (1934) New species Coleoptera V*. The Canadian Entomologist 66: 22–24. https://doi.org/10.4039/Ent6622-1

[B133] Bruner SC Scaramuzza LC Otero AR (1975) Catalogo de los insectos que atacan a las plantas economicas de Cuba. Segunda edición revisada y aumentada. Academia de Ciencias de Cuba Instituto de Zoologia, Havana, 400 pp.

[B134] Buchanan LL (1927) Notes on some light-attracted beetles from Louisiana (Coleop.). Entomological News 38: 165–70.

[B135] Buckingham GR Bennett CA (1989) *Dyscinetus morator* (Fab.) (Coleoptera: Scarabaeidae) adults attack waterhyacinth, *Eichhornia crassipes* (Pontederiaceae). The Coleopterists Bulletin 43: 27–33.

[B136] Bueno Jr R Stone JD Hinojos J (1988) The vertical migration of white grubs after peak adult flight in west Texas. Southwestern Entomologist 13: 1–9.

[B137] Bulla Jr LA Faust RM Andrews R Goodman N (1985) Insecticial bacilli. In: Dubnau DA (Ed.) The molecular biology of the bacilli, volume 2. Academic Press, New York, 185–209. https://doi.org/10.1016/B978-0-12-222702-8.50013-5

[B138] Bullock SH (1981) Notes on the phenology of inflorescences and pollination of some rain forest palms in Costa Rica. Principes 25: 101–105.

[B139] Burgeon L (1947) Catalogues raisonnés de la faune entomologique du Congo Belge. Coléoptères. Dynastinae, Valginae, Melolonthinae p. p. Annales du Musée du Congo Belge, Zoologie, Série III (II), Tome V, Fascicule 4: 277–340.

[B140] Burmeister H (1847) Handbuch der Entomologie, volume 5. TCF Enslin, Berlin, 584 pp.

[B141] Búrquez A Sarukhán KJ Pedroza AL (1987) Floral biology of a primary rainforest palm, *Astrocaryum mexicanum* Liebm. Botanical Journal of the Linnean Society 94: 407–419. https://doi.org/10.1111/j.1095-8339.1987.tb01058.x

[B142] Buss EA (2006) Flight activity and relative abundance of phytophagous scarabs (Coleoptera: Scarabaeoidea) from two locations in Florida. Florida Entomologist 89: 32–40. https://doi.org/10.1653/0015-4040(2006)89[32:FAARAO]2.0.CO;2

[B143] Buss EA Dale AG (2017) Insect pest management on turfgrass. University of Florida IFAS Extension ENY-300: 1–20.

[B144] Caceres VA Bigelow CA Richmond DS (2010) Aesthetic and economic impacts associated with four different cool-season lawn fertility and pesticide programs. HortTechnology 20: 418–426.

[B145] Caicedo AM Bellotti AC (2002) Entomofauna asociada al cultivo de espárragos en el department del Cauca, Colombia. Revista Colombiana de Entomología 28: 91–99.

[B146] Camino NB Reboredo GR (2005) A new *Oxyurida* (Thelastomatidae) from *Cyclocephala signaticollis* Burmeister (Coleoptera: Scarabaeidae) from Argentina. Journal of Parasitology 91: 890–892. https://doi.org/10.1645/GE-3447.1 10.1645/GE-3447.1 17089760

[B147] Camino NB Achinelly MF (2012) A new species of the genus *Cranifera* Kloss, 1960 (Thelastomatidae, Nematoda) parasitizing larvae of Scarabaeidae (Coleoptera) from Argentina. Estudos de Biologia 34: 57–59. https://doi.org/10.7213/estud.biol.6124

[B148] Camino-Lavín M Jiménez-Pérez A Castrejón-Gómez V Castrejón-Ayala F Figueroa-Brito R (1996) Comportamiento de una nueva trampa para escarabajos melolontidos, destructores de raices. Southwestern Entomologist 21: 325–330.

[B149] Cappaert DL Koppenhöfer AM (2003) *Steinernema scarabaei*, an entomopathogenic nematode for control of the European chafer. Biological Control 28: 379–386. https://doi.org/10.1016/S1049-9644(03)00118-X

[B150] Cappaert DL Smitley DR (2002) Parasitoids and pathogens of Japanese beetle (Coleoptera: Scarabaeidae) in southern Michigan. Environmental Entomology 31: 573–580. https://doi.org/10.1603/0046-225X-31.3.573

[B151] Carne PB (1956) *Cyclocephala signaticollis* Burmeister, an introduced pasture scarab (Coleoptera). The Proceedings of the Linnean Society of New South Wales 81: 217–221.

[B152] Carne PB (1957) A systematic revision of the Australian Dynastinae (Coleoptera: Scarabaeidae). CSIRO, Melbourne, 284 pp.

[B153] Carrillo-Ruiz H Morón MA (2003) Fauna de Coleoptera Scarabaeoidea de Cuetzalan del Progreso, Puebla, México. Acta Zoológica Mexicana (n. s.) 88: 87–121.

[B154] Carrillo-S JL Ortega-C A Gibson WW (1966) Lista de insectos en la coleccion entomologica del Instituto Nacional de Investigaciones Agricolas. Primer suplemento a la “Lista de Insectos de la colección entomológica de la Oficina de Estudios Especiales, S. A. G.”. Instituto Nacional de Investigaciones Agricola, S. A. G. Mexico, Folleto Miscelaneo 14: 1–133.

[B155] Carstens JD Eickhoff TE Baxendale FP (2002) Curative white grub control on a golf course fairway, 2001. Arthropod Management Tests 27(1): G13. https://doi.org/10.1093/amt/27.1.G13

[B156] Cartwright OL Chalumeau FE (1978) Bredin-Archbold-Smithsonian biological survey of Dominica. The superfamily Scarabaeoidea (Coleoptera). Smithsonian Contributions to Zoology 279: 1–32. https://doi.org/10.5479/si.00810282.279

[B157] Casey TL (1909) Studies in the Caraboidea and Lemellicornia. The Canadian Entomologist 41: 253–284. https://doi.org/10.4039/Ent41253-8

[B158] Casey TL (1915) A review of the American species of Rutelinae, Dynastinae and Cetoniinae Memoirs on the Coleoptera 11: 1–347.

[B159] Castelo MK Capurro AF (2000) Especifidad y denso-dependencia invera en parasitoides con oviposición fuera del hospedador: el caso de *Mallophora ruficauda* (Diptera: Asilidae) en la Pampa Argentina. Ecología Austral 10: 89–101. https://doi.org/10.1111/j.1442-9993.2009.02013.x

[B160] Castelo MK Corley JC (2010) Spatial density-dependent parasitism and specificity in the robber fly *Mallophora ruficauda* (Diptera: Asilidae). Austral Ecology 35: 72–81.

[B161] Castelo MK Crespo JE (2012) Incidence of non-immunological defenses of soil white grubs on parasitism success of *Mallophora ruficauda* larva (Diptera: Asilidae). Insects 3: 692–708. https://doi.org/10.3390/insects3030692 10.3390/insects3030692 PMC4553584 26466623

[B162] Castelo MK Lazzari CR (2004) Host-seeking behavior in larvae of the robber fly *Mallophora ruficauda* (Diptera: Asilidae). Journal of Insect Physiology 50: 331–336. https://doi.org/10.1016/j.jinsphys.2004.02.002 10.1016/j.jinsphys.2004.02.002 15081826

[B163] Castelo MK Ney-Nifle M Corley JC Bernstein C (2006) Oviposition height increases parasitisim success by the robber fly *Mallophora ruficauda* (Diptera: Asilidae). Behavioral Ecology and Sociobiology 61: 231–243. https://doi.org/10.1007/s00265-006-0254-5

[B164] Cavalcante TRM (2000) Polinização manual e natural da gravioleira (*Annona muricata* L.). MS thesis, Viçosa, Brazil: Universidad Federal de Viçosa.

[B165] Cavalcante TRM Naves RV Franceschinelli EV da Silva RP (2009) Polinização de frutos em araticum. Bragantia, Campinas 68: 13–21. https://doi.org/10.1590/S0006-87052009000100002

[B166] Chalumeau F (1982) Contribution a l’etude des Scarabaeoidea des Antilles (III). Nouveau Review d’Entomologie 12: 321–345.

[B167] Chalumeau F (1983) Les coléoptères scarabaeides des Petites Antilles (Guadeloupe à Martinique). Editions Lechevalier, Paris, 295 pp.

[B168] Chalumeau F Gruner L (1977) Scarabaeoidea des Antilles Françaises (Col.). 3 Partie: Dynastinae et Cetoniinae. Annales de la Société Entomologique de France 13: 579–612.

[B169] Chapin EA (1932) Revision of the pleurostict Scarabaeidae of Cuba and Ilse of Pines. II. Rutelinae, Dynastinae, and Cetoniinae Annals of the Entomological Society of America 25: 173–209. https://doi.org/10.1093/aesa/25.1.173

[B170] Chapin EA (1935a) New Cuban pleurostict Scarabaeidae. Memorias de la Sociedad Cubana de Historia Natural “Felipe Poey” 9: 67–75.

[B171] Chapin EA (1935b) New species of Scarabaeidae (Coleoptera) from Puerto Rico and the Virgin Islands. Journal of Agriculture of the University of Puerto Rico 19: 67–71.

[B172] Charlet LD Brewer GJ Franzmann BA (1997) Sunflower insects. In: Seiler GJ, Miller JF, Charlet LD, Meyer DW (Eds) Sunflower technology and production. Agronomy Monograph 35. American Society of Agronomy, Crop Science Society of America, Soil Science Society of America, Madison, 183–261.

[B173] Cherman MA Guedes JVC Morón MA Dal Prá E Bigolin M (2013) White grubs (Coleoptera, Melolonthidae) in the “Planalto Region”, Rio Grande do Sul state, Brazil: Key for identification, species richness, and distribution. Revista Brasileira de Entomologia 57: 271–278. https://doi.org/10.1590/S0085-56262013000300005

[B174] Cherry RH (1984a) Flooding to control the grub *Ligyrus subtropicus* (Coleoptera: Scarabaeidae) in Florida sugarcane. Journal of Economic Entomology 77: 254–257. https://doi.org/10.1093/jee/77.1.254

[B175] Cherry RH (1984b) Spatial distribution of white grubs (Coleoptera: Scarabaeidae) in Florida sugarcane. Journal of Economic Entomology 77: 1341–1343. https://doi.org/10.1093/jee/77.5.1341

[B176] Cherry RH (1985) Seasonal phenology of white grubs (Coleoptera: Scarabaeidae) in Florida sugarcane fields. Journal of Economic Entomology 78: 787–789. https://doi.org/10.1093/jee/78.4.787

[B177] Cherry RH (2012) White grubs in Florida sugarcane. EENY-664. University of Florida IFAS Extension, Gainesville, 3 pp.

[B178] Cherry RH Boucias DG (1989) Incidence of *Bacillus popilliae* in different life stages of Florida sugarcane grubs (Coleoptera: Scarabaeidae). Journal of Entomological Science 24: 526–529. https://doi.org/10.18474/0749-8004-24.4.526

[B179] Cherry RH Coale FJ (1994) Oviposition of the sugarcane grub, *Ligyrus subtropicus*, (Coleoptera: Scarabaeidae) in different soils. Journal of Agricultural Entomlogy 11: 345–348.

[B180] Cherry RH Coale FJ Porter PS (1990) Oviposition and survivorship of sugarcane grubs (Coleoptera: Scarabaeidae) at different soil moistures. Journal of Economic Entomology 83: 1355–1359. https://doi.org/10.1093/jee/83.4.1355

[B181] Cherry RH Klein MG (1997) Mortality induced by *Bacillus popilliae* in *Cyclocephala parallela* (Coleoptera: Scarabaeidae) held under simulated field temperatures. Florida Entomologist 80: 261–265. https://doi.org/10.2307/3495559

[B182] Chevrolat A (1844) In: Guérin-Méneville FE (Ed.)Iconographie du règne animal de G. Cuvier, ou representation d’après nature de l’une des espèces les plus remarquables, et souvent non encore figurées, de chaque genre d’animaux. Avec un texte descriptif mis au courant de la science. Ouvrage pouvant servir d’atlas a tous les traités de zoologie. Insectes, volume 1. J. B. Baillière, Paris, 576 pp.

[B183] Chevrolat A (1865) Coléoptères de l’Ile de Cuba. Notes, synonymies et descriptions d’espèces nouvelles. Sixième Mémoire. Famille des Lamellicornes (tribus des Mélonthides, Rutélides, Dynastides, et Cétonides, avec les indications des tribus, sous-tribus et groups du genera de Lacordaire). Annales de la Société Entomologique de France (series 4) 5: 21–35 (paginated separately as 131–145).

[B184] Chong J-H Hinson KR (2015) A comparison of trap types for assessing diversity of Scarabaeoidea on South Carolina golf courses. Journal of Economic Entomology 108: 2383–2396. https://doi.org/10.1093/jee/tov209 10.1093/jee/tov209 26453727

[B185] Christians NE Patton AJ Law QD (2016) Fundamentals of turfgrass management. Fifth edition. Wiley, Hoboken, 480 pp.

[B186] Cleare Jr LD (1925) Appendix V. Biological Division, 25^th^ February, 1924. British Guiana. Report on the Depratment of Science and Agriculture, for the year 1923: 47–51.

[B187] Cleare Jr LD (1930) The tannia beetle, *Ligyrus ebenus* DeG. Agricultural Journal of British Guiana 3: 11–23.

[B188] Cockerell TDA (1897) Biological notes on some Coleoptera from New Mexico. Journal of the New York Entomological Society 5: 149–150.

[B189] Cokendolpher JC (1993) Masked chafers, *Cyclocephala pasadenae* Casey (Coleoptera: Scarabaeidae), are poisonous to spiders. The Coleopterists Bulletin 47: 39–41.

[B190] Consolo VF Mucci V Salerno GL Berón CM (2010) Pathogenicity of bacterial isolates to *Cyclocephala signaticollis* (Coleoptera: Scarabaeidae). Biocontrol Science and Technology 20: 475–482. https://doi.org/10.1080/09583150903580537

[B191] Converse V (1998) Virulence of entomopathogenic nematodes to the western masked chafer *Cyclocephala hirta* (Coleoptera: Scarabaeidae). Journal of Economic Entomology 91: 428–432. https://doi.org/10.1093/jee/91.2.428 10.1093/jee/91.2.428 9589628

[B192] Costa FB Iannuzzi L (2010) Desenvolvimento biológico de *Cyclocephala paraguayensis* Arrow, 1904 (Coleoptera, Cyclocephalini) em laboratório. Conic e Coniti UFPE 28: 1–3.

[B193] Costa MS Silva RJ Paulian-Neto HF Pereira JB (2017) Beetle pollination and flowering rhythm of *Annona coriacea* Mart. (Annonaceae) in Brazilian cerrado: behavioral features of its principle pollinators. PLoS One 12 (2): e0171092. https://doi.org/10.1371/journal.pone.0171092 10.1371/journal.pone.0171092 PMC5289549 28152094

[B194] Costilla MA (1991) El gusano blanco *Dyscinetus rugifrons* (Burm) en la caña de azucar: Su importancia y forma manejo para su control. Avance Agroindustrial 12: 8–10.

[B195] Counts JW Hasiotis ST (2009) Neoichnological experiments with masked chafer beetles (Coleoptera: Scarabaeidae): implications for backfilled continental trace fossils. PALAIOS 24: 74–91. https://doi.org/10.2110/palo.2008.p08-026r

[B196] Couturier G Kahn F (1992) Notes on the insect fauna on two species of *Astrocaryum* (Palmae, Cocoeae, Bactridinae) in Peruvian Amazonia, with emphasis on potential pests of cultivated pests. Bulletin de l’Institut Française d’Etudes Andines 21: 715–725.

[B197] Coutinho GV Rodrigues SR da Cruz EC Abot AR (2011) Bionomic data and larval density of Scarabaeidae (Pleurosticti) in sugarcane in the central region of Mato Grosso do Sul, Brazil. Revista Brasileira de Entomologia 55: 389–395. https://doi.org/10.1590/S0085-56262011005000038

[B198] Cramer JM Meese ADJ Tuenissen PA (1975) A note on the pollination of nocturnally flowering species of *Nymphaea.* Acta Botanica Neerlandica 24: 489–490. https://doi.org/10.1111/j.1438-8677.1975.tb01039.x

[B199] Cranshaw W (2004) Garden insects of North America: the ultimate guide to backyard bugs. Princeton University Press, Princeton, 672 pp. https://doi.org/10.1515/9781400866786

[B200] Cranshaw W Ward CR (1996) Turfgrass insects in Colorado and northern New Mexico. Colorado State University Cooperative Extension, Fort Collins, 38 pp.

[B201] Crespo JE Castelo MK (2008) The ontogeny of host-seeking behavior in a parasitoid dipteran. Journal of Insect Physiology 54: 842–847. https://doi.org/10.1016/j.jinsphys.2008.03.002 10.1016/j.jinsphys.2008.03.002 18457846

[B202] Crespo JE Castelo MK (2009) Insights to host discrimination and host acceptance behavior in a parasitoid (Diptera: Asilidae): Implications for fitness. Journal of Insect Physiology 55: 1072–1078. https://doi.org/10.1016/j.jinsphys.2009.08.002 10.1016/j.jinsphys.2009.08.002 19682452

[B203] Crespo JE Castelo MK (2010) Life-history traits in a parasitoid dipteran species with free-living and obligate parastitic immature stages. Physiological Entomology 35: 160–167. https://doi.org/10.1111/j.1365-3032.2010.00727.x

[B204] Crespo JE Castelo MK (2012) Barometric pressure influences host-orientation behavior in the larva of a dipteran ectoparasitoid. Journal of Insect Physiology 58: 1562–1567. https://doi.org/10.1016/j.jinsphys.2012.09.010 10.1016/j.jinsphys.2012.09.010 23041375

[B205] Crespo JE Castelo MK (2013) Incidencia de las defensas del hospedador en el éxito de parasitismo de la larva parasitoide de *Mallophora ruficauda* (Diptera: Asilidae). Acta Zoológica Lilloana 57 (suplemento V. Reunión Argentina de Parasitoidólogos 2013): 104–106.

[B206] Crespo JE Lazzari CR Castelo MK (2011) Orientation mechanisms and sensory organs involved in host location dipteran parasitoid larva. Journal of Insect Physiology 57: 191–196. https://doi.org/10.1016/j.jinsphys.2010.11.010 10.1016/j.jinsphys.2010.11.010 21078328

[B207] Crespo JE Martínez GA Castelo MK (2015) Exposure to competitors influences parasitism decisions in ectoparasitoid fly larvae. Animal Behavior 100: 38–43. https://doi.org/10.1016/j.anbehav.2014.11.005

[B208] Croat TB (1997) A revision of Philodendron subgenus Philodendron (Araceae) for Mexico and Central America. Annals of the Missouri Botanical Garden 84: 311–704. https://doi.org/10.2307/2992022

[B209] Crocker RL (1992a) Factors affecting vertical and horizontal distribution of white grubs in experimental cages. (Texas Turfgrass Research 1991) Texas Agricultural Experiment Station Progress Report 4906: 76–77.

[B210] Crocker RL (1992b) Preliminary studies of white grub impact on turfgrass. (Texas Turfgrass Research 1991) Texas Agricultural Experiment Station Progress Report 4907: 78–82.

[B211] Crocker RL Cromroy HL Woodruff RE Nailon Jr WT Longnecker MT (1992) Incidence of *Caloglyphus phyllophagianus* (Acari: Acaridae) on adult *Phyllophaga* spp. and other Scarabaeidae (Coleoptera) in north central Texas. Annals of the Entomological Society of America 85: 462–468. https://doi.org/10.1093/aesa/85.4.462

[B212] Crocker RL Klein MG Wei X Nailon Jr WT (1999) Attraction of *Phyllophaga congrua*, *Phyllophaga crassissima*, *Phyllophaga crinita*, and *Cyclocephala lurida* (Coleoptera: Scarabaeidae) adults to selected volatile compounds. Southwestern Entomologist 24: 315–319.

[B213] Crotch GR (1873) Check list of the Coleoptera of America, north of Mexico. Naturalists’ Agency and The Salem Press F. W. Putnam & Co., Salem, 136 pp.

[B214] Crutchfield BA Potter DA (1995a) Damage relationships of Japanese Beetle and Southern Masked Chafer (Coleoptera: Scarabaeidae) grubs in cool-season turfgrasses. Journal of Economic Entomology 88: 1049–1056. https://doi.org/10.1093/jee/88.4.1049

[B215] Crutchfield BA Potter DA (1995b) Tolerance of cool-season turfgrasses to feeding by Japanese Beetle and South Masked Chafer (Coleoptera: Scarabaeidae) grubs. Journal of Economic Entomology 88: 1380–1387. https://doi.org/10.1093/jee/88.5.1380

[B216] Crutchfield BA Potter DA (1995c) Feeding by Japanese Beetle and Southern Masked Chafer grubs on lawn weeds. Crop Science 35: 1681–1684. https://doi.org/10.2135/cropsci1995.0011183X003500060028x

[B217] Crutchfield BA Potter DA Powell AJ (1994) Preferences of Japanese Beetle and Southern Masked Chafer (Coleoptera: Scarabaeidae) grubs among cool-season turfgrasses. Journal of Entomological Science 29: 398–406. https://doi.org/10.18474/0749-8004-29.3.398

[B218] Crutchfield BA Potter DA Powell AJ (1995) Irrigation and nitrogen fertilization effects on white grub injury to Kentucky bluegrass and tall fescue turf. Crop Science 35: 1122–1126.

[B219] D’Andretta MAV Martínez A (1956) Una nueva especie del género *Surutu* Martínez. Revista Brasileira de Entomologia 4: 185–194. https://doi.org/10.2135/cropsci1995.0011183X003500040034x

[B220] Daly T Buntin GD (2005) Effect of *Bacillus thuringiensis* transgenic corn for lepidopteran control on nontarget arthropods. Environmental Entomology 34: 1292–1301. https://doi.org/10.1093/ee/34.5.1292

[B221] Danell E (2010) Dokmai dogma: *Amorphophallus* and its edible beetles. http://dokmaidogma.wordpress.com/2010/05/27/amorphophallus-and-its-edible-beetles/ [Accessed February 1, 2016]

[B222] Daniel H (1945) Algunos aspectos de a lucha biológica. Los defensores del agriculor, insectos. Insectos devoradores de insectos? Revista de la Universidad de Antioquia 70: 339–359.

[B223] Dash J (1922) Troisième rapport de la Station Agronomique de la Guadeloupe. Juillet 1920 à Junin 1921, 17 pp.

[B224] Dash J (1934) Entomological Investigations. British Guiana. Adminstration Report of the Director of Agriculture for the Year 1933: 25–26.

[B225] Davis CC Endress PK Baum DA (2008) The evolution of floral gigantism. Current Opinion in Plant Biology 11: 49–57. https://doi.org/10.1016/j.pbi.2007.11.003 10.1016/j.pbi.2007.11.003 18207449

[B226] Dawson RW (1922) A synopsis of the Scarbaeidae of Nebraska. University Studies published by the University of Nebraska 22: 163–244.

[B227] Davis JJ (1916) A progress report on white grub investigations. Journal of Economic Entomology 9: 261–281. https://doi.org/10.1093/jee/9.2.261

[B228] Dechambre R-P (1979a) Missions entomologiques en Guyane et au Brésil [Coleoptera Dynastidae]. Revue Française d’Entomologie (Nouvelle Série) 1: 160–168.

[B229] Dechambre R-P (1979b) Une nouvelle espèce de *Cyclocephala* (Coleoptera Dynastidae). Nouvelle Revue d’Entomologie 9: 317–318.

[B230] Dechambre R-P (1979c) Cinq espèces nouvelles de *Stenocrates* [Col. Scarabaeoidea Dynastidae]. Revue Française d’Entomologie (Nouvelle-Series) 1: 61–64.

[B231] Dechambre R-P (1980) Six nouvelles espèces de *Cyclocephala* [Coleoptera, Dynastidae]. Revue Française d’Entomologie (Nouvelle-Series) 2: 42–49.

[B232] Dechambre R-P (1982) Deux nouvelles espèces de *Cyclocephala* [Coleoptera, Dynastidae]. Revue Française d’Entomologie (Nouvelle-Series) 4: 1–3.

[B233] Dechambre R-P (1985) Quatre nouvelles espèces de *Stenocrates* [Coleoptera, Dynastidae]. Revue Française d’Entomologie (Nouvelle-Series) 7: 142–144.

[B234] Dechambre R-P (1991a) Note synonymique sur *Surutoides mirabilis* Endrődi (Col., Dynastindae). Bulletin de la Société Entomologique de France 96: 282.

[B235] Dechambre R-P (1991b) Désignation de lectotypes de *Cyclocephala* décrits par Burmeister [Coleoptera, Dynastidae]. Revue Française de Entomologie 13: 123–124.

[B236] Dechambre R-P (1992) Description de Nouveaux Cyclocephalini et Agaocephalini In: Lachaume G (Ed.) Les Coléoptères du Monde, Vol. 14. Dynastidae Américains. Cyclocephalini – Agaocephalini – Pentodontini – Oryctini – Phileurini. Sciences Nat, Venette, 57–76.

[B237] Dechambre R-P (1995) Trois nouvelles especes de *Cyclocephala* (Coleoptera, Dynastidae). Bulletin de la Sociétè Sciences Nat 83: 12–13.

[B238] Dechambre R-P (1997) Révision des *Cyclocephala* du groupe *cribrata* Burmeister (Coleoptera, Dynastidae). Coléoptères 3: 13–27.

[B239] Dechambre R-P (1999) Vingt nouvelles espèces et une nouvelle sous-espèces de *Cyclocephala* Burmeister, 1847. Les Coléoptères du Monde 14, Supplement 1. Dynastidae américains. Hillside Books, Canterbury, 24 pp. + 1 pls.

[B240] Dechambre R-P (2000) *Ancognatha matilei*, nouvelle espèce de Cyclocephalini de Colombie (Coleoptera, Dynastidae). Revue Française d’Entomologie (Nouvelle-Series) 22: 183–184.

[B241] Dechambre R-P (2006a) Deux nouvelles espèces de *Stenocrates* (Coleoptera, Dynastidae). Coléoptères 12: 19–31.

[B242] Dechambre R-P (2006b) Une seconde espèce de *Ruteloryctes* Arrow, 1908 (Coleoptera, Dynastidae). Coléoptères 12: 53–55.

[B243] Dechambre R-P Duranton M (2005) Contribution à la connaissance des *Cyclocephala* de Guyane (Coleoptera, Dynastidae). Coléoptères 11: 67–76.

[B244] Dechambre R-P Endrődi S (1983) Une nouvelle espèce de *Cyclocephala* [Coleoptera, Dynastidae]. Revue Française d’Entomologie (Nouvelle-Series) 5: 83–84.

[B245] Dechambre R-P Endrődi S (1984) Quatre nouvelles espèces de *Cyclocephala* [Coleoptera, Dynastidae]. Revue Française d’Entomologie (Nouvelle-Series) 6: 168–172.

[B246] Dechambre R-P Hardy M (2004) Quatre nouvelles espèces de *Stenocrates* Burmeister, 1847 (Coleoptera, Dynastidae). Coléoptères 10: 209–214.

[B247] Dechambre R-P Ponchel Y (1999) Le genre *Homeomorphus* Burmeister, 1847 (Coleoptera, Dynastidae). Revue Française d’Entomologie (Nouvelle-Series) 21: 1–4.

[B248] De Coninck DIM De Schamphelaere KAC Jansen M De Messter L Janssen CR (2013) Interactive effects of a bacterial parasite and the insecticide carbaryl to life-history and physiology of two *Daphnia magna* clones differing in carbaryl sensitivity. Aquatic Toxicology 130: 149–159. https://doi.org/10.1016/j.aquatox.2013.01.008 10.1016/j.aquatox.2013.01.008 23411351

[B249] DeFoliart G (2002) Insect foods of North American Indigenous populations north of Mexico. In: DeFoliart G (Ed.) The human use of insects as a food resource: A bibliographic account in progress. Published electronically. http://labs.russell.wisc.edu/insectsasfood/the-human-use-of-insects-as-a-food-resource/

[B250] DeGeer C (1774) Memoires pour servir a l’histoire des insectes. Volume 4. Pierre Hesselberg, Stockholm, xii + 456 pp. + 19 pls.

[B251] Dejean PFMA (1821) Catalogue de la Collection de Coléoptères de M. le Baron Dejean. Crevot Libraire, Paris, 136 pp. https://doi.org/10.5962/bhl.title.11259

[B252] Dejean PFMA (1833–1836a) Catalogue des Coléoptères de la collection de M. le Comte Dejean. Méquignon-Marvis,Paris, 443 pp. [Livraisons 1–2 (1833) : 1–176; Livraison 3 (1834): 177–256; Livraison 4 (1835): 257–360; Livraison 5 (1836a): 361–443].

[B253] Dejean PFMA (1836b-1837) Catalogue des coléoptères de la collection de M. le comte Dejean. Troisième édition, revue, corrigée et augmentée. Méquignon-Marvis Père et Fils, Paris, xiv, 503 pp. [Livraisons 1–4 (1836b) : 1–384; Livraison 5 (1837): i–xiv, 385–503].

[B254] Delgado L (1991) Una especie nueva Mexicana de *Stenocrates* (Coleoptera: Melolonthidae; Dynastinae). Anales del Instituto de Biología de la Universidad Nacional Autónoma de México, Series Zoología 62: 103–108.

[B255] Delgado L (1992) Una especie nueva Mexicana de *Cyclocephala* (Coleoptera: Melolonthidae; Dynastinae). Anales del Instituto de Biología de la Universidad Nacional Autónoma de México, Series Zoología 63: 75–78.

[B256] Delgado L Castañeda H (1994) A new species and new records of *Cyclocephala* from Guatemala (Coleoptera: Melolonthidae). Journal of the New York Entomological Society 102: 456–459.

[B257] Deloya C Burgos A Blackaller J Lobo JM (1993) Los coleopteros lamelicornios de Cuernavaca, Morelos, México (Passalidae, Trogidae, Scarabaeidae y Melolonthidae). Boletin Sociedad Veracruzana de Zoología 3: 15–55.

[B258] Deloya C Morón MA (1994) Listados faunísticos de México. V. Coleópteros lamelicornios del distrito de Jojutla, Morelos, México (Melolonthidae, Scarabaeidae, Trogidae, y Passalidae). Instituto de Biología, Universidad Nacional Autónoma de México, México, Distrito Federal, 49 pp.

[B259] Deloya C Calvo-Gatica H García-Díaz OJ Rendón-Sosa M González-Hilario S Aguirre-León G (2014a) Familia Scarabaeidae Latreille, 1802. In: Deloya C, Melgar DC (Eds) Escarabajos del Estado de Guerrero (Coleoptera: Scarabaeoidea). S y G Editores, México, Distrito Federal, 69–116.

[B260] Deloya C Pacheco-Flores C Aguirre-León G Silva-Aparicio M (2014b) Escarabajos fitófagos de la Montaña Alta: vertiente externa. In: Deloya C, Melgar DC (Eds) Escarabajos del Estado de Guerrero (Coleoptera: Scarabaeoidea). S y G Editores, México, Distrito Federal, 133–140.

[B261] Deloya C Ponce Saavedra J Gasca Álvarez HJ Aguirre León G Zamora Vuelvas MC (2016) Familia Scarabaeidae Latreille, 1802. In: Deloya C, Ponce Saavedra J, Reyes Castillo P, Aguirre León G (Eds) Escarabajos del Estado de Michoacán (Coleoptera: Scarabaeoidea). Universidad Michoacana de San Nicolás de Hidalgo, Morelia, 93–142.

[B262] Del Valle EE Frizzo LS Lax P Bonora JS Palma L Desch NPB Pietrobón M Doucet ME (2017) Biological control of Diloboderus abderus (Coleoptera: Scarabaeidae) larvae using Steinernema rarum CUL (Nematoda: Steinernematidae) and Heterorhabditis bacteriophora SMC (Nematoda: Heterorhabditidae). Crop Protection 98: 184–190. https://doi.org/10.1016/j.cropro.2017.04.004

[B263] Demay M (1838) Revue Zoologique, par la Société Cuvierienne 1838: 23–24.

[B264] Dennis DS Knutson L (1988) Descriptions of pupae of South American robber flies (Diptera: Asilidae). Annals of the Entomological Society of America 81: 851–864. https://doi.org/10.1093/aesa/81.6.851

[B265] Díaz A Péfaur JE Durant P (1997) Ecology of South American páramos with emphasis on the fauna of the Venezuelan páramos. In: Wielgolaski FE (Ed.) Polar and Alpine Tundra. Ecosystems of the World 3. Elsevier, Amsterdam and New York, 263–310.

[B266] Díaz Mederos P Nájera Rincón MB Gutiérrez RL Domínguez OR Flores López HE Martínez Sifuentes JA (2006) Especies de gallina ciega (Coleoptera: Melolonthidae) y su asociación con factores agroclimáticos y de manejo del maíz en los altos de Jalisco, México. Fitosanidad 10: 209–215.

[B267] Dieringer G Delgado L (1994) Notes on the biology of *Cyclocephala jalapensis* (Coleoptera: Scarabaeidae): an endemic of eastern Mexico. Southwestern Entomologist 19: 309–311. https://doi.org/10.2307/2997167

[B268] Dieringer G Espinosa JE (1994) Reproductive ecology of *Magnolia schiedeana* (Magnoliaceae), a threatened cloud forest tree species in Veracruz, Mexico. Bulletin of the Torrey Botanical Club 121: 154–159.

[B269] Dieringer G Cabrera RL Lara M Loya L Reyes-Castillo P (1999) Beetles pollination and floral thermogenicity in *Magnolia tamaulipana* (Magnoliaceae). International Journal of Plant Sciences 160: 64–71. https://doi.org/10.1086/314099

[B270] Dieringer G Reyes-Castillo P Lara M Cabrera RL Loya L (1998) Endothermy and floral utilization of *Cyclocephala caelestis* (Coleoptera: Scarabaeoidae: Melolonthidae): a cloud forest endemic beetle. Acta Zoologica Mexicana 73: 145–153.

[B271] Diez-Rodríquez GI Hübner JK Corrêa Antunes LE Carús Guedes JV Nava DE (2015) Registro de *Cyclocephala flavipennis* Arrow, 1914 (Coleoptera: Melolonthidae) danficando plantas de mirtileiro no Brasil. Ciência Rural, Santa Maria 45: 189–191. https://doi.org/10.1590/0103-8478cr20140412

[B272] Dimmitt MA Ruibal R (1980) Exploitation of food resources by spadefoot toads (*Scaphiopus*). Copeia 4: 854–862. https://doi.org/10.2307/1444465

[B273] Dingman DW (2008) Geographical distribution of milky disease bacteria in the eastern United States based on phylogeny. Journal of Invertebrate Pathology 97: 171–181. https://doi.org/10.1016/j.jip.2007.09.002 10.1016/j.jip.2007.09.002 17959195

[B274] Dötterl S David A Boland W Silberbauer-Gottsberger I Gottsberger G (2012) Evidence for behavioral attractiveness of methoxylated aromatics in a dynastid scarab beetle-pollinated Araceae Journal of Chemical Ecology 38: 1539–1543. https://doi.org/10.1007/s10886-012-0210-y 10.1007/s10886-012-0210-y 23143663

[B275] Dougherty CT Knapp FW (1994) Oviposition and development of face flies in dung from cattle on herbage and supplemented herbage diets. Veterinary Parasitology 55: 115–127. https://doi.org/10.1016/0304-4017(94)90061-2 10.1016/0304-4017(94)90061-2 7886909

[B276] Dossey AT Morales-Ramos JA Guadalupe Rojas M (2016) Documented information for 1555 species of insects and spiders. In: Insects as sustainable food ingredients: production, processing and food applications. Elsevier Academic Press, San Diego, 311–376.

[B277] Drury D (1770) Illustration of natural history. B. White; London, UK. xxvii + 132 p. + 50 pls.

[B278] Drury D (1837) In: Westwood JO, Drury D (Eds) Illustrations of exotic entomology, containing upwards of six hundred and fifty figures and descriptions of foreign insects, interspersed with remarks and reflections on their nature and properties. Volume 1. Henry G. Bohn, London, sections paginated separately.

[B279] Dupuis F (1996) Description d’une nouvelle espèce de *Cyclocephala* Latreille, 1829, et mise au point sur les espèces du groupe *melanocephala* (Coleoptera, Dynastidae). Bulletin de la Société Entomologique de France 101: 257–260.

[B280] Dupuis F (1999) Nouveaux Cyclocephalini Neotropical (Coleoptera, Dynastidae). Revue Française d’Entomologie 21: 183–187.

[B281] Dupuis F (2006) Deux nouvelles espèces de Cyclocephalini (Coleoptera, Dynastidae). Coléoptères 12: 309–312.

[B282] Dupuis F (2008) Deux nouvelles *Cyclocephala* de Colombie (Coleoptera, Dynastidae). Coléoptères 14: 117–124.

[B283] Dupuis F (2009) *Cyclocephala rotundipenis*, nouvelle espèce de Colombie (Coleoptera, Dynastidae). Coléoptères 15: 29–32.

[B284] Dupuis F (2014) Trois nouveaux Cyclocephalini de la region andine (Coleoptera, Dynastidae). Coléoptères 20: 49–56.

[B285] Dupuis F (2017) Les *Stenocrates* Burmeister, 1847, de Guyane (Coleoptera, Dynastidae). Coléoptères 23: 49–60.

[B286] Dupuis F (2018) Espèces nouvelles ou méconnues de *Cyclocephala* Dejean, 1821 (Coleoptera, Dynastidae). Coléoptères 24: 1–12.

[B287] Dupuis F Dechambre R-P (1995) Mise au point sur les *Stenocrates* du groupe *cultor* [Coleoptera, Dynastidae]. Revue Française d’Entomologie 17: 59–61.

[B288] Dutky SR (1941) Susceptibility of ceratin scarabaeid larvae to infection by Type A milky disease. Journal of Economic Entomology 34: 215–216. https://doi.org/10.1093/jee/34.2.215

[B289] Dutky SR (1963) The milky disease. In: Steinhaus EA (Ed.) Insect pathology: an advanced treatise. Volume 2. Academic Press, New York, 75–115. https://doi.org/10.1016/B978-0-12-395603-3.50007-0

[B290] Dutrillaux B Dutrillaux A-M (2013) A South American origin of the genus *Dynastes* (Coleoptera: Scarabaeidae: Dynastinae) demonstrated by chromosomal analyses. Cytogenetic and Genome Research 141: 37–42. https://doi.org/10.1159/000351210 10.1159/000351210 23735513

[B291] Dutrillaux A-M Xie H Dutrillaux B (2007) High chromosomal polymorphism and heterozygosity in *Cyclocephala tridentata* from Guadeloupe: chromosome comparison with some other species of Dynastinae (Coleoptera: Scarabaeidae). Cytogenetic and Genome Research 119: 248–254. https://doi.org/10.1159/000112070 10.1159/000112070 18253038

[B292] Dutrillaux B Chalumeau F Dutrillaux A-M Giannoulis T Mamuris Z (2013) Séparation taxonomique en trois espèces au sein des populations de *Cyclocephala tridentata* Fabricius (Coleoptera: Scarabaeidae: Dynastinae), sur la base de critères génétoques, chromosomiques et géographiques. Annales de la Société Entomologique de France (n. s.) 49: 61–67.

[B293] Dutrillaux B Pluot-Sigwalt D Dutrillaux A-M (2014) Unbalanced sex ratio and triploidy in the genus *Cyclocephala* (Coleoptera: Scarabaeoidea: Dynastidae) in the Lesser Antilles: An example of parthenogenesis on islands? European Journal of Entomology 111(3): 1–7. https://doi.org/10.14411/eje.2014.048

[B294] Eickhoff TE Baxendale FP Heng-Moss TM (2005) Use of provaunt for control of white grubs, 2004. Arthropod Management Tests 30(1): G8.

[B295] Eickhoff TE Baxendale RW Wasem CM Baxendale FP (2006) Effect of application timing for control of white grubs using Allectus, 2005. Arthropod Management Tests 31(1): G21. https://doi.org/10.1093/amt/31.1.G21

[B296] Elizondo-Solís JM (2002) Inventario y fluctuación poblacional de insectos y arañas asociadas con Citrus sinensis en la región Huetar Norte de Costa Rica. Manejo Integrado de Plagas y Agroecología (Costa Rica) 64: 88–98.

[B297] Eliyahu D Nojima S Mori K Schal C (2009) Jail baits: how and why nymphs mimic adult females of the German cockroach, *Blattella germanica* Animal Behaviour 78: 1097–1105. https://doi.org/10.1016/j.anbehav.2009.06.035

[B298] Endrődi S (1960) Coleoptera: Melolonthidae, Subfamilia Dynastinae. In: Hanström B, Brinck P, Rudebeck G (Eds) South African Animal Life: Results of the Lund University Expedition in 1950–1951, vol. 7. Almqvist and Wiksells, Uppsala, 34–82.

[B299] Endrődi S (1963) Neue *Cyclocephala* Arten (Coleoptera, Melolonthidae, Dynastinae). Annales Historico-Naturales Musei Nationalis Hungarici 55: 323–333.

[B300] Endrődi S (1964) Eine Reihe von neuen *Cyclocephala*-Arten (Col., Melolonthidae, Dynastinae). Folia Entomologica Hungarica 17: 433–470.

[B301] Endrődi S (1966) Monographie der Dynastinae (Coleoptera, Lamellicornia). I. Teil. Entomologische Abhandlungen des Staatlichen Museums für Tierkunde Dresden 33: 1–460.

[B302] Endrődi S (1967a) Ergänzungen zur Kenntnis der Cyclocephalini. Entomologische Arbeiten aus dem Museum G. Frey Tutzing bei München 18: 406–411.

[B303] Endrődi S (1967b) Ergänzungen zu meiner Monographie der Dynastinae: Cyclocephalini (Coleoptera). Acta Zoologica Academiae Scientiarum Hungaricae 13: 83–91.

[B304] Endrődi S (1967c) Drei neue Arten der Tribe Cyclocephalini (Col., Dynastinae). Folia Entomologica Hungarica 20: 1–8

[B305] Endrődi S (1969a) The scientific results of the Hungarian Soil Zoological expeditions to South America 8. Dynastinae (Col., Melolonthidae). Folia Entomologica Hungarica 22: 377–382.

[B306] Endrődi S (1969b) Einige neue Cyclocephalini und Pentodontini (Coleoptera: Dynastinae). Acta Zoologica Academiae Scientiarum Hungaricae 15: 31–42.

[B307] Endrődi S (1970) Drei neue Dynastinen (Coleoptera, Melolonthidae) aus Amerika. Mitteilungen aus dem Museum für Naturkunde in Berlin. Zoologisches Museum und Institut für Spezielle Zoologie (Berlin) 46: 105–108. https://doi.org/10.1002/mmnz.19700460113

[B308] Endrődi S (1971a) Über neue und bekannte Dynastinen (Col., Melolonthidae). Folia Entomologica Hungarica 24: 179–184.

[B309] Endrődi S (1971b) Eine neue australische Pentodontine, *Teinogenys demarzi* n. sp. (Col. Melolonthidae). Entomologische Arbeiten aus dem Museum G. Frey Tutzing bei München 22: 105–108.

[B310] Endrődi S (1973a) Einige Dynastinen-Arten (Coleoptera: Melolonthidae) aus Bolivien. Opuscula Zoologica (Budapest) 12: 57–61.

[B311] Endrődi S (1973b) Neue Dynastinen-Formen (Coleoptera, Melolonthidae). Mitteilungen aus dem Zoologischen Museum in Berlin 49: 317–322. https://doi.org/10.1002/mmnz.4830490204

[B312] Endrődi S (1974) Monographie der Dynastinae (Col. Lamellicornia, Melolonthidae) 4. Tribus: Pentodontini der äthiopischen Region. Entomologische Arbeiten aus dem Museum G. Frey Tutzing bei München 25: 4–108.

[B313] Endrődi S (1975a) Zur Gattung *Surutu* Martínez (Coleoptera, Melolonthidae, Dynastinae). Annales Historico–Naturales Musei Nationalis Hungarici 67: 155–158.

[B314] Endrődi S (1975b) Neue Dynastinen aus dem Sonorischen und Neotropischen Gebiet (Coleoptera: Melolonthinae). Acta Zoologica Academiae Scientiarum Hungaricae 21: 257–262.

[B315] Endrődi S (1975c) *Cyclocephala hardyi* sp. n., eine neue Art aus Brasilien (Coleoptera: Melolonthidae, Dynastinae). Folia Entomologica Hungarica (N. S.) 28: 281 –284.

[B316] Endrődi S (1977a) *Aspidolea chalumeaui* sp. n., eine neue Dynastiden-Art aus Brasilien (Coleoptera). Folia Entomologica Hungarica 30: 5–6.

[B317] Endrődi S (1977b) *Alissonotum piceum bescheti* subsp. n. (Col. Melolonthidae, Dynastinae). Revue Suisse de Zoologie 84: 319–321. https://doi.org/10.5962/bhl.part.91390 https://doi.org/10.5962/bhl.part.91390

[B318] Endrődi S (1979) Neue Arten des Dynastinen Tribus Cyclocephalini (Coleoptera, Melolonthidae) aus Amerika. Annales Historico–Naturales Musei Nationalis Hungarici 71: 215–218.

[B319] Endrődi S (1980) Sechs neue Dynastinen-Arten aus Amerika und Borneo (Coleoptera: Dynastinae). Folia Entomologica Hungarica 33: 37–42.

[B320] Endrődi S (1981) Neue und seltene Dynastinen aus Südamerika und eine synonymische Bemerkung (Coleoptera, Melolonthidae). Annales Historico–Naturales Musei Nationalis Hungarici 73: 197–202.

[B321] Endrődi S (1985a) The Dynastinae of the World. Dr. W. Junk Publisher, Dordrecht, 800 pp. [+ 46 pls]

[B322] Endrődi S (1985b) Einige neue südamerikanische Dynastinae. Entomologische Blätter für Biologie und Systematik der Käfer 81: 69–74.

[B323] Endrődi S Dechambre R-P (1976) Note sur les types de *Harposcelis paradoxus* Burmeister (Col. Scarabaeidae Dynastinae). Bulletin de la Société Entomologique de France 81: 21–24.

[B324] Ennis DE Dillon AB Griffin CT (2010) Pine weevils modulate defensive behaviour in response to parasites of differing virulence. Animal Behaviour 80: 283–288. https://doi.org/10.1016/j.anbehav.2010.05.006

[B325] Erichson WF (1847a) Conspectus insectorum coleopterorum quae in Republica Peruana observata sunt. Archiv für Naturgeschichte 13: 67–185.

[B326] Erichson WF (1847b) Bericht über die wissenschaftlichen Leistungen in der Naturgeschichte der Insecten, Arachniden, Crustaceen und Entomostraceen während des Jahres 1846. Archiv für Naturgeschichte 13(2): 65–208.

[B327] Erichson WF (1848a) Insecten. In: Schomburgk R (Ed.) Reisen in British-Guiana in den Jahren 1840–1844. Versuch einer Fauna und Flora von British-Guiana 3. J. J. Weber, Leipzig, 553–617.

[B328] Erichson WF (1848b) Bericht über die wissenschaftlichen Leistungen in der Naturgeschichte der Insecten, Arachniden, Crustaceen u. Entomostraceen währen des Jahres 1847. Archiv für Naturgeschichte 14(2): 27–140.

[B329] Ervik F Tollsten L Knudsen JT (1999) Floral scent chemistry and pollination ecology in phytelephantoid palms (Arecaceae). Plant Systematics and Evolution 217: 279–297. https://doi.org/10.1007/BF00984371

[B330] Ervik F Knudsen JT (2003) Water lilies and scarabs: faithful partners for 100 million years? Biological Journal of the Linnean Society 80: 539–543. https://doi.org/10.1046/j.1095-8312.2003.00258.x

[B331] Eschscholtz JF von (1818) Decades tres eleutheratorum novorum descripsit. Mémoires de l’Académie Impériale des Sciences de St. Pétersbourg 6: 451–484.

[B332] Eschscholtz JF von (1822) Entomographien. First Edition. G. Reimer, Berlin, 128 pp. [+ 2 pls]

[B333] Evenhuis NL (2016) The insect and spider collections of the world website. http://hbs.bishopmuseum.org/codens/

[B334] Fabricius JC (1775) Systema entomologiae, sistens insectorum classes, ordines, genera, species, adiectis synonymis, locis, descriptionibus, observationibus. Officina Libraria Kortii, Flensburg and Leipzig, 832 pp.

[B335] Fabricius JC (1781) Species insectorum exhibentes eorum diffentias specificas, synonyma auctorum, loca natalia, metamorphosin adiectis observationibus, descriptionibus. Tom. I. Impensis Carol. Ernest. Bohnii, Hamburg and Kiel, 552 pp.

[B336] Fabricius JC (1787) Mantissa insectorum sistens eorum species nuper detectas adiectis characteribus genericis, differentiis specificis, emendationibus, observationibus. Volume 1. Impensis Christ. Gottl. Proft, Copenhagen, 348 pp. https://doi.org/10.5962/bhl.title.36471

[B337] Fabricius JC (1798) Supplementum entomologiae systematicae. Proft et Storch, Copenhagen, 572 pp.

[B338] Fabricius JC (1801) Systema eleutheratorum secundum ordines, genera, species: Adiectis synonymis, ocis, oberservationibus, descriptionibus. Volume 2. Impensis Bibliopolii Academici Novi, Kiel, 687 pp.

[B339] Faegri K van der Pijl L (1979) The principles of pollination ecology. Third revised edition. Pergamon Press, New York, 243 pp.

[B340] Fairmaire L (1892) Descriptions de quelques Coléoptères Argentins. Annales de la Société Entomologique de Belgique 36: 242–253.

[B341] Fairmaire L (1904) Descriptions de Lamellicornes indo-chinois nouveaux ou peu connus. Mission Pavie Indo-Chine 1879–1895, Études Diverses Recherches sur L’Histoire Naturelle de L’Indo-Chine Orientale 3: 86–90.

[B342] Fall HC (1901) List of the Coleoptera of southern California, with notes on habits and distribution. Occasional Papers of the California Academy of Sciences 8: 1–282.

[B343] Fall HC Cockerell DA (1907) The Coleoptera of New Mexico. Transactions of the American Entomological Society 33: 145–218.

[B344] Fauvel A (1861) Catalogue des insectes recueillis a la Guyane Française, par M. E. Déplanche, chirurgien auxiliaire de la marine impériale, pendant la campagne de l’aviso à vapeur le Rapide (Annérs 1854-55-56). Bulletin de la Société Linnéenne de Normandie 5: 299–327.

[B345] Fernández García I (2006) Coleópteres de la superfamilia Scarabaeoidea depositados en el Instituto de Ecología y Sistemática, Ciudad de La Habana. Cocuyo 16: 25–31.

[B346] Ferrú MA Elgueta M (2011) Lista de coleópteros (Insecta: Coleoptera) de las regiones de Arica y Parinacota y de Tarapacá, Chile. Boletín del Museo Nacional de Historia Natural, Chile 60: 9–61.

[B347] Figueroa-P A (1952) Catalago de los artropodos de las clases Arachnida e Insecta encontrados en el hombre, los animales u las plantas de la Republica de Colombia – II. Acta Agronomica 2: 199–223.

[B348] Figueroa L Ratcliffe BC (2016) A new species of *Ancognatha* Erichson (Coleoptera: Scarabaeidae: Dynastinae: Cyclocephalini) from Peru, with distributions of Peruvian *Ancognatha* species. The Coleopterists Bulletin 70: 65–72. https://doi.org/10.1649/072.070.0107

[B349] Fincher GT Stewart TB Davis R (1969) Beetle intermediate hosts for swine spirurids in southern Georgia. The Journal of Parasitology 55: 355–358. https://doi.org/10.2307/3277410 5813533

[B350] Fischer G (1823) Colèoptéra quaedam exotica, descripta. Mémoires de la Société des Naturalistes de Moscou 6: 254–267

[B351] Flanders KL DeLamar ZD Lago PK (2000) *Phyllophaga* and related species (Coleoptera: Scarabaeidae) collected in black-light traps in Alabama pastures. Journal of Entomological Science 35: 311–326. https://doi.org/10.18474/0749-8004-35.3.311

[B352] Fleutiaux E Sallé A (1889) Liste des Coléoptères de la Guadeloupe et descriptions d’espèces nouvelles. Annales de la Société Entomologique de France (series 6)9: 351–484.

[B353] Fleming WE (1948) Chlordan for control of Japanese beetle larvae. Journal of Economic Entomology 41: 905–912. https://doi.org/10.1093/jee/41.6.905 10.1093/jee/40.6.932 18858128

[B354] Forbes SA (1890) On the life history of the white grubs. Insect Life 3: 239–246.

[B355] Forbes SA (1891) On the common white grubs. (*Lachnosterna* and *Cyclocephala*). Report of the State Entomologist on the Noxious and Beneficial Insects of the State of Illinois 17: 30–50.

[B356] Forbes SA (1894) A monograph of insect injuries to Indian corn. Report of the State Entomologist on the Noxious and Beneficial Insects of the State of Illinois 18: 1–171.

[B357] Forbes SA (1896) Insect injuries to the seed and root of Indian corn. University of Illinois Agricultural Experiment Station Bulletin 44: 209–296.

[B358] Forbes SA (1907) On the life history, habits, and economic relations of the white-grubs and may-beetles. University of Illinois Agricultural Experiment Station Bulletin 116: 447–480. https://doi.org/10.5962/bhl.title.16718

[B359] Forschler BT Gardner WA (1991) Flight activity and relative abundance of phytophagous Scarabaeidae attracted to blacklight traps in Georgia. Journal of Agricultural Entomology 8: 179–187.

[B360] Foster RE Smith JP Cherry RH Hall DG (1986) *Dyscinetus morator* (Coleoptera: Scarabaeidae) as a pest of carrots and radishes in Florida. Florida Entomologist 69: 431–432. https://doi.org/10.2307/3494952

[B361] Frank JH (2008) Biological control of some insect pests of turfgrasses. In: Pessarakli M (Ed.) Handbook of turfgrass management and physiology. CRC Press, Boca Raton, 281–298.

[B362] Freitas FA de Zanuncio TV Lacerda MC Zanuncio JC (2002) Fauna de Coleoptera coletada com armadilhas luminosas em plantio de *Eucalyptus grandis* em Santa Bárbara, Minas Gerais. Revista Árvore 26: 505–511. https://doi.org/10.1590/S0100-67622002000400014

[B363] Frenzel J (1891) Uebersicht über eine Coleopterensammlung von Córdoba in Argentinien. Entomologische Nachrichten 17: 326–333.

[B364] Frost SW (1964) Insects taken in light traps at the Archbold Biological Stations, Highlands County, Florida. Florida Entomologist 47: 129–161. https://doi.org/10.2307/3493289

[B365] Frost SW (1966) Notes on common Scarabaeidae taken in light traps at Archbold Biological Station, Florida. Florida Entomologist 49: 189–194. https://doi.org/10.2307/3493442

[B366] Fuhrmann J (2013) Description of the third instar and pupa of *Geniates barbatus* Kirby (Coleoptera, Scarabaeidae, Rutelinae). Revista Brasileira de Entomologia 57: 40–46. https://doi.org/10.1590/S0085-56262013000100007

[B367] Fuxa JR (1998) Environmental manipulation for microbial control of insects. In: Barbosa P (Ed.) Conservation Biological Control. Academic Press, San Diego, 255–265. https://doi.org/10.1016/B978-012078147-8/50060-8

[B368] García-Atencia S Martínez-Hernández N (2015) Escarabajos fitófagos (Coleoptera: Scarabaeidae) del departamento del Atlántico, Colombia. Acta Zoológica Mexicana (n. s.) 31: 89–96. https://doi.org/10.1016/j.rmb.2015.07.009

[B369] García-Atencia S Martínez-Hernández N Pardo-Locarno LC (2015) Escarabajos fitófagos (Coleotpera: Scarabaeidae) en un fragment de bosque seco tropical del departmento del Atlántico, Colombia. Revista Mexicana de Biodiversidad 86: 754–763.

[B370] García-López A Micó E Numa C Galante E (2010) Spatiotemporal variation of scarab beetle assemblages (Coleoptera: Scarabaeidae: Dynastinae, Melolonthinae, Rutelinae) in the premontane rain forest in Costa Rica: A question of scale. Conservation Biology and Biodiversity 103: 956–964. https://doi.org/10.1603/AN10078

[B371] García-López A Micó E Zumbado MA Galante E (2011) Sampling scarab beetles in tropical forests: The effect of light source and night sampling periods. Journal of Insect Science 11(95): 1–14. https://doi.org/10.1673/031.011.9501 10.1673/031.011.9501 PMC3281455 22208730

[B372] García-López A Micó E Múrria C Galante E Vogler AP (2013) Beta diversity at multiple hierarchical levels: explaining the high diversity of scarab beetles in tropical montane forests. Journal of Biogerography 40: 2134–2145. https://doi.org/10.1111/jbi.12148

[B373] García-Luna D Morón MA Rojas-Gómez CV (2002) Variación en los patrones de pigmentación en tres especies de *Cyclocephala* Burmeister (Coleoptera: Melolonthidae; Dynastinae). Folia Entomológica Mexicana 41: 129–148.

[B374] García-Rivera G Contreras-Ramos A (2015) First record of *Dyscinetus laevipunctatus* Bates (Coleoptera: Scarabaeidae, Dynastinae) in an aquatic environment in Mexico. Entomological News 125: 63–69. https://doi.org/10.3157/021.125.0112

[B375] García-Robledo C (2010) Restoration of plant-pollinator interactions: pollination neighborhood and asymmetric pollen flow between restored habitats in a beetle-pollinated aroid. Restoration Ecology 18: 94–102. https://doi.org/10.1111/j.1526-100X.2010.00677.x

[B376] García-Robledo C Kattan G Murcia C Quintero-Marín P (2004) Beetle pollination and fruit predation of *Xanthosoma daguense* (Araceae) in an Andean cloud forest in Colombia. Journal of Tropical Ecology 20: 459–469. https://doi.org/10.1017/S0266467404001610

[B377] García-Robledo C Quintero-Marín P Mora-Kepfer F (2005) Geographic variation and succession of arthropod communities in inflorescences and infructescences of *Xanthosoma* (Araceae). Biotropica 37: 650–656. https://doi.org/10.1111/j.1744-7429.2005.00082.x

[B378] Gardner DS (2013) Warm weather turf troubles. SportsField Management July 2013: 20–23.

[B379] Gasca-Álvarez HJ (2013) New records of *Cyclocephala* Dejean (Coleoptera: Scarabaeidae: Dynastinae) associated with *Caladium bicolor* (Aiton) Vent. (Araceae). The Coleopterists Bulletin 67: 416–418. https://doi.org/10.1649/0010-065X-67.4.416

[B380] Gasca-Álvarez HJ (2014) Sobre la taxonomía y biología de *Cyclocephala mannheimsi* Endrődi, 1964 (Coleoptera: Scarabaeidae: Dynastinae), nuevo registro para Colombia. Acta Zoológica Mexicana (n. s.), 30(1): 174–187. https://doi.org/10.21829/azm.2014.301137

[B381] Gasca-Álvarez HJ Amat-García G (2010) Synopsis and key to the genera of Dynastinae (Coleoptera, Scarabaeoidea, Scarabaeidae) of Colombia. ZooKeys 34: 153–192. https://doi.org/10.3897/zookeys.34.309

[B382] Gasca-Álvarez HJ Ratcliffe BC Deloya C (2014) Redescription and occurrence in Suriname and Colombia of *Cyclocephala guianae* Endrődi (Coleoptera: Scarabaeidae: Dynastinae: Cyclocephalini). Dugesiana 21: 131–133.

[B383] Gasca-Álvarez HJ Deloya C (2016) Description of the female of *Cyclocephala monacha* Ratcliffe, 2008 (Coleoptera: Scarabaeidae: Dynastinae: Cyclocephalini), with a checklist of *Cyclocephala* Dejean species occurring in Colombia. The Coleopterists Bulletin 70: 645–653. https://doi.org/10.1649/0010-065X-70.3.645

[B384] Gaugler R Georgis R (1991) Culture method and efficacy of entomopathogenic nematodes (Rhabditida: Steinernematidae and Heterorhabditidae). Biological Control 1: 269–274. https://doi.org/10.1016/1049-9644(91)90077-D

[B385] Gavotto ALR de (1964) Ciclo biológico de *Cyclocephala signaticollis* Burm. (Col. Scarabaeidae) y caracteres especificos de su larva. Revista de Investigaciones Agropecuarias. Serie 5. Patología Vegetal 1: 151–161.

[B386] Georgis R Koppenhöfer AM Lacey LA Bélair G Duncan LW Grewal PS Samish M Tan L Torr P van Tol RWHM (2006) Successes and failures in the use of parasitic nematodes for pest control. Biological Control 38: 103–123. https://doi.org/10.1016/j.biocontrol.2005.11.005

[B387] Gerhardt PD Stanghellini ME (1971) Scarab larvae killing sugarbeet seedlings. Journal of Economic Entomology 64: 1311–1312. https://doi.org/10.1093/jee/64.5.1311

[B388] Germar EF (1824) Coleopterorum species novae aut minus cognitae, descriptionibus illustratae. Impensis J. S. Hendelii et Filii; Halle, Germany, 624 pp. [+ 2 pl.] https://doi.org/10.5962/bhl.title.130964

[B389] Gerstaecker A (1858) Bericht über die wissenschaftlichen Leistungen im Gebiete der Entomologie während des Jahres 1857. Archiv für Naturgeschichte 24(2): 193–480.

[B390] Gerstaecker A (1862) Bericht über die wissenschaften Leistungen im Gebiete Entomologie während des Jahres 1861. Archiv für Naturgeschichte 28(2): 273–571.

[B391] Gerstaecker A (1865) Bericht über die wissenschaftlichen Leistungen im Gebiete der Entomologie während der J. 1863–64. Archiv für Naturgeschichte 31(2): 269–681.

[B392] Gerstaecker A (1866) Bericht über die wissenschaftlichen Leistungen im Gebiete der Entomologie während der Jahre 1865–66. Archiv für Naturgeschichte 32(2): 281–468.

[B393] Gessner F (1962) A abertura das flôres de *Victoria regia*, em relação à luz. Boletim do Museu Paranense Emílio Goeldi 17: 1–13.

[B394] Ghys MI Favero M (2004) Espectro trófico de la gaviota capucho café (*Larus maculipennis*) en agroecosistemas del sudeste de la provincia de Buenos Aires, Argentina. Ornitologia Neotropical 5: 493–500.

[B395] Giannoulis T Dutrillaux A-M Stamatis C Dutrillaux B Mamuris Z (2012) *Cyclocephala* (Coleoptera: Scarabaeidae: Dynastinae) evolution in Lesser West Indies indicates a northward colonization by *C. tridentata*. Bulletin of Entomological Research 102: 325–332. https://doi.org/10.1017/S0007485311000733 10.1017/S0007485311000733 22112675

[B396] Gibbs PE Semir J Cruz ND da (1977) Floral biology of *Talauma ovata* St. Hil. (Magnoliaceae). Ciéncia e Cultura 29: 1437–1441.

[B397] Gibernau M (2003) Pollinators and visitors of aroid inflorescences. Aroideana 26: 66–83.

[B398] Gibernau M (2011) Pollinators and visitors of aroid inflorescences: an addendum. Aroideana 34: 70–83.

[B399] Gibernau M (2015a) Floral biology, pollination ecology and genetics of *Dieffenbachia* (Araceae)-A review. Aroideana 38: 19–28.

[B400] Gibernau M (2015b) Pollination ecology of two *Dieffenbachia* in French Guiana. Aroideana 38: 38–64.

[B401] Gibernau M Barabé D (2002) Pollination ecology of *Philodendron squamiferum* (Araceae). Canadian Journal of Botany 80: 316–320. https://doi.org/10.1139/b02-006

[B402] Gibernau M Barabé D Cerdan P Dejean A (1999) Beetle pollination of *Philodendron solimoesense* (Araceae) in French Guiana. International Journal of Plant Science 160: 1135–1143. https://doi.org/10.1086/314195 10.1086/314195 10568780

[B403] Gibernau M Barabé D Labat B (2000) Flowering and pollination of *Philodendron melinonii* (Araceae) in French Guiana. Plant Biology 2: 331–334. https://doi.org/10.1055/s-2000-3712

[B404] Gibernau M Barabé D Labat D Cerdan P Dejean A (2003) Reproductive biology of *Montrichardia arborescens* (Araceae) in French Guiana. Journal of Tropical Ecology 19: 103–107. https://doi.org/10.1017/S0266467403003134

[B405] Gibernau M Chartier M Barabé D (2010) Recent advances towards an evolutionary comprehension of Araceae pollination. In: Seberg O, Petersen G, Barfod AS, Davis JI (Eds) Diversity, phylogeny, and evolution in the Monocotyledons. Aarhus University Press, Aarhus, 101–114.

[B406] Gibson WW Carrillo SJ (1959) Lista de insectos en la coleccion entomologica de la Oficina de Estudios Especiales, S.A.G. Folleto Misceláneo 9: 1–254.

[B407] Gillett CPDT Gillett MPT (2015) The Dynastinae of the island of Saba, Dutch Caribbean (Coleoptera: Scarabaeidae). Insecta Mundi 0433: 1–9.

[B408] Goldwasser LP (1987) I. Branching patterns, generating rules, and astrogenetic trajectories in *Bugula* (Cheilostomata, Bryozoa). II. Mutualism and its ecological and evolutionary consequences. PhD thesis, Berkley, USA: University of California-Berkeley.

[B409] Goldwasser LP (2000) Scarab beetles, elephant ear (*Xanthosoma robustum*), and their associates. In: Natkarni NM, Wheelwright NT (Eds) Monteverde. Ecology and Conservation of a Tropical Cloud Forest. Oxford University Press, New York, 268–271.

[B410] Gómez B Villalobos FJ Ruíz L Castro AE (1999) Observaciones sobre la biología de melolóntidos (Coleoptera: Scarabaeoidea) en una localidad de Los Altos de Chiapas, Mexico. Acta Zoológica Mexicana (Nueva Serie) 78: 173–177.

[B411] Gómez B Morón MA (2010) Los escarabajos de bosques de niebla en Chiapas (Coleoptera: Melolonthidae). In: Farrera MAP, Cruz CT, Rivera ES (Eds) Los bosques mesófilos de montaña en Chiapas: Situación actual, diversidad y conservación. Univsersidad de Ciencias y Artes de Chiapas (UNICACH), Tuxtla Gutiérrez, 207–225.

[B412] Gonçalves EG Maia ACD (2006) New evidence of pollination in *Gearum brasiliense* (Araceae: Spathicarpeae). Aroideana 29: 148–151.

[B413] González JR Caballero A Montalván J (1998) Gradiente ambiental de la comunidad de insectos asociados al cultivo de la caña de azúcar en Cuba. Cultivos Tropicales 19: 69–73.

[B414] Gordon RD Anderson DM (1981) The species of Scarabaeidae (Coleoptera) associated with sugarcane in south Florida. Florida Entomologist 64: 119–138. https://doi.org/10.2307/3494604

[B415] Gottsberger G (1986) Some pollination strategies in Neotropical Savannas and Forests. Plant Systematics and Evolution 152: 29–45. https://doi.org/10.1007/BF00985349

[B416] Gottsberger G (1988) The reproductive biology of primitive angiosperms. Taxon 37: 630–643. https://doi.org/10.2307/1221105

[B417] Gottsberger G (1989) Beetle pollination and flowering rhythm of *Annona* spp. (Annonaceae) in Brazil. Entwicklungsgeschichte und Systematik der Planzen 167: 165–187.

[B418] Gottsberger G (1990) Flowers and beetles in the South American tropics. Botanica Acta 103: 360–365. https://doi.org/10.1111/j.1438-8677.1990.tb00175.x

[B419] Gottsberger G (1992) Diversität der Bestäubung und Reproduktionsbiologie von ursprünglichen Angiospermen. Stapfia 28: 11–27.

[B420] Gottsberger G (1999) Pollination and evolution in neotropical Annonaceae Plant Species Biology 14: 143–152. https://doi.org/10.1046/j.1442-1984.1999.00018.x

[B421] Gottsberger G (2016) Generalist and specialist pollination in basal angiosperms (ANITA grade, basal monocots, magnoliids, Chloranthaceae and Ceratophyllaceae): what we know now. Plant Diversity and Evolution 131: 263–362. https://doi.org/10.1127/pde/2015/0131-0085

[B422] Gottsberger G Amaral A (1984) Pollination strategies in Brazilian *Philodendron* species. Berichte der Deutschen Botanischen Gesellschaft 97: 391–410.

[B423] Gottsberger G Silberbauer-Gottsberger I (1988) Pollination strategies of *Annona* species from the cerrado vegetation in Brazil. Lagascalia 15: 665–672.

[B424] Gottsberger G Silberbauer-Gottsberger I (1991) Olfactory and visual attraction of *Erioscelis emarginata* (Cyclocephalini, Dynastinae) to the inflorescences of *Philodendron selloum* (Araceae). Biotropica 23: 23–28. https://doi.org/10.2307/2388684

[B425] Gottsberger G Silberbauer-Gottsberger I (2006) Life in the Cerrado: a South American Tropical Seasonal Ecosystem. Volume 2. Pollination and Seed Dispersal. Reta Verlag, Ulm, 383 pp.

[B426] Gottsberger G Silberbauer-Gottsberger I Seymour RS Dötterl S (2012) Pollination ecology of *Magnolia ovata* may explain the overall large flower size of the genus. Flora-Morphology, Distribution, Functional Ecology of Plants 207: 107–118. https://doi.org/10.1016/j.flora.2011.11.003

[B427] Gottsberger G Silberbauer-Gottsberger I Dötterl S (2013) Pollination and floral scent differentiation in species of the *Philodendron bipinnatifidium* complex (Araceae). Plant Systematics and Evolution 299: 793–809. https://doi.org/10.1007/s00606-013-0763-4

[B428] Gottsberger G Webber AC Hildenbrand M (1998) Nutritious tissues in flowers of Annonaceae Annonaceae Newsletter 12: 25–26.

[B429] Grayum MH (1996) Revision of Philodendron subgenus Pteromischum (Araceae) for Pacific and Caribbean Tropical America. Systematic Botany Monographs 47: 1–233. https://doi.org/10.2307/25027858

[B430] Grewal PS Grewal SK Malik VS Klein MG (2002) Differences in susceptibility of introduced and native white grub species to entomopathogenic nematodes from various geographic localities. Biological Control 24: 230–237. https://doi.org/10.1016/S1049-9644(02)00025-7

[B431] Grewal PS Koppenhöffer AM Choo HY (2005) Lawn, turfgrass and pasture applications. In: Grewal PS, Ehlers R-U, Shapiro-Ilan DI (Eds) Nematodes as biocontrol agents, CABI Publishing, Cambridge, 115–146. https://doi.org/10.1079/9780851990170.0115

[B432] Grewal PS Power KT Grewal SK Suggars A Haupricht S (2004) Enhanced consistency in biological control of white grubs (Coleoptera: Scarabaeidae) with new strains of entomopathogenic nematodes. Biological Control 30: 73–82. https://doi.org/10.1016/j.biocontrol.2003.09.016

[B433] Grewal PS Power KT Shetlar DJ (2001) Neonicotinoid insecticides alter diapause behavior and survival of overwintering white grubs (Coleoptera: Scarabaeidae). Pest Management Science 57: 852–857. https://doi.org/10.1002/ps.373 10.1002/ps.373 11561413

[B434] Grimm R (2009) *Peltonotus nasutus* Arrow, 1910 und Phaeochrous-Arten als Bestäuber von Amorphophallus paeoniifolius (Araceae) in Thailand (Coleoptera: Scarabaeidae). Entomologische Zeitschrift 119: 167–168.

[B435] Groba HF Castelo MK (2012) Chemical interaction between the larva of a dipteran parasitoid and its coleopteran host: A case of exploitation of the communication system during the searching behavior? Bulletin of Entomological Research 102: 315–323. https://doi.org/10.1017/S0007485311000691 10.1017/S0007485311000691 22127012

[B436] Grossi PC Leivas FWT Almeida LM de (2011) Dynastinae (Coleoptera: Scarabaeoidea: Melolonthidae) dos campos gerais, Paraná, Brasil. In: Marcello G (Ed.) Coletânea de Pesquisas: Parques Estaduais de Vila Velha, Cerrado e Guartelá, volume 1, number 1. Instituto Ambiental do Paraná, Curitiba, 113–123.

[B437] Grossi PC Santos MD Almeida LM (2016) Two new species of *Cyclocephala* (Coleoptera: Scarabaeoidea: Melolonthidae) from Minas Gerais State, Brazil. Zootaxa 4078: 245–251. https://doi.org/10.11646/zootaxa.4078.1.22 10.11646/zootaxa.4078.1.22 27395978

[B438] Gruner L (1971) Scarabaeidae Melolonthidae, Dynastinae, Rutelinae, Cetoniinae [Coleoptera] récoltés en Guyane Française par la mission du Muséum National D’Histoire Naturelle (1). Société Entomologique de France (N. S.) 7: 843–848.

[B439] Gruner L (1975) Étude de l’activité des adultes de divers scarabeides Antillais au moyen de piégeages lumineux et chimiques. Annales de Zoologie-Écologie Animale 7: 399–423.

[B440] Gruner L Marival D (1974) Attraction des males du scarabeide Antillais *Cyclocephala insulicola* Arrow par le phénol (Coleoptera: Dynastinae). Comptes Rendus de l’Academie d’Agriculture de France 60: 203–208.

[B441] Guérin J (1953) Coleopteros do Brasil. Departamentos de Zoologia e de Fisiologia Geral e Animal, Faculdade de Filosofia, Ciências e Letras, Universidade de São Paulo, São Paulo, 356 pp.

[B442] Guimarães LR (1944) Rutelidae, Cetonidae, Melolonthidae e Dynastidae de Monte Alegre. Papéis Avulsos do Departamento de Zoologia (São Paulo) 6: 93–102.

[B443] Gundlach J (1891) Contribucion á la entomologia Cubana. Tomo 3. A. Alvarez y Comp, Havana, 404 pp.

[B444] Guo L Desneux N Sonoda S Liang P Han P Gao X-W (2013) Sublethal and transgenerational effects of chlorantraniliprole on biological traits of the diamondback moth, *Plutella xylostella* L. Crop Protection 48: 29–34. https://doi.org/10.1016/j.cropro.2013.02.009

[B445] Gutiérrez AR (1950) Scarabaeidae del Norte de Chile. Anales de la Sociedad Científica Argentina 149 (2): 52–75.

[B446] Gyawaly S Koppenhöfer AM Wu S Kuhar TP (2016) Biology, ecology, and management of masked chafer (Coleoptera: Scarabaeidae) grubs in turfgrass. Journal of Integrated Pest Management 7(1): 1–11. https://doi.org/10.1093/jipm/pmw002

[B447] Gyawaly S Youngman RR Laub CA Kuhar TP (2015) Evaluation of insecticide application timings against white grubs in cool season turfgrass in Viriginia, 2013. Arthropod Management Tests 39 (1): G10.

[B448] Gyllenhal L (1817a) In: Schönherr CJ (Ed.) Synonymia Insectorum, oder Versuch einer Synonymie aller bisher bekannten Insecten; nach Fabricii Systema Eleutheratorum etc. geordnet, volume 1, part 3. Em. Bruzelius, Uppsala, 506 pp.

[B449] Gyllenhal L (1817b) In: Schönherr CJ (Ed.) Appendix ad C. J. Schönherr synonymiam insectorum sistens descriptions novarum specierum. Volume 1, Part 3. Scaris, in officina Lewerentziana, Stockholm, 266 pp. [+ 2 pls]

[B450] Hagstrum DW Subramanyam B (2009) Stored-product insect resource. AACC International, St. Paul, 509 pp.

[B451] Hardy AR (1974) A new species of *Cyclocephala* Latreille from California sand dunes (Coleoptera: Scarabaeidae). The Pan-Pacific Entomologist 50: 160–161.

[B452] Hardy AR (1991) A catalog of the Coleoptera of America north of Mexico. Family: Scarabaeidae, Subfamilies: Rutelinae and Dynastinae. United States Department of Agriculture, Agriculture Handbook 529-34b. Agricultural Research Service, Washington, District of Columbia, xi + 57 pp.

[B453] Harold E (1869a) Abänderungen vergebener Namen. In: Harold E (Ed.) Coleoptergische Hefte, Vol. 1, Part 5. Carl Merhoff’s Verlag, Munich, 122–125.

[B454] Harold E (1869b) Scarabaeidae. In: Gemminger M, Harold E (Eds) Catalogus Coleopterorum Hucusque Descriptorum Synonymicus et Systematicus, Vol.4. E. H. Gummi, Munich, 979–1346.

[B455] Harpootlian PJ (2001) Scarab beetles (Coleoptera: Scarabaeidae) of South Carolina. Biota of South Carolina. Vol. 2. Clemson University, Clemson, 157 pp.

[B456] Harris CR (1977) Insecticide resistance in soil insects attacking crops. In: Watson DL, Brown AWA (Eds) Pesticide management and insecticide resistance. Academic Press, New York, 321–351. https://doi.org/10.1016/B978-0-12-738650-8.50026-6

[B457] Harris ED (1959) Observations on the occurrence of a milky disease among larvae of the Northern Masked Chafer, *Cyclocephala borealis* Arrow. Florida Entomologist 42: 81–83. https://doi.org/10.2307/3492139

[B458] Harris JE (2006) Insect light traps. In: Heaps JW (Ed.) Insect management for food storage and processing. Second edition. AACC International, St. Paul, 55–66. https://doi.org/10.1016/B978-1-891127-46-5.50013-1

[B459] Hatch MH (1971) The beetles of the Pacific Northwest. Part V. Rhipiceroidea, Sternoxi, Phytophaga, Rhynchophora, and Lamellicornia. University of Washington Publications in Biology 16: 1–662.

[B460] Hatch SL White RH (2004) Additional C_4_ turf and forage grasses. In: Moser LE, Burson BL, Sollenberger LE (Eds) Warm-season (C_4_) grasses. Agronomy Monograph 45. American Society of Agronomy, Crop Science Society of America, Soil Science Society of America, Madison, 1081–1119.

[B461] Haviland DR Hernandez NM (2012) Development of management programs for white grubs in California blueberries. International Journal of Fruit Science 12: 114–123. https://doi.org/10.1080/15538362.2011.619355

[B462] Hay A Gottschalk M Holguin A (2012) Huanduj: Brugmansia. Kew Publishing, London, 424 pp.

[B463] Hayes WP (1918) Studies on the life-history of two Kansas Scarabaeidae (Coleop.). Journal of Economic Entomology 11: 136–144. https://doi.org/10.1093/jee/11.1.136

[B464] Hayes WP (1928) The epipharynx of lamellicorn larvae (Coleop.), with a key to common genera. Annals of the Entomological Society of America 21: 282–306. https://doi.org/10.1093/aesa/21.2.282

[B465] Hayes WP (1929) Morphology, taxonomy, and biology of larval Scarabaeoidea. Illinois Biological Monographs 12: 1–119.

[B466] Hayes WP McColloch JW (1928) Ecological studies of Kansas scarabaeid larvae (Coleop.). Journal of Economic Entomology 21: 249–260. https://doi.org/10.1093/jee/21.2.249

[B467] Haynes KF Potter DA (1995) Chemically mediated sexual attraction of male *Cyclocephala lurida* (Coleoptera: Scarabaeidae) and other scarabaeid beetles to immature stages. Environmental Entomology 24: 1302–1306. https://doi.org/10.1093/ee/24.5.1302

[B468] Haynes KF Potter DA Collins JT (1992) Attraction of male beetles to grubs: evidence for evolution of a sex pheromone from larval odor. Journal of Chemical Ecology 18: 1117–1124. https://doi.org/10.1007/BF00980067 10.1007/BF00980067 24254152

[B469] Hayward KJ (1946) Departamento de Entomología. Revista Industrial y Agrícola de Tucumán 36: 60–72.

[B470] Heikertinger F (1919) Die Koleopteren-Schausammlung des Naturhistorischen Museums in Wien. Koleopterologische Rundschau 8: 1–14.

[B471] Held DW Eaton T Rogers M Gels J Lopez R Eliason E Potter DA (2000a) Comparison of Mach-2 (halofenozide), Merit (imidacloprid), and Meridian (CGA 293, 343: thiamethoxam) applied for control of white grubs in turf, 1999. Arthropods Management Tests 25(1): G24. https://doi.org/10.1093/amt/25.1.G24

[B472] Held DW Kreuger B Mason N Lopez R Eliason E Walston A Potter DA (2000b) Effect of post-treatment irrigation on the efficacy of CGA 293,343 (Meridian) against white grubs in turf 1998. Arthropods Management Tests 25(1): G23. https://doi.org/10.1093/amt/25.1.G23

[B473] Held DW Ray CH (2009) Asiatic garden beetle *Maladera castanea* (Coleoptera: Scarabaeidae) grubs found in damaged turf in Alabama. Florida Entomologist 92: 670–672. https://doi.org/10.1653/024.092.0426

[B474] Held DW Walston A Eliason E Kreuger B Mason N Potter DA (2000c) Effect of timing on the efficacy of Meridian (CGA 293,343: thiamethoxam) for control of white grubs in turf, 1998. Arthropods Management Tests 25(1): G22. https://doi.org/10.1093/amt/25.1.G22

[B475] Held DW Walston A Eliason E Kreuger B Mason N Potter DA (2000d) Efficacy of Mach-2 formulations applied for post-egg lay (PEL) control of white grubs in turg, 1998. Arthropod Management Tests 25(1): G25. https://doi.org/10.1093/amt/25.1.G25

[B476] Heller PR Kline D (2005a) Curative applications of Provaunt and Merit formulations to suppress scarab white grubs, 2004. Arthropod Management Tests 30(1): G32. https://doi.org/10.1093/amt/30.1.G32

[B477] Heller PR Kline D (2005b) Mach 2 formulation application timing efficacy study, 2004. Arthropod Management Tests 30(1): G40. https://doi.org/10.1093/amt/30.1.G40

[B478] Heller PR Kline D (2005c) Mach 2 preventive application formulation white grub efficacy study, 2004. Arthropod Management Tests 30(1): G41. https://doi.org/10.1093/amt/30.1.G41

[B479] Heller PR Kline D (2007a) Application of DPXE2Y45 and a Scotts experimental granular to preventively suppress scarab white grubs, 2006. Arthropod Management Tests 32(1): G16. https://doi.org/10.1093/amt/32.1.G16

[B480] Heller PR Kline D (2007b) Curative timing study with applications of DPXE2Y45 and Dylox to suppress scarab white grubs, 2006. Arthropod Management Tests 32 (1): G17. https://doi.org/10.1093/amt/32.1.G17

[B481] Heller PR Kline D (2007c) Preventive application of Arena at various rates to suppress white grubs, 2006. Arthropod Management Tests 32(1): G18. https://doi.org/10.1093/amt/32.1.G18

[B482] Heller PR Kline D (2007d) Early May application of Arena to preventively suppress white grubs, 2006. Arthropod Management Tests 32(1): G19. https://doi.org/10.1093/amt/32.1.G19

[B483] Heller PR Kline D (2007e) Early july application of Merit to preventively suppress white grubs, 2006. Arthropod Management Tests 32(1): G20. https://doi.org/10.1093/amt/32.1.G20

[B484] Heller PR Kline D (2007f) Preventive applications of Meridian formulations and Merit 75WP to suppress white grubs, 2006. Arthropod Management Tests 32(1): G21. https://doi.org/10.1093/amt/32.1.G21

[B485] Heller PR Kline D (2007g) Mid-June application of DPXE2Y45 and Merit formulations to preventively suppress scarab white grubs, 2006. Arthropod Management Tests 32(1): G15. https://doi.org/10.1093/amt/32.1.G15

[B486] Heller PR Kline D (2007h) Mid-May application of DPXE2Y45 and Merit to preventively suppress scarab white grubs, 2006. Arthropod Management Tests 32(1): G14. https://doi.org/10.1093/amt/32.1.G14

[B487] Heller PR Kline D (2007i) Mid-April application of DPXE2Y45 and Merit to preventively suppress scarab white grubs, 2006. Arthropod Management Tests 32(1): G13. https://doi.org/10.1093/amt/32.1.G13

[B488] Heller PR Kline D Ellis PJ (2006a) Application of Allectus and Merit to preventively suppress white grubs at three timing intervals, 2005. Arthropod Management Tests 31(1): G34. https://doi.org/10.1093/amt/31.1.G34

[B489] Heller PR Kline D Ellis PJ (2006b) Application of Arena and Merit to suppress white grubs at preventive and curative timing intervals, 2005. Arthropod Management Tests 31(1): G35. https://doi.org/10.1093/amt/31.1.G35

[B490] Heller PR Kline D Ellis PJ (2006c) Late summer curative scarab grub suppression with trichlorfon, 2005. Arthropod Management Tests 31(1): G39. https://doi.org/10.1093/amt/31.1.G39

[B491] Heller PR Kline D Ellis PJ (2006d) Mid-August scarab grub efficacy study evaluating applications of DPXE2Y45 and Merit, 2005. Arthropod Management Tests 31(1): G40. https://doi.org/10.1093/amt/31.1.G40

[B492] Heller PR Kline D Ellis PJ (2006e) Early July application of DPXE2Y45, Grubex, and Merit to preventively suppress scarab white grubs, 2005. Arthropod Management Tests 31(1): G38. https://doi.org/10.1093/amt/31.1.G38

[B493] Heller PR Kline D Ellis PJ (2006f) Application of DPXE2Y45 and Merit to preventively suppress white grubs based at two timing intervals, 2005. Arthropod Management Tests 31(1): G36. https://doi.org/10.1093/amt/31.1.G36

[B494] Heller PR Kline D Houseman A (2008a) Preventive timing study with Aloft and Merit formulations to suppress scarab white grubs, 2007. Arthropod Management Tests 33(1): G11.

[B495] Heller PR Kline D Houseman A (2008b) Preventive timing study with Aloft, Arena, and Merit formulations to suppress scarab white grubs, 2007. Arthropod Management Tests 33(1): G12. https://doi.org/10.1093/amt/33.1.G12

[B496] Heller PR Kline D Houseman A (2008c) Preventive timing study with Meridian and Merit formulations to suppress scarab white grubs, 2007. Arthropod Management Tests 33(1): G13. https://doi.org/10.1093/amt/33.1.G13

[B497] Heller PR Kline D Houseman A (2008d) Curative timing application of Meridian, Dylox, and Arena formulations to suppress scarab white grubs, 2007. Arthropod Management Tests 33(1): G14. https://doi.org/10.1093/amt/33.1.G14

[B498] Heller PR Kline D Houseman A (2008e) Allectus application timing study to suppress scarab white grubs, 2007. Arthropod Management Tests 33(1): G15. https://doi.org/10.1093/amt/33.1.G15

[B499] Heller PR Kline D Houseman A (2008f) Mid-April application of Acelepyrn and Merit formulations to preventively suppress scarab white grubs, 2007. Arthropod Management Tests 33 (1): G16.

[B500] Heller PR Kline D Houseman A (2008g) Mid-May application of Acelepyrn granular and SC formulations compared with Merit formulations to preventively suppress scarab white grubs, 2007. Arthropod Management Tests 33(1): G21.

[B501] Heller PR Kline D Houseman A (2008h) Mid-July application of Acelepryn and Merit formulations to preventively suppress scarab white grubs, 2007. Arthropod Management Tests 33(1): G23. https://doi.org/10.1093/amt/33.1.G23

[B502] Heller PR Kline D Houseman A (2008i) Curative timing application of insect parasitic nematodes and Dylox 6.2G to suppress scarab white grubs, 2007. Arthropod Management Tests 33(1): G24. https://doi.org/10.1093/amt/33.1.G24

[B503] Heller PR Kline D Houseman A (2008j) Early May application of Acelepryn, Meridian, and Merit formulations to preventively suppress scarab white grubs, 2007. Arthropod Management Tests 33(1): G17. https://doi.org/10.1093/amt/33.1.G17

[B504] Heller PR Kline D Houseman A (2008k) Mid June application of Acelepryn, Meridian, and Merit formulations to preventively suppress scarab white grubs, 2007. Arthropod Management Tests 33(1): G18. https://doi.org/10.1093/amt/33.1.G18

[B505] Heller PR Kline D Houseman A (2008l) Mid-June irrigation study of Acelepryn, Mach 2, and Merit formulations to preventively suppress scarab white grubs, 2007. Arthropod Management Tests 33(1): G19. https://doi.org/10.1093/amt/33.1.G19

[B506] Heller PR Kline D Houseman A (2008m) Mid-May application of Acelepryn, Grubex, and Merit-fertilizer formulations to preventively suppress scarab white grubs, 2007. Arthropod Management Tests 33(1): G22. https://doi.org/10.1093/amt/33.1.G22

[B507] Heller PR Kline D Houseman A (2009a) Curative study with Dylox 80 to suppress scarab white grubs, 2008. Arthropods Management Tests 34(1): G11. https://doi.org/10.4182/amt.2009.G11

[B508] Heller PR Kline D Houseman A (2009b) Efficacy of arena formulations to preventively suppress white grubs when applied at peak egglay, 2008. Arthropod Management Tests 34(1): G18. https://doi.org/10.4182/amt.2009.G18

[B509] Heller PR Kline D Houseman A (2009c) Preventive application study with Allectus and Aloft formulations to suppress scarab white grubs, 2008. Arthropod Management Tests 34(1): G43. https://doi.org/10.4182/amt.2009.G43

[B510] Heller PR Kline D Houseman A (2009d) Preventive applications of Arena and Meridian formulations to suppress scarab white grubs, 2008. Arthropod Management Tests 34(1): G21. https://doi.org/10.4182/amt.2009.G21

[B511] Heller PR Kline D Houseman A (2009e) Curative applications of Arena and experimental formulations to suppress scarab white grubs, 2008. Arthropod Management Tests 34(1): G19. https://doi.org/10.4182/amt.2009.G19

[B512] Heller PR Kline D Houseman A (2009f) Efficacy of Arena formulations to preventively suppress scarab white grubs when applied at peak adult beetle flight, 2008. Arthropod Management Tests 34(1): G17. https://doi.org/10.4182/amt.2009.G17

[B513] Heller PR Kline D Houseman A (2009g) Preventive applications of Merit to suppress scarab white grubs, 2008. Arthropod Management Tests 34(1): G20. https://doi.org/10.4182/amt.2009.G20

[B514] Heller PR Kline D Houseman A (2009h) Preventive applications of experimental and Grubex formulations to suppress scarab white grubs, 2008. Arthropod Management Tests 34(1): G22. https://doi.org/10.4182/amt.2009.G22

[B515] Heller PR Kline D Houseman A (2009i) Timing application study with Aloft, Merit, and experimental formulations to suppress scarab white grubs, 2008. Arthropod Management Tests 34(1): G12. https://doi.org/10.4182/amt.2009.G12

[B516] Heller PR Kline D Houseman A (2009j) Preventive timing and irrigation study with applications of Acelepryn and Merit formulations to suppress scarab white grubs, 2008. Arthropod Management Tests 34(1): G42. https://doi.org/10.4182/amt.2009.G42

[B517] Heller PR Kline D Houseman A (2009k) Preventive study with applications of experimental formulations, Acelepryn and Merit to suppress scarab white grubs, 2008. Arthropod Management Tests 34(1): G41. https://doi.org/10.4182/amt.2009.G41

[B518] Heller PR Kline D Houseman A (2009l) Curative timing study with applications of experimental formulations, Acelepryn, Arena, and Dylox 80SP to suppress scarab white grubs, 2008. Arthropod Management Tests 34(1): G13. https://doi.org/10.4182/amt.2009.G13

[B519] Heller PR Walker R (1999a) Curative management of white grubs with 1.5G experimental formulations of halofenozide and Mach 2 liquid insecticide for residual suppression of white grubs, 1998. Arthropod Management Tests 24(1): G36. https://doi.org/10.1093/amt/24.1.G36

[B520] Heller PR Walker R (1999b) Management of white grubs with applications of experimental CGA-293343 formulations, isofenphos, imidacloprid, and halofenozide, 1998. Arthropod Management Tests 24(1): G38. https://doi.org/10.1093/amt/24.1.G38

[B521] Heller PR Walker R (1999c) Curative evaluation with Mach-2 and Merit to suppress northern masked chafer grubs, 1998. Arthropod Management Tests 24(1): G64. https://doi.org/10.1093/amt/24.1.G64

[B522] Heller PR Walker R (1999d) Curative management of northern masked chafer grubs with Dylox 6.2 G, Mach-2 1.5G, and Merit 0.5G, 1998. Arthropod Management Tests 24(1): G79. https://doi.org/10.1093/amt/24.1.G79

[B523] Heller PR Walker R (2000a) Summer suppression of white grubs with experimental formulations of CGA-293343 in comparison with merit, 1999. Arthropod Management Tests 25(1): G67. https://doi.org/10.1093/amt/25.1.G67

[B524] Heller PR Walker R (2000b) Suppression of white grubs with applications of experimental CGA293343 formulations, imidacloprid, and halofenozide, 1999. Arthropod Management Tests 25(1): G32. https://doi.org/10.1093/amt/25.1.G32

[B525] Heller PR Walker R (2001a) Evaluating the effect of Mach-2 formulations and application timing on management of white grubs, 2000. Arthropod Management Tests 26(1): G20. https://doi.org/10.1093/amt/26.1.G20

[B526] Heller PR Walker R (2001b) Suppression of white grubs with applications of experimental CGA-293,343 formulations, imidacloprid, trichlorfon, and halofenozide, 2000. Arthropod Management Tests 26(1): G22. https://doi.org/10.1093/amt/26.1.G22

[B527] Heller PR Walker R (2001c) Timing study to suppress scarab white grub species, 2000. Arthropod Management Tests 26(1): G61. https://doi.org/10.1093/amt/26.1.G61

[B528] Heller PR Walker R (2001d) Suppression of white grubs with applications of experimental CGA293343 formulations, imidacloprid, and halofenozide, 2000. Arthropod Management Tests 26(1): G21. https://doi.org/10.1093/amt/26.1.G21

[B529] Heller PR Walker R (2002a) Early fall evaluation of registered and experimental formulations to curatively suppress white grubs, 2001. Arthropod Management Tests 27(1): G61. https://doi.org/10.1093/amt/27.1.G61

[B530] Heller PR Walker R (2002b) Preventive suppression of white grubs with applications of conventional and experimental formulations, 2001. Arthropod Management Tests 2(1): G64. https://doi.org/10.1093/amt/27.1.G64

[B531] Heller PR Walker R (2002c) Evaluation of registered formulations to curatively suppress white grubs, 2001. Arthropod Management Tests 27(1): G39. https://doi.org/10.1093/amt/27.1.G39

[B532] Heller PR Walker R (2002d) Curative suppression of white grubs with applications of conventional formulations, 2001. Arthropod Management Tests 27(1): G62. https://doi.org/10.1093/amt/27.1.G62

[B533] Heller PR Walker R (2002e) Preventive suppression of white grubs with applications of experimental and conventional formulations of imidacloprid, halofenozide, and permethrin, 2001. Arthropod Management Tests 27(1): G17. https://doi.org/10.1093/amt/27.1.G17

[B534] Heller PR Walker R (2002f) Suppression of white grubs with applications of experimental and conventional formulations, 2001. Arthropod Management Tests 27(1): G37.

[B535] Heller PR Walker R (2002g) Midsummer evaluation of registered and experimental formulations to suppress white grubs, 2001. Arthropod Management Tests 27(1): G63. https://doi.org/10.1093/amt/27.1.G63

[B536] Heller PR Walker R (2003a) Mid-late summer and early fall evaluation of registered and experimental formulations to suppress Japanese beetle and northern masked chafer grubs, 2002. Arthropod Management Tests 28(1): G12. https://doi.org/10.1093/amt/28.1.G12

[B537] Heller PR Walker R (2003b) Late summer evaluation of registered formulations to suppress white grubs, 2002. Arthropod Management Tests 28(1): G13. https://doi.org/10.1093/amt/28.1.G13

[B538] Heller PR Walker R (2003c) Application of Mach 2 and Merit formulations for preventive suppression of scarab grubs, 2002. Arthropod Management Tests 28(1): G14. https://doi.org/10.1093/amt/28.1.G14

[B539] Heller PR Walker R (2003d) Timing of applications of Mach 2 and Merit for suppression of white grubs, 2002. Arthropod Management Tests 28(1): G15. https://doi.org/10.1093/amt/28.1.G15

[B540] Heller PR Walker R (2003e) Application of insecticide formulations to preventively suppress white grubs with varying irrigation strategies, 2002. Arthropod Management Tests 28(1): G33. https://doi.org/10.1093/amt/28.1.G33

[B541] Heller PR Walker R (2003f) Application of insecticide formulations to preventively suppress white grubs, 2002. Arthropod Management Tests 28(1): G31. https://doi.org/10.1093/amt/28.1.G31

[B542] Heller PR Walker R (2003g) Curative suppression of white grubs with applications of insecticide formulations, 2002. Arthropod Management Tests 28(1): G32. https://doi.org/10.1093/amt/28.1.G32

[B543] Heller PR Walker R (2004a) Spring and mid-summer evaluations of experimental formulations to suppress Japanese beetle and northern masked chafer grubs, 2002. Arthropod Management Tests 29(1): G15. https://doi.org/10.1093/amt/29.1.G15

[B544] Heller PR Walker R (2004b) White grub suppression timing study with applications of Dylox and Merit formulations, 2002. Arthropod Management Tests 29(1): G16. https://doi.org/10.1093/amt/29.1.G16

[B545] Heller PR Walker R (2004c) Preventive management of white grubs with imidacloprid and curative management of white grubs with trichlorfon, 2002. Arthropod Management Tests 29 (1): G9. https://doi.org/10.1093/amt/29.1.G9

[B546] Heller PR Walker R (2005a) Late-July application of halofenozide and imidacloprid formulations to preventively suppress scarab grubs, 2003. Arthropod Management Tests 30(1): G10. https://doi.org/10.1093/amt/30.1.G10

[B547] Heller PR Walker R (2005b) Mid-June application of experimental formulations to preventively suppress northern masked chafer grubs, 2003. Arthropod Management Tests 30(1): G11. https://doi.org/10.1093/amt/30.1.G11

[B548] Heller PR Walker R (2005c) Mid-May application of halofenozide and imidacloprid formulations to preventively suppress scarab grubs, 2003. Arthropod Management Tests 30(1): G12. https://doi.org/10.1093/amt/30.1.G12

[B549] Heller PR Walker R (2005d) Application of insecticide formulations to preventively suppress white grubs with three post irrigation strategies, 2003. Arthropod Management Tests 30(1): G39. https://doi.org/10.1093/amt/30.1.G39

[B550] Heller PR Walker R (2005e) Late-fall application of carbaryl, imidacloprid, and trichorfon formulations to curatively suppress scarab white grubs, 2003. Arthropod Management Tests 30(1): G9.

[B551] Heller PR Walker R Rowan M (2000a) Formulation and timing effects of Mach-2 on mixed populations of white grubs, 1999. Arthropod Management Tests 25(1): G30. https://doi.org/10.1093/amt/25.1.G30

[B552] Heller PR Walker R Rowan M (2000b) Evaluating the effect of application timing on management of white grubs with experimental CGA293343, and Dylox, 1999. Arthropod Management Tests 25(1): G29. https://doi.org/10.1093/amt/25.1.G29

[B553] Henderson A (1984) Observations on pollination of *Cryosophila albida*. Principes 28: 120–126.

[B554] Heng-Moss TM Eickhoff TE Baxendale FP (2005) Use of Merit and competitive products for control of white grubs, 2004. Arthropod Management Tests 30(1): G13. https://doi.org/10.1093/amt/30.1.G13

[B555] Heng-Moss TM Sitz RD Baxendale FP Baxendale RW (2010) Evaluations of experimental granular formulations for control of southern masked chafers, 2009. Arthropod Management Tests 35(1): G12. https://doi.org/10.4182/amt.2010.G12

[B556] Henshaw S (1885) List of the Coleoptera of America, north of Mexico. American Entomological Society, Philadelphia, 161 pp.

[B557] Herbst JFW (1789) Natursystem aller bekannten in- und ausländlischen Insecten, als eine fortsezung der von Büffonschen Naturgeschichte. Nach dem System des Ritters von Linné und Fabricius zu bearbeiten angefangen von Carl Gustav Jablonsky und fortgesezt von Johann Friedrich Wilhelm Herbst. Der Käfer 2. Joachim Pauli, Berlin, 336 pp.

[B558] Herbst JFW (1790) Natursystem aller bekannten in- und ausländlischen fortsezung der von Büffonschen Naturgeschichte. Der Käfer 3. Joachim Pauli, Berlin, Germany, 325 pp.

[B559] Heyne A Taschenberg O (1907) Die exotischen Käfer in Wort und Bild. G. Reusche, Leipzig, 262 pp. [+ 39 pls.]

[B560] Hielkema AJ (2017) Some corrections and remarks regarding the nomenclature of Neotropical Athyreini, Passalini, Rutelini, Cyclocephalini, Dynastini and Oryctini (Coleoptera: Scarabaeoidea). Insecta Mundi 0561: 1–18.

[B561] Hirthe G Porembski S (2003) Pollination of *Nymphaea lotus* (Nymphaeaceae) by rhinoceros beetles and bees in the northeastern Ivory Coast. Plant Biology 5: 670–676. https://doi.org/10.1055/s-2003-44717

[B562] Höhne W (1921) Eine neue *Erioscelis* (Col. Dyn.). Deutsche Entomologische Zeitschrift 1921: 108–109.

[B563] Höhne W (1922a) Beitrag zur Kenntnis der Cyclocephaliden (Col., Dyn.). Deutsche Entomologische Zeitchrift 1922: 81–95.

[B564] Höhne W (1922b) *Paraspidolea helleri* n. sp. (Col. Dyn.). Deutsche Entomologische Zeitchrift 1922: 371–373. https://doi.org/10.1002/mmnd.48019220404

[B565] Höhne W (1922c) *Aspidolea (subg. Aspidolites) atricollis* n. sp. (Col. Dyn.). Deutsche Entomologische Zeitchrift 1922: 374–376.

[B566] Höhne W (1922d) *Ancognatha ustulata* Burm. n. subsp. ustulatoides (Col. Dyn.). Deutsche Entomologische Zeitchrift 1922: 373–374.

[B567] Höhne W (1923a) Neue Dynastiden (Col.). Deutsche Entomologische Zeitschrift 1923: 252–255. https://doi.org/10.1002/mmnd.48019230304

[B568] Höhne W (1923b) Neue Cyclocephalen (Col. Dyn.). Deutsche Entomologische Zeitschrift 1923: 345–373. https://doi.org/10.1002/mmnd.48019230402

[B569] Hope FW (1837) The Coleopterists’ Manual, Containg the Lamellicorn Insects of Linnaeus and Fabricius. H. G. Bohn, London, 121 pp.

[B570] Horn GH (1871) Descriptions of new Coleoptera of the United States, with notes on known species. Transactions of the American Entomological Society 3: 325–344.

[B571] Horn GH (1875) Synonymical notes and description of new species of North American Coleoptera. Transactions of the American Entomological Society 5: 126–162.

[B572] Horn GH (1894) The Coleoptera of Baja California. Proceedings of the California Academy of Sciences, Second Series 4: 302–449.

[B573] Howden HF (1955) Some new species and records of North American Scarabaeidae (Coleoptera). Proceedings of the Entomological Society of Washington 57: 257–264.

[B574] Howden HF (1970) Jamaican Scarabaeidae: Notes and descriptions (Coleoptera). The Canadian Entomologist 102: 1–15. https://doi.org/10.4039/Ent1021-1

[B575] Howden HF Campbell JM (1974) Observations on some Scarabaeoidea in the Colombian Sierra Nevada de Santa Marta. The Coleopterists Bulletin 28: 109–114.

[B576] Howden HF Endrődi S (1966) Five new species of *Cyclocephala* Latreille from North and Central America. Canadian Entomologist 98: 295–302. https://doi.org/10.4039/Ent98295-3

[B577] Huerta-Espino J (2005) La Fauna de la Reserva Biológica Indio Maiz. Unpublished Report, IBESo (Investigación sobre Biodiversidad, Ecología y Sociedad) Financiado por PASMA. Ministry of Foreign Affairs of Demark, Danida, Copenhagen, 33 pp.

[B578] Hussaini SS (2014) Potential of entomopathogenic nematodes in integrated pest management. In: Abrol DP (Ed.) Integrated Pest Management: Current concepts and ecological perspective. Elsevier Academic Press, San Diego, 193–223. https://doi.org/10.1016/B978-0-12-398529-3.00012-9

[B579] Illiger K (1802a) In: Olivier GA (Ed.) Entomologie, ou histoire naturelle des insectes, avec leurs caractères génériques et spécifiques, leur description, leur synonymie, et leur figure enluminée. Coléoptères. Tome Premier. Baudouin, Paris, sections paginated separately.

[B580] Illiger K (1802b) In: Olivier GA (Ed.) Entomologie oder Naturgeschichte der Insekten mit ihren Gattungs- und ArtMerkmalen, ihrer Beschreibung und Synonymie. Käfer. Zweiter Theil. Karl Reichard, Braunschweig, 266 pp. [+ 5 pls]

[B581] Illiger K (1805) II. Zusätze, Berichtigungen und Bemerkungen zu Fabricii Systema Eleutheratorum Tomus II. Magazin für Insektenkunde 4: 69–174.

[B582] Illiger K (1806) VI. Nachtrag zu den Zusätze, Bemerkungen und Berichtigungen zu Fabricii Systema Eleutheratorum. Magazin für Insektenkunde 5: 221–246.

[B583] Imhoff L (1856) Versuch einer Einführung in das Studium der Koleoptern. L. Imhoff [“auf Kosten des Verfassers”]. Schweighauser, Basel, xxxi + [2] + 118 + [2] + 272 + [25] pp. [+ 25 pls]

[B584] International Commission on Zoological Nomenclature (ICZN) (1964) International Code of Zoological Nomenclature asopted by the XV International Congress of Zoology. International Trust for Zoological Nomenclature, London, 176 pp.

[B585] Instituto Colombiano Agropecuario. Programa de Entomología. Bogota (Colombia) (ICA) (1977) Problemas en exportación de banano. Notas y Noticias Entomologicas July/August: 51.

[B586] Ivie MA Marske KA Foley IA Ivie LL (2008) Appendix 2. Species lists of the beetles, non-beetle hexapods and non-hexapod invertebrates of Montserrat. In: Young RP (Ed.) A biodiversity assessment of the Centre Hills, Montserrat. Durrell Conservation Monograph No. 1. Durrell Wildlife Conservation Trust, Jersey, 237–311.

[B587] Jackson TA Klein MG (2006) Scarabs as pests: a continuing problem. The Coleopterists Bulletin 60(sp. 5): 102–119.

[B588] Jacquelin du Val PNC (1857) Insectes. Ordre des coléoptères, Lin. In: Sagra R (Ed.) Histoire Physique, Politique, et Naturelle de l’île de Cuba, Volume 7. A Bertrand, Paris, 1–136.

[B589] Jacques HE (1951) How to know the beetles. WC Brown Co., Dubuque, 372 pp.

[B590] Jameson ML (1998) Phylogenetic analysis of the subtribe Rutelina and revision of the *Rutela* generic groups (Coleoptera: Scarabaeidae: Rutelinae: Rutelini). Bulletin of the University of Nebraska State Museum 14 (1997): 1–184.

[B591] Jameson ML Drumont A (2013) Aroid scarabs in the genus *Peltonotus* Burmeister (Coleoptera, Scarabaeidae, Dynastinae): key to species and new distributional data. ZooKeys 320: 63–95. https://doi.org/10.3897/zookeys.320.5352 10.3897/zookeys.320.5352 PMC3744152 23950684

[B592] Jameson ML Jákl S (2010) Synopsis of the aroid scarabs in the genus *Peltonotus* Burmeister (Scarabaeidae, Dynastinae, Cyclocephalini) from Sumatra and descriptions of a new species. ZooKeys 34: 141–152. https://doi.org/10.3897/zookeys.34.302

[B593] Jameson ML Wada K (2004) Revision of the genus *Peltonotus* Burmeister (Coleoptera: Scarabaeidae: Dynastinae) from Southeastern Asia. Zootaxa 502: 1–66. https://doi.org/10.11646/zootaxa.502.1.1

[B594] Jameson ML Wada K (2009) Five new species of *Peltonotus* Burmeister (Scarabaeidae: Dynastinae: Cyclocephalini) from Southeast Asia. Insecta Mundi 0102: 1–16.

[B595] Jameson ML Ratcliffe BC Malý V (2002) Review of the genus *Acrobolbia* with remarks on its classification and a key to the world genera of Cyclocephalini (Coleoptera: Scarabaeidae: Dynastinae). Folia Heyrovskyana 10: 1–15.

[B596] Jameson ML Oishi DR Ratcliffe BC McQuate GT (2009) Two additional invasive scarabaeoid beetles (Coleoptera: Scarabaeidae: Dynastinae) in Hawaii. Proceedings of the Hawaiian Entomological Society 41: 25–30.

[B597] Janssens A (1942) Dynastinae (Coleoptera Lamellicornia) Fam. Scarabaeidae. Exploration du Parc National Albert (Mission G. F. de Witte [1933-1935]) 38: 3–49 + 3 pls.

[B598] Jaramillo J Borgemeister C Ebssa L Gaigl A Tobón R Zimmermann G (2005) Effect of combined applications of *Metarhizium anisopliae* (Metsch.) Sorokin (Deuteromycotina: Hyphomycetes) strain CIAT 224 and different dosages of imidacloprid on the subterranean burrower bug *Cyrtomenus bergi* Froeschner (Hemiptera: Cydnidae). Biological Control 34: 12–20. https://doi.org/10.1016/j.biocontrol.2005.03.021

[B599] Jaynes HA Gardner TR (1924) Selective parasitism by *Tiphia* sp. Journal of Economic Entomology 17: 366–369. https://doi.org/10.1093/jee/17.3.366

[B600] Johnson JP (1941a) White grubs during 1941. Bulletin of the Connecticut Agricultural Experiment Station 461: 523–530.

[B601] Johnson JP (1941b) Cyclocephala (Ochrosidia) borealis in Connecticut. Journal of Agricultural Research 62: 79–86.

[B602] Johnson SA Nicolson SW (2001) Pollen digestion by flower-feeding Scarabaeidae: protea beetles (Cetoniini) and monkey beetles (Hopliini). Journal of Insect Physiology 47: 725–733. https://doi.org/10.1016/S0022-1910(00)00166-9 10.1016/s0022-1910(00)00166-9 11356419

[B603] Joly LJ (1995a) Nuevos registros y redescripcion de dos especies negras de *Cyclocephala* (Coleoptera, Melolonthidae, Dynastinae) de Venezuela. Boletín de Entomología Venezolana (N. S.) 10: 167–175.

[B604] Joly LJ (1995b) *Cyclocephala rangelana* Chapin y *Cyclocephala vidua* Endrődi, dos especies Antillanas estrechamente relacionadas (Coleoptera, Melolonthidae, Dynastinae, Cyclocephalini). Boletín de Entomología Venezolana (N. S.) 10: 57–67.

[B605] Joly LJ (1998) Una especie nueva de *Cyclocephala* de la República Dominicana y descripción del macho de *C. vinosa* Arrow 1937, de Jamaica (Coleoptera, Melolonthidae, Dynastinae, Cyclocephalini). Boletín de Entomología Venezolana (N. S.) 13: 45–55.

[B606] Joly LJ (2000a) A new species of *Cyclocephala* Latreille from the Venezuelan llanos (Coleoptera: Scarabaeidae: Dynastinae). The Coleopterists Bulletin 54: 333–338. https://doi.org/10.1649/0010-065X(2000)054[0333:ANSOCL]2.0.CO;2

[B607] Joly LJ (2000b) A new species of *Cyclocephala* from Venezuela related to *Cyclocephala castanea* (Olivier) and *C. hardyi* Endrődi (Coleoptera: Scarabaeidae: Dynastinae). The Coleopterists Bulletin 54: 520–531. https://doi.org/10.1649/0010-065X(2000)054[0520:ANSOCF]2.0.CO;2

[B608] Joly LJ (2003) *Cyclocephala minuchae*, nueva especie de Venezuela y redescripción de C. vincentiae Arrow (Coleoptera: Scarabaeidae: Dynastinae: Cyclocephalini). Entomotropica 18: 37–47.

[B609] Joly LJ (2005) Una nueva especie de *Cyclocephala* Latreille de la Amazonia Venezolana (Coleoptera, Scarabaeidae, Dynastinae, Cyclocephalini). Entomotropica 20: 1–5.

[B610] Joly LJ (2009) Review of the species in the *Cyclocephala bicolor* Laporte species group (Coleoptera: Scarabaeidae: Dynastinae). Zootaxa 2048: 47–64.

[B611] Joly LJ (2010) Una nueva especie de *Cyclocephala* Dejean de Perú, con la redescripción de *C. spilopyga* Erichson, 1847 y *C. pygidiata* Dupuis, 1999 (Coleoptera, Scarabaeidae, Dynastinae, Cyclocephalini). Entomotropica 25: 133–148.

[B612] Joly LJ Escalona HE (2002a) Revisión del género *Chalepides* Casey, 1915 (Coleoptera: Scarabaeidae: Dynastinae: Cyclocephalini). Entomotropica 17: 37–90.

[B613] Joly LJ Escalona HE (2002b) Dos nuevas especies de *Dyscinetus* Harold de Argentina (Coleoptera: Scarabaeidae: Dynastinae: Cyclocephalini). Entomotropica 17: 197–205.

[B614] Joly LJ Escalona HE (2010) El género *Dyscinetus* Harold (Coleoptera: Scarabaeidae: Dynastinae: Cyclocephalini) en Venezuela y la descripción de una nueva especie. Papéis Avulsos de Zoologia Museu de Zoologia da Universidade de São Paulo 50: 203–210.

[B615] Jordan TA Youngman RR Laub CL Tiwari S Balderson TK Moore DM Saphir M (2012) Fall soil sampling method for predicting spring infestation of white grubs (Coleoptera: Scarabaeidae) in corn and the benefits of clothianidin seed treatment in Virginia. Crop Protection 39: 57–62. https://doi.org/10.1016/j.cropro.2012.04.006

[B616] Kawanishi CY Splittstoesser CM Tashiro H Steinkraus KH (1974) *Ataenius spretulus*, a potentially important turf pest, and its associated milky disease bacterium. Environmental Entomology 3: 177–181. https://doi.org/10.1093/ee/3.1.177

[B617] Kaya HK Klein MG Burlando TM Harrison RE Lacey LA (1992) Prevalence of two *Bacillus popilliae* Dutky morphotypes and blue disease in *Cyclocephala hirta* LeConte (Coleoptera: Scarabaeidae) populations in California. The Pan-Pacific Entomologist 68: 38–45.

[B618] Kaya HK Klein MG Burlando TM (1993) Impact of *Bacillus popilliae*, *Rickettsiella popilliae* and entomopathogenic nematodes on a population of the scarabaeid, *Cyclocephala hirta* Biocontrol Science and Technology 3: 443–453. https://doi.org/10.1080/09583159309355299

[B619] Kaya HK Burlando TM Choo HY Thurston GS (1995) Integration of entomopathogenic nematodes with *Bacillus thuringiensis* or pesticidal soap for control of insect pests. Biological Control 5: 432–441. https://doi.org/10.1006/bcon.1995.1052

[B620] King IN (1914) The Coleoptera of Henry County, Iowa. Proceedings of the Iowa Academy of Science 21: 317–340.

[B621] King ABS (1984) Biology and identification of white grubs (*Phyllophaga*) of economic importance in Central America. Tropical Pest Management 30: 36–50. https://doi.org/10.1080/09670878409370850

[B622] Kirk VM (1970) A list of the beetles of South Carolina, Part 2 – Mountain, Piedmont, and southern Coastal Plain. Technical Bulletin 1038, South Carolina Agricultural Experiment Station, Clemson University: 1–117.

[B623] Kirk VM Balsbaugh Jr EU (1975) A list of the beetles of South Dakota. South Dakota State University Agricultural Experiment Station Technical Bulletin 42: 1–139.

[B624] Kirsch T (1870) Beiträge zur Käferfauna von Bogotà (Sechstes Stück). Berliner Entomologische Zeitschrift 14: 337–378.

[B625] Kirsch T (1873) Beiträge zur Kenntniss der Peruanischen Käferfauna auf Dr. Abendroth’s Sammlungen basirt (zweites Stück). Berliner Entomologische Zeitschrift 17: 339–418.

[B626] Kirsch T (1885) Neue südamerikanische Käfer (Drittes Stück). Nitidulidae, Pectinicornia und Lamellicornia Berliner Entomologische Zeitschrift 29: 207–224. https://doi.org/10.1002/mmnd.18850290206

[B627] Klein MG (1988) Pest management of soil-inhabiting insects with microorganisms. Agriculture, Ecosystems and Environment 24: 337–349. https://doi.org/10.1016/0167-8809(88)90077-1

[B628] Knoch AW (1801) Neue Beyträge zue Insectenkunde. Schwickert’s Publishing, Leipzig, 208 pp. [+ 9 pls]

[B629] Knuth PEOW Appel O Loew E (1904) Handbuch der Blütenbiologie, unter Zugrundelegung von Herman Müllers Werk: “Die Befruchtung der Blumen durch Insekten.” Volume 3. Wilhelm Engelmann, Leipzig, 570 pp.

[B630] Kolbe H (1907) Coleopteren. Ergebnisse der Hamburger Magalcaensischen Sammelreise 1892/93. II. Band. Arthropoden: 1–125. [+ 3 pls, sections paginated separately]

[B631] Koppenhöfer AM (2008) Integrated pest management of white grubs. In: Pessarakli M (Ed.) Handbook of turfgrass management and physiology. CRC Press, Boca Raton, 315–334.

[B632] Koppenhöfer AM Behile RW Dunlap CA Fisher J Laird C Vittum PJ (2008) Pellet formulations of sex pheromone components for mating disruption of oriental beetle (Coleoptera: Scarabaeidae) in turfgrass. Environmental Entomology 37: 1126–1135. https://doi.org/10.1093/ee/37.5.1126 10.1603/0046-225x(2008)37[1126:pfospc]2.0.co;2 19036191

[B633] Koppenhöfer AM Brown IM Gaugler R Grewal PS Kaya HK Klein MG (2000a) Synergism of entomopathogenic nematodes and imidacloprid against white grubs: greenhouse and field evaluation. Biological Control 19: 245–251. https://doi.org/10.1006/bcon.2000.0863

[B634] Koppenhöfer AM Choo HY Kaya HK Lee DW Gelernter WD (1999) Increased field and greenhouse efficacy against scarab grubs with a combination of an entomopathogenic nematode and *Bacillus thuringiensis* Biological Control 14: 37–44. https://doi.org/10.1006/bcon.1998.0663

[B635] Koppenhöfer AM Cowles RS Cowles EA Fuzy EM Kaya HK (2002a) Effect of neonicotinoid synergists on entomopathogenic nematode fitness. Entomologia Experimentalis et Applicata 106: 7–18. https://doi.org/10.1046/j.1570-7458.2003.00008.x

[B637] Koppenhöfer AM Cowles RS Cowles EA Fuzy EM Kaya HK (2002b) Comparison of neonicotinoid insecticides as synergists for entomopathogenic nematodes. Biological Control 24: 90–97. https://doi.org/10.1016/S1049-9644(02)00008-7

[B638] Koppenhöfer AM Fuzy EM (2003a) Effects of turfgrass endophytes (Clavicipitaceae: Ascomycetes) on white grub (Coleoptera: Scarabaeidae) control by the entomopathogenic nematode *Heterorhabditis bacteriophora* (Rhabditida: Heterorhabditidae). Environmental Entomology 32: 392–396. https://doi.org/10.1603/0046-225X-32.2.392

[B639] Koppenhöfer AM Fuzy EM (2003b) *Steinernema scarabaei* for the control of white grubs. Biological Control 28: 47–59. https://doi.org/10.1016/S1049-9644(03)00048-3

[B640] Koppenhöfer AM Fuzy EM (2003c) Ecological characterization of *Steinernema scarabaei*, a scarab-adapted entomopathogenic nematode from New Jersey. Journal of Invertebrate Pathology 83: 139–148. https://doi.org/10.1016/S0022-2011(03)00056-9 10.1016/s0022-2011(03)00056-9 12788283

[B641] Koppenhöfer AM Fuzy EM (2006) Effect of soil type on infectivity and persistence of the entomopathogenic nematodes *Steinernema scarabaei*, *Steinernema glaseri*, *Heterorhabditis zealandica*, and *Heterorhabditis bacteriophora* Journal of Invertebrate Pathology 92: 11–22. https://doi.org/10.1016/j.jip.2006.02.003 10.1016/j.jip.2006.02.003 16563427

[B642] Koppenhöfer AM Fuzy EM (2008a) Effect of the anthranilic diamide insecticide, chlorantraniliprole, on *Heterorhabditis bacteriophora* (Rhabditida: Heterorhabditidae) efficacy against white grubs (Coleoptera: Scarabaeidae). Biological Control 45: 93–102. https://doi.org/10.1016/j.biocontrol.2007.10.014

[B643] Koppenhöfer AM Fuzy EM (2008b) Attraction of four entomopathogenic nematodes to four white grub species. Journal of invertebrate pathology 99: 227–234. https://doi.org/10.1016/j.jip.2008.05.003 10.1016/j.jip.2008.05.003 18597774

[B644] Koppenhöfer AM Fuzy EM (2009) Long-term effects and persistence of *Steinernema scarabaei* applied for suppression of *Anomala orientalis* (Coleoptera: Scarabaeidae). Biological Control 48: 63–72. https://doi.org/10.1016/j.biocontrol.2008.09.005

[B645] Koppenhöfer AM Fuzy EM Crocker RL Gelernter WD Polavarapu S (2004) Pathogenicity of *Heterorhabditis bacteriophora*, *Steinernema glaseri*, and *S. scarabaei* (Rhabditida: Heterorhabditidae, Steinernematidae) against 12 white grub species (Coleoptera: Scarabaeidae). Biocontrol Science and Technology 14: 87–92. https://doi.org/10.1080/0958315031000151701

[B646] Koppenhöfer AM Grewal PS (2005) Compatibility and interactions with agrochemicals and other biocontrol agents. In: Grewal PS, Ehlers R-U, Shapiro-Ilan DI (Eds) Nematodes as biocontrol agents, CABI Publishing, Cambridge, 363–384. https://doi.org/10.1079/9780851990170.0363

[B647] Koppenhöfer AM Grewal PS Fuzy EM (2006) Virulence of the entomopathogenic nematodes *Heterorhabditis bacteriophora*, *Heterorhabditis zealandica*, and *Steinernema scarabaei* against five white grub species (Coleoptera: Scarabaeidae) of economic importance in turfgrass in North America. Biological Control 38: 397–404. https://doi.org/10.1016/j.biocontrol.2005.12.013

[B648] Koppenhöfer AM Grewal PS Fuzy EM (2007) Differences in penetration routes and establishment rates of four entomopathogenic nematode species into four white grub species. Journal of Invertebrate Pathology 94: 184–195. https://doi.org/10.1016/j.jip.2006.10.005 10.1016/j.jip.2006.10.005 17156793

[B649] Koppenhöfer AM Grewal PS Kaya HK (2000b) Synergism of imidacloprid and entomopathogenic nematodes against white grubs: the mechanism. Entomologia Experimentalis et Applicata 94: 283–293. https://doi.org/10.1046/j.1570-7458.2000.00630.x

[B650] Koppenhöfer AM Jackson TA Klein MG (2012) Bacteria for use against soil-inhabiting insects. In: Lacey LA (Ed.) Manual of techniques in invertebrate pathology. Second edition. Elsevier Academic Press, San Diego, 129–149. https://doi.org/10.1016/B978-0-12-386899-2.00005-1

[B651] Koppenhöfer AM Kaya HK (1996) Coexistence of two steinernematid nematode species (Rhabditida: Steinernematidae) in the presence of two host species. Applied Soil Ecology 4: 221–230. https://doi.org/10.1016/S0929-1393(96)00121-7

[B652] Koppenhöfer AM Kaya HK (1997) Additive and synergistic interaction between entomopathogenic nematodes and *Bacillus thuringiensis* for scarab grub control. Biological Control 8: 131–137. https://doi.org/10.1006/bcon.1996.0498

[B653] Koppenhöfer AM Kaya HK (1998) Synergism and imidacloprid and an entomopathogenic nematode: a novel approach to white grub (Coleoptera: Scarabaeidae) control in turfgrass. Journal of Economic Entomology 91: 618–623. https://doi.org/10.1093/jee/91.3.618

[B654] Koppenhöfer AM Latin R McGraw BA Brosnan JT Crow WT (2013) Integrated pest management. In: Stier JC, Horgan BP, Bonos SA (Eds) Turgrass: biology, use, and management. Agronomy Monograph 56. American Society of Agronomy, Crop Science Society of America, Soil Science Society of America, Madison, 933–1006. https://doi.org/10.2134/agronmonogr56.c25

[B655] Koppenhöfer AM Polavarapu S Fuzy EM Zhang A Ketner K Larsen T (2005) Mating disruption of oriental beetle (Coleoptera: Scarabaeidae) in turfgrass using microencapsulated formulations of sex pheromone components. Environmental Entomology 34: 1408–1417. https://doi.org/10.1603/0046-225X-34.6.1408 10.1603/0046-225x(2008)37[1126:pfospc]2.0.co;2 19036191

[B656] Koppenhöfer AM Wilson M Brown I Kaya HK Gaugler R (2000c) Biological control agents for white grubs (Coleoptera: Scarabaeidae) in anticipation of the establishment of the Japanese beetle in California. Journal of Economic Entomology 93: 71–80. https://doi.org/10.1603/0022-0493-93.1.71 10.1603/0022-0493-93.1.71 14658514

[B657] Koppenhöfer AM Wu S (2016) Microbial control of insect pests of turfgrass. In: Lacey LA (Ed.) Microbial control of insect and mite pests: from theory to practice. Elsevier Academic Press, San Diego, 331–341.

[B658] Krajcik M (2005) Dynastinae of the World Checklist (Coleoptera: Scarabaeidae: Dynastinae). Animma.X, Supplement No. 2, Pilsen, 122 pp.

[B659] Krajcik M (2012) Checklist of the world Scarabaeoidea. Animma.X Supplement 5, Pilsen, 278 pp.

[B660] Krell F-T Hirthe G Seine R Porembski S (2003) Rhinoceros beetles pollinate water lilies in Africa (Coleoptera: Scarabaeidae: Dynastinae; Magnoliidae: Nymphaeceae). Ecotropica 9: 103–106.

[B661] Kress WJ Beach JH (1994) Flowering plant reproductive systems. In: McDade L, Bawa KS, Hespenheide HA, Hartshorn GS (Eds) La Selva: Ecology and Natural History of a Neotropical Rainforest. The University of Chicago Press, Chicago, 161–182.

[B662] Krinsky WL (2009) Beetles (Coleoptera) In: Mullen GR, Durden LA (Eds) Medical and veterinary entomology. Second edition. Elsevier Academic Press, San Diego, 101–114.

[B663] Kriska NL Young DK (2005) New Wisconsin records for *Cyclocephala lurida* (Coleoptera: Scarabaeidae). The Great Lakes Entomologist 38: 201–202.

[B664] Küchmeister HA Webber C Silberbauer-Gottsberger I Gottsberger G (1998) A Polinização e sua relação com a termogênese em espécies de Arecaceae e Annonaceae da Amazônia central. Acta Amazonica 28: 217–245. https://doi.org/10.1590/1809-43921998283245

[B665] Kuldau G Bacon C (2008) Clavicipitaceous endophytes: Their ability to enhance resistance of grasses to multiple stresses. Biological Control 46: 57–71. https://doi.org/10.1016/j.biocontrol.2008.01.023

[B666] Kusui Y (1992) On a species of the genus *Chalepides* from the Kii Penisula. The Nanki Seibutu 34: 64–65.

[B667] Lachance M-A Starmer WT Rosa CA Bowles JM Stuart J Baker F Janzen DH (2001) Biogeography of the yeasts of ephemeral flowers and their insects. FEMS Yeast Research 1: 1–8. https://doi.org/10.1111/j.1567-1364.2001.tb00007.x 10.1111/j.1567-1364.2001.tb00007.x 12702457

[B668] Lachaume G (1992) Les Coléoptères du Monde, Vol. 14. Dynastidae Américains. Cyclocephalini – Agaocephalini – Pentodontini – Oryctini – Phileurini. Sciences Nat, Venette, 89 pp. [+ 16 pls]

[B669] Lacordaire JT (1856) Histoire Naturelle de Insectes. Genera des Coléoptères ou Exposé Méthodique et Critique de Tous les Genres Proposès Jusqu’ici dans cet Ordre d’Insectes. Contenant les Families de Pectinicornes et Lamellicornes, Volume 3. Roret, Paris, 594 pp.

[B670] Laporte [=Castelnau] FLNC de (1840) Histoire naturelle des insectes Coléoptères; avec une introduction renfermant l’anatomie et la physiologie des animaux articulés, par M. Brullé. Tome premier. Histoire naturelle des animaux articulés, annelides, crustacés, arachnides, myriapodes et insectes Tome troisieme. P. Duménil, Paris, 324 pp. [+ 19 pls]

[B671] Latreille PA (1829) Les crustacés, les arachnids et les insectes, distribués en familles naturelles, ouvrage formant les tomes 4 et 5 de celui de M. Le Baron Cuvier sur le Règne Animal (deuxième edition), tome premier. Déterville and Crochard, Paris, 584 pp.

[B672] Latreille PA (1837) The animal kingdom arranged according to its organization, serving as a foundation for the natural history of animals and an introduction to comparative anatomy. With figures designed after nature: the Crustacea, Arachnides, and Insecta Translated from the latest French edition. With additional notes, illustrated by nearly 800 coloured plates in four volumes. Vol. IV. Insecta-Zoophytes. G. Henderson, London, xxxii. [+ 139 pls]

[B673] LeConte JL (1854) Descriptions of new Coleoptera collected by Thos. H. Webb, M. D., in the years 1850–51 and 52, while Secretary to the U. S. and Mexican Boundary Commission. Proceedings of the Academy of Natural Sciences of Philadelphia 7: 220–225.

[B674] LeConte JL (1861) New species of Coleoptera inhabiting the Pacific district of the United States. Proceedings of the Academy of Natural Sciences of Philadelphia 13: 338–359.

[B675] LeConte JL (1862) Classification of the Coleoptera of North America. Prepared for the Smithsonian Institution. Smithsonian Miscellaneous Collections 3: i–xxv, 1–214.

[B676] LeConte JL (1863) New species of North American Coleoptera. Prepared for the Smithsonian Institution. Part 1. Smithsonian Miscellaneous Collections 167: 1–86.

[B677] LeConte JL (1866) Additions to the Coleopterous fauna of the United States. No 1. Proceedings of the Academy of Natural Sciences of Philadelphia 18: 361–394.

[B678] Lederer J (1864a) Bücher-Anzeigen [Teil a]. Wiener Entomologische Monatsschrift 8: 93–97.

[B679] Lederer J (1864b) Bücher-Anzeigen. Wiener Entomologische Monatsschrift 8: 58–64.

[B680] Leng CW (1920) Catalogue of the Coleoptera of America, north of Mexico. John D. Sherman Jr., Mount Vernon, 470 pp.

[B681] Leng CW Mutchler AJ (1914) A preliminary list of the Coleoptera of the West Indies as recorded to January 1, 1914. Bulletin of the American Museum of Natural History 33: 391–493.

[B682] Leng CW Mutchler AJ (1917) Supplement to the preliminary list of the Coleoptera of the West Indies. Bulletin of the American Museum of Natural History 37: 191–220.

[B683] Leng CW Mutchler AJ (1933) Second and third supplements 1925 to 1932 (inclusive) to catalogue of the Coleoptera of America, north of America. John D. Sherman Jr., Mount Vernon, 112 pp.

[B684] Leonard MD (1923) A list of the insects of New York. Cornell University Agricultural Experiment Station Memoir 101: 1–1121.

[B685] Lewis JD Bremer DJ Keeley SJ Fry JD (2012) Wilt-based irrigation in Kentucky bluegrass: effects on visual quality and irrigation amounts among cultivars. Crop Science 52: 1881–1890. https://doi.org/10.2135/cropsci2012.01.0033

[B686] Li H Bouwer G (2014) Evaluation of the synergistic activities of *Bacillus thuringiensis* Cry proteins against Helicoverpa armigera (Lepidoptera: Noctuidae). Journal of Invertebrate Pathology 121: 7–13. https://doi.org/10.1016/j.jip.2014.06.005 10.1016/j.jip.2014.06.005 24963598

[B687] Li X-Y Cowles RS Cowles EA Gaugler R Cox-Foster DL (2007) Relationship between the successful infection by entomopathogenic nematodes and the host immune response. International Journal for Parasitology 37: 365–374. https://doi.org/10.1016/j.ijpara.2006.08.009 10.1016/j.ijpara.2006.08.009 17275827

[B688] Li X-Y Cowles EA Cowles RS Gaugler R Cox-Foster DL (2009) Characterization of immunosuppressive surface coat proteins from *Steinernema glaseri* that selectively kill blood cells in susceptible hosts. Molecular and Biochemical Parasitology 165: 162–169. https://doi.org/10.1016/j.molbiopara.2009.02.001 10.1016/j.molbiopara.2009.02.001 19428663

[B689] Lima A da Costa (1953) Insetos do Brasil. 8.˚ Tomo. Capítulo XXIX. Coleópteros 2.^a^ Parte. Escola Nacional de Agronomia Série Didática N.˚ 10 – 1953, Rio de Janeiro, 323 pp.

[B690] Lima Nogueira GA de Rodrigues SR Tiago EF (2013) Biological aspects of *Cyclocephala tucumana* Brethes, 1904 and *Cyclocephala melanocephala* (Fabricius, 1775) (Coleoptera: Scarabaeidae). Biota Neotropica 13: 86–90. https://doi.org/10.1590/S1676-06032013000100009

[B691] Linnaeus C (1767) Systema naturae, Tom. 1. Pars II. Editio duodecima reformata. Impensis Direct. Laur. Salvii, Stockholm, 1364 pp.

[B692] Linsley EG (1960) Observations on some matinal bees at flowers of *Cucurbita*, *Ipomoea* and *Datura* in desert areas of New Mexico and southeastern Arizona. Journal of the New York Entomological Society 68: 13–20.

[B693] Lobo JM Morón MA (1993) La modificación de las comunidades de coleópteros Melolonthidae y Scarabaeidae en dos áreas protegidas mexicanas tras dos décadas de estudios faunísticos. Giornale Italiano de Entomologia 6: 391–406.

[B694] López Cristóbal U (1941) “Gusanos blancos” de las sementeras. Boletín de la Universidad Nacional de La Plata 6: 1–3.

[B695] López-García MM García-Atencia S Amat-García G (2015) Escarabajos fitófagos (Coleoptera: Scarabaeidae “Pleurosticti”) de los Andes orientales de Colombia (Departamentos de Santander, Boyacá y Cundinamarca). Boletín Científico Centro de Museos: Museo de Historia Natural 19: 322–358.

[B696] Lucas R (1896) Bericht über die wissenschaftlichen Leistungen im Gebiete der Entomologie während des Jahres 1895. Archiv für Naturgeschichte 62 (2, 2): 1–302.

[B697] Lucas R (1899) Bericht über die wissenschaftlichen Leistungen im Gebiete der Entomologie während des Jahres 1898. Archiv für Naturgeschichte 65 (2, 2): 1–912.

[B698] Lucas R (1918a) Catalogus alphabeticus generum et subgenerum Coleopterorum orbis terrarium totius (famil., trib., subtr., sect. inel.). Pars I. Archiv für Naturgeschichte, Abteilung A 84 (1): 1–128.

[B699] Lucas R (1918b) Catalogus alphabeticus generum et subgenerum Coleopterorum orbis terrarium totius. Pars I. Archiv für Naturgeschichte, Abteilung A 84 (4): 449–576.

[B700] Lucero Malfa AM Peña Villamil LA Cultid L (2006) Efecto de *Steinernema* sp. sobre larvas de *Ancognatha scarabaeoides* (Coleoptera: Scarabaeidae) en condiciones de laboratorio e invernadero. Revista Corpoica – Ciencia y Tecnología Agropecuaria 7: 66–69. https://doi.org/10.21930/rcta.vol7_num1_art:62

[B701] Luederwaldt H (1915) Insekten am Licht. Zeitschrift für wissenschaftliche Insektenbiologie 11: 304–309.

[B702] Luederwaldt H (1916) Biologische Notizen über brasilianische Coleopteren. Zeitschrift für wissenschaftliche Insektenbiologie 12: 293–298.

[B703] Luederwaldt H (1926) *Cyclocephala cribrata* Burm. (Lamellicornidae-Dynastinae), habitante legal das bromeliaceas. Revista do Museu Paulista 14: 129–132.

[B704] Luederwaldt H Pinto da Fonesca J (1922) A Ilha dos Alcatrazes. Revista do Museu Paulista 13: 440–486.

[B705] Lugo-García G Ortega-Arenas L González-Hernández H Aragón-García A Romero-Nápoles J Cortés RR. (2009) Descripción de las larvas de tercer instar de Melolonthidae (Coleoptera) asociadas al cultivo de Agave tequilana var. azul y su fluctuación poblacional en Jalisco, México. Neotropical Entomology 38: 769–780. https://doi.org/10.1590/S1519-566X2009000600010 10.1590/s1519-566x2009000600010 20098923

[B706] Lugo-García GA Morón MA Aragón A Ortega LD Reyes-Olivas A Sánchez BH (2013) Especies nocturnas de Scarabaeoidea (Coleoptera: Polyphaga) en el norte de Sinaloa, México. Revista Colombiana de Entomología 39: 95–104.

[B707] Lugo-García GA Ortega-Arenas LD Aragón-García A González-Hernández H Romero-Nápoles J Reyes-Olivas Á Morón MA (2012) Especies de gallina ciega (Coleoptera: Scarabaeoidea) asociadas al cultivo de maíz en Ahome, Sinaloa, México. Agrociencia 46: 307–320.

[B708] Luna JM Alvarez JA Villaseñor JI (2007) Colección de Coleoptera (Insecta). In: Castillo-Cerón JM, Luna JM (Eds) Colecciones del Centro de Investigaciones Biológicas. Universidad Autónoma del Estado de Hidalgo, Pachuca, 33–48.

[B709] Lunau K (2002) The evolution of flowering plants, flower visitors and interactions between them – a look at flower biology with Gerd von Wahlert. Bonner Zoologische Monographien 50: 109–136.

[B710] Machatschke JW (1972) Scarabaeoidea: Melolonthidae, Rutelinae. Coleopterorum Catalogous Supplementa 66 (1): 1–361.

[B711] Machatschke JW (1974) Scarabaeoidea: Melolonthidae, Rutelinae. Coleopterorum Catalogous Supplementa 66 (2): 363–429.

[B712] MacLean DB (1977) Seasonal and spatial variation of species diversity in collections of Scarabaeidae, Elateridae, and Cerambycidae from west central Indiana. Proceedings of the Indiana Academy of Science 87: 252–528.

[B713] MacLeay WS (1819) *Horae Entomologicae*: or essays on the annulose animals. volume 1, part 1. Containing: General observations on the geography, manners, and natural affi nities of the insects which compose the genus Scarabaeus of Linnaeus; To which are added a few incidental remarks on the genera Lucanus and Hister of the same author. With an appendix and plates. S. Bagster, London, 160 pp.

[B714] Maddock DE Fehn CF (1958) Human ear invasions by adult scarabaeoid beetles. Journal of Economic Entomology 51: 546–547. https://doi.org/10.1093/jee/51.4.546a

[B715] Maes J-M (1987) Catalogo de los Scarabaeidae (Coleoptera) de Nicaragua. Revista Nicaragüense de Entomología 1: 27–60.

[B716] Maes J-M (1992) Fauna entomologica del department de Zelaya, Nicaragua (segunda nota). Revista Nicaragüense de Entomología 19: 29–41.

[B717] Maes J-M (1994) Los Dynastinae (Coleoptera: Scarabaeidae) de Nicaragua. Revista Nicaragüense de Entomología 30: 1–44.

[B718] Maes J-M Ratcliffe BC (1996) Scarabaeidae nuevos para la fauna de Nicaragua. Revista Nicaragüense de Entomología 34: 17–18.

[B719] Maes J-M Ratcliffe BC Jameson ML (1997) Fauna entomologica de la Reserva Natural Bosawas, Nicaragua. XI. Escarabajos (Coleoptera: Scarabaeidae) nuevos para la fauna de Nicaragua. Revista Nicaragüense de Entomología 39: 41–45.

[B720] Maes J-M Tellez Robleto J (1988) Catalogo de los insectos y arthropodos terrestres asociados a las principales plantas de importancia economica en Nicaragua. Revista Nicaragüense de Entomología 5: 1–95.

[B721] Maia ACD Schlindwein C (2006) *Caladium bicolor* (Araceae) and *Cyclocephala celata* (Coleoptera, Dynastinae): A well-established pollination system in the northern Atlantic rainforest of Pernambuco, Brazil. Plant Biology 8: 529–534. https://doi.org/10.1055/s-2006-924045 10.1055/s-2006-924045 16906489

[B722] Maia ACD Schlindwein C Navarro DMAF Gibernau M (2010) Pollination of *Philodendron acutatum* (Araceae) in the Atlantic forest of northeastern Brazil: A single scarab beetle species guarantees high fruit set. International Journal of Plant Sciences 171: 740–748. https://doi.org/10.1086/654846

[B723] Maia ACD Dötterl S Kaiser R Silberbauer-Gottsberger I Teichert H Gibernau M Navarro DM do AF Schlindwein C Gottsberger G (2012) The key role of 4-methyl-5-vinythiazole in the attraction of scarab beetle pollinators: a unique olfactory floral signal shared by Annonaceae and Araceae. Journal of Chemical Ecology 38: 1072–1080. 10.1007/s10886-012-0173-z 22918609

[B724] Maia ACD Gibernau M Carvalho AT Gonçalves EG Schlindwein C (2013a) The cowl does not make the monk: scarab beetle pollination of the Neotropical aroid *Taccarum ulei* (Araceae: Spathicarpeae). Biological Journal of the Linnean Society 108: 22–34. https://doi.org/10.1111/j.1095-8312.2012.01985.x

[B725] Maia ACD Gibernau M Dötterl S Navarro DMAF Seifert K Müller T Schlindwein C (2013b) The floral scent of *Taccarum ulei* (Araceae): Attraction of scarab beetle pollinators to an unusual aliphatic acyloin. Phytochemistry 93: 71–78. https://doi.org/10.1016/j.phytochem.2013.03.005 10.1016/j.phytochem.2013.03.005 23582213

[B726] Maia ACD Lima CT de Navarro DM do AF Chartier M Giulietti AM Machado IC (2014) The floral scents of Nymphaea subg. Hydrocallis (Nymphaeaceae), the New World night-blooming water lilies, and their relation with putative pollinators. Phytochemistry 103: 67–75. https://doi.org/10.1016/j.phytochem.2014.04.007 10.1016/j.phytochem.2014.04.007 24814399

[B727] Maldonado M Sakuragui CM Trigo JR Rodrigues D (2015) The selective florivory of *Erioscelis emarginata* matches its role as a pollinator of *Philodendron* Entomologia Experimentalis et Applicata 156: 290–300. https://doi.org/10.1111/eea.12332

[B728] Malý V (2006) A new species of *Cyclocephala* from Central America and descriptive notes of two species of *Cyclocephala* from South America (Coleoptera, Scarabaeidae, Dynastinae). Les Cahiers Magellanes Hors-Série 22: 1–11.

[B729] Manetti PL Alvarez Castillo HA Carmona D López AN (1994) Test of Agrochemicals and Cultivars 15 (Annals of Applied Biology 124, Supplement): 12–14.

[B730] Manetti PL Alvarez Castillo HA Carmona DM López AN Vincini AM Huarte M (1996) Response of potato cultivars to natural infection by soil insects. Test of Agrochemicals and Cultivars 17 (Annals of Applied Biology 128, Supplement): 82–83. https://doi.org/10.1111/j.1744-7348.1996.tb07920.x

[B731] Mankin RW Brandhorst-Hubbard J Flanders KL Zhang M Crocker RL Lapointe SL McCoy CW Fisher JR Weaver DK (2000) Eavesdropping on insects hidden in soil and interior structures plants. Journal of Economic Entomology 93: 1173–1182. https://doi.org/10.1603/0022-0493-93.4.1173 10.1603/0022-0493-93.4.1173 10985028

[B732] Mannerheim CG (1829) Description de quarante nouvelles espèces de scarabéides du Brésil. Nouveaux Mémoires de la Société Impériale des Naturalistes de Moscou 1: 29–80.

[B733] Marschall AFG (1857) Personen-, Orts- und Sach-Register der fünf ersten Jahrgänge (1851–1855) der Sitzungsberichte und Abhandlungen des Wiener zoologisch-botanischen Vereines. Verhandlungen der Zoologisch-Botanischen Gesellschaft in Österreich (Index Volumes 1–5): 1–160.

[B734] Mariconi FAM (1959) Dois novos insetos em laranjeira. O Biológico 25: 244–249.

[B735] Marín Jarillo A (2001) Abundancia del complejo “gallina ciega” (Coleóptera: Melolonthidae) asociado al cultivo de maíz en el Centro de México. Agricultura Técnica en México 27: 119–131.

[B736] Marino P Villamizar L Espinel C Cotes AM (2004) Caracterización de prototipos de bioplaguicidas granulados a base de *Metarhizium anisopliae* para el control de *Ancognatha scarabaeoides* (Coleóptera: Melolonthidae). Revista Colombiana Entomología 30: 43–49.

[B737] Marquet J Roguet D (2003) Contribution à la connaissance des Coléoptères Scarabaéides de la Martinique. Le Coléoptèriste 6: 9–13.

[B738] Martínez A (1954) Notas coleopterologicas VI. Anales de la Sociedad Cientifica Argentina 157: 19–27.

[B739] Martínez A (1955) Un nuevo genero y especie de escarabeido dinastino (Col. Scarabaeidae, Dynastinae). Mitteilungen der Münchner Entomologischen Gesellschaft 44/45: 242–249.

[B740] Martínez A (1957) Scarabaeoidea Neotropica IV. Un nuevo Cyclocephalini (Col. Scarab. Dynastidae). Neotropica 3: 29–32.

[B741] Martínez A (1960a) Un nuevo género de Cyclocephalini (Col., Scarab., Dyasti.). Ciencia (Mexico) 20: 97–98.

[B742] Martínez A (1960b) Una nueva especie de *Eremophygus* (Col. Scarab. Rutelin.). Ciencia (Mexico) 20: 131–133.

[B743] Martínez A (1964) Scarabaeoidea Neotropica IX (Coleoptera). Una nueva especie de *Cyclocephala* Latreille. Neotropica 10: 87–94.

[B744] Martínez A (1965a) Notas Coleopterologicas X. Dos nuevas especies de Cyclocephalini Neotropicales (Dynastinae). Anales de la Sociedad Cientifica Argentina 179: 63–74.

[B745] Martínez A (1965b) Scarabaeoidea Neotropica X. *Paraclinidia*, nuevo subgénero de *Cyclocephala* (Col. Scarab. Dynastinae). Neotropica 11: 13–18.

[B746] Martínez A (1966) Algunos Dynastinae neotropicales nuevos o poco conocidos (Coleoptera). Neotropica 12: 72–80.

[B747] Martínez A (1967) Scarabaeoidea Neotropica XIII. Una nueva especie de *Cyclocephala* Latreille, 1829 (Scarabaeidae, Dynastinae). Neotropica 14: 127–131.

[B748] Martínez A (1968a) Notas sobre Cyclocephalini americanos con descripción de dos nuevas especies (Col. Scarab., Dynast.). Ciencia (Mexico) 26: 185–190.

[B749] Martínez A (1968b) Scarabaeoidea Neotropica XIV. Una nueva especie de *Cyclocephala* de Bolivia (Col. Scarabaeoidae, Dynastinae). Neotropica 14: 23–26.

[B750] Martínez A (1968c) Scarabaeoidea Neotropica XV. Una nueva especie de *Cyclocephala* de Brasil (Col. Scarabaeidae, Dynastinae). Neotropica 14: 81–84.

[B751] Martínez A (1969) Dos nuevas *Cyclocephala* mexicanas (Col. Scarab. Dynastinae). Acta Zoológica Mexicana 9: 1–8.

[B752] Martínez A (1975a) Una nueva especie de *Aspidolea* de Ecuador (Col. Scarabaeidae, Dynastinae). Entomologische Arbeiten aus dem Museum G. Frey Tutzing bei München 26: 307–313.

[B753] Martínez A (1975b) *Cyclocephala sudamericanas* nuevas o poco conocidas (Col. ScarabaeidaeDynastinae). Entomologische Arbeiten aus dem Museum G. Frey Tutzing bei München 26: 263–274.

[B754] Martínez A (1978a) Una nueva especie de “*Cyclocephala*” de Paraguay. Acta Científica, Serie Entomolgía 12: 5–8.

[B755] Martínez A (1978b) Algunos Cyclocephalini neotropicales nuevos (Col. Scarab. Dynastinae). Acta Científica, Serie Entomolgía 12: 8–19.

[B756] Martínez A Martínez A (1981) Una nueva especie de *Cyclocephala* de Venezuela (Col. Scarab. Dynastinae, Cyclocephalini). Revista de la Sociedad Entomológica Argentina 39: 203–206.

[B757] Martínez A Morón MA (1984) Una nueva especie de *Cyclocephala* Latreille de Venezuela (Coleoptera; Melolonthidae; Dynastinae). Folia Entomológica Mexicana 62: 47–57.

[B758] Martínez GA Crespo JE Castelo MK (2013) Influencia de la densidad de coespecíficos sobre la orientación y discriminación de hospedadores en *Mallophora ruficauda* (Diptera: Asilidae). Acta Zoológica Lilloana 57: 92–94.

[B759] Martínez-Morales I Morón MA (2013) Los sistemas reproductivos en hembras de Melolonthinae, Rutelinae, y Dynastinae (Coleoptera: Scarabaeoidea, Melolonthidae). Southwestern Entomologist 40: 369–385. https://doi.org/10.3958/059.040.0211

[B760] Marques OM Gil-Santana HR (2009) Dynastinae (Coleoptera, Scarabaeidae) em um agroecossistema da região sul da Bahia, Brasil. Revista Brasileira de Zoociências 11: 145–151.

[B761] Martorell LF (1939) Insects observed in the state of Aragua, Venezuela, South America. The Journal of Agriculture of the University of Puerto Rico 23: 177–232.

[B762] Martorell LF Salas AE (1939) Additional insect records from Venezuela. The Journal of Agriculture of the University of Puerto Rico 23: 233–255.

[B763] Mashtoly TA Abolmaaty A Thompson N El-Zemaity ME-S Hussien MI Alm SR (2010) Enhanced toxicity of *Bacillus thuringiensis japonensis* Strain Buibui toxin to oriental beetle and northern masked chafer (Coleoptera: Scarabaeidae) larvae with *Bacillus* sp. NFD2. Journal of Economic Entomology 103: 1547–1554. https://doi.org/10.1603/EC10028 10.1603/ec10028 21061952

[B764] Mashtoly TA El-Zemaity ME-S Hussien MI Alm SR (2009) LC and LD_50_ values of *Bacillus thuringiensis* Serovar *japonensis* Strain Buibui toxin to oriental beetle and northern masked chafer larvae (Coleoptera: Scarabaeidae). Journal of Economic Entomology 102: 1891–1895. https://doi.org/10.1603/029.102.0520 10.1603/029.102.0520 19886454

[B765] Mauco L Favero M (2004) Diet of the Common Tern (*Sterna hirundo*) during nonbreeding season in Mar Chiquita Lagoon, Buenos Aires, Argentina. Orntiologia Neotropical 15: 121–131.

[B766] McAtee WL (1940) An experiment in songbird management. The Auk 57: 333–348. https://doi.org/10.2307/4079000

[B767] McNamara J (1991) Family Scarabaeidae: scarab beetles. In: Bousquet Y (Ed.) Checklist of beetles of Canada and Alaska. Agriculture Canada, Ottawa, 145–158.

[B768] Melsheimer FE Haldeman SS LeConte JL (1853) Catalogue of the described Coleoptera of the United States. Smithsonian Institution, Washington, District of Columbia, 174 pp.

[B769] Méndez-Aguilar M de J Castro-Ramírez AE Barrantes RA Pacheco-Flores C Ramírez-Salinas C (2005) Eficacia de dos tipos de recolecta para registrar la diversidad de melolóntidos nocturnos (Coleoptera: Scarabaeoidea). Acta Zoológica Mexicana (n.s.) 21: 109–124.

[B770] Mendheim H (1953) Insekten als Zwischenwirte von Helminthen nebst einigen Bemerkungen über neue Zwischenwirte des Rattenbandwurms. Nachrichtenblatt der Bayerischen Entomologen 2: 69–70.

[B771] Merchant ME Crocker RL (1996) White grubs in Texas turfgrass. Texas A&M AgriLife Extension E-publication. (http://extentopubs.tamu.edu/e-211.html) [accessed 17 July 2017]

[B772] Metcalf CL Flint WP (1939) Destructive and useful insects: their habits and control. Second Edition. McGraw-Hill, New York, 981 pp.

[B773] Metcalf CL Flint WP Metcalf RL (1951) Destructive and useful insects: their habits and control. Third Edition. McGraw-Hill, New York, 1071 pp.

[B774] Meyer RW (1978) Insects and other arthropods of economic importance in Indiana during 1978. Proceedings of the Indiana Academy of Science 88: 194–199.

[B775] Meyer RW (1979) Insects and other arthropods of economic importance in Indiana during 1979. Proceedings of the Indiana Academy of Science 89: 210–214.

[B776] Meyer RW (1981) Insects and other arthropods of economic importance in Indiana during 1981. Proceedings of the Indiana Academy of Science 91: 292–297.

[B777] Milius S (2003) Warm-blooded plants? Ok, there’s no blood, but they do make their own heat. Science News 13 Dec. 2003: 379+. https://doi.org/10.2307/4019064

[B778] Miller JB (1870) White grubs in strawberry beds. The American Entomologist and Botanist 2(10): 307.

[B779] Miller TA (1985) Structure and physiology of the circulatory system. In: Kerkut GA, Gilbert LI (Eds) Comprehensive insect physiology, biochemistry, and pharmacology. Integument, respiration, and circulation. Pergamon Press, New York, 289–354. https://doi.org/10.1016/B978-0-08-030804-3.50014-5

[B780] Minor P Morón MA (2016) Coleópteros lamelicornios (Coleoptera: Scarabaeoidea) de la barranca de Huehuetitla, Tlaxcala, México. Acta Zoológica Mexicana (n.s.) 32: 310–322.

[B781] Miskimen GW Bond RM (1970) The insect fauna of St. Croix, United States Virigin Islands. The New York Academy of Sciences Scientific survey of Porto Rico and the Virgin Islands 13(1): 1–114.

[B782] Mitasuhashi J (2016) Edible insects of the world. CRC Press, Boca Raton, 296 pp. https://doi.org/10.1201/9781315367927

[B783] Miyake Y (2000) New or little known scarabaeid beetles from Southeast Asia IV (Insecta: Coleoptera: Scarabaeidae). Science Report of the Research Institute of Evolutionary Biology 9: 105–120.

[B784] Miyake Y Yamaya S (1994) Some new scarabaeid species from southern Asia preserved in the Nagaoka Municipal Science Museum (II). Bulletin of the Nagaoka Municipal Science Museum 29: 37–43.

[B785] Moffat AJ (1890) Rare captures. The Canadian Entomologist 22: 60. https://doi.org/10.4039/Ent2260-3

[B786] Mondaca J (2011) Primer registro de *Cyclocephala modesta* (Scarabaeidae: Dynastinae: Cyclocephalini) en Chile. Revista Chilena de Entomologia 36: 33–38.

[B787] Mondaca J (2016) A new, high-elevation species of the genus *Ancognatha* Erichson (Coleoptera: Scarabaeidae: Dynastinae) from Chile. The Coleopterists Bulletin 70: 59–64. https://doi.org/10.1649/072.070.0106

[B788] Mondino EA López AN Castillo HAA Carmona DM (1997) Ciclo de vida de *Cyclocephala signaticollis* Burmeister, 1847 (Coleoptera: Scarabaeidae: Dynastinae) y su relación con los factores ambientales. Elytron 11: 145–156.

[B789] Monte O (1933) Pragas e moléstias do Chá. Boletim de Agricultura, Zootecnia e Veterinaria 6: 597–600.

[B790] Montgomery BE Amos JM (1940) Contributions to a list of the Coleoptera of the Clark County State Forest. Proceedings of the Indiana Academy of Science 50: 251–258.

[B791] Montoya GC Madrigal-C A Ramírez CA (1994) Evaluación de trampas de luz para el control de adultos de Scarabaeidae (Coleoptera) en cultivos de papa en La Unión (Antioquia). Revista Colombiana de Entomología 20: 130–136.

[B792] Moore I (1937) A list of the beetles of San Diego County, California. San Diego Society of Natural History-Occasional Papers 2: 1–109. https://doi.org/10.5962/bhl.part.6209

[B793] Moore G (1958) Observaciones sobre ataques del escarabajo negro del trigo (*Dyscinetus gagates*) en plantaciones forestales. Revista Forestal Argentina 2: 90–92.

[B794] Moore MR (2012) A new female elytral character for the tribe Cyclocephalini (Coleoptera: Scarabaeidae: Dynastinae) and an observation of its possible function. The Coleopterists Bulletin 66: 200–2002. https://doi.org/10.1649/072.066.0303

[B795] Moore MR Beza-Beza CF Wickell DA Beck JB Jameson ML (2015) Molecules, morphology and *Mimeoma* scarabs: evolutionary and taxonomic implications for a palm-associated scarab group. Systematic Entomology 40: 891–900. https://doi.org/10.1111/syen.12139

[B796] Moore MR Jameson ML (2013) Floral associations of cyclocephaline scarab beetles. Journal of Insect Science 13 (100): 1–43. https://doi.org/10.1673/031.013.10001 10.1673/031.013.10001 PMC4062068 24738782

[B797] Moragues G (2010) *Stenocrates duplicatus* Endrödi, 1967, nouveau dynaste pour la faune guyanaise. Contribution á L’Étude des Coléoptères de Guyane 1: 9–10.

[B798] Mora-Aguilar EF Delgado L (2012) A new species of *Cyclocephala* Dejean (Coleoptera: Scarabaeidae: Dynastinae: Cyclocephalini) from the cloud forests of southeastern Mexico and descriptions of the female of *Cyclocephala berti* Delgado. The Coleopterists Bulletin 66: 139–142. https://doi.org/10.1649/072.066.0209

[B799] Morales-Rodriguez A Peck DC (2009) Synergies between biological and neonicotinoid insecticides for the curative control of the white grubs *Amphimallon majale* and *Popillia japonica* Biological Control 51: 169–180. https://doi.org/10.1016/j.biocontrol.2009.06.008

[B800] Mora-Urpí J (1982) Polinización en *Bactris gasipaes* H. B. K. (Palmae): Nota Adicional. Revista de Biología Tropical 30: 174–176.

[B801] Mora-Urpí J Solís EM (1980) Polinización en *Bactris gasipaes* H. B. K. (Palmae). Revista de Biología Tropical 28: 153–174.

[B802] Morelli E (1989) Clave para la identificación de las larvas de tres especies del género *Cyclocephala* presentes en Uruguay (Coleoptera, Dynastidae). European Association of Coleopterology. International Congress of Coleopterology, Abstracts Volume. Barcelona, Spain. September 18–23, 1989: 121.

[B803] Morelli E (1991) Descripción de la larva y de la pupa de *Cyclocephala signaticollis* Burmeister, 1847 (Coleoptera: Scarabaeidae: Dynastinae) y observaciones sobre su biología. Elytron 5 (supplement 1): 189–195.

[B804] Morelli E Alzugaray R (1994) Descripcion de la larva de *Cyclocephala testacea* Burmeister, 1847 y clave para la determinacion de larvas de cuatro especies del genero *Cyclocephala* en el Uruguay (Coleoptera, Dynastinae). Revista Brasileira de Biologia 54: 77–84.

[B805] Moritz C (1836) Notizen zur Fauna der Insel Puertorico. Archiv für Naturgeschichte 2: 373–392.

[B806] Morón MA (1977a) Descripción del macho de *Cyclocephala picta* Burm. 1847 (Coleoptera: Melolonthidae, Dynastinae). Anales del Instituto de Biología de la Universidad Nacional Autónoma de México (serie Zoología) 48: 133–140.

[B807] Morón MA (1977b) Redescripción de *Cyclocephala jalapensis* Casey (Coleoptera, Melolonthidae, Dynastinae). Folia Entomológica Mexicana 38: 13–16.

[B808] Morón MA (1979) Fauna de Coleópteros Lamelicornios de la estación de biología, “Los Tuxtlas”, Veracruz, Unam. México. Anales del Instituto de Biología de la Universidad Nacional Autónoma de México, Series Zoología 50: 375–454.

[B809] Morón MA (1981) Fauna de coleópteros Melolonthidae de la Reserva de la Biósfera “La Michilia”, Durango, México. Folia Entomológica Mexicana 50: 3–69.

[B810] Morón MA (1994) Fauna de Coleoptera Lamellicornia en las montañas del noreste de Hidalgo, México. Acta Zoológica Mexicana (n.s.) 63: 7–59.

[B811] Morón MA (1997) Notas sobre *Cyclocephala* Latreille (Coleoptera: Melolonthidae, Dynastinae) associadas con *Xanthosoma* Schott (Araceae) en Chiapas, México. Giornale Italiano di Entomologia 8: 399–407.

[B812] Morón MA Deloya C (1991) Los coleopteros lamelicornios de la Reserva de la Biósfera “La Michilia”, Durango, Mexico. Folia Entomológica Mexicana 81: 209–283.

[B813] Morón MA Deloya C Delgado-Castillo L (1988) Fauna de coleopteros Melolonthidae, Scarabaeidae y Trogidae de la region de Chamela, Jalisco, México. Folia Entomológica Mexicana 77: 313–378.

[B814] Morón MA Deloya C Ramírez-Campos A Hernández-Rodriquez S (1998) Fauna de Coleoptera Lamellicornia de la region de Tepic, Nayarit, Mexico. Acta Zoológica Mexicana (n. s.)75: 73–116.

[B815] Morón MA Zaragoza S (1976) Coleópteros Melolonthidae y Scarabaeidae de Vill de Allende, Estado de México. Anales del Instituto de Biología, Universidad Nacional Autónoma de México. Serie Zoología 47: 83–118.

[B816] Morón MA Márquez J (2012) Nuevos registros estatales y nacionales de escarabajos (Coleoptera: Scarabaeoidea) y comentarios sobre su distribution. Revista Mexicana de Biodiversidad 83: 698–711. https://doi.org/10.7550/rmb.28386

[B817] Morón MA Hernandez-Rodriguez S Ramirez-Campos A (1996) El complejo “gallina ciega” (Coleoptera: Melolonthidae) asociado con la caña de azucar en Nayarit, Mexico. Folia Entomológica Mexicana 98: 1–44.

[B818] Morón MA Lugo-García GA Aragón-García A (2014) Description of the third instar larvae of five species of *Cyclocephala* (Coleoptera, Melolonthidae, Dynastinae) from Mexico. Revista Brasileira de Entomologia 58: 219–228. https://doi.org/10.1590/S0085-56262014000300001

[B819] Morón MA Villalobos FJ Deloya C (1985) Fauna de coleopteros lamelicornios de Boca del Chajul, Chiapas, México. Folia Entomológica Mexicana 66: 57–118.

[B820] Morón-Ríos A Morón MA (2001) La fauna de Coleoptera Melolonthidae de la reserva de la biósfera “El Triunfo”, Chiapas, México. Acta Zoológica Mexicana (n.s.)84: 1–25.

[B821] Morrill WL (1979) Coloration and temporal flight activity of some scarabiids. Journal of the Georgia Entomological Society 14: 255–258.

[B822] Mottern JL Heinz KM Ode PJ (2004) Evaluating biological control of fire ants using phorid flies: effects on competitive interactions. Biological Control 30: 566–583. https://doi.org/10.1016/j.biocontrol.2004.02.006

[B823] Moss WW Funk RC (1965) Studies on the developmental chaetotaxy of *Dyscinetonyssus hystricosus* n. g., n. sp. (Acari: Mesostigmata: Laelaptoidea). Acarologia 7: 235–267.

[B824] Muegge MA Payne C Bader R (2000) Evaluation of Mach-2 and Merit for control of white grubs in turf, 1999. Arthropod Management Tests 25(1): G74. https://doi.org/10.1093/amt/25.1.G74

[B825] Muegge MA Quigg R (2002) Evaluation of Mach 2 and Merit for control of white grubs in turf, 2000. Arthropod Management Tests 27(1): G9. https://doi.org/10.1093/amt/27.1.G9

[B826] Múñoz-Hernández A Morón MA Aragón A (2008) Coleoptera Scarabaeoidea de la región de Teziutlán, Puebla, México. Acta Zoológica Mexicana (n.s.) 24(3): 55–78.

[B827] Muramoto R (2000) A new spesies [*sic*] of the ruteline genus *Peltonotus* (Coleoptera, Scarabaeidae) from southern Vietnam. Kogane 1: 9–11.

[B828] Murray A (1857) Descriptions of new Coleoptera from the western Andes and the neighbourhood of Quito. The Edinburgh New Philosophical Journal 5 (new series): 220–234.

[B829] Murray NA (1993) Revision of *Cymbopetalum* and *Porcelia* (Annonaceae). Systematic Botany Monographs 40: 1–3, 5–87, 89–121. https://doi.org/10.2307/25027830

[B830] Navarrete-Heredia JL Delgado L Fierros-López HE (2001) Coleoptera Scarabaeoidea de Jalisco, México. Dugesiana 8: 37–93.

[B831] Negrisoli AS Garcia MS Barbosa Negrisoli CRC Bernardi D da Silva A (2010) Efficacy of entomopathogenic nematodes (Nematoda: Rhabditida) and insecticide mixtures to control *Spodoptera frugiperda* (Smith, 1797) (Lepidoptera: Noctuidae) in corn crops. Crop Protection 29: 677–683. https://doi.org/10.1016/j.cropro.2010.02.002

[B832] Neiswander CR (1938a) The annial white grub. Ohio Agricultural Experiment Station Bulletin 592: 50–51.

[B833] Neiswander CR (1938b) The annual white grub, *Ochrosidia villosa* Burm., in Ohio lawns. Journal of Economic Entomology 31: 340–344. https://doi.org/10.1093/jee/31.3.340

[B834] Neiswander CR (1951) Duration of the effectiveness of lead arsenate applied to turf for white grub control. Journal of Economic Entomology 44: 221–224. https://doi.org/10.1093/jee/44.2.221

[B835] Neita-Moreno JC (2011) Escarabajos (Coleoptera: Scarabaeoidea) del departamento del Chocó, Colombia. Revista Biodiversidad Neotropical 1: 17–27. https://doi.org/10.18636/bioneotropical.v1i1.25

[B836] Neita-Moreno JC Orozco-Araujo J Ratcliffe BC (2006) Escarabajos (Scarabaeidae: Pleurosticti) de la selva baja del bosque pluvial tropical “BP-T”, Chocó, Colombia. Acta Zoológica Mexicana (n. s.) 22: 1–32.

[B837] Neita-Moreno JC Ratcliffe BC Calberto G (2007) Immature stages of *Aspidolea singularis* (Coleoptera: Scarabaeidae: Cyclocephalini). Revista Colombiana de Entomología 33: 178–182.

[B838] Neita-Moreno JC Gaigl A (2008) Escarabajos de Importancia Agrícola en Colombia (Coleoptera: Scarabaeidae “Pleurosticti”). Produmedios. Universidad Nacional de Colombia, Bogotá, Colombia, 161 p.

[B839] Neita-Moreno JC Morón MA (2008) Estados immaduros de *Ancognatha ustulata* (Coleoptera: Melolonthidae: Cyclocephalini). Revista Mexicana de Biodiversidad 79: 355–361.

[B840] Neita-Moreno JC Yepes F (2011) Descripción de larva y pupa de *Dyscinetus dubius* (Coleoptera: Melolonthidae: Dynastinae: Cyclocephalini). Revista Colombiana de Entomología 37: 152–156.

[B841] Núñez-Avellaneda LA (2014) Patrones de asociación entre insectos polinizadores y palmas silvestres en Colombia con énfasis en palmas de importancia económica. PhD thesis, Bogotá, Colombia: Universidad Nacional de Colombia, Facultad de Ciencias.

[B842] Núñez-Avellaneda LA Neita-Moreno JC (2009) Rol de los escarabajos Cyclocephalini (Dynastinae: Scarabaeidae) en la polinización de palmas silvestres en Colombia. In: Hernández-Ortiz V, Deloya C, Castillo PR (Eds) Memorias VIII Reuníon Latinoamericana de Escarabaeidología (Coleoptera: Scarabaeoidea). Instituto de Ecología, Xalapa, 16–17.

[B843] Núñez-Avellaneda LA Rojas-Robles R (2008) Biología reproductiva y ecología de la polinización de la palma milpesos *Oenocarpus bataua* en los Andes Colombianos. Caldasia 30: 101–125.

[B844] Nussembaum AL Lecuona RE (2012) Selection of *Beauveria bassiana* sensu lato and *Metarhizium anisopliae* sensu lato isolates as microbial control agents against the boll weevil (*Anthonomus grandis*) in Argentina. Journal of Invertebrate Pathology 110: 1–7. https://doi.org/10.1016/j.jip.2012.01.010 10.1016/j.jip.2012.01.010 22326392

[B845] Oestergaard J Belau C Strauch O Ester A van Rozen K Ehlers R-U (2006) Biological control of *Tipula paludosa* (Diptera: Nematocera) using entomopathogenic nematodes (*Steinernema* spp.) and Bacillus thuringiensis subsp. israelensis Biological Control 39: 525–531. https://doi.org/10.1016/j.biocontrol.2006.07.003

[B846] Ogloblin AA (1941) Los insectos polenizadores de *Philodendron* en la gobernacion de Misiones*. Revista Argentina de Zoogeografia 1: 33–39.

[B847] Ohaus F (1900) Bericht über eine entomologische Reise nach Centralbrasilien. Entomologische Zeitung 61: 193–274.

[B848] Ohaus F (1909) Berichte über eine Entomologische Studienreise in Südamerika. Entomologische Zeitung 70: 3–139.

[B849] Ohaus F (1910) Neue südamerikanische Dynastiden. (Col.). Deutsche Entomologische Zeitschrift 1910: 671–690. https://doi.org/10.1002/mmnd.4801910606

[B850] Ohaus F (1911) Neue Coleoptera lamellicornia aus Argentinien. Deutsche Entomologische Zeitschrift 7: 553–565.

[B851] Ohaus F (1912) Beiträge zur Kenntnis der Ruteliden. X. Stettiner Entomologische Zeitung 73: 273–319.

[B852] Ohaus F (1918) Scarabaeidae: Euchirinae, Phaenomerinae, Rutelinae. Coleopterorum Catalogus 20: 1–241.

[B853] Ohaus F (1929) Beitrag sur Kenntnis der Rutelinen (Col. lamell.). Deutsche Entomologische Zeitschrift 1929: 385–406.

[B854] Ohaus F (1934a) XXVII. Beitrag zur Kenntnis der Ruteliden (Col. Scarabaeidae). Mitteilungen der Deutschen Entomologischen Gesellschaft 5: 9–15.

[B855] Ohaus F (1934b) Coleoptera lamellicornia, Fam. Scarabaeidae, Subfam. Rutelinae T.1: Tribus Rutelini. Genera Insectorum Fasc. 199A: 1–172.

[B856] Oliveira HN de Ávila CJ (2011) Ocorréncia de *Cyclocephala forsteri* em *Acronomia aculeata* Pesquisa Agropecuária Tropical 41: 293–295. https://doi.org/10.5216/pat.v41i2.8769

[B857] Olivier GA (1789) Entomologie, ou Histoire Naturelle des Insectes, avec Leurs Caractères Génériques et Spécifi ques, leurs Description, leur Synonymie, et leur Figure Enluminée. Coléoptères, volume 1. Baudouin, Paris, 476 pp. (genera paginated separately). https://doi.org/10.5962/bhl.title.61905

[B858] Onore G (1997) A brief note on edible insects in Ecuador. Ecology of Food and Nutrition 36: 277–285. https://doi.org/10.1080/03670244.1997.9991520

[B859] Onore G (2005) Edible insects in Ecuador. In: Paoletti MG (Ed.) Ecological Implications of Minilivestock: potential of insects, rodents, frogs, and snails. Science publishers INC., Enfield, 343–352.

[B860] Otavo SE Parrado-Rosselli Á Noriega JA (2013) Superfamilia Scarabaeoidea (Insecta: Coleoptera) como elemento bioindicator de perturbación antropogénica en un parque nacional amazónico. Revista de Biología Tropical 61: 735–752. https://doi.org/10.15517/rbt.v61i2.11219 23885586

[B861] Otoya IA (1945) Anotaciones sobre el género *Ancognatha* y descripción de una nueva especie (Scarabaeidae). Caldasia 3: 273–292.

[B862] Pacheco-F C Castro Ramírez AE Morón MA Gómez y Gómez B (2008) Fauna de escarabajos melolóntidos (Coleoptera: Scarabaeoidea) en el municipio de Villaflores, Chiapas, Mexico. Acta Zoológica Mexicana (n. s.) 24: 139–168.

[B863] Pacheco-F C Deloya C Cortés-G P (2006) Phytophagous scarab beetles from the Central Region of Guerrero, Mexico (Coleoptera: Scarabaeidae: Melolonthinae, Rutelinae, Dynastinae, Cetoniinae). Revista Colombiana de Entomología 32: 191–199.

[B864] Padin SB Bello GM dal Vasicek AL (1996) Potencial bioinsecticida de hongos entomopatógenos de plagas en granos almacenados. Revista de la Facultad de Agronomia 15: 1–7.

[B865] Palacios-Rios M Rico-Gray V Fuentes E (1990) Inventario preliminar de los Coleoptera Lamellicornia de la zona de Yaxchilan, Chiapas, México. Folia Entomológica Mexicana 78: 49–60.

[B866] Pardo-Locarno LC (2013) Escarabajos (Coleoptera: Melolonthidae) del plan aluvial del Río Cauca, Colombia I. Ensamblaje, fichas bioecológicas, extinciones locales y clave para adultos. Dugesiana 20: 1–15.

[B867] Pardo-Locarno LC Franco Cruz MP Alarcón Gaviria AA (1995) Estudios preliminares de las chisas (Coleoptera: Lamellicornia) de San Antonio, Cauca. Registros y observaciones en Laprosticti y Pleurosticti. Revista Colombiana de Entomología 21: 51–57.

[B868] Pardo-Locarno LC Montoya-Lerma J van Schoonhoven A (2003) Abundancia de chisas rizófagas (Coleoptera: Melolonthidae) en agroecosistemas de Caldono y Buenos Aires, Cauca, Colombia. Revista Colombiana de Entomología 29: 177–183.

[B869] Pardo-Locarno LC Montoya-Lerma J Bellotti AC van Schoonhoven A (2005a) Structure and composition of the white grub complex (Coleoptera: Scarabaeidae) in agroecological systems of northern Cauca, Colombia. Florida Entomologist 88: 355–363. https://doi.org/10.1653/0015-4040(2005)88[355:SACOTW]2.0.CO;2

[B870] Pardo-Locarno LC Montoya-Lerma J van Schoonhoven A Morón MA (2005b) Riqueza del complejo chisa (Coleoptera: Melolonthidae) en cuatro agroecosistemas del Cauca, Colombia. Acta Agronómica 54(4): 1–11.

[B871] Pardo-Locarno LC Gonzalez R Montoya-Lerma J (2006) Description of a new species and new country records of *Ancognatha* Erichson (Coleoptera: Scarabaeidae: Dynastinae) from Colombia. Zootaxa 1139: 63–68.

[B872] Pardo-Locarno LC Arroyo JE Quiñónez F (2008) Observaciones de los escarabajos copronecrófagos y sapromelífagos de San Luis Robles, Nariño. Boletín Científico Centro de Museos: Meseo de Historia Natural 8: 113–139.

[B873] Pardo-Locarno LC Ramírez-Pava B Villota H Villanueva O Bahamón W (2011) Ensamblaje de escarabajos Melolonthidae (Coleoptera: Scarabaeoidea) asociados con pasturas en el departamento del Caquetá y su posible relación con la salubridad edáfica. Acta Agronómica 60(3): 1–12.

[B874] Park Y-L Tak J-H (2016) Essential oils for arthropod pest management in agricultural production systems. In: Preedy VR (Ed.) Essential oils in food preservation, flavor and safety. Elsevier Academic Press, San Diego, 61–70. https://doi.org/10.1016/B978-0-12-416641-7.00006-7

[B875] Parker RD Cosper JC Fromme DD (1999) Efficacy of soil insecticides on white grubs and Mexican corn rootworm, 1998. Arthropod Management Tests 24(1): F33. https://doi.org/10.1093/amt/24.1.F33

[B876] Passoa S (1983) Lista de los insectos asociados con los granos básicos y otros cultivos selectivos en Honduras. Ceiba 25(1): 7–97.

[B877] Patiño LMS (2004) Reconocimiento de especies del complejo chisa (Coleoptera-Melolonthidae) asociados a los cultivos de yuca y pasto en el municipio de Pereira y Alrededores. Masters thesis, Manizales, Colombia: Universidad de Caldas.

[B878] Paulian R (1947) Scarabaeoidea. In: Fleutiaux E, Legros C, Lepesme P, Paulian R (Eds) Faune de l’Empire Français 7. Coléoptères de Antilles, Volume 1. Libraire Larose, Paris, 17–84.

[B879] Paulian R (1954) Coléoptères Dynastides, Chironides et Dynamopides de l’Afrique noire française. Bulletin de l’Institute Française d’Afrique Noire (Série A, Sciences Naturelles) 16: 1119–1221.

[B880] Paulian R (1958) Coléoptères scarabéides de l’Indochine (rutélines et cétonines). Annales de la Société Entomologique de France 127: 73–105.

[B881] Paulino-Neto HF (2014) Polinização e biologia reprodutiva de araticum-liso (*Annona coriacea* Mart.: Annonaceae) em uma área de Cerrado paulista: Implicações para fruticulture. Revista Brasileira de Fruticultura 36(special edition): 132–140.

[B882] Pava-O J Castillo-C E González-O A Patiño-C H (1983) Aspectos de interes fitosanitario de la palma de chontaduro *Bactris gasipaes* H. B. K. en algunas regiones del Valle y Chocó. Acta Agronómica 33: 25–35.

[B883] Peck DC (2009) Long-term effects of imidacloprid on the abundance of surface- and soil-active nontarget fauna in turf. Agricultural and Forest Entomology 11: 405–419. https://doi.org/10.1111/j.1461-9563.2009.00454.x

[B884] Peck SB (2009) The beetles of St. Lucia, Lesser Antilles (Insecta: Coleoptera); diversity and distributions. Insecta Mundi 0106: 1–34.

[B885] Peck SB (2010) The beetles of the island of St. Vincent, Lesser Antilles (Insecta: Coleoptera); diversity and distributions. Insecta Mundi 144: 1–77.

[B886] Peck SB (2016) The beetles of the Lesser Antilles (Insecta, Coleoptera): diversity and distributions. Insecta Mundi 460: 1–360.

[B887] Peck SB Cook J Hardy Jr JD (2002) Beetle fauna of the island of Tobago, Trinidad and Tobago, West Indies. Insecta Mundi 16: 9–23.

[B888] Peck SB Thomas MC (1998) A distributional checklist of the beetles (Coleoptera) of Florida. Arthropods of Florida and Neighboring Land Areas 16: i-viii, 1–180.

[B889] Pellmyr O (1985) *Cyclocephala*: visitor and probable pollinator of *Caladium bicolor* (Araceae). Acta Amazonica 15: 269–272. https://doi.org/10.1590/1809-43921985152272

[B890] Perera OP Snodgrass GL Allen KC Jackson RE Becnel JJ O’Leary PF Luttrell RG (2012) The complete genome sequence of a single-stranded RNA virus from the tarnished plant bug, *Lygus lineolaris* (Palisot de Beauvois). Journal of Invertebrate Pathology 109: 11–19. https://doi.org/10.1016/j.jip.2011.08.004 10.1016/j.jip.2011.08.004 21939663

[B891] Pereira J Schlindwein C Antonini Y Maia ACD Dötterl S Martins C Navarro DMDAF Oliveira R (2014) *Philodendron adamantium* (Araceae) lures its single cyclocephaline pollinator with specific scent volatiles. Biological Journal of the Linnean Society 111: 679–691. https://doi.org/10.1111/bij.12232

[B892] Pérez Dominguez JF (1991) Fluctuacion estacional de poblaciones de adultos de gallina ciega (Coleoptera: Melolonthidae) en el centro de Jalisco, Mexico. Agrociencia. Serie Proteccion Vegetal 2: 27–41.

[B893] Perkins BD (1974) Arthropods that stress waterhyacinth. Pest Articles and News Summaries 20: 304–314. https://doi.org/10.1080/09670877409411855

[B894] Peterson A (1951) Larvae of insects. Part 2. Coleoptera, Diptera, Neuroptera, Siphonaptera, Mecoptera, Trichoptera. Edwards Bros., Ann Arbor, 416 pp.

[B895] Pfeiffer DG Axtell RC (1980) Coleoptera of poultry manure in caged-layer houses in North Carolina. Environmental Entomology 9: 21–28. https://doi.org/10.1093/ee/9.1.21

[B896] Phillips WJ Fox H (1924) The rough-headed corn stalk-beetle. United States Department of Agriculture Department Bulletin 1267: 1–34. https://doi.org/10.5962/bhl.title.64800

[B897] Pierson LM Schaeffer SK Stamm MD Heng-Moss TM (2008) Application timing of Meridian for control of bluegrass billbugs and southern masked chafers, 2007. Arthropod Management Tests 33(1): G44. https://doi.org/10.1093/amt/33.1.G44

[B898] Pike KS Rivers RL Mayo ZB (1977) Geographical distributions of the known *Phyllophaga* and *Cyclocephala* species in the North Central States. University of Nebraska Agricultural Experiment Station Miscellaneous Publications 34: 1–13.

[B899] Pike KS River RL Ratcliffe BC Oseto CY Mayo ZB (1976) A world bibliography of the genus *Cyclocephala* (Coleoptera: Scarabaeidae). Miscellaneous Publication of the University of Nebraska Agricultural Experiment Station 32: 1–32.

[B900] Pinto R Zanuncio Jr JS Zanuncio TV Zanuncio JC Lacerda MC (2004) Coleópteros coletados com armadilhas luminosas em plantio de *Eucalyptus urophylla* na região amazônica Brasileira. Ciência Florestal 14: 111–119. https://doi.org/10.5902/198050981787

[B901] Polivka JB (1950) A new insecticide to control turf insects. Journal of Economic Entomology 43: 391–392. https://doi.org/10.1093/jee/43.3.391

[B902] Polivka JB (1952) Control of the northern masked chafer with aldrin and dieldrin. Journal of Economic Entomology 45: 347–348. https://doi.org/10.1093/jee/45.2.347

[B903] Polivka JB (1959) The biology and control of turf grubs. Ohio Agricultural Experiment Station Research Bulletin 829: 1–30.

[B904] Polivka JB (1960) Grub population in turf varies with pH levels in Ohio soils. Journal of Economic Entomology 53: 860–863. https://doi.org/10.1093/jee/53.5.860

[B905] Polivka JB (1965) Effectivness of insecticides for the control of white grubs in turf. Ohio Agricultural Research and Development Center Research Circular 140: 1–7.

[B906] Ponchel Y (2006) The Dynastidae of the world. Biologie et collecte de quelques dynastides. Available online: http://dynastidae.voila.net/biologie.html. Accessed: 5 September 2015.

[B907] Ponchel Y (2009) Deux nouvelles espèces de Cyclocephalini de Guyane française (Coleoptera Dynastidae). L’Entomologiste 65: 183–185.

[B908] Ponchel Y (2010) Note sur *Cyclocephala virgo* Dechambre, 1999 et mise au point sur trois espèces de Dynastidae récemment décrites de Guyane (Coleoptera Dynastidae). L’Entomologiste 66: 171–172.

[B909] Ponchel Y (2011) Liste actualisée des Dynastinae de Guyane (Coleoptera, Scarabaeidae). Contribution á L’Étude des Coléoptères de Guyane 4: 60–61.

[B910] Ponchel Y (2015) Note sur les Dynastinae floricoles de Guyane (Coleoptera, Scarabaeidae). Contribution à l’étude des coléoptères de Guyane 9: 36–38. [Supplément au Bulletin de liaison d’ACOREP-France “Le Coléoptériste”].

[B911] Ponchel Y Dechambre R-P (2003) Deux nouveaux Cyclocephalini néotropicaux (Coleoptera, Dynastidae). Coléoptères 9: 267–270.

[B912] Poole RW Gentili P (1996) Nomina Insecta Nearctica. Volume 1: Coleoptera, Strepsiptera. Entomological Information Services, Rockville, 827 pp.

[B913] Popay AJ (2009) Insect pests. In: Fribourg HA, Hannaway DB, West CP (Eds) Tall Fescue for the Twenty-first Century. Agronomy Monograph 53. American Society of Agronomy, Crop Science Society of America, Soil Science Society of America, Madison, 129–149. https://doi.org/10.2134/agronmonogr53.c9

[B914] Popenoe EA (1876) A list of Kansas Coleoptera Transactions of the Kansas Academy of Science 5: 21–40. https://doi.org/10.2307/3623498

[B915] Popenoe EA (1881) A sketch of the beetle fauna of Kansas. Second Biennial Report of the State Board of Agriculture, to the Legislature of the State of Kansas for the years 1879–1880: 476–489.

[B916] Posada Ochoa L (1989) Lista de insectos dañinos y otras plagas en Colombia. Cuarta Edicion. ICA Boletin Tecnico 43: i–xix, 1–662.

[B917] Potascheff C de M de (2010) Ecologia da polinização de *Eschweilera nana* Miers., uma Lecythidaceae do Cerrado. PhD thesis, São Paulo, Brazil: Universidade Estadual Paulista, “Júlio de Mesquita Filho”, Instituto de Biociências-Rio Claro.

[B918] Potascheff C de M de Mori SA Lombardi JA (2014) Pollination ecology of the Cerrado species *Eschweilera nana* (Lecythidaceae subfam. Lecythidoideae). Brittonia 66: 191–206.

[B919] Potter DA (1980) Flight activity and sex attraction of northern and southern masked chafers in Kentucky turfgrass. Annals of the Entomological Society of America 73: 414–417. https://doi.org/10.1093/aesa/73.4.414

[B920] Potter DA (1981) Seasonal emergence and flight of northern and southern masked chafers in relation to air and soil temperature and rainfall patterns. Environmental Entomology 10: 793–797. https://doi.org/10.1093/ee/10.5.793

[B921] Potter DA (1982) Influence of feeding by grubs of the southern masked chafer on quality and yield of Kentucky bluegrass. Journal of Economic Entomology 75: 21–24. https://doi.org/10.1093/jee/75.1.21

[B922] Potter DA (1983) Effect of soil moisture on oviposition, water absorption, and survival of southern masked chafer (Coleoptera: Scarabaeidae) eggs. Environmental Entomology 12: 1223–1227. https://doi.org/10.1093/ee/12.4.1223

[B923] Potter DA (1995) Masked chafers. In: Brandenburg RL, Villani MG (Eds) Handbook of turfgrass insect pests. Entomological Society of America, Lanham, 70–72.

[B924] Potter DA (1998) Destructive turfgrass insects: biology, diagnosis, and control. Ann Arbor Press, Chelsea, 424 pp.

[B925] Potter DA Gordon FC (1984) Susceptibility of *Cyclocephala immaculata* (Coleoptera: Scarabaeidae) eggs and immatures to heat and drought in turf grass. Environmental Entomology 13: 794–799. https://doi.org/10.1093/ee/13.3.794

[B926] Potter DA Haynes KF (1993) Field-testing pheromone traps for predicting masked chafer (Coleoptera: Scarabaeidae) grub density in golf course turf and home lawns. Journal of Entomological Science 28: 205–212. https://doi.org/10.18474/0749-8004-28.2.205

[B927] Potter DA Patterson CG Redmond CT (1992) Influence of turfgrass species and tall fescue endophyte on feeding ecology of japenese beetle and southern masked chafer grubs (Coleoptera: Scarabaeidae). Journal of Economic Entomology 85: 900–909. https://doi.org/10.1093/jee/85.3.900

[B928] Potter DA Powell AJ Spicer PG Williams DW (1996) Cultural practices affect root-feeding white grubs (Coleoptera: Scarabaeidae) in turfgrass. Journal of Economic Entomology 89: 156–164. https://doi.org/10.1093/jee/89.1.156

[B929] Potter DA Williamson RC Haynes KF Powell Jr AJ (2000) Cultural control, risk assessment, and environmentally responsible management of scarab grubs and cutworms in turfgrass. In: Clark JM, Kenna MP (Eds) Fate and Management of Turfgrass Chemicals. ACS Symposium Series 743. American Chemical Society, Washington, District of Columbia, 383–396.

[B930] Power KT An R Grewal PS (2009) Effectiveness of *Heterohabditis bacteriophora* strain GPS11 applications targeted against different instars of the Japanese beetle *Popillia japonica* Biological Control 48: 232–236. https://doi.org/10.1016/j.biocontrol.2008.10.014

[B931] Prance GT (1976) The pollination and androphore structure of some Amazonian Lecythidaceae Biotropica 8: 235–241. https://doi.org/10.2307/2989715

[B932] Prance GT (1980) A note on the pollination of *Nymphaea amazonum* Mart. and Zucc. (Nymphaeaceae). Brittonia 32: 505–507. https://doi.org/10.2307/2806159

[B933] Prance GT Arias JR (1975) A study of the floral biology of *Victoria amazonica* (Poepp.) Sowerby (Nymphaeaceae). Acta Amazonica 5: 109–139. https://doi.org/10.1590/1809-43921975052109

[B934] Prance GT Anderson AB (1976) Studies of the floral biology of Neotropical Nymphaeaceae 3. Acta Amazonica 6: 163–170. https://doi.org/10.1590/1809-43921976062163

[B935] Prell H (1914) Beiträge zur Kenntnis der Dynastinen X (Col.). Entomologische Mitteilungen 3: 197–226. https://doi.org/10.5962/bhl.part.5084

[B936] Prell H (1934) Beiträge zur Kenntnis der Dynastinen (XII). Beschreibungen und Bemerkungen. Entomologische Zeitschrift 47: 162–164.

[B937] Prell H (1936) Beiträge zur Kenntnis der Dynastinen. Über die Homonymieverhältnisse der Namen von Gattungen und Untergattungen. Entomologische Blätter für Biologie und Systematik der Käfer 32: 147–150.

[B938] Prell H (1937a) Beiträge zur Kenntnis der Dynastinen (XVI) (Col.). Die Dynastinen der Fabriciusschen Sammlung im Zoologisch Museum der Universität Kiel. Deutsche Entomologische Zeitschrift 1936: 179–190.

[B939] Prell H (1937b) Beiträge zur Kenntnis der Dynastinen (XV, 1). Neue Arten und Rassen. Entomologische Zeitschrift 50: 495–496.

[B940] Prell H (1937c) Beiträge zur Kenntnis der Dynastinen (XV, 2). Neue Arten und Rassen. Entomologische Zeitschrift 51: 8–10.

[B941] Price JF Kring JB (1991) *Dyscinetus morator* (Coleoptera: Scarabaeidae) flight activity, food plant acceptance, damage and control in *Caladium* Florida Entomologist 74: 415–421. https://doi.org/10.2307/3494835

[B942] Prokofiev AM (2012) New and noteworthy pleurostict scarab beetles (Coleoptera: Scarabaeidae). Calodema 220: 1–33.

[B943] Prokofiev AM (2013) New synonyms in Dynastinae (Coleoptera: Scarabaeidae). Actual Problems of Modern Science 1: 131. [In Russian]

[B944] Prokofiev AM (2014) New and noteworthy scarab beetles from Asia and America (Coleoptera Lamerllicornia). Calodema 330: 1–25.

[B945] Prudhomme M (1906) Catalogue des coléoptères de la Guyane Française recuillis par M. Prudhomme de 1870 a 1906. Imprimerie du Gouvernement, Cayenne, 46 pp.

[B946] Puker A Rodrigues SR Tiago EF dos Santos WT (2009) Espécies de Scarabaeidae fitófagos (Insecta: Coleoptera) associadas ao sistema radicular de *Acronomia aculeata* (Jacq.) Lodd. Ex Mart. (Arecaceae). Biota Neotropica 9: 105–109. https://doi.org/10.1590/S1676-06032009000300009

[B947] Pye JD (2010) The distribution of circularly polarized light reflection in the Scarabaeoidea (Coleoptera). Biological Journal of the Linnean Society 100: 585–596. https://doi.org/10.1111/j.1095-8312.2010.01449.x

[B948] Quensel C (1806) In: Schönherr CJ (Ed.) Synonymia Insectorum, oder Versuch einer Synonymie aller bisher bekannten Insecten; nach Fabricii Systema Eleutheratorum etc. geordnet, volume 1, part 1. Heinr. A. Nordström, Stockholm, 293 pp.

[B949] Raguso RA Henzel C Buchmann SL Nabhan GP (2003) Trumpet flowers of the Sonoran Desert: floral biology of *Peniocereus* cacti and sacred *Datura* International Journal of Plant Sciences 164: 877–892. https://doi.org/10.1086/378539

[B950] Raid RN Cherry RH (1992) Pathogenicity of Metarhizium anisopliae var. major (Metschnikoff) Sorokin to a sugarcane grub *Ligyrus subtropicus* (Blatchley) (Coleoptera: Scarabaeidae). Journal of Agricultural Entomology 9: 11–16.

[B951] Ramírez LC Alonso CP (2016) Two late Pleistocene members of the white-grub complex, one of the most destructive insect pests of turfgrass. Revista Brasileira de Paleontologia 19: 531–536. https://doi.org/10.4072/rbp.2016.3.16

[B952] Ramírez N (1992) Especificidad de los sistemas de polinizacion en una comunidad arbustiva de la Guyana Venezolana. Ecotropicos 5: 1–19. https://doi.org/10.1111/j.1095-8339.1992.tb00294.x

[B953] Ramirez N Brito Y (1992) Pollination biology in a palm swamp community in the Venezuelan central plains. Botanical Journal of the Linnean Society 110: 277–302.

[B954] Ramírez-Ponce A Allende-Canseco J Morón MA (2009) Fauna de coleópteros lamelicornios de Santiago Xiacui, Sierra Norte, Oaxaca, México. Acta Zoológica Mexicana (n.s.)25: 323–343.

[B955] Ramírez-Salinas C Morón MA Castro-Ramírez AE (2004) Descripción de los estados inmaduros de tres especies de *Anomala*, *Ancognatha* y *Ligyrus* (Coleoptera: Melolonthidae: Rutelinae y Dynastinae) con observaciones de su biología. Acta Zoológica Mexicana (n.s.)20: 67–82.

[B956] Ramm CM Fithian RA Prochaska TJ Baxendale FP (2010) Evaluation of Aloft effectiveness in discouraging forager behavior, and curative control of southern masked chafer, 2009. Arthropod Management Tests 35(1): G13. https://doi.org/10.4182/amt.2010.G13

[B957] Ramos-Elorduy J Pino Moreno JM (2002) Edible insects of Chiapas, Mexico. Ecology of Food and Nutrition 41: 271–299. https://doi.org/10.1080/03670240214081

[B958] Ramos-Elorduy J Pino Moreno JM (2004) Los Coleoptera comestibles de México. Anales del Instituto de Biología, Universidad Nacional Autónoma de México, Serie Zoología 75: 149–183.

[B959] Ranger CM Reding ME Oliver JB Moyseenko JJ Youssef N (2009) Toxicity of botanical formulations to nursery-infesting white grubs (Coleoptera: Scarabaeidae). Journal of Economic Entomology 102: 304–308. https://doi.org/10.1603/029.102.0140 10.1603/029.102.0140 19253649

[B960] Ranger CM Reding ME Oliver JB Moyseenko JJ Youssef N Krause CR (2013) Acute toxicity of plant essential oils to scarab larvae (Coleoptera: Scarabaeidae) and their analysis by gas chromatography-mass spectrometry. Journal of Economic Entomology 106: 159–167. https://doi.org/10.1603/EC12319 10.1603/ec12319 23448028

[B961] Ratcliffe BC (1977) Four new species of Neotropical Cyclocephalini (Coleoptera: Scarabaeidae). Acta Amazonica 7: 429–434. https://doi.org/10.1590/1809-43921977073429

[B962] Ratcliffe BC (1978) New species of *Stenocrates* from Brazil (Coleoptera: Scarabaeidae). Acta Amazonica 8: 489–496. https://doi.org/10.1590/1809-43921978083489

[B963] Ratcliffe BC (1981) New species and distribution records of *Surutu* from Amazonian Brazil (Coleoptera: Scarabaeidae: Dynastinae). The Coleopterists Bulletin 35: 107–112.

[B964] Ratcliffe BC (1985) Key to the New World Genera of Adult Cyclocephalini http://www-museum.unl.edu/research/entomology/Guide/Scarabaeoidea/Scarabaeidae/Dynastinae/Dynastinae-Tribes/Cyclocephalini/Cyclocephalini-Key/CyclocephaliniK.html. Accessed 29 August 2015.

[B965] Ratcliffe BC (1986) Two new species of *Dyscinetus* from the West Indies and South America (Coleoptera: Scarabaeidae: Dynastinae). The Coleopterists Bulletin 40: 75–80.

[B966] Ratcliffe BC (1989) Corrections and clarifications to Endrődi’s “The Dynastinae of the World” (Coleoptera: Scarabaeidae). The Coleopterists Bulletin 43: 275–278.

[B967] Ratcliffe BC (1991) The scarab beetles of Nebraska. Bulletin of the University of Nebraska State Museum 12: 1–333.

[B968] Ratcliffe BC (1992a) Nine new species and 11 country records of *Cyclocephala* (Coleoptera: Scarabaeidae: Dynastinae) from Panama and Costa Rica. The Coleopterists Bulletin 46: 216–235.

[B969] Ratcliffe BC (1992b) New species and country records of Brazilian *Cyclocephala* (Coleoptera: Scarabaeidae: Dynastinae). Tijdschrift voor Entomologie 135: 179–190.

[B970] Ratcliffe BC (1992c) Two new species of *Cyclocephala* from Arizona and Mexico and a note on melanistic *C. melanocephala* (Coleoptera: Scarabaeidae: Dynastinae). The Coleopterists Bulletin 46: 250–255.

[B971] Ratcliffe BC (1992d) A new species of *Ancognatha* from Panama (Coleoptera: Scarabaeidae: Dynastinae). The Coleopterists Bulletin 46: 256–259.

[B972] Ratcliffe BC (2002a) A checklist of the Scarabaeoidea (Coleoptera) of Panama. Zootaxa 32: 1–48. https://doi.org/10.11646/zootaxa.32.1.1

[B973] Ratcliffe BC (2002b) Dynastinae MacLeay 1819. In: Arnett RH, Thomas MC, Skelley PE, Frank JH (Eds) American beetles. Volume 2. Polyphaga: Scarabaeoidea through Curculionoidea. CRC Press, Boca Raton, 64–67.

[B974] Ratcliffe BC (2003) The dynastine scarab beetles of Costa Rica and Panama (Coleoptera: Scarabaeidae: Dynastinae). Bulletin of the University of Nebraska State Museum 16: 1–506.

[B975] Ratcliffe BC (2008) More new species of *Cyclocephala* Dejean, 1821 from South America (Scarabaeidae: Dynastinae: Cyclocephalini). The Coleopterists Bulletin 62: 221–241. https://doi.org/10.1649/1066.1

[B976] Ratcliffe BC (2014) A new genus and species of Dynastinae (Coleoptera: Scarabaeidae) from the Galápagos Islands, Ecuador, other new species of Cyclocephalini, Pentodontini, and Phileurini from South America, and a revised key to the genera of New World Pentodontini The Coleopterists Bulletin 68: 663–680. https://doi.org/10.1649/0010-065X-68.4.663

[B977] Ratcliffe BC (2015) A revised catalog of the species *Stenocrates* Burmeister (Coleoptera: Scarabaeidae: Dynastinae: Cyclocephalini), with descriptions of three new species from Peru and Brazil and *Stenocrates inpai* Ratcliffe, 1978 placed in junior synonymy with *Stenocrates popei* Endrődi, 1971. The Coleopterists Bulletin 69: 773–779. https://doi.org/10.1649/0010-065X-69.4.773

[B978] Ratcliffe BC Cave RD (2002) New species of *Cyclocephala* from Honduras and El Salvador (Coleoptera: Scarabaeidae: Dynastinae: Cyclocephalini). The Coleopterists Bulletin 56: 152–157. https://doi.org/10.1649/0010-065X(2002)056[0152:NSOCFH]2.0.CO;2

[B979] Ratcliffe BC Cave RD (2006) The dynastine scarab beetles of Honduras, Nicaragua and El Salvador (Coleoptera: Scarabaeidae: Dynastinae). Bulletin of the University of Nebraska State Museum 21: 1–424.

[B980] Ratcliffe BC Cave RD (2008) The Dynastinae (Coleoptera: Scarabaeidae) of the Bahamas with a description of a new species of *Cyclocephala* from Great Inagua Island. Insecta Mundi 0024: 1–10.

[B981] Ratcliffe BC Cave RD (2009) New species of *Cyclocephala* Dejean, 1821 from Guatemala (Scarabaeidae: Dynastinae: Cyclocephalini). The Coleopterists Bulletin 63: 325–332. https://doi.org/10.1649/1171.1

[B982] Ratcliffe BC Cave RD (2010) The Dynastinae (Coleoptera: Scarabaeidae) of the Cayman Islands (West Indies), with descriptions of *Tomarus adoceteus*, new species (Pentodontini) and *Caymania nitidissima*, new genus and species (Phileurini). Insecta Mundi 0139: 1–16.

[B983] Ratcliffe BC Cave RD (2015) The dynastine scarab beetles of the West Indies (Coleoptera: Scarabaeidae: Dynastinae). Bulletin of the University of Nebraska State Museum 28: 1–346.

[B984] Ratcliffe BC Cave RD (2017) The dynastine scarab beetles of the United States and Canada (Coleoptera: Scarabaeidae: Dynastinae). Bulletin of the University of Nebraska State Museum 30: 1–298.

[B985] Ratcliffe BC Cave RD Cano EB (2013) The dynastine scarab beetles of Mexico, Guatemala, and Belize (Coleoptera: Scarabaeidae: Dynastinae). Bulletin of the University of Nebraska State Museum 27: 1–666.

[B986] Ratcliffe BC Delgado-Castillo L (1990) New species and notes of *Cyclocephala* from Mexico (Coleoptera: Scarabaeidae: Dynastinae). Folia Entomológica Mexicana 80: 41–57.

[B987] Ratcliffe BC Hoffman RL (2011) *Cyclocephala nigricollis* Burmeister (Coleoptera: Scarabaeidae: Dynastinae: Cyclocephalini). What is it? and where is it? The Coleopterists Bulletin 65: 135–138. https://doi.org/10.1649/072.065.0207

[B988] Ratcliffe BC Morón MA (1997) Dynastinae In: Morón MA, Ratcliffe BC, Deloya C (Eds) Atlas de los Escarabajos de México. Coleoptera: Lamellicornia Volume 1. Familia Melolonthidae. Comisión Nacional para el Conocimiento y Uso de la Biodiversidad (CONABIO) and Sociedad Mexicana de Entomologia, México, Distrito Federal, 53–98.

[B989] Ratcliffe BC Paulsen MJ (2008) The scarabaeoid beetles of Nebraska. Bulletin of the University of Nebraska State Museum 22: 1–570.

[B990] Ratcliffe BC Jameson ML Figueroa L Cave RD Paulsen MJ Cano EB Beza-Beza C Jimenez-Ferbans L Reyes-Castillo P (2015) Beetles (Coleoptera) of Peru: A survey of families. Scarabaeoidea The Journal of the Kansas Entomogical Society 88: 186–207. https://doi.org/10.2317/kent-88-02-186-207.1

[B991] Rebek EJ (2013) Timing trial for white grub control in bermudagrass with preventative insecticides, 2012. Arthropod Management Tests 38(1): G6. https://doi.org/10.4182/amt.2013.G6

[B992] Rebek EJ Royer TA Walker NR (2009a) Evaluation of early and late applications of Acelepryn (chlorantraniliprole) for control of white grubs in bermudagrass turf in Oklahoma, 2008. Arthropod Management Tests 34(1): G4. https://doi.org/10.4182/amt.2009.G4

[B993] Rebek EJ Royer TA Walker NR (2009b) Evaluation of preventative and curative applications of Arena (clothianidin) for control of white grubs in bermudagrass, 2008. Arthropod Management Tests 34(1): G5. https://doi.org/10.4182/amt.2009.G5

[B994] Reboredo GR Camino NB (2000) Two new *Rhabditida* species (Nematoda: Rhabditidae) parasites of *Cyclocephala signaticollis* (Coleoptera: Scarabaeidae) in Argentina. Journal of Parasitology 86: 819–821. https://doi.org/10.1645/0022-3395(2000)086[0819:TNRSNR]2.0.CO;2 10.1645/0022-3395(2000)086[0819:TNRSNR]2.0.CO;2 10958463

[B995] Redmond CT Potter DA (2010) Incidence of turf-damaging white grubs (Coleoptera: Scarabaeidae) and associated pathogens and parasitoids on Kentucky golf courses. Environmental Entomology 39: 1838–1847. https://doi.org/10.1603/EN10172 10.1603/EN10172 22182549

[B996] Redmond CT Umeda K Potter DA (2012a) Masked chafers. In: Brandenburg RL, Freeman CA (Eds) Handbook of turfgrass insect pests, 2nd ed. Entomological Society of America, Lanham, 53–55.

[B997] Redmond CT Williams DM Potter DA (2012b) Comparison of scarab grub populations and associated pathogens and parasitoids in warm- or cool-season grasses used on transitional zone golf courses. Journal of Economic Entomology 105: 1320–1328. https://doi.org/10.1603/EC12027 10.1603/ec12027 22928312

[B998] Redtenbacher L (1868) Coleopteren. Reise der Österreichischen Fregatte Novara um die Erde in den Jahren 1857, 1858, 1859 unter den Befehlen des Commodore B. von Wüllerstorf-Urbair. Zoologischer Theil. Zweiter Band. 1. Abtheilung A. Kaiserlich-Königlichen Hof- und Staatsdruckerei, Wien, 249 pp. [+ 5 pls]

[B999] Reiche ML (1859) Notes synonymiques sur le cinquième volume de l’Handbuch der Entomologie, par M. H. Burmeister, Berlin, 1840. Coléoptères Lamellicornes, Xylophiles. Annales de la Sociétè Entomologique de France 3(7): 5–19.

[B1000] Reinert JA (1979) Response of white grubs infesting Bermudagrass to insecticides. Journal of Economic Entomology 72: 546–548. https://doi.org/10.1093/jee/72.4.546

[B1001] Remedi de Gavotto A (1964) Ciclo biológico de *Cyclocephala signaticollis* Burm. (Col. Scarabaeidae) y caracteres específicos de su larva. Revista de Investigaciones Agropecuarias (Argentina) 5: 151–161.

[B1002] Remillet M Silvain JF Tavakilian G (1982) L’entomofaune des graminees fourrageres en Guyane Française. Caribbean Food Crops Society, Eighteen Annual Meeting. Volume 18: 277–282.

[B1003] Remillet M (1988) Catalogue des insects ravageurs des cultures en Guyane Française. Office de la recherche scientifique et technique outre-mer. Institut Française de Recherche Scientifique pour le Développement en Coopération. Collection Études et Thèses, Paris, 238 pp.

[B1004] Renkema JM Difonzo CD Smith JL Schaafsma AW (2015) Effect of European chafer larvae (Coleoptera: Scarabaeidae) on winter wheat and role of neonicotinoid seed treatments in their management. Journal of Economic Entomology 108: 566–575. https://doi.org/10.1093/jee/tov002 10.1093/jee/tov002 26470167

[B1005] Restrepo-Giraldo H Morón MA Vallejo F Pardo-Locarno LC López-Avila A (2003) Catálogo de Coleoptera Melolonthidae (Scarabaeidae Pleurosticti) de Colombia. Folia Entomológica Mexicana 42: 239–263.

[B1006] Reyes Novelo E Morón MA (2005) Fauna de Coleoptera Melolonthidae y Passalidae de Tzucacab y Conkal, Yucatán, México. Acta Zoológica Mexicana (n.s.)21: 15–49.

[B1007] Reynolds WC Reynolds DS Puckett RT Wade T Elmore MT (2016) Monitoring destructive scarab beetles in Texas turfgrass. Southwestern Entomologist 41: 921–931. https://doi.org/10.3958/059.041.0423

[B1008] Rice ME (1994) Damage assessment of the annual white grub, *Cyclocephala lurida* (Coleoptera: Scarabaeidae), in corn and soybean. Journal of Economic Entomology 87: 220–222. https://doi.org/10.1093/jee/87.1.220

[B1009] Richmond EA (1962) The fauna and flora of Horn Island, Mississippi. Gulf Research Reports 1: 59–118. https://doi.org/10.18785/grr.0102.01

[B1010] Rickson FR Cresti M Beach JH (1990) Plant cells which aid in pollen digestion within a beetle’s gut. Oecologia 82: 424–426. https://doi.org/10.1007/BF00317493 10.1007/BF00317493 28312721

[B1011] Riegel GT (1942a) Relative abundance of *Cyclocephala immaculata* and *C. borealis* at Urbana. Transactions of the Illinois State Academy of Science 34: 234–235.

[B1012] Riegel GT (1942b) *Cyclocephala abrupta* in Illinois (Coleop.: Scarab.). Transactions of the Illinois State Academy of Science 35: 215.

[B1013] Riegel GT (1948) Sex and altitude of flight in *Cyclocephala* (Coleoptera: Scarabaeidae). Transactions of the Illinois State Academy of Science 41: 113–115.

[B1014] Riehs PJ (2005) Similaridade entre comunidades de Dynastinae (Coleoptera, Scarabaeidae) do leste e centro-oeste do Paraná: umaabordagem paleoclimática. Ambiência-Revista do Centro de Ciências Agrárias e Ambientais 1: 59–69.

[B1015] Riehs PJ (2006) Fenologia de algumas espécies do gênero *Cyclocephala* (Coleoptera, Scarabaeidae) do leste e centro-oeste do Paraná, Brasil. Revista Ciências Exatas e Naturais 8: 201–223.

[B1016] Riley EG Wolfe CS (2003) An annotated checklist of the Scarabaeoidea of Texas (Coleoptera). Southwestern Entomologist Supplement 26: 1–37.

[B1017] Ritcher PO (1940a) *Cyclocephala immaculata* Oliv. as a test insect. Journal of Economic Entomology 33: 704. https://doi.org/10.1093/jee/33.4.704 https://doi.org/10.1093/jee/33.4.704

[B1018] Ritcher PO (1940b) Kentucky white grubs. Kentucky Agricultural Experiment Station Bulletin 401: 1–151.

[B1019] Ritcher PO (1944) Dynastinae of North America: with descriptions of the larvae and keys to genera and species (Coleoptera: Scarabaeidae). Kentucky Agricultural Experiment Station Bulletin 467: 1–51.

[B1020] Ritcher PO (1958) Biology of Scarabaeidae Annual Review of Entomology 3: 311–334. https://doi.org/10.1146/annurev.en.03.010158.001523

[B1021] Ritcher PO (1966) White grubs and their allies. Oregon State Monographs (Studies in Entomology) 4: 1–219.

[B1022] Ritcher PO Baker CW (1974) Ovariole numbers in Scarabaeoidea (Coleoptera: Lucanidae, Passalidae, Scarabaeidae). Proceedings of the Entomological Society of Washington 76: 481–494.

[B1023] Rivera-Gasperín SL Carrillo-Ruiz H Morón MA Yanes-Gómez G (2013) Fauna de Coleoptera Melolonthidae (Scarabaeoidea) en el Rancho Canaletas, Pas del Macho, Veracruz, México. Acta Zoológica Mexicana (n.s.)29: 194–208.

[B1024] Rivera-Gasperín SL Morón MA (2013) Análisis filogenético del subgénero Phyllophaga (Triodonyx) (Coleoptera: Melolonthidae: Melolonthinae). Revista Mexicana de Biodiversidad 84: 802–817. https://doi.org/10.7550/rmb.34034

[B1025] Roberts RJ (1963a) Improved methods for obtaining and rearing first-instar *Cyclocephala immaculata* larvae for experimentation. Journal of Economic Entomology 56: 538–540. https://doi.org/10.1093/jee/56.4.538

[B1026] Roberts RJ (1963b) Availability of dieldrin to adult *Blissus leucopterus* and larval *Cyclocephala immaculata* in treated sand, loam, and much soils. Journal of Economic Entomology 56: 781–785. https://doi.org/10.1093/jee/56.6.781

[B1027] Robinson M (1947) A new species of *Stenocrates* from Central America. Entomological News 58: 233–234.

[B1028] Rodrigues SR Nogueira GAL Echeverria RR Oliveira VS (2010) Aspectos biológicos de *Cyclocephala verticalis* Burmeister (Coleoptera: Scarabaeidae). Neotropical Entomology 39: 15–18. https://doi.org/10.1590/S1519-566X2010000100003 10.1590/s1519-566x2010000100003 20305894

[B1029] Rodrigues SR Carmo JI do Santos Oliveira V dos Tiago EL Taira TL (2011) Ocorrência de larvas de Scarabaeidae fitófagos (Insecta: Coleoptera) em diferentes sistemas de sucessão de culturas. Pesquisa Agropecuária Tropical 41: 87–93. https://doi.org/10.5216/pat.v41i1.7698

[B1030] Rogers CE (1992) Insect pests and strategies for their management in cultivated sunflower. Field Crops Research 30: 301–332. https://doi.org/10.1016/0378-4290(92)90005-T

[B1031] Rogers ME Cole TJ Ramaswamy SB Potter DA (2003) Behavioral changes in Japanese beetle and masked chafer grubs (Coleoptera: Scarabaeidae) after parasitism by tiphiid wasps (Hymenoptera: Tiphiidae). Environmental Entomology 32: 618–625. https://doi.org/10.1603/0046-225X-32.3.618

[B1032] Rogers ME Potter DA (2004a) Preovipositional behaviors of *Tiphia pygidialis* and *Tiphia vernalis* (Hymenoptera: Tiphiidae), parasitoids of white grubs (Coleoptera: Scarabaeidae). Annals of the Entomological Society of America 97: 605–612. https://doi.org/10.1603/0013-8746(2004)097[0605:PBOTPA]2.0.CO;2

[B1033] Rogers ME Potter DA (2004b) Biology of *Tiphia pygidialis* (Hymenoptera: Tiphiidae), a parasitoid of masked chafer (Coleoptera: Scarabaeidae) grubs, with notes on the seasonal occurrence of *Tiphia vernalis* in Kentucky. Environmental Entomology 33: 520–527. https://doi.org/10.1603/0046-225X-33.3.520

[B1034] Rogers ME Potter DA (2004c) Potential for sugar sprays and flowering plants to increase parasitism of white grubs (Coleoptera: Scarabaeidae) by tiphiid wasps (Hymenoptera: Tiphiidae). Environmental Entomology 33: 619–626. https://doi.org/10.1603/0046-225X-33.3.619

[B1035] Romero-López AA Negrete-Yankelevich S de la Rosa I Geissert D (2012) Presencia de “gallinas ciegas” (Coleoptera: Scarabaeoidea: Melolonthidae) en el bosque mesófilo y su distribución espacial en un pastizal. Southwestern Entomologist 37: 419–422. https://doi.org/10.3958/059.037.0319

[B1036] Romero-López AA Rodríguez-Palacios E Alarcón-Gutiérrez E Geissert D Barois I (2015) Effects of white grubs on soil water infiltration. Neotropical Entomology 44: 134–139. https://doi.org/10.1007/s13744-015-0273-x 10.1007/s13744-015-0273-x 26013131

[B1037] Romero-López M Morón MA (2017) Dos nuevas especies de Coleoptera Melolonthidae de la Costa Grande de Guerrero, México. Southwestern Entomologist 42: 889–900. https://doi.org/10.3958/059.042.0327

[B1038] Ronqui DC Lopes J (2006) Composição e diversidade de Scarabaeoidea (Coleoptera) atraídos por armadilha de luz em área rural norte do Paraná, Brasil. Iheringia, Série Zoologia 96: 103–108. https://doi.org/10.1590/S0073-47212006000100018

[B1039] Rosa CA Morais PB Santos SR Peres Neto RR Mendonça-Hagler LC Hagler AN (1995) Yeast communities associated with different plant resources in sandy coastal plains of southeastern Brazil. Mycological Research 99: 1047–1054. https://doi.org/10.1016/S0953-7562(09)80771-5

[B1040] Rosa CA Lachance MA Starmer WT Barker JSF Bowles JM Schlag-Edler B (1999) *Kodamaea nitidulidarum*, *Candida restingae* and *Kodamaea anthophila*, three new related yeast species from ephemeral flowers. International Journal and Systematic Bacteriology 49: 309–318. https://doi.org/10.1099/00207713-49-1-309 10.1099/00207713-49-1-309 10028276

[B1041] Roth RR Newsom JD Joanen T McNease LL (1972) The daily and seasonal behavior patterns of the Clapper Rail (*Rallus longirostris*) in the Louisiana coastal marshes. Proceedings of the Annual Conference of the Southeastern Association of Game and Fish Commissioners 26: 136–159.

[B1042] Rothwell NL Smitley DR (1999) Impact of golf course mowing practices on *Ataenius spretulus* (Coleoptera: Scarabaeidae) and its natural enemies. Environmental Entomology 28: 358–366. https://doi.org/10.1093/ee/28.3.358

[B1043] Royer TA Walker NR (2005a) White grub control in bermudagrass turf, 2004. Arthropod Management Tests 30(1): G20. https://doi.org/10.1093/amt/30.1.G20

[B1044] Royer TA Walker NR (2005b) White grub control in turfgrass, 2004. Arthropod Management Tests 30(1): G44. https://doi.org/10.1093/amt/30.1.G44

[B1045] Royer TA Walker NR Rebek EJ (2009) Evaluation of early and late applications of DPX E2Y45 (chlorantraniliprole) for control of white grubs in bermudagrass turf in Oklahoma, 2007. Arthropod Management Tests 34(1): G6. https://doi.org/10.4182/amt.2009.G6

[B1046] Roze JA (1955) Lista preliminar de la familia Scarabaeidae (*sensu lato*) (Coleoptera) de Venezuela. Boletín del Museo de Ciencias Naturales (Caracas, Venezuela) 1: 39–63.

[B1047] Ruiz BN Pamalpa CN (1990) Observaciones sobre las chisas (Coleoptera: Scarabaeidae) en Nariño. Revista ICA: Publicación Científica del Instituto Colombiano Agropecuario 25: 275–282.

[B1048] Ruiz BN Posada OL (1986) Aspectos biologicos de las chisas en la sabana de Bogota. Revista Colombiana de Entomología 11: 21–26.

[B1049] Saenz A Morelli E (1984) El genero *Cyclocephala* en el Uruguay (Coleoptera: Dynastinae). Revista de la Facultad de Humanidades y Ciencias. Series Ciencias Biologicas 1: 469–489.

[B1050] Saenz A Morelli E (1985) El genero *Chalepides* Casey, en el Uruguay. (Coleoptera: Dynastinae). Comunicaciones Zoologicas del Museo de Historia Natural de Montevideo 11: 1–9.

[B1051] Salgado-Neto G Vaz MAB Guedes JVC Muniz MFB Blume E (2016) Dispersão de *Fusarium oxysporum* por larvas de *Cyclocephala modesta*, *Dyscinetus gagates* e *Dilboderus abderus* no Brasil. Ciência Rural, Santa Maria 46: 943–949. https://doi.org/10.1590/0103-8478cr20150471

[B1052] Saltin J-P Ratcliffe BC (2012) A review of the distribution of *Harposcelis paradoxus* Burmeister, 1847 (Coleoptera: Scarabaeidae: Dynastinae: Cyclocephalini) with a new country record for Peru. Dugesiana 18: 147–151.

[B1053] Samples TJ Sorochan JC Brilman LA Stier JC (2009) Tall fescue as turf in the United States. In: Fribourg HA, Hannaway DB, West CP (Eds) Tall Fescue for the Twenty-first Century. Agronomy Monograph 53. American Society of Agronomy, Crop Science Society of America, Soil Science Society of America, Madison, 445–481. https://doi.org/10.2134/agronmonogr53.c26

[B1054] Sanchez Soto S (1997) Nuevos registros de Melolonthidae (Coleoptera) para el estado de Tabasco, México. Folia Entomológica Mexicana 100: 67–70.

[B1055] Sanchez Soto S (1998) Nuevos datos de distribucion de Melolonthidae (Coleoptera) en México, con nuevos registros para Tabasco. Folia Entomológica Mexicana 102: 75–76.

[B1056] Sanderson MW (1939) An annual white grub, *Cyclocephala immaculata* Oliv. Arkansas Agricultural Experiment Station Bulletin 386: 67–68.

[B1057] Sanderson MW (1940) Arkansas Cyclocephalini with notes on Burmeister types (Coleoptera: Scarabaeidae). Annals of the Entomological Society of America 33: 377–384. https://doi.org/10.1093/aesa/33.2.377

[B1058] San Martín LE (1968) Control químico de los gusanos de suelo en cultivos de papa en Balcarce. IDIA 64–72.

[B1059] Santos V Ávila CJ (2007) Aspectos bioecológicos de *Cyclocephala forsteri* Endrődi, 1963 (Coleoptera: Melolonthidae) no estado do Mato Grosso do Sul. Revista de Agricultura 82: 298–303.

[B1060] Santos Fava W da Silva Covre W Sigrist MR (2011) *Attalea phalerata* and *Bactris glaucescens* (Arecaceae, Arecoideae): Phenology and pollination ecology in the Pantanal, Brazil. Flora 206: 575–584. https://doi.org/10.1016/j.flora.2011.02.001

[B1061] Santos Fava W Gomes VGN (2017) “Back-to-bud” strategy in *Nymphaea amazonum* (Nymphaeaceae): A protogynous macrophyte of the Pantanal wetlands. Aquatic Botany 140: 1–3. https://doi.org/10.1016/j.aquabot.2017.04.001

[B1062] Saylor LW (1936) New California and Texas scarabs. Journal of Entomology and Zoology 28: 1–4.

[B1063] Saylor LW (1937) Revision of California *Cyclocephala* (Coleoptera: Scarabaeidae). Journal of Entomology and Zoology 29: 67–70.

[B1064] Saylor LW (1945) Synoptic revision of the United States scarab beetles of the subfamily Dynastinae, No. 1: Tribe Cyclocephalini. Journal of the Washington Academy of Sciences 35: 378–386.

[B1065] Saylor LW (1946) Revision of the scarab beetles of the dynastine genus *Erioscelis*. Proceedings of the Entomological Society of Washington 48: 61–66.

[B1066] Saylor LW (1948) Contributions toward a knowledge of the insect fauna of Lower California. No. 10. Coleoptera: Scarabaeidae Proceedings of the California Academy of Sciences, 4^th^ Series, 24: 337–374.

[B1067] Scammell HB (1917) Cranberry insect problems and suggestions for solving them. United States Department of Agriculture Farmers’ Bulletin 860: 1–42.

[B1068] Scariot AO Lieras E Hay JD (1991) Reproductive biology of the palm *Acronomia aculeata* in central Brazil. Biotropica 23: 12–22. https://doi.org/10.2307/2388683

[B1069] Schaeffer C (1906) On *Bradycinetus* and *Bolboceras* of North America, with notes on other Scarabaeidae. Transactions of the American Entomological Society 32: 249–260.

[B1070] Schaeffer SK Stamm MD Toda L Baxendale FP (2008) Efficacy of new imidacloprid formulations for control of southern masked chafers, 2007. Arthropod Management Tests 33(1): G47. https://doi.org/10.1093/amt/33.1.G47

[B1071] Schatz GE (1985) A new *Cymbopetalum* (Annonaceae) from Costa Rica and Panama with observations on natural hybridization. Annals of the Missouri Botanical Graden 72: 535–538. https://doi.org/10.2307/2399102

[B1072] Schatz GE (1987) Systematic and ecological studies of Central American Annonaceae. PhD thesis, Madison, USA: University of Wisconsin-Madison.

[B1073] Schawaller W (1994) Die Käfersammlung am Naturkundemuseum Stuttgart. Stuttgarter Beiträge zur Naturkunde, Serie A (Biologie) 508: 1–40.

[B1074] Schönherr CJ (1806) Synonymia Insectorum, oder Versuch einer Synonymie aller bisher bekannten Insecten; nach Fabricii Systema Eleutheratorum geordnet, volume 1, part 1. Heinr. A. Nordström, Stockholm, 293 pp. https://doi.org/10.5962/bhl.title.66107

[B1075] Schönherr CJ (1817) Synonymia Insectorum, oder Versuch einer Synonymie aller bisher bekannten Insecten; nach Fabricii Systema Eleutheratorum etc. geordnet, volume 1, part 3. Em. Bruzelius, Uppsala, 506 pp.

[B1076] Schrader J Oonincx DGAB Ferreira MP (2016) North American entomophagy. Journal of Insects as Food and Feed 2: 111–120. https://doi.org/10.3920/JIFF2016.0003

[B1077] Schrottky C (1908) Blumen und Insekten in Paraguay. Zeitschrift für wissenschaftliche Insektenbiologie 4: 22–26.

[B1078] Schrottky C (1910) Die Befruchtung von *Philodendron* und *Caladium* durch einen Käfer (*Erioscelis emarginata*). Zeitschrift für wissenschaftliche Insektenbiologie 6: 67–68.

[B1079] Schrottky C (1913) Die Entomologische Literatur Süd-Amerikas 1905–1912. Zeitschrift für wissenschaftliche Insektenbiologie 9: 346–352.

[B1080] Schwarz EA (1878) The Coleoptera of Florida. Proceedings of the American Philosophical Society 17: 353–469.

[B1081] Seidlitz G (1904) Coleoptera. Archiv für Naturgeschichte 70 (2, 2): 54–356.

[B1082] Seidlitz G (1905) Coleoptera für 1904. Archiv für Naturgeschichte 71(2, 2): 44–360.

[B1083] Senoner A (1864) Bücher-Anzeigen [Teil c]. Wiener Entomologische Monatsschrift 8: 139–140.

[B1084] Seres A Ramírez N (1995) Biologia floral y polinizacion de algunas Monocotiledoneas de un Bosque Nublado Venezolano. Annals of the Missouri Botanical Garden 82: 61–81. https://doi.org/10.2307/2399981

[B1085] Seymour RS Matthews PDG (2006) The role of thermogenesis in the pollination biology of the Amazon waterlily *Victoria amazonica* Annals of Botany 98: 1129–1135. https://doi.org/10.1093/aob/mcl201 10.1093/aob/mcl201 PMC2803590 17018568

[B1086] Seymour RS White CR Gibernau M (2003) Heat reward for insect pollinators: scarab beetles save energy by making themselves at home inside a warm flower. Nature 426: 243–244. https://doi.org/10.1038/426243a 10.1038/426243a 14628037

[B1087] Seymour RS White CR Gibernau M (2009) Endothermy of dynastine scarab beetles (*Cyclocephala colasi*) associated with pollination biology of a thermogenic arum lily (*Philodendron solimoesense*). The Journal of Experimental Biology 212: 2960–2968. https://doi.org/10.1242/jeb.032763 10.1242/jeb.032763 19717678

[B1088] Shapiro-Ilan DI Cottrell TE (2005) Susceptibility of lady beetles (Coleoptera: Coccinellidae) to entomopathogenic nematodes. Journal of Invertebrate Pathology 89: 150–156. https://doi.org/10.1016/j.jip.2005.04.002 10.1016/j.jip.2005.04.002 15913642

[B1089] Sharp D (1877) Description of some new species of beetles (Scarabaeidae) from Central America. The Journal of the Linnean Society of London (Zoology)13: 129–138. https://doi.org/10.1111/j.1096-3642.1877.tb02376.x

[B1090] Shetlar DJ Andon J (2013) Preventive control of white grubs on a golf course turf, 2008. Arthropod Management Tests 38(1): G15. https://doi.org/10.4182/amt.2013.G15

[B1091] Shetlar DJ Andon J (2015) Curative control of white grubs in turf, 2010. Arthropod Management Tests 40(1): G15. https://doi.org/10.1093/amt/tsv190

[B1092] Shockley FW Cline AR (2004) A contribution to the inventory of Coleoptera of Missouri: New records from Benton County. Journal of the Kansas Entomological Society 77: 280–284. https://doi.org/10.2317/0304.21.1

[B1093] Silberbauer-Gottsberger I (1990) Pollination and evolution in palms. Phyton 30: 213–233.

[B1094] Silberbauer-Gottsberger I Gottsberger G Webber AC (2003) Morphological and functional flower characteristics of New and Old World Annonaceae with respect to their mode of pollination. Taxon 52: 701–718. https://doi.org/10.2307/3647345

[B1095] Silberbauer-Gottsberger I Gottsberger RA Gottsberger G (1997) Flowering rhythm and pollination in a hybrid population of *Annona* in a small cerrado area in Mato Grosso, Brazil. Annonaceae Newsletter 11: 55–60.

[B1096] Silberbauer-Gottsberger I Webber AC Küchmeister H Gottsberger G (2001) Convergence in beetle-pollinated central Amazonian Annonaceae, Araceae, Arecaceae, and Cyclanthaceae. In: Gottsberger G, Liede S (Eds) Life forms and dynamics in tropical forests. Dissertationes Botanicae 346. J. Cramer in der Gebrüder Bornträger Verlagsbuchhandlung, Berlin and Stuttgart, 165–183.

[B1097] Silvera Guido A (1965) Natural enemies of weed plants. Final report. Unpublished report, Department Sanidad Vegetal, University de la Republic, Montevideo.

[B1098] Sim RF (1934) Characters useful in distinguishing larvae of *Popillia japonica* and other introduced Scarabaeidae from native species. United States Department of Agriculture Circular 334: 1–20. https://doi.org/10.5962/bhl.title.64092

[B1099] Sima DB Srivastava M (2014a) Floral visitors of different crops as recorded from an agro-ecosystem near Jhunjhunu, Rajasthan (India). International Journal of Science and Research 3: 1732–1738.

[B1100] Sima DB Srivastava M (2014b) A comparative study of insect collection made by employing two different methods of collection in an agro-ecosystem near Jhunjhunun, Rajasthan, India. International Journal of Science and Research 3: 1739–1748.

[B1101] Sima DB Srivastava M (2016) A study on entomo-fauna as recorded from cauliflower crop in an agro-ecosystem near Bikaner, Rajasthan, India. International Journal of Current Microbiology and Applied Sciences 5: 539–545. https://doi.org/10.20546/ijcmas.2016.504.061

[B1102] Sites RW (2017) The aroid scarab *Peltonotus nasutus* (Coleoptera: Scarabaeidae) in Thailand and its association with *Amorphophallus paeoniifolius* (Araceae). Natural History Bulletin of the Siam Society 62: 107–109.

[B1103] Smith ABT (2003) Checklist of the Scarabaeoidea of the Nearctic Realm. Version 3. Electronically published, Lincoln, 74 pp.

[B1104] Smith ABT (2009) Checklist and Nomenclatural Authority File of the Scarabaeoidea of the Nearctic Realm including Canada, the continental United States, and the northern Mexican states of Baja California, Baja California Sur, Chihuahua, Coahuila de Zaragoza, Durango, Nuevo Leon, Sinaloa, Sonora, Tamaulipas, and Zacatecas. Version 4 - released 22 April 2009. Electronically published, Canadian Museum of Nature, Ottawa, 97 pp.

[B1105] Smith JB (1910) Coleoptera. Annual Report of the New Jersey State Museum 1909: 195–406.

[B1106] Smyth EG (1915) Report of work at the South Coast Laboratory. Thrid Report of the Board of Commissioners of Agriculture of Porto Rico from the period from July 1, 1913 to July 1, 1914: 40–53.

[B1107] Smyth EG (1916) Report of the South Coast Laboratory. Fourth Report of the Board of Commissioners of Agriculture of Porto Rico for the period from July 1, 1914, to June 30, 1915: 45–49.

[B1108] Soave GE Camperi AR Darrieu CA Cicchino AC Ferretti V Juarez M (2006) White-faced Ibis diet in Argentina. Waterbirds: The International Journal of Waterbird Biology 29: 191–197. https://doi.org/10.1675/1524-4695(2006)29[191:WIDIA]2.0.CO;2

[B1109] Soderstrom M (2008) Botanical gardens. In: Jorgensen SE, Fath B (Eds) Encyclopedia of Ecology. Volume 1 A–C. Elsevier Science, Amsterdam, 495–502. https://doi.org/10.1016/B978-008045405-4.00320-7

[B1110] Souza TB Maia ACD Albuquerque CMR de Iannuzzi L (2014a) Description of *Cyclocephala distincta* Burmeister (Coleoptera: Scarabaeidae: Dynastinae: Cyclocephalini) immatures and identification key for third instars of some *Cyclocephala* species. Zootaxa 3872: 180–186. https://doi.org/10.11646/zootaxa.3872.2.4 10.11646/zootaxa.3872.2.4 25544079

[B1111] Souza TB Maia ACD Schlindwein C Albuquerque LSC de Iannuzzi L (2014b) The life *Cyclocephala celata* Dechambre, 1980 (Coleoptera: Scarabaeidae: Dynastinae) in captivity with descriptions of the immature stages. Journal of Natural History 48: 275–283. https://doi.org/10.1080/00222933.2013.791886

[B1112] Souza TB Maia ACD Albuquerque CMR de Iannuzzi L (2015) Biology and management of the masked chafer *Cyclocephala distincta* Burmeister (Melolonthidae, Dynastinae, Cyclocephalini). Revista Brasileira de Entomologia 59: 37–42. https://doi.org/10.1016/j.rbe.2015.02.004

[B1113] Squire FA (1932) Principles crop pests: sugar cane, rice, coconuts. Department of Agriculture (British Guiana), Report of the Entomological Division for the year 1932: 135–140.

[B1114] Squire FA (1933) Principles crop pests: sugar cane; rice and padi; pineapples; coconuts. Department of Agriculture (British Guiana), Report of the Entomological Division for the year 1933: 125–128.

[B1115] Squire FA (1972) Entomological problems in Bolivia. PANS Pest Articles and News Summaries 18: 249–268. https://doi.org/10.1080/09670877209411802

[B1116] Stahl A (1882) Catálogo del cabinete zoológico del Dr. A. Stahl, en Bayamon (Pto.-Rico). Precidido de una clasificacion sistemática de los animales que corresponden á esta fauna. Parte Segunda. Boletin Mercantil; San Jaun, Puerto Rico, 129–249.

[B1117] Stahl CF Scaramuzza LC (1929) Soil insects attacking sugar cane in Cuba. Tropical Plant Research Foundation, Bulletin 10: 1–19.

[B1118] Stahly DP Klein MG (1992) Problems with *in vitro* production of spores of *Bacillus popilliae* for use in biological control of the Japanese beetle. Journal of Invertebrate Pathology 60: 283–291. https://doi.org/10.1016/0022-2011(92)90010-2

[B1119] Staines Jr CL (1984) An annotated checklist of the Scarabaeoidea (Coleoptera) of Maryland. Maryland Entomologist 2: 79–89.

[B1120] Staines Jr CL (1990) *Dyscinetus morator* (Coleoptera: Scarabaeidae) feeding on roots of azaleas (*Rhododendron* spp.). Entomological News 101: 98.

[B1121] Stamm MD Baxendale RW (2008a) Effect of application timing with Acelepryn for control of southern masked chafers, 2007. Arthropod Management Tests 33(1): G48. https://doi.org/10.1093/amt/33.1.G48

[B1122] Stamm MD Baxendale RW Koch KG Prochaska TJ (2012) *Chromobacterium subtsugae* (MBI-203) for control of southern masked chafers, 2011. Arthropod Management Tests 37(1): G9.

[B1123] Stamm MD Baxendale RW Pierson LM Baxendale FP (2009) Efficacy of SDS502 granules for curative control of southern masked chafer. Arthropod Management Tests 34(1): G16. https://doi.org/10.4182/amt.2009.G16

[B1124] Stamm MD Chess J Heng-Moss TM (2008b) Preventive applications of Acelepryn for control of southern masked chafers, 2007. Arthropod Management Tests 33(1): G49. https://doi.org/10.1093/amt/33.1.G49

[B1125] Stamm MD Marchi Werle LS Kock KG Prochaska TJ (2015) Evaluation of Acelepryn for control of southern masked chafers, 2013. Arthropod Management Tests 39(1): G7.

[B1126] Stamm MD Prochaska TJ Matz NA Baxendale RW (2013) Efficacy of *Chromobacterium subtsugae* for control of southern masked chafer, 2012. Arthropod Management Tests 38(1): G16. https://doi.org/10.4182/amt.2013.G16

[B1127] Stansly PA Cherry RH Sosa Jr O (1994) Relative abundance of white grubs (Coleoptera: Scarabaeidae) in Florida sugarcane on sand and muck soil. Journal of the American Society of Sugar Cane Technologists 14: 19–24.

[B1128] Stechauner-Rohringer R Pardo-Locarno LC (2010) Redescripción de inmaduros, ciclo de vida, distribución e importancia agrícola de *Cyclocephala lunulata* Burmeister (Coleóptera: Melolonthidae: Dynastinae) en Colombia. Boletín Científico de Historia Natural Universidad de Caldas 14: 203–220.

[B1129] Steinheil E (1874) Symbolae ad historiam Coleopterorum Argentiniae meridionalis. II. Centuria. Atti della Società Italiana di Scienze Naturali 15: 554–578.

[B1130] Stevenson JA (1918) The green muscardine fungus in Porto Rico (*Metarrhizium anisopliae* [Metsch.] Sorokin). The Journal of the Department of Agriculture of Porto Rico 2: 19–32.

[B1131] Stock SP Campbell JF Nadler SA (2001) Phylogeny of *Steinernema* Travassos, 1927 (Cephalobina: Steinernematidae) inferred from ribosomal DNA sequences and morphological characters. The Journal of Parasitology 87: 877–889. https://doi.org/10.2307/3285148 10.1645/0022-3395(2001)087[0877:POSTCS]2.0.CO;2 11534654

[B1132] Stone JD (1986) Time and height of flight of adults of white grubs (Coleoptera: Scarabaeidae) in the southwestern United States. Environmental Entomology 15: 194–197. https://doi.org/10.1093/ee/15.1.194

[B1133] Strand E (1912) Beiträge zur Kenntnis der Hymenopterenfauna von Paraguay auf Grund der Sammlungen und Beobachtungen von Prof. J. D. Anisits. Zoologische Jahrbücher. Abteilung für Systematik, Geographie und Biologie der Tiere 33: 257–346.

[B1134] Stuart RJ Barbercheck ME Grewal PS Taylor RAJ Hoy CW (2006) Population biology of entomopathogenic nematodes: Concepts, issues, and models. Biological Control 38: 80–102. https://doi.org/10.1016/j.biocontrol.2005.09.019

[B1135] Sturm J (1843) Catalog der Kaefer-Sammlung von Jacob Sturm. Sebald’s Buchdruckerei, Nuremberg, 386 pp. [+ 6 pls] https://doi.org/10.5962/bhl.title.37837

[B1136] Su K Bremer DJ Keeley SJ Fry JD (2008) Rooting characteristics and canopy responses to drought of turfgrasses including hybrid bluegrasses. Agronomy Journal 100: 949–956. https://doi.org/10.2134/agronj2007.0292

[B1137] Subramanian S Muthulakshmi M (2016) Entomopathogenic nematodes. In: Omkar (Ed.) Ecofriendly pest management for food security. Elsevier Academic Press, San Diego, 368–410. https://doi.org/10.1016/B978-0-12-803265-7.00012-9

[B1138] Suggars Downing A (1994) Effect of irrigation and spray volume on efficacy of entomopathogenic nematodes (Rhabditida: Heterorhabditidae) against white grubs (Coleoptera: Scarabaeidae). Journal of Economic Entomology 87: 643–646. https://doi.org/10.1093/jee/87.3.643

[B1139] Sutton MQ (1988) Insects as food: Aboriginal entomophagy in the great basin. Ballena Press Anthropological Papers 33. Ballena Press, Menlo Park, 115 pp.

[B1140] Swan LA Papp CS (1972) Common insects of North America. Harper and Row, New York, 750 pp.

[B1141] Swenk MH (1911) Notes on some insects injurious in Nebraska in 1910. Journal of Economic Entomology 4: 283–286. https://doi.org/10.1093/jee/4.2.283

[B1142] Swenk MH (1913) The principle insects injurious to agriculture during 1911–1912. Bulletin of the Nebraska State Entomologist 1: 1–104.

[B1143] Tanada Y Kaya HK (1993) Insect pathology. First edition. Elsevier Academic Press, San Diego, 666 pp.

[B1144] Tashiro H (1987) Turfgrass insects of the United States and Canada. Comstock Publishing Associates, Cornell University Press, Ithaca, 391 pp.

[B1145] Theunis W (1998) Susceptibility of the taro beetle, *Papuana uninodis*, to entomopathogenic nematodes. International Journal of Pest Management 44: 139–143. https://doi.org/10.1080/096708798228239

[B1146] Thien LB Bernhardt P Devall MS Chen Z-D Luo Y-B Fan J-H Yuan L-C Williams JH (2009) Pollination biology of basal angiosperms (ANITA grade). American Journal of Botany 96: 166–182. https://doi.org/10.3732/ajb.0800016 10.3732/ajb.0800016 21628182

[B1147] Thomas DB (1993) Scarabaeidae (Coleoptera) of the Chiapanecan forests: A faunal survey and chorographic analysis. The Coleopterists Bulletin 47: 363–408.

[B1148] Thunberg CP (1814) Coleoptera capensia, antennis lemellatis, sive clava fissile instructa. Mémoires de l’Académie Impériale des Sciences de St. Pétersbourg 6 (1813–1814): 395–450.

[B1149] Thurston GS Kaya HK Burlando TM Harrison RE (1993) Milky disease bacterium as a stressor to increase susceptibility of scarabaeid larvae to an entomopathogenic nematode. Journal of Invertebrate Pathology 61: 167–172. https://doi.org/10.1006/jipa.1993.1030

[B1150] Thurston GS Kaya HK Gaugler R (1994) Characterizing the enhanced susceptibility of milky disease-infected scarabaeid grubs to entomopathogenic nematodes. Biological Control 4: 67–73. https://doi.org/10.1006/bcon.1994.1012

[B1151] Toda L Baxendale FP Heng-Moss TM (2006) Use of DPX E2Y45 for control of white grubs, 2005. Arthropod Management Tests 31(1): G27. https://doi.org/10.1093/amt/31.1.G27

[B1152] Toda L Eickhoff TE Heng-Moss TM (2007) DPX E2Y45 for control of white grubs, 2006. Arthropod Management Tests 32(1): G7. https://doi.org/10.1093/amt/32.1.G7

[B1153] Touroult J Dalens P-H Ponchel Y (2010) Échantillonnage des Dynastidae par piégeage lumineux: comparaison entre le début et la fin de nuit en Guyane (Coleoptera, Scarabaeoidea, Dynastidae). Contribution á L’Étude des Coléoptères de Guyane 1: 11–14.

[B1154] Tremoleras J (1910) Coleopterologische Skizze von Uruguay. Entomologische Blätter 6: 22–28.

[B1155] Ulmen K Newzella R Hubweber L Schmitt M Klug T Ahrens D (2010) Contribution to a catalogue of types preserved in the collection of Zoologisches Forschungmuseum Alexander Koenig (ZFMK): Coleoptera: 1. Checklist of taxa. Bonn Zoological Bulletin 58: 5–48.

[B1156] Ungaro MRG Toledo NMP de Gobbo CRLF (1985) Competição de cultivares de girassol (*Helianthus annuus* L.). Revista de Agricultura 60: 193–215.

[B1157] Útima OA Vallejo-E LF (2008) Escarabajos Melolonthidae (Scarabaeidae-Pleurosticti) de la Montaña Cafetera, departamento de Risaralda, Colombia. Agronómica 16 (2): 31–44.

[B1158] Valerio CE (1984) Insect visitors of the inflorescence of the aroid *Dieffenbachia oerstedii* (Araceae) in Costa Rica. Brenesia 22: 139–146.

[B1159] Valerio CE (1988) Notes on phenology and pollination of *Xanthosoma wendlandii* (Araceae) in Costa Rica. Revista de Biología Tropical 36: 55–61.

[B1160] Valla JJ Cirino DR (1972) Biología floral del irupé, *Victoria cruziana* D’Orb. (Nymphaeaceae). Darwiniana 17: 477–500.

[B1161] Vallejo F Morón MA (2008) Description of the immature stages and redescription of the adults of *Ancognatha scarabaeoides* Erichson (Coleoptera: Scarabaeidae: Dynastinae), a member of the soil white grub assemblage in Colombia. The Coleopterists Bulletin 62: 154–164. https://doi.org/10.1649/1022.1

[B1162] Van Dinther JBM (1960) Insect pests of cultivated plants in Suriname. Agricultural Experiment Station Surinam Bulletin 76: 1–159.

[B1163] Vargas E Abarca G (1998) Relación entre el estrés y las bacterias entomopatógenas Pantoea (Erwinia) agglomerans (*herbicola*) y *Bacillus cereus* en jobotos (Col: Melolonthidae) (*Phyllophaga* spp., *Anomala* spp. y *Cyclocephala* spp.), en Costa Rica. Agronomía Mesoamericana 9: 25–30.

[B1164] Vásquez N Sánchez G (2004) Propuesta de manejo integrado de las chisas (Coleoptera, Melolonthidae) en el cultivo de arracacha. In: Hermann M, Hidalgo OA (Eds) Raíces Andinas: Contribuciones al conocimiento y a la capacitación. Conservación y uso de la biodiversidad de raíces y tubérculos andinos: Una década de investigación para el desarrollo (1993–2003). Centro Internacional de la Papa (CIP), Lima, 127–147.

[B1165] Venzon M Pallini Filho A (1995) Registro de *Stenocrates cultor* Burmeister (Coleoptera, Scarabaeidae) nas culturas de inhame, cará e batata doce. Ecossistema 20: 212.

[B1166] Vidal R Riva R Giacomozzi RO (1979) Numeros cromosomicos de Coleoptera de la Argentina. Physis 37: 341–342.

[B1167] Villalobos-Moreno A Pardo-Locarno LC Cabrero-Sañudo FJ Ospina-Torres R Gómez-Murillo IJ (2016) Inventario preliminar de los escarabajos de la familia Melolonthidae (Coleoptera: Scarabaeoidea) en un robledal del nororiente de los Andes Colombianos. Boletín de la Sociedad Entomológica Aragonesa 58: 159–167.

[B1168] Villalobos-Moreno A Pardo-Locarno LC Cabrero-Sañudo FJ Ospina-Torres R Gómez-Murillo IJ (2017) Escarabajos fitófagos (Coleoptera: Scarabaeoidea) de un robledal Andino del nororiente Colombiano. Boletín de la Sociedad Entomológica Aragonesa 61: 115–136.

[B1169] Villalta R (1988) Estudio de la biologia floral e identificacion de agentes polinizadores de guanábana *(Annona muricata* L.) en la zona atlantica de Costa Rica. Thesis. Heredia, Costa Rica, Universidad Nacional.

[B1170] Villegas-Urbano NP (2004) Reconocimiento de especies del complejo chisa (Coleoptera: Melolonthidae) asociados al cultivo de cebolla y pasto en la localidad de La Florida, Risaralda. Masters Thesis, Manizales, Colombia: Universidad de Caldas.

[B1171] Villegas-Urbano NP Gaigl A Vallejo LF (2008) El complejo chisa (Coleoptera: Melolonthidae) asociado a cebolla y pasto en Risaralda, Colombia. Revista Colombiana de Entomología 34: 83–89.

[B1172] Vincini AM López AN Manetti PL Alvarez-Castillo H Carmona DM (2000) Descripción de los estados inmaduros de *Dyscinetus rugifrons* (Burmeister, 1847) (Coleoptera: Scarabaeidae: Dynastinae). Elytron 14: 91–98.

[B1173] Vitorino M Buss A Valle P (2008) Ocorrência de *Dyscinetus rugifrons* Burmeister (Scarabaeidae: Dynastinae, Cyclocephalini) em plantios de Palmeira-Real-da-Austrália (*Archontophoenix* spp. H. Wendl. & Drude), no Vale-do-Itajaí, SC. Neotropical Entomology 37: 347–348. https://doi.org/10.1590/S1519-566X2008000300018 10.1590/s1519-566x2008000300018 18641910

[B1174] Vittum PJ Villani MG Tashiro H (1999) Turfgrass insects of the United States and Canada. Cornell University Press, Ithaca, 422 pp.

[B1175] Voeks RA (2002) Reproductive ecology of the piassava palm (*Attalea funifera*) of Bahia, Brazil. Journal of Tropical Ecology 18: 121–136. https://doi.org/10.1017/S0266467402002079

[B1176] Voet JE (1769) Catalogus systematicus Coleopterorum. Volume 1. G. Bakhuysen, The Hague, sections paginated separately + 54 pls.

[B1177] Von Bayern T (1897) Meine Reise in die Brasilianischen Tropen. D. Remmer, Berlin, 544 pp.

[B1178] Walker NR Royer TA (2002) Effects of insecticides and delayed irrigation on management of white grubs, 2001. Arthropod Management Tests 27(1): G10. https://doi.org/10.1093/amt/27.1.G10

[B1179] Wallin L (2001) Catalogue of type specimens. 3. Entomology. Version 4. Uppsala University, Museum of Evolution, Zoology Section, Uppsala, 55 pp.

[B1180] Warren GW Potter DA (1983) Pathogenicity of *Bacillus popilliae* (*Cyclocephala* Strain) and other milky disease bacteria in grubs of the southern masked chafer (Coleoptera: Scarabaeidae). Journal of Economic Entomology 76: 69–73. https://doi.org/10.1093/jee/76.1.69

[B1181] Weber A (2008) Pollination in the plants of the Golfo Dulce area. Stapfia 88: 509–538.

[B1182] Weber A Huber W Weissenhofer A Zamora N Zimmermann G (Eds) (2001) An introductory field guide to the flowering plants of the Golfo Dulce Rain Forests, Costa Rica. Corcovado National Park and Piedras Blancas National Park. Stapfia 78: 1–462 + 108 pls.

[B1183] Webber AC (1981) Biologia floral de algumas Annonaceae na região de Manaus AM. Masters thesis, Manaus, Brazil: Instituto Nascional de Pesquiras da Amazonia.

[B1184] Webber AC Gottsberger G (1993) Floral biology and pollination of *Cymbopetalum euneurum* in Manaus, Amazonia. Annonaceae Newsletter 9: 25–28.

[B1185] Wedin WF Huff DR (1996) Bluegrasses. In: Moser LE, Buxton DR, Casler MD (Eds) Cool-season forage grasses. Agronomy Monograph 34. American Society of Agronomy, Crop Science Society of America, Soil Science Society of America, Madison, 665–690.

[B1186] Weinhold AP Baxendale FP (2001) Application timing of Mach-2 on fertilizer carriers for control of white grubs, 2000. Arthropod Management Tests 26(1): G35. https://doi.org/10.1093/amt/26.1.G35

[B1187] Weinhold AP Baxendale FP Grisso RD (1999) High pressure injection of liquid insecticides for control of masked chafer larvae, 1998. Arthropod Management Tests 24(1): G53. https://doi.org/10.1093/amt/24.1.G53

[B1188] Werner FG Butler Jr GD (1965) Some notes on the life history of *Plega banksi* (Neuroptera: Mantispidae). Annals of the Entomological Society of America 58: 66–68. https://doi.org/10.1093/aesa/58.1.66

[B1189] White RT (1947) Milky disease infecting *Cyclocephala* larvae in the field. Journal of Ecoonomic Entomology 40: 912–914. https://doi.org/10.1093/jee/40.6.912 10.1093/jee/40.6.912 18858114

[B1190] Wickham HF (1894) The Coleoptera of Canada. IV. The pleurostict Scarabaeidae of Onatario and Quebec. The Canadian Entomologist 26: 259–264. https://doi.org/10.4039/Ent26259-9

[B1191] Wickham HF (1896) A list of some Coleoptera from the northern portions of New Mexico and Arizona. Bulletin from the Laboratories of Natural History, State University of Iowa 3 (4): 153–171.

[B1192] Wiehe PO (1951) Leaf scald and chlorotic streak: Two sugar cane diseases occurring in British Guiana. Lecture to the British Guiana Sugar Producers’ Association, 27^th^ August 1951. The Daily Chronicle Ltd, Georgetown, 33 pp.

[B1193] Wiersema JH (1987) A monograph of the Nymphaea subgenus Hydrocallis (Nymphaeaceae). Systematic Botany Monographs 16: 1–112. https://doi.org/10.2307/25027681

[B1194] Williamson RC Brandenburg R Thompson S (2004) Turfgrass insects of the United States: biology and management. In: Capinera JL (Ed.) Encyclopedia of Entomology. Volume 3, P-Z. Kluwer Academic Publishers, Dordrecht, 2372–2402. https://doi.org/10.1007/0-306-48380-7_4440

[B1195] Williamson RC Brandenburg R Thompson S (2008) Turfgrass insects of the United States: biology and management. In: Capinera JL (Ed.) Encyclopedia of Entomology. Second Edition. Volume 4, S-Z. Springer, Dordrecht, 3955–3992.

[B1196] Williamson RC Held DW Brandenburg R Baxendale F (2013) Turfgrass insect pests. In: Stier JC, Horgan BP, Bonos SA (Eds) Turgrass: biology, use, and management. Agronomy Monograph 56. American Society of Agronomy, Crop Science Society of America, Soil Science Society of America, Madison, 809–890. https://doi.org/10.2134/agronmonogr56.c23

[B1197] Wilson MJ Lewis EE Yoder F Gaugler R (2003) Application pattern and persistence of the entomopathogenic nematode *Heterorhabditis bacteriophora*. Biological Control 26: 180–188.

[B1198] Wolcott GN (1923) Insectae Portoricensis. A preliminary annotated checklist of the insects of Porto Rico, with descriptions of some news (*sic*) species. Journal of Agriculture of the University of Puerto Rico 7: 1–312. https://doi.org/10.1016/S1049-9644(02)00125-1

[B1199] Wolcott GN (1936) “Insectae borinquenses”: A revised annotated check-list of the insects of Puerto Rico. The Journal of Agriculture of the University of Puerto Rico 20: 1–600.

[B1200] Wolcott GN (1937) What the giant Surinam toad, *Bufo marinus* L., is eating now in Puerto Rico. The Journal of Agriculture of the University of Puerto Rico 21: 79–84.

[B1201] Wolcott GN (1948) Insects of Puerto Rico. Journal of Agriculture of the University of Puerto Rico 32: 1–975.

[B1202] Woodruff RE (1970) The “rice beetle,” *Dyscinetus morator* (Fab.) (Coleoptera: Scarabaeidae). Entomology Circular 103. Florida Department of Agriculture and Consumer Services, Division of Plant Industry, Gainesville.

[B1203] Woodruff RE (1973) The scarab beetles of Florida (Coleoptera: Scarabaeidae) Part 1. The Laprosticti (subfamilies: Scarabaeinae, Aphodiinae, Hybosorinae, Ochodaeinae, Geotrupinae, Acanthocerinae). Arthropods of Florida and Neighboring Land Areas 8: xi + 1–220.

[B1204] Woodruff RE (2013) Rice beetle, *Dyscinetus morator* (Fabricius) (Insecta: Coleoptera: Scarabaeidae). EENY-102 (originally published as DPI Entomology Circular 103). University of Florida, IFAS Extension, Gainesville, 3 pp.

[B1205] Woodruff RE Beck BM Skelley PE Schotman CYL Thomas MC (1998) Checklist and bibliography of the insects of Grenada and the Grenadines. Memoir Series of the Center for Systematic Entomology 2: 1–286.

[B1206] Wu S Gong Q Fan K Sun R Xu Y Zhang K (2017) Synergistic effect of entomopathogenic nematodes and thiamethoxam in controlling *Bradysia odoriphaga* Yang and Zhang (Diptera: Sciaridae). Biological Control 111: 53–60. https://doi.org/10.1016/j.biocontrol.2017.05.006

[B1207] Wu S Youngman RR Kok LT Laub CA Pfeiffer DG (2014) Interaction between entomopathogenic nematodes and entomopathogenic fungi applied to third instar southern masked chafer white grubs, *Cyclocephala lurida* (Coleoptera: Scarabaeidae), under laboratory and greenhouse conditions. Biological Control 76: 65–73. https://doi.org/10.1016/j.biocontrol.2014.05.002

[B1208] Yanes-Gómez G Morón MA (2010) Fauna de coleópteros Scarabaeoidea de Santo Domingo Huehuetlán, Puebla, México. Su Potencial como indicadores ecológicos. Acta Zoológica Mexicana (n. s.) 26: 123–145.

[B1209] Yepes-Rodriguez FC Nivia-Jiménez DC Pérez-Betancur E (2013) Lista de algunas especies de la subfamilia Dynastinae (*Aspidolea*, *Cyclocephala*, *Euetheola*, y *Ligyrus*) del Museo Entomológico Francisco Luís Gallego. Boletin del Museo Entomológico Francisco Luís Gallego 5: 20–25.

[B1210] Yildrim E Hoy CW (2003) Interaction between cyromazine and the entomopathogenic nematode *Heterorhabditis bacteriophora* Poinar ‘‘GPS11’’ for control of onion maggot, *Delia antiqua* (Meigen). Crop Protection 22: 923–927. https://doi.org/10.1016/S0261-2194(03)00091-7

[B1211] Young HJ (1986) Beetle pollination of *Dieffenbachia longispatha* (Araceae). American Journal of Botany 73: 931–944. https://doi.org/10.1002/j.1537-2197.1986.tb12133.x

[B1212] Young HJ (1987) Aroid observations: *Philodendron rothschuhianum*. Aroideana 10: 22.

[B1213] Young HJ (1988a) Differential importance of beetle species pollinating *Dieffenbachia longispatha* (Araceae). Ecology 69: 832–844. https://doi.org/10.2307/1941033

[B1214] Young HJ (1988b) Neighborhood size in a beetle pollinated tropical aroid: effects of low density and asynchronous flowering. Oecologia 76: 461–466. https://doi.org/10.1007/BF00377043 10.1007/BF00377043 28312028

[B1215] Young HJ (1990) Pollination and reproductive biology of an understory neotropical aroid. In: Bawa KS, Hadley M (Eds) Reproductive Ecology of Tropical Forest Plants. UNESCO and The Parthenon Publishing Group, Park Ridge, 151–164.

[B1216] Young RM (1992) A new *Cyclocephala* from a Costa Rican cloud forest (Scarabaeidae: Dynastinae). The Coleopterists Bulletin 46: 52–55.

[B1217] Young RM Le Tirant S (2005) A new *Cyclocephala* from montane Colombia (Scarabaeidae: Dynastinae). The Coleopterists Bulletin 59: 267–270. https://doi.org/10.1649/772

[B1218] Young CA Hume DE McCulley RL (2013) Forages and pastures symposium. Fungal endophytes of tall fescue and perennial ryegrass: pasture friend or foe? Journal of Animal Science 2013.91: 2379–2394. https://doi.org/10.2527/jas.2012-5951 10.2527/jas.2012-5951 23307839

[B1219] Youngman RR Midgarden DG Herbert Jr DA Nixon KH Brann DE (1993) Evaluation of preplant method for detecting damage to germinating corn seeds by multiple species of insects. Environmental Entomology 22: 1251–1259. https://doi.org/10.1093/ee/22.6.1251

[B1220] Zenger JT Gibb TJ (2001) Identification and impact of egg predators of *Cyclocephala lurida* and *Popillia japonica* (Coleoptera: Scarabaeidae) in turfgrass. Environmental Entomology 30: 425–430. https://doi.org/10.1603/0046-225X-30.2.425

[B1221] Zhang M Crocker RL Mankin RW Flanders KL Brandhorst-Hubbard JL (2003a) Acoustic identification and measurement of activity patterns of white grubs in soil. Journal of Economic Entomology 96: 1770–1779. https://doi.org/10.1093/jee/96.6.1770 10.1603/0022-0493-96.6.1704 14977106

[B1222] Zhang M Crocker RL Mankin RW Flanders KL Brandhorst-Hubbard JL (2003b) Acoustic estimation of infestations and population densities of white grubs (Coleoptera: Scarabaeidae) in turfgrass. Journal of Economic Entomology 96: 1704–1710. https://doi.org/10.1093/jee/96.6.1704 10.1603/0022-0493-96.6.1770 14977114

[B1223] Zimsen E (1964) The type material of I. C. Fabricius. Munksgaard, Copenhagen, 656 pp.

